# Innovative approach for high-throughput exploiting sex-specific markers in Japanese parrotfish *Oplegnathus fasciatus*

**DOI:** 10.1093/gigascience/giae045

**Published:** 2024-07-19

**Authors:** Yongshuang Xiao, Zhizhong Xiao, Lin Liu, Yuting Ma, Haixia Zhao, Yanduo Wu, Jinwei Huang, Pingrui Xu, Jing Liu, Jun Li

**Affiliations:** Center for Ocean Mega-Science, Key Laboratory of Breeding Biotechnology and Sustainable Aquaculture (CAS), Institute of Oceanology, Chinese Academy of Sciences, Qingdao, 266071, China; Laboratory for Marine Biology and Biotechnology, Qingdao Marine Science and Technology Center, Qingdao, 266071, China; Shandong Province Key Laboratory of Experimental Marine Biology, Institute of Oceanology, Chinese Academy of Sciences, Qingdao, 266071, China; Center for Ocean Mega-Science, Key Laboratory of Breeding Biotechnology and Sustainable Aquaculture (CAS), Institute of Oceanology, Chinese Academy of Sciences, Qingdao, 266071, China; Laboratory for Marine Biology and Biotechnology, Qingdao Marine Science and Technology Center, Qingdao, 266071, China; Shandong Province Key Laboratory of Experimental Marine Biology, Institute of Oceanology, Chinese Academy of Sciences, Qingdao, 266071, China; Weihai Hao Huigan Marine Biotechnology Co., Weihai, 26449, China; Wuhan Frasergen Bioinformatics Co., Ltd, East Lake High-Tech Zone, Wuhan, 430073, China; Center for Ocean Mega-Science, Key Laboratory of Breeding Biotechnology and Sustainable Aquaculture (CAS), Institute of Oceanology, Chinese Academy of Sciences, Qingdao, 266071, China; Laboratory for Marine Biology and Biotechnology, Qingdao Marine Science and Technology Center, Qingdao, 266071, China; Shandong Province Key Laboratory of Experimental Marine Biology, Institute of Oceanology, Chinese Academy of Sciences, Qingdao, 266071, China; Center for Ocean Mega-Science, Key Laboratory of Breeding Biotechnology and Sustainable Aquaculture (CAS), Institute of Oceanology, Chinese Academy of Sciences, Qingdao, 266071, China; Laboratory for Marine Biology and Biotechnology, Qingdao Marine Science and Technology Center, Qingdao, 266071, China; Shandong Province Key Laboratory of Experimental Marine Biology, Institute of Oceanology, Chinese Academy of Sciences, Qingdao, 266071, China; Center for Ocean Mega-Science, Key Laboratory of Breeding Biotechnology and Sustainable Aquaculture (CAS), Institute of Oceanology, Chinese Academy of Sciences, Qingdao, 266071, China; Laboratory for Marine Biology and Biotechnology, Qingdao Marine Science and Technology Center, Qingdao, 266071, China; Shandong Province Key Laboratory of Experimental Marine Biology, Institute of Oceanology, Chinese Academy of Sciences, Qingdao, 266071, China; Center for Ocean Mega-Science, Key Laboratory of Breeding Biotechnology and Sustainable Aquaculture (CAS), Institute of Oceanology, Chinese Academy of Sciences, Qingdao, 266071, China; Laboratory for Marine Biology and Biotechnology, Qingdao Marine Science and Technology Center, Qingdao, 266071, China; Shandong Province Key Laboratory of Experimental Marine Biology, Institute of Oceanology, Chinese Academy of Sciences, Qingdao, 266071, China; Center for Ocean Mega-Science, Key Laboratory of Breeding Biotechnology and Sustainable Aquaculture (CAS), Institute of Oceanology, Chinese Academy of Sciences, Qingdao, 266071, China; Laboratory for Marine Biology and Biotechnology, Qingdao Marine Science and Technology Center, Qingdao, 266071, China; Shandong Province Key Laboratory of Experimental Marine Biology, Institute of Oceanology, Chinese Academy of Sciences, Qingdao, 266071, China; Center for Ocean Mega-Science, Key Laboratory of Breeding Biotechnology and Sustainable Aquaculture (CAS), Institute of Oceanology, Chinese Academy of Sciences, Qingdao, 266071, China; Center for Ocean Mega-Science, Key Laboratory of Breeding Biotechnology and Sustainable Aquaculture (CAS), Institute of Oceanology, Chinese Academy of Sciences, Qingdao, 266071, China; Laboratory for Marine Biology and Biotechnology, Qingdao Marine Science and Technology Center, Qingdao, 266071, China; Shandong Province Key Laboratory of Experimental Marine Biology, Institute of Oceanology, Chinese Academy of Sciences, Qingdao, 266071, China

**Keywords:** large-segment insertion/deletion, bulk primers design, e-PCR technology, *Oplegnathus fasciatus*, high-throughput identification

## Abstract

**Background:**

The use of sex-specific molecular markers has become a prominent method in enhancing fish production and economic value, as well as providing a foundation for understanding the complex molecular mechanisms involved in fish sex determination. Over the past decades, research on male and female sex identification has predominantly employed molecular biology methodologies such as restriction fragment length polymorphism, random amplification of polymorphic DNA, simple sequence repeat, and amplified fragment length polymorphism. The emergence of high-throughput sequencing technologies, particularly Illumina, has led to the utilization of single nucleotide polymorphism and insertion/deletion variants as significant molecular markers for investigating sex identification in fish. The advancement of sex-controlled breeding encounters numerous challenges, including the inefficiency of current methods, intricate experimental protocols, high costs of development, elevated rates of false positives, marker instability, and cumbersome field-testing procedures. Nevertheless, the emergence and swift progress of PacBio high-throughput sequencing technology, characterized by its long-read output capabilities, offers novel opportunities to overcome these obstacles.

**Findings:**

Utilizing male/female assembled genome information in conjunction with short-read sequencing data survey and long-read PacBio sequencing data, a catalog of large-segment (>100 bp) insertion/deletion genetic variants was generated through a genome-wide variant site-scanning approach with bidirectional comparisons. The sequence tagging sites were ranked based on the long-read depth of the insertion/deletion site, with markers exhibiting lower long-read depth being considered more effective for large-segment deletion variants. Subsequently, a catalog of bulk primers and simulated PCR for the male/female variant loci was developed, incorporating primer design for the target region and electronic PCR (e-PCR) technology. The Japanese parrotfish (*Oplegnathus fasciatus*), belonging to the Oplegnathidae family within the Centrarchiformes order, holds significant economic value as a rocky reef fish indigenous to East Asia. The criteria for rapid identification of male and female differences in Japanese parrotfish were established through agarose gel electrophoresis, which revealed 2 amplified bands for males and 1 amplified band for females. A high-throughput identification catalog of sex-specific markers was then constructed using this method, resulting in the identification of 3,639 (2,786 INS/853 DEL, ♀ as reference) and 3,672 (2,876 INS/833 DEL, ♂ as reference) markers in conjunction with 1,021 and 894 high-quality genetic sex identification markers, respectively. Sixteen differential loci were randomly chosen from the catalog for validation, with 11 of them meeting the criteria for male/female distinctions. The implementation of cost-effective and efficient technological processes would facilitate the rapid advancement of genetic breeding through expediting the high-throughput development of sex genetic markers for various species.

**Conclusions:**

Our study utilized assembled genome information from male and female individuals obtained from PacBio, in addition to data from short-read sequencing data survey and long-read PacBio sequencing data. We extensively employed genome-wide variant site scanning and identification, high-throughput primer design of target regions, and e-PCR batch amplification, along with statistical analysis and ranking of the long-read depth of the variant sites. Through this integrated approach, we successfully compiled a catalog of large insertion/deletion sites (>100 bp) in both male and female Japanese parrotfish.

## Introduction

Sexual dimorphism is common in the animal kingdom, but the mechanisms of sex determination are diverse [1, 2]. Thus, sex-determination research has always been a topic of interest in biology. Fish occupy a key position in the vertebrate evolutionary system, and because of their wide distribution, large number of species, and diverse sex-determination mechanisms, they are an important target for the study of sex-determination mechanisms [3–9]. However, most fish have a low degree of differentiation between the sex chromosomes, which is difficult to detect from the morphology of the chromosomes [10]. The development of sex-specific molecular markers and sex-control biotechnology has provided an important technical way to increase fish production and economic value and has also laid the foundation for deciphering the molecular mechanism of fish sex determination [11]. Researchers have succeeded in developing sex-specific or correlated molecular markers in many fish species using a variety of techniques, including simple sequence repeat (SSR), random amplification of polymorphic DNA (RAPD), amplified fragment length polymorphism (AFLP), single nucleotide polymorphism (SNP), and insertions/deletions (indels) [5, 6, 12–17]. However, these methods for obtaining sex-specific markers have many problems, such as low throughput, single markers, cumbersome development and experimental procedures, high development costs, low amplification efficiency, and inconvenient on-site detection. The first issue to be addressed was to establish a high-throughput and easy-to-detect marker procedure for sex-specific identification in the investigation of sex dimorphism, mechanisms of sex differentiation, and development and use of sex-producing traits.

In recent years, with the rapid development of genome sequencing technology and the significant decrease in the cost of sequencing, it has become possible to perform whole-genome sequencing of important cultured species. Rapid developments in next-generation sequencing (NGS) technologies have created significant opportunities for the discovery and use of species germplasm resources [4]. In 2002, the genome sequencing of *Takifugu rubripes* was successfully accomplished, marking it as the pioneering model fish species to undergo complete genome sequencing [18]. Subsequently, over the course of nearly 16 years, the whole-genome sequencing of more than 40 fish species, including *Oryzias latipes, Gasterosteus aculeatus, Pelteobagrus fulvidraco, Mola mola, Nothobranchius furzeri*, and *Salmo salar*, has been carried out on a national and international scale [19–30]. Over the span of approximately 16 years until 2018, the majority of fish genomes were sequenced utilizing second-generation sequencing technology, specifically Illumina [4, 18–21]. Despite the high-throughput capabilities of second-generation sequencing, the relatively short sequencing read lengths pose challenges in obtaining precise genome maps and accurate information on chromosome structural variation. This limitation is especially pronounced in genomes with high complexity and repetitive regions.

At present, third-generation sequencing technology with high precision of long fragments represented by single-molecule real-time sequencing has matured, which includes Pacific Biosciences’ (PacBio) single-molecule real-time sequencing technology (SMRT), Oxford Nanopore’s single-molecule nanopore sequencing technology, and so on. Among them, PacBio RS sequencing technology is the most widely used and commercialized core third-generation sequencing technology and has been widely used in the genome assembly of several species, such as *Oropetium thomaeum* and *Lates calcarifer* [31–33]. The initial assembly of the human telomere-to-telomere (T2T) CHM13 genome, utilizing PacBio HiFi and Oxford Nanopore ultralong-read sequencing, achieved a contig N50 of 154.26 Mb, resulting in a seamless assembly that fills a crucial gap in the human DNA genetic code map [31, 34–36]. In addition, Hi-C technology based on next-generation sequencing technology has gradually become a favorable tool for chromosome assembly [37]. With the rapid development of sequencing technology and the improvement of assembly technology, the genome integrity and accuracy have been greatly improved, especially in marine fishes, and the assembly now obtained contig N50 up to the level of 28 Mb for *Sciaenops ocellatus* [28]. Thus, the development of next-generation sequencing technologies has made it possible to exploit high-throughput sex-specific markers in high-precision assembled genome data.

*Oplegnathus fasciatus* (Temminck & Schlegel, 1844, Fishbase ID: 1709; NCBI:txid881956; marinespecies.org:taxname:277,892) is a rocky reef fish distributed in a wide range of shallow waters around Korea, Japan, China, and Hawaii [38–41]. Japanese parrotfish have become an important fishery resource for offshore cage culture and fish stocking in marine ranches in China, Japan, and Korea [40, 41]. Japanese parrotfish have been a commercially important species for sashimi production and recreational angling, with engineered aquaculture in China yielding ex-factory prices of up to US$30/kg [41]. Meanwhile, Japanese parrotfish are characterized by a multiple X_1_X_1_X_2_X_2_/X_1_X_2_Y sex chromosome system [42–45]. Sexual dimorphism has been observed in Japanese parrotfish, with males growing faster than females, especially at weights greater than 500 g [7, 8, 44, 45]. Therefore, the breeding of new strains with a male predominance has become one of the goals for the future breeding of Japanese parrotfish. The development of appropriate genetic sex markers was a prerequisite for achieving a new variety of Japanese parrotfish with male predominance. Currently, available markers for the genetic characterization of Japanese parrotfish are extremely limited and have mainly been identified by AFLP, SSR, and Illumina technologies, which constrains the process of exploiting new varieties of Japanese parrotfish [46–48]. The completion of the female and male genomes of Japanese parrotfish has enabled high-throughput development of large segments to visualize genetic sex markers from a genome-wide perspective.

In this work, a catalog of genomic differences between females and males has been constructed based on the assembled genome information of Japanese parrotfish by using bidirectional whole-genome sequence alignment [49, 50]. Using the difference catalogs, we obtain datasets of insertion and deletion variant sites larger than 100 bp by screening and use short-read sequencing data surveys from genome sequencing and long-read PacBio sequencing data to test the confidence of large segmental variant sites [51]. Utilizing the Japanese parrotfish sequence tagged sites as a foundation, we employ e-simulation amplification technology to conduct a genome-wide PCR assay aimed at identifying single amplified-banded variant loci between female and male genomes [51]. Then, we perform PCR amplification and agarose gel electrophoresis for the mutant loci detected by simulated PCR. Since Japanese parrotfish exhibit an X_1_X_1_X_2_X_2_/X_1_X_2_Y sex chromosome system, a single primer pair amplifies 2 bands in males and 1 band in females.

**Figure 1:**
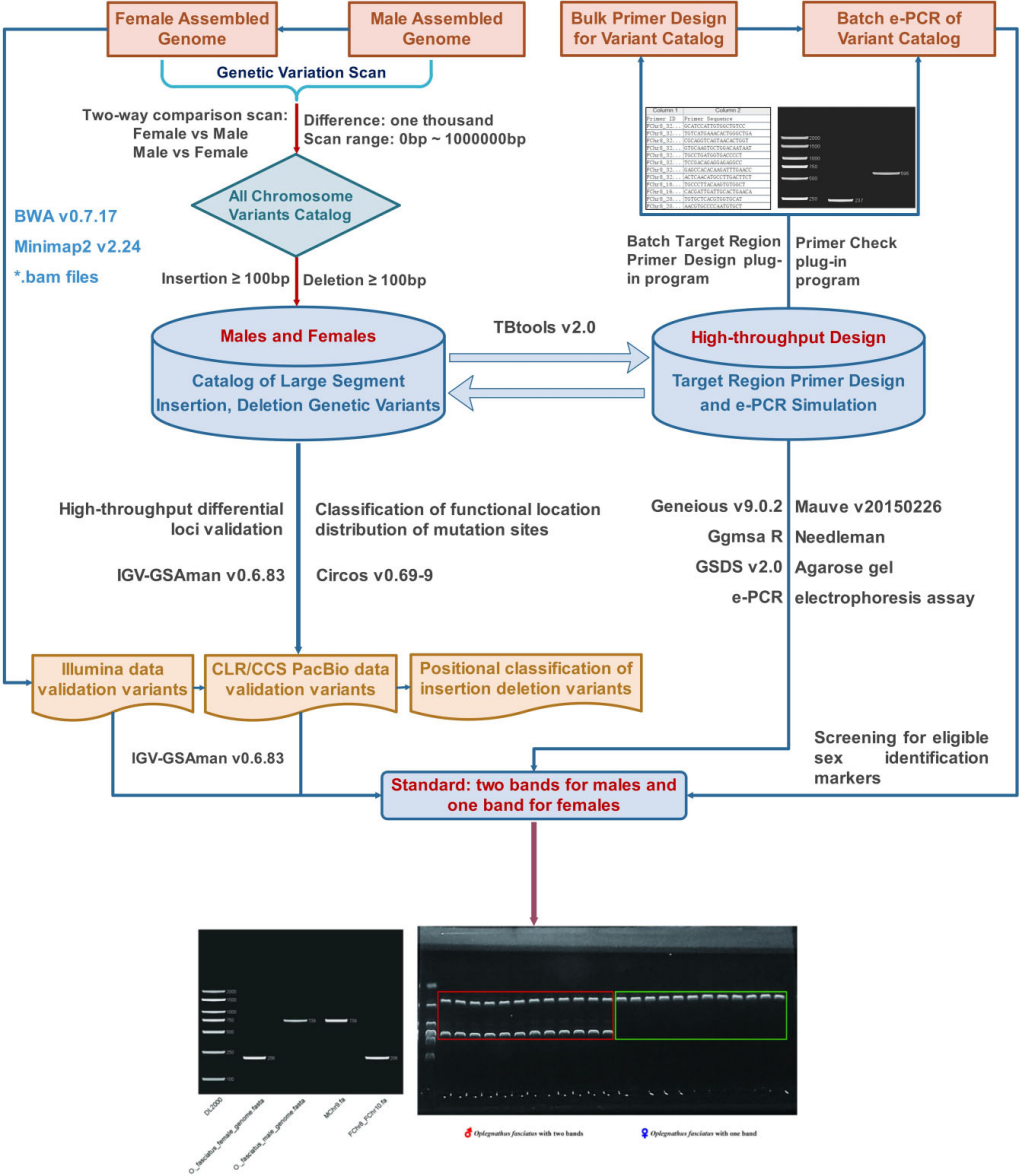
High-throughput exploitation process of genetic sex markers for Japanese parrotfish.

The article has been organized as follows: First, a fast scanning method for female and male assembled genome variant loci is introduced, and an insertion/deletion dataset of large segments has been constructed. Using the constructed insertion/deletion dataset of large fragments, high-throughput candidate molecular marker loci for female and male genetic sex identification of Japanese parrotfish are identified. Then, the new technique of PCR electronic simulation with bulk amplification is used to perform simulated amplification of high-throughput candidate molecular marker loci to screen primers for the single amplification of the female and male genomes with amplification differences. Finally, a high-throughput precision marker dataset for male and female genetic sex identification is obtained and tested by agarose gel electrophoresis (Fig. 1).

## Materials and Methods

### Sample collection, sequencing, and genome quality

Male and female Japanese parrotfish captured from Qingdao, Shandong Province (Yellow Sea), were utilized for genome sequencing and assembly. High-quality genomic DNA was extracted from fresh muscle tissue and blood samples from pairs of male and female Japanese parrotfish using high-throughput sequencing on the Illumina platform (Illumina, Inc.) and PacBio Sequel (Pacific Biosciences of California) [44, 45]. Genomic DNA from fresh muscle tissue and blood samples of male and female Japanese parrotfish pairs was extracted to construct short insert libraries (300–350 bp) for males and females, respectively, using standard protocols provided by the Illumina HiSeq X Ten platform (Illumina, Inc.; RRID:SCR_016385). Raw reads totaling approximately 90.7 Gb and 63.9 Gb were generated. After removing splice-containing sequences and low-quality and redundant reads, approximately 80.8 Gb and 56.6 Gb of pure data were obtained from the genomes of males and females, respectively, corresponding to Q20 averages of 97.55% and 97.60%, respectively (Table 1). Genomic DNA libraries (20 kb) for male and female fish were then constructed using the PacBio Sequel platform (Pacific Biosciences of California) (Table 1). Sequencing was performed on the PacBio Sequel platform using the Sequel Binding Kit 2.0, Sequel Sequencing Kit 2.1, and Sequel SMRT Cell 1 M v2. Seven and 5 SMRT cells were used for sequencing the male and female genomes, respectively, with SMRT LINK 5.0 used to filter the raw data from the zero-mode waveguide. We obtained 62.8 Gb and 39.8 Gb of long-read length PacBio sequencing data for female (accession SRP160016) and male (accession SRP220007) genome assembly, respectively [44, 45]. The female and male Japanese parrotfish genome were *de novo* assembled using Canu (RRID:SCR_015880) (v 1.4) with corrected error rate set to 0.040, followed by redundancy removal using Redundans (v 0.13c) (minCoverage = 15), genome polishing with Arrow tool in SMRT Link 5.0, and final assembly refinement with Pilon (RRID:SCR_014731) using Illumina NGS short reads from the genome surveys. Based on the male and female draft genomes and Hi-C technology, we obtained high-quality genomes at the chromosome level of female (768.8 Mb, accession SUB14064808) and male (762.2 Mb, accession SUB14065793) Japanese parrotfish, and the contig N50 and scaffold N50 of the male and female fish genomes reached 2.13 Mb/2.18 Mb and 33.5 Mb/32.4 Mb, respectively [44, 45]. To assess the genomic completeness of assembled male and female Japanese parrotfish, we subjected the assembled sequences to BUSCO (RRID:SCR_015008) (v 5) evaluation (BUSCO, actinopterygii_odb10), with complete BUSCOs assessed values up to 98.2% and 98.0% for male and female genomes, respectively (Table 1).

**Table 1:** Parameters for genome sequencing and assembly quality assessment of chromosome-level genomes of male and female Japanese parrotfish

Illumina platform (short-read sequencing data survey)		
	Male Japanese parrotfish	Female Japanese parrotfish
Reads number	432,091,798	613,163,122
Raw data (bp)	63,910,932,339	90,702,298,813
Clean data (bp)	56,617,669,600	80,887,039,002
Read length (bp)	148,147	148,147
Q20 (%)	97.9, 97.3	98.0, 97.1
Q30 (%)	93.8, 92.0	94.1, 91.4
PacBio Sequel platform (long-read PacBio sequencing data)		
Polymerase read number	3,674,737	4,760,021
Polymerase read bases (bp)	39,844,228,109	62,877,786,278
Average polymerase read length (bp)	10,847	13,219
Polymerase read N50 (bp)	18,850	22,964
Subreads number	4,711,720	6,689,662
Total bases of subreads (bp)	39,793,172,442	62,783,888,050
Average subread length (bp)	8,448	9,397
Length of genome (bp)	762,267,613	768,808,243
Number of contigs	1,355	1,372
Contigs N50 (bp)	2,183,645	2,130,780
Number of scaffolds	23	24
Scaffold N50 (bp)	32,431,321	33,548,962
Genome coverage (×)	251.1	314.6
Complete BUSCOs (%)	98.2	98.0

Male and female genomic quality parameters of Japanese parrotfish [44, 45].

### Large-fragment variant loci scanning and identification

We employed the genome variant site scanning plugin with accompanying parameters (diff: oneInThousand, VarRange: 0 ∼ 1,000,000 bp, BatchSize: 500 bp, Min Align Length for Cov Calc 10,000 bp, Min Align Length for Var Calling: 50,000 bp) in TBtools (RRID:SCR_023018) (v 2.0) software to conduct bidirectional comparisons of the complete genomic sequences of male and female organisms [51, 52]. Additionally, we established catalogs containing genomic disparities between males and females. To classify base substitution, insertion, and deletion sites, as well as to screen large insertion/deletion sites (>100 bp) against a differential catalogs, we utilized the Table Row Extract program. In order to further evaluate the stability and accuracy of the dataset containing large-segment insertion/deletion sites, we employed SyRI software (python3 syri_env/bin/syri -c out.filtered.coords -d out.filtered.delta -r FM.fasta -q genome.format.fa –nosnp) to autonomously detect variant sites (≥100 bp) in the assembled genomes of both male and female individuals and subsequently compared these sites with our existing dataset of variant sites. Additionally, we utilized BWA (v 0.7.8) (bwa-0.7.17/bwa mem -t 6 -SP genome.fa M2_1P.fq.gz M2_2P.fq.gz | samtools [RRID:SCR_005227] view -bS > alignment.bam) and Minimap2 (v 2.24) (minimap2 –ax map-pb –t 8 genome.fasta M1.subreads.fasta > M1.subreads.sam) software to align the short-read sequencing data generated from the Illumina platform and the long-read sequencing data obtained from the PacBio platform to the male and female genomes, respectively, in order to validate the large-segment variant sites [53, 54]. To further improve the exploitation efficiency of sex-specific markers based on long reads, we calculated the read depth of long reads of insertion/deletion sites and then ranked the insertion/deletion site markers based on the depth of long reads; for large-segment deletion variants, the lower the depth of long reads, the more effective the markers were, so we constructed a frequency map of the distribution of the depth of long reads of the large-segment deletion sites of the male and female genomes and then classified the markers. Subsequently, employing the Batch Target Region Primer Design plug-in and electronic PCR (e-PCR) plug-in in TBtools (v 2.0) software, we designed primers for high-throughput amplification and screened them for single amplification bands at the insertion and deletion sites specific to the male and female genomes [51]. To identify the origin of large-segment insertion/deletion sites, we evaluated whether the variant sites in the dataset were transposable elements by integrating RepeatModeler (RRID:SCR_015027) (v 2.0.1) and RepeatMasker (RRID:SCR_012954) (v 4.1.2) software. The results of transposable element determination obtained by the 2 software were combined and the duplicates were removed for all transposable elements identification results. At the same time, we utilized TRF (RRID:SCR_022193) (v 4.09.1) software to evaluate whether the variant sites in the dataset were tandem repeats.

The positional distribution of female and male genetic variant loci on the genome was depicted using Circos (RRID:SCR_011798) (v 0.69–9) software [55]. Homology comparison and visualization of insertion/deletion sites with male and female linear genomes were conducted using a combination of Geneious (RRID:SCR_010519) (v 9.0.2) software and the Mauve program (RRID:SCR_012852) (v 20,150,226) [56, 57]. The comparison of genetic sex marker sequences with the Global Alignment standard was performed using the Needleman Wunsch plug-in in TBtools (v 2.0) [51]. Additionally, the visualization of genetic sex marker variant site regions was accomplished using the Ggmsa program [58]. GSDS (v 2.0) software was utilized to illustrate the location of the variant site within the functional region of the gene [59]. A total of 24 tails from both male and female Japanese parrotfish specimens were collected and distinguished through gonadal histology. Simultaneously, we gathered 93 tails from wild population samples of Japanese parrotfish along the coasts of Zhejiang and Shandong Provinces, China, to verify the efficacy of sex markers across various geographic regions. The reliability of the genetic sex markers was assessed by randomly identifying male and female populations using e-PCR in conjunction with agarose gel electrophoresis.

## Results

### Construction of insertion/deletion variant loci datasets for large segments

**Figure 2:**
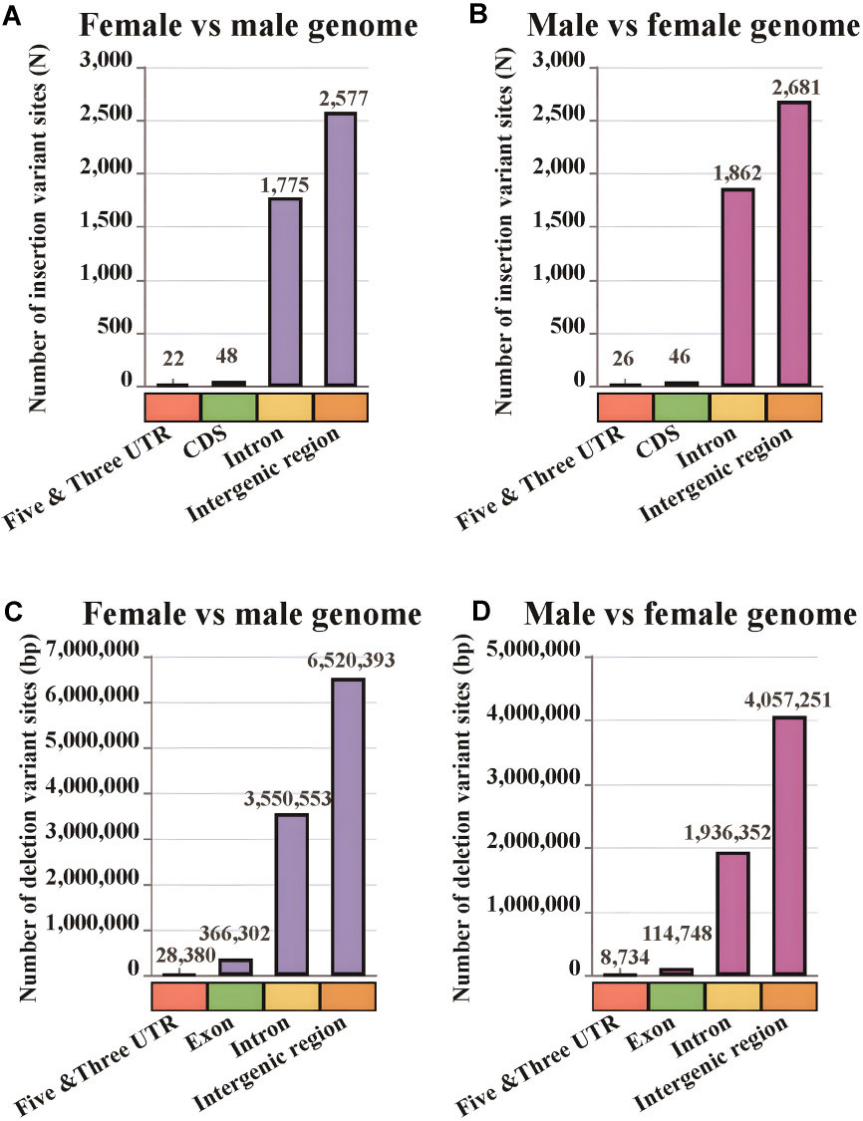
Distribution statistics of insertion and deletion fragment (>100 bp) positions in genomic functional regions of Japanese parrotfish. (A) Distribution statistics of insertion variant loci using the female fish genome as a reference. (B) Distribution statistics of insertion variant loci utilizing the male fish genome as a point of reference. (C) Distribution statistics of deletion variant length using the female fish genome as a reference. (D) Distribution statistics of deletion variant loci using the male fish genome as a reference. The colors red, green, yellow, and orange represent the UTR, CDS, intron, and intergenic regions, respectively. The frequency of insertions (A, B) was quantified based on the number of events, while deletions (C, D) were characterized by their length in nucleotides.

**Figure 3:**
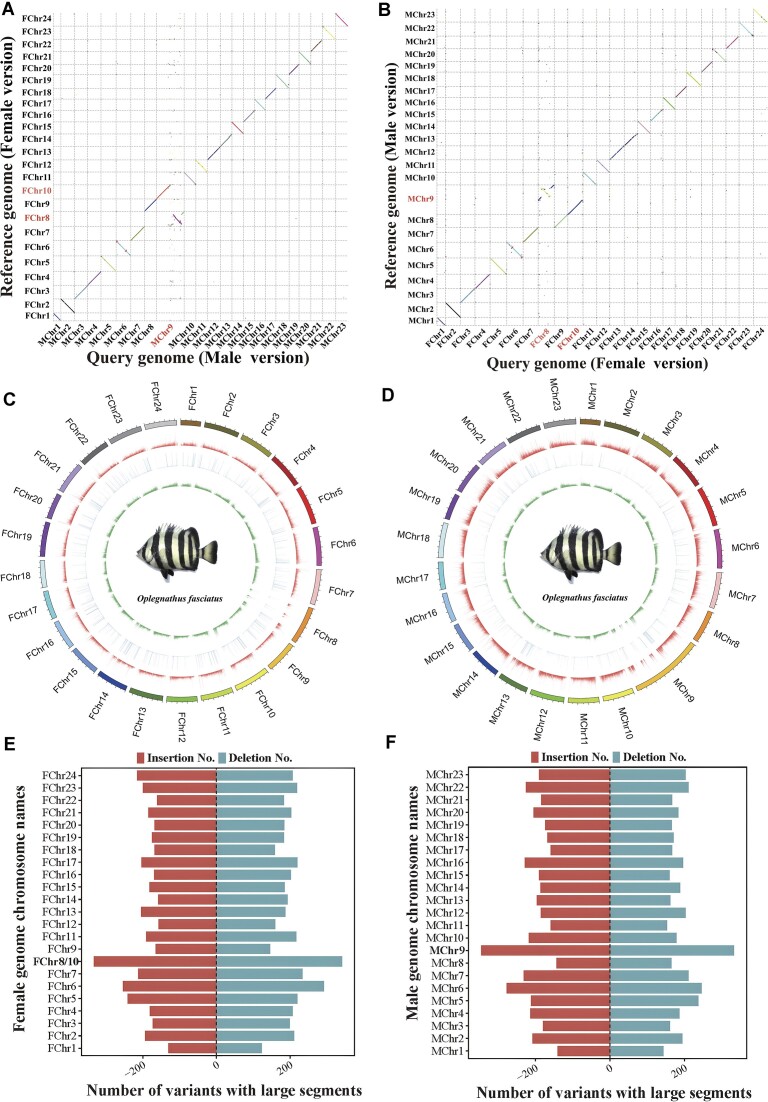
The covariance of genomic sequence differences between the male and female genomes of Japanese parrotfish and distribution statistics of variant sites. (A) The female genome (reference) of *Japanese parrotfish* vs. the male genome for homology comparison using the minimap2 software with a 5,000-bp sliding window. The female genome is represented on the vertical axis, with female chromosomes 8 and 10 homologous to male chromosome 9. (B) The comparison of homology between the male genome of Japanese parrotfish and the female genome was conducted using the minimap2 software with a 5,000-bp sliding window. The male genome is represented on the vertical axis, revealing homology between male chromosome 9 and female chromosomes 8 and 10. (C) Distribution statistics of variant loci using the female fish genome as a reference. Insertions (>100 bp) are summarized by number of events. Deletions (>100 bp) are summarized by length in nucleotides. From outer to inner circles are the chromosome name, positional distribution of insertion sites, base length distribution of deletion sites, and base substitution distribution, respectively. (D) Distribution statistics of variant loci using the male fish genome as a reference. (E, F) Dual-valued histograms corresponding to the Table 3 show the number of insertion/deletion sites per chromosome with reference to the female (4,422 insertion sites, 4,675 deletion sites) and male (4,615 insertion sites, 4,385 deletion sites) genomes, respectively, where chromosome 9 in males was homologous to chromosomes 8 and 10 in females. Insertion sites are represented by negative values, and deletion sites are represented by positive values.

Using the female genome as a reference, we obtained 1,919,620 male and female differential variant sites with unfiltered data, including 191,915 insertion sites, 325,620 deletion sites, and 1,402,085 nucleotide substitutions (Supplementary Female_reference_all_variation_Set.xls). To screen suitable field-actionable markers for large-band genetic sex identification, we screened this insertion/deletion variant site catalog for variants larger than 100 bp and obtained 4,422 insertion sites with a differential sequence size of 5,908,147 bp, of which 0.11% were located in 5′ UTRs, 0.38% in 3′ UTRs, 40.14% in intronic regions, 1.08% in CDS regions, and 58.28% in intergenic regions (Fig. 2, Supplementary Fig. S1, Supplementary Female_reference_INS/DEL100_Set.xlsx). In addition, we obtained 4,675 deletion sites with a differential sequence size of 10,465,628 bp (Supplementary Female_reference_INS/DEL100_Set.xlsx). As the deletion variant sites spanned multiple genomic functional regions, we performed statistical analysis of the actual locations where the deletion bases were located, with 0.08% located in 5′ UTRs, 0.19% in 3′ UTRs, 33.93% in intronic regions, 3.50% in CDS regions, and 62.30% in intergenic regions (Fig. 2, Table 2). The highest number of chromosomal mutations occurred in female fish chromosomes 8 and 10, with 333 and 341 insertion and deletion sites, respectively (Fig. 3).

**Table 2:** Statistics of the positional distribution of insertion/deletion variants in the male and female genomes of Japanese parrotfish

	Female genome as a reference		Male genome as a reference	
Type of gene function region	Insertion variant loci No. (>100 bp)	Deletion variant length (>100 bp)	Insertion variant loci No. (>100 bp)	Deletion variant length (>100 bp)
5′ UTR	5	7,859	6	2,482
3′ UTR	17	20,521	20	6,252
CDS	48	366,302	46	114,748
Intron	1,775	3,550,553	1,862	1,936,352
Intergenic region	2,577	6,520,393	2,681	4,057,251
Total	4,422	10,465,628	4,615	6,117,085

Insertions (>100 bp) were summarized by number of events. Deletions (>100 bp) were summarized by length in nucleotides.

Using the male Japanese parrotfish genome as a reference, 1,926,295 female/male differential variant sites were obtained with unfiltered data, including 326,823 insertion sites, 192,672 deletion sites and 1,406,800 nucleotide substitution sites (Supplementary Female_reference_all_variation_Set.xls). Through large-fragment screening (>100 bp), we obtained 4,615 insertion sites with a differential sequence size of 10,100,983 bp, of which 0.13% were located in 5′ UTRs, 0.43% in 3′ UTRs, 40.35% in intronic regions, 1.00% in CDS regions, and 58.09% in intergenic regions (Fig. 2, Supplementary Fig. S1, Table 2, Supplementary Male_reference_INS100_Set.xlsx). The chromosome with the highest number of mutations was chromosome 9 in male Japanese parrotfish, in which the numbers of insertion and deletion sites were 345 and 333, respectively (Fig. 3); male chromosome 9 was homologous to chromosomes 8 and 10 of female Japanese parrotfish. Male chromosome 6 had the second highest number of mutations, with 289 insertion sites and 256 deletion sites (Fig. 3).

**Figure 4:**
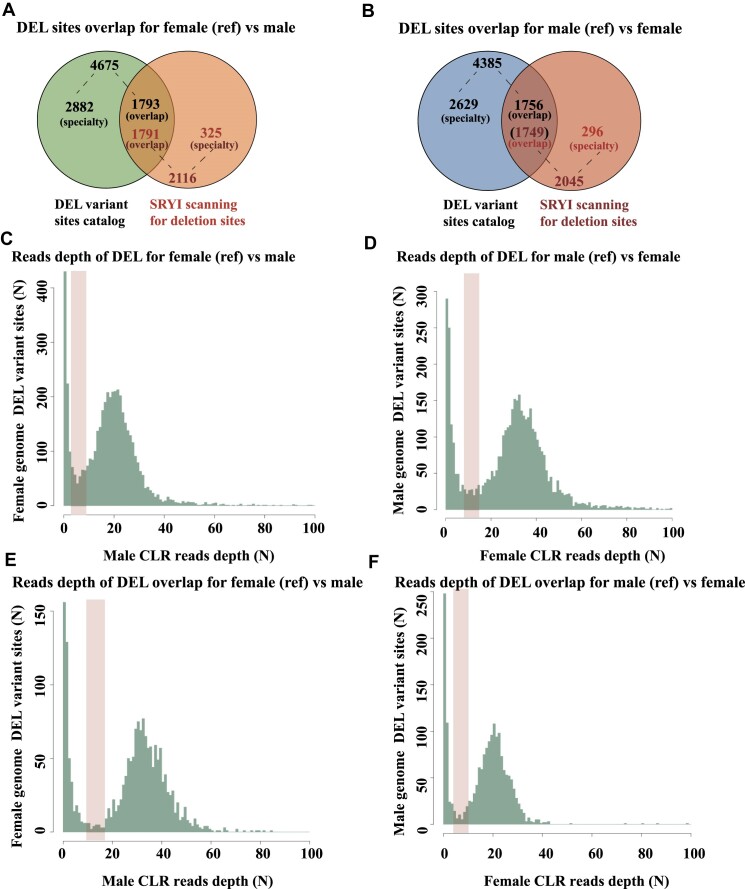
Intersection results of 2 variant site-scanning methods and ranking of variant sites with long-read depth frequency distributions. (A) Venn results of deletion locus data were obtained based on TBtools and SyRI software using the female fish genome as a reference, and 1,791 out of 2,116 deletion loci obtained based on SyRI software were shared deletion variant loci. (B) Utilizing the male fish genome as a point of reference for acquiring deletion site data in the context of Venn analysis, it was found that 1,749 out of the 2,045 deletion sites identified through the use of the SyRI software were common deletion variant sites. (C) Depth distribution of CLR reads in male fish using all female genomic deletion locus (>100 bp) information as a reference, with the area where high-quality male and female genetic marker information was located before the orange labeled trough position (∼5.4). (D) The depth distribution of CLR reads in females was analyzed by utilizing male genomic deletion sites as a reference (>100 bp), focusing on the region preceding the orange labeled trough position (∼12.5) where high-quality male and female genetic marker information was present. (E, F) Depth distribution of CLR reads in males and females based on intersecting male and female genomic deletion site information (>100 bp), respectively.

The SyRI software was further utilized to assess variant sites (>100 bp) between the male and female reference genomes of Japanese parrotfish. By utilizing the male genome as a reference for comparison with the female genome, a total of 2,045 deletion sites (>100 bp) and 2,110 insertion sites (>100 bp) were identified. Additionally, comparison of the female genome with the male genome as a reference revealed 2,116 large-segment deletion sites (>100 bp) and 2,055 large-segment insertion sites (>100 bp). Considering the long-read length depth calculations and ranking large segments of deletion sites were beneficial when utilizing short-read sequencing data and long-read PacBio sequencing data to analyze visual differences between male and female genomes. Our study revealed that 1,791 deletion sequence tag sites identified through SyRI scanning overlapped with deletion sites identified in the female fish reference genome, accounting for 84.64% of the total 2,116 deletion sites identified (Fig. 4A, Table 3). Meanwhile, the SyRI scanning method identified 1,749 deletion STS that intersected with deletion sites in the reference genome of male fish, representing 85.53% of the total 2,045 deletion sites (Fig. 4B, Table 3). In the context of large-deletion variants, a correlation was observed between the long-read depth and the effectiveness of markers. A frequency distribution map was created for the long-read depths of large-deletion loci within male and female genomes, followed by marker categorization. The results indicated that markers with long-read depths below 5.48 (895, ♀ as reference) and 12.50 (1,021, ♂ as reference) demonstrated high recognition rates as sex markers, based on the male and female genomes as reference (Fig. 4C–F). The findings further indicated that a substantial proportion of deletion sequence tag sites referencing the female genome were situated in transposable element regions (78.44%, *n* = 3,667) and tandem repeat sequence regions (33.67%, *n* = 1,574). Similarly, statistical analysis revealed that a notable percentage of deletion sequence tag sites referencing the male genome was linked to transposable element regions (77.08%, *n* = 3,380) and tandem repeat regions (31.90%, *n* = 1,399) (Table 4).

**Table 3:** Statistical cross-validation of multiple scanning of genome-wide large insertion/deletion sequence marker sites

Reference genome	Female vs. male			Male vs. female		
Mutation type	Insertion		Deletion	Insertion		Deletion
Variant length (>100 bp)	4,422		4,675	4,615		4,385
Chromosomes	FChr1	131	123	MChr1	140	143
	FChr2	194	211	MChr2	208	194
	FChr3	173	199	MChr3	179	161
	FChr4	181	207	MChr4	214	186
	FChr5	241	220	MChr5	212	237
	FChr6	254	292	MChr6	277	246
	FChr7	212	234	MChr7	231	211
	FChr8/10	333	341	MChr8	143	165
	FChr9	165	146	MChr9	345	333
	FChr11	191	217	MChr10	217	179
	FChr12	157	160	MChr11	159	153
	FChr13	205	187	MChr12	185	203
	FChr14	158	193	MChr13	196	162
	FChr15	182	185	MChr14	186	188
	FChr16	169	202	MChr15	190	160
	FChr17	204	220	MChr16	228	196
	FChr18	168	159	MChr17	159	167
	FChr19	175	183	MChr18	168	171
	FChr20	168	184	MChr19	174	166
	FChr21	185	203	MChr20	205	183
	FChr22	161	183	MChr21	184	167
	FChr23	200	219	MChr22	225	211
	FChr24	215	207	MChr23	190	203
SyRI-based scanning of male and female mutant sites (≥100 bp)		2,116		2,045		
Intersection of the 2 methods		1,791 (84.64%)		1,749 (85.53%)		

**Table 4:** Statistics of transposable elements and tandem repeat sequences attributed to large deletion sequence tag sites obtained with male and female reference genomes

Reference genome	Mutation type	Transposable elements	Tandem repeat	Total
Female vs. male	Deletion	3,667 (78.44%)	1,574 (33.67%)	4,675
Male vs. female	Deletion	3,380 (77.08%)	1,399 (31.90%)	4,385

### High-throughput primer design and e-PCR bulk simulation for target regions

The identified large insertion/deletion variants were utilized as target regions for sex identification in both male and female genomes. High-throughput primers were designed to generate primer catalogs for the specified target markers. A total of 4,016 and 1,314 primer pairs were obtained by high-throughput primer design for the insertion/deletion target regions, respectively, using the female genome as the reference (Table 5). To further test the accuracy and uniqueness of the high-throughput primers across the genome, we performed genome-wide electronic simulated amplification of the obtained primer pairs by using e-PCR. A total of 2,786 and 853 genome-wide single amplification band primer pairs were identified through screening of 5,735 primer pairs and 957 primer pairs with single bands, respectively, resulting in amplification products ranging from 100 to 2,000 bp (Table 5, Supplementary Female_Male_INS/DEL100_PrimerSet.xlsx). Utilizing the Japanese parrotfish male genome as a point of reference, 4,131 and 1,199 primer pairs were obtained to target insertions/deletions of specific variant sites, respectively (Table 2, Supplementary Female_Male_INS/DEL100_PrimerSet.xlsx). Notably, 2,876 and 796 primer pairs for whole-genome single amplification bands (100∼2,000 bp) were identified through screening of 3,236 primer pairs and 882 primer pairs with a single band (Table 5, Supplementary Female_Male_INS/DEL100_PrimerSet.xlsx). Variations in the efficacy of PCR high-pass primer design at insertion and deletion sites may be attributed to disparities in structural characteristics and GC content within the regions flanking the insertion/deletion variants.

**Table 5:** Statistics of large-segment differential loci after primer design, e-PCR amplification, and simulated amplification products and size screening

Reference genome	Mutation type	Variant length (>100 bp)	High-throughput design of primers for variant sites	e-PCR of primers with single band	Single band and suitable amplification size (100–2,000 bp)
Female vs. male	Insertion	4,422	4,016	5,735	2,786
	Deletion	4,675	1,314	957	853
Male vs. female	Insertion	4,615	4,131	3,236	2,876
	Deletion	4,385	1,199	882	796

### Validation of molecular markers for genetic sex identification of females and males

**Figure 5:**
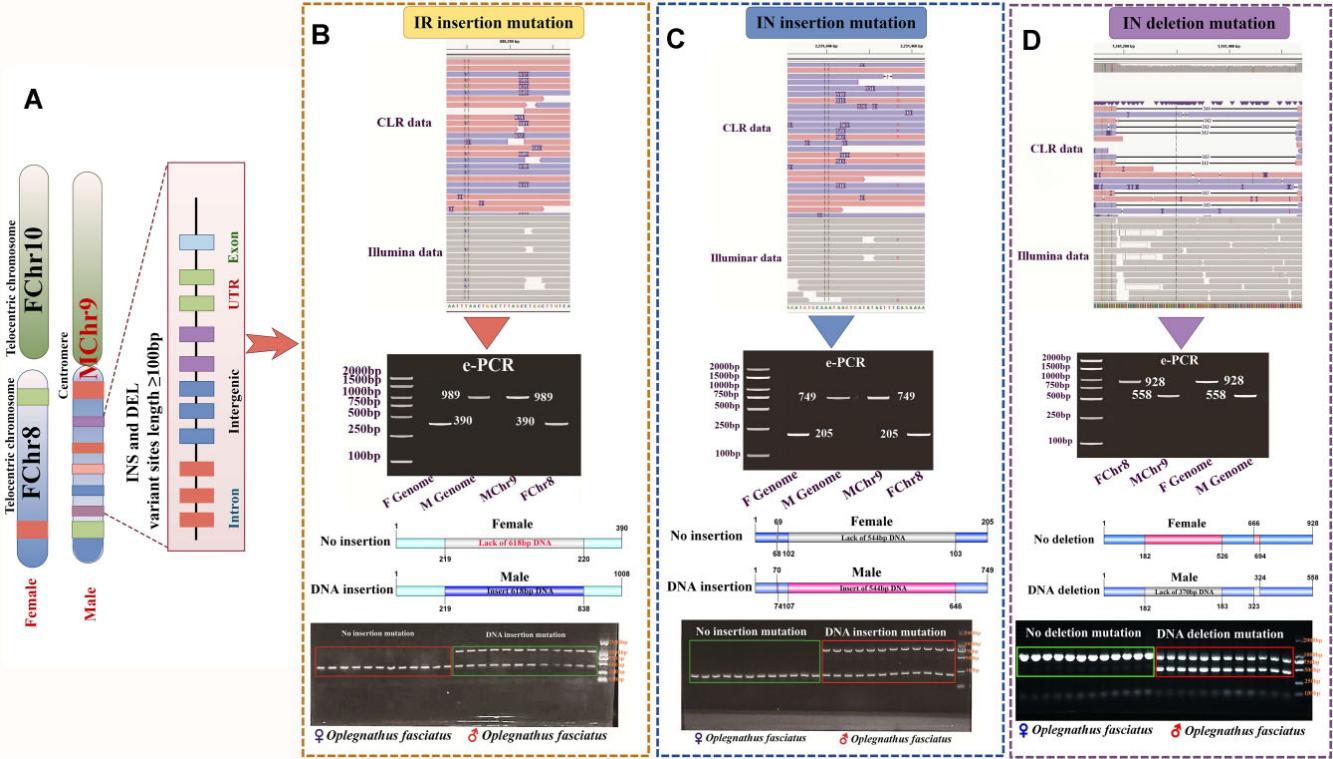
Representative genetic sex marker (insertion/deletion variant sites) located in the intergenic region (IR) and intronic region (IN) of the Japanese parrotfish genome (STOSOd393e). (A) Location of male and female sex markers in the male (MChr9) and female (FChr8) genomes. (B) Male and female sex genetic markers were located at loci in the intergenic region, using the female fish genome as a reference (Supplementary Fig. S2). Illumina and CLR clean data of Bam comparison to the genome for insertion/deletion site validity detection. Pink and blue data from PacBio represent the same and opposite orientation of the female genome and gray represents Illumina clean data. The e-PCR amplified bands were 390 bp and 989 bp in size, corresponding to DNA insertion and deletion patterns and agarose gel electrophoresis for identification of male and female genetically different populations. (C) By utilizing the female genome as a point of comparison, the genetic markers for male and female sexes were identified at specific insertion sites within *nuf2* gene introns (Supplementary Fig. S3, Supplementary Fig. S4). The resulting e-PCR amplified bands, measuring 205 bp and 749 bp in size, were indicative of distinct DNA insertion patterns and utilized in agarose gel electrophoresis for the purpose of distinguishing between male and female genetically differentiated groups. Males exhibited 2 amplified bands, while females exhibited only 1. (D) By utilizing the female fish genome as a point of comparison, the genetic markers for male and female sexes were situated within the deletion sites located in the introns of genes (Supplementary Fig. S5, Supplementary Fig. S6). The amplified bands produced through e-PCR were observed to be 558 bp and 928 bp in length, aligning with the DNA deletion patterns and agarose gel electrophoresis maps utilized for distinguishing genetically distinct groups of males and females. Specifically, 2 bands were amplified in males, while only 1 band was observed in females.

We selected markers located in the intergenic and intragenic regions for female and male genetic sex identification for validation testing. Using the female genome as a reference, a 599-bp DNA fragment was inserted into the male genome at position 880,783 bp on female chromosome 8 (after TTTAGC), corresponding to e-PCR amplicons of 390 bp and 1,008 bp, respectively (Fig. 5, Supplementary Fig. S2). Sequence comparisons of the genetic sex markers showed that the genetic differences between male and female markers were mainly concentrated in the region of 200–800 bp. This variant locus was further confirmed as a valid insertion site using female fish as a reference genome, synthesized using genomic survey data from short-read sequencing data survey of male fish and long-read sequencing data from PacBio platform for read assignment (Fig. 4, Supplementary Fig. S1). The results of PCR amplification and agarose gel electrophoresis were consistent with the results of e-PCR corresponding to 2 bands (390 bp and 1,008 bp) for males and 1 band (390 bp) for females. According to the genetic sex identification marker localization, the marker lies between the *samd3* gene (sterile alpha motif.) (Ofa002334) and *elf3* gene (E74-like factor 3) (Ofa002335) in the female genome (Fig. 5, Supplementary Fig. S2).

The intragenic markers obtained from screening were also used to test the effectiveness of female and male genetic sex identification in Japanese parrotfish. In the region corresponding to the intron of the female *nuf2* gene (component of the NDC80 kinetochore complex) (Ofa002358) on chromosome 8 (2,231,443 bp, after GCAAATA), a 518-bp DNA fragment was inserted into the intron of the male *nuf2* gene, corresponding to e-PCR amplified fragment sizes of 206 bp and 739 bp, respectively (Supplementary Fig. S3). With the female genome as a reference, we utilized the male short-read sequencing data and long-read PacBio sequencing data for read assignment and further confirmed that this candidate variant site was a valid insertion site (Supplementary Fig. S4). Comparison of the sequences for the genetic sex marker showed that the genetic differences between the male and female markers were mainly in the region of 90–650 bp. The results of PCR amplification and agarose gel electrophoresis further showed that the males exhibited 2 bands (206 bp and 739 bp), and the females had 1 band (206 bp), in agreement with the e-PCR results (Supplementary Fig. S4).

In addition to the male insertion genetic sex markers, a male deletion genetic sex marker was also identified. In the region corresponding to female chromosome 8 from 1,161,186 bp (after GATGAGGAAAG) to 1,161,530 bp (before TATGAAGTCT), the males were missing a 343-bp DNA fragment, corresponding to e-PCR amplicon sizes of 558 bp and 928 bp, respectively (Supplementary Fig. S5). A comparison of sequence differences, short-read sequencing data, and long-read PacBio sequencing data read assignments all confirmed that the genetic differences between male and female markers were mainly concentrated in the 182–551 bp and 665–693 bp regions (Supplementary Fig. S6). PCR amplification and agarose gel electrophoresis assays showed 2 bands in males (558 bp and 928 bp) and 1 band in females (928 bp), which was consistent with the results of e-PCR (Supplementary Fig. S6). A similar genetic sex marker for the deletion locus in males was also identified in the region corresponding to chromosome 8 of females at 921,537 bp (after AAATGTGGCGG), with deletion of a 299-bp DNA fragment and insertion of a 119-bp DNA multifragment in males. PCR amplification and agarose gel electrophoresis tests showed 2 bands in males (748 bp and 926 bp) and 1 band in females (926 bp), consistent with the e-PCR results (Supplementary Fig. S7, Supplementary Fig. S8).

## Discussion

Fish physiological sex is influenced by environmental factors, particularly temperature, resulting in discrepancies between the physiological phenotype and genotype of sex [10, 11]. Consequently, it is crucial within the field of fish aquaculture to discover a straightforward approach capable of rapidly determining the genetic sex [60]. DNA molecular markers are created by exploiting the prevalence of polymorphisms in the genomic DNA of eukaryotic organisms, which serves as indicators of individual variations at the DNA level [7]. A review of the literature revealed that over the past decade, approximately 48% of recent studies on male and female sex determination have predominantly employed molecular biology techniques such as restriction fragment length polymorphism (RFLP), RAPD, SSR, and AFLP [60–71]. Meanwhile, with the advancement of second-generation high-throughput sequencing technologies, particularly Illumina, the utilization of sex markers based on SNP/indel variants has increased to approximately 51% [16, 72–77]. With the emergence and rapid advancement of third-generation sequencing technology, exemplified by long-fragment read-length PacBio sequencing, the development of third-generation high-throughput sex markers based on insertions or deletions of large fragments (>100 bp) has become feasible [37, 44, 45]. Our study focuses on Japanese parrotfish with the X_1_X_1_X_2_X_2_/X_1_X_2_Y system [43–45, 47]. We use the assembled genome information of male and female individuals, along with short-read and long-read data, to identify variant sites and evaluate read depth. We also design primers for target regions and perform batch amplification using e-PCR (Figs. 1–3, Tables 2 and 3). Subsequently, a high-throughput identification catalog of sex-specific markers has been established through this methodology, leading to the discovery of 3,639 (2,786 INS/853 DEL, ♀ as reference) and 3,672 (2,876 INS/833 DEL, ♂ as reference) markers, along with 1,021 and 894 high-quality genetic sex identification markers, respectively (Tables 2 and 3). Following agarose gel electrophoresis, 2 amplification bands for males and 1 amplification band for females are successfully identified, enabling rapid differentiation between the sexes in Japanese parrotfish. This advancement in sex marker development, utilizing third-generation high-throughput and precise techniques, will expedite the progress of genetic breeding and facilitate the establishment of genetic sex markers.

Variation in genome structure is intricately linked to human diseases, evolution, gene regulation, and species phenotypes in the biological realm, making it a prominent area of study in genomics research [78]. Previous studies have shown that DNA sequences with insertions and deletions undergo increased selective pressure [79–83]. The presence of deletions and insertions in DNA sequences, regardless of their location in noncoding or coding regions, inevitably influences the functionality of the original sequence to varying extents [78]. Furthermore, evidence also suggests that the length and placement of insertions are not random but rather heavily influenced by the adjacent DNA sequence [79–83]. Prior research has indicated that insertion/deletion variants accounted for 16%, 24%, 13%, 25%, and 15% of genomic genetic polymorphisms in various organisms, including *Drosophila melanogaster, Caenorhabditis elegans, Mus musculus castaneus, Homo sapiens*, and *Arabidopsis thaliana* [34–36, 84–91]. Recent advancements in long-read length sequencing technology have allowed for a more precise examination of human genetic variation, revealing that insertions and deletions collectively contribute to a high portion (13.8%, 11.1%) of genetic polymorphisms in the human genome [87–91]. The focus of genomic variation studies has predominantly been on SNPs and indels of fewer than 50 base pairs due to the limitations of high-throughput sequencing technologies for short read lengths. However, larger genomic structural variants, which encompassed genetic variations not fully captured by SNPs and indels, are closely linked to biological phenotypes and might significantly influence phenotypic traits, disease susceptibility, and adaptive capacity [78]. The utilization of long-read length sequencing technologies such as PacBio and ONT has enabled the generation of extended DNA sequences, thereby enhancing the assembly of diploid genomes of superior quality and the identification of structural variants (SVs) across substantial genomic regions [92]. Previous research has documented 7 deletions (>100 bp) within approximately 700-bp large segments of the *ZmBAM1d* gene, demonstrating a positive association with maize kernel weight. These deletions are consistently inherited in high-yielding maize lines [93]. In mammalian studies, deletion of approximately 40 Mb on chimpanzee chromosome 2, as compared to humans and gorillas within the great ape family, has been identified, leading to stable genetic differentiation among species [94]. Similar results have been documented in *Astyanax mexicanus, Gasterosteus aculeatus, Equus caballus*, and *Canis lupus familiaris* [20, 95–97, 99]. The emergence of long-read length sequencing technology has propelled large-fragment genomic structural variation to the forefront of genetic variation research, particularly in investigations of species’ adaptive evolution [92, 93, 98, 99]. The findings of this study indicate that insertions and deletions identified using the female and male Japanese parrotfish genomes as references constituted 26.96% and 26.97% of the genetic polymorphisms, respectively. Our research has contributed new insights into the prevalence of large-segment insertion/deletion events for Japanese parrotfish, indicating that these events predominantly occurred in the intergenic region in 58.09%–66.33% of instances, with the intronic region following closely at 31.65%–40.35% of cases (Fig. 2, Supplementary Fig. S1, Table 2). We also observe a higher proportion of deletion sites compared to insertion sites. A similar study conducted on the *β-fibrinogen* intron of birds has reported similar findings [100]. Furthermore, this study presents novel findings indicating that over 77% of the identified large deletion sequence tag sites in the genomes of both female and male Japanese parrotfish are attributed to transposable elements (Table 4). It is evident that transposable elements are prevalent in the animal genome and significantly contribute to its evolutionary processes. Previous research has demonstrated that a significant portion (69.5%) of genomic structural variation in large segments of tomato is situated within the transposable element region, which is closely linked to desirable traits in tomato [101]. Comparable studies have also been conducted on gene regulation in human brain diseases and genomic structural variation in evolutionary distinct phenotypes of great apes [99, 102]. Transposable elements have the potential to drive genome and chromosome evolution through various mechanisms, such as insertion-induced gene variation, gene retrotransposition, chromosome recombination, and gene duplication, and serve as sites for ectopic recombination. These findings underscore the importance of transposable elements in facilitating adaptive evolution for Japanese parrotfish. The presence of large fragments of insertion/deletion sequence marker sites is frequently influenced by natural selection pressures, with their formation being intricately linked to adjacent DNA sequences and commonly correlated with characteristic phenotypic traits and aberrant gene regulation [82, 83, 98, 99, 101, 102]. Specifically, the manifestation of albino symptoms in dogs resulting from a 965-kb deletion on chromosome 18 exhibits stable inheritance patterns, while a 4.8-Mb deletion variant on the chromosome of male domestic chickens similarly displays heritability across subsequent generations [96, 103]. Consistent inheritance of variants within populations has also been documented in academic research on maize (*ZmBAM1d*), *Astyanax mexicanus, Ailuropoda melanoleuca* (*Bace2*), great apes (*acan*), and human diseases such as hypertension (*ace*) [93, 95, 99, 104]. The findings from the population genetic sex marker amplification analysis conducted in this study indicated that the insertion/deletion markers exhibited stability in both male and female populations of Japanese parrotfish. Additionally, analysis of gonadal histology and sex-validated marker data from various geographic populations demonstrate the consistent inheritance of large segments of the insertion/deletion markers in individuals of both sexes across different geographic regions (Supplementary Fig. S9). This observation provided additional evidence to support the theory that large-segment insertions and deletions are not random occurrences but rather influenced by natural selection pressures [79, 81–83]. Based on the present findings, the stability of large-segment insertions and deletions in sequence tag sites is evident. However, this should not be taken to imply that all sequence tag sites exhibit this characteristic. A comprehensive evaluation of the evolutionary rate of sequence tags and the expansion of variant marker detection is necessary for ongoing research in this area.

## Conclusions

These results highlight the substantial prevalence of insertion and deletion events in the genetic makeup derived from the X_1_X_1_X_2_X_2_/X_1_X_2_Y sex-determination system of Japanese parrotfish. In our study, we have successfully exploited a novel approach for the development of genetic sex markers in Japanese parrotfish. This approach involved the utilization of a primer batch electronic design technique, which utilized the insertion and deletion loci as the target regions. Additionally, we implement an electronic batch amplification technique, allowing for the efficient and rapid high-throughput identification and screening of a substantial number of variant sites. This methodology represents a significant advancement in the field of Japanese parrotfish genetic sex marker development. The study yields a greater number of genetic sex markers with higher amplification efficiency compared to those generated by previous technologies spanning the first or second generation. This methodology is applicable not only to the efficient generation of genetic sex markers for the X_1_X_1_X_2_X_2_/X_1_X_2_Y sex-determination system but also to the determination of genetic sex in various sex-determination types (including autosomal and sex chromosome differences). It was particularly well suited for sex-determination systems featuring heteromorphic chromosomes, such as the XY-type, ZW-type, XX/XY_1_Y_2_-type, ZZ/ZW_1_W_2_-type, and other species with different sex-determination mechanisms.

## Supplementary Material

giae045_GIGA-D-23-00373_Original_Submission

giae045_GIGA-D-23-00373_Revision_1

giae045_GIGA-D-23-00373_Revision_2

giae045_Response_to_Reviewer_Comments_Original_Submission

giae045_Response_to_Reviewer_Comments_Revision_1

giae045_Reviewer_1_Report_Original_Submission Syed Farhan Ahmad -- 1/23/2024 Reviewed

giae045_Reviewer_1_Report_Revision_1 Syed Farhan Ahmad -- 5/1/2024 Reviewed

giae045_Reviewer_2_Report_Original_Submission Frederic Brunet -- 1/29/2024 Reviewed

giae045_Reviewer_2_Report_Revision_1 Frederic Brunet -- 4/23/2024 Reviewed

giae045_Reviewer_3_Report_Original_Submission Christiaan Henkel -- 1/30/2024 Reviewed

giae045_Reviewer_3_Report_Revision_1 Christiaan Henkel -- 5/14/2024 Reviewed

giae045_Supplemental_Files

## Data Availability

Sequencing data are available from NCBI BioProjects PRJNA486885 and PRJNA563003. The code used for statistics on insertions and deletions as well as annotation scripts and base variant region visualization scripts can be access from Figshare [105]. All additional supporting data are available in the *GigaScience* repository, GigaDB [106].

## Additional Files

**Supplementary Fig. S1**. Frequency of insertion and deletions (>100 bp) in functional regions of the *O. fasciatus* genome. (a) Number and frequency of insertion events using the female *O. fasciatus* genome as reference. (b) Distribution statistics of deletion variant length using the female *O. fasciatus* genome as a reference. (c) Number and frequency of insertion variant loci utilizing the male *O. fasciatus* genome as a point of reference. (d) Distribution statistics of deletion variant loci using the male *O. fasciatus* genome as a reference. Non-trans-variation represented the intron and intergenic regions. Trans-variation represented the UTR, CDS regions. The frequency of insertions (a, c) was quantified based on the number of events, while deletions (b, d) were characterized by their length in nucleotides.

**Supplementary Fig. S2**. Representative type of genetic sex marker (insertion/deletion variant sites) located in the intergenic region of the *O. fasciatus* genome. (a) Information on the location of insertion/deletion sequence differences in male and female genetic sex marker and their differences in comparison with male and female genomic sequences. (b) Location of male and female sex markers in the male and female genomes. (c) Illumina and CLR clean data of Bam comparison to genome for insertion/deletion site validity detection. (d) Nucleotide sequence comparison of marker for female and male genetic sex identification. (e) Visualization of regions of nucleotide sequence heterogeneity in markers for female and male genetic sex identification. (f) Electronically simulated amplification results of target marker primer (e-PCR). (g) Results of PCR amplification and agarose gel electrophoresis detection of target marker (2 bands for males and 1 band for females).

**Supplementary Fig. S3**. Representative genetic sex marker (insertion/deletion variant sites) located in the intron region of the *nuf2* gene in *O. fasciatus*. (a) Collinearity relationships between male and female genetic sex markers and their association with the male and female genomes. (b) Location of male and female sex markers in the male and female genomes. (c) Unit composition of gene functional regions, occurrence of insertions and deletions of genetic sex markers, and sequence length patterns.

**Supplementary Fig. S4**. Validity testing of genetic markers located in the intronic region of the *nuf2* gene. (a) Illumina and CLR clean data of Bam comparison to the genome for insertion/deletion site validity detection. (b) Nucleotide sequence comparison of markers for female and male genetic sex identification. (c) Comparison of genetic sex marker sequences based on the global alignment standard. (d) Visualization regions of nucleotide sequence heterogeneity in markers for female and male genetic sex identification. (e) Electronically simulated amplification results of the target marker primer (e-PCR). (f) Results of PCR amplification and agarose gel electrophoresis detection of target markers (2 bands for males and 1 band for females).

**Supplementary Fig. S5**. Representative male deletion genetic sex marker (insertion/deletion variant sites). (a) Collinearity relationships between male and female genetic sex markers and their association with the male and female genomes. (b) Location of male and female sex markers in the male and female genomes. (c) Unit composition of gene functional regions and occurrence of male deletion genetic sex markers.

**Supplementary Fig. S6**. Validity testing of the male deletion genetic markers. (a) Illumina and CLR clean data of Bam comparison to the genome for insertion/deletion site validity detection. (b) Nucleotide sequence comparison of markers for female and male genetic sex identification. (c) Comparison of genetic sex marker sequences based on the global alignment standard. (d) Visualization regions of nucleotide sequence heterogeneity in markers for female and male genetic sex identification. (e) Electronically simulated amplification results of the target marker primer (e-PCR). (f) Results of PCR amplification and agarose gel electrophoresis detection of target markers (2 bands for males and 1 band for females).

**Supplementary Fig. S7**. Representative type of genetic sex marker (insertion/deletion variant sites) with the male deletion of the *O. fasciatus* genome. (a) Colinearity relationships between male and female genetic sex markers and their association with the male and female genomes. (b) Location of male and female sex markers in the male and female genomes. (c) Illumina and CLR clean data of Bam comparison to genome for insertion/deletion site validity detection. (d) Nucleotide sequence comparison of marker for female and male genetic sex identification. (e) Visualization of regions of nucleotide sequence heterogeneity in markers for female and male genetic sex identification.

**Supplementary Fig. S8**. Electronically simulated amplification results of target marker primer (e-PCR) (left). Results of PCR amplification and agarose gel electrophoresis detection of target marker (2 bands for males and 1 band for females) (right).

**Supplementary Fig. S9**. Male and female sex genetic markers were located in the intergenic region (IR), insertion sites in introns (IN), and their validation in different geographic populations, using the male genome as a reference. (a) Wild population samples were collected from offshore populations in Shandong and Zhejiang. (b) Wild samples were examined histologically for male gonads. Testis histology with 200 µm and 40 µm for I and II locations. Psp, primary spermatocyte; Ssp, secondary spermatocyte; St, spermatid; Sz, spermatozoa. (c) Wild samples were examined histologically for female gonads. Ovary histology with 250 µm and 25 µm. I, primary growth chromatin nucleolar stage; II–III, primary growth perinucleolus stage; II, early perinucleolus stage; III–IV, late perinucleolus stage. (d) Location of male and female sex markers in the male (MChr9) and female (FChr8) genomes. (e) Using the male genome as a reference, male and female sex genetic markers were located at deletion sites in the intergenic region. The e-PCR amplified bands were 374 bp and 1,045 bp in size, corresponding to DNA deletion pattern maps and agarose gel electrophoresis maps for population identification of male and female genetic differences. Two bands were amplified in males and 1 band in females. (f) Male and female sex genetic markers were located at deletion sites in the intronic region. The e-PCR amplified bands were 528 bp and 1,251 bp in size, corresponding to DNA deletion pattern maps and agarose gel electrophoresis maps for population identification of male and female genetic differences. Two bands were amplified in males and 1 band in females. (g) Male and female sex genetic markers were located at deletion sites in the intronic region. The e-PCR amplified bands were 125 bp and 472 bp in size, corresponding to DNA deletion pattern maps and agarose gel electrophoresis maps for population identification of male and female genetic differences. Two bands were amplified in males and 1 band in females.

## Abbreviations

AFLP: amplified fragment length polymorphism; BUSCO: Benchmarking Universal Single-Copy Orthologs; CCS: circular consensus sequence; CLR: continuous long read; e-PCR: electronic PCR; NGS: next-generation sequencing; RAPD: random amplification of polymorphic DNA; RFLP: restriction fragment length polymorphism; SMRT: single-molecule real-time sequencing technology; SSR: simple sequence repeat; SV: structural variant; T2T: telomere-to-telomere.
