# Supplementary material for: Innovative approach for high-throughput exploiting sex-specific markers in Japanese parrotfish Oplegnathus fasciatus
Source: Gigascience. 2024 Jul 19;13:giae045. doi: 10.1093/gigascience/giae045 (PMC11258905; doi:10.1093/gigascience/giae045)
Supplement: giae045_GIGA-D-23-00373_Original_Submission [file giae045_giga-d-23-00373_original_submission.pdf]

GigaScience

A novel and scalable method for high-throughput and accurate exploitation of sex-specific markers: a case study on Oplegnathus fasciatus

--Manuscript Draft--

**Manuscript Number:**	GIGA-D-23-00373
**Full Title:**	A novel and scalable method for high-throughput and accurate exploitation of sex-specific markers: a case study on Oplegnathus fasciatus
**Article Type:**	Research
**Funding Information:**	National Outstanding Youth Science Fund Project of National Natural Science Foundation of China (No. 42276107; No. 32270472) — Prof Yongshuang Xiao
**Abstract:**	Background<br><br>The utilization of sex-specific molecular markers has emerged as a significant technical approach to augment fish production and enhance economic value, while also establishing a fundamental basis for unraveling the intricate molecular mechanisms governing fish sex determination. In the past ten years, the field of genetic sex marker mining has primarily relied on first-generation development methods such as RFLP, RAPD, SSR, and AFLP, as well as second-generation techniques utilizing Illumina's SNP/InDel markers. However, the progress of sex-controlled breeding has been hindered by several factors, including the limited efficiency of the aforementioned methods, complex experimental procedures, high development costs, a high incidence of false positives, unstable markers, and inconvenient on-site testing. Nevertheless, the emergence and rapid advancement of third-generation sequencing technology offers new opportunities for overcoming these limitations.<br><br>Findings<br><br>Using male-female linear genomic information combined with Illumina survey and PacBio CLR / CCS data, a database of large-segment (>100 bp) insertion–deletion genetic variants was constructed using a genome-wide variant site scanning method with bidirectional comparisons. A database of bulk primers and simulated PCR for the male–female variant loci were then constructed, employing primer design for the target region and e-PCR technology. Finally, the criteria for rapid identification of male and female differences were established based on agarose gel electrophoresis with two amplified bands for males and one amplified band for females. A high-throughput identification database of sex-specific markers for Oplegnathus fasciatus was constructed utilizing this method, yielding 3,645 (2,791 INS/854 DEL, ♀ as reference) and 3,872 (3,039 INS/833 DEL, ♂ as reference) genetic sex identification markers. Four differential loci were randomly selected from the database for validation, and the results met the criteria for male–female differences. Utilization of this technology will accelerate the identification of genetic sex markers for species, contributing to the rapid development of genetic breeding.<br><br>Conclusions<br><br>By utilizing the linear genomic information of male and female individuals acquired through PacBio sequencing, along with data from Illumina surveys and PacBio CLR/CCS. Our research extensively employed whole-genome variant site scanning and identification, high-throughput primer design for the target regions, and e-PCR batch amplification. By employing this comprehensive approach, which encompassed genome-wide variant site scanning, high-throughput primer design within the desired region, and e-PCR batch amplification and validation methodologies, we successfully developed a database encompassing insertion/deletion loci in large segments (>100 bp) for both male and female O. fasciatus.
**Corresponding Author:**	Yongshuang Xiao, phD<br>Institute of Oceanology Chinese Academy of Sciences<br>Qingdao, CHINA

Powered by Editorial Manager® and ProduXion Manager® from Aries Systems Corporation

**Corresponding Author Secondary Information:**	
**Corresponding Author's Institution:**	Institute of Oceanology Chinese Academy of Sciences
**Corresponding Author's Secondary Institution:**	
**First Author:**	shuang Yong Xiao
**First Author Secondary Information:**	
**Order of Authors:**	shuang Yong Xiao
	Yongshuang Xiao, phD
	Zhizhong Xiao
	Daoyuan Ma
	Jing Liu
	Jun Li
**Order of Authors Secondary Information:**	
**Additional Information:**	
**Question**	**Response**
Are you submitting this manuscript to a special series or article collection?	No
**Experimental design and statistics**<br><br>Full details of the experimental design and statistical methods used should be given in the Methods section, as detailed in our Minimum Standards Reporting Checklist. Information essential to interpreting the data presented should be made available in the figure legends.<br><br>Have you included all the information requested in your manuscript?	Yes
**Resources**<br><br>A description of all resources used, including antibodies, cell lines, animals and software tools, with enough information to allow them to be uniquely identified, should be included in the Methods section. Authors are strongly encouraged to cite Research Resource Identifiers (RRIDs) for antibodies, model organisms and tools, where possible.	Yes

Powered by Editorial Manager® and ProduXion Manager® from Aries Systems Corporation

Have you included the information requested as detailed in our Minimum Standards Reporting Checklist?	
**Availability of data and materials**<br><br>All datasets and code on which the conclusions of the paper rely must be either included in your submission or deposited in publicly available repositories (where available and ethically appropriate), referencing such data using a unique identifier in the references and in the “Availability of Data and Materials” section of your manuscript.<br><br>Have you have met the above requirement as detailed in our Minimum Standards Reporting Checklist?	Yes

A novel and scalable method for high-throughput and accurate exploitation of sex-specific markers: a case study on *Oplegnathus fasciatus*

Yongshuang Xiao[1,2,3,4*†], Zhizhong Xiao[1,2,3,4†], Daoyuan Ma[1,2,3,4], Jing Liu[1], Jun Li[1,2,3,4*]

[1]Center for Ocean Mega-Science, Institute of Oceanology, Chinese Academy of Sciences, Qingdao, China， [2]CAS and Shandong Province Key Laboratory of Experimental Marine Biology, Institute of Oceanology, Chinese Academy of Sciences, Qingdao, China， [3]Key Laboratory of Breeding Biotechnology and Sustainable Aquaculture, Chinese Academy of Sciences, Qingdao, China， [4]Laboratory for Marine Biology and Biotechnology, Qingdao National Laboratory for Marine Science and Technology, Qingdao, China.

† Co-first authors

*Correspondence address: Yongshuang Xiao, Mega-Science, Chinese Academy of Sciences, 7 Nanhai Road, Qingdao, 266071, China; Tel: +86-053282896729; E-mail: dahaishuang1982@163.com; Jun Li, Institute of Oceanology, Chinese Academy of Sciences, 7 Nanhai Road, Qingdao, 266071, China; Tel: +86-053282898718; E-mail: junli@qdio.ac.cn.

†Contributed equally to this work.

Yongshuang Xiao, ORCID: 0000-0002-1979-4555

Zhizhong Xiao, ORCID: 0000-0003-2403-1381

Daoyuan Ma, ORCID: 0000-0002-9271-4371

Abstract

Background

The utilization of sex-specific molecular markers has emerged as a significant technical approach to augment fish production and enhance economic value, while also establishing a fundamental basis for unraveling the intricate molecular mechanisms governing fish sex determination. In the past ten years, the field of genetic sex marker mining has primarily relied on first-generation development methods such as RFLP, RAPD, SSR, and AFLP, as well as second-generation techniques utilizing Illumina's SNP/InDel markers. However, the progress of sex-controlled breeding has been hindered by several factors, including the limited efficiency of the aforementioned methods, complex experimental procedures, high development costs, a high incidence of false positives, unstable markers, and inconvenient on-site testing. Nevertheless, the emergence and rapid advancement of third-generation sequencing technology offers new opportunities for overcoming these limitations.

Findings

Using male-female linear genomic information combined with Illumina survey and PacBio CLR / CCS data, a database of large-segment (>100 bp) insertion–deletion genetic variants was constructed using a genome-wide variant site scanning method with bidirectional comparisons. A database of bulk primers and simulated PCR for the male–female variant loci were then constructed, employing primer design for the target region and e-PCR technology. Finally, the criteria for rapid identification of male and female differences were established based on agarose gel electrophoresis with two amplified bands for males and one amplified band for females. A high-throughput identification database of sex-specific markers for *Oplegnathus fasciatus* was constructed utilizing this method, yielding 3,645 (2,791 INS/854 DEL, ♀ as reference) and 3,872 (3,039 INS/833 DEL, ♂ as reference) genetic sex identification markers. Four differential loci were randomly selected from the database for validation, and the results met the criteria for male–female differences. Utilization of this technology will accelerate the identification of genetic sex markers for species, contributing to the rapid development of genetic breeding.

Conclusions

By utilizing the linear genomic information of male and female individuals acquired through PacBio sequencing, along with data from Illumina surveys and PacBio

CLR/CCS. Our research extensively employed whole-genome variant site scanning and identification, high-throughput primer design for the target regions, and e-PCR batch amplification. By employing this comprehensive approach, which encompassed genome-wide variant site scanning, high-throughput primer design within the desired region, and e-PCR batch amplification and validation methodologies, we successfully developed a database encompassing insertion/deletion loci in large segments (>100 bp) for both male and female *O. fasciatus*.

***Keywords*:** large-segment insertion/deletion; bulk primers design; e-PCR technology; *Oplegnathus fasciatus*; high-throughput identification

Data description

Introduction

Sexual dimorphism is common in the animal kingdom, but the mechanisms of sex determination are diverse[1, 2]. Thus, sex research has always been a topic of interest in biology. Fish occupy a key position in the vertebrate evolutionary system, and because of their wide distribution, large number of species and diverse sex determination mechanisms, they are an important target for the study of sex determination mechanisms[3-9]. However, most fish have a low degree of differentiation between the sex chromosomes, which is difficult to detect from the morphology of the chromosomes[10]. The development of sex-specific molecular markers and sex-control biotechnology has provided an important technical way to increase fish production and economic value and has also laid the foundation for deciphering the molecular mechanism of fish sex determination[11]. Researchers have succeeded in developing sex-specific or correlated molecular markers in many fish species using a variety of techniques, including SSR, RAPD, AFLP, SNP and In Del[5, 6, 12-17]. However, these methods for obtaining sex-specific markers have many problems, such as low throughput, single markers, cumbersome development and experimental procedures, high development costs, low amplification efficiency and inconvenient on-site detection. The first issue to be addressed was to establish a high-throughput and easy-to-detect marker procedure for sex-specific identification in the investigation of sex dimorphism, mechanisms of sex differentiation, and development and use of sex-producing traits.

In recent years, with the rapid development of genome sequencing technology and the significant decrease in the cost of sequencing, it has become possible to perform

whole-genome sequencing of important cultured species. Rapid developments in next-generation sequencing (NGS) technologies have created significant opportunities for the discovery and use of species germplasm resources[4]. In 2002, the genome sequencing of *Takifugu rubripes* was completed, making it the first model fish to have its entire genome sequenced[18]. Since then, whole-genome sequencing of more than 40 fish species, such as *Oryzias latipes*, *Gasterosteus aculeatus* and *Salmo salar*, has been completed both nationally and internationally[19-21]. Most of these fish genomes have been sequenced using second-generation sequencing (Illumina), and although second-generation sequencing technology has high throughput, the sequencing read lengths are relatively short[4, 18-21]. Therefore, it is difficult to obtain high-quality genome maps and accurate high-throughput information on chromosome structural variation by relying on second-generation sequencing technology alone. This is particularly true for genomes that are highly complex and have highly repetitive regions.

At present, third-generation sequencing technology with high precision of long fragments represented by single-molecule real-time sequencing has matured, which includes Helicos' single-molecule sequencing technology (SMSTM), Oxford Nanopore's single-molecule nanopore sequencing technology, Pacific Biosciences' (PacBio) single-molecule real-time sequencing technology (SMRT), and so on. Among them, PacBio RS sequencing technology is the most widely used and commercialized core third-generation sequencing technology and has been widely used in the genome assembly of several species, such as an Asian man, *Oropetium thomaeum* and *Lates calcarifer*[22-24]. For the Chinese human genome sequenced by PacBio technology, the assembly level reached a contig N50 of 8.3 Mb, which met the standard of the completed map[22]. In addition, HiC technology based on next-generation sequencing technology has gradually become a favourable tool for chromosome assembly[25]. With the rapid development of sequencing technology and the improvement of assembly technology, the genome integrity and accuracy have been greatly improved, especially in marine fishes, and the assembly now obtained Contig N50 up to the level of 28 Mb for *Sciaenops ocellatus*[26]. Thus, the development of next-generation sequencing technologies has made it possible to exploit high-throughput sex-specific markers in high-precision linear genomic data.

Oplegnathus fasciatus (Temminck & Schlegel, 1844, Fishbase ID: 1709) is a rocky reef fish distributed in a wide range of shallow waters around Korea, Japan, China and Hawaii[27-30]. *O. fasciatus* has become an important fishery resource for offshore cage

culture and fish stocking in marine ranches in China, Japan and Korea[29, 30]. *O. fasciatus* has been a commercially important species for sashimi production and recreational angling, with engineered aquaculture in China yielding ex-factory prices of up to US$30/kg[30]. Meanwhile, *O. fasciatus* is characterized by a multiple $X_1X_1X_2X_2/X_1X_2Y$ sex chromosome system[31-34]. Sexual dimorphism has been observed in *O. fasciatus*, with males growing faster than females, especially at weights greater than 500 grams[7, 8, 33, 34]. Therefore, the breeding of new strains with a male predominance has become one of the goals for future breeding of *O. fasciatus*. The development of appropriate genetic sex markers is a prerequisite for achieving a new variety of *O. fasciatus* with male predominance. Currently, available markers for the genetic characterization of *O. fasciatus* are extremely limited and have mainly been identified by AFLP, SSR and Illumina technologies, which constrained the process of exploiting new varieties of *O. fasciatus*[35-37]. The completion of the female and male genomes of *O. fasciatus* has enabled high-throughput development of large segments to visualize genetic sex markers from a genome-wide perspective.

In this work, a database of genomic differences between females and males was constructed based on the linear genomic information of *O. fasciatus* by using bidirectional whole-genome sequence alignment[38, 39]. Using the difference database, we obtained datasets of insertion and deletion variant sites larger than 100 bp by screening and used Illumina data from genome sequencing and CLR/CCS data to test the confidence of large segmental variant sites[40]. On the basis of the *O. fasciatus* large-banded variant loci, we used PCR e-simulation amplification technology to perform a genome-wide PCR e-simulation high-throughput amplification assay on the high-confidence large-banded variant loci dataset and to screen for single amplified-banded variant loci in both the female and male genomes[40]. Then, we performed PCR amplification and agarose gel electrophoresis for the mutant loci detected by PCR electron simulation. Since *O. fasciatus* exhibits an $X_1X_1X_2X_2/X_1X_2Y$ sex chromosome system, a single primer amplifies two bands in males and one primer in females.

The paper was organized as follows: First, a fast scanning method for female and male linear genomic variant loci was introduced, and an insertion/deletion dataset of large segments was constructed. Using the constructed insertion/deletion dataset of large fragments, high-throughput candidate molecular marker loci for female and male genetic sex identification of *O. fasciatus* were identified. Then, the new technique of PCR electronic simulation with bulk amplification was used to perform simulated

amplification of high-throughput candidate molecular marker loci to screen primers for the single amplification of the female and male genomes with amplification differences. Finally, a high-throughput precision marker dataset for male and female genetic sex identification was obtained and tested by agarose gel electrophoresis (Figure 1).

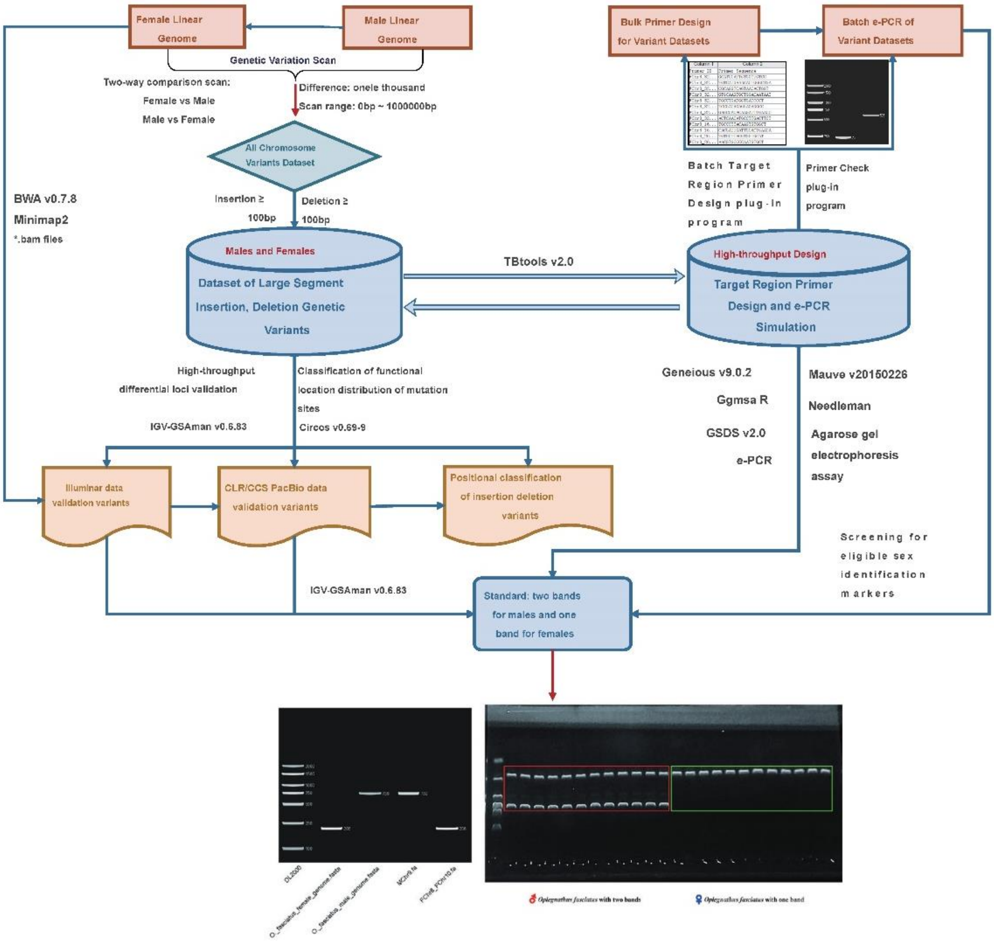

Figure 1: High-throughput exploitation process of genetic sex markers for *O. fasciatus*

Materials and Methods

We employed the genome variant site scanning plugin with accompanying parameters (diff: oneInThousand, VarRange: 0~1000000 bp, BatchSize: 500 bp, Min Align Length for Cov Calc 10000 bp, Min Align Length for Var Calling: 50000 bp) in TBtools (v 2.0) software to conduct bidirectional comparisons of the complete genomic sequences of male and female organisms[40, 41]. Additionally, we established databases containing genomic disparities between males and females. To classify base substitution, insertion, and deletion sites, as well as to screen large insertion-deletion sites (>100 bp) against a differential database, we utilized the Table Row Extract program. Furthermore, we utilized BWA (v 0.7.8) and Minimap2 software to align the survey data obtained

through Illumina sequencing and the CLR/CCS data reads obtained through PacBio sequencing to the male and female genomes using default parameters to conduct a large-scale comparison as a means of validation[42, 43]. Subsequently, employing the Batch Target Region Primer Design plug-in and e-PCR plug-in in TBtools (v 2.0) software, we designed primers for high-throughput amplification and screened them for single amplification bands at the insertion and deletion sites specific to the male and female genomes[40].

The positional distribution of female and male genetic variant loci on the genome was depicted using Circos (v 0.69-9) software[44]. Homology comparison and visualization of insertion-deletion sites with male and female linear genomes were conducted using a combination of Geneious (v 9.0.2) software and Mauve program (v 20150226)[45, 46]. The comparison of genetic sex marker sequences with the Global Alignment standard was performed using the Needleman Wunsch plug-in in TBtools (v 2.0)[40]. Additionally, the visualization of genetic sex marker variant site regions was accomplished using Ggmsa program[47]. GSDS (v 2.0) software was utilized to illustrate the location of the variant site within the functional region of the gene[48]. We performed functional enrichment analysis using Metascape (https://metascape.org/) to identify enriched Gene Ontology (GO) and Kyoto Encyclopedia of Genes and Genomes (KEGG) pathways associated with genes harbouring significant insertion/deletion variants within extensive segments of the exon region[49]. The analysis was conducted with specific parameters, including a minimum overlap of 1, a *p value* cut-off of 0.05, and a minimum enrichment threshold of 1.5. A total of 24 tails from both male and female *O. fasciatus* specimens were collected and distinguished through gonadal histology. The reliability of the genetic sex markers was assessed by randomly identifying male and female populations using e-PCR in conjunction with agarose gel electrophoresis.

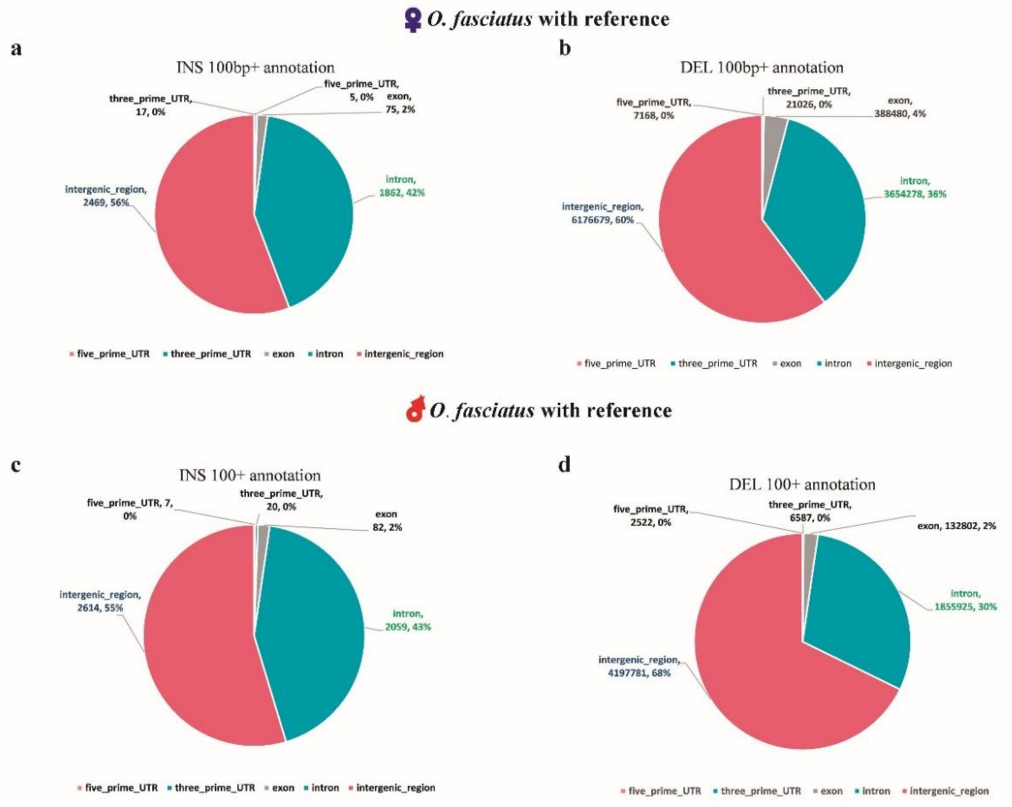

Figure 2: Distribution statistics of insertion and deletion fragment positions in genomic functional regions of *O. fasciatus*.

(a) Distribution statistics of insertion variant loci using the female fish genome as a reference. (b) Distribution statistics of deletion variant length using the female fish genome as a reference. (c) Distribution statistics of insertion variant loci using the male fish genome as a reference. (d) Distribution statistics of deletion variant loci using the male fish genome as a reference.

Results

Construction of insertion/deletion variant loci datasets for large segments

Using the female genome as a reference, we obtained 1,919,620 male and female differential variant sites, including 191,915 insertion sites, 325,620 deletion sites and 1,402,085 nucleotide substitutions (Figure 2). To screen suitable field-actionable markers for large-band genetic sex identification, we screened this insertion/deletion variant site database for variants larger than 100 bp and obtained 4,428 insertion sites with a differential sequence size of 6,003,658 bp, of which 0.11% were located in 5′ UTRs, 0.38% in 3′ UTRs, 42.05% in intronic regions, 1.69% in exonic regions, and 55.76% in intergenic regions (Figure 2, Supplementary Female_reference_INS/DEL100_Set.xlsx). In addition, we obtained 4,691 deletion sites with a differential sequence size of 10,247,631 bp (Supplementary Female_reference_INS/DEL100_Set.xlsx). As the deletion variant sites spanned multiple genomic functional regions, we performed statistical analysis of the actual

locations where the deletion bases were located, with 0.07% located in 5′ UTRs, 0.21% in 3′ UTRs, 35.66% in intronic regions, 3.79% in exonic regions, and 60.27% in intergenic regions (Figure 2, Table 1). The highest number of chromosomal mutations occurred in female fish chromosomes 8 and 10, with 335 and 344 insertion and deletion sites, respectively (Figure 3).

Using the male *O. fasciatus* genome as a reference, 1,926,295 female–male differential variant sites were obtained, including 326,823 insertion sites, 192,672 deletion sites and 1,406,800 nucleotide substitution sites (Figure 2). Through large fragment screening (>100 bp), we obtained 4,782 insertion sites with a differential sequence size of 10,447,125 bp, of which 0.15% were located in 5′ UTRs, 0.42% in 3′ UTRs, 43.06% in intronic regions, 1.72% in exonic regions, and 54.66% in intergenic regions (Figure 2, Table 1, Supplementary Male_reference_INS100_Set.xlsx). The chromosome with the highest number of mutations was chromosome 9 in male *O. fasciatus*, in which the number of insertion and deletion sites were 354 and 345, respectively (Figure 3); male chromosome 9 is homologous to chromosomes 8 and 10 of female *O. fasciatus*. Male chromosome 6 had the second highest number of mutations, with 289 insertion sites and 256 deletion sites (Figure 3).

Table 1. Statistics of the positional distribution of insertion/deletion variants in the male and female genomes of *O. fasciatus*.

Type of gene function region	Female genome as a reference		Male genome as a reference	
	Insertion variant loci no. (>100 bp)	Deletion variant length (>100 bp)	Insertion variant loci no. (>100 bp)	Deletion variant length (>100 bp)
5′ UTR	5	7,168	7	2,522
3′ UTR	17	21,026	20	6,587
Exon	75	388,480	82	132,802
Intron	1,862	3,654,278	2,059	1,855,925
Intergenic region	2,469	6,176,679	2,614	4,197,781
Total	4,428	10,247,631	4,782	6,195,617

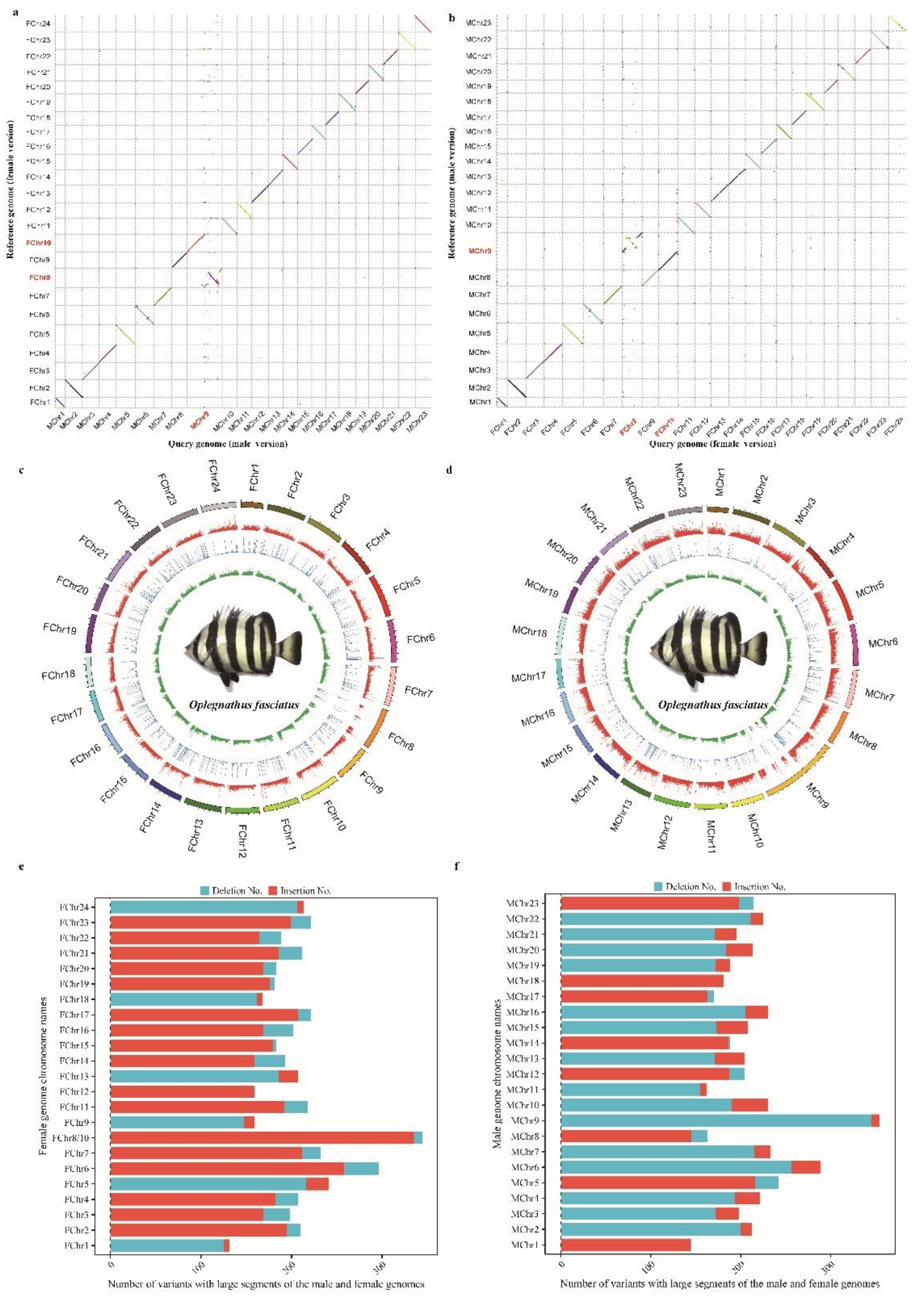

Figure 3: The covariance of genomic sequence differences between the male and female genomes of *O. fasciatus* and distribution statistics of variant sites.

(a) The female genome (reference) of *O. fasciatus* vs. the male genome. (b) The male genome (reference) of *O. fasciatus* vs. the female genome. (c) Distribution statistics of variant loci using the female fish genome as a reference. (d) Distribution statistics of variant loci using the male fish genome as a reference. The outer to inner circles show chromosome information, insertion site distribution, deletion site distribution and base substitution distribution, respectively. (e) - (f) Dual-valued histograms showing the number of insertion/deletion sites per chromosome with reference to the male and female genomes, respectively, where chromosome 9 in males was homologous to chromosomes 8 and 10 in females.

Table 2. Statistics of large-segment differential loci after primer design, e-PCR amplification, and simulated amplification products and size screening.

Reference genome	Mutation type	Variant length (>100 bp)	High-throughput design of primers and e-PCR	Single band and suitable amplification size (100~2000 bp)
Female vs. Male	Insertion	4,428	5,044	2,791
	Deletion	4,691	1,628	854
Male vs. Female	Insertion	4,782	5,505	3,039
	Deletion	4,520	1,545	833

High-throughput primer design and e-PCR bulk simulation for target regions

We used the identified large insertion/deletion variants as target regions for male and female sex identification and designed high-throughput primers to construct high-throughput primer datasets for the target markers. A total of 5,044 and 1,628 primer pairs were obtained by high-throughput primer design for the insertion/deletion target regions, respectively, using females as the reference (Table 2). To further test the accuracy and uniqueness of the high-throughput primers across the genome, we performed genome-wide electronic simulated amplification of the obtained primer pairs by using e-PCR. A total of 2,791 and 854 genome-wide single amplification band primer pairs were obtained by screening from 5,044 primer pairs and 1,628 primer pairs, respectively, which yielded amplification products of 100 to 2,000 bp (Table 2, Supplementary Female_Male_INS/DEL100_PrimerSet.xlsx). Using the *O. fasciatus* male genome as a reference, we obtained 5,505 and 1,545 primer pairs corresponding to insertions/deletions of the target variant sites, respectively (Table 2, Supplementary Female_Male_INS/DEL100_PrimerSet.xlsx). Among them, 3,039 and 833 primer pairs for whole-genome single amplification bands were obtained (Table 2, Supplementary Female_Male_INS/DEL100_PrimerSet.xlsx).

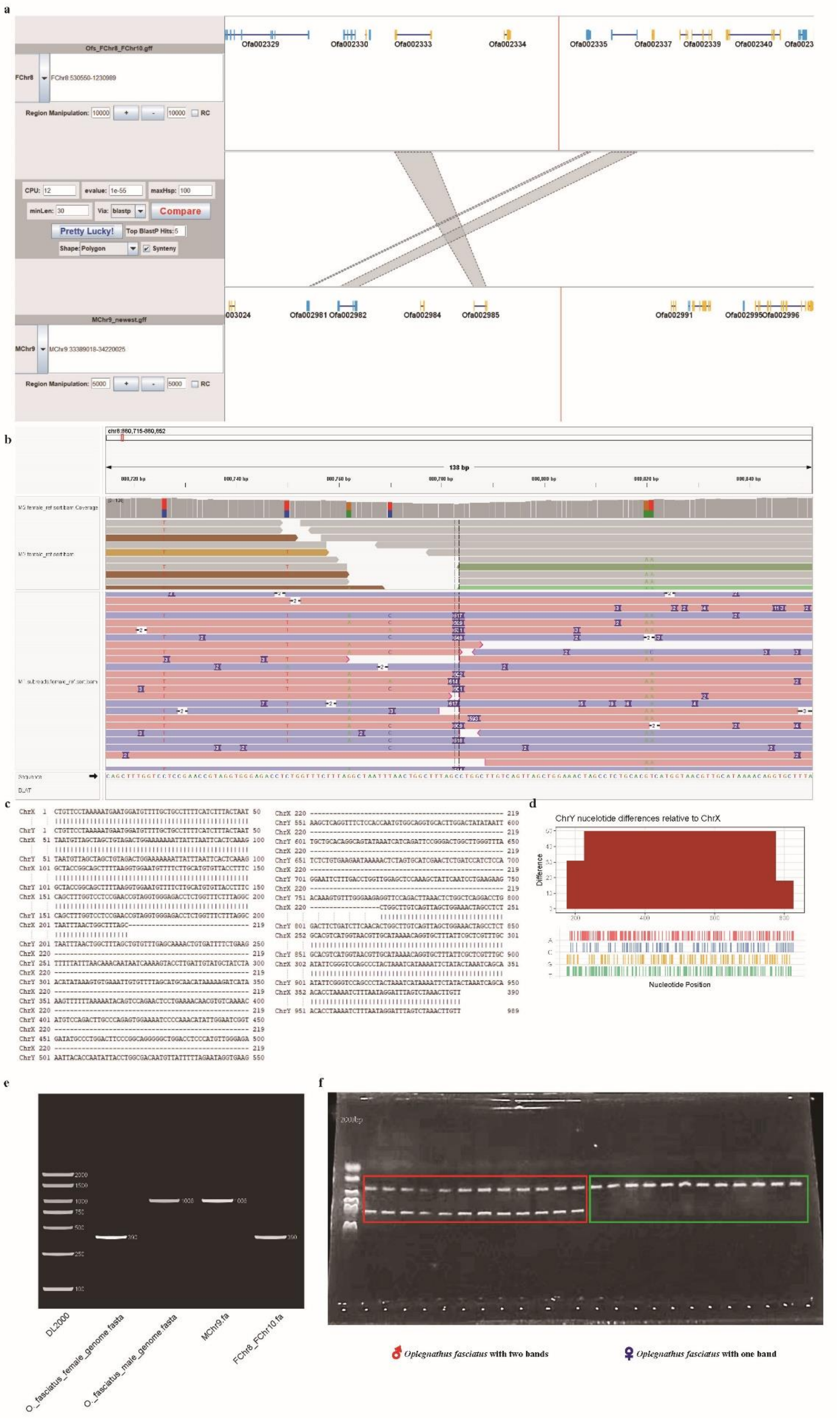

Figure 4: Representative genetic sex marker (insertion deletion variant sites) located in the intergenic region of the *O. fasciatus* genome.

(a) Location of male and female sex markers in the male and female genomes. (b) Illumina and CLR clean data of Bam comparison to the genome for insertion/deletion site validity detection. (c) Nucleotide sequence comparison of

markers for female and male genetic sex identification. (d) Visualization of regions of nucleotide sequence heterogeneity in markers for female and male genetic sex identification. (e) Electronically simulated amplification results of the target marker primer (e-PCR). (f) Results of PCR amplification and agarose gel electrophoresis detection of target markers (two bands for males and one band for females).

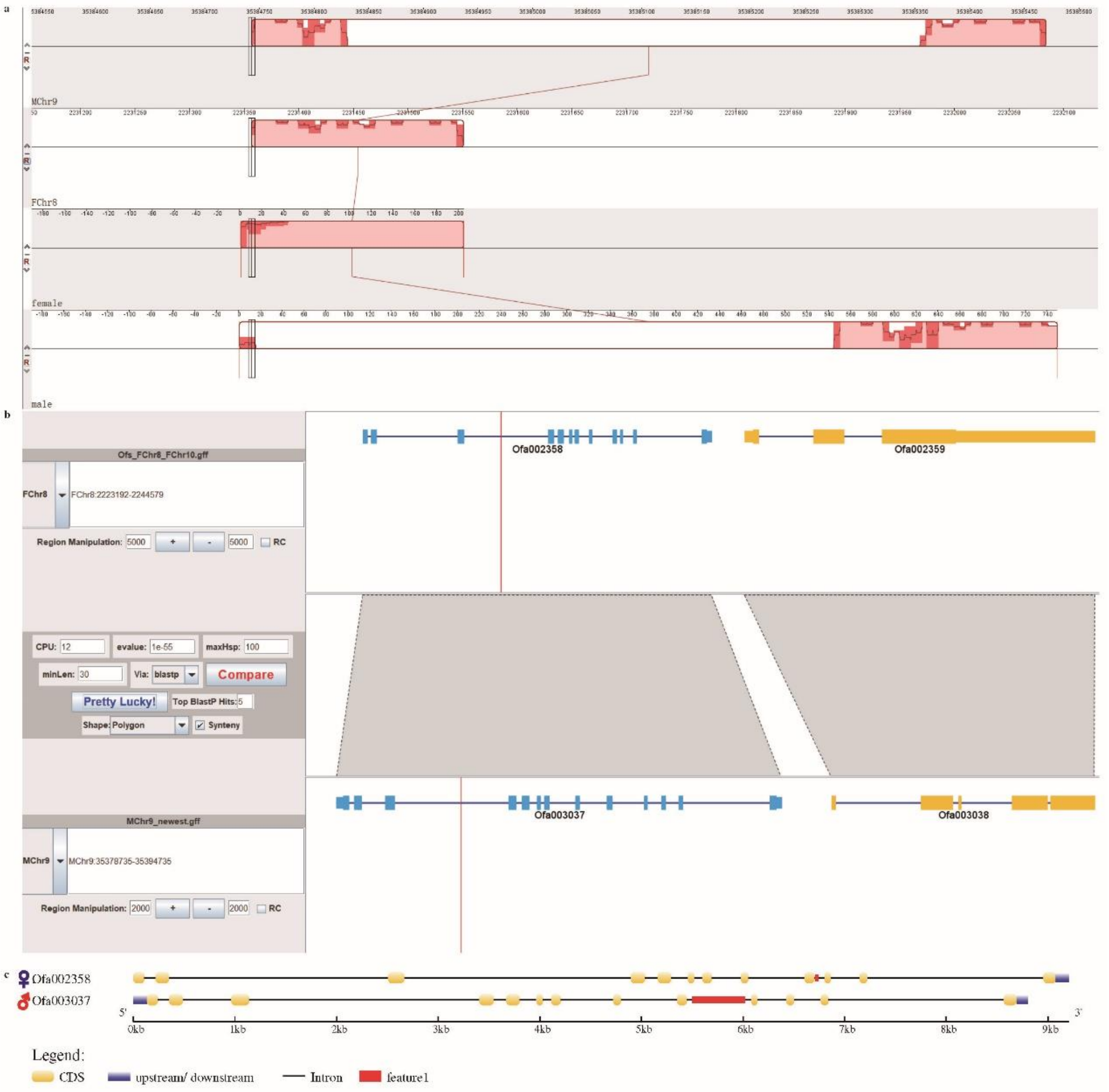

Figure 5: Representative genetic sex marker (insertion and deletion variant sites) located in the intron region of the *nuf2* gene in *O. fasciatus*.

(a) Collinearity relationships between male and female genetic sex markers and their association with the male and female genomes. (b) Location of male and female sex markers in the male and female genomes. (c) Unit composition of gene functional regions, occurrence of insertions and deletions of genetic sex markers, and sequence length patterns.

Validation of molecular markers for genetic sex identification of females and males

We selected markers located in the intergenic and intragenic regions for female and male genetic sex identification for validation testing. Using the female genome as a reference, a 599 bp base sequence was inserted into the male genome at position 880,783 bp on female chromosome 8 (after TTTAGC), corresponding to e-PCR

amplicons of 390 bp and 1008 bp, respectively (Figure 4, Figure S1). Sequence comparisons of the genetic sex markers showed that the genetic differences between male and female markers were mainly concentrated in the region of 200~800 bp. This variant locus was further confirmed as a valid insertion site using female fish as a reference genome, synthesized using genomic survey data from Illumina sequencing of male fish and CLR data from PacBio sequencing for read assignment (Figure 4, Figure S1). The results of PCR amplification and agarose gel electrophoresis were consistent with the results of e-PCR corresponding to two bands (390 bp and 1008 bp) for males and one band (390 bp) for females. According to the genetic sex identification marker localization, the marker lies between the *samd3* gene (sterile alpha motif.) (Ofa002334) and *elf3* gene (E74-like factor 3) (Ofa002335) in the female genome (Figure 4).

The intragenic markers obtained from screening were also used to test the effectiveness of female and male genetic sex identification in *O. fasciatus*. In the region corresponding to the intron of the female *nuf2* gene (component of the NDC80 kinetochore complex) (Ofa002358) on chromosome 8 (2,231,443 bp, after GCAAATA), a 518 bp base sequence was inserted into the intron of the male *nuf2* gene, corresponding to e-PCR amplified fragment sizes of 206 bp and 739 bp, respectively (Figure 5). With the female genome as a reference, we utilized the male Illumina survey data and PacBio CLR data for read assignment and further confirmed that this candidate variant site was a valid insertion site (Figure 6). Comparison of the sequences for the genetic sex marker showed that the genetic differences between the male and female markers were mainly in the region of 90~650 bp. The results of PCR amplification and agarose gel electrophoresis further showed that the males exhibited two bands (206 bp and 739 bp), and the females had one band (206 bp), in agreement with the e-PCR results (Figure 6).

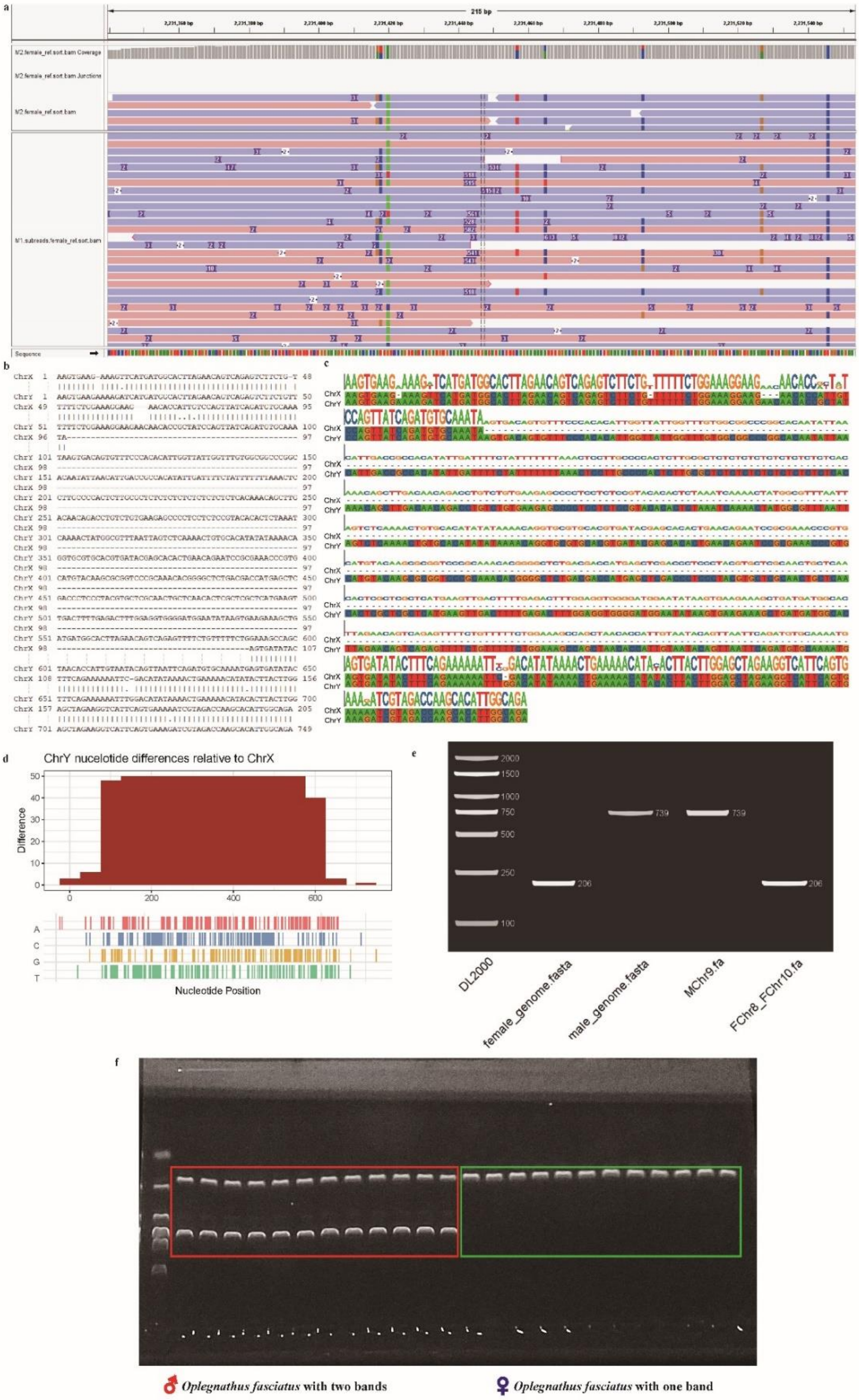

Figure 6: Validity testing of genetic markers located in the intronic region of the *nuf2* gene.

(a) Illumina and CLR clean data of Bam comparison to the genome for insertion/deletion site validity detection. (b) Nucleotide sequence comparison of markers for female and male genetic sex identification. (c) Comparison of genetic sex marker sequences based on the global alignment standard. (d) Visualization regions of nucleotide sequence heterogeneity in markers for female and male genetic sex identification. (e) Electronically simulated amplification results of the target marker primer (e-PCR). (f) Results of PCR amplification and agarose gel electrophoresis detection of target markers (two bands for males and one band for females).

In addition to the male insertion genetic sex markers, a male deletion genetic sex marker was also identified. In the region corresponding to female chromosome 8 from 1,161,186 bp (after GATGAGGAAAG) to 1,161,530 bp (before TATGAAGTCT), the males were missing a 343 bp base sequence, corresponding to e-PCR amplicon sizes of 557 bp and 928 bp, respectively (Figure 7). A comparison of sequence differences, Illumina survey data and PacBio CLR data read assignments all confirmed that the genetic differences between male and female markers were mainly concentrated in the 181~550 bp and 664~692 bp regions (Figure 8). PCR amplification and agarose gel electrophoresis assays showed two bands in males (557 bp and 928 bp) and one band in females (928 bp), which was consistent with the results of e-PCR (Figure 8). A similar genetic sex marker for the deletion locus in males was also identified in the region corresponding to chromosome 8 of females at 921,537 bp (after AAATGTGGCGG), with deletion of a 299 bp base sequence and insertion of a 119 bp multifragmented base sequence in males. PCR amplification and agarose gel electrophoresis tests showed two bands in males (748 bp and 926 bp) and one band in females (926 bp), consistent with the e-PCR results (Figure S2, Figure S3).

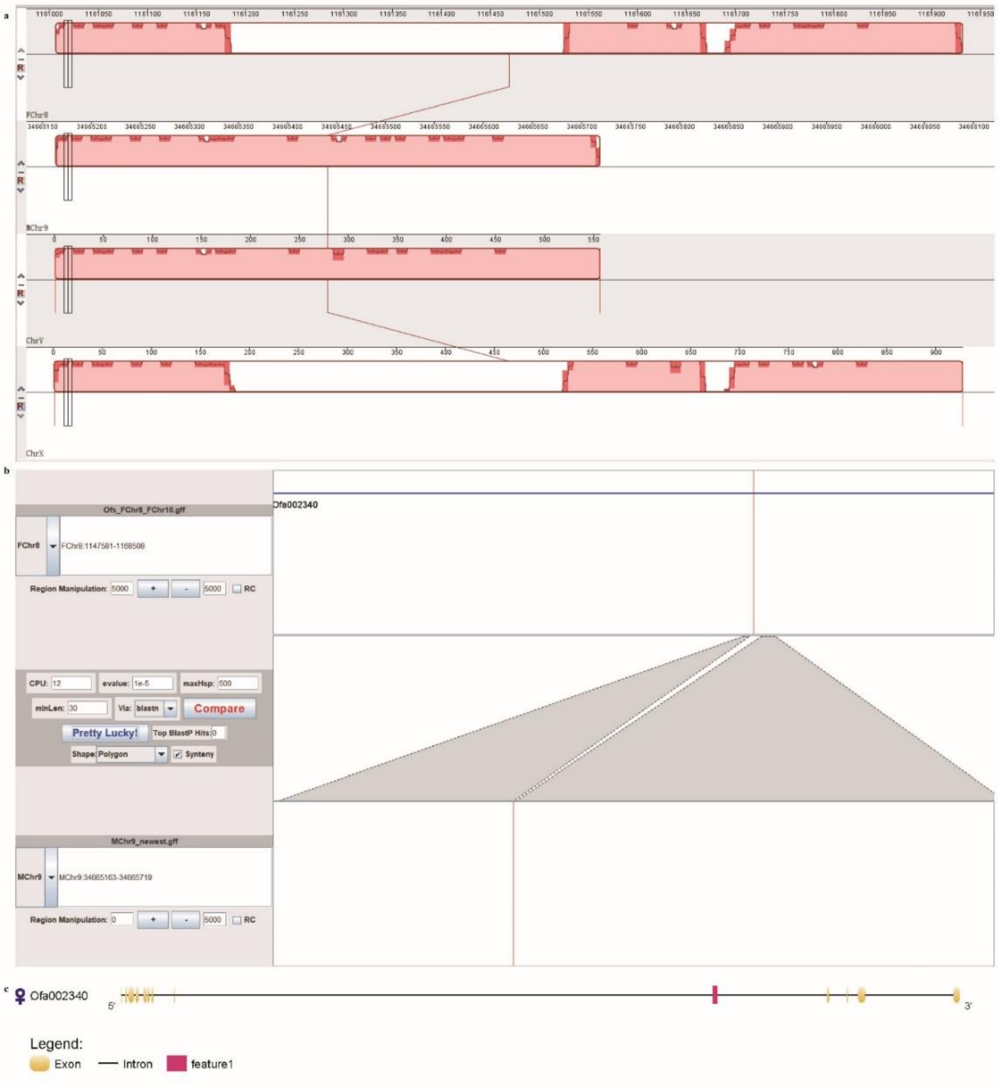

Figure 7: Representative male deletion genetic sex marker (insertion deletion variant sites).

(a) Collinearity relationships between male and female genetic sex markers and their association with the male and female genomes. (b) Location of male and female sex markers in the male and female genomes. (c) Unit composition of gene functional regions and occurrence of male deletion genetic sex markers.

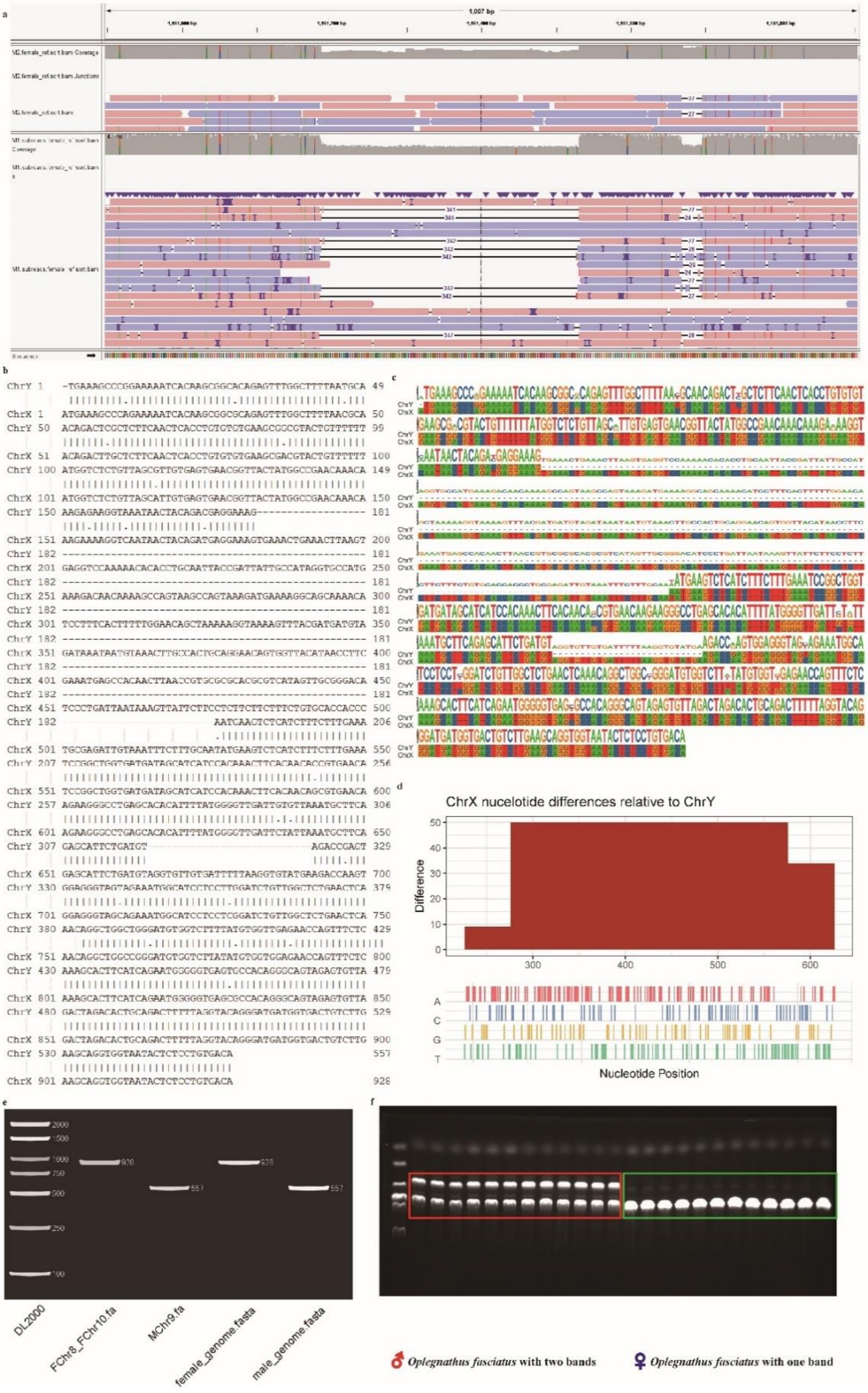

Figure 8: Validity testing of the male deletion genetic markers.

(a) Illumina and CLR clean data of Bam comparison to the genome for insertion/deletion site validity detection. (b) Nucleotide sequence comparison of markers for female and male genetic sex identification. (c) Comparison of genetic sex marker sequences based on the global alignment standard. (d) Visualization regions of nucleotide sequence heterogeneity in markers for female and male genetic sex identification. (e) Electronically simulated amplification results of the target marker primer (e-PCR). (f) Results of PCR amplification and agarose gel

electrophoresis detection of target markers (two bands for males and one band for females).

Enrichment analysis of functional genes for genetic sex markers

To further investigate the effect of large-segment insertion/deletion loci on gene function in female and male *O. fasciatus*, we initiated an enrichment analysis of functional genes for genetic sex markers. Using the female genome as a reference, 73 and 435 genes were affected by large-segment insertion/deletion sites located in the exonic region (Supplementary Female_reference_INS_DEL100_ExonSet_annotation.xlsx). The genes involved in insertion site markers were enriched in glycerophospholipid metabolism, blood circulation and other biological processes and involved in the molecular regulation of phosphatidylinositol deacylase activity, histone H3R2 demethylase activity, choline kinase activity, thioredoxin-disulfide reductase (NADP) activity, arylamine N-acetyltransferase activity, and calcium-dependent phospholipase A2 activity (Figure S4). Genes involved in the deletion site markers were enriched in biological processes such as regulation of morphogenesis of the epithelium, protein maturation, cell-matrix adhesion, cranial nerve formation, regulation of lysosome size, dendritic cell chemotaxis, and regulation of systemic arterial blood pressure by circulatory renin-angiotensin and participated in molecular regulation of glial cell-derived neurotrophic factor receptor binding, carbohydrate proton symporter activity, D-amino-acid oxidase activity, ATP-activated inwards rectifier potassium channel activity, ATP-dependent protein folding chaperone, sterol binding, and other molecular regulatory processes (Figure S4). In addition, KEGG results showed that the genes involved in the deletion site markers were enriched in regulatory pathways such as cholesterol metabolism and sphingolipid metabolism (Figure S4).

Using the male fish genome as a point of reference, it was observed that the exon region contained significant loci for large-segment insertions and deletions, encompassing 79 and 233 genes, respectively (Supplementary Male_reference_ INS_DEL100_ExonSet_annotation.xlsx). Furthermore, in conjunction with the findings pertaining to the deletion sites in female fish, gene enrichment analysis revealed a notable association with the regulation of vascular development and the promotion of myelin formation **(Figure S5)**. Additionally, the insertion site marker genes exhibited enrichment in functions such as stem cell population maintenance, response to sucrose, UDP-N-acetylgalactosamine metabolic process, digestive system

development, negative regulation of the execution phase of apoptosis, positive regulation of actin nucleation, negative regulation of cyclin-dependent protein serine/threonine kinase activity, intermediate filament bundle assembly, resolution of meiotic recombination intermediates, and biological regulation of certain processes (Figure S5). In addition, the related genes were involved in the molecular regulation of hepatocyte growth factor receptor activity, N6-isopentenyladenosine methyltransferase activity, ketohexokinase activity, keratin filament binding, sterol 12-alpha-hydroxylase activity, carbohydrate proton symporter activity, ATP-activated inwards rectifier potassium channel activity, alpha-N-acetylneuraminate alpha-2,8-sialyltransferase activity, superoxide-generating NADPH oxidase activator activity, and laminin binding (Figure S5). KEGG results showed that the genes involved in the insertion site markers were enriched in regulatory pathways such as caffeine metabolism, glycerophospholipid metabolism, selenium compound metabolism, and glycosylphosphatidylinositol (GPl) anchor biosynthesis (Figure S5). Deletion site marker genes were enriched in the reregulation of structural morphogenesis, negative regulation of the GIS phase transition of the cell cycle, pancreatic regeneration, regulation of deoxyribonuclease activity, activation of basophils, localization of proteins in the T-tubules, germline stem cell division, mitotic/amnestic phase transition of the mitotic cell cycle, and other biological processes (Figure S6). In addition, these genes participate in molecular regulatory processes such as phosphatidylinositol deacylase activity, 5-tyrosyl DNA phosphodiesterase activity, fatty acid α-hydroxylase activity, ATP adenylyltransferase activity, mRNA N6-methyladenosine dioxygenase activity, N-acylneuraminic acid cytidylyltransferase activity, vascular endothelial growth factor receptor 3 binding, phosphatidylethanolamine transfer activity, neurotransmitter receptor activity involved in the regulation of postsynaptic cytosolic calcium concentration, cosynaptic receptor binding, and structural formation of chromatin. KEGG results showed that genes involved in the insertion-deletion site markers were enriched in the regulatory pathways of tight junctions, caffeine metabolism, ubiquitin-mediated protein hydrolysis, cell adhesion molecules, and the apelin signalling pathway (Figure S6).

Discussion

The majority of fish exhibit limited sex chromosome differentiation, which poses challenges in identifying them based on chromosome morphology[10]. Additionally,

fish sex is influenced by environmental factors, particularly temperature, resulting in discrepancies between the physiological phenotype and genotype of sex[11]. Consequently, it is crucial within the field of aquaculture to discover a straightforward approach capable of rapidly determining the genetic composition of fish sex[50]. DNA molecular markers are created by exploiting the prevalence of polymorphisms in the genomic DNA of eukaryotic organisms, which serve as indicators of individual variations at the DNA level[7]. Analysis of relevant literature reveals that over the past decade, approximately 48% of genetic sex markers were derived from first-generation techniques such as RFLP, RAPD, SSR, and AFLP[50-61]. Meanwhile, second-generation techniques such as Illumina's SNP/InDel identified approximately 51% of sex markers[16, 62-67]. However, the advancement of sex-controlled breeding has faced limitations due to various factors, such as low throughput of the above methods, cumbersome experimental procedures, expensive development cost, high rate of false positives, unstable markers, and inconvenient on-site testing. With the emergence and rapid advancement of third-generation sequencing technology, exemplified by long-fragment read-length PacBio sequencing, the development of third-generation high-throughput sex markers based on insertions or deletions of large fragments (>100 bp) has become feasible[25, 33, 34]. In this study, *O. fasciatus* with the $X_1X_1X_2X_2/X_1X_2Y$ system served as the focal point[32-34, 36]. By utilizing the linear genomic information of male and female individuals acquired through PacBio sequencing, along with data from Illumina surveys and PacBio CLR/CCS, we extensively employed whole-genome variant site scanning and identification, high-throughput design of primers for the target regions, and e-PCR batch amplification (Figure 1). By employing a comprehensive approach involving genome-wide variant site scanning, high-throughput primer design within the desired region, and e-PCR batch amplification and validation methodologies, we successfully developed a database encompassing insertion/deletion loci in large segments (>100 bp) for both male and female *O. fasciatus* (Figure 2, Figure 3, Table 1, Table 2). This effort yielded a total of 3,645 (2,791 insertions and 854 deletions) and 3,872 (3,039 insertions and 833 deletions) genetic markers in females and males, respectively, serving as a reliable reference for sex identification. Following agarose gel electrophoresis, two amplification bands for males and one amplification band for females were successfully identified, enabling rapid differentiation between the sexes in *O. fasciatus*. This advancement in sex marker development, utilizing third-generation high-throughput and precise techniques, will expedite the progress of

genetic breeding and facilitate the establishment of genetic sex markers.

Prior research has demonstrated that DNA sequences containing insertions and deletions experience heightened selective pressure[68, 69]. Both deletions and insertions in DNA sequences, whether in noncoding or coding regions, inevitably impact the functionality of the original sequence to varying degrees. Furthermore, evidence suggests that the length and placement of insertions are not random but rather heavily influenced by the adjacent DNA sequence[68, 69]. In recent years, research has revealed that insertions and deletions contribute to 16.2% and 24.9% of the genetic polymorphisms in *Drosophila melanogaster* and *Caenorhabditis elegans*, respectively[70, 71]. Furthermore, investigations into human genetic variation have demonstrated that polymorphisms resulting from insertions and deletions account for 15.6% and 18% of genetic polymorphisms, respectively[72, 73]. The findings of this study indicate that insertions and deletions identified using the female and male *O. fasciatus* genomes as references constituted 25.26% and 26.99% of the genetic polymorphisms, respectively. Our study has provided additional insights into the occurrence of large-segment insertion-deletion events, revealing that these events occur in the intergenic region in 68% of cases, followed by the intronic region in 43% of cases (Figure 3, Table 1). Furthermore, we observed a higher proportion of deletion sites compared to insertion sites. A similar study conducted on the β-fibrinogen intron of birds reported similar findings[74]. The findings from the population genetic sex marker amplification analysis conducted in this study indicated that the insertion-deletion markers exhibited stability in both male and female populations of *O. fasciatus*. This observation provided additional evidence to support the theory that large-segment insertions and deletions are not a random occurrence but rather influenced by natural selection pressures. Notably, the genes located at these large insertion-deletion sites were predominantly associated with cholesterol metabolism, sphingolipid metabolism, and other glycerophospholipid metabolism processes. This observation indicates a potential direction for future investigations into the divergent functional traits between males and females.

Conclusions

These results highlighted the substantial prevalence of insertion and deletion events in the genetic makeup derived from the $X_1X_1X_2X_2/X_1X_2Y$ sex determination system of *O. fasciatus*. In our study, we successfully introduced a novel approach for the

development of genetic sex markers in *O. fasciatus*. This approach involved the utilization of a primer batch electronic design technique, which utilized the insertion and deletion loci as the target regions. Additionally, we implemented an electronic batch amplification technique, allowing for the efficient and rapid high-throughput identification and screening of a substantial number of variant sites. This methodology represents a significant advancement in the field of *O. fasciatus* genetic sex marker development. The study yielded a greater number of genetic sex markers with higher amplification efficiency compared to those generated by previous technologies spanning the first or second generation. This methodology was applicable not only to the efficient generation of genetic sex markers for the $X_1X_1X_2X_2/X_1X_2Y$ sex-determination system but also to the determination of genetic sex in various sex-determination types (including autosomal and sex chromosome differences). It is particularly well suited for sex-determination systems featuring heteromorphic chromosomes, such as the XY-type, ZW-type, XX/XY_1Y_2-type, ZZ/ZW_1W_2-type, and other species with different sex-determination mechanisms.

Ethics Statement

This research was approved by the Animal Care and Use committee of the Chinese Academy of Science.

Competing Interests

The authors declare no competing interests.

Data Availability

All additional supporting data are available in the GigaScience repository, GigaDB[75].

Availability of Supporting Source Code and Requirements

The code used for statistics on insertions and deletions as well as annotation scripts and base variant region visualization scripts are stored in jihuGitLab (https://jihulab.com/gigascience/sex-markers) with Username: xiaoyongshuang19@163.com and Login Password: Wwyxys1982 [75].

Abbreviations

CLR: Continuous Long Reads; CCS: circular consensus sequence; GO: Gene ontology; KEGG: Kyoto Encyclopaedia of Genes and Genomes.

References

1. Hughes JF, Skaletsky H, Pyntikova T, Graves TA, van Daalen SK, Minx PJ, et al. Chimpanzee and human Y chromosomes are remarkably divergent in structure and gene content. Nature. 2010;463 7280:536-9. doi:10.1038/nature08700.
2. Bitencourt JA, Sampaio I, Ramos RT, Vicari MR and Affonso PR. First Report of Sex Chromosomes in Achiridae (Teleostei: Pleuronectiformes) with Inferences About the Origin of the Multiple X(1)X(1)X(2)X(2)/X(1)X(2)Y System and Dispersal of Ribosomal Genes in Achirus achirus. Zebrafish. 2017;14 1:90-5. doi:10.1089/zeb.2016.1333.
3. Chen S, Zhang G, Shao C, Huang Q, Liu G, Zhang P, et al. Whole-genome sequence of a flatfish provides insights into ZW sex chromosome evolution and adaptation to a benthic lifestyle. Nat Genet. 2014;46 3:253-60. doi:10.1038/ng.2890.
4. Lu G and Luo M. Genomes of major fishes in world fisheries and aquaculture: Status, application and perspective. Aquaculture and Fisheries. 2020;5 4:163-73. doi:10.1016/j.aaf.2020.05.004.
5. Chen SL, Ji XS, Shao CW, Li WL, Yang JF, Liang Z, et al. Induction of mitogynogenetic diploids and identification of WW super-female using sex-specific SSR markers in half-smooth tongue sole (Cynoglossus semilaevis). Marine biotechnology (New York, NY). 2012;14 1:120-8. doi:10.1007/s10126-011-9395-2.
6. Pan ZJ, Li XY, Zhou FJ, Qiang XG and Gui JF. Identification of Sex-Specific Markers Reveals Male Heterogametic Sex Determination in Pseudobagrus ussuriensis. Marine biotechnology (New York, NY). 2015;17 4:441-51. doi:10.1007/s10126-015-9631-2.
7. Ma Y, Xiao Y, Xiao Z, Wu Y, Zhao H and Li J. Identification of Male-Specific Molecular Marker and Development of PCR-Based Genetic Sex Identification Technique in Spotted Knifejaw (Oplegnathus punctatus). Marine biotechnology (New York, NY). 2022;24 5:969-78. doi:10.1007/s10126-022-10160-w.
8. Wu Y, Xiao Y, Xiao Z, Ma Y, Zhao H and Li J. Identification of Male-Specific Molecular Markers by Recombination of RhoGEF10 Gene in Spotted Knifejaw (Oplegnathus punctatus). Genes (Basel). 2022;13 7 doi:10.3390/genes13071262.
9. Li XY and Gui JF. Diverse and variable sex determination mechanisms in vertebrates. Sci China Life Sci. 2018;61 12:1503-14. doi:10.1007/s11427-018-9415-7.
10. Chen J, Zhu Z and Hu W. Progress in research on fish sex determining genes. Water Biology and Security. 2022;1 1 doi:10.1016/j.watbs.2022.100008.
11. Shen Z-G and Wang H-P. Molecular players involved in temperature-dependent sex determination and sex differentiation in Teleost fish. Genetics Selection Evolution. 2014;46 1:26. doi:10.1186/1297-9686-46-26.
12. Dan C, Mei J, Wang D and Gui JF. Genetic differentiation and efficient sex-specific marker development of a pair of Y- and X-linked markers in yellow catfish. International journal of biological sciences. 2013;9 10:1043-9. doi:10.7150/ijbs.7203.
13. Liao X, Xu G and Chen SL. Molecular method for sex identification of half-smooth tongue sole (Cynoglossus semilaevis) using a novel sex-linked microsatellite marker. Int J Mol Sci. 2014;15 7:12952-8. doi:10.3390/ijms150712952.
14. Ning Y, Liu X, Wang ZY, Guo W, Li Y and Xie F. A genetic map of large yellow croaker Pseudosciaena crocea. Aquaculture. 2007;264 1-4:16-26. doi:10.1016/j.aquaculture.2006.12.042.
15. Xiao S, Wang P, Zhang Y, Fang L, Liu Y, Li JT, et al. Gene map of large yellow croaker

(Larimichthys crocea) provides insights into teleost genome evolution and conserved regions associated with growth. Scientific reports. 2015;5:18661. doi:10.1038/srep18661.

16. Lin A, Xiao S, Xu S, Ye K, Lin X, Sun S, et al. Identification of a male-specific DNA marker in the large yellow croaker (Larimichthys crocea). Aquaculture. 2017;480:116-22. doi:https://doi.org/10.1016/j.aquaculture.2017.08.009.
17. Tao W, Zhu X, Cao J, Xiao H, Dong J, Kocher TD, et al. Screening and characterization of sex-linked DNA markers in Mozambique tilapia (Oreochromis mossambicus). Aquaculture. 2022;557 doi:10.1016/j.aquaculture.2022.738331.
18. Aparicio S, Chapman J, Stupka E, Putnam N, Chia JM, Dehal P, et al. Whole-genome shotgun assembly and analysis of the genome of Fugu rubripes. Science (New York, NY). 2002;297 5585:1301-10. doi:10.1126/science.1072104.
19. Mitani H, Kamei Y, Fukamachi S, Oda S, Sasaki T, Asakawa S, et al. The medaka genome: why we need multiple fish models in vertebrate functional genomics. Genome dynamics. 2006;2:165-82. doi:10.1159/000095103.
20. Jones FC, Grabherr MG, Chan YF, Russell P, Mauceli E, Johnson J, et al. The genomic basis of adaptive evolution in threespine sticklebacks. Nature. 2012;484 7392:55-61. doi:10.1038/nature10944.
21. Davidson WS, Koop BF, Jones SJ, Iturra P, Vidal R, Maass A, et al. Sequencing the genome of the Atlantic salmon (Salmo salar). Genome biology. 2010;11 9:403. doi:10.1186/gb-2010-11-9-403.
22. Shi L, Guo Y, Dong C, Huddleston J, Yang H, Han X, et al. Long-read sequencing and de novo assembly of a Chinese genome. Nature Communications. 2016;7 1:12065. doi:10.1038/ncomms12065.
23. VanBuren R, Bryant D, Edger PP, Tang H, Burgess D, Challabathula D, et al. Single-molecule sequencing of the desiccation-tolerant grass Oropetium thomaeum. Nature. 2015;527 7579:508-11. doi:10.1038/nature15714.
24. Vij S, Kuhl H, Kuznetsova IS, Komissarov A, Yurchenko AA, Van Heusden P, et al. Chromosomal-Level Assembly of the Asian Seabass Genome Using Long Sequence Reads and Multi-layered Scaffolding. PLoS genetics. 2016;12 4:e1005954. doi:10.1371/journal.pgen.1005954.
25. Burton JN, Adey A, Patwardhan RP, Qiu R, Kitzman JO and Shendure J. Chromosome-scale scaffolding of de novo genome assemblies based on chromatin interactions. Nature biotechnology. 2013;31 12:1119-25. doi:10.1038/nbt.2727.
26. Xiao Y, Liu J, Wei J, Xiao Z, Li J and Ma Y. Improved high-quality reference genome of red drum facilitates the processes of resistance-related gene exploration. Sci Data. 2023;10 1:774. doi:10.1038/s41597-023-02699-7.
27. Schembri PJ, Bodilis P, Evans J and Francour PJAIeP. Occurrence of barred knifejaw, Oplegnathus fasciatus (Actinopterygii: Perciformes: Oplegnathidae), in Malta (Central Mediterranean) with a discussion on possible modes of entry. 2010;40 2:101-4.
28. Mundy BCJBMBiZ. Checklist of the fishes of the Hawaiian Archipelago. 2005;6:1-704.
29. An HS, Kim MJ, Hong SWJG and Genomics. Genetic diversity of rock bream Oplegnathus fasciatus in Southern Korea. 2008;30 5:451-9.
30. Xiao Y, Li J, Ren G, Ma D, Wang Y, Xiao Z, et al. Pronounced population genetic differentiation in the rock bream Oplegnathus fasciatus inferred from mitochondrial DNA sequences. 2016;27

3:2045-52.
31. Xu D, Lou B, Bertollo LAC and Cioffi MdBJMC. Chromosomal mapping of microsatellite repeats in the rock bream fish Oplegnathus fasciatus, with emphasis of their distribution in the neo-Y chromosome. 2013;6:1-6.
32. Xue R, An H, Liu Q, Xiao Z, Wang Y and Li JJOLS. Karyotype and Ag-NORs in male and female of Oplegnathus punctatus. 2016;47:626-32.
33. Xiao Y, Xiao Z, Ma D, Liu J and Li JJG. Genome sequence of the barred knifejaw Oplegnathus fasciatus (Temminck & Schlegel, 1844): The first chromosome-level draft genome in the family Oplegnathidae. 2019;8 3:giz013.
34. Xiao Y, Xiao Z, Ma D, Zhao C, Liu L, Wu H, et al. Chromosome-Level Genome Reveals the Origin of Neo-Y Chromosome in the Male Barred Knifejaw Oplegnathus fasciatus. iScience. 2020;23 4:101039. doi:https://doi.org/10.1016/j.isci.2020.101039.
35. Xu T, Shao C, Liao X, Ji X and Chen SJCg. Isolation and characterization of polymorphic microsatellite DNA markers in the rock bream (Oplegnathus fasciatus). 2009;10:527-9.
36. Xu D, Lou B, Xu H, Li S and Geng ZJMb. Isolation and characterization of male-specific DNA markers in the rock bream Oplegnathus fasciatus. 2013;15:221-9.
37. Gong J, Zhao J, Ke Q, Li B, Zhou Z, Wang J, et al. First genomic prediction and genome‐ wide association for complex growth‐ related traits in Rock Bream (Oplegnathus fasciatus). 2022;15 4:523-36.
38. NCBI Sequence Read Archive https://identifiersorg/ncbi/insdcsra:SRP220007. 2020.
39. Xiao Y, Xiao Z, Ma D, Li J and Liu J. Supporting data for "Genome sequence of the barred knifejaw Oplegnathus fasciatus (Temminck & Schlegel, 1844): the first chromosome-level draft genome in the family Oplegnathidae". GigaScience Database. 2019:http://dx.doi.org/10.5524/100556.
40. Chen C, Wu Y and Xia R. A painless way to customize Circos plot: From data preparation to visualization using TBtools. iMeta. 2022;1 3 doi:10.1002/imt2.35.
41. Chen C, Chen H, Zhang Y, Thomas HR, Frank MH, He Y, et al. TBtools: An Integrative Toolkit Developed for Interactive Analyses of Big Biological Data. Mol Plant. 2020;13 8:1194-202. doi:10.1016/j.molp.2020.06.009.
42. Li H. Minimap2: pairwise alignment for nucleotide sequences. J Bioinformatics. 2018;34 18:3094-100.
43. Li H and Durbin RJb. Fast and accurate short read alignment with Burrows–Wheeler transform. 2009;25 14:1754-60.
44. Krzywinski M, Schein J, Birol I, Connors J, Gascoyne R, Horsman D, et al. Circos: an information aesthetic for comparative genomics. 2009;19 9:1639-45.
45. Kearse M, Moir R, Wilson A, Stones-Havas S, Cheung M, Sturrock S, et al. Geneious Basic: an integrated and extendable desktop software platform for the organization and analysis of sequence data. 2012;28 12:1647-9.
46. Darling AE, Mau B and Perna NTJPo. progressiveMauve: multiple genome alignment with gene gain, loss and rearrangement. 2010;5 6:e11147.
47. Zhou L, Feng T, Xu S, Gao F, Lam TT, Wang Q, et al. ggmsa: A visual exploration tool for multiple sequence alignment and associated data. 2022;23 4:bbac222.
48. Hu B, Jin J, Guo A-Y, Zhang H, Luo J and Gao GJB. GSDS 2.0: an upgraded gene feature visualization server. 2015;31 8:1296-7.

49. Zhou Y, Zhou B, Pache L, Chang M, Khodabakhshi AH, Tanaseichuk O, et al. Metascape provides a biologist-oriented resource for the analysis of systems-level datasets. 2019;10 1:1523.
50. Mei J and Gui JF. Genetic basis and biotechnological manipulation of sexual dimorphism and sex determination in fish. Sci China Life Sci. 2014;44 12:5.
51. Agawa Y, Saiki T, Miyamoto T, Ikeue Y, Honryo T and Sawada YJFS. Identification of the male sex-linked DNA sequence of cultured white trevally Pseudocaranx dentex. 2022;88 2:319-27.
52. Goyat S, Grewal A, Singh D, Katiyar R, Tewari S, Nainwal R, et al. Sex-linked AFLP marker identification in dioecious Betelvine (Piper betle L.). 2019;94 4:422-7.
53. Hashem MHJDJoVS. Early sexing of Tilapia nilotica (Oreochromis niloticus) by using short sequence repeats (SSRs) molecular markers. 2022;8 2:22-5.
54. Hua J, Qiang J, Tao Y, Li Y, Lu S and Bing XJAR. Development and validation of a PCR-RFLP/TaqMan MGB probe method for rapid sex identification of largemouth bass (Micropterus salmoides). 2023;30:101593.
55. Jia X, Lu J, Tang X, Fan Y and Gao YJBPS. A new method for molecular sex identification in the emu (Dromaius novaehollandiae). 2023:1-6.
56. Lajan S and Al-Barzinji YJIJoAS. Detection of quantitative loci correlation with growth traites in local quail using PCR-RFLP technique. 2022;53 1:16-26.
57. Law D, Ping VC, Yee TC, HR ED, Mohamed MH, Fazry S, et al. Use of amplified fragment length polymorphism and sequence characterized amplified region marker for identifying the sex of the Oxyeleotris marmorata. 2021.
58. Mohamed G, Youssef M, Mohamed E and Mahgoub AAJAJoAS. Identification of Sex-specific Molecular Markers in Barbel and Nile Carp using SCoT and ISSR Markers. 2021;52 1:60-73.
59. Panagiotopoulou H, Marzecki K, Gawor J, Kuhl H, Koper M, Weglenski P, et al. Extensive search of genetic sex markers in Siberian (Acipenser baerii) and Atlantic (A. oxyrinchus) sturgeons. 2023;573:739517.
60. Vaux F, Aycock HM, Bohn S, Rasmuson LK and O’Malley KG. Sex identification PCR–RFLP assay tested in eight species of Sebastes rockfish. Conservation Genetics Resources. 2020;12 4:541-4. doi:10.1007/s12686-020-01150-y.
61. Wen M, Zhang Y, Wang S, Wang Y, Qin Q, Tao M, et al. Characterization of sex locus and sex-specific sequences in the mandarin fishes. 2022;561:738650.
62. Zheng S, Wang X, Zhang S, Long J, Tao W, Li M, et al. Screening and characterization of sex-linked DNA markers and marker-assisted selection in the Southern catfish (Silurus meridionalis). Aquaculture. 2020;517:734783. doi:https://doi.org/10.1016/j.aquaculture.2019.734783.
63. Liu H, Pang M, Yu X, Zhou Y, Tong J and Fu B. Sex-specific markers developed by next-generation sequencing confirmed an XX/XY sex determination system in bighead carp (Hypophthalmichehys nobilis) and silver carp (Hypophthalmichthys molitrix). DNA Res. 2018;25 3:257-64. doi:10.1093/dnares/dsx054.
64. Han C, Zhu Q, Lu H, Wang C, Zhou X, Peng C, et al. Screening and characterization of sex-specific markers developed by a simple NGS method in mandarin fish (Siniperca chuatsi). Aquaculture. 2020;527:735495. doi:https://doi.org/10.1016/j.aquaculture.2020.735495.
65. Palaiokostas C, Bekaert M, Khan MG, Taggart JB, Gharbi K, McAndrew BJ, et al. Mapping and validation of the major sex-determining region in Nile tilapia (Oreochromis niloticus L.) using RAD sequencing. 2013;8 7:e68389.
66. Wang L, Xie N, Shen Y, Ye B, Yue GH and Feng XJMB. Constructing high-density genetic

maps and developing sexing markers in northern snakehead (Channa argus). 2019;21:348-58.

67. Wu X, Zhao L, Fan Z, Lu B, Chen J, Tan D, et al. Screening and characterization of sex-linked DNA markers and marker-assisted selection in blue tilapia (Oreochromis aureus). Aquaculture. 2021;530:735934. doi:https://doi.org/10.1016/j.aquaculture.2020.735934.

68. DN CJHG. Mechanisms of insertional mutagenesis in human genes causing genetic disease. 1991;87:409-15.

69. Krawczak M and Cooper DNJHg. Gene deletions causing human genetic disease: mechanisms of mutagenesis and the role of the local DNA sequence environment. 1991;86:425-41.

70. Berger J, Suzuki T, Senti K-A, Stubbs J, Schaffner G and Dickson BJJNg. Genetic mapping with SNP markers in Drosophila. 2001;29 4:475-81.

71. Wicks SR, Yeh RT, Gish WR, Waterston RH and Plasterk RHJNg. Rapid gene mapping in Caenorhabditis elegans using a high density polymorphism map. 2001;28 2:160-4.

72. Dawson E, Chen Y, Hunt S, Smink LJ, Hunt A, Rice K, et al. A SNP resource for human chromosome 22: extracting dense clusters of SNPs from the genomic sequence. 2001;11 1:170-8.

73. Devine SE, Mills RE, Luttig CT, Larkins CE, Beauchamp A, Tsui C, et al. An initial map of insertion and deletion (INDEL) variation in the. 2006.

74. Prychitko TM, Moore WSJMB and Evolution. Alignment and phylogenetic analysis of β-fibrinogen intron 7 sequences among avian orders reveal conserved regions within the intron. 2003;20 5:762-71.

75. Xiao Y, Xiao Z, Ma Y, Zhao H, Wu Y, Ma D, et al. Datasets and markers development workflow run scripts for high-throughput exploitation of genetic sex markers. figshare. 2023.

Figure legends

Figure 1. High-throughput exploitation process of genetic sex markers for *O. fasciatus*.

Figure 2. Distribution statistics of insertion and deletion fragment positions in genomic functional regions of *O. fasciatus*.

(a) Distribution statistics of insertion variant loci using the female fish genome as a reference. (b) Distribution statistics of deletion variant length using the female fish genome as a reference. (c) Distribution statistics of insertion variant loci using the male fish genome as a reference. (d) Distribution statistics of deletion variant loci using the male fish genome as a reference.

Figure 3. The covariance of genomic sequence differences between the male and female genomes of *O. fasciatus* and distribution statistics of variant sites.

(a) The female genome (reference) of *O. fasciatus* vs. the male genome. (b) The male genome (reference) of *O. fasciatus* vs. the female genome. (c) Distribution statistics of variant loci using the female fish genome as a reference. (d) Distribution statistics of variant loci using the male fish genome as a reference. The outer to inner circles show chromosome information, insertion site distribution, deletion site distribution and base substitution distribution, respectively. (e) **-** (f) Dual-valued histograms showing the number of insertion/deletion sites per chromosome with reference to the male and female genomes, respectively, where chromosome 9 in males was homologous to chromosomes 8 and 10 in females.

Figure 4. Representative genetic sex marker (insertion deletion variant sites) located in the intergenic region of the *O. fasciatus* genome.

(a) Location of male and female sex markers in the male and female genomes. (b) Illumina and CLR clean data of Bam comparison to the genome for insertion/deletion site validity detection. (c) Nucleotide sequence comparison of markers for female and male genetic sex identification. (d) Visualization of regions of nucleotide sequence heterogeneity in markers for female and male genetic sex identification. (e) Electronically simulated amplification results of the target marker primer (e-PCR). (f) Results of PCR amplification and agarose gel electrophoresis detection of target markers (two bands for males and one band for females).

Figure 5. Representative genetic sex marker (insertion and deletion variant sites) located in the intron region of the *nuf2* gene in *O. fasciatus*.

(a) Collinearity relationships between male and female genetic sex markers and their association with the male and female genomes. (b) Location of male and female sex markers in the male and female genomes. (c) Unit composition of gene functional regions, occurrence of insertions and deletions of genetic sex markers, and sequence length patterns.

Figure 6. Validity testing of genetic markers located in the intronic region of the *nuf2* gene.
(a) Illumina and CLR clean data of Bam comparison to the genome for insertion/deletion site validity detection. (b) Nucleotide sequence comparison of markers for female and male genetic sex identification. (c) Comparison of genetic sex marker sequences based on the global alignment standard. (d) Visualization regions of nucleotide sequence heterogeneity in markers for female and male genetic sex identification. (e) Electronically simulated amplification results of the target marker primer (e-PCR). (f) Results of PCR amplification and agarose gel electrophoresis detection of target markers (two bands for males and one band for females).

Figure 7. Representative male deletion genetic sex marker (insertion deletion variant sites).
(a) Collinearity relationships between male and female genetic sex markers and their association with the male and female genomes. (b) Location of male and female sex markers in the male and female genomes. (c) Unit composition of gene functional regions and occurrence of male deletion genetic sex markers.

Figure 8. Validity testing of the male deletion genetic markers.
(a) Illumina and CLR clean data of Bam comparison to the genome for insertion/deletion site validity detection. (b) Nucleotide sequence comparison of markers for female and male genetic sex identification. (c) Comparison of genetic sex marker sequences based on the global alignment standard. (d) Visualization regions of nucleotide sequence heterogeneity in markers for female and male genetic sex identification. (e) Electronically simulated amplification results of the target marker primer (e-PCR). (f) Results of PCR amplification and agarose gel electrophoresis detection of target markers (two bands for males and one band for females).

Figure1

Click here to access/download;Figure;Figure 1.pdf

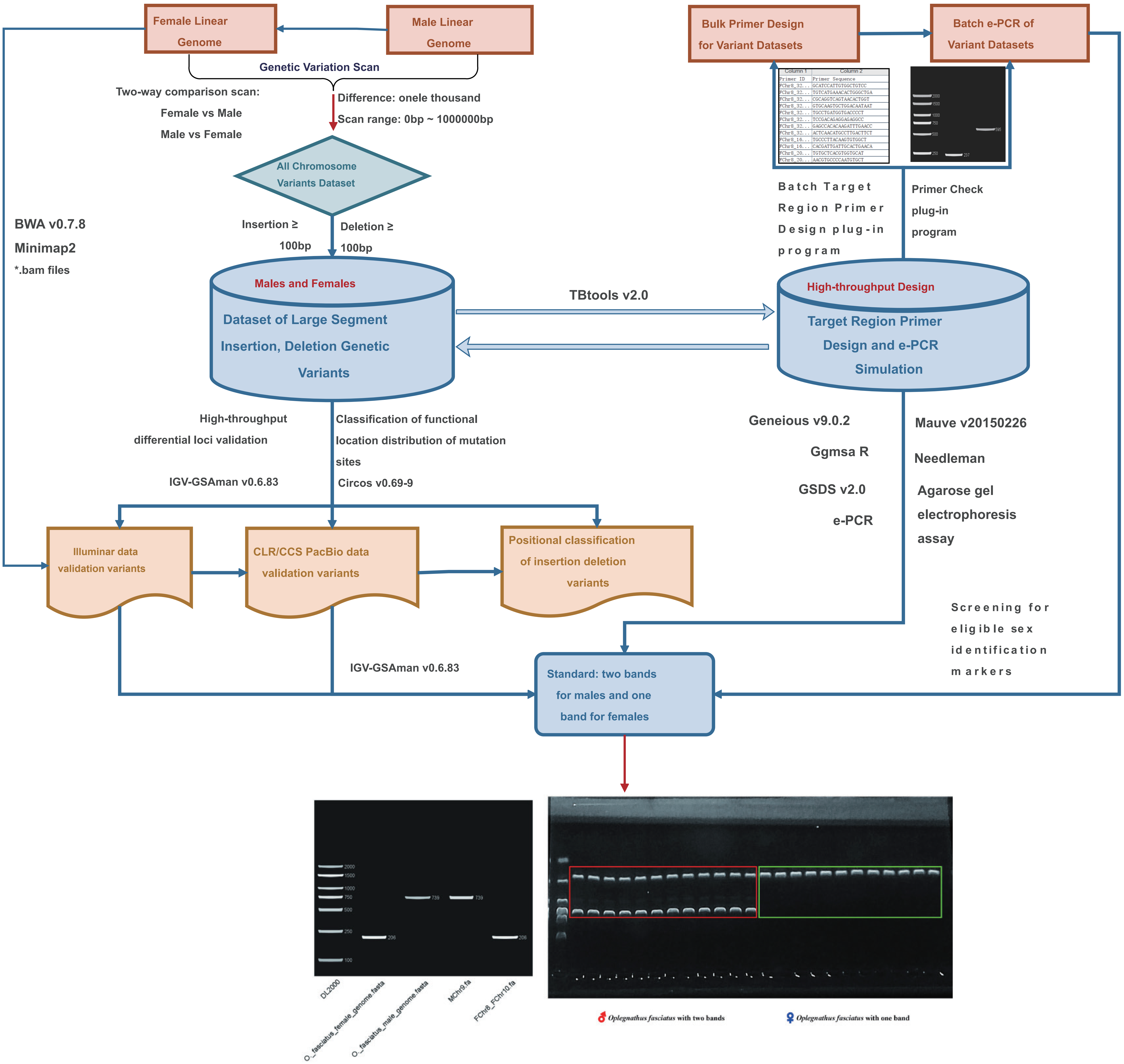

Figure2

Click here to access/download;Figure;Figure 2.pdf

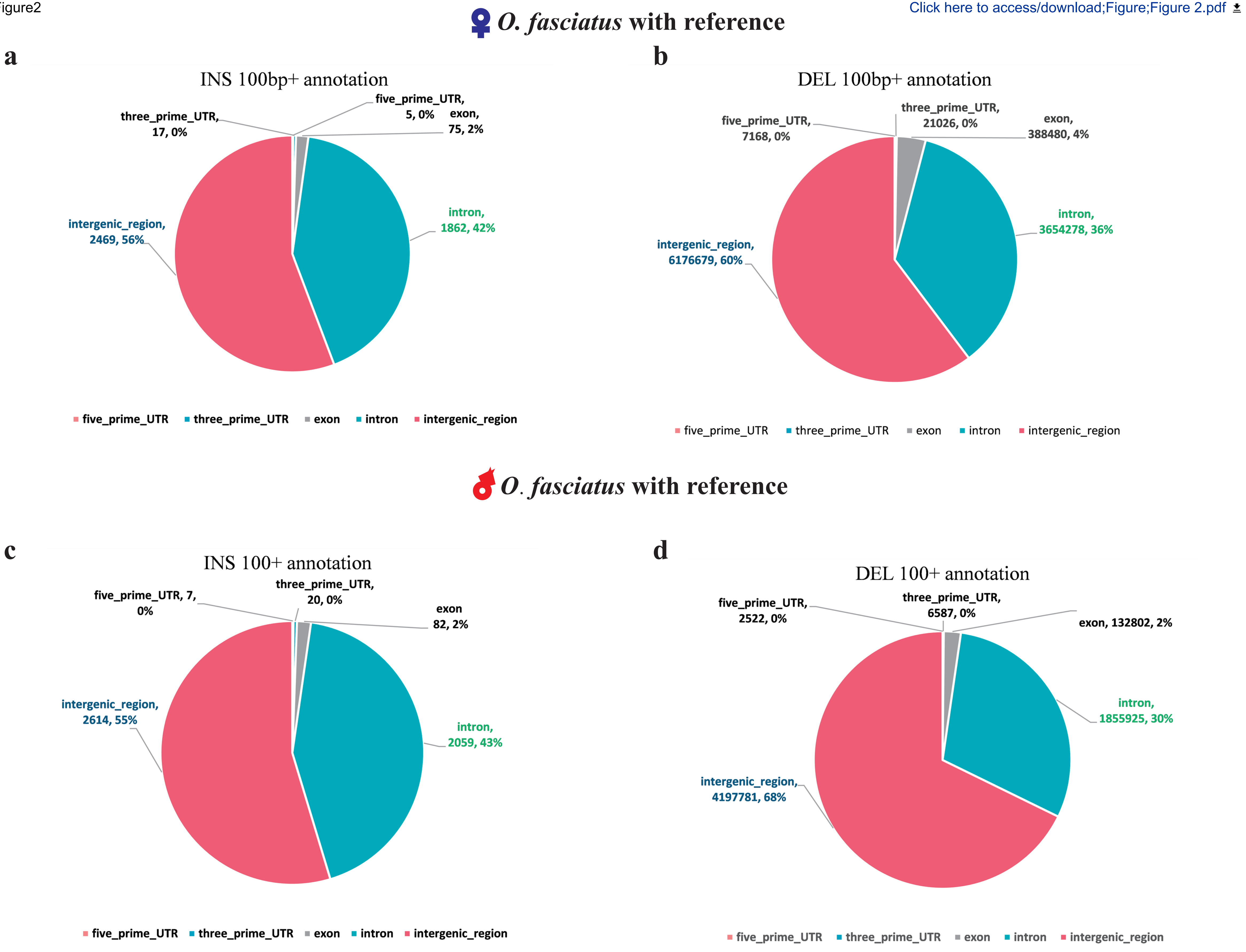

Figure3

Click here to access/download;Figure;Figure 3.pdf

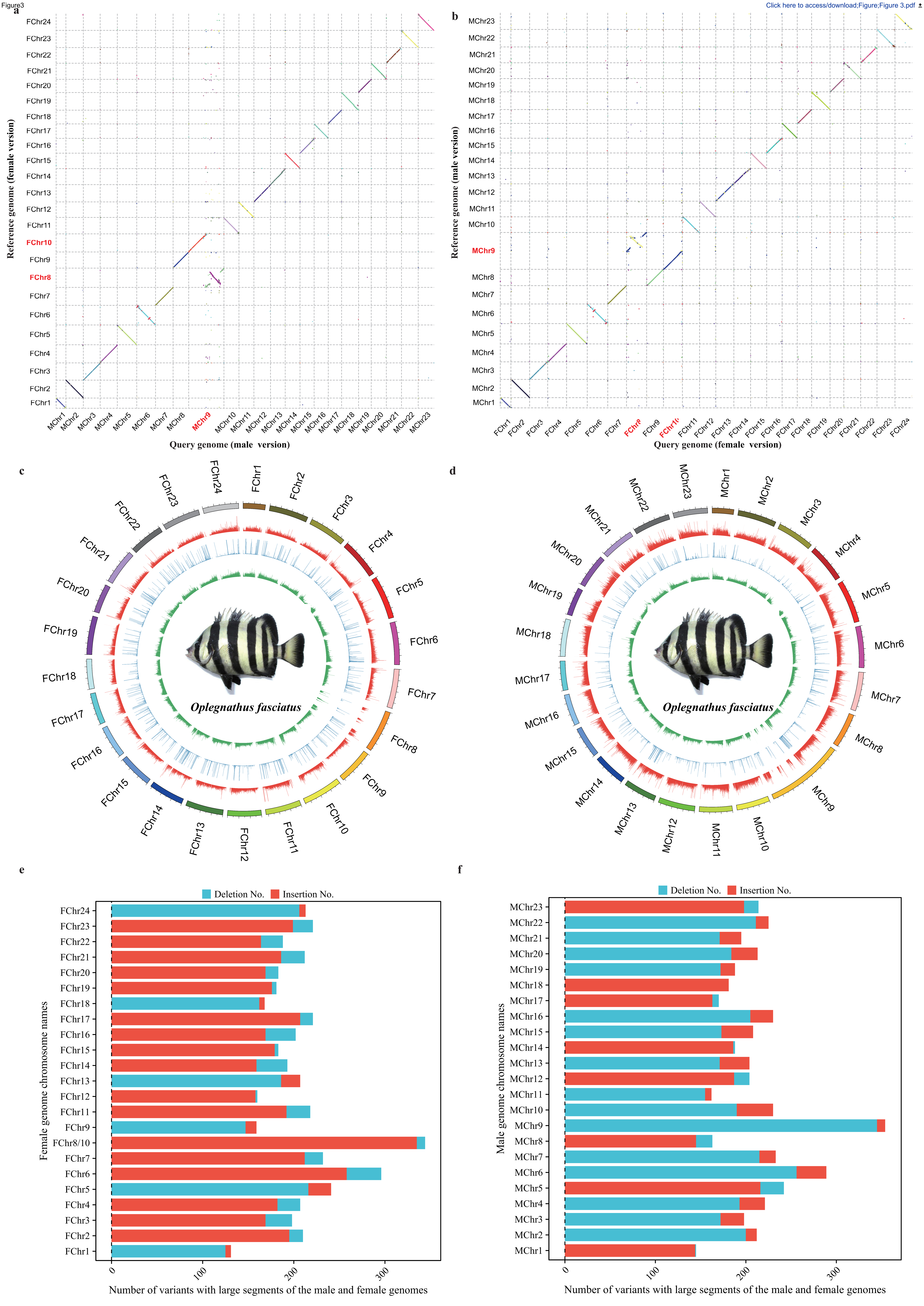

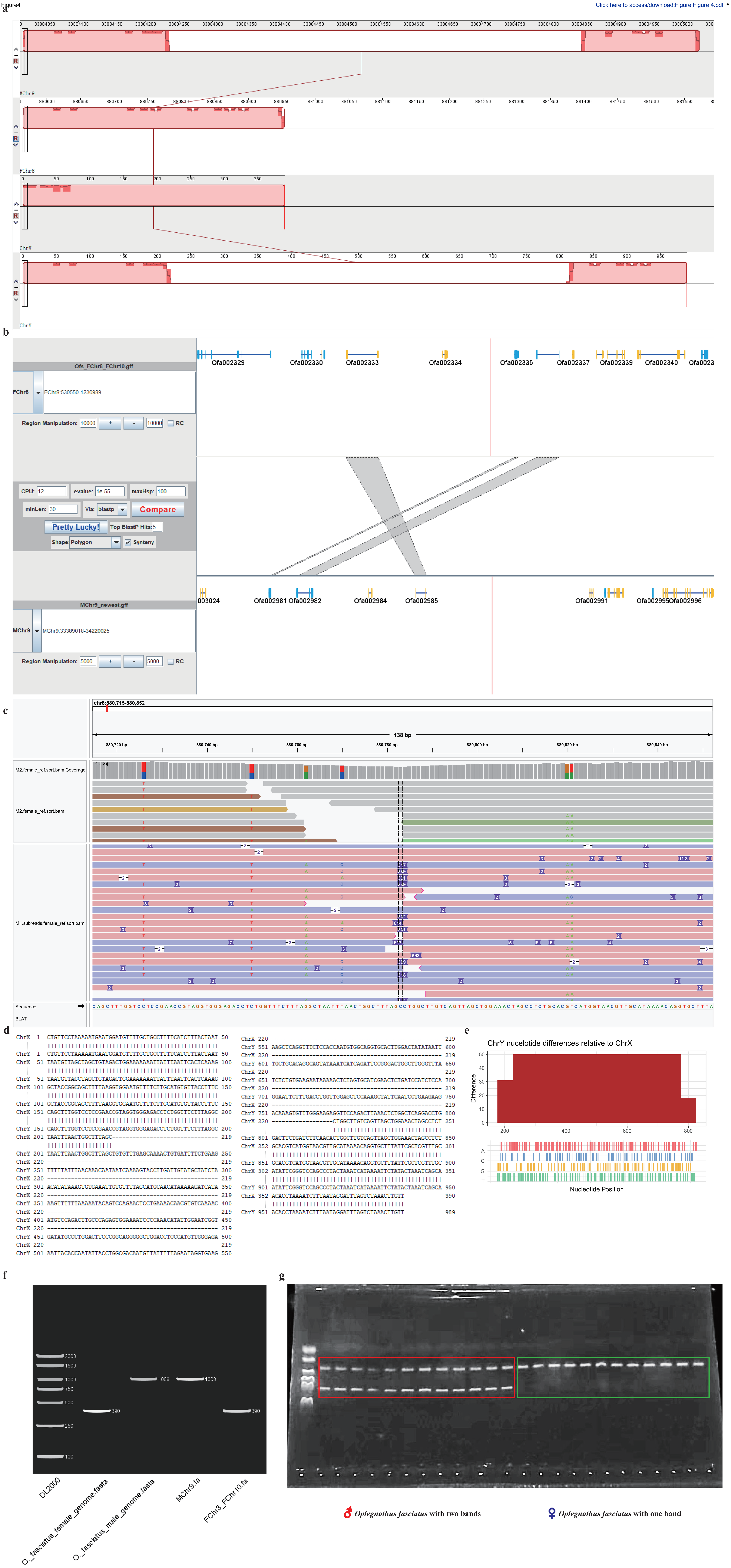
a
MChr9
FChr8
ChrX
ChrY
b
Ofs_FChr8_FChr10.gff
FChr8:530550-1230989
Region Manipulation:
Compare
Pretty Lucky!
Top BlastP Hits:
Synteny
MChr9_newest.gff
MChr9:33389018-34220025
c
chr8:880,715-880,852
138 bp
M2.female_ref.sort.bam Coverage
M2.female_ref.sort.bam
M1.subreads.female_ref.sort.bam
Sequence
BLAT
d
e
ChrY nucelotide differences relative to ChrX
Difference
Nucleotide Position
f
DL2000
O._fasciatus_female_genome.fasta
O._fasciatus_male_genome.fasta
MChr9.fa
FChr8_FChr10.fa
g
♂ Oplegnathus fasciatus with two bands
♀ Oplegnathus fasciatus with one band

Figure5

Click here to access/download;Figure;Figure 5.pdf

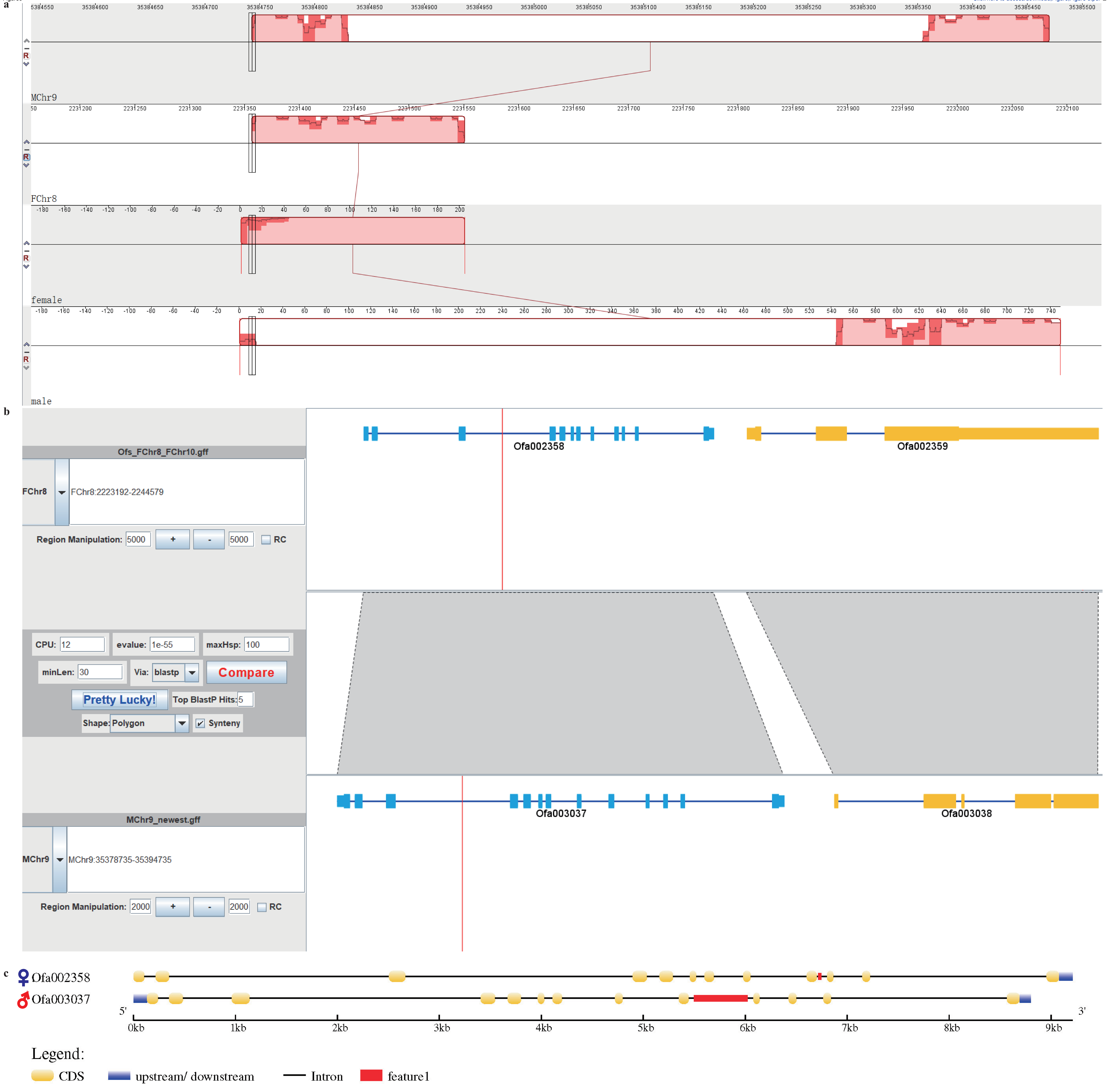

Figure6
Click here to access/download;Figure;Figure 6.pdf

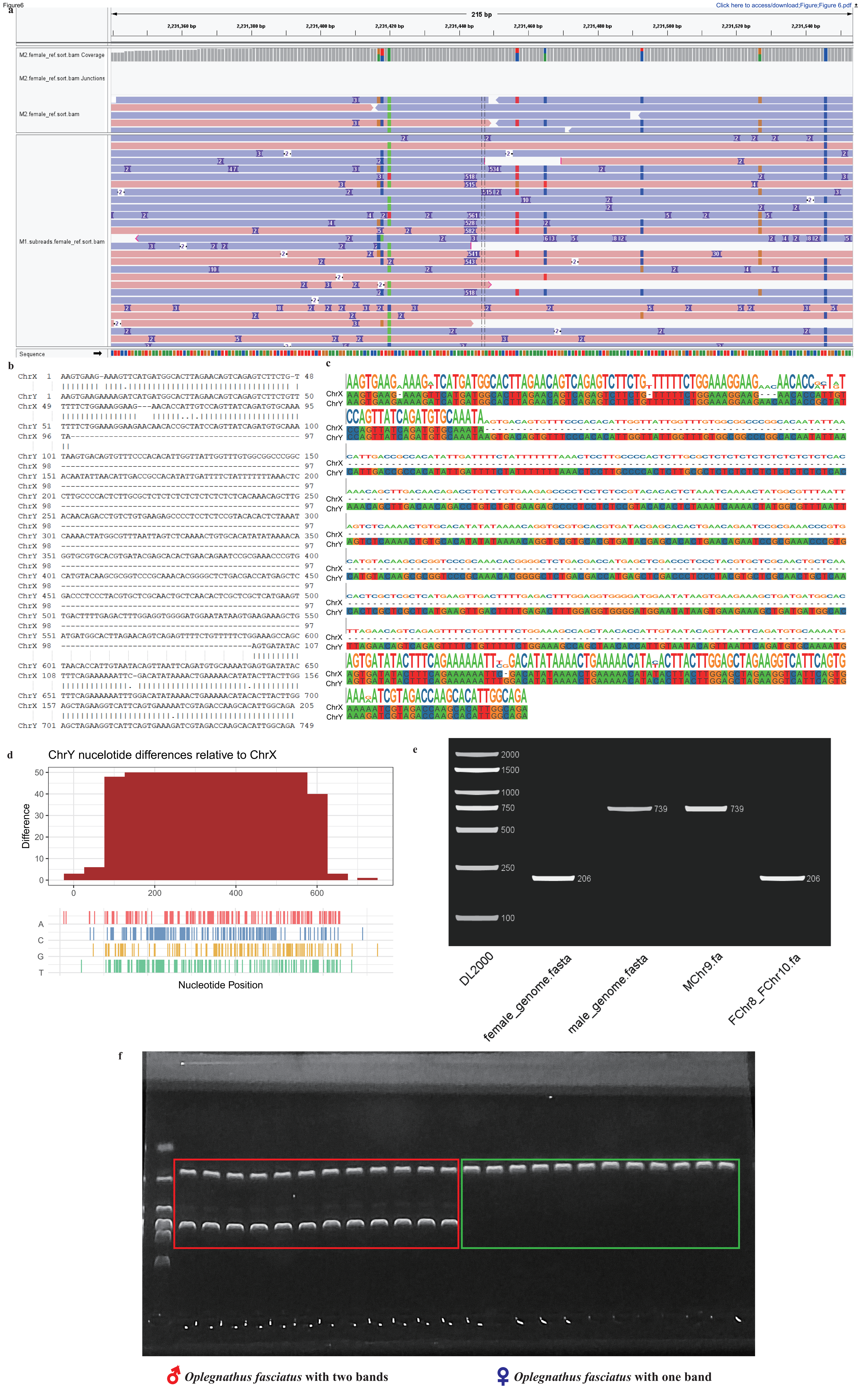

Figure7
Click here to access/download;Figure;Figure 7.pdf

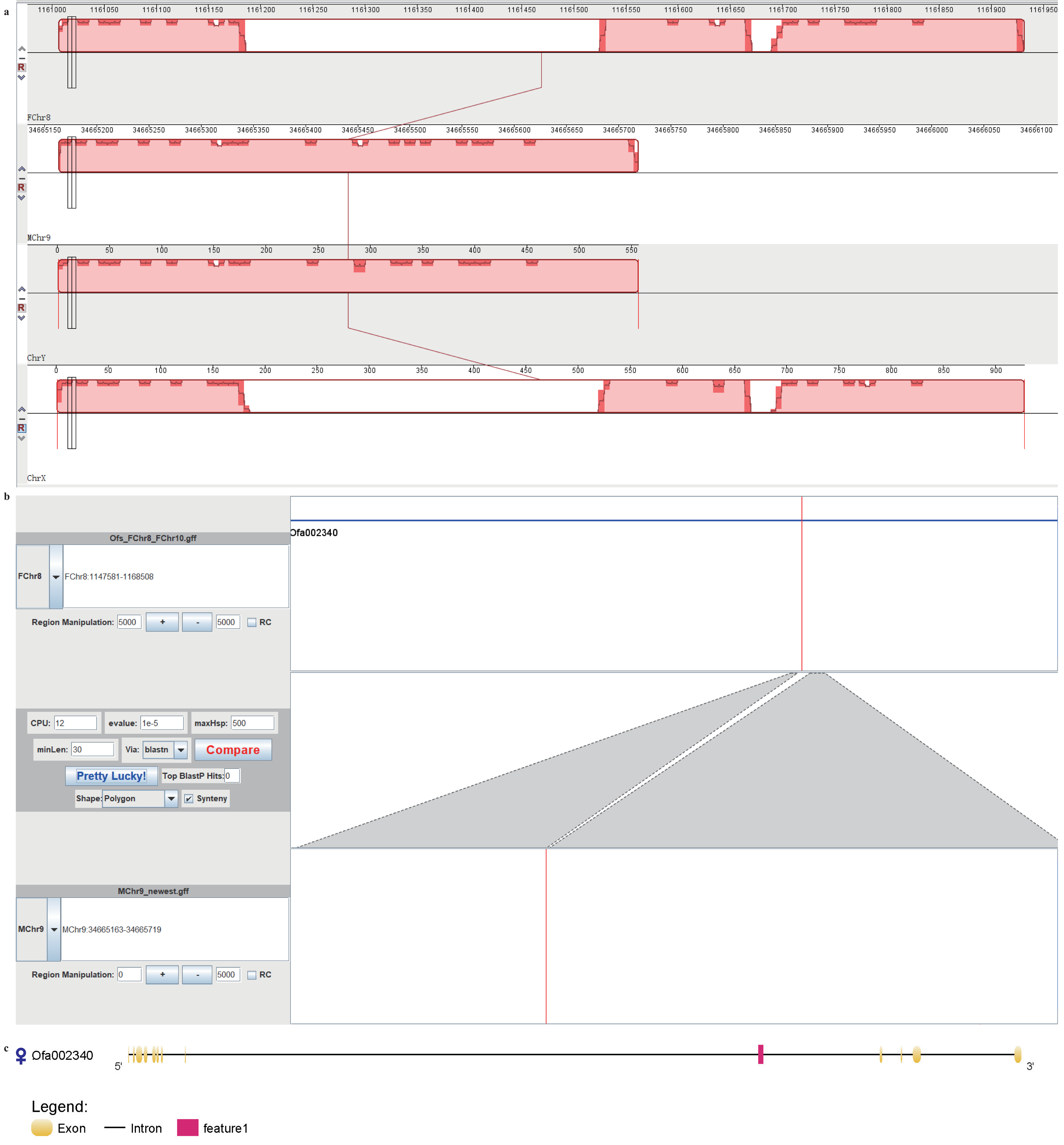

Click here to access/download;Figure;Figure 8.pdf

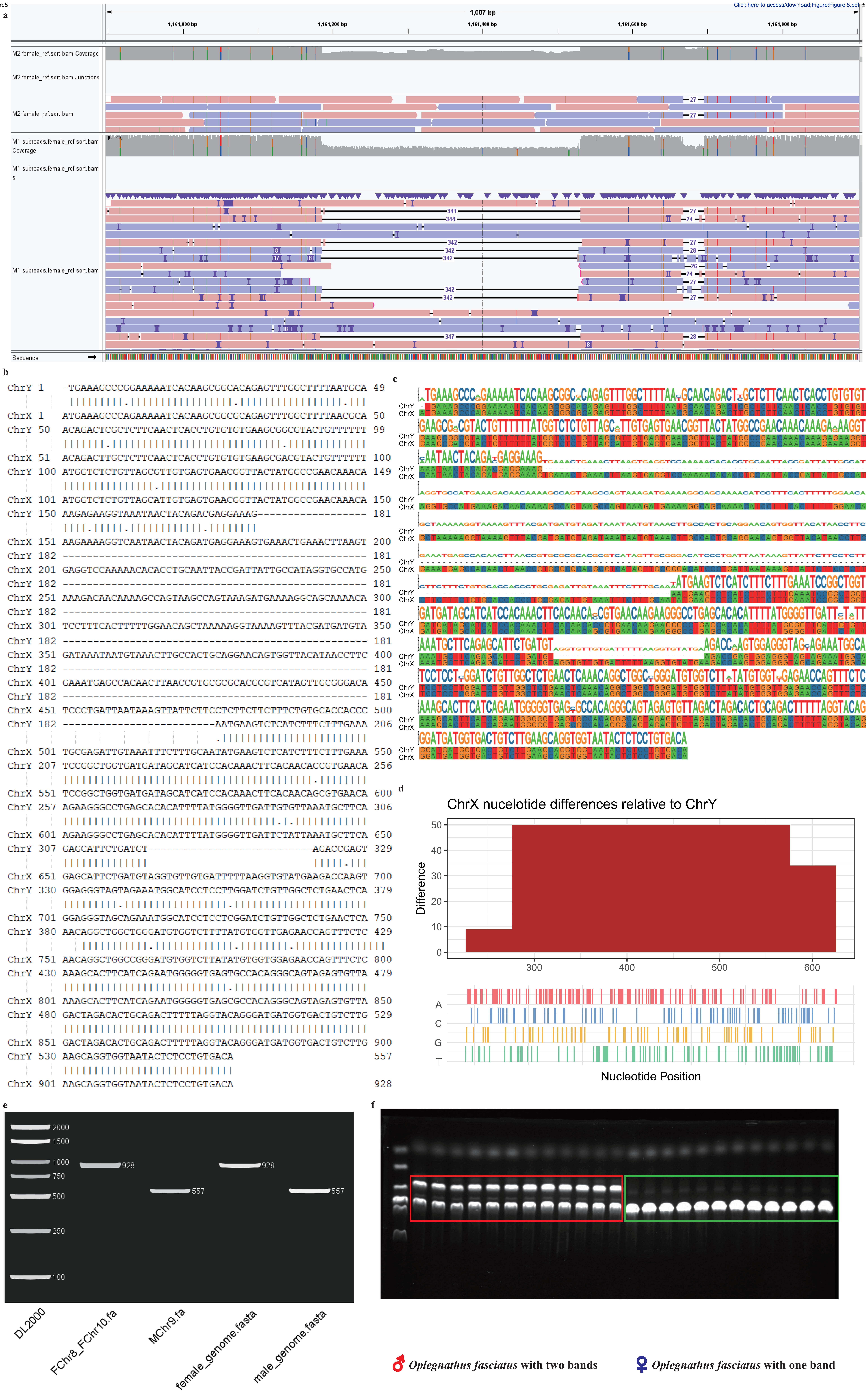

FigureS1 Click here to access/download;Figure;Figure S1.pdf

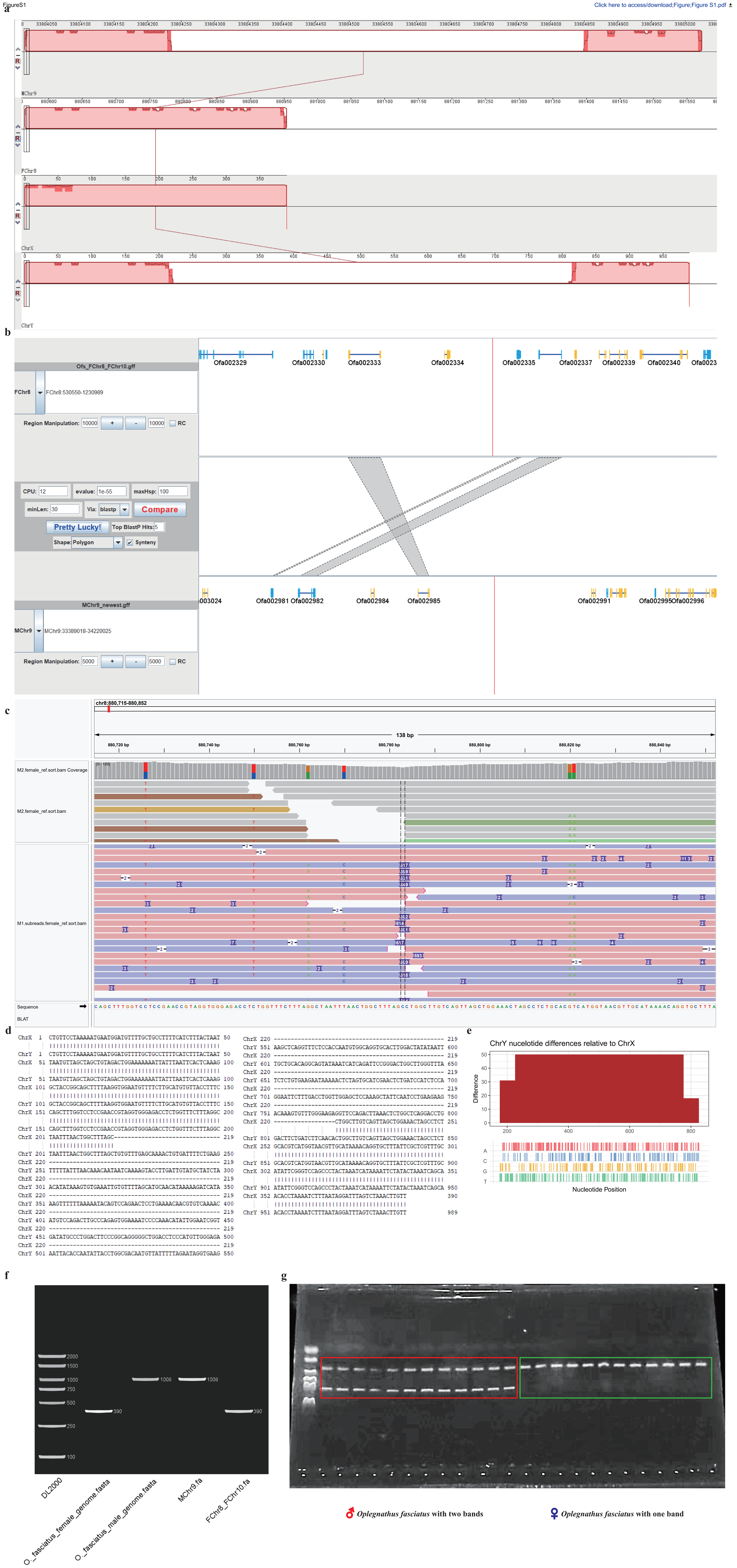

FigureS2

Click here to access/download;Figure;Figure S2.pdf

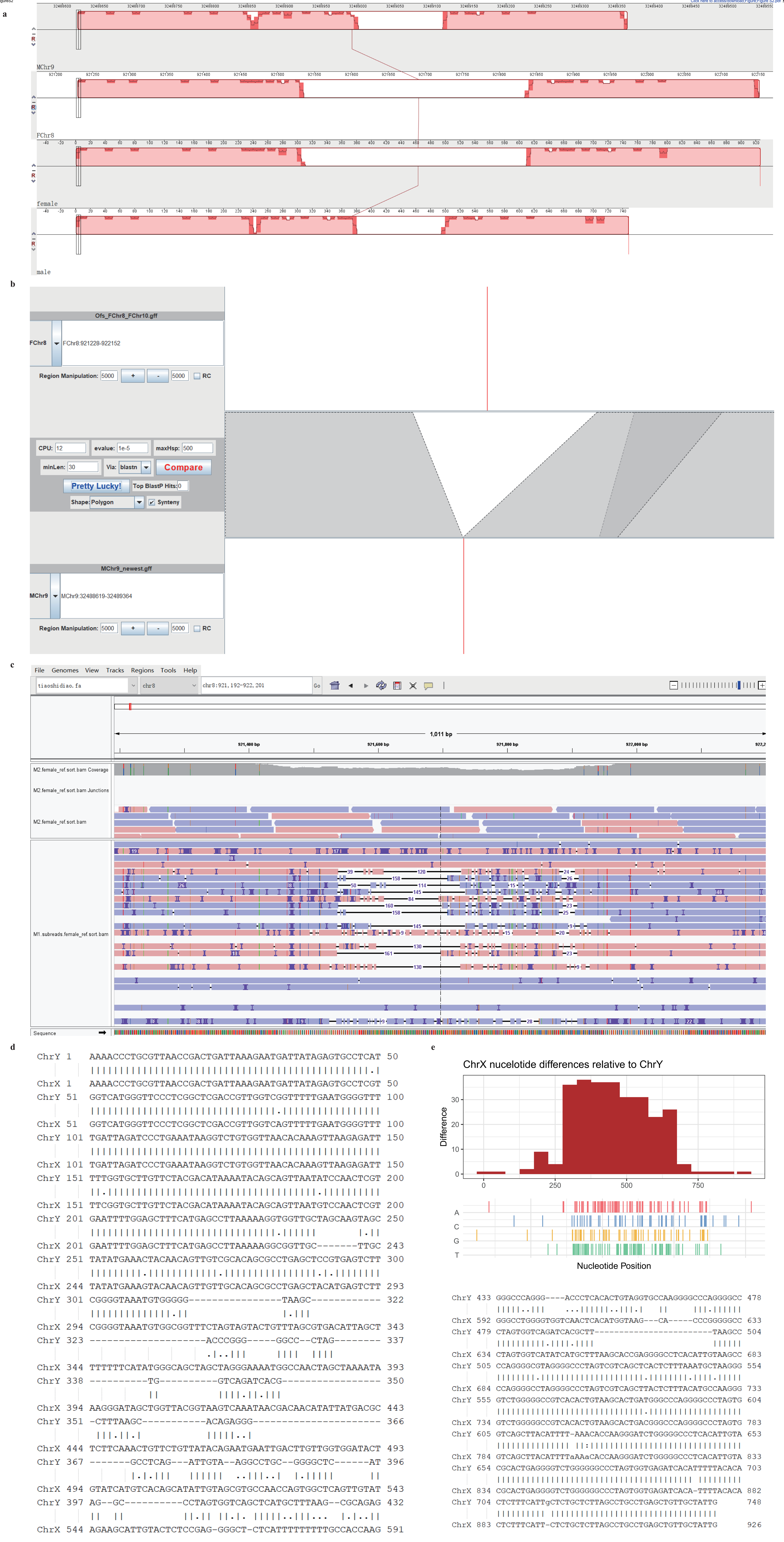

FigureS3
Click here to access/download;Figure;Figure S3.pdf

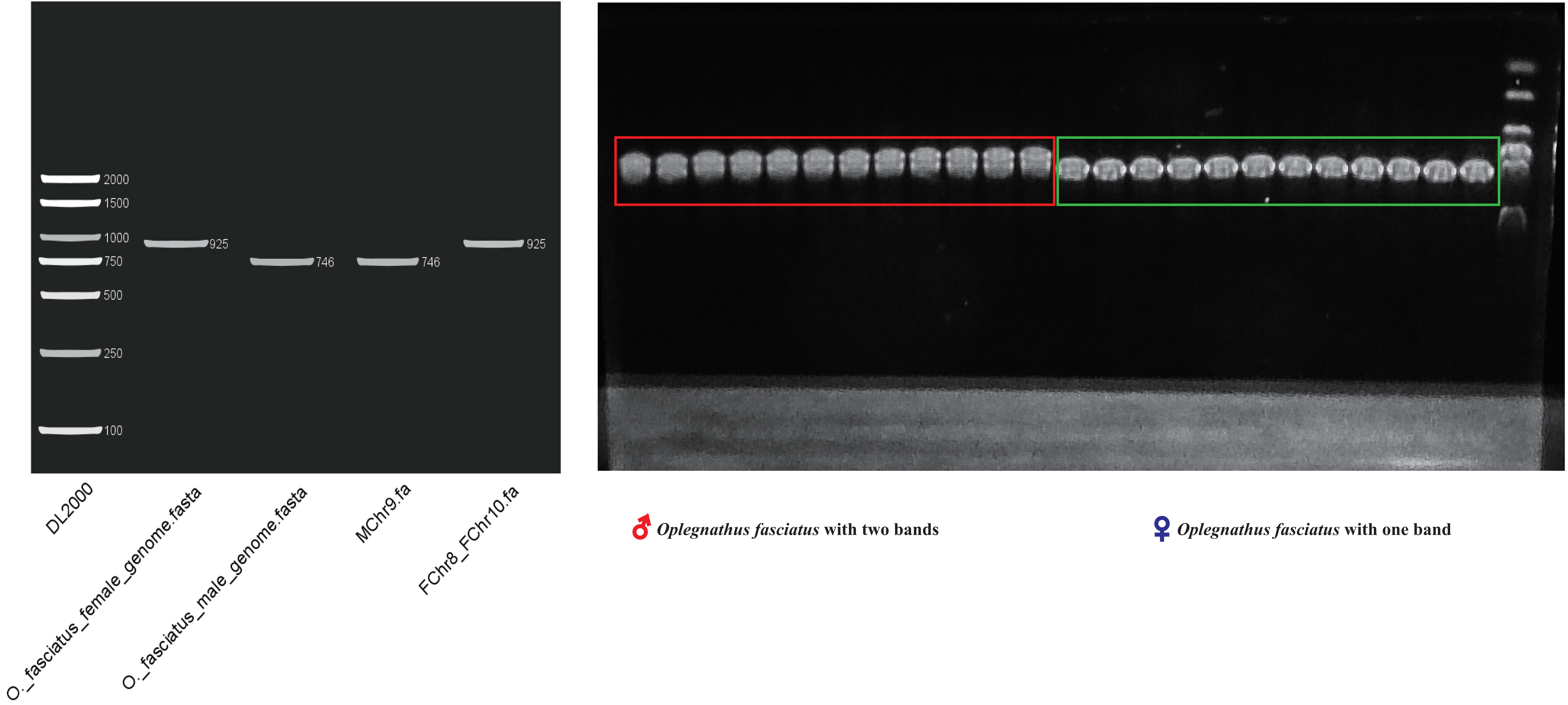

Click here to access/download;Figure;Figure S4.pdf

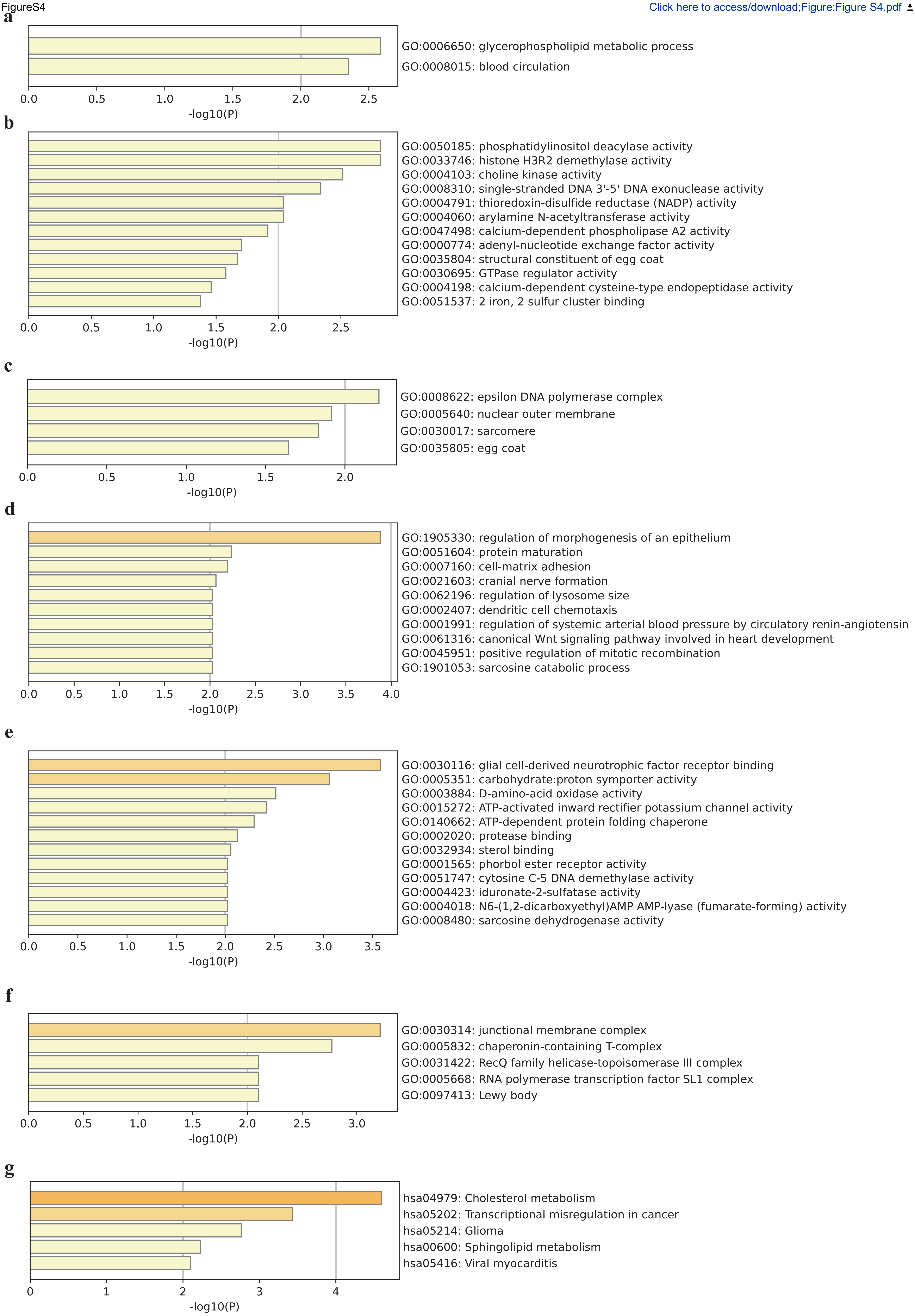

FigureS5
Click here to access/download;Figure;Figure S5.pdf

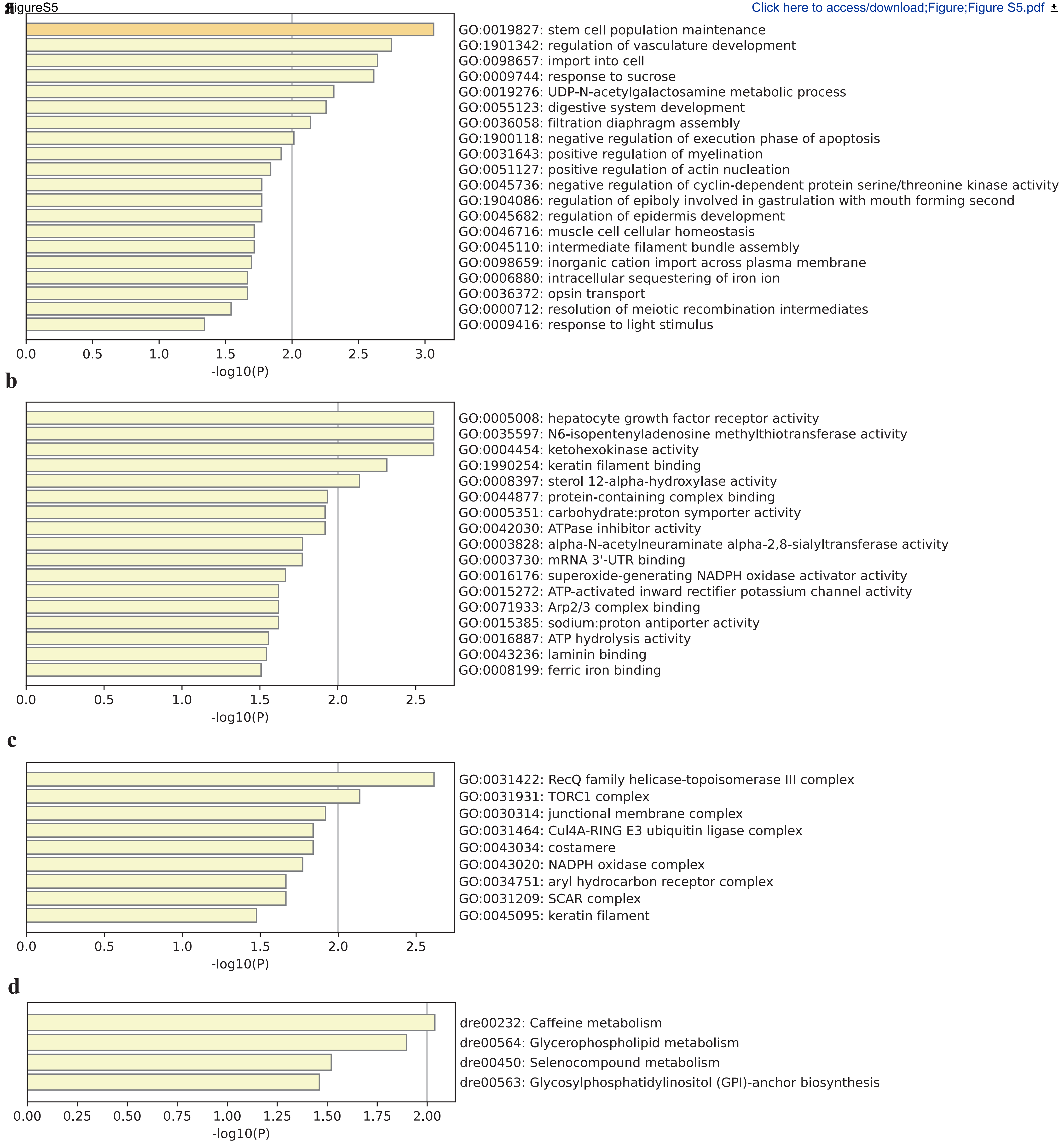

Click here to access/download;Figure;Figure S6.pdf

FigureS6

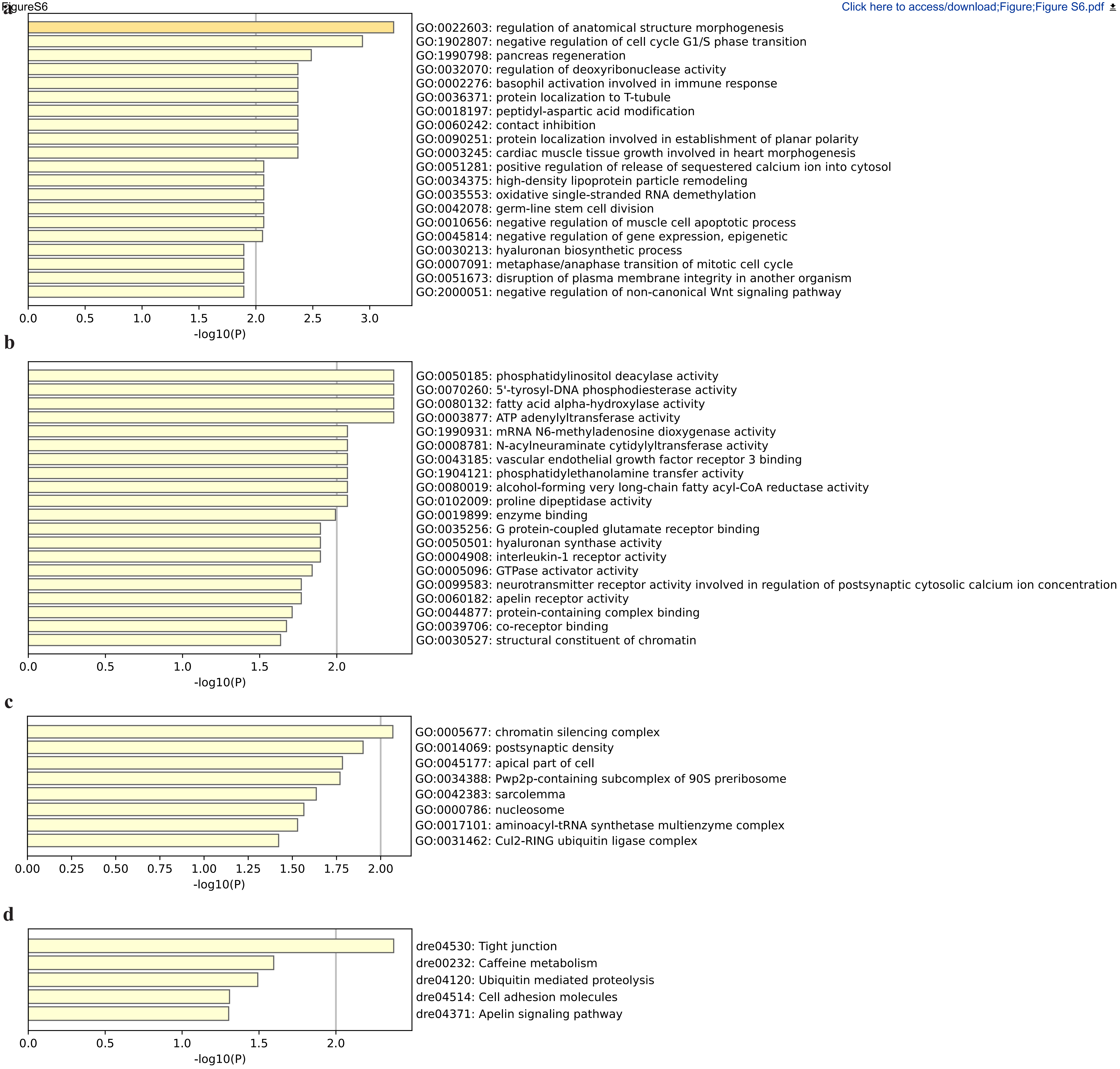

Click here to access/download
Supplementary Material
Supplementary information.docx

Large insertion and deletion data sets based on female genome

Click here to access/download
Supplementary Material
Female_reference_ INS_DEL 100_Set.xlsx

Large insertion and deletion data sets based on male genome

Click here to access/download

Supplementary Material

Male_reference_ INS_DEL 100_Set.xlsx

Large-segment insertion/deletion sites located in the exonic region
based on the female genome

Click here to access/download
Supplementary Material
Female_reference_ INS_DEL
100_ExonSet_annotation.xlsx

Large-segment insertion/deletion sites located in the exonic region
based on the male genome

Click here to access/download
Supplementary Material
Male_reference_ INS_DEL
100_ExonSet_annotation.xlsx

Click here to access/download
Supplementary Material
Female_Male_INSD_EL100_PrimerSet.xlsx
