# Supplementary material for: Innovative approach for high-throughput exploiting sex-specific markers in Japanese parrotfish Oplegnathus fasciatus
Source: Gigascience. 2024 Jul 19;13:giae045. doi: 10.1093/gigascience/giae045 (PMC11258905; doi:10.1093/gigascience/giae045)
Supplement: giae045_Supplemental_Files [file giae045_supplemental_files.zip › Figure S6.pdf]

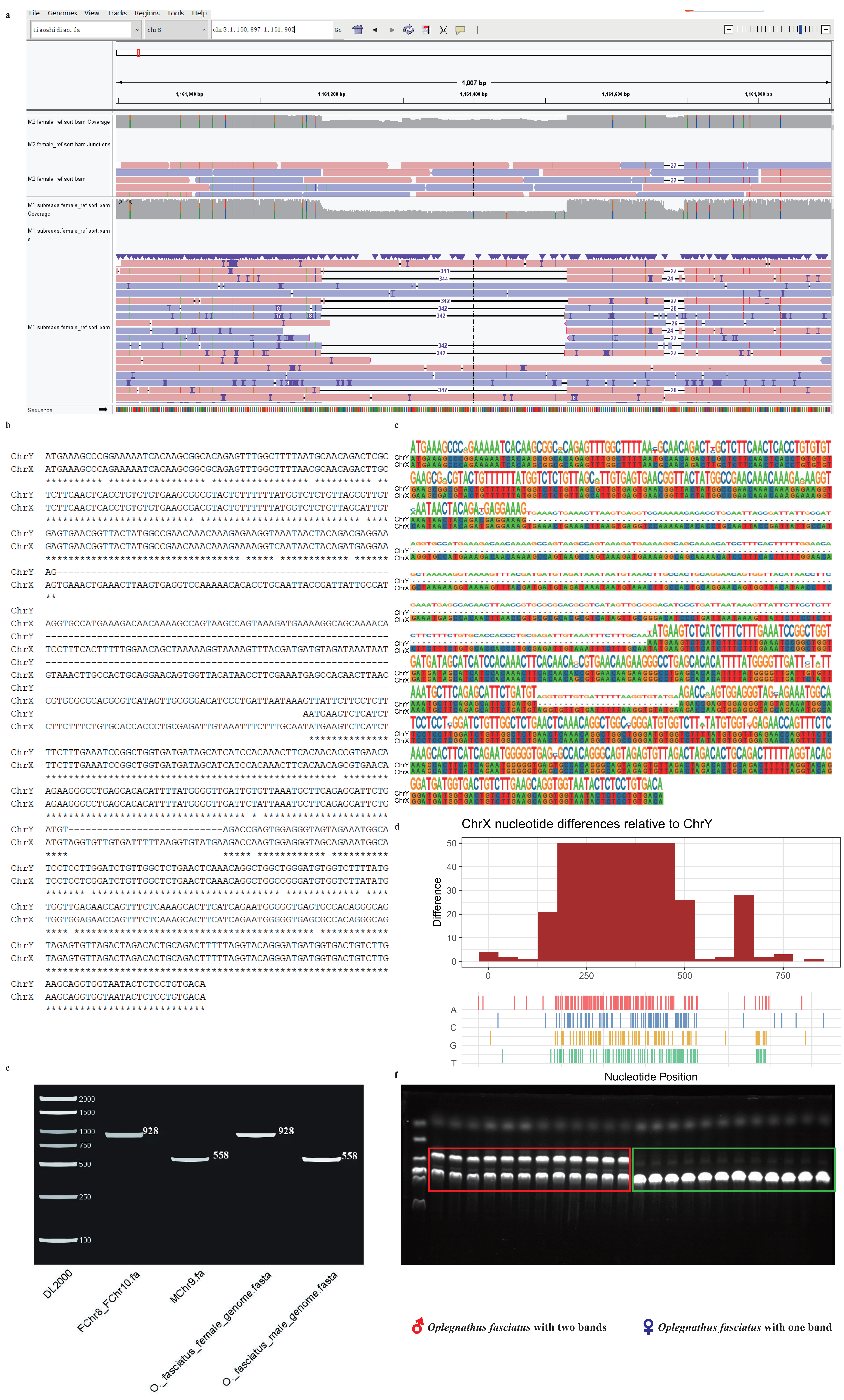
a
File Genomes View Tracks Regions Tools Help
tiaoshidiao.fa
chr8
chr8:1,160,897-1,161,902
Go
1,007 bp
1,161,000 bp
1,161,200 bp
1,161,400 bp
1,161,600 bp
1,161,800 bp
M2.female_ref.sort.bam Coverage
M2.female_ref.sort.bam Junctions
M2.female_ref.sort.bam
M1.subreads.female_ref.sort.bam Coverage
M1.subreads.female_ref.sort.bam
Sequence
b
ChrY ATGAAAGCCCGGAAAAATCACAAGCGGCACAGAGTTTGGCTTTTAATGCAACAGACTCGC
ChrX ATGAAAGCCCAGAAAAATCACAAGCGGCGCAGAGTTTGGCTTTTAACGCAACAGACTTGC
ChrY TCTTCAACTCACCTGTGTGTGAAGCGGCGTACTGTTTTTTATGGTCTCTGTTAGCGTTGT
ChrX TCTTCAACTCACCTGTGTGTGAAGCGACGTACTGTTTTTTATGGTCTCTGTTAGCATTGT
ChrY GAGTGAACGGTTACTATGGCCGAACAAACAAAGAGAAGGTAAATAACTACAGACGAGGAA
ChrX GAGTGAACGGTTACTATGGCCGAACAAACAAAGAGAAAAGGTCAATAACTACAGATGAGGAA
ChrY AG
ChrX AGTGAAACTGAAACTTAAGTGAGGTCCAAAAACACACCTGCAATTACCGATTATTGCCAT
ChrX AGGTGCCATGAAAGACAACAAAAGCCAGTAAGCCAGTAAAGATGAAAAGGCAGCAAAACA
ChrX TCCTTTCACTTTTTGGAACAGCTAAAAAGGTAAAAGTTTACGATGATGTAGATAAATAAT
ChrX GTAAACTTGCCACTGCAGGAACAGTGGTTACATAACCTTCGAAATGAGCCACAACTTAAC
ChrX CGTGCGCGCACGCGTCATAGTTGCGGGACATCCCTGATTAATAAAGTTATTCTTCCTCTT
ChrY AATGAAGTCTCATCT
ChrX CTTCTTTCTGTGCACCACCCTGCGAGATTGTAAATTTCTTTGCAATATGAAGTCTCATCT
ChrY TTCTTTGAAATCCGGCTGGTGATGATAGCATCATCCACAAACTTCACAACACCGTGAACA
ChrX TTCTTTGAAATCCGGCTGGTGATGATAGCATCATCCACAAACTTCACAACAGCGTGAACA
ChrY AGAAGGGCCTGAGCACACATTTTATGGGGTTGATTGTGTTAAATGCTTCAGAGCATTCTG
ChrX AGAAGGGCCTGAGCACACATTTTATGGGGTTGATTCTATTAAATGCTTCAGAGCATTCTG
ChrY ATGT AGACCGAGTGGAGGGTAGTAGAAATGGCA
ChrX ATGTAGGTGTTGTGATTTTTAAGGTGTATGAAGACCAAGTGGAGGGTAGCAGAAATGGCA
ChrY TCCTCCTTGGATCTGTTGGCTCTGAACTCAAACAGGCTGGCTGGGATGTGGTCTTTTATG
ChrX TCCTCCTCGGATCTGTTGGCTCTGAACTCAAACAGGCTGGCCGGGATGTGGTCTTATATG
ChrY TGGTTGAGAACCAGTTTCTCAAAGCACTTCATCAGAATGGGGGTGAGTGCCACAGGGCAG
ChrX TGGTGGAGAACCAGTTTCTCAAAGCACTTCATCAGAATGGGGGTGAGCGCCACAGGGCAG
ChrY TAGAGTGTTAGACTAGACACTGCAGACTTTTTAGGTACAGGGATGATGGTGACTGTCTTG
ChrX TAGAGTGTTAGACTAGACACTGCAGACTTTTTAGGTACAGGGATGATGGTGACTGTCTTG
ChrY AAGCAGGTGGTAATACTCTCCTGTGACA
ChrX AAGCAGGTGGTAATACTCTCCTGTGACA
c
ChrY
ChrX
d
ChrX nucleotide differences relative to ChrY
Difference
Nucleotide Position
A
C
G
T
e
2000
1500
1000
750
500
250
100
928
558
928
558
DL2000
FChr8_FChr10.fa
MChr9.fa
O_fasciatus_female_genome.fasta
O_fasciatus_male_genome.fasta
f
♂ Oplegnathus fasciatus with two bands
♀ Oplegnathus fasciatus with one band
