# Supplementary material for: Innovative approach for high-throughput exploiting sex-specific markers in Japanese parrotfish Oplegnathus fasciatus
Source: Gigascience. 2024 Jul 19;13:giae045. doi: 10.1093/gigascience/giae045 (PMC11258905; doi:10.1093/gigascience/giae045)
Supplement: giae045_Supplemental_Files [file giae045_supplemental_files.zip › Figure S7.pdf]

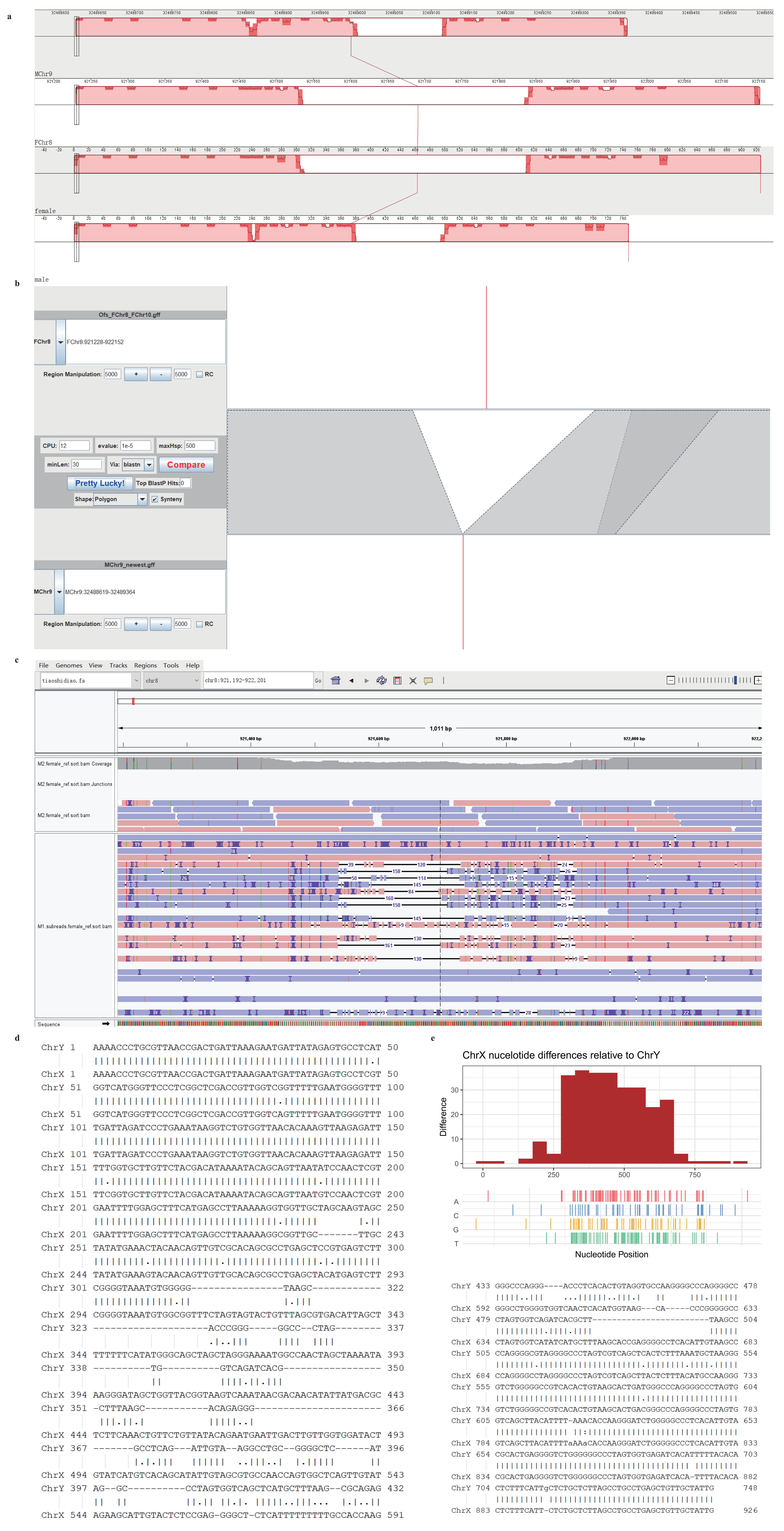
a
MChr9
FChr8
female
male
b
Ofs_FChr8_FChr10.gff
FChr8
FChr8:921228-922152
Region Manipulation:
5000
+
-
5000
RC
CPU:
12
evalue:
1e-5
maxHsp:
500
minLen:
30
Via:
blastn
Compare
Pretty Lucky!
Top BlastP Hits:
0
Shape:
Polygon
Synteny
MChr9_newest.gff
MChr9
MChr9:32488619-32489364
Region Manipulation:
5000
+
-
5000
RC
c
File
Genomes
View
Tracks
Regions
Tools
Help
tiaoshidiao.fa
chr8
chr8:921,192-922,201
Go
1,011 bp
921,400 bp
921,600 bp
921,800 bp
922,000 bp
M2.female_ref.sort.bam Coverage
M2.female_ref.sort.bam Junctions
M2.female_ref.sort.bam
M1.subreads.female_ref.sort.bam
Sequence
d
ChrY 1 AAAACCCTGCGTTAACCGACTGATTAAAGAATGATTATAGAGTGCCTCAT 50
ChrX 1 AAAACCCTGCGTTAACCGACTGATTAAAGAATGATTATAGAGTGCCTCGT 50
ChrY 51 GGTCATGGGTTCCCTCGGCTCGACCGTTGGTCGGTTTTTGAATGGGGTTT 100
ChrX 51 GGTCATGGGTTCCCTCGGCTCGACCGTTGGTCAGTTTTTGAATGGGGTTT 100
ChrY 101 TGATTAGATCCCTGAAATAAGGTCTGTGGTTAACACAAAGTTAAGAGATT 150
ChrX 101 TGATTAGATCCCTGAAATAAGGTCTGTGGTTAACACAAAGTTAAGAGATT 150
ChrY 151 TTTGGTGCTTGTTCTACGACATAAAATACAGCAGTTAATATCCAACTCGT 200
ChrX 151 TTCGGTGCTTGTTCTACGACATAAAATACAGCAGTTAATGTCCAACTCGT 200
ChrY 201 GAATTTTGGAGCTTTCATGAGCCTTAAAAAGGTGGTTGCTAGCAAGTAGC 250
ChrX 201 GAATTTTGGAGCTTTCATGAGCCTTAAAAAGGCGGTTGC-------TTGC 243
ChrY 251 TATATGAAACTACAACAGTTGTCGCACAGCGCCTGAGCTCCGTGAGTCTT 300
ChrX 244 TATATGAAAGTACAACAGTTGTTGCACAGCGCCTGAGCTACATGAGTCTT 293
ChrY 301 CGGGGTAAATGTGGGGG-----------------TAAGC------------ 322
ChrX 294 CGGGGTAAATGTGGCGGTTTCTAGTAGTACTGTTTAGCGTGACATTAGCT 343
ChrY 323 --------------------ACCCGGG-----GGCC--CTAG--------- 337
ChrX 344 TTTTTTCATATGGGCAGCTAGCTAGGGAAAATGGCCAACTAGCTAAAATA 393
ChrY 338 ----------TG----------GTCAGATCACG------------------ 350
ChrX 394 AAGGGATAGCTGGTTACGGTAAGTCAAATAACGACAACATATTATGACGC 443
ChrY 351 -CTTTAAGC-----------ACAGAGGG---------------------- 366
ChrX 444 TCTTCAAACTGTTCTGTTATACAGAATGAATTGACTTGTTGGTGGATACT 493
ChrY 367 -------GCCTCAG---ATTGTA--AGGCCTGC--GGGGCTC------AT 396
ChrX 494 GTATCATGTCACAGCATATTGTAGCGTGCCAACCAGTGGCTCAGTTGTAT 543
ChrY 397 AG--GC----------CCTAGTGGTCAGCTCATGCTTTAAG--CGCAGAG 432
ChrX 544 AGAAGCATTGTACTCTCCGAG-GGGCT-CTCATTTTTTTTTGCCACCAAG 591
e
ChrX nucelotide differences relative to ChrY
Difference
0
10
20
30
0
250
500
750
A
C
G
T
Nucleotide Position
ChrY 433 GGGCCCAGGG----ACCCTCACACTGTAGGTGCCAAGGGGCCCAGGGGCC 478
ChrX 592 GGGCCTGGGGTGGTCAACTCACATGGTAAG---CA-----CCCGGGGCC 633
ChrY 479 CTAGTGGTCAGATCACGCTT------------------------TAAGCC 504
ChrX 634 CTAGTGGTCATATCATGCTTTAAGCACCGAGGGGCCTCACATTGTAAGCC 683
ChrY 505 CCAGGGGCGTAGGGGCCCTAGTCGTCAGCTCACTCTTTAAATGCTAAGGG 554
ChrX 684 CCAGGGGCCTAGGGGCCCTAGTCGTCAGCTTACTCTTTACATGCCAAGGG 733
ChrY 555 GTCTGGGGGCCGTCACACTGTAAGCACTGATGGGCCCAGGGGCCCTAGTG 604
ChrX 734 GTCTGGGGGCCGTCACACTGTAAGCACTGACGGGCCCAGGGGCCCTAGTG 783
ChrY 605 GTCAGCTTACATTTT-AAACACCAAGGGATCTGGGGGCCCTCACATTGTA 653
ChrX 784 GTCAGCTTACATTTTaAAaCACCAAGGGATCTGGGGGCCCTCACATTGTA 833
ChrY 654 CGCACTGAGGGGTCTGGGGGGCCCTAGTGGTGAGATCACATTTTTACACA 703
ChrX 834 CGCACTGAGGGGTCTGGGGGGCCCTAGTGGTGAGATCACA-TTTTACACA 882
ChrY 704 CTCTTTCATTgCTCTGCTCTTAGCCTGCCTGAGCTGTTGCTATTG 748
ChrX 883 CTCTTTCATT-CTCTGCTCTTAGCCTGCCTGAGCTGTTGCTATTG 926
