# Supplementary material for: Innovative approach for high-throughput exploiting sex-specific markers in Japanese parrotfish Oplegnathus fasciatus
Source: Gigascience. 2024 Jul 19;13:giae045. doi: 10.1093/gigascience/giae045 (PMC11258905; doi:10.1093/gigascience/giae045)
Supplement: giae045_Supplemental_Files [file giae045_supplemental_files.zip › Figure S8.pdf]

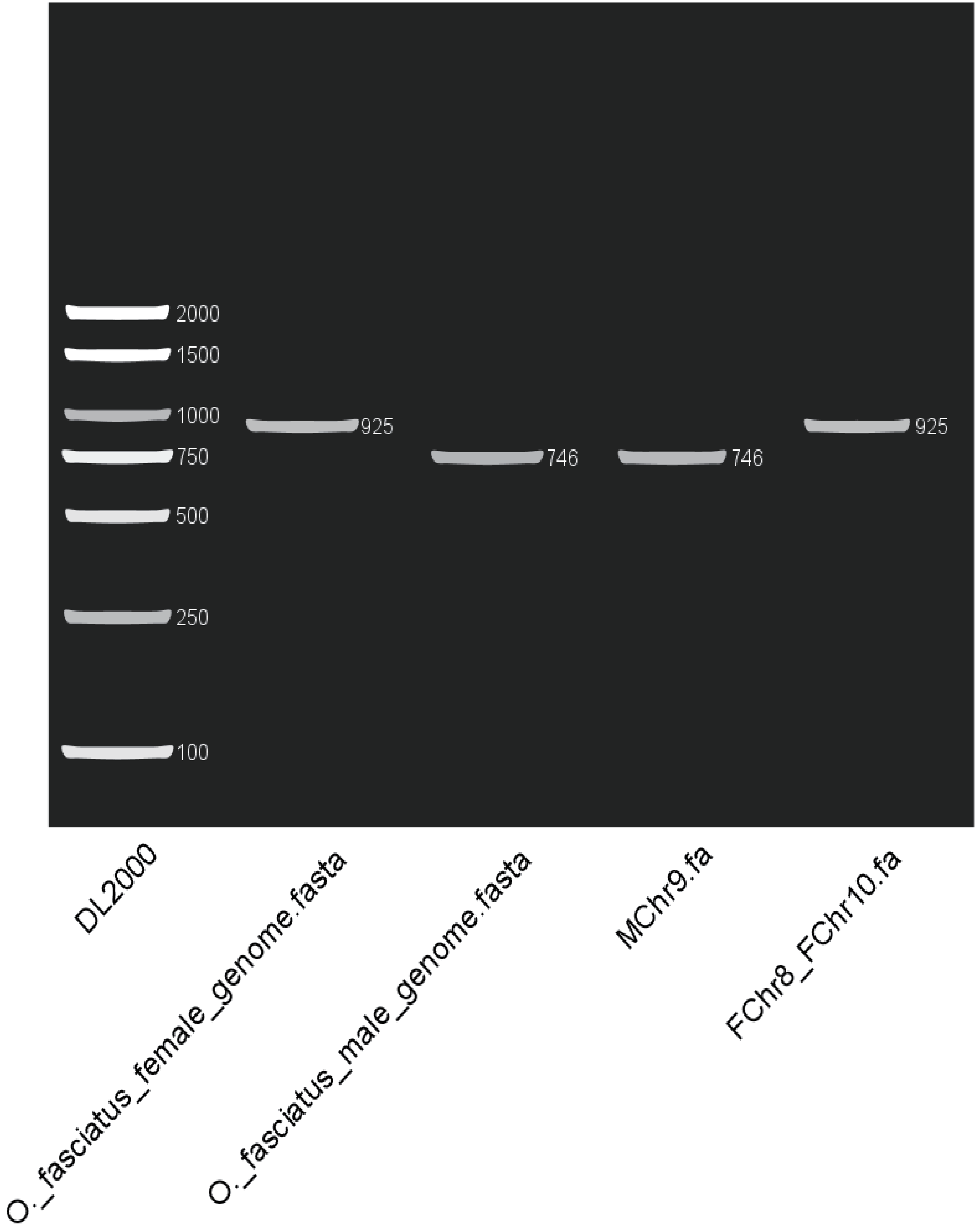

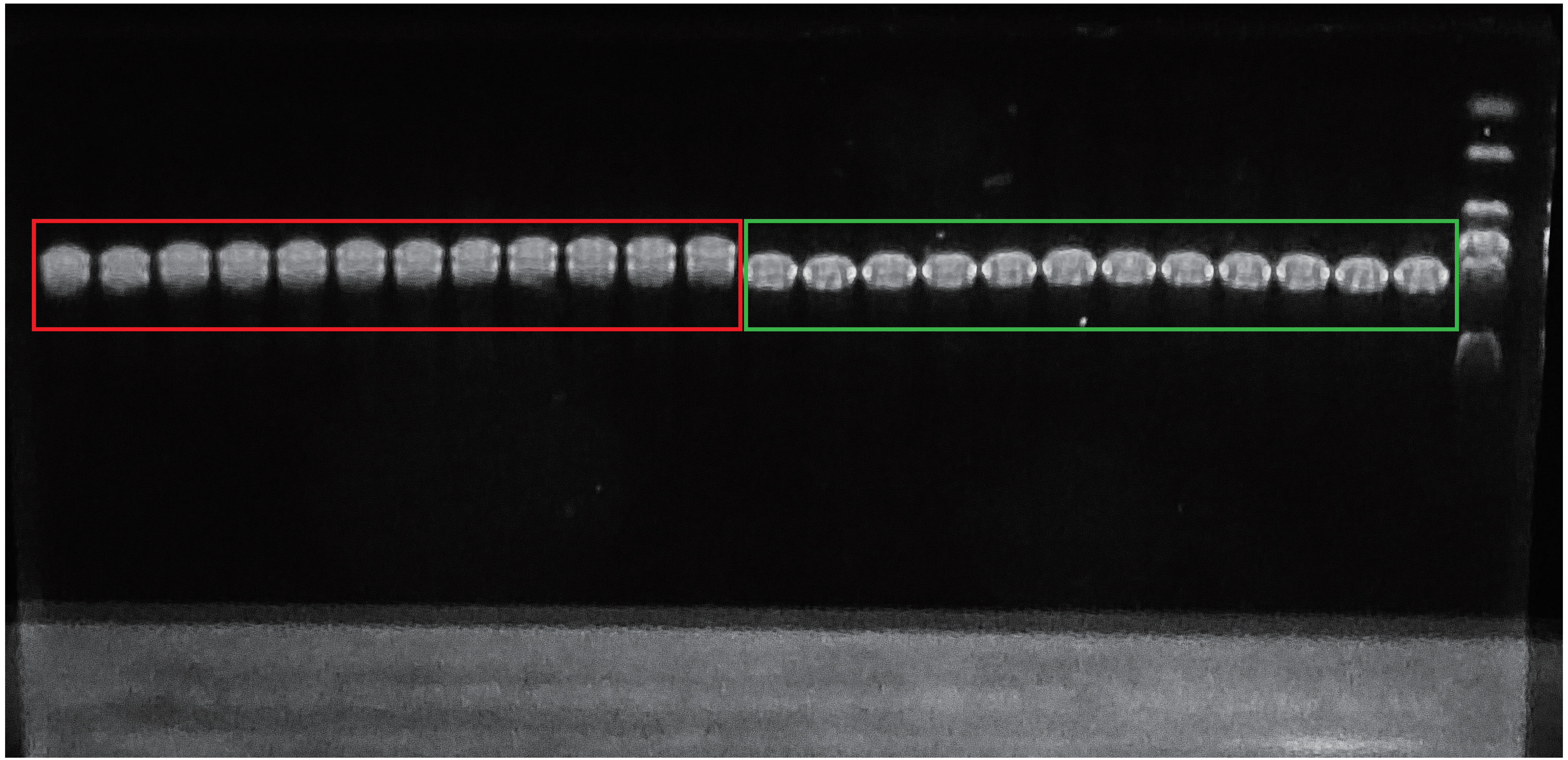

♂ *Oplegnathus fasciatus* with two bands

♀ *Oplegnathus fasciatus* with one band
