# Supplementary material for: Innovative approach for high-throughput exploiting sex-specific markers in Japanese parrotfish Oplegnathus fasciatus
Source: Gigascience. 2024 Jul 19;13:giae045. doi: 10.1093/gigascience/giae045 (PMC11258905; doi:10.1093/gigascience/giae045)
Supplement: giae045_Supplemental_Files [file giae045_supplemental_files.zip › Supplementary information.docx]

**Supplementary material**

**Innovative approach for high-throughput exploiting sex-specific markers in japanese parrotfish *Oplegnathus fasciatus***

Yongshuang Xiao^1,2,3,4†^, Zhizhong Xiao^1,2,3,4†^, Yuting Ma^1,2,3,4†^, Haixia Zhao^1,2,3,4†^, Yanduo Wu^1,2,3,4†^, Jinwei Huang^1,2,3,4^, Pingrui Xu^1,2,3,4^, Lin Liu^5*^, Jing Liu^1*^, Jun Li^1,2,3,4*^

^1^Center for Ocean Mega-Science, Institute of Oceanology, Chinese Academy of Sciences, Qingdao, China，^2^CAS and Shandong Province Key Laboratory of Experimental Marine Biology, Institute of Oceanology, Chinese Academy of Sciences, Qingdao, China，^3^Key Laboratory of Breeding Biotechnology and Sustainable Aquaculture, Chinese Academy of Sciences, Qingdao, China，^4^Laboratory for Marine Biology and Biotechnology, Qingdao National Laboratory for Marine Science and Technology, Qingdao, China, ^5^Wuhan Frasergen Bioinformatics Co., Ltd. East Lake High-Tech Zone, Wuhan, China.

^†^ Co-first authors

∗Correspondence address: Lin Liu, Wuhan Frasergen Bioinformatics Co., Ltd. East Lake High-Tech Zone, Wuhan, 430075, China; Tel: +86-053282896729; E-mail: liulin199306@163.com; Jing Liu, Institute of Oceanology, Chinese Academy of Sciences, 7 Nanhai Road, Qingdao, 266071, China; Tel: +86-053282898790; E-mail: jliu@qdio.ac.cn; Jun Li, Institute of Oceanology, Chinese Academy of Sciences, 7 Nanhai Road, Qingdao, 266071, China; Tel: +86-053282898718; E-mail: junli@qdio.ac.cn.

†Contributed equally to this work.

Yongshuang Xiao, ORCID: 0000-0002-1979-4555

Zhizhong Xiao, ORCID: 0000-0003-2403-1381

Yuting Ma, ORCID: 0000-0002-7158-9364

Haixia Zhao, ORCID: 0009-0003-8305-6009

Yanduo Wu, ORCID: 0000-0003-0060-1685

Jinwei Huang, ORCID: 0009-0003-5238-8318

Lin Liu, ORCID: 0009-0001-7315-2529

Jing Liu, ORCID: 0000-0001-8868-0386

Jun Li, ORCID: 0009-0005-2415-1895

Supplementary Figure numbers: 9


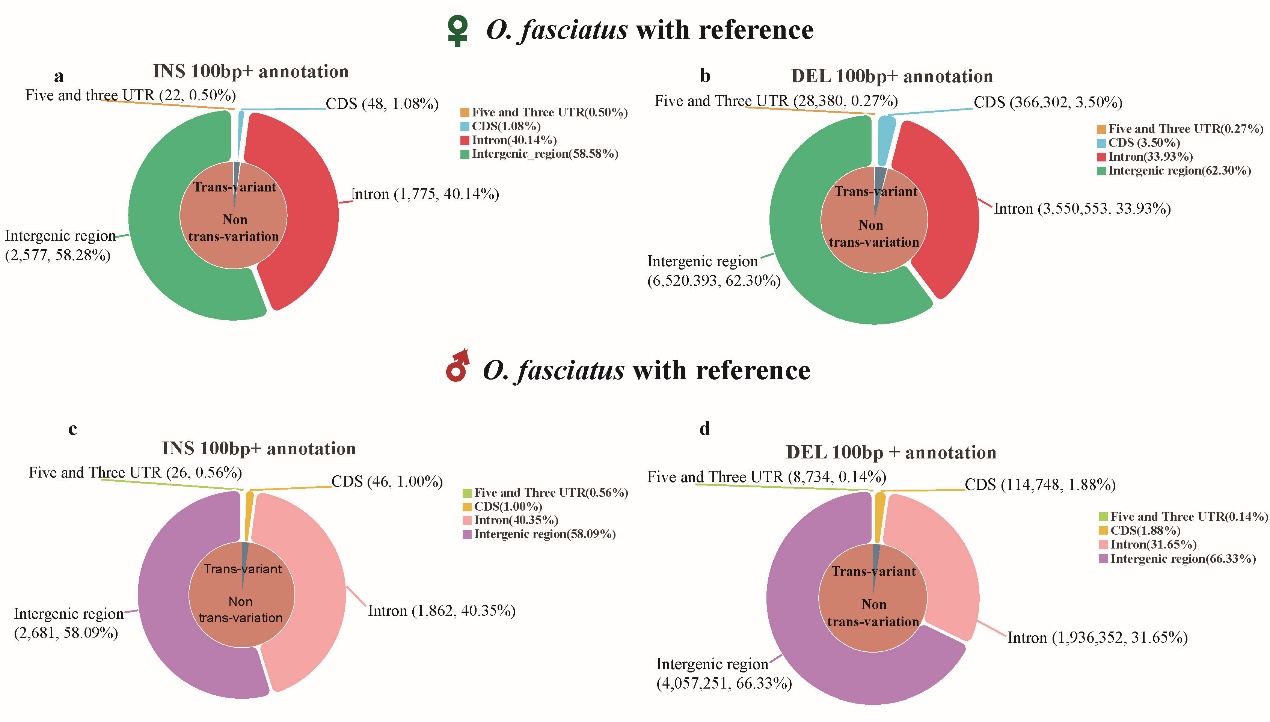


**Figure S1:** Frequency of insertion and deletions (＞100bp) in functional regions of the *O. fasciatus* genome

(a) Number and frequency of insertion events using the female *O. fasciatus* genome as reference. (b) Distribution statistics of deletion variant length using the female *O. fasciatus* genome as a reference. (c) Number and frequency of insertion variant loci utilizing the male *O. fasciatus* genome as a point of reference. (d) Distribution statistics of deletion variant loci using the male *O. fasciatus* genome as a reference.

Note: Non trans-variation represented the intron, and intergenic regions. Trans-variation represented the UTR, CDS regions. The frequency of insertions (a, c) was quantified based on the number of events, while deletions (b, d) were characterized by their length in nucleotides.


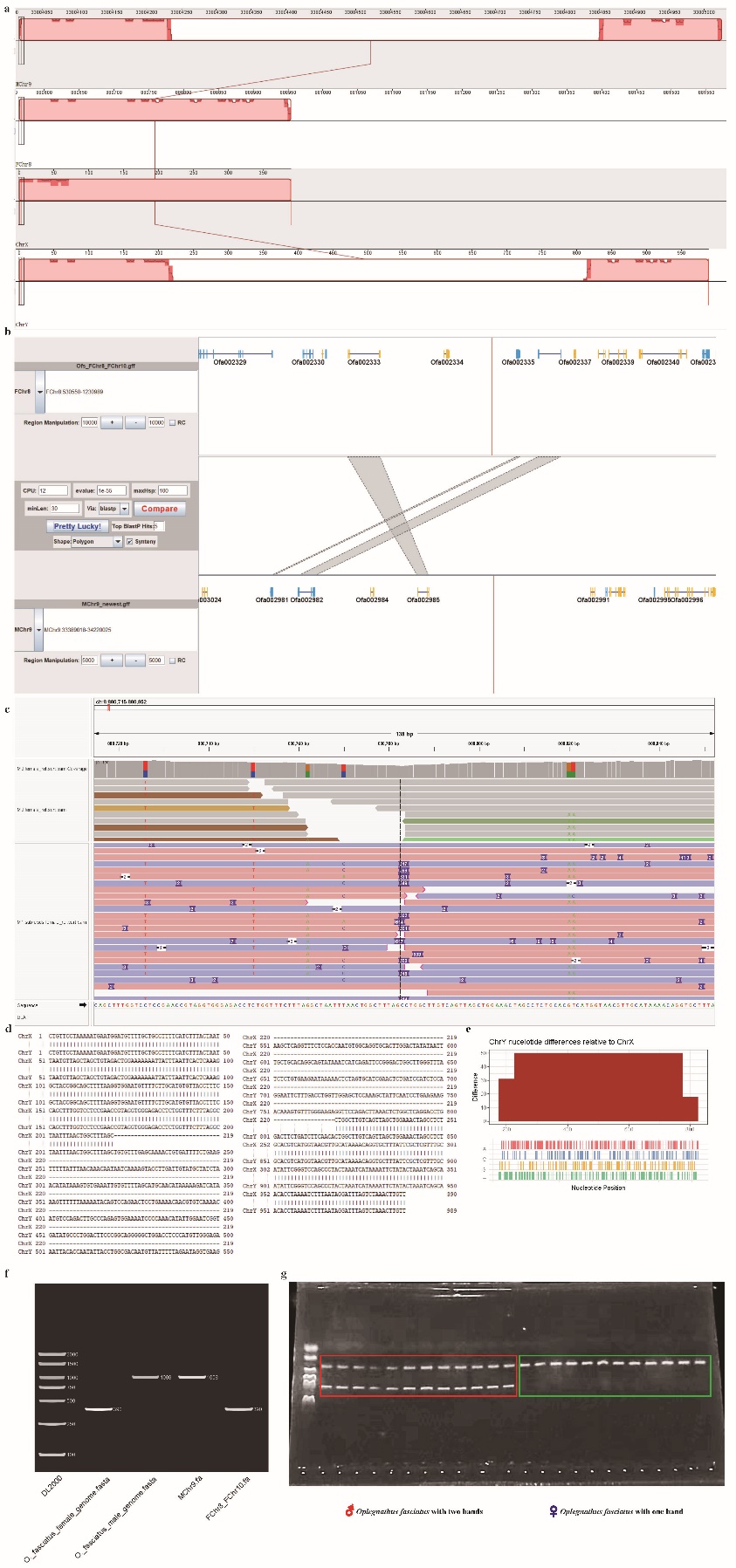


**Figure S2. Representative type of genetic sex marker (insertion deletion variant sites) located in the intergenic region of the *O. fasciatus* genome.**

(a) Information on the location of insertion/deletion sequence differences in male and female genetic sex marker and their differences in comparison with male and female genomic sequences. (b) Location of male and female sex markers in the male and female genomes. (c) Illumina and CLR clean data of Bam comparison to genome for insertion/deletion site validity detection. (d) Nucleotide sequence comparison of marker for female and male genetic sex identification. (e) Visualization of regions of nucleotide sequence heterogeneity in markers for female and male genetic sex identification. (f) Electronically simulated amplification results of target marker primer (e-PCR). (g) Results of PCR amplification and agarose gel electrophoresis detection of target marker (two bands for males and one band for females).


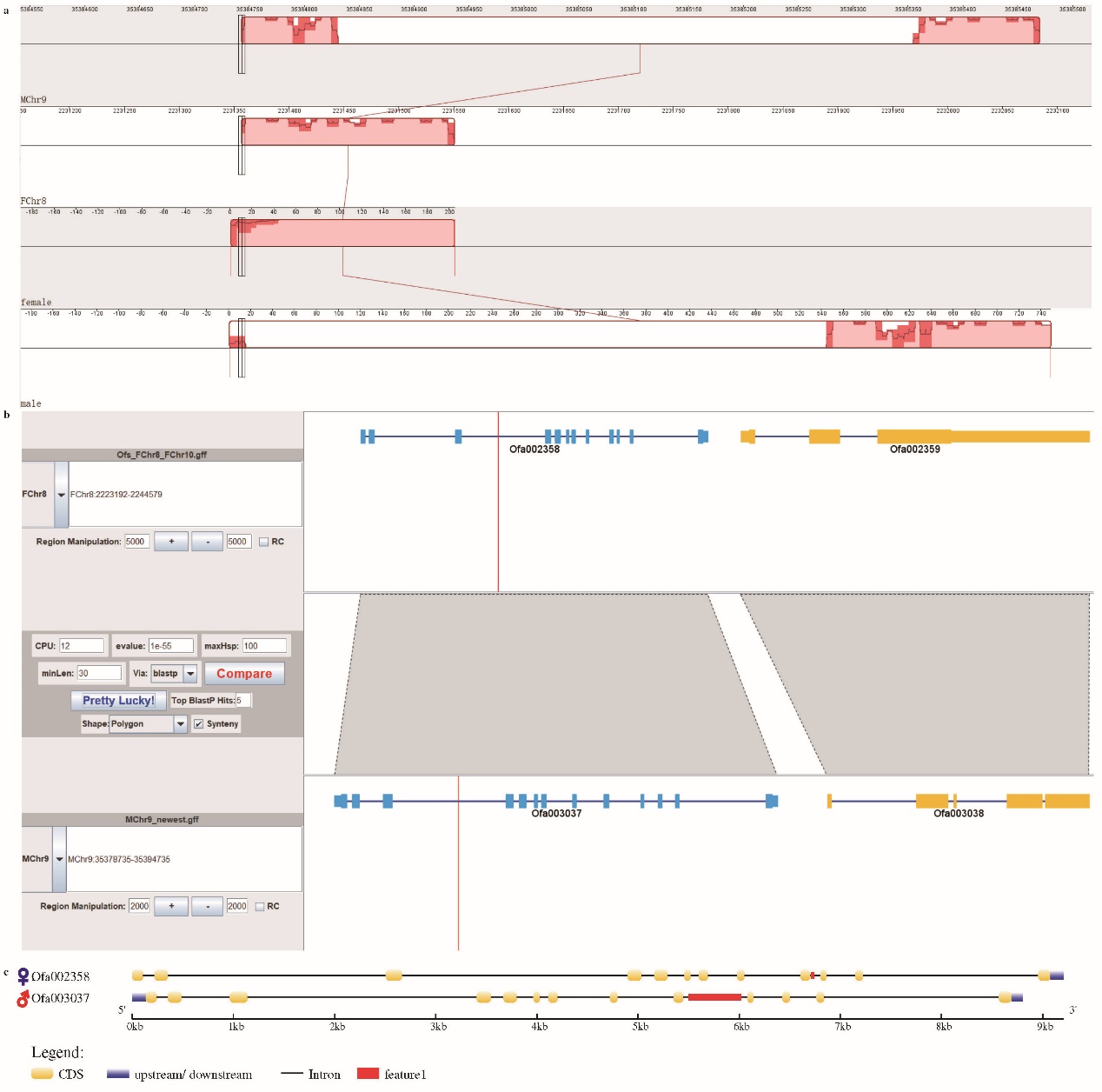


**Figure** **S3:** Representative genetic sex marker (insertion and deletion variant sites) located in the intron region of the *nuf2* gene in *O. fasciatus*.

(a) Collinearity relationships between male and female genetic sex markers and their association with the male and female genomes. (b) Location of male and female sex markers in the male and female genomes. (c) Unit composition of gene functional regions, occurrence of insertions and deletions of genetic sex markers, and sequence length patterns.


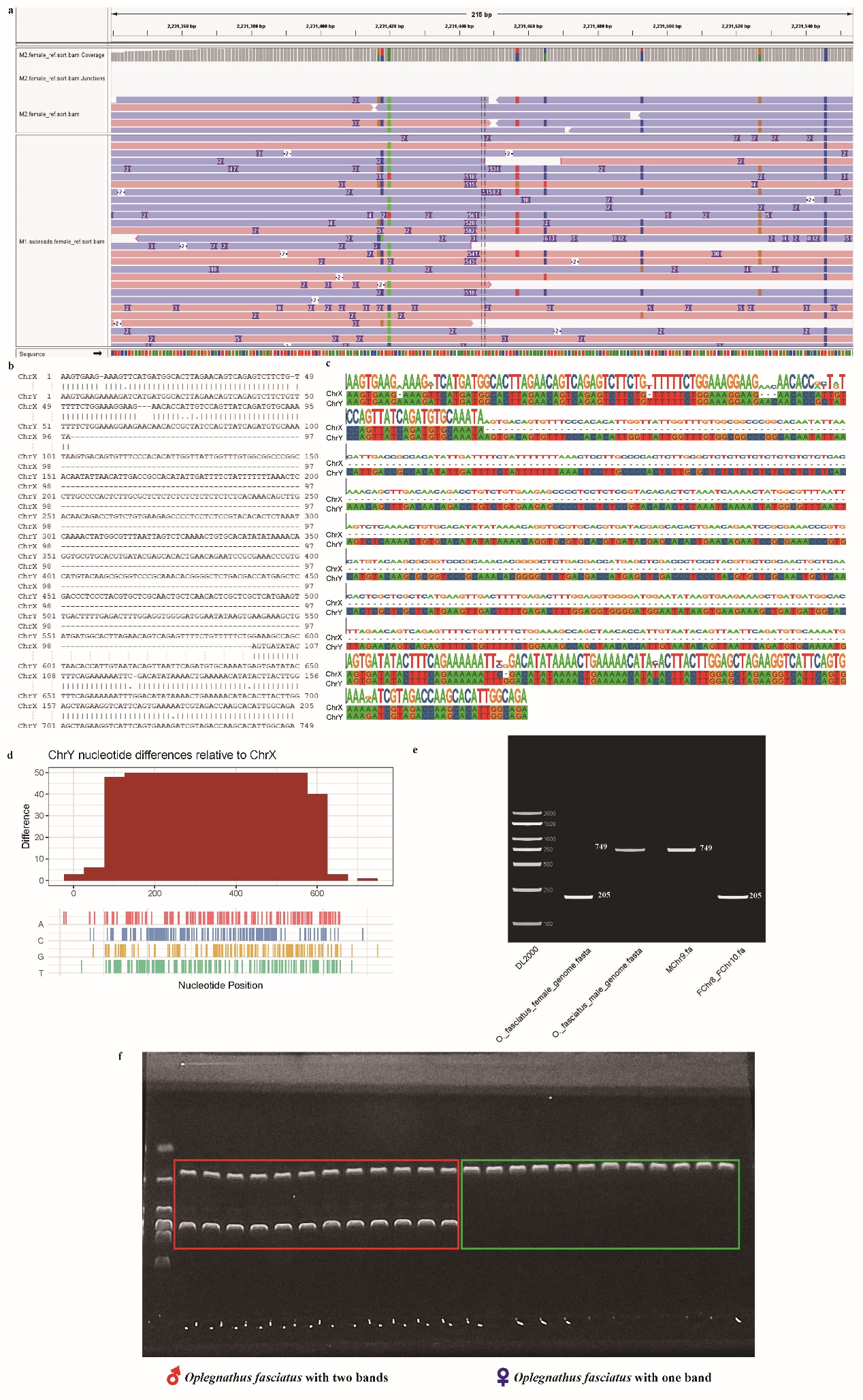


**Figure S4:** Validity testing of genetic markers located in the intronic region of the *nuf2* gene.

(a) Illumina and CLR clean data of Bam comparison to the genome for insertion/deletion site validity detection. (b) Nucleotide sequence comparison of markers for female and male genetic sex identification. (c) Comparison of genetic sex marker sequences based on the global alignment standard. (d) Visualization regions of nucleotide sequence heterogeneity in markers for female and male genetic sex identification. (e) Electronically simulated amplification results of the target marker primer (e-PCR). (f) Results of PCR amplification and agarose gel electrophoresis detection of target markers (two bands for males and one band for females).


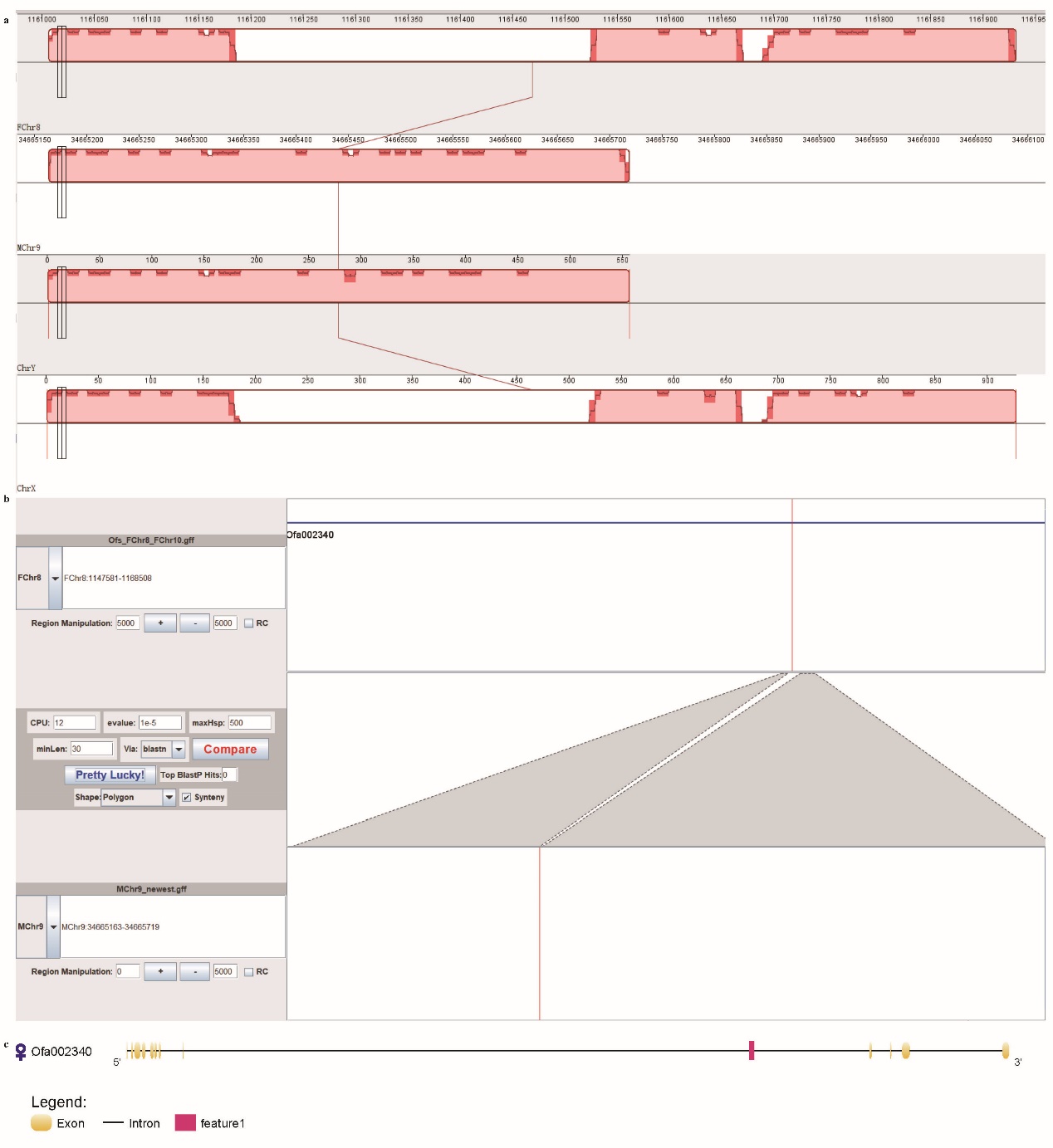


**Figure S5:** Representative male deletion genetic sex marker (insertion deletion variant sites).

(a) Collinearity relationships between male and female genetic sex markers and their association with the male and female genomes. (b) Location of male and female sex markers in the male and female genomes. (c) Unit composition of gene functional regions and occurrence of male deletion genetic sex markers.


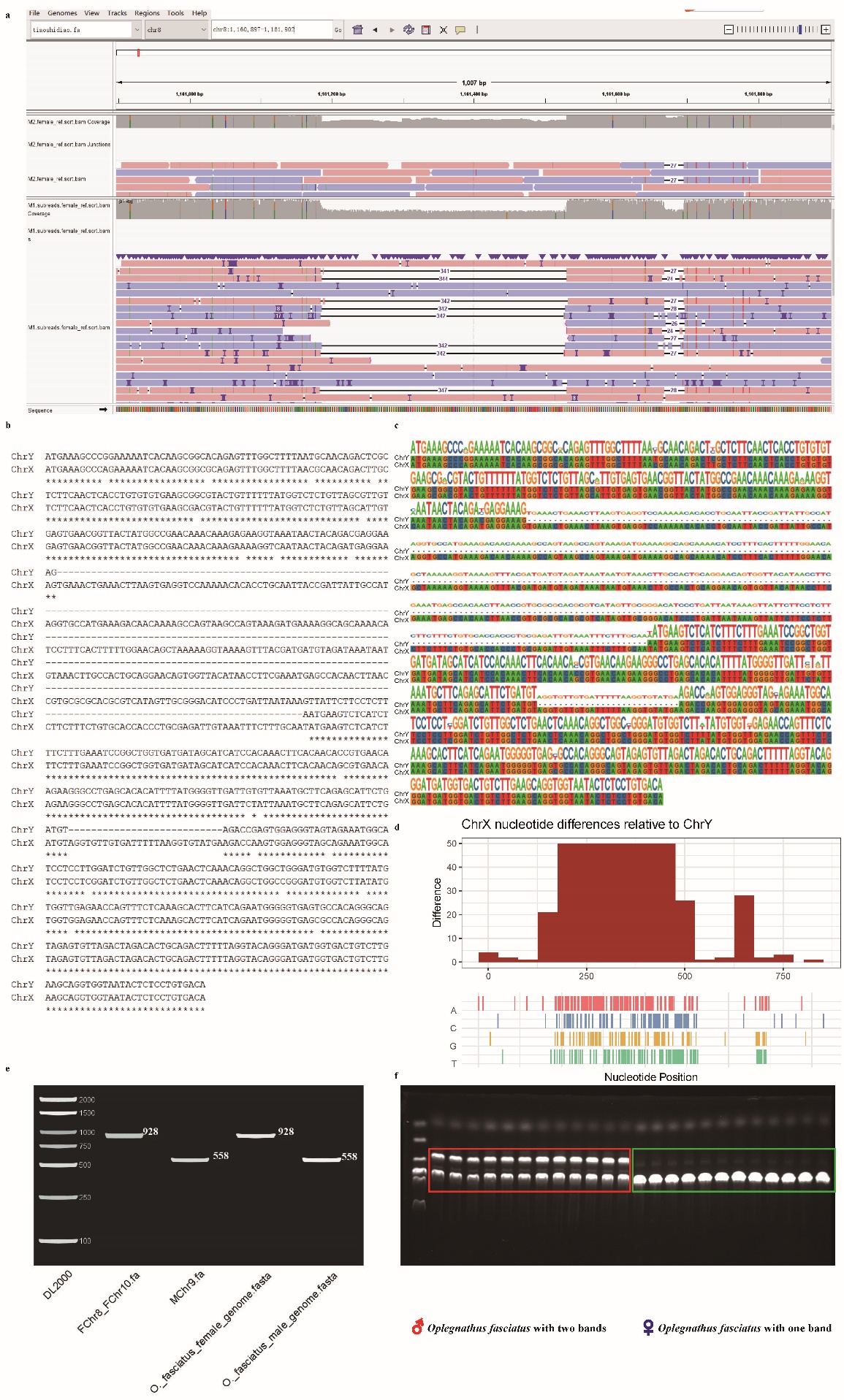


**Figure S6:** Validity testing of the male deletion genetic markers.

(a) Illumina and CLR clean data of Bam comparison to the genome for insertion/deletion site validity detection. (b) Nucleotide sequence comparison of markers for female and male genetic sex identification. (c) Comparison of genetic sex marker sequences based on the global alignment standard. (d) Visualization regions of nucleotide sequence heterogeneity in markers for female and male genetic sex identification. (e) Electronically simulated amplification results of the target marker primer (e-PCR). (f) Results of PCR amplification and agarose gel electrophoresis detection of target markers (two bands for males and one band for females).


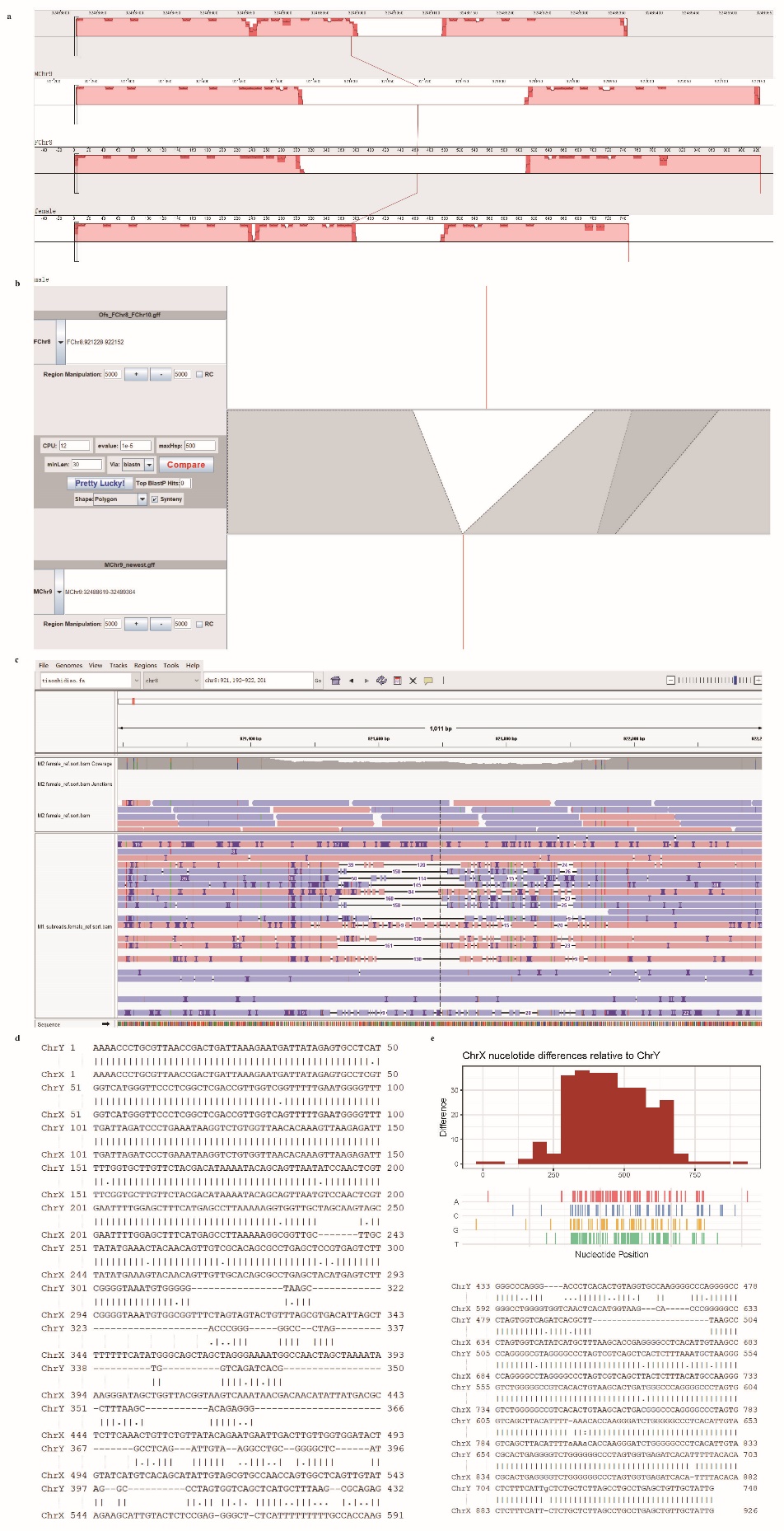


**Figure S7. Representative type of genetic sex marker (insertion deletion variant sites)** **with the male deletion of the *O. fasciatus* genome.**

(a) Co-linearity relationships between male and female genetic sex markers and their association with the male and female genomes. (b) Location of male and female sex markers in the male and female genomes. (c) Illumina and CLR clean data of Bam comparison to genome for insertion/deletion site validity detection. (d) Nucleotide sequence comparison of marker for female and male genetic sex identification. (e) Visualization of regions of nucleotide sequence heterogeneity in markers for female and male genetic sex identification.


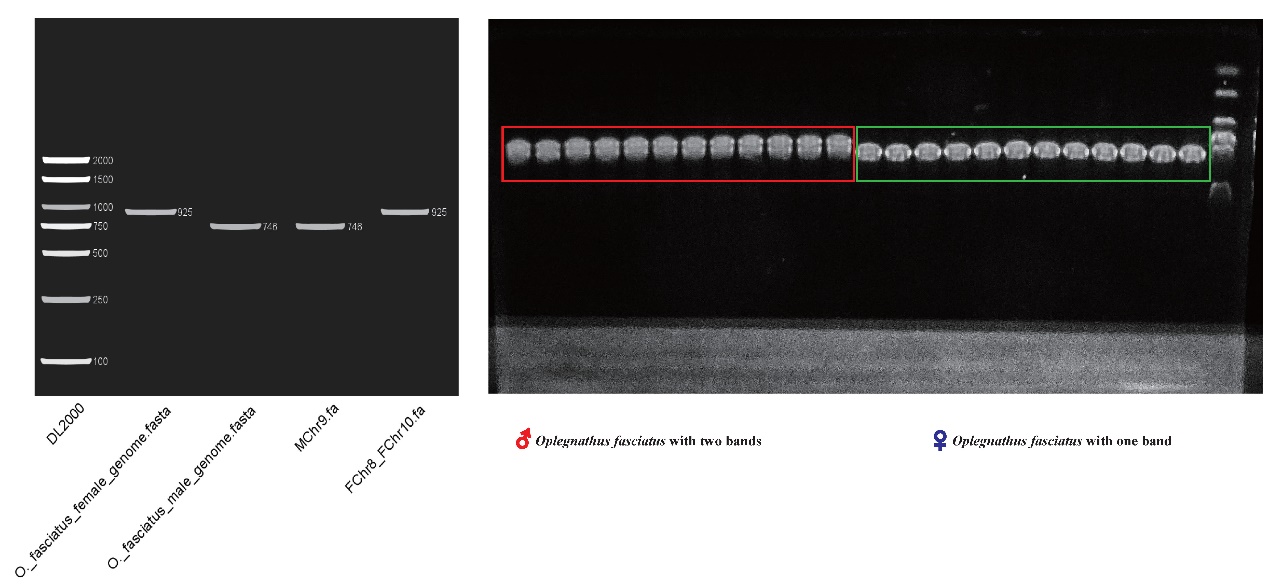


**Figure S8.** Electronically simulated amplification results of target marker primer (e-PCR) (Left). Results of PCR amplification and agarose gel electrophoresis detection of target marker (two bands for males and one band for females) (Right).


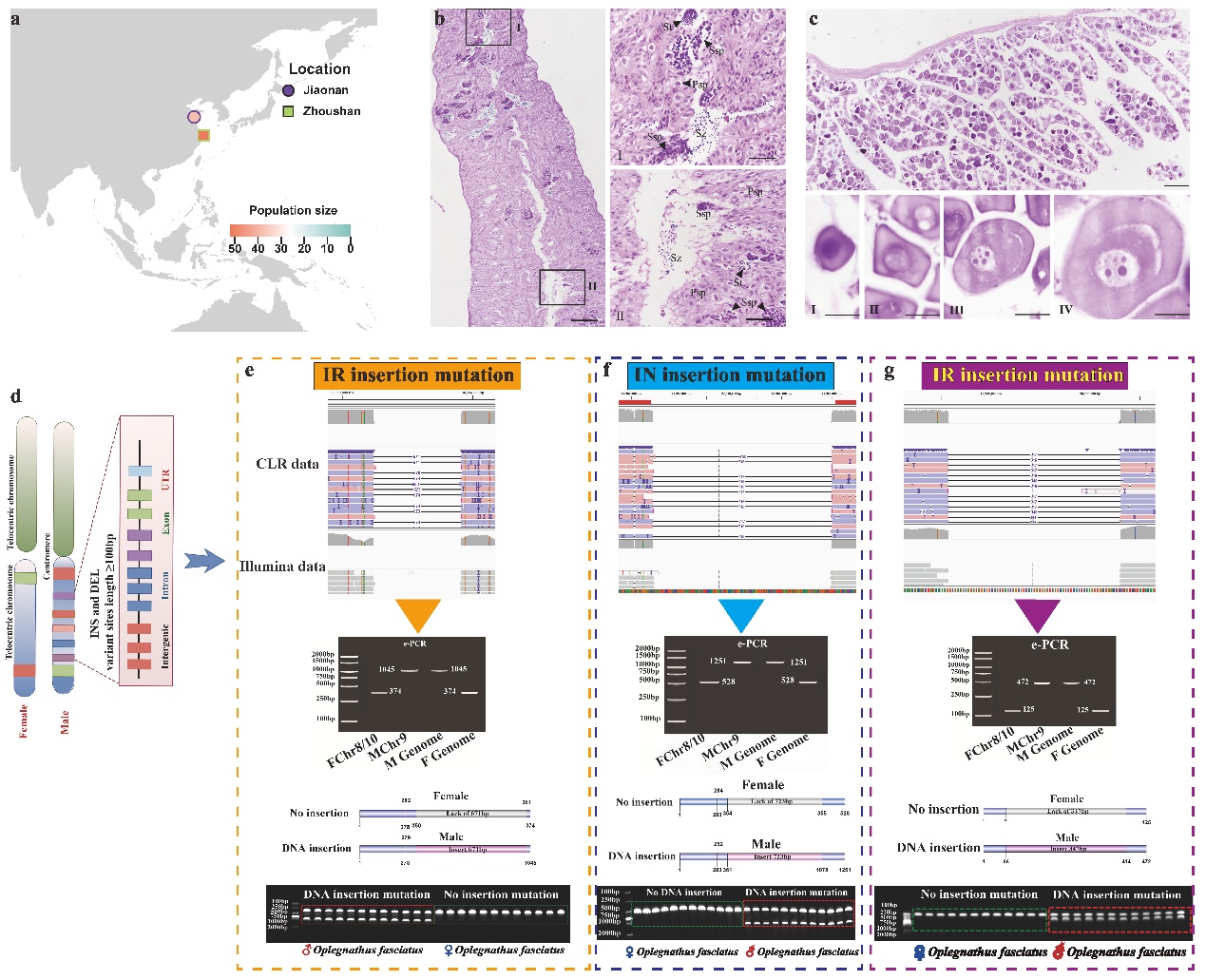


Figure S9. Male and female sex genetic markers were located in the intergenic region (IR), insertion sites in introns (IN) and their validation in different geographic populations, using the male genome as a reference.

(a) Wild population samples were collected from offshore populations in Shandong and Zhejiang. (b) Wild samples were examined histologically for male gonads. Testis histology with 200µm and 40µm for I and II locations. Sz, spermatozoa; St, Spermatid; Ssp, Secondary Spermatocyte; Psp, Primary spermatocyte. (c) Wild samples were examined histologically for female gonads. Ovary histology with 250µm and 25µm. I, primary growth chromatin nucleolar stage; II-III, primary growth perinucleolus stage; II, early perinucleolus stage; III-IV, late perinucleolus stage. (d) Location of male and female sex markers in the male (MChr9) and female (FChr8) genomes. (e) Using the male genome as a reference, male and female sex genetic markers were located at deletion sites in the intergenic region. The e-PCR amplified bands were 374bp and 1045bp in size, corresponding to DNA deletion pattern maps and agarose gel electrophoresis maps for population identification of male and female genetic differences. Two bands were amplified in males and one band in females. (f) Male and female sex genetic markers were located at deletion sites in the intronic region. The e-PCR amplified bands were 528bp and 1251bp in size, corresponding to DNA deletion pattern maps and agarose gel electrophoresis maps for population identification of male and female genetic differences. Two bands were amplified in males and one band in females. (g) Male and female sex genetic markers were located at deletion sites in the intronic region. The e-PCR amplified bands were 125bp and 472bp in size, corresponding to DNA deletion pattern maps and agarose gel electrophoresis maps for population identification of male and female genetic differences. Two bands were amplified in males and one band in females.
