# Supplementary figures and images for: Innovative approach for high-throughput exploiting sex-specific markers in Japanese parrotfish Oplegnathus fasciatus
Source: Gigascience. 2024 Jul 19;13:giae045. doi: 10.1093/gigascience/giae045 (PMC11258905; doi:10.1093/gigascience/giae045)

## ♀ *O. fasciatus* with reference

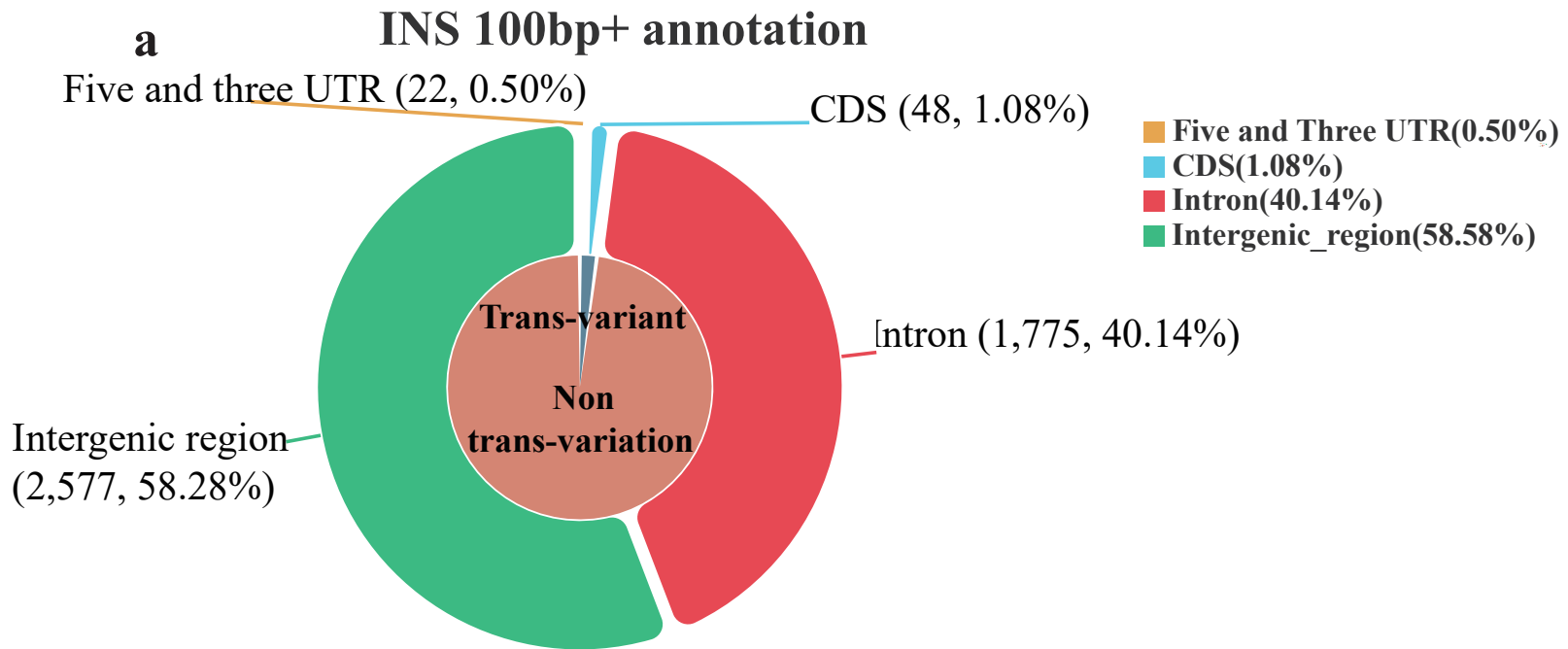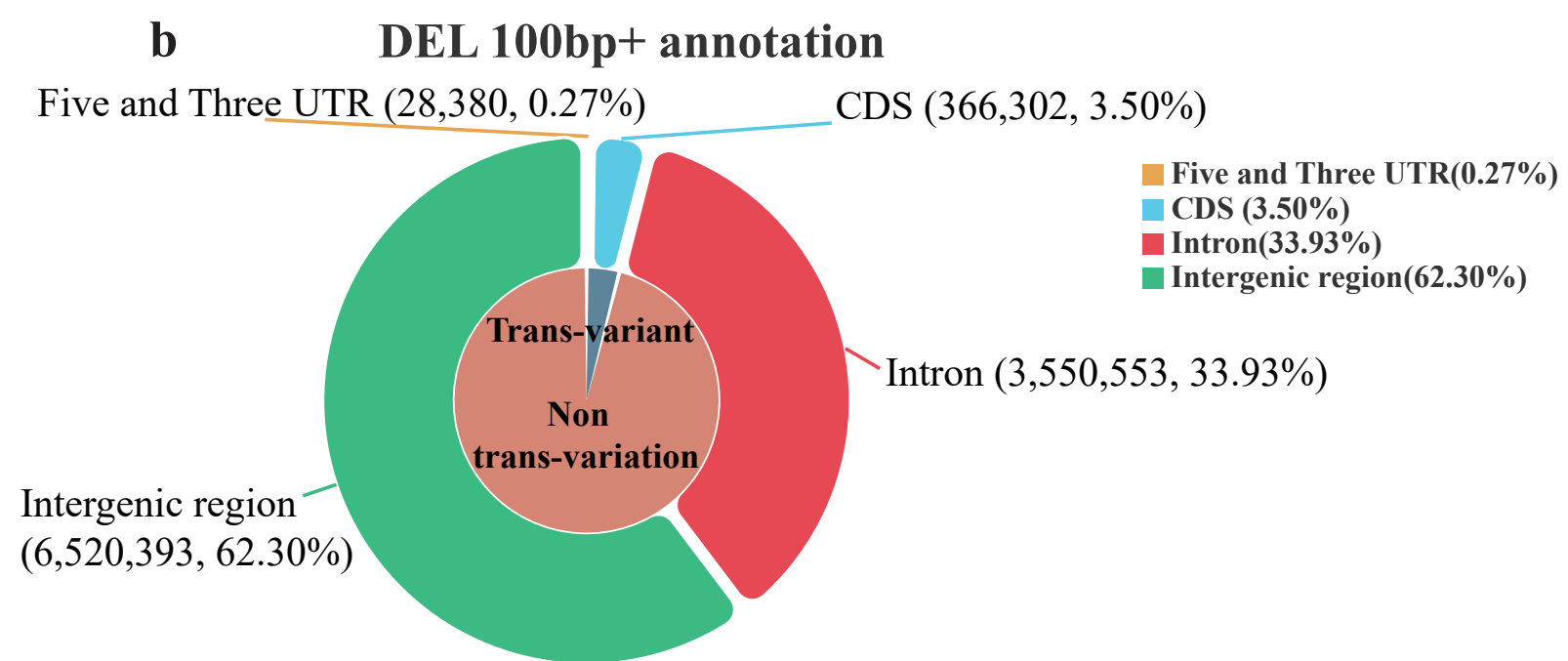

## ♂ *O. fasciatus* with reference

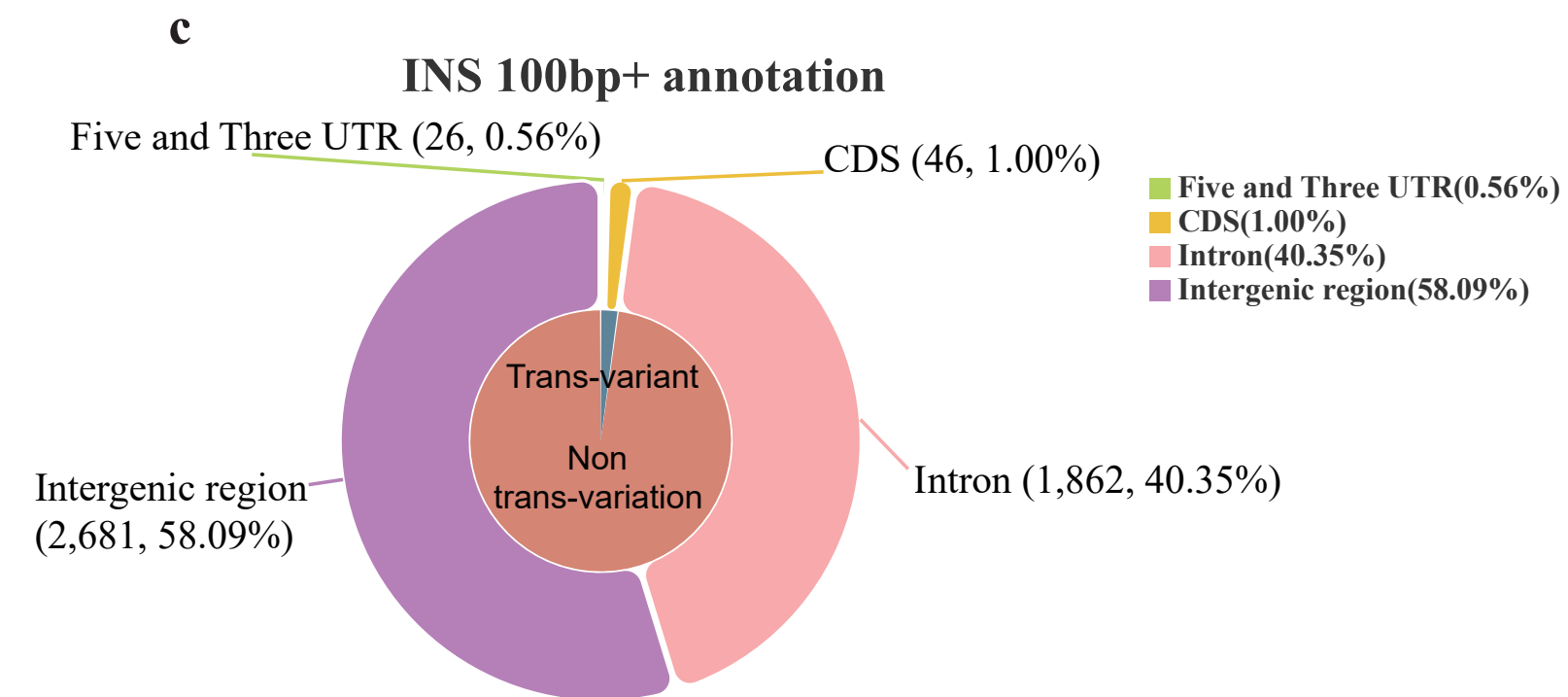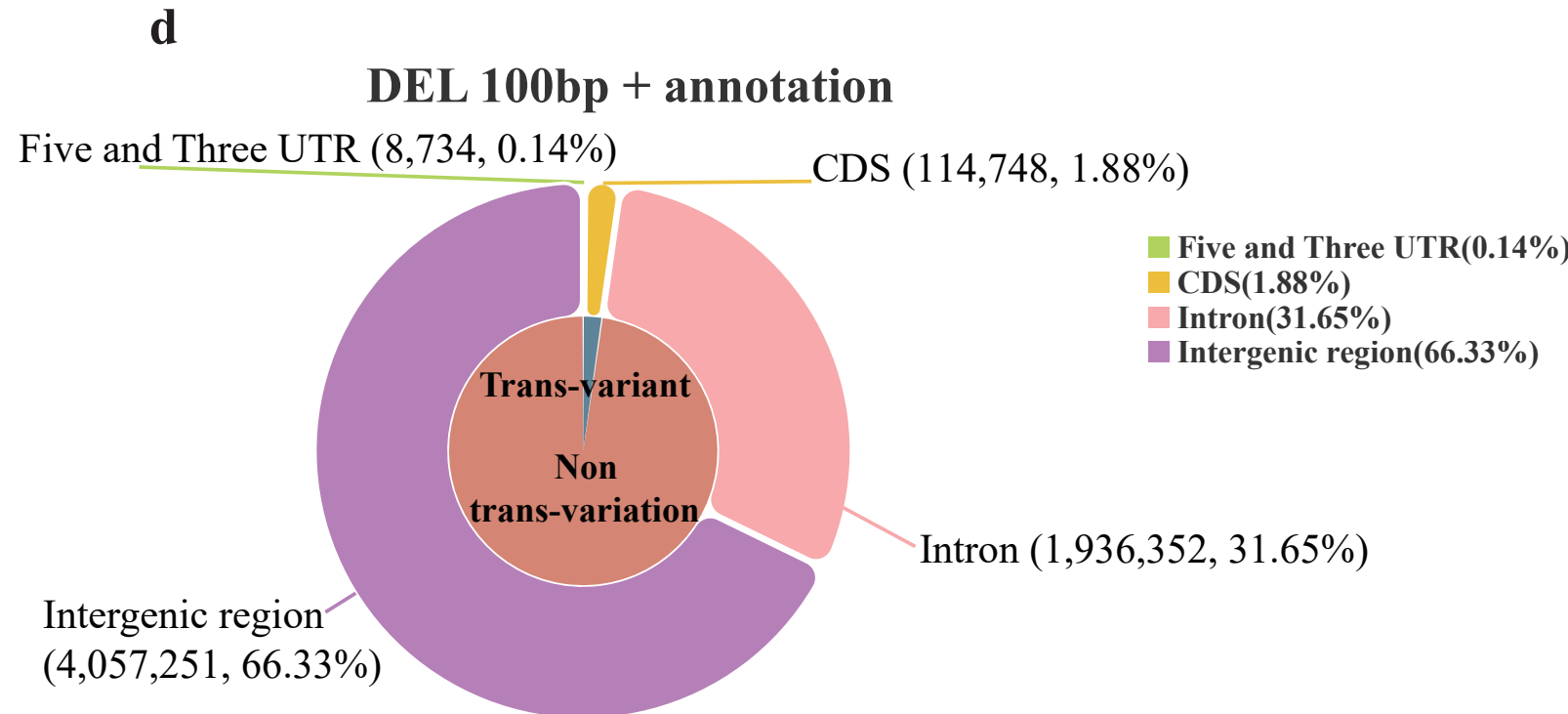

Supplement: giae045_Supplemental_Files [file giae045_supplemental_files.zip › Figure S1.pdf]

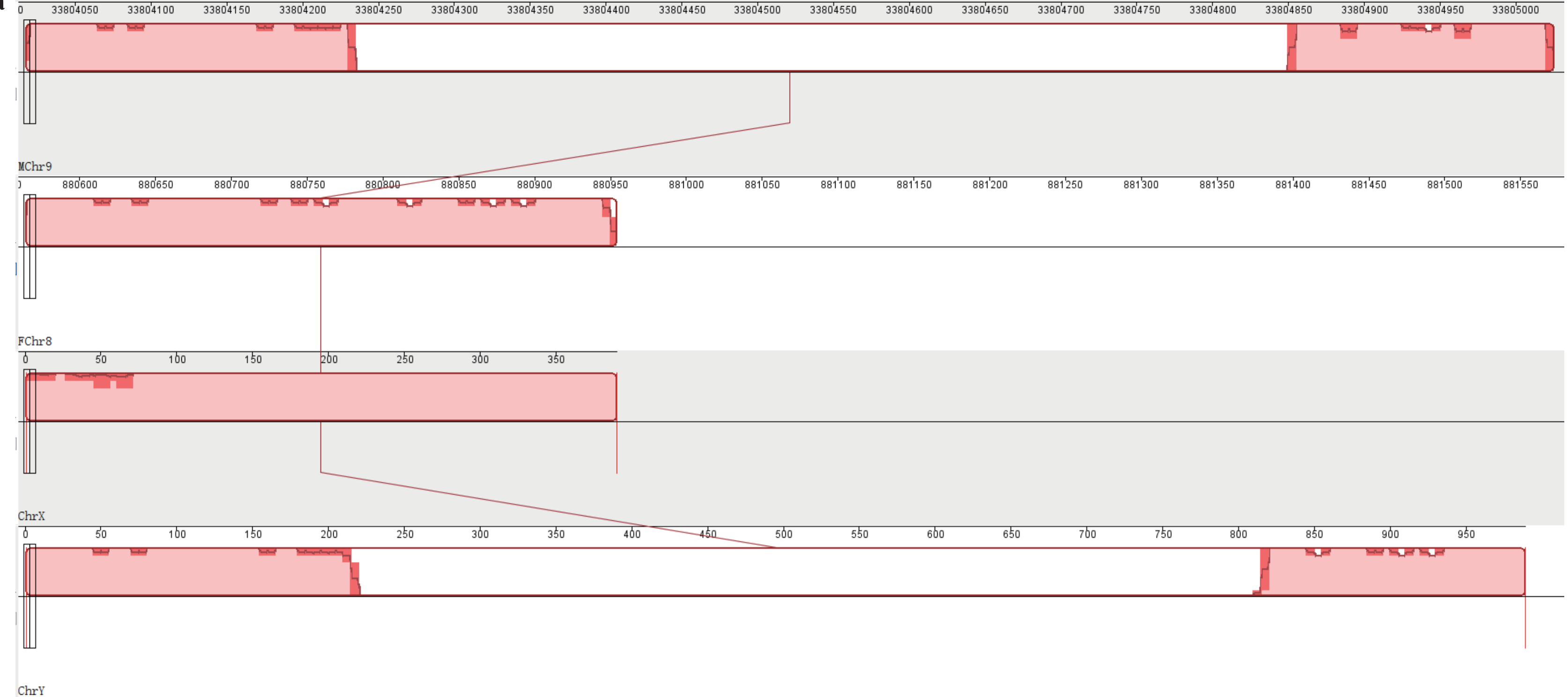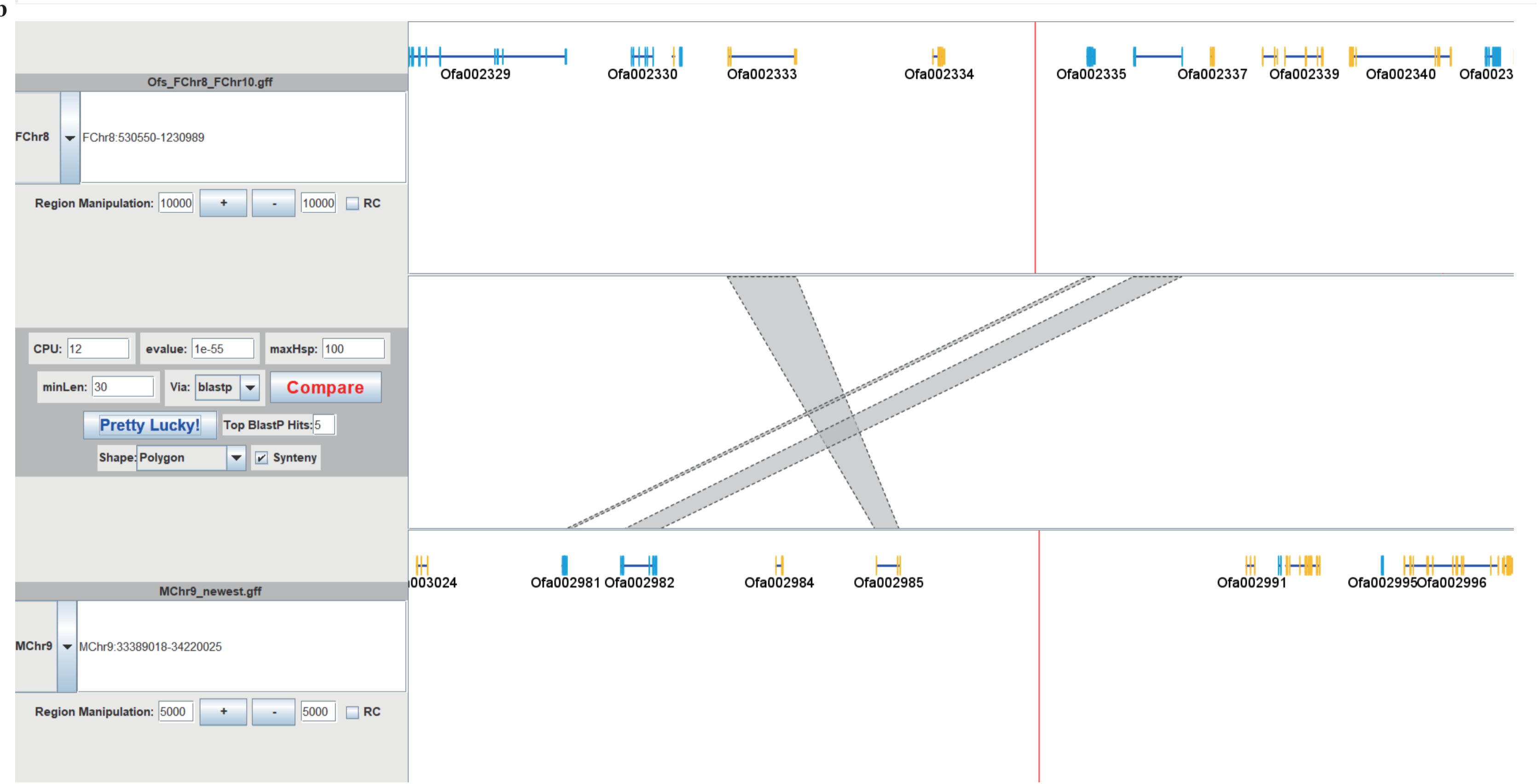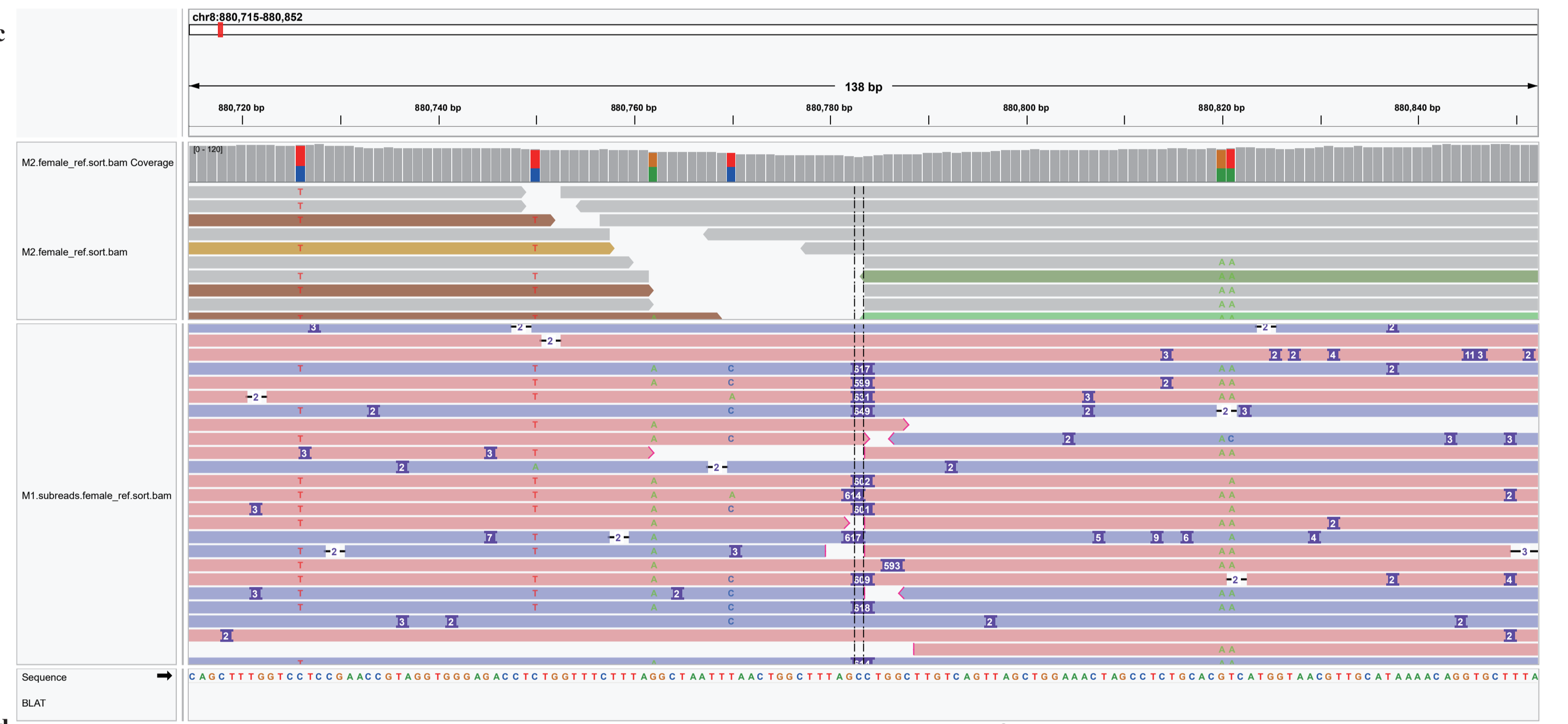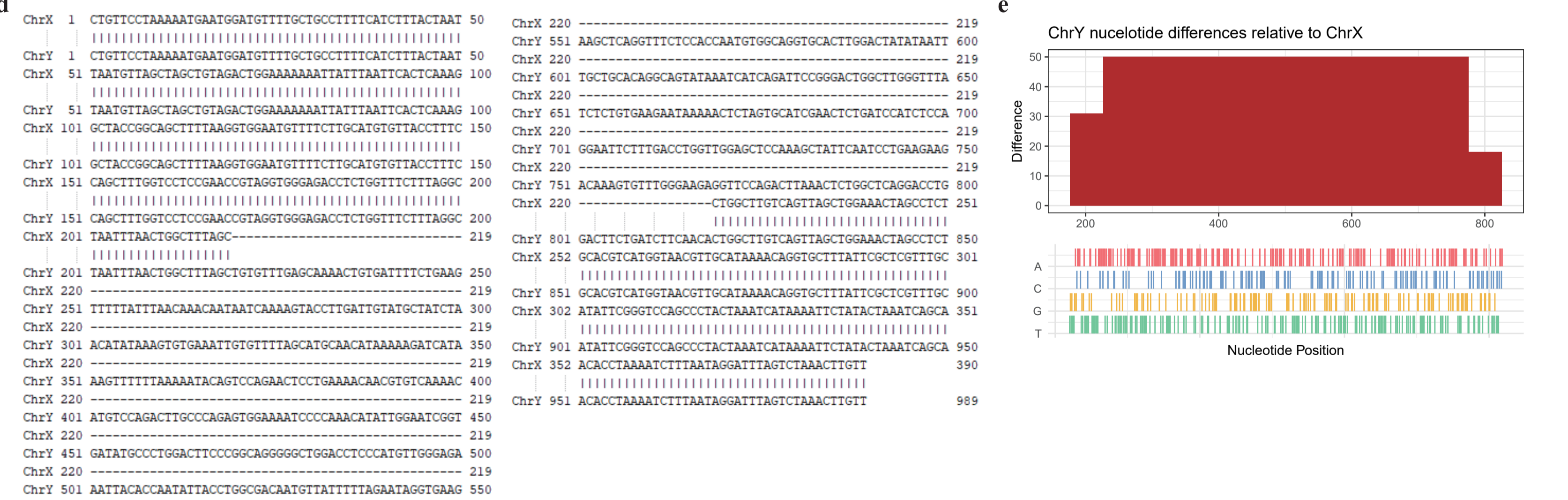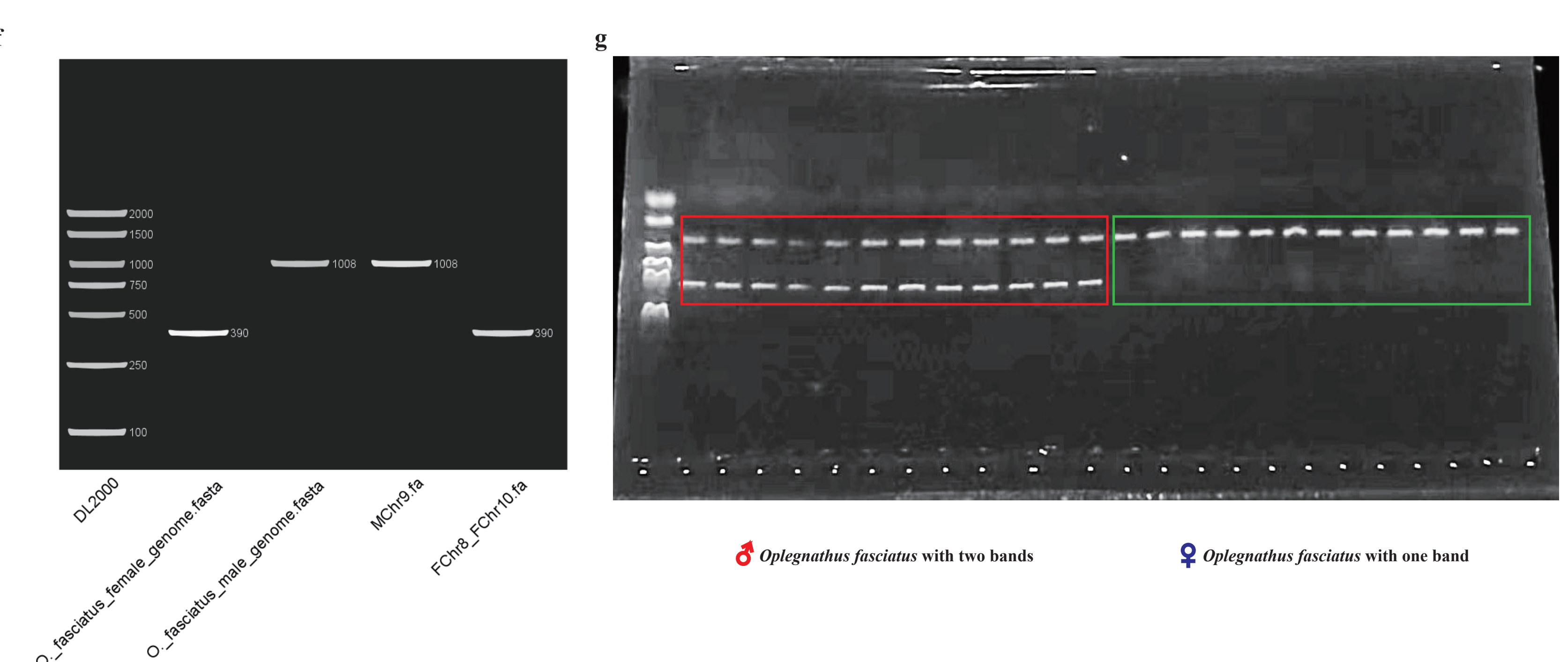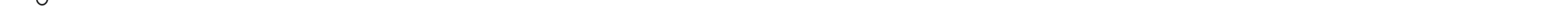

Supplement: giae045_Supplemental_Files [file giae045_supplemental_files.zip › Figure S2.pdf]

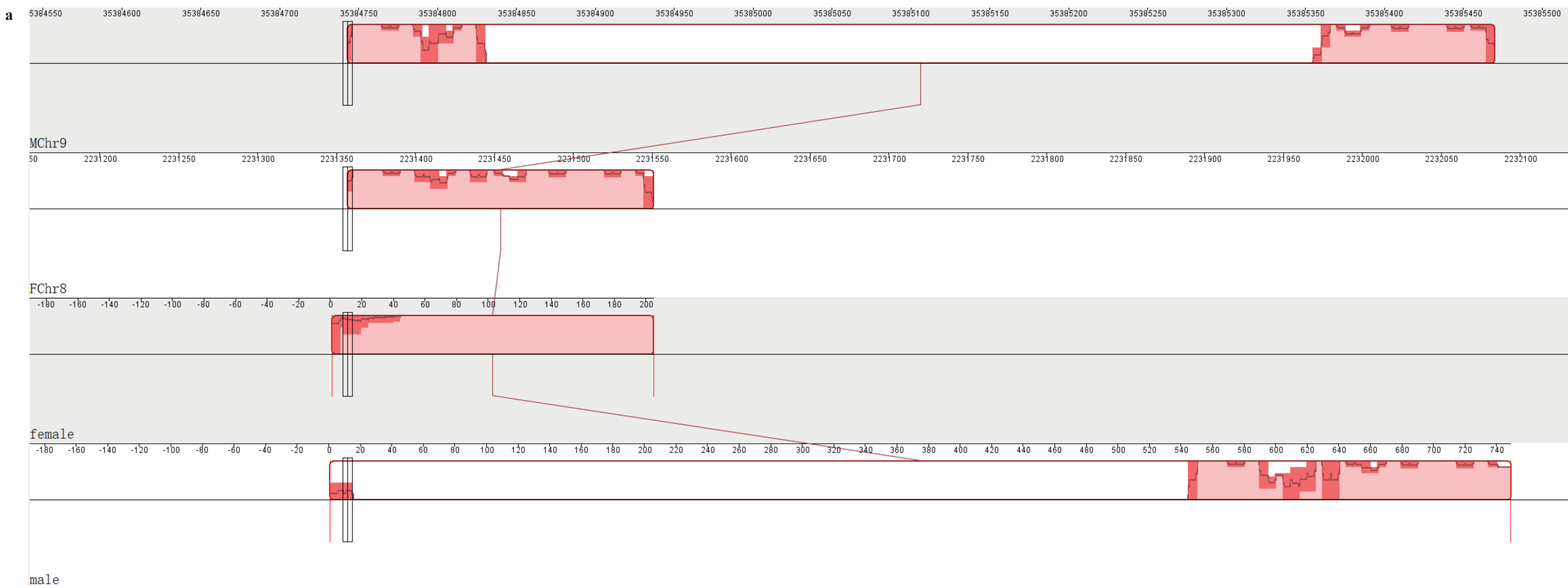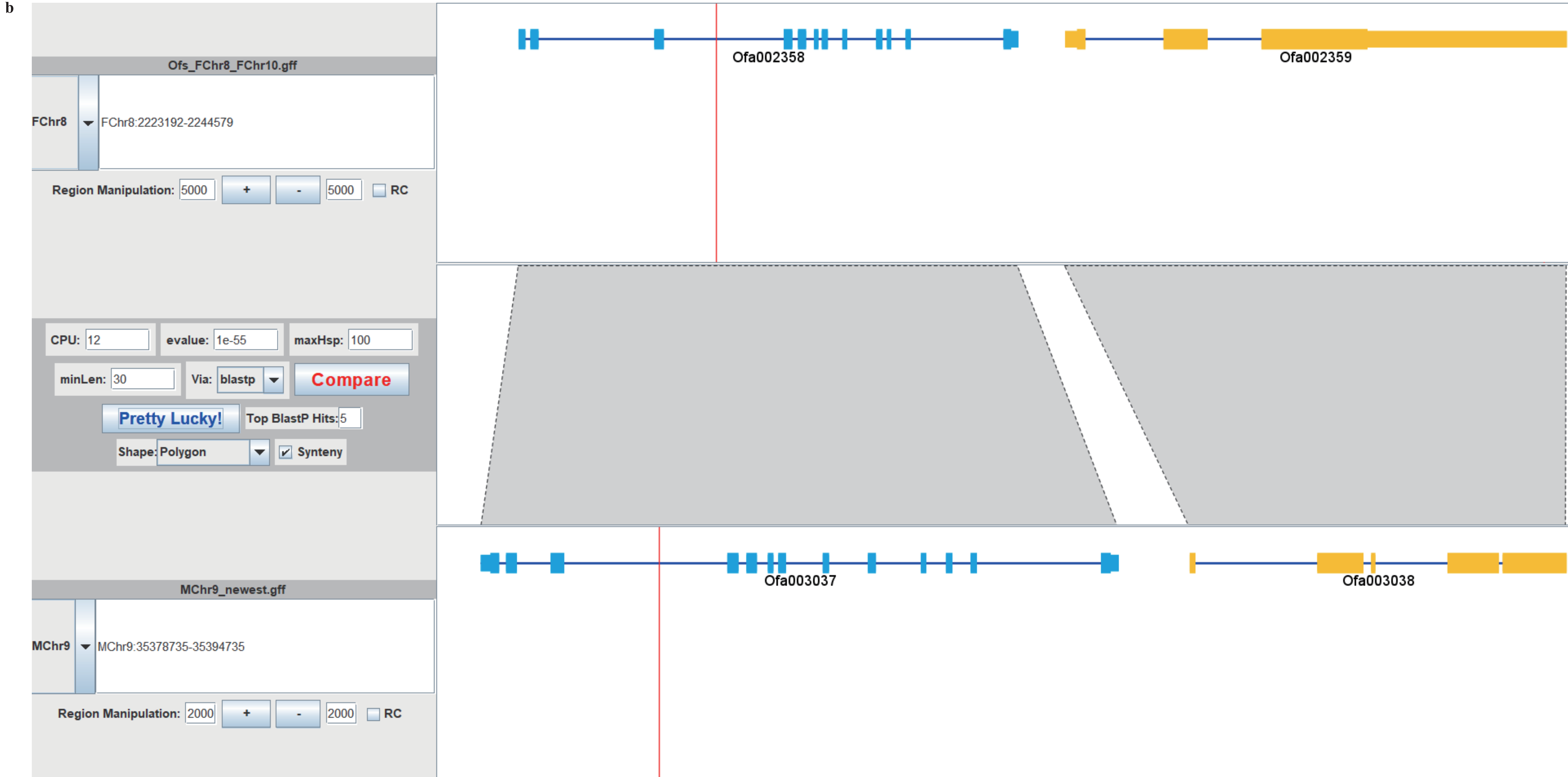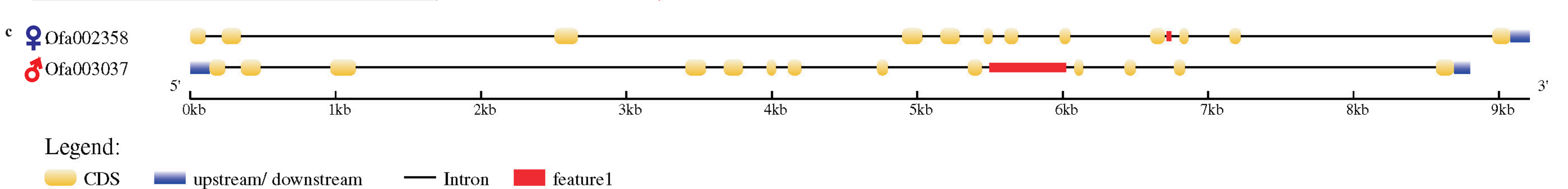

Supplement: giae045_Supplemental_Files [file giae045_supplemental_files.zip › Figure S3.pdf]

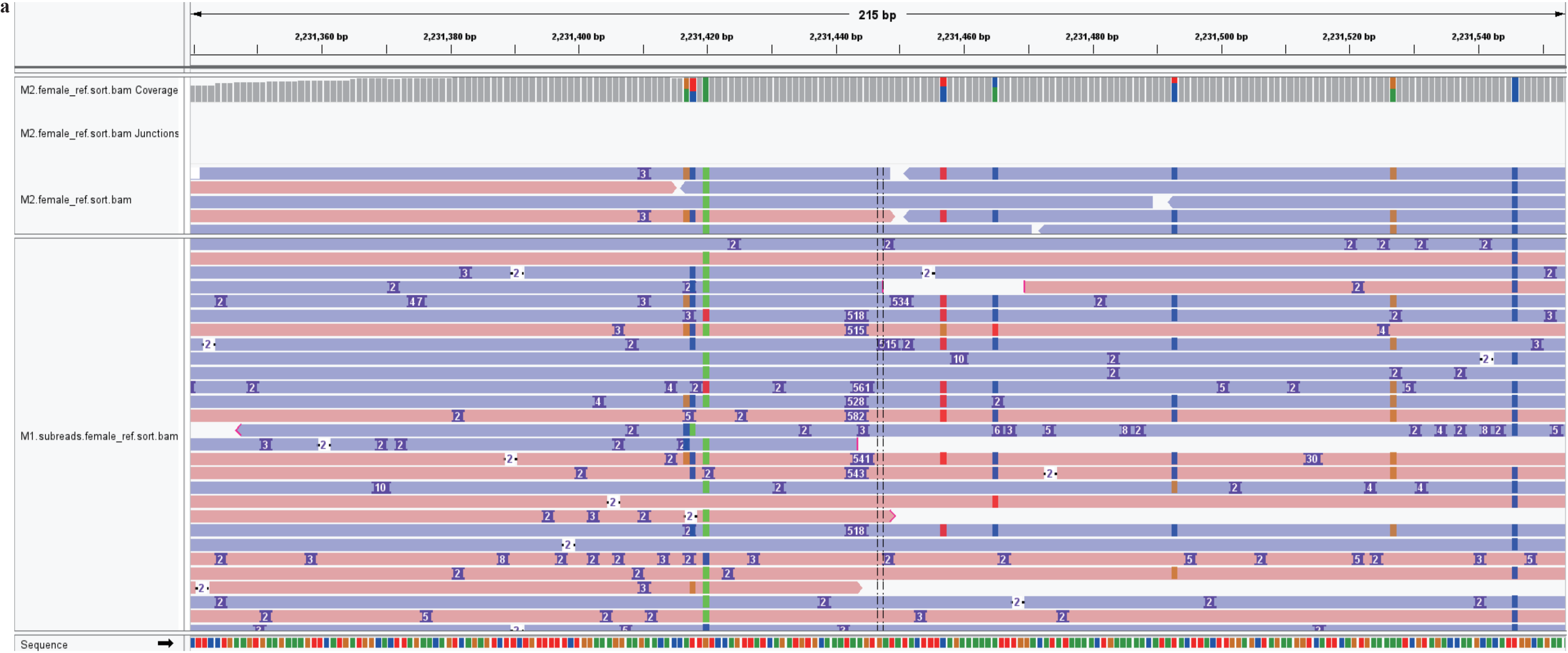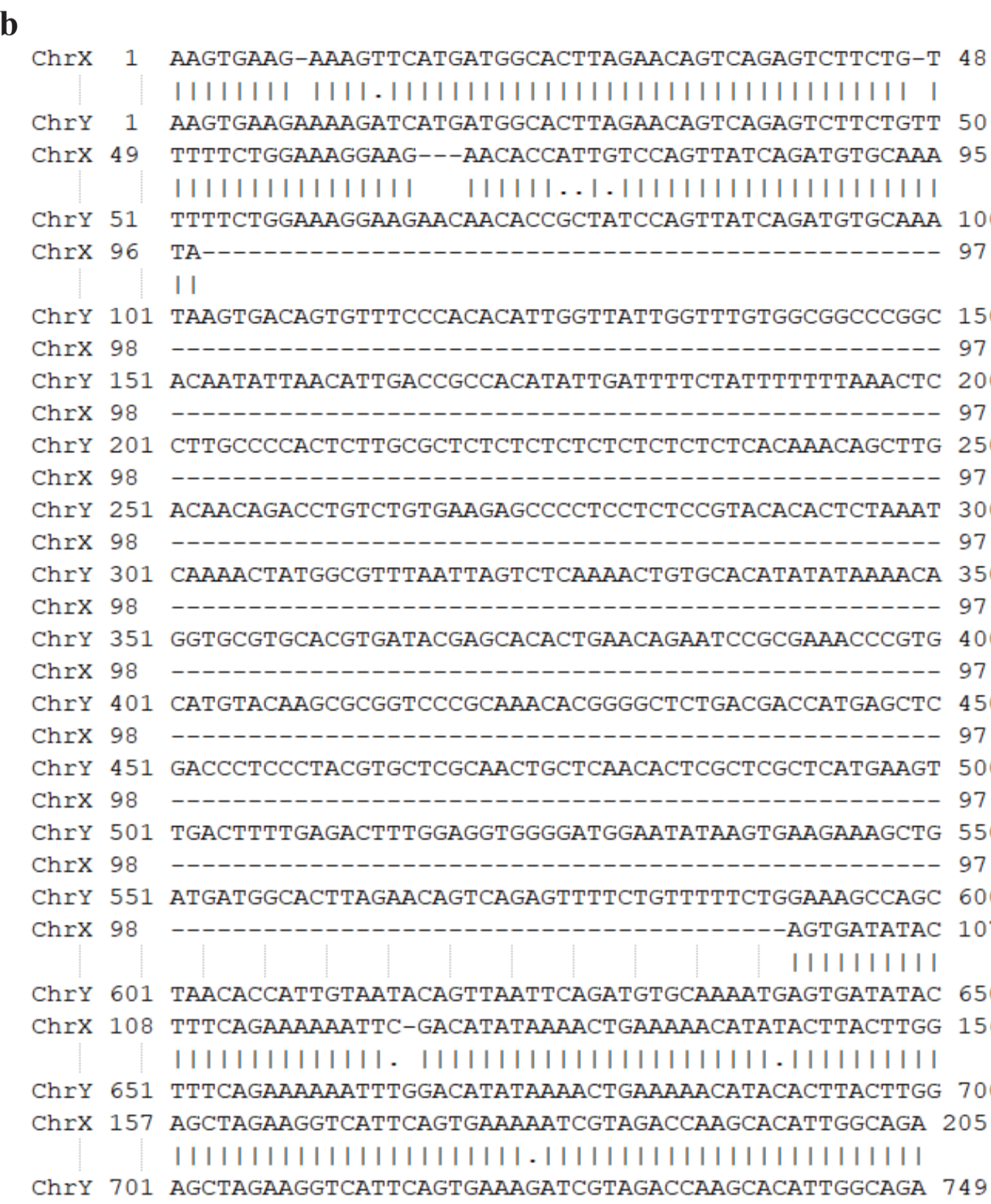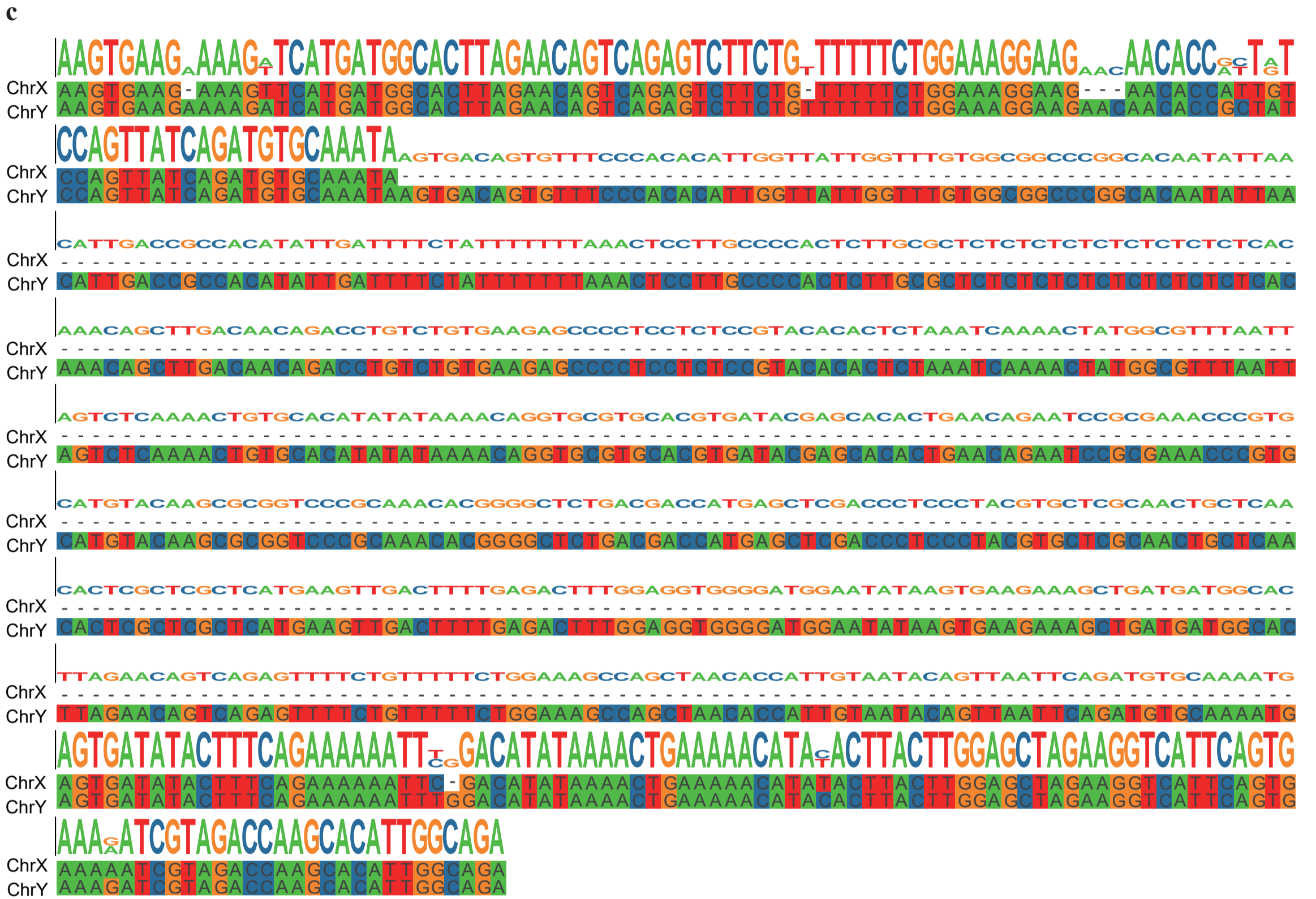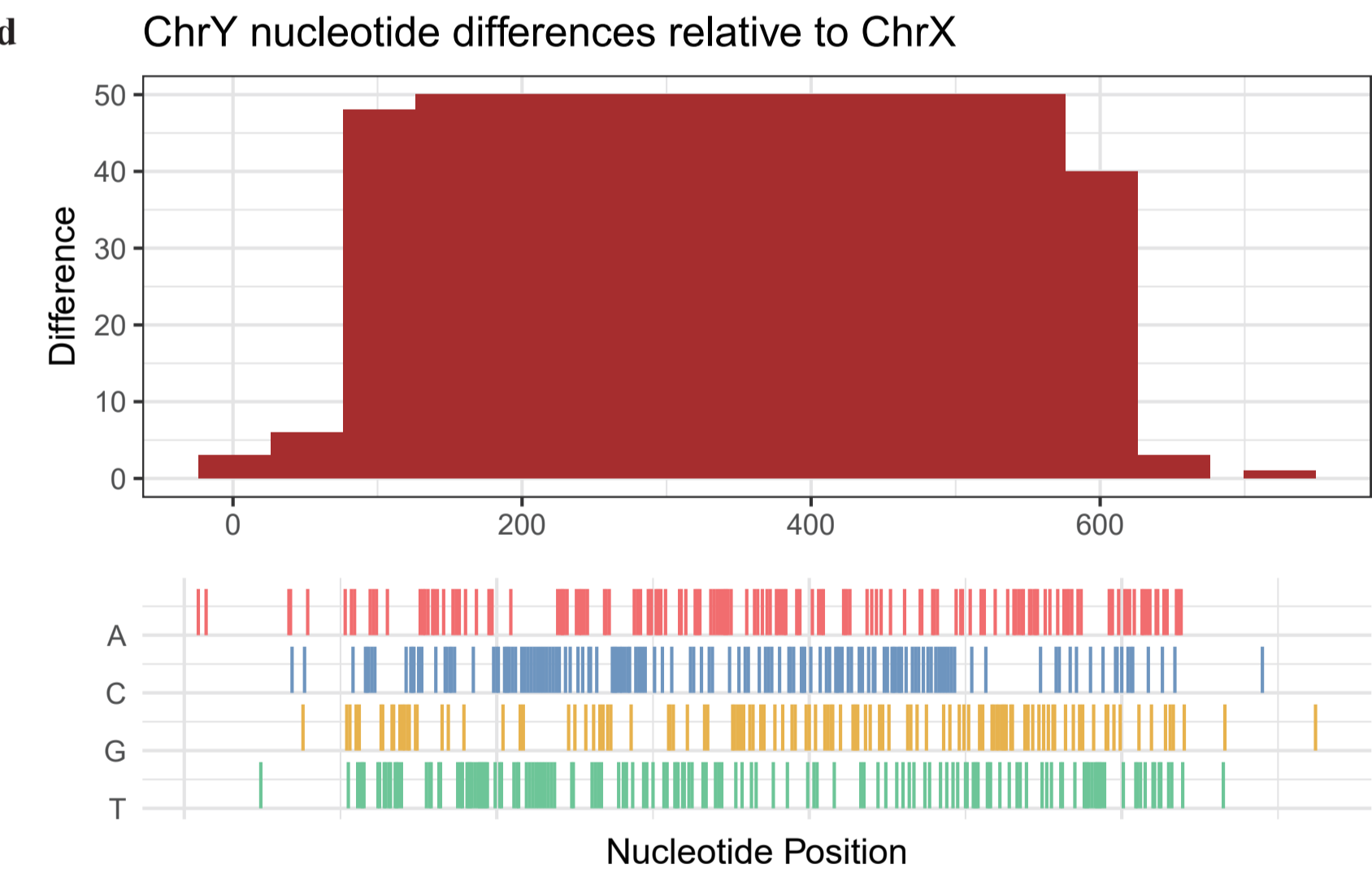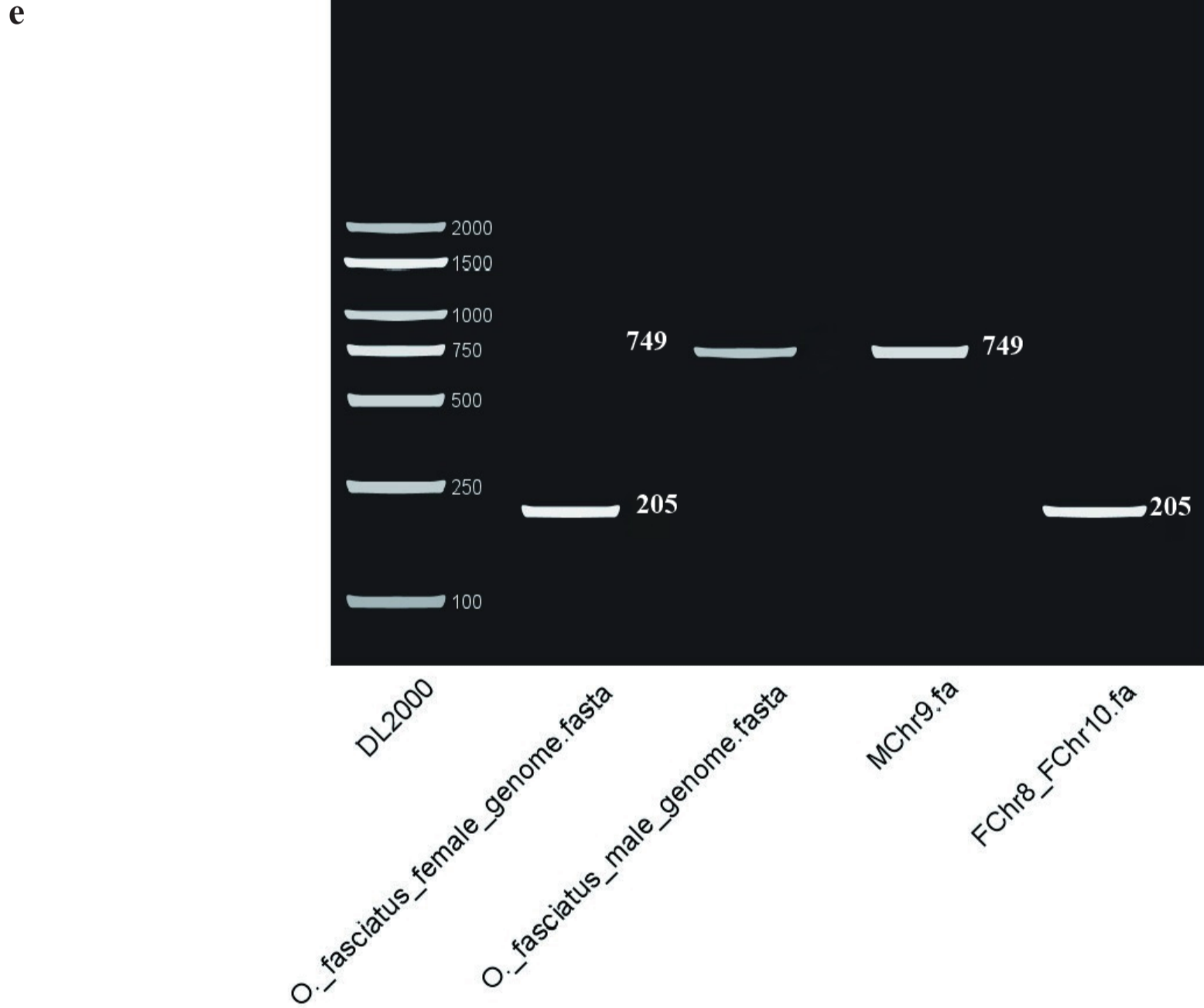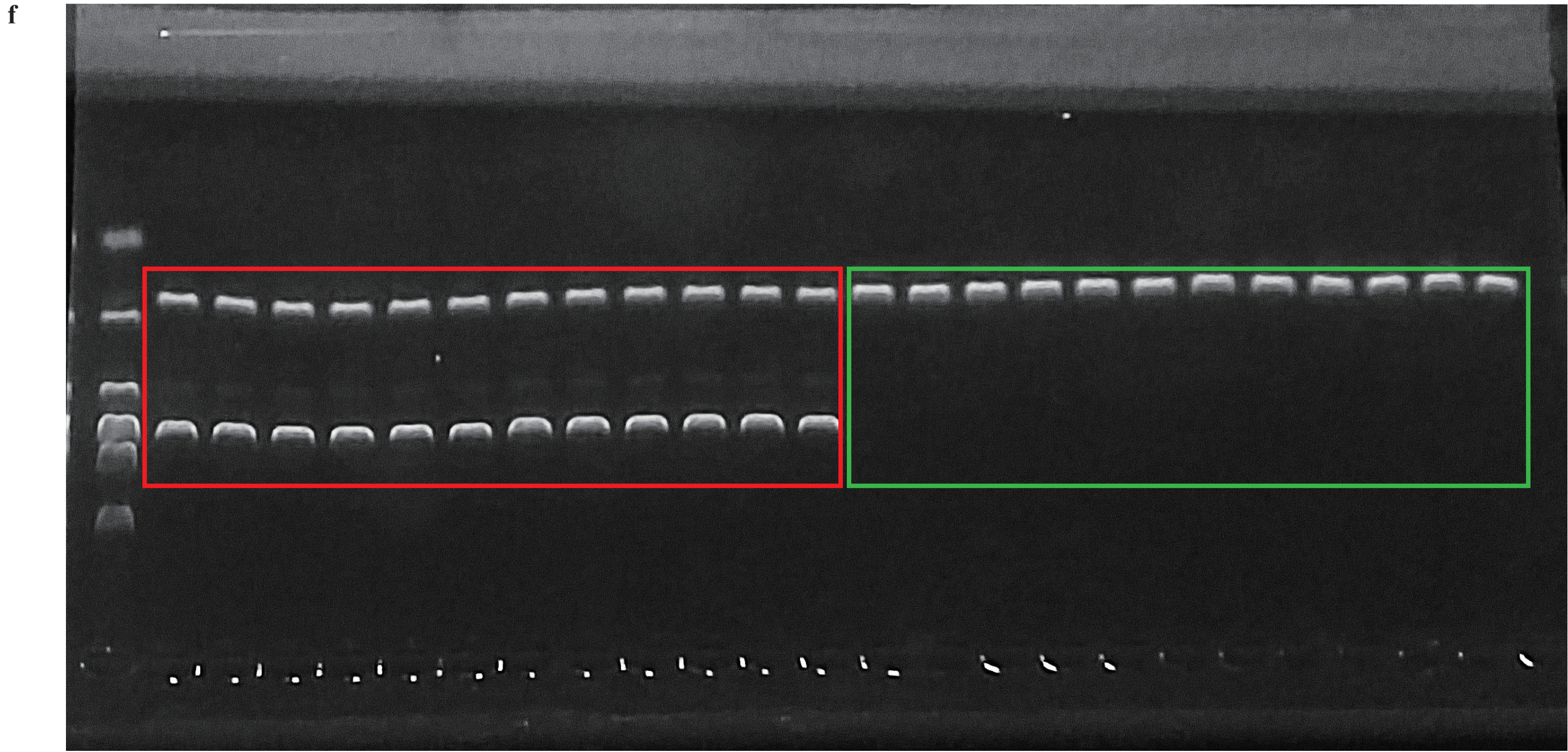

♂ *Oplegnathus fasciatus* with two bands

♀ *Oplegnathus fasciatus* with one band

Supplement: giae045_Supplemental_Files [file giae045_supplemental_files.zip › Figure S4.pdf]

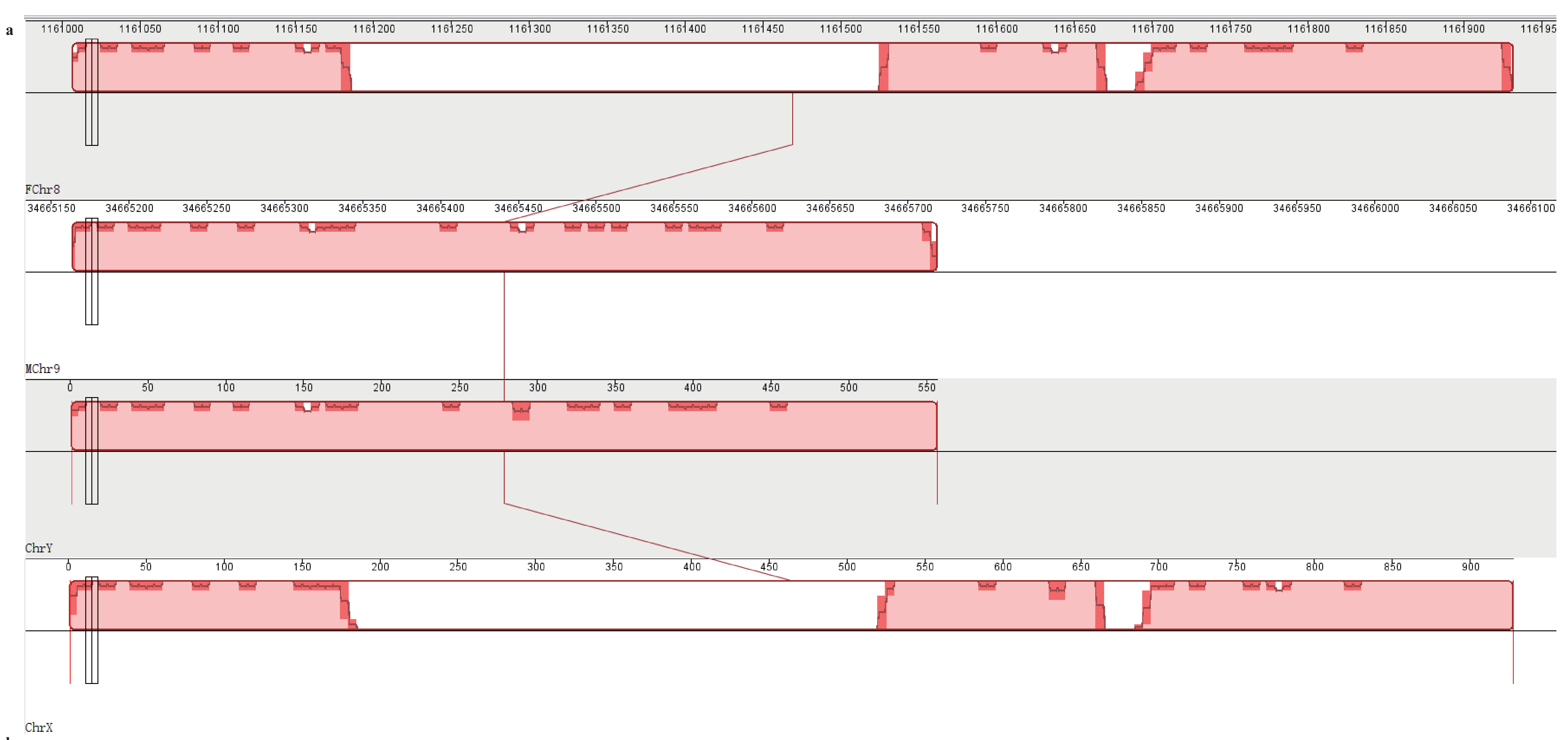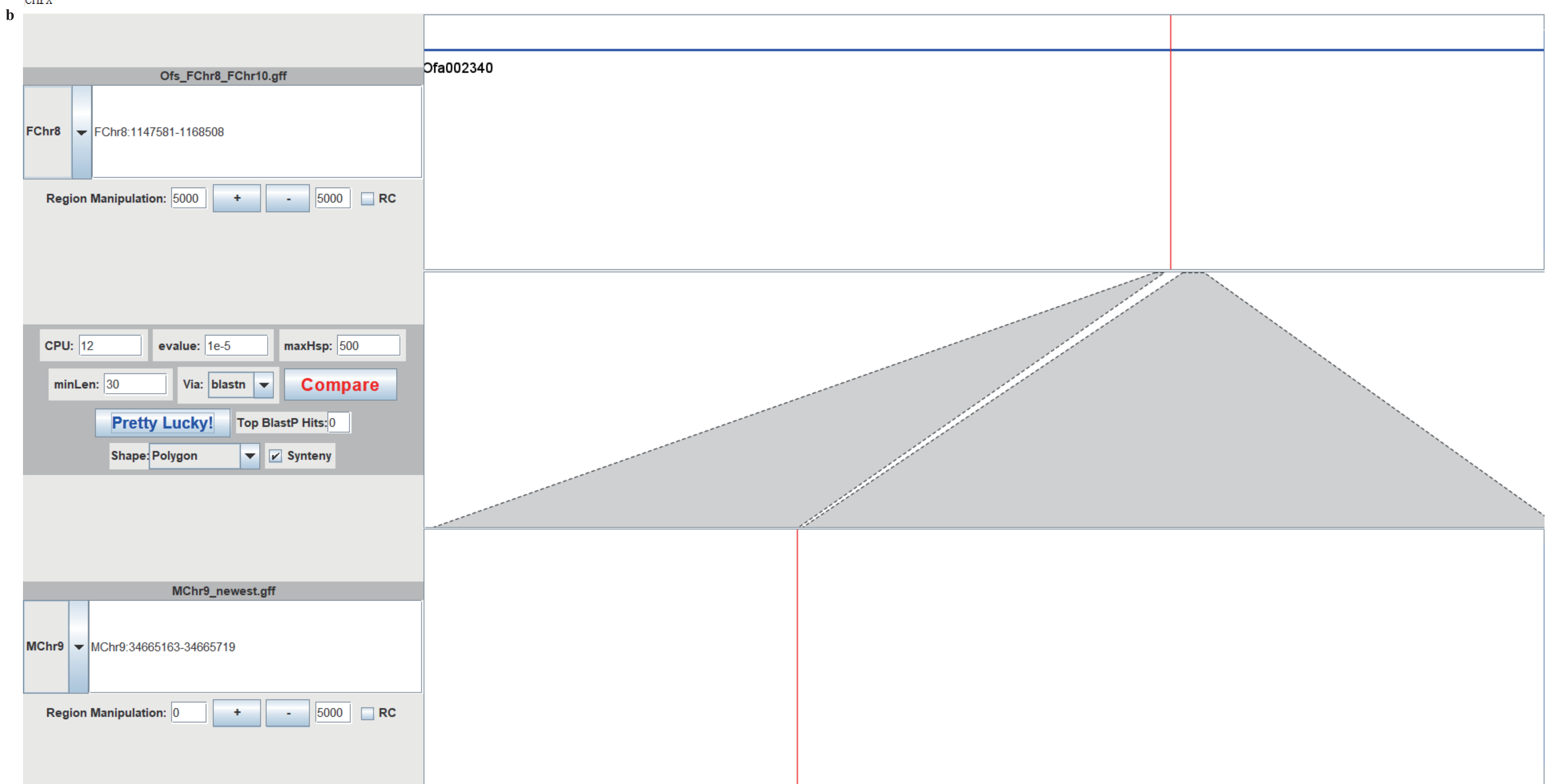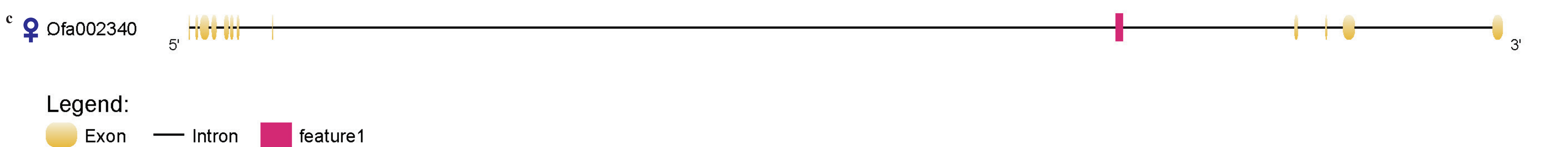

Supplement: giae045_Supplemental_Files [file giae045_supplemental_files.zip › Figure S5.pdf]
