# Supplementary material for: Innovative approach for high-throughput exploiting sex-specific markers in Japanese parrotfish Oplegnathus fasciatus
Source: Gigascience. 2024 Jul 19;13:giae045. doi: 10.1093/gigascience/giae045 (PMC11258905; doi:10.1093/gigascience/giae045)
Supplement: giae045_GIGA-D-23-00373_Revision_1 [file giae045_giga-d-23-00373_revision_1.pdf]

GigaScience

Innovative approach for high-throughput exploiting sex-specific markers in japanese parrotfish Oplegnathus fasciatus

--Manuscript Draft--

**Manuscript Number:**	GIGA-D-23-00373R1
**Full Title:**	Innovative approach for high-throughput exploiting sex-specific markers in japanese parrotfish Oplegnathus fasciatus
**Article Type:**	Research
**Funding Information:**	National Outstanding Youth Science Fund Project of National Natural Science Foundation of China (No. 42276107; No. 32270472) — Prof Yongshuang Xiao
**Abstract:**	Background<br>The use of sex-specific molecular markers has become a prominent method in enhancing fish production and economic value, as well as providing a foundation for understanding the complex molecular mechanisms involved in fish sex determination. Over the past decades, research on male and female sex identification has predominantly employed molecular biology methodologies such as RFLP (Restriction Fragment Length Polymorphism), RAPD (Random Amplification of Polymorphic DNA), SSR (Simple Sequence Repeat), and AFLP (Amplified Fragment Length Polymorphism). The emergence of high-throughput sequencing technologies, particularly Illumina, has led to the utilization of SNP and InDel variants as significant molecular markers for investigating sex identification in fish. The advancement of sex-controlled breeding encounters numerous challenges, including the inefficiency of current methods, intricate experimental protocols, high costs of development, elevated rates of false positives, marker instability, and cumbersome field testing procedures. Nevertheless, the emergence and swift progress of PacBio high-throughput sequencing technology, characterized by its long read output capabilities, offers novel opportunities to overcome these obstacles.<br>Findings<br>Utilizing male-female assembled genome information in conjunction with short-read sequencing data survey and long-read PacBio sequencing data, a catalog of large-segment (>100 bp) insertion-deletion genetic variants was generated through a genome-wide variant site scanning approach with bidirectional comparisons. The sequence tagging sites (STS) were ranked based on the long-read depth of the insertion deletion site, with markers exhibiting lower long read depth being considered more effective for large segment deletion variants. Subsequently, a catalog of bulk primers and simulated PCR for the male-female variant loci was developed, incorporating primer design for the target region and e-PCR technology. The Japanese parrotfish (Oplegnathus fasciatus), belonging to the Oplegnathidae family within the Centrarchiformes order, holds significant economic value as a rocky reef fish indigenous to East Asia. The criteria for rapid identification of male and female differences in O. fasciatus were established through agarose gel electrophoresis, which revealed two amplified bands for males and one amplified band for females. A high-throughput identification catalog of sex-specific markers was then constructed using this method, resulting in the identification of 3,639 (2,786 INS/853 DEL, ♀ as reference) and 3,672 (2,876 INS/833 DEL, ♂ as reference) markers in conjunction with 1,021 and 894 high-quality genetic sex identification markers, respectively. Sixteen differential loci were randomly chosen from the catalog for validation, with 11 of them meeting the criteria for male-female distinctions. The implementation of cost-effective and efficient technological processes would facilitate the rapid advancement of genetic breeding through expediting the high-throughput development of sex genetic markers for various species.<br>Conclusions<br>Our study utilized assembled genome information from male and female individuals obtained from PacBio, in addition to data from short-read sequencing data survey and long-read PacBio sequencing data. We extensively employed genome-wide variant site scanning and identification, high-throughput primer design of target regions, and e-PCR batch amplification, along with statistical analysis and ranking of the long reads depth of the variant sites. Through this integrated approach, we successfully compiled

Powered by Editorial Manager® and ProduXion Manager® from Aries Systems Corporation

	a catalog of large insertion/deletion sites (>100 bp) in both male and female O. fasciatus.
**Corresponding Author:**	Yongshuang Xiao, phD<br>Institute of Oceanology Chinese Academy of Sciences<br>Qingdao, CHINA
**Corresponding Author Secondary Information:**	
**Corresponding Author's Institution:**	Institute of Oceanology Chinese Academy of Sciences
**Corresponding Author's Secondary Institution:**	
**First Author:**	shuang Yong Xiao
**First Author Secondary Information:**	
**Order of Authors:**	shuang Yong Xiao
	Yongshuang Xiao, phD
	Zhizhong Xiao
	Yuting Ma
	Haixia Zhao
	Yanduo Wu
	Jinwei Huang
	Pingrui Xu
	Lin Liu
	Jing Liu
	Jun Li
**Order of Authors Secondary Information:**	
**Response to Reviewers:**	Reviewer #1:<br>In this manuscript, Xiao et al performed bioinformatic analysis of Illumina and PacBio sequencing data to detect insertion/deletion variants, coupled with PCR based experimental validation to enable identification of genetic sex markers in a fish species. Overall, the manuscript main strength is the findings of sex specific markers in both male and female genomes as clearly supported by PCR results. But there are still a few shortcomings that needs to be addressed. Below I explain my major points:<br><br><br>In this manuscript, the authors assert that they have created a database of insertion/deletion-based sex-markers. After reviewing, it appears that this claim may be overselling their findings. In bioinformatics, the term "database" typically refers to an online portal for users to explore data. Therefore, it is suggested to consider revising the term "database" to "variants catalog" for clarity and accuracy.<br>Reply:<br>By carefully reading the reviewer' comments, we realized that we had indeed obtained a catalog rather than a database of male and female sex markers for Oplegnathus fasciatus, which was a conceptual error. We would like to thank the reviewer for his professional advice. Based on the reviewer's comments, we have revised all the technical procedures and database-related contents of the paper into catalogs, and the list of changes is as follows:<br>1. We have changed "Datasets" in "All Chromosome variants Dataset", "Bulk Primer Design for Variant Datasets" and "Batch e-PCR of Variant Datasets" to "All Chromosome variants catalog", " Bulk Primer Design for Variant catalog ", and “Batch e-PCR of Variant catalog” respectively, in the technology flow. Please see the figure below.<br><br>2. Line 46, we revised “a database of large-segment (>100 bp) insertion–deletion” as

Powered by Editorial Manager® and ProduXion Manager® from Aries Systems Corporation

	"a catalog of large-segment (>100 bp) insertion–deletion".<br>3. Line 48, we revised "A database of bulk primers and simulated PCR for the…." as "A catalog of bulk primers and simulated PCR for the….".<br>4. Line 53-54, we revised "A high-throughput identification database of sex-specific markers for Oplegnathus fasciatus was constructed utilizing this method…" as "A high-throughput identification catalog of sex-specific markers for Oplegnathus fasciatus was constructed utilizing this method".<br>5. Line 56, we revised "Four differential loci were randomly selected from the database for validation, and the……" as "Four differential loci were randomly selected from the catalog for validation, and the…….".<br>6. Line 68, we revised "we successfully developed a database encompassing insertion/deletion loci in large segments (>100 bp)" as "we successfully developed a catalog encompassing insertion/deletion loci in large segments (>100 bp)".<br>7. Line 145, we revised "In this work, a database of genomic differences between females and males was" as "In this work, a catalog of genomic differences between females and males was".<br>8. Line 147-148, we revised "Using the difference database, we obtained datasets of insertion and deletion variant sites larger than 100 bp…." as "Using the difference catalog, we obtained catalogs of insertion and deletion variant sites larger than 100 bp…..".<br>9. Line 166-167, we revised "a high-throughput precision marker dataset for male and female genetic sex identification was obtained and tested by agarose gel electrophoresis.." as "a high-throughput precision marker catalog for male and female genetic sex identification was obtained and tested by agarose gel electrophoresis….".<br>10. Line 176-177, we revised "we established databases containing genomic disparities between males and females." as "we established catalogs containing genomic disparities between males and females.".<br>11. Line 178-179, we revised "as well as to screen large insertion-deletion sites (>100 bp) against a differential database," as "as well as to screen large insertion-deletion sites (>100 bp) against a differential catalogs,".<br>12. Line 221-222, we revised "we screened this insertion/deletion variant site database for variants larger than" as "we screened this insertion/deletion variant site catalogs for variants larger than".<br>13. Line 462-463, we revised "we successfully developed a database encompassing insertion/deletion loci in large segments (>100 bp) for both male and female O. fasciatus" as "we successfully developed a catalog encompassing insertion/deletion loci in large segments (>100 bp) for both male and female O. fasciatus".<br><br>In addition to mapping Illumina and PacBio reads against references, did the author also consider to directly map both male and female genomes using pairwise whole-genome aligner (for example LASTZ) to see the differences in both genomes? Additionally, I did not find any relevant information in the manuscript on the quality of reference genomes assemblies for male and female fish used for the reads alignments. Authors should clarify whether or not the variants detection was affected by any possible artifacts in the genome assemblies?<br>Reply:<br>We would like to extend our sincere thanks to you for your insights and thoughtful suggestions. And it was your suggestion that led us to introduce a new assay to assess the validity of the large fragment insertion deletion sites we obtained.<br>We used SYRI software for the evaluation of variant sites (≥100bp) between the male and female reference genomes of Oplegnathus fasciatus, and using the male genome as a reference for comparison with the female genome, we obtained 2045 deletion sites (≥100bp) and 2110 insertion sites (≥100bp). Meanwhile, we obtained 2116 large segment deletion sites (≥100bp) and 2055 large segment insertion sites (≥100bp) by comparing the female genome with the male genome as a reference. Considering large segment deletion sites better represents the visualization differences between male and female genomes, especially when performing the second generation Illumina and the third generation PacBio CLR data read segments repost visualization presentation. We found that by using the intersection of deletion sites, the results of SYRI scan using male fish as the reference genome showed that 1749 variants out of 2045 deletion sites were in our deletion site catalog set, which accounted for 85.53% of the total number of deletion sites. The results using female fish as the reference genome showed that 1791 out of 2116 deletion sites were in our deletion site catalog set, accounting for 84.64% of the total number of deletion sites. The results of SYRI

	confirmed that we obtained the large insertion deletion sites of Oplegnathus fasciatus are stable, and also obtained more information about the large insertion deletion site for O. fasciatus. We have put the information of large segment insertion deletion sites obtained by SYRI and the information of large segment insertion deletion sites intersected with those obtained by us for male and female differences as a supplementary result file in the Appendix, and the intersection results are also provided.<br><br>The SYRI software runs the following script:<br>1) nucmer --mum -l 100 -c 1000 FM.fasta OfM_230823_filter_rename.format.fa<br>2) delta-filter -i 90 -l 100 out.delta > out.filtered.delta<br>3) Show-coords -THrd out.filtered.delta > out.filtered.coords<br>4) python3 syri_env/bin/syri -c out.filtered.coords -d out.filtered.delta -r FM.fasta -q OfM_230823_filter_rename.format.fa --nosnp<br>As your mentioned, we should have stated in the text the assembly quality evaluation parameters of the reference genome used for genetic sex marker exploitation. Therefore, we supplemented the text with the following quality parameters for the assembly of the Oplegnathus fasciatus male and female genomes as follows:<br>“The assembly of the male and female genomes of O. fasciatus was successfully completed utilizing PacBio CLR sequencing technology in conjunction with Hi-C assisted assembly technology. The resulting genomes achieved high quality at the chromosome level, with the female genome size measuring 768.81 Mb, contig N50 at 2.13 Mb, and scaffold N50 at 33.55 Mb, while the male genome size was 762.27 Mb, contig N50 at 2.18 Mb, and scaffold N50 at 32.43 Mb. The Benchmarking Universal Single-Copy Orthologs (BUCO) analysis of the genome assemblies for males and females demonstrated a high level of completeness, with percentages reaching 96.6% and 97.2%, respectively.”<br>As you noted, it is important to consider the potential impact of artifacts on the variant sites obtained in the genome assembly. We have incorporated your suggestion by conducting an analysis of the reads depth of the variants, specifically focusing on insertion and deletion sites. This analysis involved counting the reads depth for these sites using male and female reference genomes, and ranking the reads depth of the large fragments of the variants. By implementing this approach, we aimed to enhance the accuracy of the sex marker and mitigate the influence of assembly artifacts on the results.<br>For the efficiency of long-read based sex-specific markers, authors should clarify how many variants were experimentally validated ? Were there any cases of false-positive variants?<br>Reply:<br>We express our gratitude to you for your valuable feedback and suggestions. The development of low-cost high-throughput gender markers utilizing long reads and genomic information represents a research endeavor of significant revolutionary potential. Our current focus is on improving and validating the efficacy of sex-specific markers based on long reads. To this end, we are undertaking a two-pronged approach: first, assessing the stability and reliability of insertion-deletion sites. In this study, we employed two methods to conduct a comprehensive genome-wide analysis and successfully identified stable insertion-deletion sites (results are shown in the Venn diagram). As in our previous study, we found that the male and female chromosomes of O. fasciatus differed and that the males underwent a chromosome fusion event. Moreover, the male fusion chromosome (MChr9) showed significant structural variation compared to its homologous chromosomes (FChr8 and FChr10 in females). Therefore, we focused on the differences between male and female homologous chromosomes that underwent chromosomal structural variation. In order to avoid the influence of the structural dimorphism of the male chromosome on the exploitation of sex markers, we focused on the detection and validation of large segment deletion sites by using the male genome as the reference genome, and by replicating the long segment CLR reads obtained from females to the reference genome of males.<br>We obtained deletion sites for 4675 (female genome as reference) and 4385 (male genome as reference) large segments based on structural variation scans (1~1,000,000bp) at the chromosome level. At the same time, we performed a large

segment (≥100bp) variant site scan of the male and female assembled genomes using SYRI software, and obtained 2116 (female genome as reference) and 2045 (male genome as reference) variant sites, respectively, and 84.64% and 85.53% of the variant sites intersected with the variant catalogs obtained by us, respectively. In order to further improve the exploitation efficiency of sex-specific markers based on long reads, we first calculated the read depth of long reads of insertion deletion sites, and then ranked the insertion deletion site markers based on the depth of long reads, for large segment deletion variants, the lower the depth of long reads, the more effective the markers are, so we constructed a frequency map of the distribution of the depth of long reads of the large segment deletion sites of the male and female genomes, and then classified the markers. We found that long read depths less than 5.48 (895 markers) and 12.50 (1,021 markers) belonged to high-recognition sex markers, using the male and female genomes as references, respectively.
As we have known, stable variant loci are the basis for the exploitation of genetic sex markers, but effective amplification is fundamental for the application of sex markers. In order to validate high-throughput markers for male and female sex and to assist conventional agarose gel electrophoresis PCR, we introduced an electronic simulation of PCR method (e-PCR) for high-throughput amplification of variant markers. Based on this method, on the one hand, we can quickly identify the loci that can be amplified by a single PCR strip, and on the other hand, this method can assist us in the design of more effective primers for the target variant loci, and then quickly carry out the agarose gel electrophoresis PCR assay for the development of markers for both male and female genetic sexes. We targeted the fusion chromosome (MChr9) of male fish and the homologous chromosome (FChr8 and FChr10) variant loci of its female fish for marker validation and selection, and selected a total of 17 loci located in the intergenic region, intronic region and other typical markers for testing, and successfully completed the validation of the male and female of 11 loci. Meanwhile, we found that the insertion deletion sites are stable, but the non-specific amplification caused by the primers is often an unfavorable factor affecting the subsequent development of sex markers, so how to improve the specificity of the amplification primers and adjust the conditions of the PCR reaction (using touch down method) are the key to the development of markers after the need.
In the manuscript's abstract, there are numerous undefined abbreviations and technical terms. Authors should revise the abstract to minimize the use of jargon. For example:
Line 36-37: "first-generation development methods such as RFLP, 37 RAPD, SSR, and AFLP as well as second-generation techniques utilizing Illumina's 38 SNP/InDel markers."
It is confusing molecular biology techniqies with next generation sequencing (NGS) techniques.
Line 45: "Illumina survey" can be revised "short-read sequencing data survey"
Line 46: "PacBio CLR / CCS" can be revised "long-read PacBio sequencing"
Reply:
We are very grateful to you for your professional advice, which made us realize that biotechnology and sequencing technology are two different concepts, although they are closely related. Therefore, we have reorganized and rewritten the abstract according to your suggestions as follows:
"Background
The use of sex-specific molecular markers has become a prominent method in enhancing fish production and economic value, as well as providing a foundation for understanding the complex molecular mechanisms involved in fish sex determination. Over the past decades, research on male and female sex identification has predominantly employed molecular biology methodologies such as RFLP (Restriction Fragment Length Polymorphism), RAPD (Random Amplification of Polymorphic DNA), SSR (Simple Sequence Repeat), and AFLP (Amplified Fragment Length Polymorphism). The emergence of high-throughput sequencing technologies, particularly Illumina, has led to the utilization of SNP and InDel variants as significant molecular markers for investigating sex identification in fish. The advancement of sex-controlled breeding encounters numerous challenges, including the inefficiency of current methods, intricate experimental protocols, high costs of development, elevated rates of false positives, marker instability, and cumbersome field testing procedures. Nevertheless, the emergence and swift progress of PacBio high-throughput sequencing technology, characterized by its long read output capabilities, offers novel opportunities to overcome these obstacles."
We also appreciate your wonderful suggestions. First, we revised "Illumina survey" and

Powered by Editorial Manager® and ProduXion Manager® from Aries Systems Corporation

	"PacBio CLR / CCS" as "short-read sequencing data survey" and "long-read PacBio sequencing", respectively, as follows:<br>"Utilizing male-female linear genomic information in conjunction with short-read sequencing data survey and long-read PacBio sequencing data, a catalog of large-segment (>100 bp) insertion-deletion genetic variants was generated through a genome-wide variant site scanning approach with bidirectional comparisons. The sequence tagging sites (STS) were ranked based on the long read depth of the insertion deletion site, with markers exhibiting lower long read depth being considered more effective for large segment deletion variants. Subsequently, a catalog of bulk primers and simulated PCR for the male-female variant loci was developed, incorporating primer design for the target region and e-PCR technology. The criteria for rapid identification of male and female differences in Oplegnathus fasciatus were exploited through agarose gel electrophoresis, which revealed two amplified bands for males and one amplified band for females. A high-throughput identification catalog of sex-specific markers was then constructed using this method, resulting in the identification of 3,645 and 3,872 markers in conjunction with 1,021 and 894 high-quality genetic sex identification markers, respectively. Sixteen differential loci were randomly chosen from the catalog for validation, with 11 of them meeting the criteria for male-female distinctions. The implementation of cost-effective and efficient technological processes within the establishment will facilitate the rapid advancement of genetic breeding through expediting the high-throughput development of sex genetic markers for various species."<br>"Conclusions<br>Our study utilized linear genomic information from male and female individuals obtained from PacBio, in addition to data from short-read sequencing data survey and long-read PacBio sequencing. We extensively employed genome-wide variant site scanning and identification, high-throughput primer design of target regions, and e-PCR batch amplification, along with statistical analysis and ranking of the long reads depth of the variant sites. Through this integrated approach, we successfully compiled a catalog of large insertion/deletion sites (>100 bp) in both male and female O. fasciatus."<br>On this basis, we revised the manuscript from "Illumina survey and PacBio CLR/CCS data" to "short-read sequencing data survey" and "long-read PacBio sequencing" as follows:<br>1) Line 318-320, we revised "we utilized the male Illumina survey data and PacBio CLR data for read assignment and further confirmed that this candidate variant site was a valid insertion site" as "we utilized the male short-read sequencing data and long-read PacBio sequencing data for read assignment and further confirmed that this candidate variant site was a valid insertion site".<br>2) Line 338-340, we revised "A comparison of sequence differences, Illumina survey data and PacBio CLR data read assignments all confirmed that the genetic differences between male and female markers were mainly concentrated in the.." as "A comparison of sequence differences, short-read sequencing data and long-read PacBio sequencing data assignments all confirmed that the genetic differences between male and female markers were mainly concentrated in the….".<br>3) Line 449-452, we revised "along with data from Illumina surveys and PacBio CLR/CCS, we extensively employed whole-genome variant site scanning and identification, high-throughput design of primers for the target regions, and e-PCR batch amplification.." as "along with data from the short-read sequencing data and long-read PacBio sequencing data, we extensively employed whole-genome variant site scanning and identification, high-throughput design of primers for the target regions, and e-PCR batch amplification…"<br>The methods described in this manuscript is not clear and missing details. how many samples were sequenced using Illumina and PacBio? How was the sequencing data preprocessed for QC, poor reads filtering and adapter trimming? What source, database and accession ID was the reference genomes of male and female downloaded? It is mentioned that aligner BWA (v 0.7.8) and Minimap2 were used, but as could not follow which aligner map Illumina or PacBio reads?<br>Reply:<br>We are very grateful to your constructive comments, and we have added the sample sources of the genomes and the quality evaluation parameters of the high-throughput sequencing data as follows:<br>Table 1. Parameters for Illumina and PacBio high-throughput sequencing and sequencing quality assessment of male and female O. fasciatus assembled genomes

Powered by Editorial Manager® and ProduXion Manager® from Aries Systems Corporation

	"Sample collection, sequencing and genome quality<br>Male and female O. fasciatus captured from Qingdao, Shandong Province (Yellow Sea) were utilized for genome sequencing and assembly. High-quality genomic DNA was extracted from fresh muscle tissue and blood samples from pairs of male and female O. fasciatus using the Illumina platform (Illumina Inc., San Diego, CA, USA) and PacBio Sequel (Pacific Biosciences of California, Menlo Park, CA, USA). Genomic DNA from fresh muscle tissue and blood samples of male and female O. fasciatus pairs was extracted to construct short insert libraries (300~350 bp) for males and females, respectively, using standard protocols provided by the Illumina HiSeq X Ten platform (Illumina Inc., San Diego, CA, USA). Raw reads totaling approximately 90.7 Gb and 63.9 Gb were generated. After removing splice-containing sequences, low-quality, and redundant reads, approximately 80.8 Gb and 56.6 Gb of pure data were obtained from the genomes of males and females, respectively, corresponding to Q20 averages of 97.55% and 97.60%, respectively. Genomic DNA libraries (20 kb) for male and female fish were then constructed using the PacBio Sequel platform (Pacific Biosciences of California, Menlo Park, CA, USA). Sequencing was performed on the PacBio Sequel platform using the Sequel Binding Kit 2.0, Sequel Sequencing Kit 2.1, and Sequel SMRT Cell 1M v2. Seven and five SMRT Cells were used for sequencing the male and female genomes, respectively, with SMRT LINK 5.0 used to filter the raw data from the zero-mode waveguide. We obtained 62.8Gb and 39.8Gb of long read length PacBio sequencing data for female (accession SRP160016) and male (accession SRP220007) genome assembly, respectively. The female and male O. fasciatus genome were de novo assembled using Canu v1.4 with Corrected-Error-Rate set to 0.040, followed by redundancy removal using Redundans v0.13c (minCoverage = 15), genome polishing with Arrow tool in SMRT Link 5.0, and final assembly refinement with Pilon using Illumina NGS short reads from the genome surveys. Based on the male and female draft genomes and Hi-C technology, we obtained high-quality genomes at the chromosome level of female (768.8Mb, accession SUB14064808) and male (762.2Mb, accession SUB14065793) O. fasciatus, and the contig N50 and scaffold N50 of the male and female fish genomes reached 2.13 Mb/2.18 Mb and 33.5 Mb/32.4 Mb, respectively. To assess the genomic completeness of assembled male and female O. fasciatus, we subjected the assembled sequences to BUSCO version 5 evaluation (BUSCO, actinopterygii_odb10), with Complete BUSCOs assessed values up to 98.2% and 98.0% for male and female genomes, respectively."<br>One of my main concerns is the simulated e-PCR. I am not sure, how much this technique is reliable for validation studies and what is the rate of false positive/negative relative to the actual experimental PCR technique. My question to authors, Are, there any previous studied that have utilized the e-PCR amplified fragment sizes for genome variants validation? If yes, it is recommended to cite with brief description of reliability of this e-PCR versus normal PCR in lab.<br>Reply:<br>Thanks to your meticulous suggestions, as you mentioned e-PCR is indeed a very interesting tool, we checked the development of e-PCR, single sequence tagged site (STS) was proposed by Olson et al in 1989, and later Schuler Gregory D. Later, in Rotmistrovsky et al (2004), this technique was developed and published in the Nucleic Acids Research, which allowed the use of e-PCR with a single primer on the web, specifically for the Human Genome UCSC website database. As envisioned by Schuler Gregory D. (1997), the proponent of e-PCR, the advantage of e-PCR is that STS loci can be identified without any experimentation, which will be important for modern genomics research. The limitations of computer language, limited genomic data and the amplification of a single STS have not allowed the proposed rapid visualisation, making the technical application of e-PCR limited.<br>Gregory D. Schuler. (1997) Sequence Mapping by Electronic PCR, Genome Research, 7:541–550.<br>Kirill Rotmistrovsky, Wonhee Jang and Gregory D. Schuler. (2004) A web server for performing electronic PCR. Nucleic Acids Research, 32: W108-W112.<br>With the development of high-throughput sequencing technology and the improvement of genome analysis methods, the flux identification of sequence tagged sites (STSs) has increased dramatically, and e-PCR prediction of individual STSs as well as conventional PCR assays are not capable of timely validation, which limits the high-throughput identification of variant markers and high-throughput development. Therefore, we integrated high-throughput identification of large insertion-deletion sequence marker sites, high-throughput design of e-PCR primers for STS, and high-

throughput amplification and validation of e-PCR primers for STS to realize high-throughput development and validation of male and female genetic (STS) markers based on male and female genome data, and accelerate the process of fish genetic breeding. Let's take a case study to illustrate efficient visualization of single and high-throughput sequence tagged sites.

Female genome STS

GGACCGGGGCAAGGGACCCACACAATCGATGATCAAGTGTTCAAAAGGCTGTCC
AATTGCAGGGATTGGATGCAAGGGAGCAGGTTTAACAACTTGATTAGGCTTGCCA
GTGATCTGACAGGTATGGCATGTTTTGATGAAGAGAGCGACGTTGCATGGCCAGA
AGAAATATTGCAGTATACGATGGTAGGTTTTATGGATGCCAAGATGACCTGCCACA
TCACCATGTGACGTCTGGAGTACTTTACTGCGACAGTTGGTAGGAACAACAACTTT
AAAAATTGGCTCTCCTACAAACTGTTCACCATGGGGCATTCACTTTCTAACCAGGT
TACTGGTTAGAAAGTGAAAGCCTCCCTGCACTCAGATGTTAG

Male genome STS

GGACCGGGGCAAGGGACCCACACAATCGATGATCAAGTGTTCAAAAGGCTGTCC
AATTGCAGGGATTGGATGCAAGGGAGCAGGTTTAACAACTTGATTAGGCTTGCCA
GTGATCTGACAGGTATGGCATGTTTTGATGAAGAGAGCGACGTCGCGTGGCCAG
AAGAAATAATGCAGTATACGATGGTAGGTTTTATGGATGCCAAGATGACCTGCCA
CATCACCATGTGACGTCTGGAGTACTTTACTGCGACATTTGGTAGGAACAACAACT
TTTGTTGGCTCTCCTACAAACTGTTCACCATGGGGCATTCACTTTCTAACCAGGTT
ACTGGTTAGAAAGTGAATGCCCCATGGTGAACAGTTCTAGGAGGAAATACCACTG
AGCATTATTTCTCACCTCTGTCGCTGGCACAACTCTGTCAAATAGCTCAGTTAAGG
AAGAATCTGCCTTCTGCTCTGCCACTAACTCAGCCTGAGAACCTGCTAGCTGGTC
AACTGGCAATGAAACTGGCAACTCCAAATACTCAGGCTCCACCACATCCTGTGCG
GCATGGCTGGCAGCACGGGTGACAGCACAGGCTGGAAAAGTGTCATCCAGTGAC
CTCTCTGCGGGTGCTACCACAGTGGGAGACGTTGGCTGCTTCACTACATTTGGCA
CGCCATCGGCCCACACCTTGTCCCCAGCCAAATCATTCCCCAAAATCATATGCAC
CCCTTCAACAGGCAACTGGGGACGGACACCAACTGACACTTCACCCTTTACCACA
AGTGAGACTCACTTGGTGCAAAGGAACAGATAATGGAACCAAACCCATTCCTAGA
ATGATTACACACCCACCAGTATCAGTTTTGGATGAAAATGGCAACATAGACTGAAA
GAATCCAACGCACCAGTGTCTCTTAGAATTTTAATGGGAACCTGCACACTACTCCC
CACCAATGAGACCAACCCATCCGATTTGAAGGCAGTTTGATCAGCTCGCAGTGAC
GCCAGGTCAGTATGTGACTGCAACTGACCAGCCTCCCTGCACTCAGATGTTAG

On this basis, STS high-throughput e-PCR amplification detection was realized by using high-throughput scanning of sequence tagged sites combined with e-PCR method. High-throughput e-PCR amplification not only allows rapid detection of the amplification efficiency of STS loci, but also provides an aid and reference for primer design for subsequent conventional PCR.

High-throughput e-PCR amplification assay for STS

The gene otology enrichment findings seems to be out of context from the scope of the manuscript. The authors described the enriched GO terms for the exonic variants detected in their analysis, but there is no discussions for the meaningful biology of these functions and most of these results are just descriptive reports without further mechanisms and consequences explanation.

Reply:

We appreciate your constructive comments, and based on your and the other reviewers' common suggestions, which we agreed by deliberation were extremely professional, we have removed that section from the manuscript.

The figures captions are not clearly described to explain the data. For example, in figure 3,

Line 251-252, what is meaning of different alignments patterns in dotplots? All the captions should be explanatory for the data plotted in figures.

Reply:

Thanks to your professional advice, We have organized all the images as requested the results are explained below

Figure 2: Distribution statistics of insertion and deletion fragment (> 100bp) positions in genomic functional regions of O. fasciatus.

(a) Distribution statistics of insertion variant loci using the female fish genome as a reference. (b) Distribution statistics of insertion variant loci utilizing the male fish genome as a point of reference. (c) Distribution statistics of deletion variant length

Powered by Editorial Manager® and ProduXion Manager® from Aries Systems Corporation

using the female fish genome as a reference. (d) Distribution statistics of deletion variant loci using the male fish genome as a reference.
Note: The colors red, green, yellow, and orange represented the UTR, CDS, intron, and intergenic regions. The frequency of insertions (a, b) was quantified based on the number of events, while deletions (c, d) were characterized by their length in nucleotides.

Figure 3: The covariance of genomic sequence differences between the male and female genomes of O. fasciatus and distribution statistics of variant sites.
(a) The female genome (reference) of O. fasciatus vs. the male genome for homology comparison using the minimap2 software with a 5000 bp sliding window. The female genome was the vertical axis, and female chromosomes 8 and 10 were homologous to male chromosome 9. (b) The comparison of homology between the male genome of O. fasciatus and the female genome was conducted using the minimap2 software with a 5000 bp sliding window. The male genome was represented on the vertical axis, revealing homology between male chromosome 9 and female chromosomes 8 and 10. (c) Distribution statistics of variant loci using the female fish genome as a reference. Insertions (>100 bp) were summarized by number of events. Deletions (>100 bp) were summarized by length in nucleotides. From outer to inner circles were the chromosome name, positional distribution of insertion sites, base length distribution of deletion sites, and base substitution distribution, respectively. (d) Distribution statistics of variant loci using the male fish genome as a reference. (e) - (f) Dual-valued histograms corresponding to the Table 3 showed the number of insertion/deletion sites per chromosome with reference to the female (4,422 inertion sites, 4,675 deletion sites) and male (4,615 inertion sites, 4,385 deletion sites) genomes, respectively, where chromosome 9 in males was homologous to chromosomes 8 and 10 in females. Insertion sites were represented by negative values, and deletion sites were represented by positive values..

Figure 4: Intersection results of two variant site scanning methods and ranking of variant sites with long read depth frequency distributions
(a) Venn results of deletion locus data were obtained based on TBtools and SyRI software using the female fish genome as a reference, and 1,791 out of 2,116 deletion loci obtained based on SyRI software were shared deletion variant loci. (b) Utilizing the male fish genome as a point of reference for acquiring deletion site data in the context of Venn analysis, it was found that 1,749 out of the 2,045 deletion sites identified through the use of the SyRI software were common deletion variant sites. (c) Depth distribution of CLR reads in male fish using all female genomic deletion locus (＞100bp) information as a reference, with the area where high-quality male and female genetic marker information was located before the orange labeled trough position (~5.4). (d) The depth distribution of CLR reads in females was analyzed by utilizing male genomic deletion sites as a reference (>100bp), focusing on the region preceding the orange labeled trough position (~12.5) where high-quality male and female genetic marker information was present. (e) - (f) Depth distribution of CLR reads in males and females based on intersecting male and female genomic deletion site information (>100bp), respectively.

Figure 5: Representative genetic sex marker (insertion deletion variant sites) located in the intergenic region (IR), Intronic region (IN) of the O. fasciatus genome (STOSOd393e).
(a) Location of male and female sex markers in the male (MChr9) and female (FChr8) genomes. (b) Male and female sex genetic markers were located at loci in the intergenic region, using the female fish genome as a reference (Figure S2). Illumina and CLR clean data of bam comparison to the genome for insertion/deletion site validity detection. Red represented length long data from PacBio and gray represented was Illumina clean data. The e-PCR amplified bands were 390bp and 989bp in size, corresponding to DNA insertion and deletion patterns and agarose gel electrophoresis for identification of male and female genetically different populations. (c) By utilizing the female genome as a point of comparison, the genetic markers for male and female sexes were identified at specific insertion sites within nuf2 gene introns (Figure S3, Figure S4). The resulting e-PCR amplified bands, measuring 205bp and 749bp in size, were indicative of distinct DNA insertion patterns and were utilized in agarose gel electrophoresis for the purpose of distinguishing between male and female genetically differentiated groups. Males exhibited two amplified bands, while females exhibited

Powered by Editorial Manager® and ProduXion Manager® from Aries Systems Corporation

only one. (d) By utilizing the female fish genome as a point of comparison, the genetic markers for male and female sexes were situated within the deletion sites located in the introns of genes (Figure S5, Figure S6). The amplified bands produced through e-PCR were observed to be 558bp and 928bp in length, aligning with the DNA deletion patterns and agarose gel electrophoresis maps utilized for distinguishing genetically distinct groups of males and females. Specifically, two bands were amplified in males, while only one band was observed in females.

Some figure (Figure 7), have unnecessary portion with alignment browser screen, it should be cropped or manually edited for better visualization.
Reply:
Thanks to your detailed advice, we cropped the resultant display image (Figure 7) after it appeared to be unnecessary when aligning the browser screen. At the same time, we processed all the other results that had similar problems.

Figure 7

Figure 5

Minor comments:
The use of term "linear genomic information" is not very clear. Authors should clearly explain what they mean by linear genome? Whether this term mean genome assembly or comparative genomic analysis?
Reply:
Thanks to your professional advice, as you said the linear genome information was unclear and what they actually meant was the assembled genome, therefore, we have changed all the linear genome to assembled genome information in the manuscript according to your suggestion as follows
1) Line 197-198, we revised “sequencing technologies has made it possible to exploit high-throughput sex-specific markers in high-precision linear genomic data.” as “sequencing technologies has made it possible to exploit high-throughput sex-specific markers in high-precision assembled genome data.”.
2) Line 219-220, we revised “constructed based on the linear genomic information of O. fasciatus by using bidirectional whole-genome sequence alignment..” as “constructed based on the assembled genome information of O. fasciatus by using bidirectional whole-genome sequence alignment”.
3) Line 231-232, we revised “First, a fast scanning method for female and male linear genomic variant loci was introduced…..” as “First, a fast scanning method for female and male assembled genome variant loci was introduced……”.
4) Line 598-599, we revised “By utilizing the linear genomic information of male and female individuals acquired through PacBio sequencing,” as “By utilizing the assembled genome information of male and female individuals acquired through PacBio sequencing,”.

On Line 147, "Using the difference database" should be corrected "Using different databases"
Reply:
Thanks to your detailed advice, we revised “Using the difference database,” as “Using the difference catalogs,”

Reviewer #2: Dear colleagues,

Than you very much for giving me the opportunity to read and comment the article of Xiao et al., entitled "A novel and scalable method for high-throughput and accurate exploitation of sex-specific markers: a case study on Oplegnathus fasciatus".
Most Eukaryotes do sex, and pluricellular organisms have highly differentiated organs, using sex determination systems and sex differentiation genes. In mammals, sex chromosomes are highly dimorphic, while most fish genomes show no distinction of their sexual chromosomes, and the genes responsible for the sex determination have been hard to characterize, sometimes being located in regions full of repeated sequences. Also, if sexual organs are easy to visualize and are used for chick sexing

Powered by Editorial Manager® and ProduXion Manager® from Aries Systems Corporation

at just one day old in the egg industry (male chicks are culled as they are an unwanted by-product of egg production), such a selection can be harder to do in fish. Sexing the striped beakfish (Oplegnathus fascinatus), a major in Asian fish farm industry, is less easy, and the sexual differentiation can only be observed once the animals have passed the 500 g weight size, when males start to grow faster that females, bringing better yields. Among the different methods and technics used to
sex the individuals in the past, none were really conclusive. The authors propose to take advantage of the long reads provided with the third generation sequencing to access a class of mutations that are hundred-bp long insertions and deletions, not taken much into consideration before then. They were able to find occurrences of long indels that could be used as proxies of genetic sex markers.
In this work, the authors do present convincing technics and results. However, in that framework, it can be expected that the authors will also provide the weaknesses of this technic. Here, I see at least two points that should be considered. The first one is related to population genetics and the second should deal with the origin, if any, of these deletions and insertions, and their stability.
Starting with the second, what is the origin of such insertions and deletions? Could it be due to the mobilisation of a transposable element? Or any hot spot of deletion due for example to the formation of a fragile non-conventional B-DNA? Is there any repetitive sequence? Could these deletions or insertions occur frequently?
Reply:
We are very grateful to you for your constructive comments, which led us to think deeply and explore the origin of insertion deletion sites in large segments as well as the drivers, which would facilitate the extension and progression of O. punctatus from structural genomics to functional genomics. Therefore, we carefully examined the your suggestions and added variant site transposons and tandem repeat systematic analyses as follows:
"To identify the origin of large segment insertion deletion sites, we evaluated whether the variant sites in the dataset were transposons by integrating RepeatModeler (v 2.0.1) and RepeatMasker (v 4.1.2) softwares. The results of transposon determination obtained by the two softwares were combined and the duplicates were removed for all transposon identification results. At the same time, we utilized TRF (v 4.09.1) software to evaluate whether the variant sites in the dataset were tandem repeats." Since deletion variants in large segments can more clearly show the differences between males and females, especially the read-return effect of the long read-length information of females with the male genome as the reference was more effective, we counted the sequence tagging sites (STS) attributed to transposons or tandem repeats of large segment deletions of males and females with the male and male genomes as the references, respectively.
Table 4 Statistics of transposons and tandem repeat sequences attributed to large deletion sequence tag sites obtained with male and female reference genomes
The results showed that 78.44% (3667) of the deletion sequence tag sites with female as reference belonged to transposons and 33.67% (1574) of the STSs belonged to tandem repeat sequences. Meanwhile, the statistical results showed that 3380 (77.08%) of the missing sequence tag sites with male fish as reference belonged to transposons and 1399 (31.90%) of the STSs belonged to tandem repeats. As you predicted, at present, more than 77% of the large-band deletion sequence tag sites obtained from both female and male fish genomes as references are from transposons. The widespread presence of transposons in animal genomes and their important roles in the evolution of animal genomes, transposons can cause genome and chromosome evolution in a variety of ways, including insertion-induced gene mutations, gene transposons can cause genome and chromosome evolution in many ways, including insertion-induced gene mutation, gene backward movement, chromosome recombination, gene duplication and providing pivot points for ectopic recombination, etc., which is of great significance to adaptive gene evolution.
"The findings further indicated that a substantial proportion of deletion sequence tag sites referencing the female genome were situated in transposon regions (78.44%, n=3,667) and tandem repeat sequences regions (33.67%, n=1,574). Similarly, statistical analysis revealed that a notable percentage of deletion sequence tag sites referencing the male genome were linked to transposon regions (77.08%, n=3,380) and tandem repeat regions (31.90%, n=1,399) (Table 4)."

The RepeatModeler (v 2.0.1), RepeatMasker (v 4.1.2) and TRF (v 4.09.1) softwares run the following scripts:

Powered by Editorial Manager® and ProduXion Manager® from Aries Systems Corporation

RepeatModeler (v 2.0.1)
1) RepeatModeler-2.0.1/BuildDatabase -name mydb genome.fa > assembly_final.fasta.repeatmodeler.log
2) RepeatModeler-2.0.1/RepeatModeler -database mydb -pa 6 > run.out
3) mv ./RM_* ./result
4) cat result/consensi.fa.classified > final.library
RepeatMasker (v 4.1.2)
1) RepeatMasker-4.1.2/RepeatMasker/RepeatMasker -nolow -no_is -norna -parallel 2 -lib RepeatMasker-4.1.2/RepeatMasker/Libraries/RepeatMasker.lib genome.fa
2) RepeatMasker-4.1.2/RepeatMasker/RepeatMasker -nolow -no_is -norna -parallel 2 -lib final.library genome.fa
TRF (v 4.09.1)
1) trf-v4.09.1/trf genome.fa 2 7 7 80 10 50 2000 -d -h

The first question deals with the level of polymorphism of these indels. How frequent are these insertions/deletions comparing different geographic populations?
I understand that these two points are not the main interest of the present article. However, these evolutionary considerations would deserve to be taken into account, at least correctly addressed in the discussion.
Reply:
Thanks to your professional advice, this issue made us think seriously and deeply, on the basis of which we extended the results of the seven markers (four stable) that had been validated in the original manuscript to validate the new nine genetic sex markers (six stable) in different geographic populations, and the population samples used were from the Zhoushan population and the Jiaonan population, and the results indicated that although the samples were from different geographic populations, large segments of the deletion sequence tag loci were stable across geographic populations with male and female differences as follows.

Validation of long read deletion sites for geographic populations using the male genome as a reference
As you mentioned large segments of insertion deletions are worth considering in terms of evolutionary considerations, especially since we found that most of the deletion sites belonged to transposons, which was the source of genetic diversity of the species and the origin of adaptive variation, so we reviewed the insertion deletion cases and discussed them in the Discussion section as follows:
“Variation in genome structure was intricately linked to human diseases, evolution, gene regulation, and species phenotypes in the biological realm, making it a prominent area of study in genomics research [79]. Previous studies have shown that DNA sequences with insertions and deletions undergo increased selective pressure [80-84]. The presence of deletions and insertions in DNA sequences, regardless of their location in noncoding or coding regions, inevitably influences the functionality of the original sequence to varying extents [79]. Furthermore, evidence suggested that the length and placement of insertions were not random but rather heavily influenced by the adjacent DNA sequence [80-82, 85, 86]. Prior research has indicated that insertion deletion variants accounted for 16%, 24%, 13%, 25%, and 15% of genomic genetic polymorphisms in various organisms, including Drosophila melanogaster, Caenorhabditis elegans, mouse, human, and Arabidopsis thaliana [34-36, 87-94]. Recent advancements in long length sequencing technology have allowed for a more precise examination of human genetic variation, revealing that insertions and deletions collectively contribute to a high portion (13.8%, 11.1%) of genetic polymorphisms in the human genome [90-94]. The focus of genomic structural variation studies has predominantly been on single nucleotide polymorphisms (SNPs) and insertions/deletions (InDels) of less than 50 base pairs due to the limitations of high-throughput sequencing technologies for short read lengths. However, larger genomic structural variants, which encompassed genetic variations not fully captured by SNPs and InDels, were closely linked to biological phenotypes and might significantly influence phenotypic traits, disease susceptibility, and adaptive capacity [79]. The utilization of long-read length sequencing technologies such as PacBio and ONT has enabled the generation of extended DNA sequences, thereby enhancing the assembly of diploid genomes of superior quality and the identification of structural variants (SVs) across substantial genomic regions [95]. Prvious research has documented seven deletions (>100bp) within approximately 700bp large segments of the ZmBAM1d gene, demonstrating a positive association with maize kernel weight. These deletions were

	consistently inherited in high-yielding maize lines [96]. In mammalian studies, a deletion of approximately 40Mb on chimpanzee chromosome 2, as compared to humans and gorillas within the great ape family, has been identified, leading to stable genetic distinctions among species [97]. Similar results have been documented in Astyanax mexicanus, Gasterosteus aculeatus, racehorses, and dogs [98-101]. The emergence of long read-length sequencing technology has propelled large fragment genomic structural variation to the forefront of genetic variation research, particularly in investigations of species' adaptive evolution [95, 96, 102, 103]. The findings of this study indicated that insertions and deletions identified using the female and male O. fasciatus genomes as references constituted 26.96% and 26.97% of the genetic polymorphisms, respectively. Our research has contributed new insights into the prevalence of large-segment insertion-deletion events for O. fasciatus, indicating that these events predominantly occurred in the intergenic region in 58.09%~66.33% of instances, with the intronic region following closely at 31.65%~40.35% of cases (Figure 2, Figure S1 Table 2). We also observed a higher proportion of deletion sites compared to insertion sites. A similar study conducted on the β-fibrinogen intron of birds reported similar findings [104]. Furthermore, this study presented novel findings indicating that over 77% of the identified large deletion sequence tag sites in the genomes of both female and male O. fasciatus were attributed to transposons (Table 4). It was evident that transposons were prevalent in the animal genome and significantly contribute to its evolutionary processes. Previous research has demonstrated that a significant portion (69.5%) of genomic structural variation in large segments of tomato was situated within the transposon region, which was closely linked to desirable traits in tomato [105]. Comparable studies have also been conducted on gene regulation in human brain diseases and genomic structural variation in evolutionary distinct phenotypes of great apes [103, 106]. Transposons have the potential to drive genome and chromosome evolution through various mechanisms such as insertion-induced gene variation, gene retrotransposition, chromosome recombination, gene duplication, and serving as sites for ectopic recombination. These findings underscored the importance of transposons in facilitating adaptive evolution for O. fasciatus. The presence of large fragments of insertion deletion sequence marker sites was frequently influenced by natural selection pressures, with their formation being intricately linked to adjacent DNA sequences and commonly correlated with characteristic phenotypic traits and aberrant gene regulation [83, 84, 102, 103, 105, 106]. Specifically, the manifestation of albino symptoms in dogs resulting from a 965-kb deletion on chromosome 18 exhibited stable inheritance patterns, while a 4.8 Mb deletion variant on the chromosome of male domestic chickens similarly displayed heritability across subsequent generations [100, 107]. Consistent inheritance of variants within populations has also been documented in academic research on maize (ZmBAM1d), Astyanax mexicanus, Ailur...
**Additional Information:**	
**Question**	**Response**
Are you submitting this manuscript to a special series or article collection?	No
**Experimental design and statistics**<br><br>Full details of the experimental design and statistical methods used should be given in the Methods section, as detailed in our Minimum Standards Reporting Checklist. Information essential to interpreting the data presented should be made available in the figure legends.<br><br>Have you included all the information requested in your manuscript?	Yes

**Resources**<br><br>A description of all resources used, including antibodies, cell lines, animals and software tools, with enough information to allow them to be uniquely identified, should be included in the Methods section. Authors are strongly encouraged to cite Research Resource Identifiers (RRIDs) for antibodies, model organisms and tools, where possible.<br><br>Have you included the information requested as detailed in our Minimum Standards Reporting Checklist?	Yes
**Availability of data and materials**<br><br>All datasets and code on which the conclusions of the paper rely must be either included in your submission or deposited in publicly available repositories (where available and ethically appropriate), referencing such data using a unique identifier in the references and in the "Availability of Data and Materials" section of your manuscript.<br><br>Have you have met the above requirement as detailed in our Minimum Standards Reporting Checklist?	Yes

Reply point-to-point Click here to access/download;Manuscript;Reply point-to-point.docx

GIGA-D-23-00373
A novel and scalable method for high-throughput and accurate exploitation of sex-specific markers: a case study on Oplegnathus fasciatus
Yongshuang Xiao; Zhizhong Xiao; Daoyuan Ma; Lin Liu; Jing Liu; Jun Li
GigaScience

Dear Prof Xiao,

Your manuscript "A novel and scalable method for high-throughput and accurate exploitation of sex-specific markers: a case study on Oplegnathus fasciatus" (GIGA-D-23-00373) has been assessed by our reviewers. Although it is of interest, we are unable to consider it for publication in its current form. The reviewers have raised a number of points which we believe would improve the manuscript and may allow a revised version to be published in GigaScience.

Their reports, together with any other comments, are below. Please also take a moment to check our website at https://www.editorialmanager.com/giga/ for any additional comments that were saved as attachments.

In addition, please register any new software application in the bio.tools and SciCrunch.org databases to receive RRID (Research Resource Identification Initiative ID) and biotoolsID identifiers, and include these in your manuscript. Computational workflows should be registered in workflowhub.eu and the DOIs cited in the relevant places in the manuscript. These will facilitate tracking, reproducibility and re-use of your tool.

If you are able to fully address these points, we would encourage you to submit a revised manuscript to GigaScience. Once you have made the necessary corrections, please submit online at:

https://www.editorialmanager.com/giga/

If you have forgotten your username or password please use the "Send Login Details" link to get your login information. For security reasons, your password will be reset.

Please include a point-by-point within the 'Response to Reviewers' box in the submission system. Please ensure you describe additional experiments that were carried out and include a detailed rebuttal of any criticisms or requested revisions that you disagreed with. Please also ensure that your revised manuscript conforms to the journal style, which can be found in the Instructions for Authors on the journal homepage. If the data and code has been modified in the revision process please be sure to update the public versions of this too.

The due date for submitting the revised version of your article is 29 Apr 2024.

I look forward to receiving your revised manuscript soon.

Best wishes,

Hongfang Zhang
GigaScience
www.gigasciencejournal.com

Reviewer reports:

Reviewer #1:

In this manuscript, Xiao et al performed bioinformatic analysis of Illumina and PacBio sequencing data to detect insertion/deletion variants, coupled with PCR based experimental validation to enable identification of genetic sex markers in a fish species. Overall, the manuscript main strength is the findings of sex specific markers in both male and female genomes as clearly supported by PCR results. But there are still a few shortcomings that needs to be addressed. Below I explain my major points:

In this manuscript, the authors assert that they have created a database of insertion/deletion-based sex-markers. After reviewing, it appears that this claim may be overselling their findings. In bioinformatics, the term "database" typically refers to an online portal for users to explore data. Therefore, it is suggested to consider revising the term "database" to "variants catalog" for clarity and accuracy.

Reply:

By carefully reading the reviewer' comments, we realized that we had indeed obtained a catalog rather than a database of male and female sex markers for *Oplegnathus fasciatus*, which was a conceptual error. We would like to thank the reviewer for his professional advice. Based on the reviewer's comments, we have revised all the technical procedures and database-related contents of the paper into catalogs, and the list of changes is as follows:

1. We have changed "Datasets" in "All Chromosome variants Dataset", "Bulk Primer Design for Variant Datasets" and "Batch e-PCR of Variant Datasets" to **"All Chromosome variants catalog", " Bulk Primer Design for Variant catalog ", and “Batch e-PCR of Variant catalog” respectively, in the technology flow. Please see the figure below.**

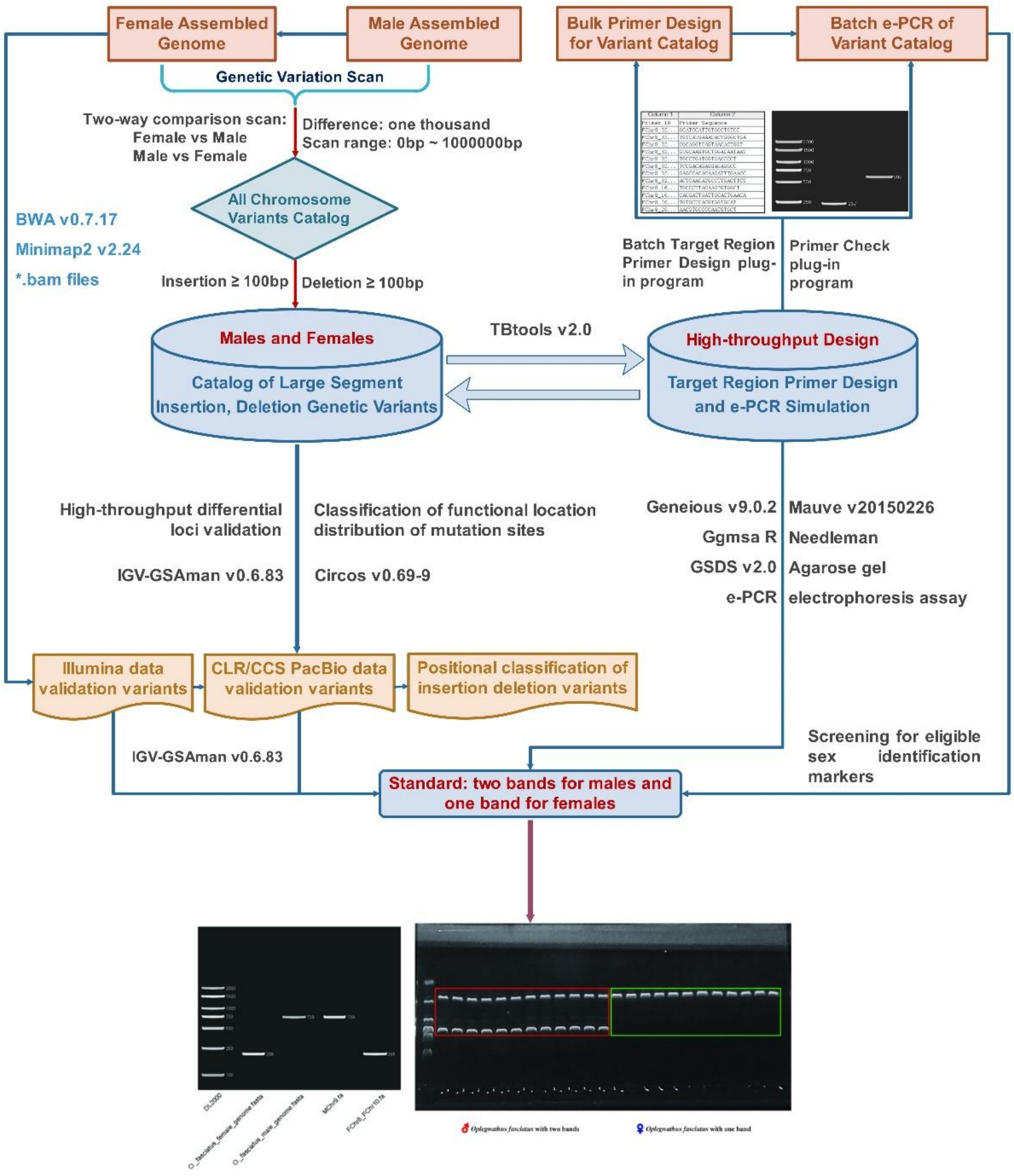

2. Line 46, we revised "a database of large-segment (>100 bp) insertion–deletion" as **"a catalog of large-segment (>100 bp) insertion–deletion".**

3. Line 48, we revised "A database of bulk primers and simulated PCR for the…." as **"A catalog of bulk primers and simulated PCR for the….".**

4. Line 53-54, we revised "A high-throughput identification database of sex-specific markers for *Oplegnathus fasciatus* was constructed utilizing this method…" as "**A high-throughput identification catalog of sex-specific markers for *Oplegnathus fasciatus* was constructed utilizing this method**".

5. Line 56, we revised "Four differential loci were randomly selected from the database for validation, and the……" as **"Four differential loci were randomly selected from**

the catalog for validation, and the…….”.

6. Line 68, we revised “we successfully developed a database encompassing insertion/deletion loci in large segments (>100 bp)” as **“we successfully developed a catalog encompassing insertion/deletion loci in large segments (>100 bp)”**.

7. Line 145, we revised “In this work, a database of genomic differences between females and males was” as **“In this work, a catalog of genomic differences between females and males was”**.

8. Line 147-148, we revised “Using the difference database, we obtained datasets of insertion and deletion variant sites larger than 100 bp….” as “Using the difference **catalog**, we obtained **catalogs** of insertion and deletion variant sites larger than 100 bp…..”.

9. Line 166-167, we revised “a high-throughput precision marker dataset for male and female genetic sex identification was obtained and tested by agarose gel electrophoresis..” as **“a high-throughput precision marker catalog for male and female genetic sex identification was obtained and tested by agarose gel electrophoresis….”**.

10. Line 176-177, we revised “we established databases containing genomic disparities between males and females.” as **“we established catalogs containing genomic disparities between males and females.”**.

11. Line 178-179, we revised “as well as to screen large insertion-deletion sites (>100 bp) against a differential database,” **as “as well as to screen large insertion-deletion sites (>100 bp) against a differential catalogs,”**.

12. Line 221-222, we revised “we screened this insertion/deletion variant site database for variants larger than” as **“we screened this insertion/deletion variant site catalogs for variants larger than”**.

13. Line 462-463, we revised “we successfully developed a database encompassing insertion/deletion loci in large segments (>100 bp) for both male and female *O. fasciatus*” as **“we successfully developed a catalog encompassing insertion/deletion loci in large segments (>100 bp) for both male and female *O. fasciatus*”**.

In addition to mapping Illumina and PacBio reads against references, did the author also consider to directly map both male and female genomes using pairwise whole-genome aligner (for example LASTZ) to see the differences in both genomes? Additionally, I did not find any relevant information in the manuscript on the quality of reference genomes assemblies for male and female fish used for the reads alignments. Authors should clarify whether or not the variants detection was affected by any possible artifacts in the genome assemblies?

Reply:

We would like to extend our sincere thanks to you for your insights and thoughtful suggestions. And it was your suggestion that led us to introduce a new assay to assess the validity of the large fragment insertion deletion sites we obtained.

We used SYRI software for the evaluation of variant sites (≥100bp) between the male and female reference genomes of *Oplegnathus fasciatus*, and using the male genome as a reference for comparison with the female genome, we obtained 2045 deletion sites (≥100bp) and 2110 insertion sites (≥100bp). Meanwhile, we obtained 2116 large segment deletion sites (≥100bp) and 2055 large segment insertion sites (≥100bp) by comparing the female genome with the male genome as a reference. Considering large segment deletion sites better represents the visualization differences between male and female genomes, especially when performing the second generation Illumina and the third generation PacBio CLR data read segments repost visualization presentation. We found that by using the intersection of deletion sites, the results of SYRI scan using male fish as the reference genome showed that 1749 variants out of 2045 deletion sites were in our deletion site catalog set, which accounted for 85.53% of the total number of deletion sites. The results using female fish as the reference genome showed that 1791 out of 2116 deletion sites were in our deletion site catalog set, accounting for 84.64% of the total number of deletion sites. The results of SYRI confirmed that we obtained the large insertion deletion sites of *Oplegnathus fasciatus* are stable, and also obtained more information about the large insertion deletion site for *O. fasciatus*. We have put the information of large segment insertion deletion sites obtained by SYRI and the information of large segment insertion deletion sites intersected with those obtained by us for male and female differences as a supplementary result file in the Appendix, and the intersection results are also provided.

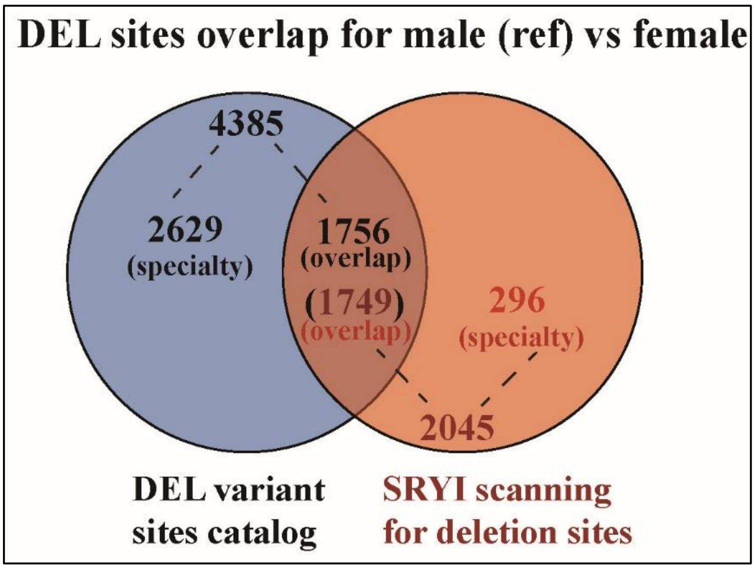

Large fragment deletion site validity was assessed by SYRI software using the male fish genome as a reference

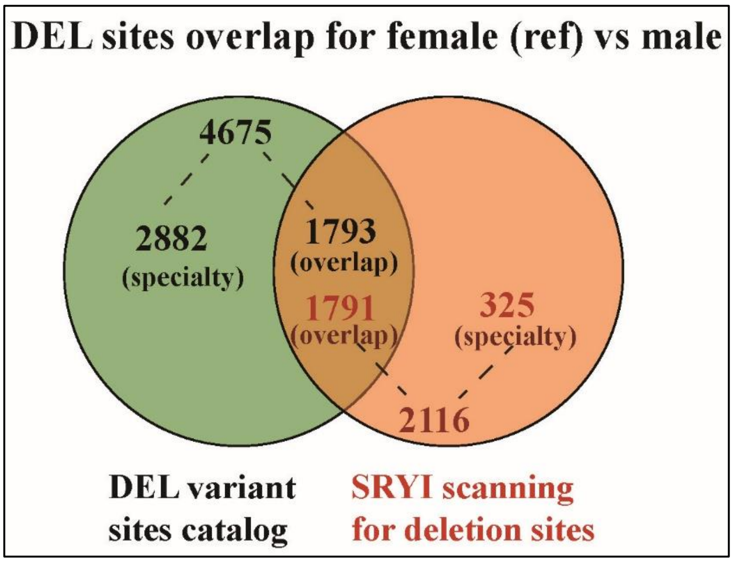

Large fragment deletion site validity was assessed by SYRI software using the female fish genome as a reference

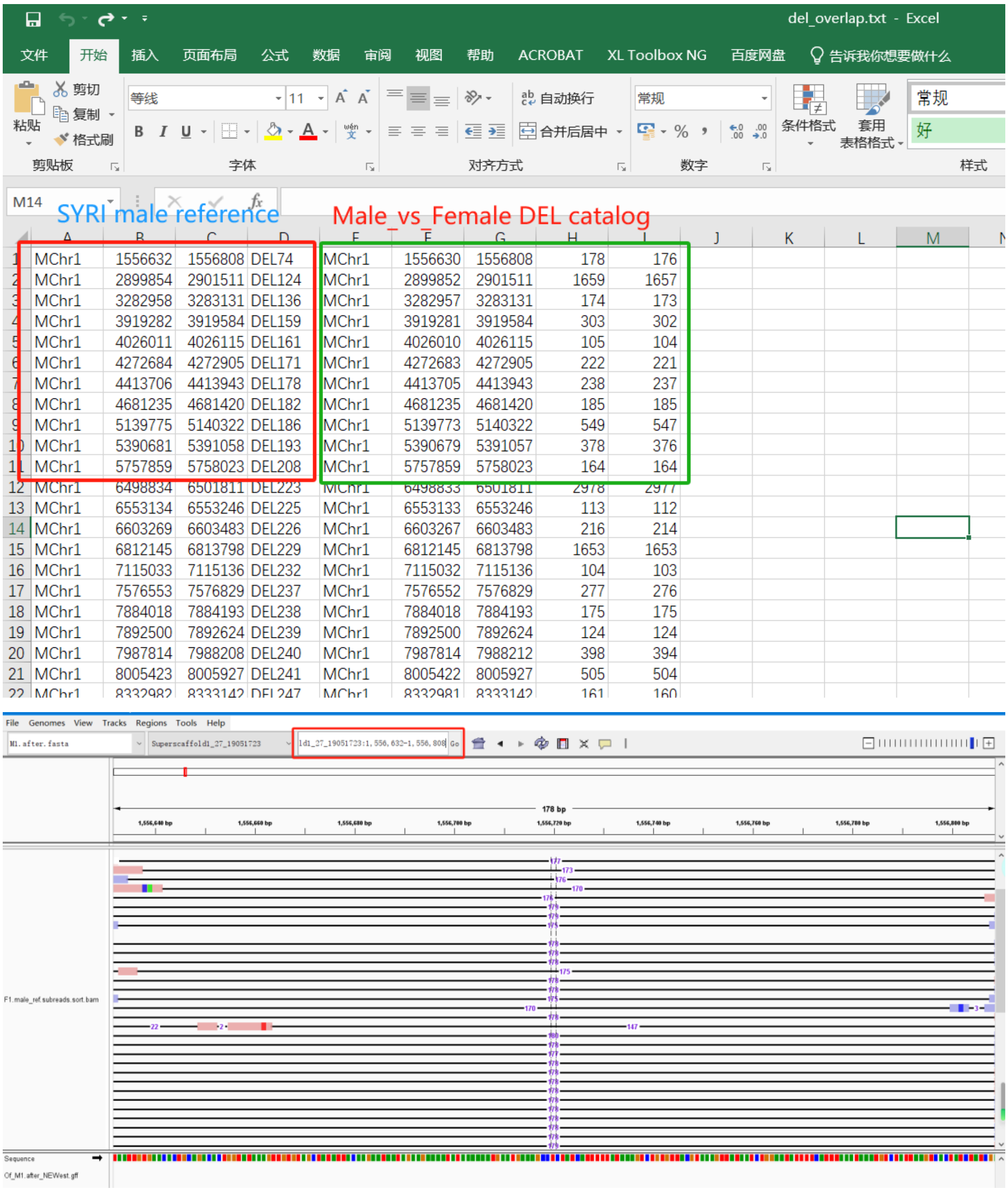

	A	B	C	D	E	F	G	H	I
1	MChr1	1556632	1556808	DEL74	MChr1	1556630	1556808	178	176
2	MChr1	2899854	2901511	DEL124	MChr1	2899852	2901511	1659	1657
3	MChr1	3282958	3283131	DEL136	MChr1	3282957	3283131	174	173
4	MChr1	3919282	3919584	DEL159	MChr1	3919281	3919584	303	302
5	MChr1	4026011	4026115	DEL161	MChr1	4026010	4026115	105	104
6	MChr1	4272684	4272905	DEL171	MChr1	4272683	4272905	222	221
7	MChr1	4413706	4413943	DEL178	MChr1	4413705	4413943	238	237
8	MChr1	4681235	4681420	DEL182	MChr1	4681235	4681420	185	185
9	MChr1	5139775	5140322	DEL186	MChr1	5139773	5140322	549	547
10	MChr1	5390681	5391058	DEL193	MChr1	5390679	5391057	378	376
11	MChr1	5757859	5758023	DEL208	MChr1	5757859	5758023	164	164
12	MChr1	6498834	6501811	DEL223	MChr1	6498833	6501811	2978	2977
13	MChr1	6553134	6553246	DEL225	MChr1	6553133	6553246	113	112
14	MChr1	6603269	6603483	DEL226	MChr1	6603267	6603483	216	214
15	MChr1	6812145	6813798	DEL229	MChr1	6812145	6813798	1653	1653
16	MChr1	7115033	7115136	DEL232	MChr1	7115032	7115136	104	103
17	MChr1	7576553	7576829	DEL237	MChr1	7576552	7576829	277	276
18	MChr1	7884018	7884193	DEL238	MChr1	7884018	7884193	175	175
19	MChr1	7892500	7892624	DEL239	MChr1	7892500	7892624	124	124
20	MChr1	7987814	7988208	DEL240	MChr1	7987814	7988212	398	394
21	MChr1	8005423	8005927	DEL241	MChr1	8005422	8005927	505	504
22	MChr1	8332982	8333142	DEL247	MChr1	8332981	8333142	161	160

SYRI and male fish genomes were used as references to obtain CLR reads with intersecting large deletion sites for repo IGV display

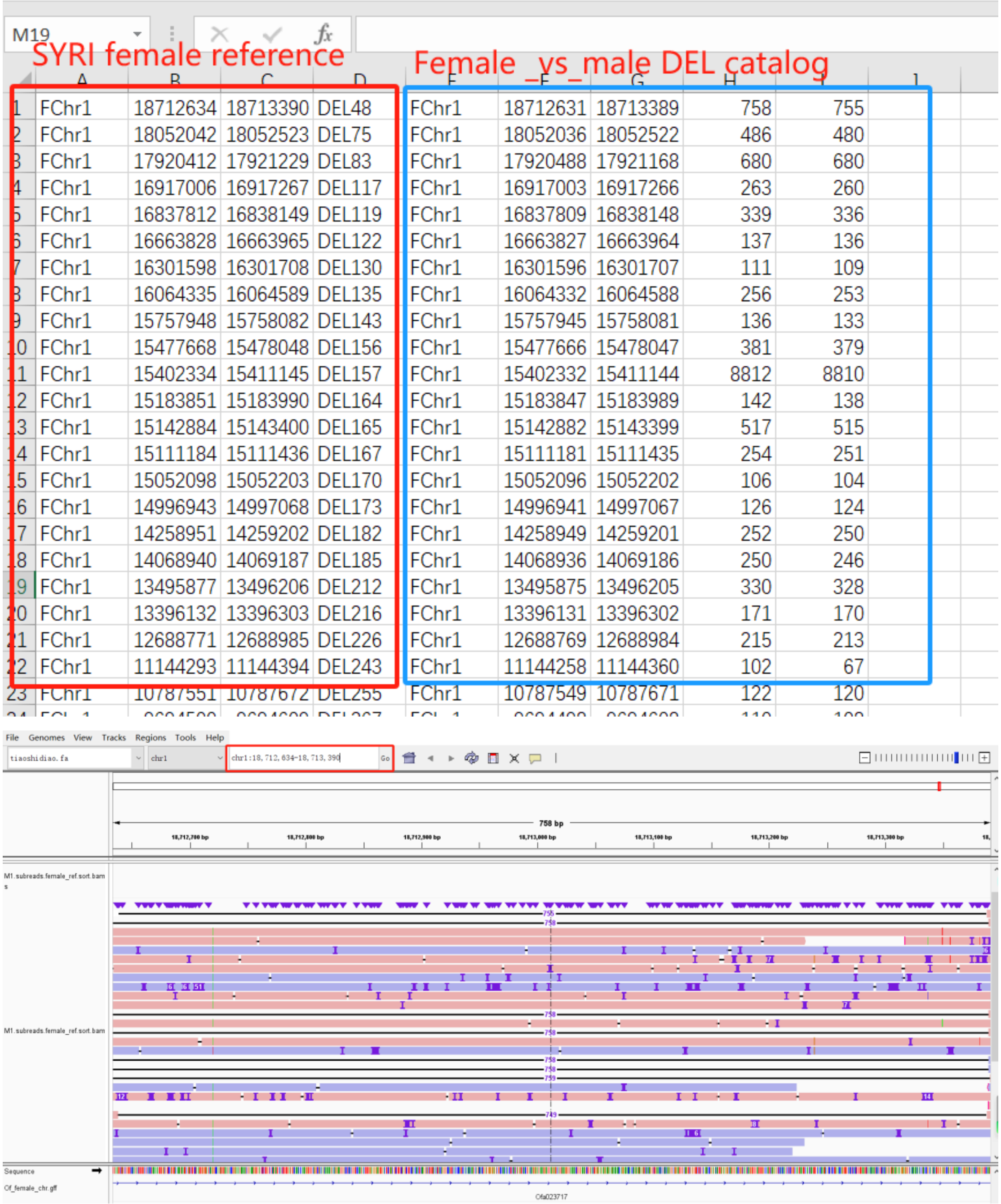

	A	B	C	D	E	F	G	H	I
1	FChr1	18712634	18713390	DEL48	FChr1	18712631	18713389	758	755
2	FChr1	18052042	18052523	DEL75	FChr1	18052036	18052522	486	480
3	FChr1	17920412	17921229	DEL83	FChr1	17920488	17921168	680	680
4	FChr1	16917006	16917267	DEL117	FChr1	16917003	16917266	263	260
5	FChr1	16837812	16838149	DEL119	FChr1	16837809	16838148	339	336
6	FChr1	16663828	16663965	DEL122	FChr1	16663827	16663964	137	136
7	FChr1	16301598	16301708	DEL130	FChr1	16301596	16301707	111	109
8	FChr1	16064335	16064589	DEL135	FChr1	16064332	16064588	256	253
9	FChr1	15757948	15758082	DEL143	FChr1	15757945	15758081	136	133
10	FChr1	15477668	15478048	DEL156	FChr1	15477666	15478047	381	379
11	FChr1	15402334	15411145	DEL157	FChr1	15402332	15411144	8812	8810
12	FChr1	15183851	15183990	DEL164	FChr1	15183847	15183989	142	138
13	FChr1	15142884	15143400	DEL165	FChr1	15142882	15143399	517	515
14	FChr1	15111184	15111436	DEL167	FChr1	15111181	15111435	254	251
15	FChr1	15052098	15052203	DEL170	FChr1	15052096	15052202	106	104
16	FChr1	14996943	14997068	DEL173	FChr1	14996941	14997067	126	124
17	FChr1	14258951	14259202	DEL182	FChr1	14258949	14259201	252	250
18	FChr1	14068940	14069187	DEL185	FChr1	14068936	14069186	250	246
19	FChr1	13495877	13496206	DEL212	FChr1	13495875	13496205	330	328
20	FChr1	13396132	13396303	DEL216	FChr1	13396131	13396302	171	170
21	FChr1	12688771	12688985	DEL226	FChr1	12688769	12688984	215	213
22	FChr1	11144293	11144394	DEL243	FChr1	11144258	11144360	102	67
23	FChr1	10787551	10787672	DEL255	FChr1	10787549	10787671	122	120
24	[illegible]	[illegible]	[illegible]	[illegible]	[illegible]	[illegible]	[illegible]	[illegible]	[illegible]

SYRI and female fish genomes were used as references to obtain CLR reads with intersecting large deletion sites for repo IGV display

The SYRI software runs the following script:

1) nucmer --mum -l 100 -c 1000 FM.fasta OfM_230823_filter_rename.format.fa

2) delta-filter -i 90 -l 100 out.delta > out.filtered.delta

3) Show-coords -THrd out.filtered.delta > out.filtered.coords

4) python3 syri_env/bin/syri -c out.filtered.coords -d out.filtered.delta -r FM.fasta -q OfM_230823_filter_rename.format.fa --nosnp

As your mentioned, we should have stated in the text the assembly quality evaluation parameters of the reference genome used for genetic sex marker exploitation. Therefore, we supplemented the text with the following quality parameters for the assembly of the *Oplegnathus fasciatus* male and female genomes as follows:

"The assembly of the male and female genomes of *O. fasciatus* was successfully completed utilizing PacBio CLR sequencing technology in conjunction with Hi-C assisted assembly technology. The resulting genomes achieved high quality at the chromosome level, with the female genome size measuring 768.81 Mb, contig N50 at 2.13 Mb, and scaffold N50 at 33.55 Mb, while the male genome size was 762.27 Mb, contig N50 at 2.18 Mb, and scaffold N50 at 32.43 Mb. The Benchmarking Universal Single-Copy Orthologs (BUCO) analysis of the genome assemblies for males and females demonstrated a high level of completeness, with percentages reaching 96.6% and 97.2%, respectively."

As you noted, it is important to consider the potential impact of artifacts on the variant sites obtained in the genome assembly. We have incorporated your suggestion by conducting an analysis of the reads depth of the variants, specifically focusing on insertion and deletion sites. This analysis involved counting the reads depth for these sites using male and female reference genomes, and ranking the reads depth of the large fragments of the variants. By implementing this approach, we aimed to enhance the accuracy of the sex marker and mitigate the influence of assembly artifacts on the results.

For the efficiency of long-read based sex-specific markers, authors should clarify how many variants were experimentally validated ? Were there any cases of false-positive variants?

Reply:

We express our gratitude to you for your valuable feedback and suggestions. The development of low-cost high-throughput gender markers utilizing long reads and genomic information represents a research endeavor of significant revolutionary potential. Our current focus is on improving and validating the efficacy of sex-specific markers based on long reads. To this end, we are undertaking a two-pronged approach: first, assessing the stability and reliability of insertion-deletion sites. In this study, we employed two methods to conduct a comprehensive genome-wide analysis and successfully identified stable insertion-deletion sites (results are shown in the Venn diagram). As in our previous study, we found that the male and female chromosomes of *O. fasciatus* differed and that the males underwent a chromosome fusion event.

Moreover, the male fusion chromosome (MChr9) showed significant structural variation compared to its homologous chromosomes (FChr8 and FChr10 in females). Therefore, we focused on the differences between male and female homologous chromosomes that underwent chromosomal structural variation. In order to avoid the influence of the structural dimorphism of the male chromosome on the exploitation of sex markers, we focused on the detection and validation of large segment deletion sites by using the male genome as the reference genome, and by replicating the long segment CLR reads obtained from females to the reference genome of males.

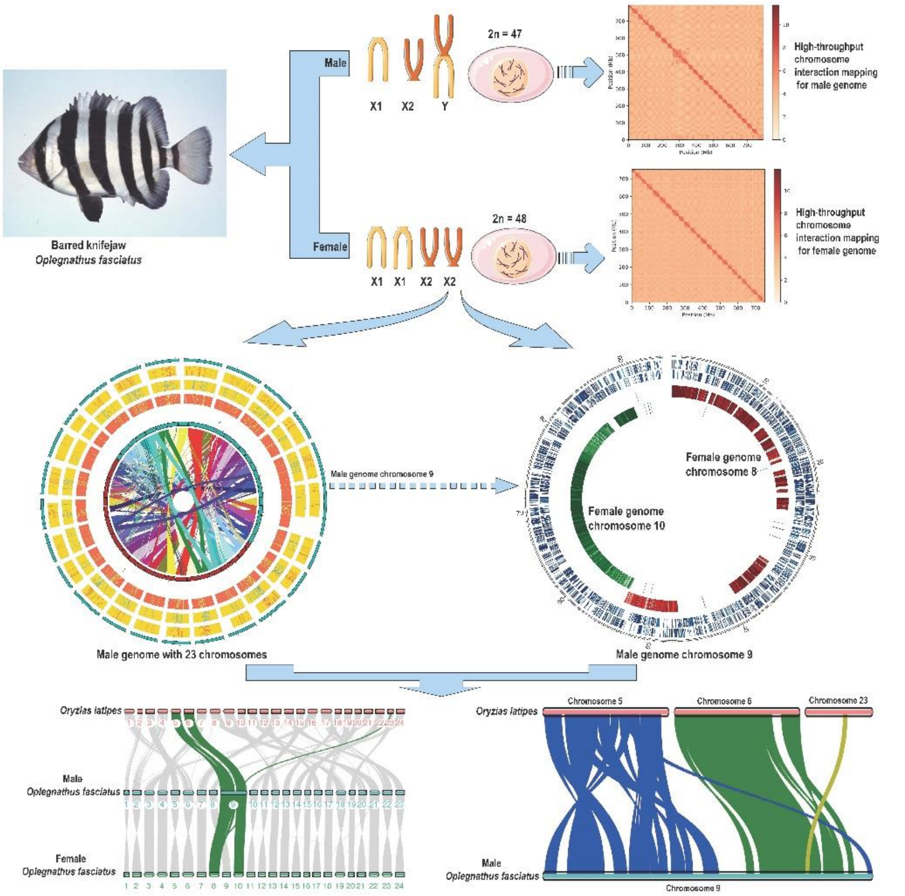

Male and female chromosomal differences in *O. fasciatus* and a chromosomal fusion event in males

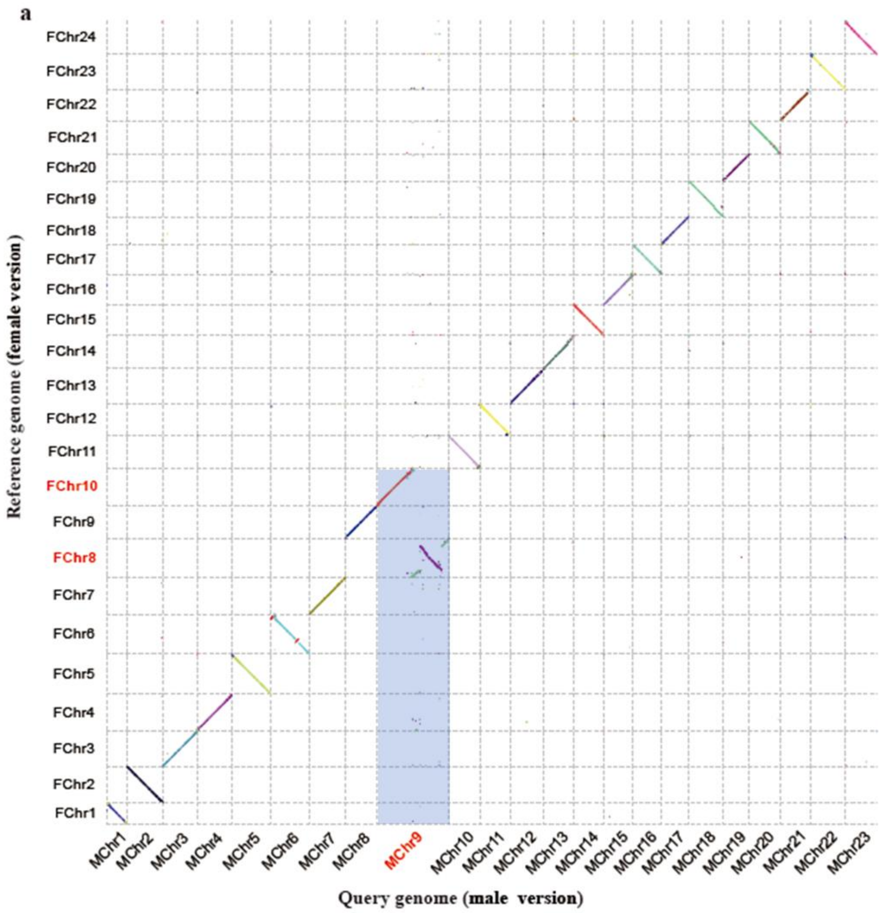

Male fish fusion chromosome (MChr9) undergoes significant structural variation compared to its homologous chromosomes (female FChr8 and FChr10)

We obtained deletion sites for 4675 (female genome as reference) and 4385 (male genome as reference) large segments based on structural variation scans (1~1,000,000bp) at the chromosome level. At the same time, we performed a large segment (≥100bp) variant site scan of the male and female assembled genomes using SYRI software, and obtained 2116 (female genome as reference) and 2045 (male genome as reference) variant sites, respectively, and 84.64% and 85.53% of the variant sites intersected with the variant catalogs obtained by us, respectively. In order to further improve the exploitation efficiency of sex-specific markers based on long reads, we first calculated the read depth of long reads of insertion deletion sites, and then ranked the insertion deletion site markers based on the depth of long reads, for large segment deletion variants, the lower the depth of long reads, the more effective the markers are, so we constructed a frequency map of the distribution of the depth of long reads of the large segment deletion sites of the male and female genomes, and then classified the markers. We found that long read depths less than 5.48 (895 markers) and 12.50 (1,021 markers) belonged to high-recognition sex markers, using the male and

female genomes as references, respectively.

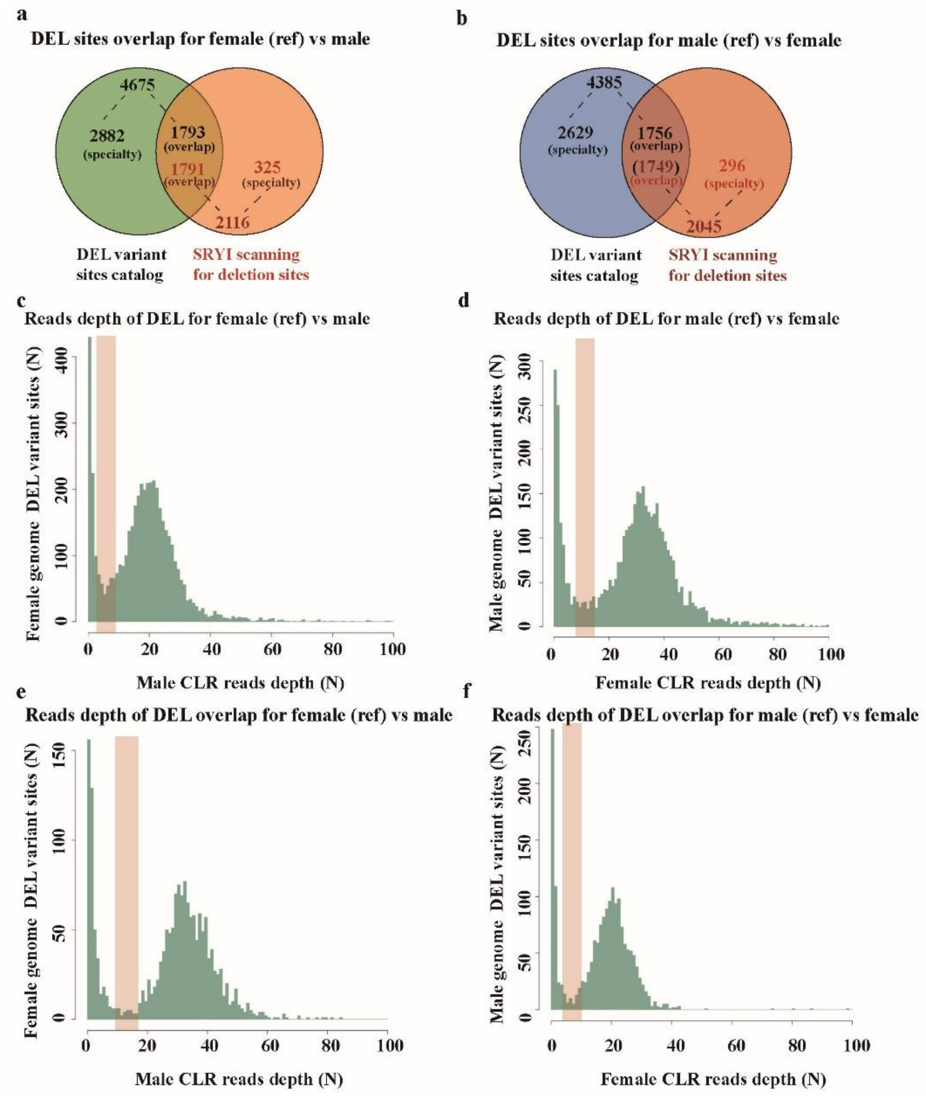

Intersection results of two variant site scanning methods and ranking of variant sites with long read depth frequency distributions

As we have known, stable variant loci are the basis for the exploitation of genetic sex markers, but effective amplification is fundamental for the application of sex markers. In order to validate high-throughput markers for male and female sex and to assist conventional agarose gel electrophoresis PCR, we introduced an electronic simulation of PCR method (e-PCR) for high-throughput amplification of variant markers. Based on this method, on the one hand, we can quickly identify the loci that can be amplified by a single PCR strip, and on the other hand, this method can assist us in the design of more effective primers for the target variant loci, and then quickly carry out the agarose gel electrophoresis PCR assay for the development of markers for both male and female genetic sexes. We targeted the fusion chromosome (MChr9) of male fish and the homologous chromosome (FChr8 and FChr10) variant loci of its female

fish for marker validation and selection, and selected a total of 17 loci located in the intergenic region, intronic region and other typical markers for testing, and successfully completed the validation of the male and female of 11 loci. Meanwhile, we found that the insertion deletion sites are stable, but the non-specific amplification caused by the primers is often an unfavorable factor affecting the subsequent development of sex markers, so how to improve the specificity of the amplification primers and adjust the conditions of the PCR reaction (using touch down method) are the key to the development of markers after the need.

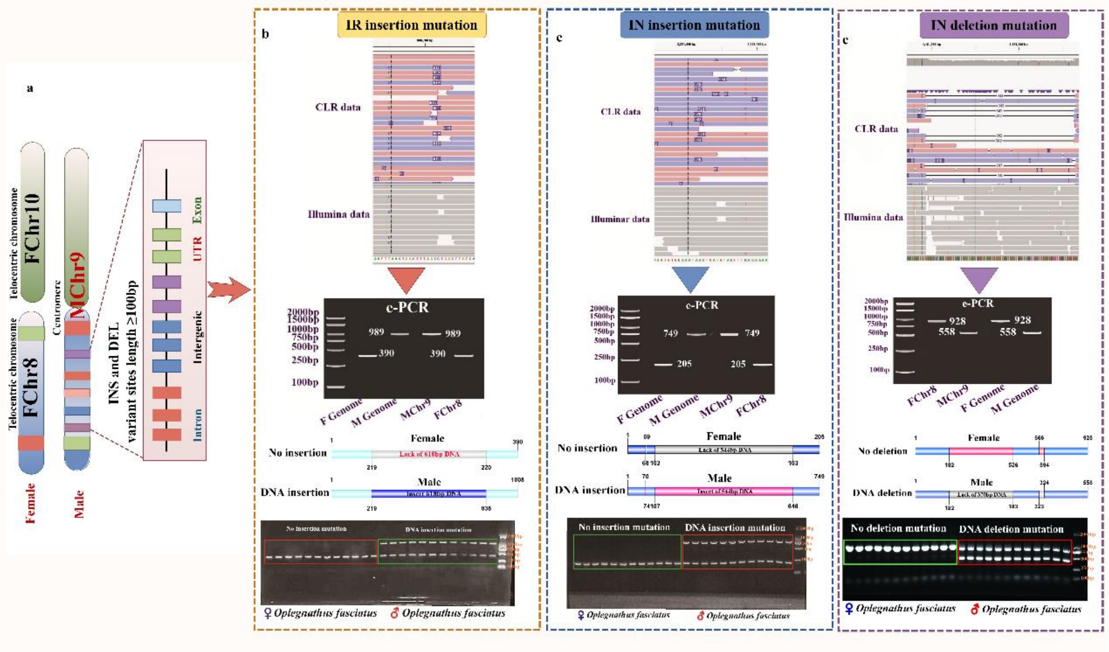

Validation of long read variant loci in males using the female genome as a reference

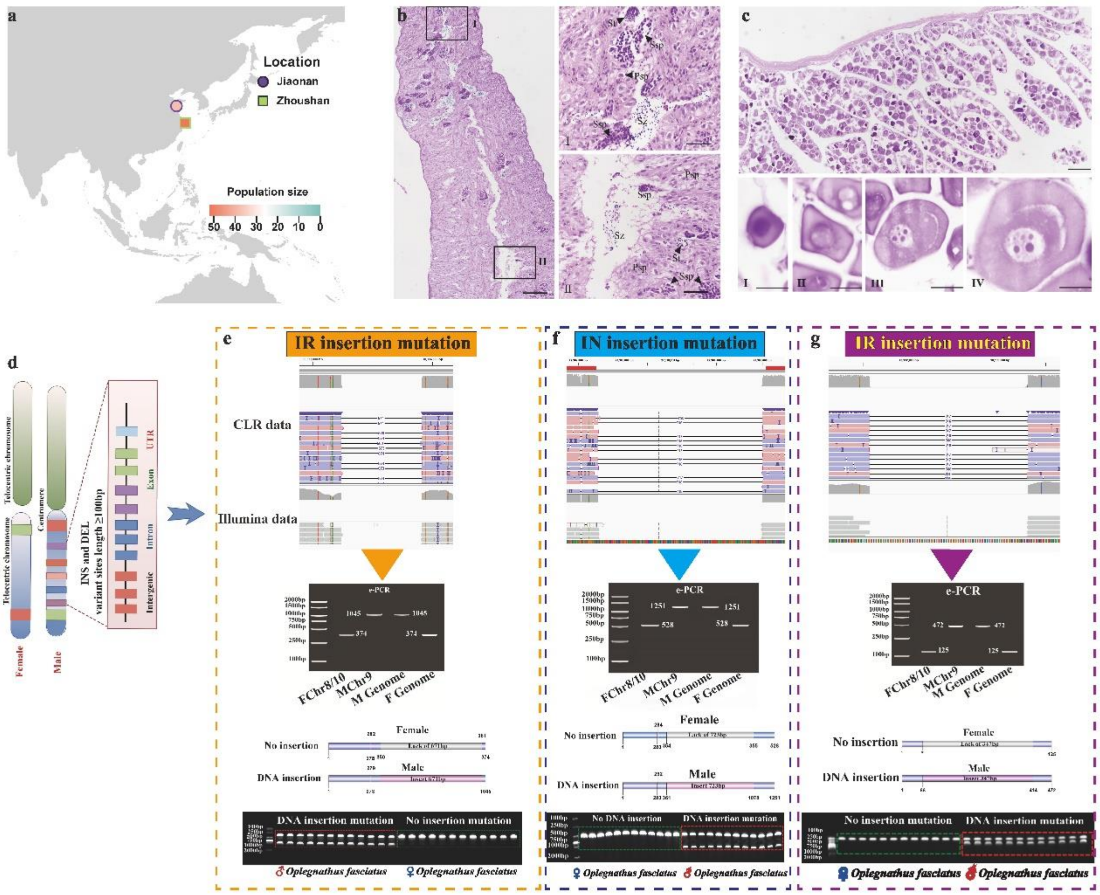

Validation of long read deletion sites for geographic populations using the male genome as a reference

In the manuscript's abstract, there are numerous undefined abbreviations and technical terms. Authors should revise the abstract to minimize the use of jargon. For example:

Line 36-37: "first-generation development methods such as RFLP, 37 RAPD, SSR, and AFLP as well as second-generation techniques utilizing Illumina's 38 SNP/InDel markers."

It is confusing molecular biology techniqies with next generation sequencing (NGS) techniques.

Line 45: "Illumina survey" can be revised "short-read sequencing data survey"

Line 46: "PacBio CLR / CCS" can be revised "long-read PacBio sequencing"

Reply:

We are very grateful to you for your professional advice, which made us realize that biotechnology and sequencing technology are two different concepts, although they are closely related. Therefore, we have reorganized and rewritten the abstract according to your suggestions as follows:

“**Background**

The use of sex-specific molecular markers has become a prominent method in enhancing fish production and economic value, as well as providing a foundation for understanding the complex molecular mechanisms involved in fish sex determination. Over the past decades, research on male and female sex identification has

predominantly employed molecular biology methodologies such as RFLP (Restriction Fragment Length Polymorphism), RAPD (Random Amplification of Polymorphic DNA), SSR (Simple Sequence Repeat), and AFLP (Amplified Fragment Length Polymorphism). The emergence of high-throughput sequencing technologies, particularly Illumina, has led to the utilization of SNP and InDel variants as significant molecular markers for investigating sex identification in fish. The advancement of sex-controlled breeding encounters numerous challenges, including the inefficiency of current methods, intricate experimental protocols, high costs of development, elevated rates of false positives, marker instability, and cumbersome field testing procedures. Nevertheless, the emergence and swift progress of PacBio high-throughput sequencing technology, characterized by its long read output capabilities, offers novel opportunities to overcome these obstacles."

We also appreciate your wonderful suggestions. First, we revised "Illumina survey" and "PacBio CLR / CCS" as "short-read sequencing data survey" and "long-read PacBio sequencing", respectively, as follows:

"Utilizing male-female linear genomic information in conjunction with short-read sequencing data survey and long-read PacBio sequencing data, a catalog of large-segment (>100 bp) insertion-deletion genetic variants was generated through a genome-wide variant site scanning approach with bidirectional comparisons. The sequence tagging sites (STS) were ranked based on the long read depth of the insertion deletion site, with markers exhibiting lower long read depth being considered more effective for large segment deletion variants. Subsequently, a catalog of bulk primers and simulated PCR for the male-female variant loci was developed, incorporating primer design for the target region and e-PCR technology. ~~Finally, t~~The criteria for rapid identification of male and female differences in *Oplegnathus fasciatus* were exploited ~~based on~~through agarose gel electrophoresis, w~~ith~~hich revealed two amplified bands for males and one amplified band for females. A high-throughput identification catalog of sex-specific markers ~~for Oplegnathus fasciatus was~~was then constructed u~~tilizs~~sing this method, ~~yielding~~resulting in the identification of 3,645 and 3,872 markers in conjunction with 1,021 and 894 high-quality genetic sex identification markers,

respectively.~~17~~ Sixteen differential loci were randomly ~~selected~~chosen from the catalog for validation, ~~and~~with 11 of the~~ results met~~m meeting the criteria for male~~-~~-female di~~fferences. Utiliz~~stinctions. The implementation of cost-effective and efficient technological processes within the establishment will facilitate the rapid advancement of genetic breeding through expediting the high-throughput development of sex genetic markers for various species."

"**Conclusions**

Our study utilized linear genomic information from male and female individuals obtained from PacBio, in addition to data from short-read sequencing data survey and long-read PacBio sequencing. We extensively employed genome-wide variant site scanning and identification, high-throughput primer design of target regions, and e-PCR batch amplification, along with statistical analysis and ranking of the long reads depth of the variant sites. Through this integrated approach, we successfully compiled a catalog of large insertion/deletion sites (>100 bp) in both male and female *O. fasciatus*."

On this basis, we revised the manuscript from "Illumina survey and PacBio CLR/CCS data" to "short-read sequencing data survey" and "long-read PacBio sequencing" as follows:

1) Line 318-320, we revised "we utilized the male Illumina survey data and PacBio CLR data for read assignment and further confirmed that this candidate variant site was a valid insertion site" as "we utilized the male short-read sequencing data and long-read PacBio sequencing data for read assignment and further confirmed that this candidate variant site was a valid insertion site".

2) Line 338-340, we revised "A comparison of sequence differences, Illumina survey data and PacBio CLR data read assignments all confirmed that the genetic differences between male and female markers were mainly concentrated in the.." as "A comparison of sequence differences, short-read sequencing data and long-read PacBio sequencing data assignments all confirmed that the genetic differences between male and female markers were mainly concentrated in the….".

3) Line 449-452, we revised "along with data from Illumina surveys and PacBio

CLR/CCS, we extensively employed whole-genome variant site scanning and identification, high-throughput design of primers for the target regions, and e-PCR batch amplification..” as “along with data from the short-read sequencing data and long-read PacBio sequencing data, we extensively employed whole-genome variant site scanning and identification, high-throughput design of primers for the target regions, and e-PCR batch amplification…”

The methods described in this manuscript is not clear and missing details. how many samples were sequenced using Illumina and PacBio? How was the sequencing data preprocessed for QC, poor reads filtering and adapter trimming? What source, database and accession ID was the reference genomes of male and female downloaded? It is mentioned that aligner BWA (v 0.7.8) and Minimap2 were used, but as could not follow which aligner map Illumina or PacBio reads?

Reply:

We are very grateful to your constructive comments, and we have added the sample sources of the genomes and the quality evaluation parameters of the high-throughput sequencing data as follows:

Table 1. Parameters for Illumina and PacBio high-throughput sequencing and sequencing quality assessment of male and female *O. fasciatus* assembled genomes

Illumina platform (Short-read sequencing data survey)		
	Male *O. fasciatus*	Female *O. fasciatus*
Reads number	432,091,798	613,163,122
Raw data (bp)	63,910,932,339	90,702,298,813
Clean data (bp)	56,617,669,600	80,887,039,002
Read length (bp)	148, 147	148, 147
Q20 (%)	97.9, 97.3	98.0, 97.1
Q30 (%)	93.8, 92.0	94.1, 91.4
PacBio Sequel platform (Long-read PacBio sequencing data)		
	Male *O. fasciatus*	Female *O. fasciatus*
Polymerase read number	3,674,737	4,760,021
Polymerase read bases (bp)	39,844,228,109	62,877,786,278
Average polymerase read length (bp)	10,847	13,219

Polymerase read N50 (bp)	18,850	22,964
Subreads number	4,711,720	6,689,662
Total bases of subreads (bp)	39,793,172,442	62,783,888,050
Average subread length (bp)	8,448	9,397
Length of genome (bp)	762,267,613	768,808,243
Number of contigs	1,355	1,372
Contigs N50 (bp)	2,183,645	2,130,780
Number of scaffold	23	24
Scaffold N50 (bp)	32,431,321	33,548,962
Genome coverage (X)	251.1	314.6
Complete BUSCOs (%)	98.2	98.0

"**Sample collection, sequencing and genome quality**

Male and female *O. fasciatus* captured from Qingdao, Shandong Province (Yellow Sea) were utilized for genome sequencing and assembly. High-quality genomic DNA was extracted from fresh muscle tissue and blood samples from pairs of male and female *O. fasciatus* using the Illumina platform (Illumina Inc., San Diego, CA, USA) and PacBio Sequel (Pacific Biosciences of California, Menlo Park, CA, USA). Genomic DNA from fresh muscle tissue and blood samples of male and female *O. fasciatus* pairs was extracted to construct short insert libraries (300~350 bp) for males and females, respectively, using standard protocols provided by the Illumina HiSeq X Ten platform (Illumina Inc., San Diego, CA, USA). Raw reads totaling approximately 90.7 Gb and 63.9 Gb were generated. After removing splice-containing sequences, low-quality, and redundant reads, approximately 80.8 Gb and 56.6 Gb of pure data were obtained from the genomes of males and females, respectively, corresponding to Q20 averages of 97.55% and 97.60%, respectively. Genomic DNA libraries (20 kb) for male and female fish were then constructed using the PacBio Sequel platform (Pacific Biosciences of California, Menlo Park, CA, USA). Sequencing was performed on the PacBio Sequel platform using the

Sequel Binding Kit 2.0, Sequel Sequencing Kit 2.1, and Sequel SMRT Cell 1M v2. Seven and five SMRT Cells were used for sequencing the male and female genomes, respectively, with SMRT LINK 5.0 used to filter the raw data from the zero-mode waveguide. We obtained 62.8Gb and 39.8Gb of long read length PacBio sequencing data for female (accession SRP160016) and male (accession SRP220007) genome assembly, respectively. The female and male *O. fasciatus* genome were de novo assembled using Canu v1.4 with Corrected-Error-Rate set to 0.040, followed by redundancy removal using Redundans v0.13c (minCoverage = 15), genome polishing with Arrow tool in SMRT Link 5.0, and final assembly refinement with Pilon using Illumina NGS short reads from the genome surveys. Based on the male and female draft genomes and Hi-C technology, we obtained high-quality genomes at the chromosome level of female (768.8Mb, accession SUB14064808) and male (762.2Mb, accession SUB14065793) *O. fasciatus*, and the contig N50 and scaffold N50 of the male and female fish genomes reached 2.13 Mb/2.18 Mb and 33.5 Mb/32.4 Mb, respectively. To assess the genomic completeness of assembled male and female *O. fasciatus*, we subjected the assembled sequences to BUSCO version 5 evaluation (BUSCO, actinopterygii_odb10), with Complete BUSCOs assessed values up to 98.2% and 98.0% for male and female genomes, respectively."

One of my main concerns is the simulated e-PCR. I am not sure, how much this technique is reliable for validation studies and what is the rate of false positive/negative relative to the actual experimental PCR technique. My question to authors, Are, there any previous studied that have utilized the e-PCR amplified fragment sizes for genome variants validation? If yes, it is recommended to cite with brief description of reliability of this e-PCR versus normal PCR in lab.

Reply:

Thanks to your meticulous suggestions, as you mentioned e-PCR is indeed a very interesting tool, we checked the development of e-PCR, single sequence tagged site (STS) was proposed by Olson et al in 1989, and later Schuler Gregory D. Later, in Rotmistrovsky et al (2004), this technique was developed and published in the Nucleic Acids Research, which allowed the use of e-PCR with a single primer on the web, specifically for the Human Genome UCSC website database. As envisioned by Schuler

Gregory D. (1997), the proponent of e-PCR, the advantage of e-PCR is that STS loci can be identified without any experimentation, which will be important for modern genomics research. The limitations of computer language, limited genomic data and the amplification of a single STS have not allowed the proposed rapid visualisation, making the technical application of e-PCR limited.

GENOME METHODS

Sequence Mapping by Electronic PCR

Gregory D. Schuler[1]

National Center for Biotechnology Information, National Library of Medicine, National Institutes of Health, Bethesda, Maryland 20984

The highly specific and sensitive PCR provides the basis for sequence-tagged sites (STSs), unique landmarks that have been used widely in the construction of genetic and physical maps of the human genome. Electronic PCR (e-PCR) refers to the process of recovering these unique sites in DNA sequences by searching for subsequences that closely match the PCR primers and have the correct order, orientation, and spacing that they could plausibly prime the amplification of a PCR product of the correct molecular weight. A software tool was developed to provide an efficient implementation of this search strategy and allow the sort of en masse searching that is required for modern genome analysis. Some sample searches were performed to demonstrate a number of factors that can affect the likelihood of obtaining a match. Analysis of one large sequence database record revealed the presence of several microsatellite and gene-based markers and allowed the exact base-pair distances among them to be calculated. This example provides a demonstration of how e-PCR can be used to integrate the growing body of genomic sequence data with existing maps, reveal relationships among markers that existed previously on different maps, and correlate genetic distances with physical distances.

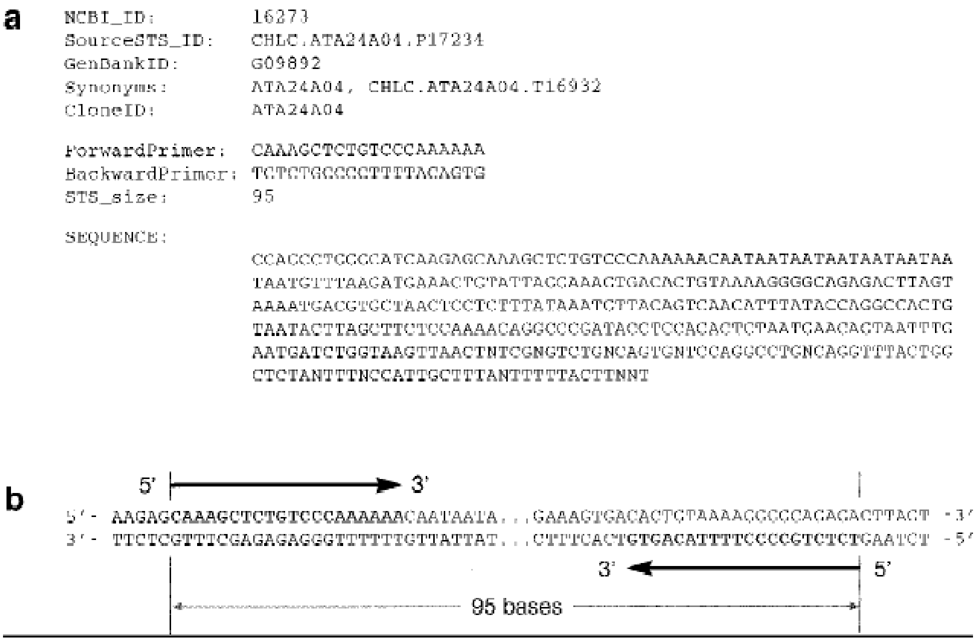

Figure 1 PCR primer sequences from a typical dbSTS record and their relationship to a query sequence that might be searched by e-PCR. (*a*) A few selected fields are shown from dbSTS record 16273 (GenBank accession no. G09892), including various names and identifiers, the sequences of the forward and reverse primers (both in 5′ → 3′ orientation), the size of the PCR product, and the sequence of the amplicon and flanking regions. (*b*) For a query sequence that is the same sense as the sequence of the dbSTS record, a successful match will include the forward primer followed by the inverse (i.e., reverse-compliment) of the reverse primer. On the other hand, if the query sequence is of the opposite sense (imagine the lower strand reversed), it will be the reverse primer followed by the inverse of the forward primer.

Gregory D. Schuler. (1997) Sequence Mapping by Electronic PCR, Genome Research, 7:541–550.

W108–W112 *Nucleic Acids Research, 2004, Vol. 32, Web Server issue*
DOI: 10.1093/nar/gkh450

A web server for performing electronic PCR

Kirill Rotmistrovsky, Wonhee Jang and Gregory D. Schuler*

National Center for Biotechnology Information, National Library of Medicine, National Institutes of Health, Bethesda, MD 20984, USA

Received February 27, 2004; Revised and Accepted April 21, 2004

ABSTRACT

'Electronic PCR' (e-PCR) refers to a computational procedure that is used to search DNA sequences for sequence tagged sites (STSs), each of which is defined by a pair of primer sequences and an expected PCR product size. To gain speed, our implementation extracts short 'words' from the 3′ end of each primer and stores them in a sorted hash table that can be accessed efficiently during the search. One recent improvement is the use of overlapping discontinuous words to allow matches to be found despite the presence of a mismatch. Moreover, it is possible to allow gaps in the alignment between the primer and the sequence. The effect of these changes is to improve sensitivity without significantly affecting specificity. The new software provides a search mode using a query STS against a sequence database to augment the previously available mode using a query sequence against an STS database. Finally, e-PCR may now be used through a web service, with search results linked to other web resources such as the UniSTS database and the MapViewer genome browser. The e-PCR web server may be found at www.ncbi.nlm.nih.gov/sutils/e-pcr.

in being able to cross-reference large collections of PCR primer pairs and sequences.

We have previously described the concept of 'electronic PCR' (e-PCR) as a computational procedure for finding sequence tagged sites within DNA sequences and provided an efficient implementation of this procedure (2). To gain speed we employed the commonly used strategy of hashing, in which the bases from a window of size *W* (a 'word') are used as an index into a hash table (for an overview of program parameters, see Table 1). Each time a matching word is found, a portion of the sequence is checked for an alignment to the corresponding primer. Finally, a match is reported if both primers are found in the correct orientation and imply a product size that is within *M* bases of the expected size (Figure 1). Increasing the value of *W* accelerates the search by reducing the background of word matches that must be investigated. In the original implementation of the program, only one word was hashed per primer and the requirement that its *W* contiguous bases match exactly led to a loss of sensitivity. Even though mismatches were allowed in the primer alignment step, their presence within the hashed word was sufficient to deny a match.

In previous reports, we have described applications of e-PCR for binding genomic and transcribed sequences to map positions (2) and for using STS maps to assess the quality and completeness of a genomic sequence (3). Here we describe algorithmic changes that result in improved sensitivity, a search mode in which a query STS can be compared to a sequence database, and a web server for performing e-PCR.

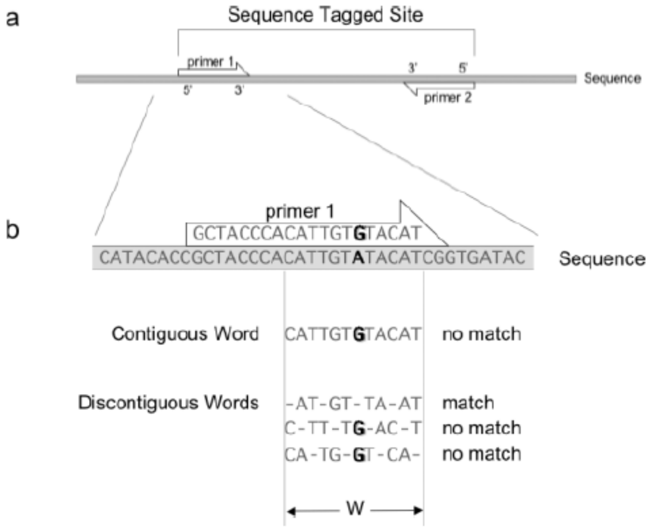

Figure 1. Electronic PCR concepts. (**a**) An STS is defined by a pair of primers which anneal to the target DNA in opposite orientations. Each primer is extended on its 3′ end in the direction of the other primer by *Taq* polymerase. Multiple cycles of annealing and extension lead to a substantial amplification of the STS sequence, also known as the 'amplicon'. (**b**) By default, a single contiguous word of *W* bases ($W = 9$ in this example) is extracted from the 3′ end of each primer. Any mismatch or gap within this region is sufficient to eliminate a match. With discontiguous words enabled ($F = 3$), three words are indexed, each with a 'wildcard' position every third base. Staggering of the wildcard positions ensures that no single mismatch in this region will deny a match.

Kirill Rotmistrovsky, Wonhee Jang and Gregory D. Schuler. (2004) A web server for performing electronic PCR. Nucleic Acids Research, 32: W108-W112.

With the development of high-throughput sequencing technology and the improvement of genome analysis methods, the flux identification of sequence tagged sites (STSs) has increased dramatically, and e-PCR prediction of individual STSs as well as conventional PCR assays are not capable of timely validation, which limits the high-throughput identification of variant markers and high-throughput development. Therefore, we integrated high-throughput identification of large insertion-deletion sequence marker sites, high-throughput design of e-PCR primers for STS, and high-throughput amplification and validation of e-PCR primers for STS to realize high-throughput development and validation of male and female genetic (STS) markers based on male and female genome data, and accelerate the process of fish genetic breeding. Let's take a case study to illustrate efficient visualization of single and high-throughput sequence tagged sites.

Female genome STS

GGACCGGGGCAAGGGACCCACACAATCGATGATCAAGTGTTCAAAAGG
CTGTCCAATTGCAGGGATTGGATGCAAGGGAGCAGGTTTAACAACTTGATT
AGGCTTGCCAGTGATCTGACAGGTATGGCATGTTTTGATGAAGAGAGCGAC
GTTGCATGGCCAGAAGAAATATTGCAGTATACGATGGTAGGTTTTATGGATG
CCAAGATGACCTGCCACATCACCATGTGACGTCTGGAGTACTTTACTGCGA
CAGTTGGTAGGAACAACAACTTTAAAAATTGGCTCTCCTACAAACTGTTCA
CCATGGGGCATTCACTTTCTAACCAGGTTACTGGTTAGAAAGTGAA**AGCCT
CCCTGCACTCAGATGTTAG**

Male genome STS

GGACCGGGGCAAGGGACCCACACAATCGATGATCAAGTGTTCAAAAGG
CTGTCCAATTGCAGGGATTGGATGCAAGGGAGCAGGTTTAACAACTTGATT
AGGCTTGCCAGTGATCTGACAGGTATGGCATGTTTTGATGAAGAGAGCGAC
GTCGCGTGGCCAGAAGAAATAATGCAGTATACGATGGTAGGTTTTATGGATG
CCAAGATGACCTGCCACATCACCATGTGACGTCTGGAGTACTTTACTGCGA
CATTTGGTAGGAACAACAACTTTTGTTGGCTCTCCTACAAACTGTTCACCAT
GGGGCATTCACTTTCTAACCAGGTTACTGGTTAGAAAGTGAATGCCCCATG
GTGAACAGTTCTAGGAGGAAATACCACTGAGCATTATTTCTCACCTCTGTC

GCTGGCACAACTCTGTCAAATAGCTCAGTTAAGGAAGAATCTGCCTTCTGC

TCTGCCACTAACTCAGCCTGAGAACCTGCTAGCTGGTCAACTGGCAATGAA

ACTGGCAACTCCAAATACTCAGGCTCCACCACATCCTGTGCGGCATGGCTG

GCAGCACGGGTGACAGCACAGGCTGGAAAAGTGTCATCCAGTGACCTCTC

TGCGGGTGCTACCACAGTGGGAGACGTTGGCTGCTTCACTACATTTGGCAC

GCCATCGGCCCACACCTTGTCCCCAGCCAAATCATTCCCCAAAATCATATGC

ACCCCTTCAACAGGCAACTGGGGACGGACACCAACTGACACTTCACCCTT

TACCACAAGTGAGACTCACTTGGTGCAAAGGAACAGATAATGGAACCAAA

CCCATTCCTAGAATGATTACACACCCACCAGTATCAGTTTTGGATGAAAATG

GCAACATAGACTGAAAGAATCCAACGCACCAGTGTCTCTTAGAATTTTAAT

GGGAACCTGCACACTACTCCCCACCAATGAGACCAACCCATCCGATTTGAA

GGCAGTTTGATCAGCTCGCAGTGACGCCAGGTCAGTATGTGACTGCAACTG

ACC**<u>AGCCTCCCTGCACTCAGATGTTAG</u>**

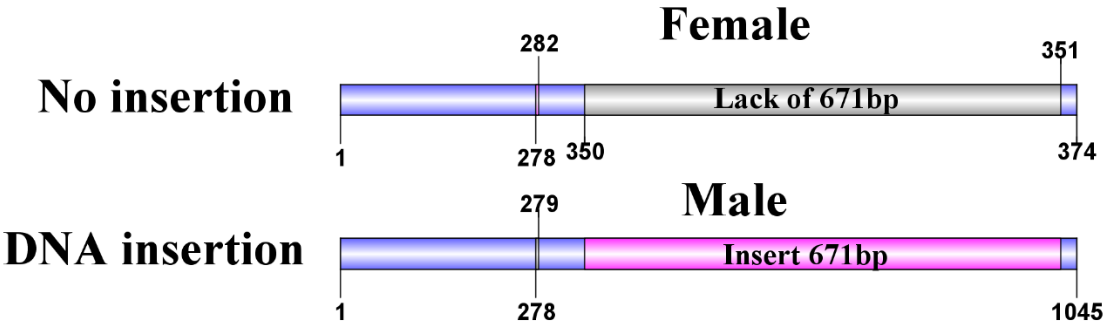

Sequence marker site male-female difference pattern map

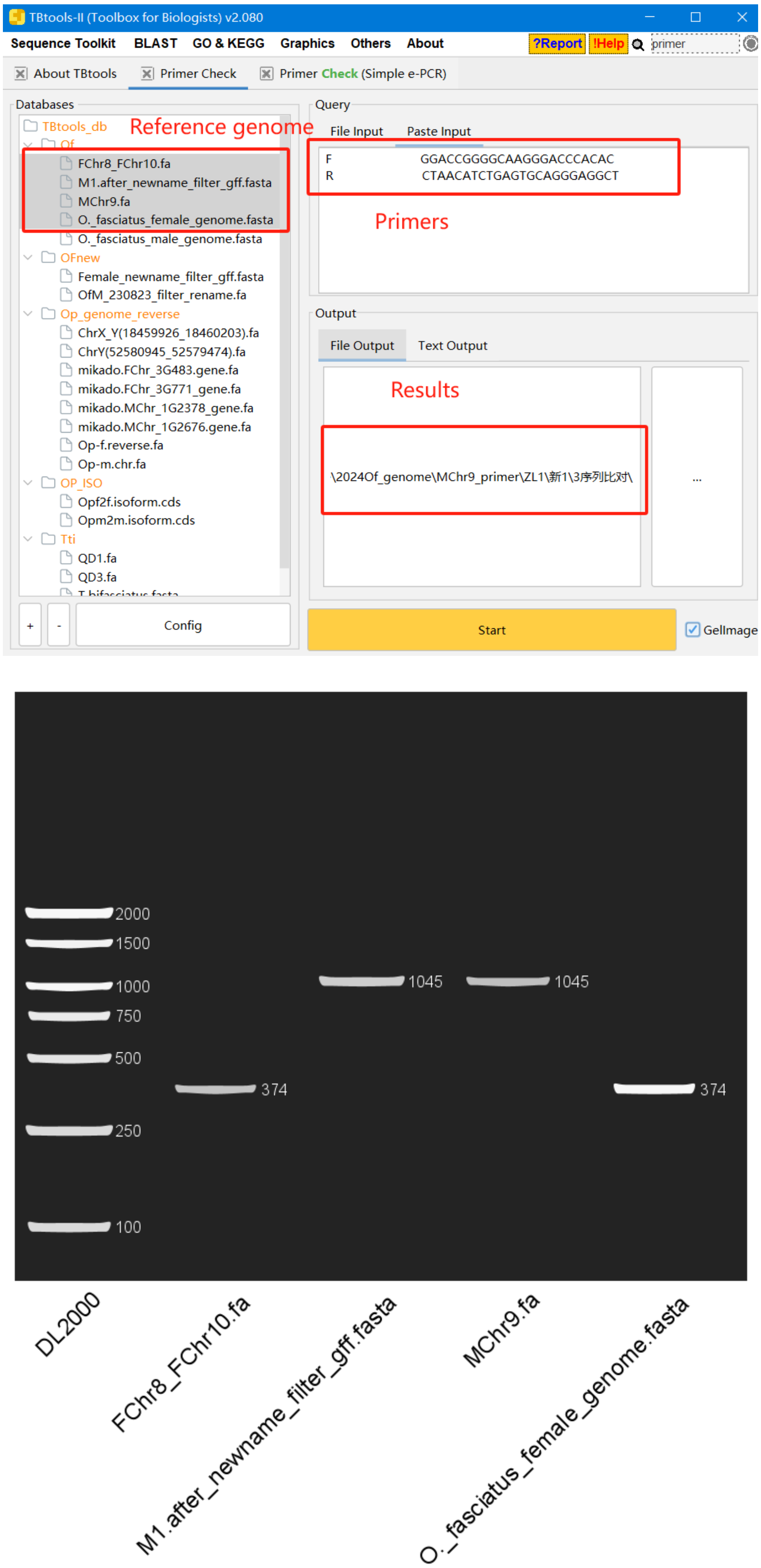

Electronic PCR visualization and display process for sequence tagged sites

On this basis, STS high-throughput e-PCR amplification detection was realized by using high-throughput scanning of sequence tagged sites combined with e-PCR method.

High-throughput e-PCR amplification not only allows rapid detection of the amplification efficiency of STS loci, but also provides an aid and reference for primer design for subsequent conventional PCR.

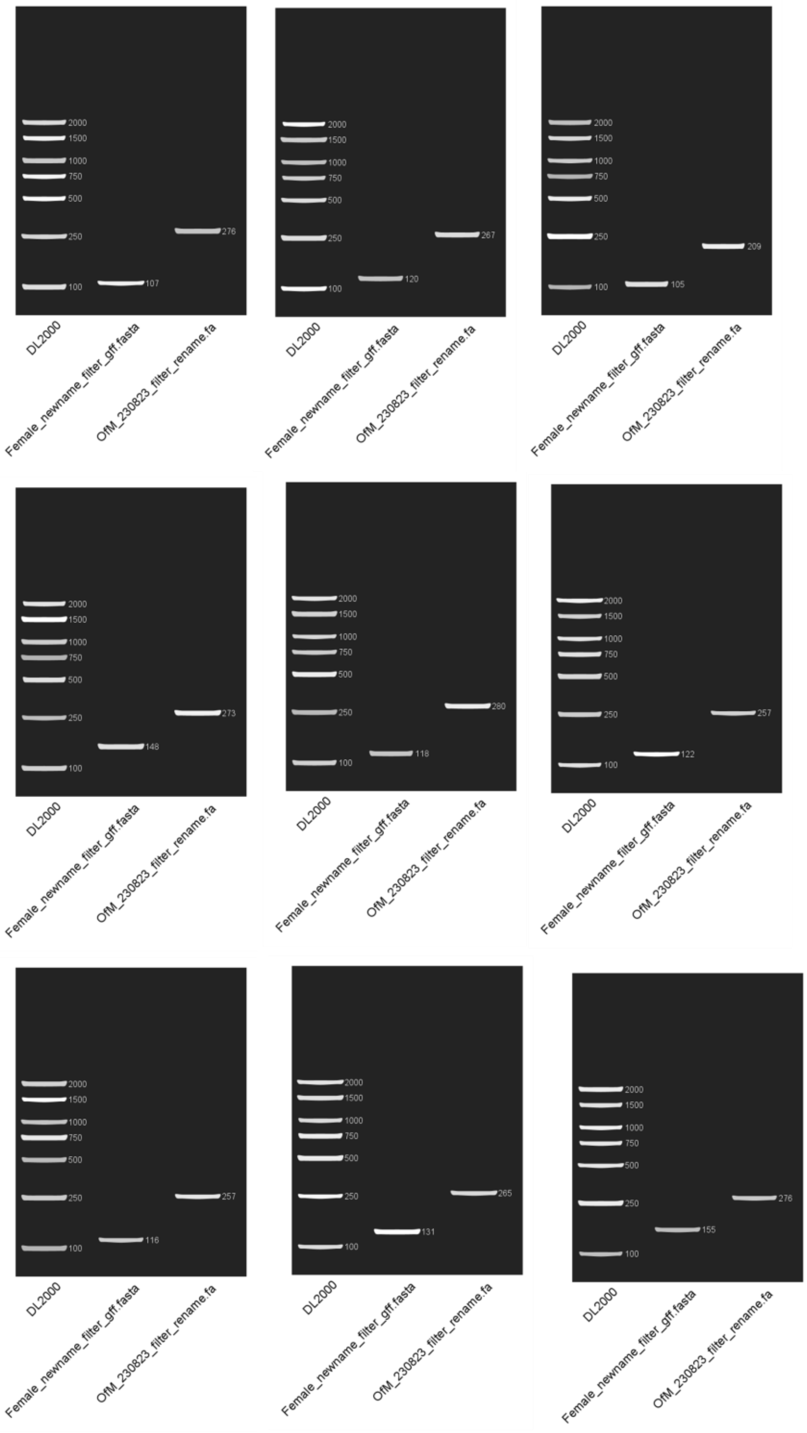

High-throughput e-PCR amplification assay for STS

The gene otology enrichment findings seems to be out of context from the scope of the manuscript. The authors described the enriched GO terms for the exonic variants detected in their analysis, but

there is no discussions for the meaningful biology of these functions and most of these results are just descriptive reports without further mechanisms and consequences explanation.

Reply:

We appreciate your constructive comments, and based on your and the other reviewers' common suggestions, which we agreed by deliberation were extremely professional, we have removed that section from the manuscript.

The figures captions are not clearly described to explain the data. For example, in figure 3, Line 251-252, what is meaning of different alignments patterns in dotplots? All the captions should be explanatory for the data plotted in figures.

Reply:

Thanks to your professional advice, We have organized all the images as requested the results are explained below

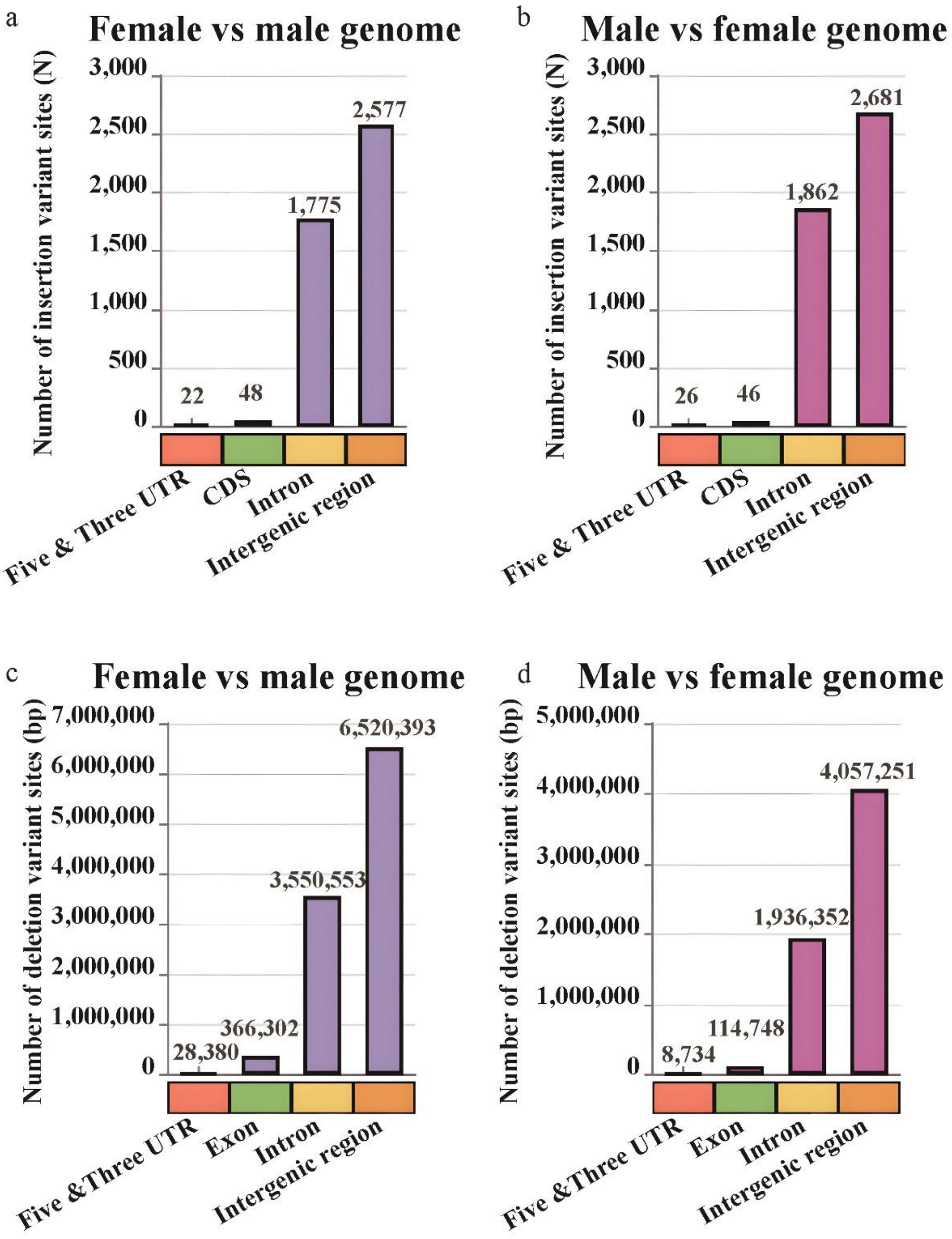

Figure 2: Distribution statistics of insertion and deletion fragment (＞100bp) positions in genomic functional regions of *O. fasciatus*.

(a) Distribution statistics of insertion variant loci using the female fish genome as a reference. (b) Distribution statistics of insertion variant loci utilizing the male fish genome as a point of reference. (c) Distribution statistics of deletion variant length using the female fish genome as a reference. (d) Distribution statistics of deletion variant loci using the male fish genome as a reference.

Note: The colors red, green, yellow, and orange represented the UTR, CDS, intron, and intergenic regions. The frequency of insertions (a, b) was quantified based on the number of events, while deletions (c, d) were characterized by their length in nucleotides.

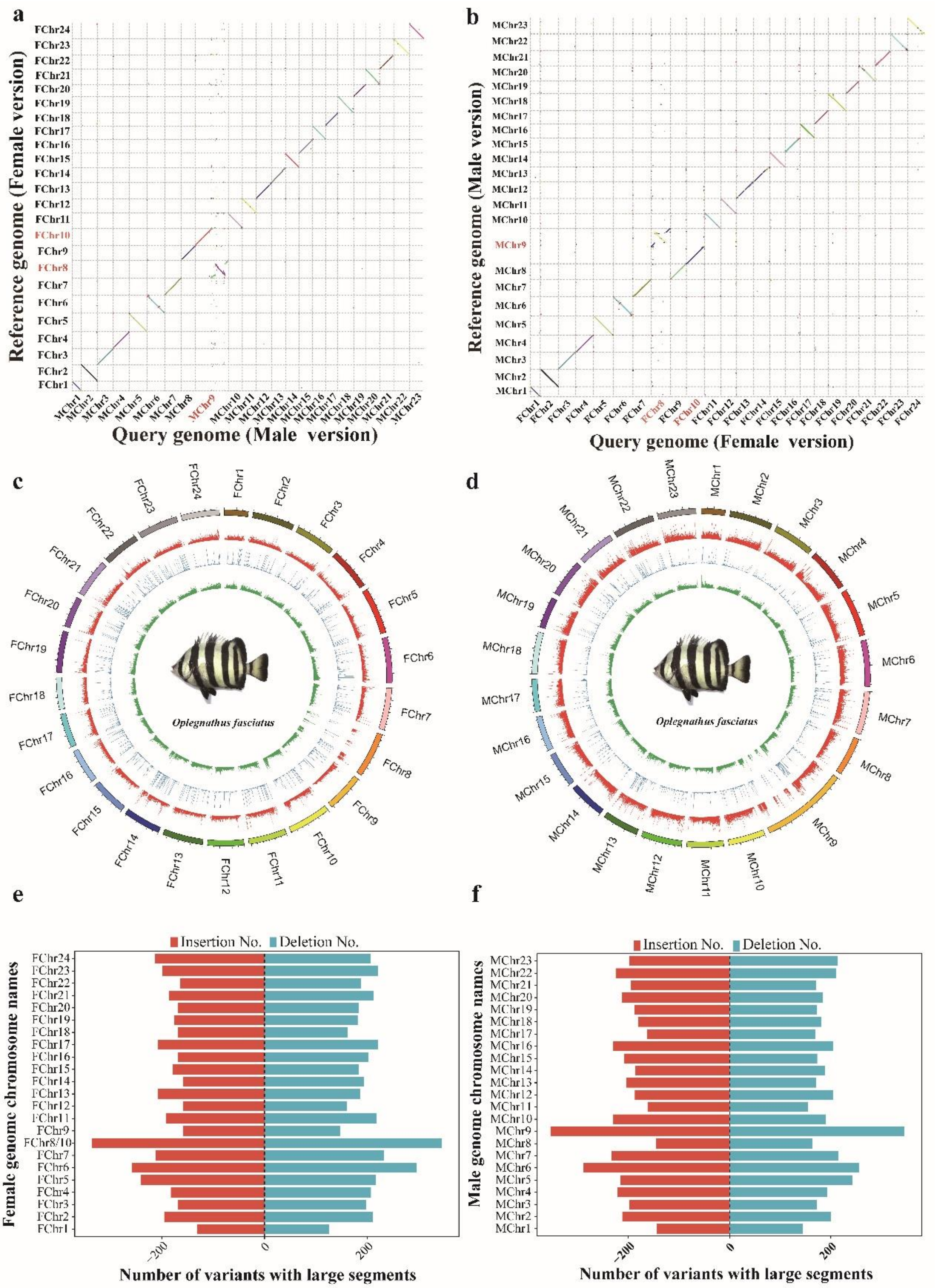

Figure 3: The covariance of genomic sequence differences between the male and female genomes of *O. fasciatus* and distribution statistics of variant sites.

(a) The female genome (reference) of *O. fasciatus* vs. the male genome for homology comparison using the minimap2 software with a 5000 bp sliding window. The female genome was the vertical axis, and female chromosomes 8 and 10 were homologous to male chromosome 9. (b) The comparison of homology between the male genome of *O. fasciatus* and the female genome was conducted using the minimap2 software with a 5000 bp sliding window. The male genome was represented on the vertical axis, revealing homology between male chromosome 9 and female chromosomes 8 and 10. (c) Distribution statistics of variant loci using the female fish

genome as a reference. Insertions (>100 bp) were summarized by number of events. Deletions (>100 bp) were summarized by length in nucleotides. From outer to inner circles were the chromosome name, positional distribution of insertion sites, base length distribution of deletion sites, and base substitution distribution, respectively. (d) Distribution statistics of variant loci using the male fish genome as a reference. (e) - (f) Dual-valued histograms corresponding to the Table 3 showed the number of insertion/deletion sites per chromosome with reference to the female (4,422 inertion sites, 4,675 deletion sites) and male (4,615 inertion sites, 4,385 deletion sites) genomes, respectively, where chromosome 9 in males was homologous to chromosomes 8 and 10 in females. Insertion sites were represented by negative values, and deletion sites were represented by positive values..

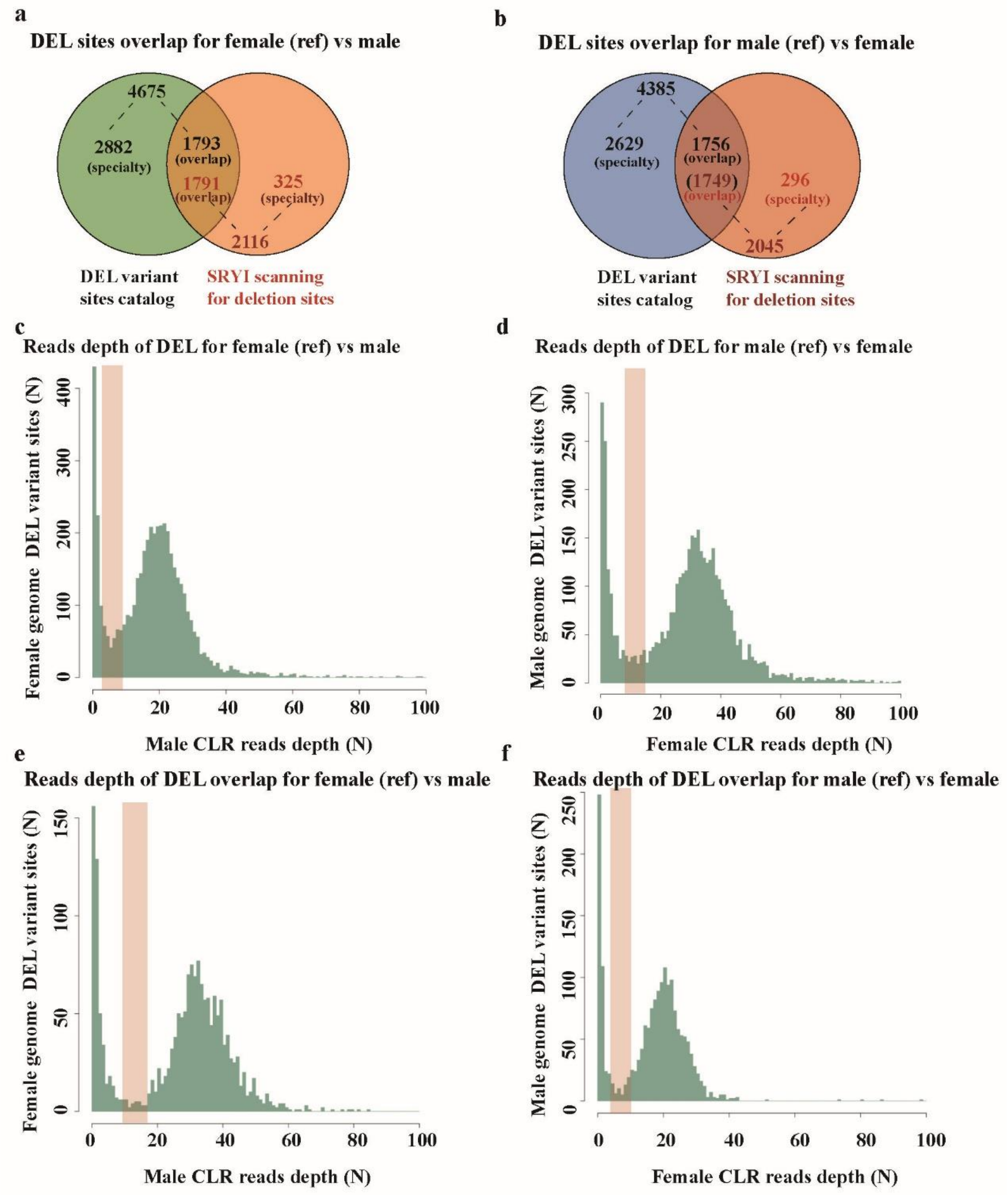

Figure 4: Intersection results of two variant site scanning methods and ranking of variant sites with long read depth frequency distributions

~~**Table 2.** Statistics of large-segment differential loci after primer design, e-PCR amplification, and~~

~~simulated amplification products and size screening.~~

~~Reference genome~~	~~Mutation type~~	~~Variant length (>100 bp)~~	~~High-throughput design of primers and e-PCR~~	~~Single band and suitable amplification size (100~2000 bp)~~
~~Female vs. Male~~	~~Insertion~~	~~4,428~~	~~5,044~~	~~2,791~~
	~~Deletion~~	~~4,691~~	~~1,628~~	~~854~~
~~Male vs. Female~~	~~Insertion~~	~~4,782~~	~~5,505~~	~~3,039~~
	~~Deletion~~	~~4,520~~	~~1,545~~	~~833~~

(a) Venn results of deletion locus data were obtained based on TBtools and SyRI software using the female fish genome as a reference, and 1,791 out of 2,116 deletion loci obtained based on SyRI software were shared deletion variant loci. (b) Utilizing the male fish genome as a point of reference for acquiring deletion site data in the context of Venn analysis, it was found that 1,749 out of the 2,045 deletion sites identified through the use of the SyRI software were common deletion variant sites. (c) Depth distribution of CLR reads in male fish using all female genomic deletion locus (＞100bp) information as a reference, with the area where high-quality male and female genetic marker information was located before the orange labeled trough position (~5.4). (d) The depth distribution of CLR reads in females was analyzed by utilizing male genomic deletion sites as a reference (>100bp), focusing on the region preceding the orange labeled trough position (~12.5) where high-quality male and female genetic marker information was present. (e) - (f) Depth distribution of CLR reads in males and females based on intersecting male and female genomic deletion site information (>100bp), respectively.

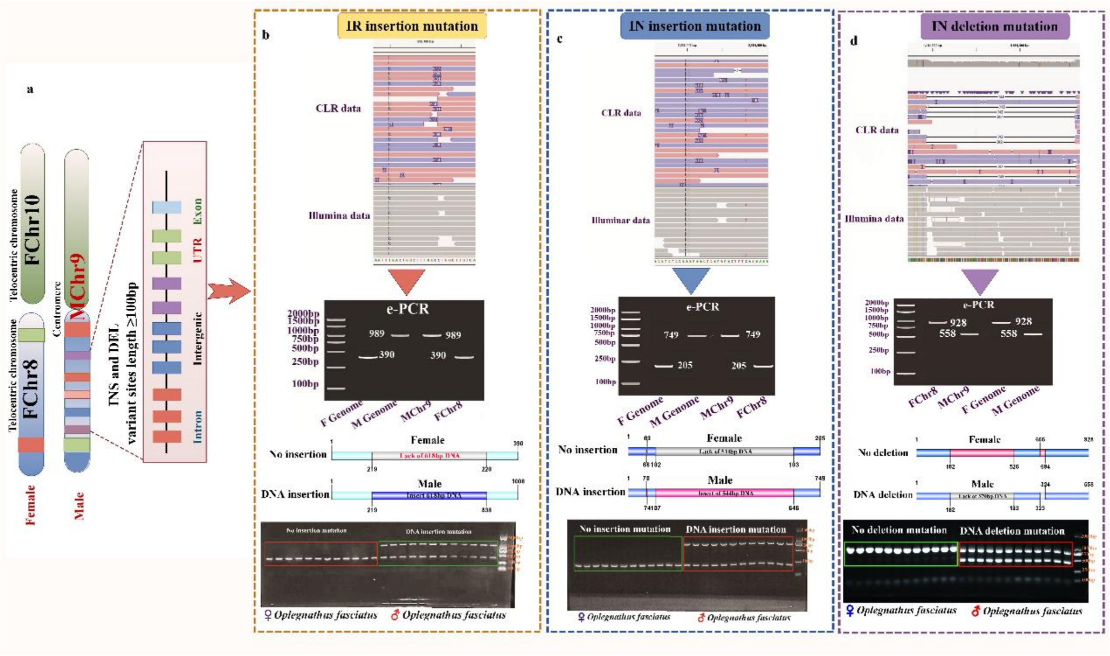

Figure 5: Representative genetic sex marker (insertion deletion variant sites) located in the intergenic region (IR), Intronic region (IN) of the *O. fasciatus* genome (STOSOd393e).

(a) Location of male and female sex markers in the male (MChr9) and female (FChr8) genomes. (b) Male and female sex genetic markers were located at loci in the intergenic region, using the female fish genome as a reference (Figure S2). Illumina and CLR clean data of bam comparison to the genome for insertion/deletion site validity detection. Red represented length long data from PacBio and gray represented was Illumina clean data. The e-PCR amplified bands were 390bp and 989bp in size, corresponding to DNA insertion and deletion patterns and agarose gel electrophoresis for identification of male and female genetically different populations. (c) By utilizing the female genome as a point of comparison, the genetic markers for male and female sexes were identified at specific insertion

sites within *nuf2* gene introns (Figure S3, Figure S4). The resulting e-PCR amplified bands, measuring 205bp and 749bp in size, were indicative of distinct DNA insertion patterns and were utilized in agarose gel electrophoresis for the purpose of distinguishing between male and female genetically differentiated groups. Males exhibited two amplified bands, while females exhibited only one. (d) By utilizing the female fish genome as a point of comparison, the genetic markers for male and female sexes were situated within the deletion sites located in the introns of genes (Figure S5, Figure S6). The amplified bands produced through e-PCR were observed to be 558bp and 928bp in length, aligning with the DNA deletion patterns and agarose gel electrophoresis maps utilized for distinguishing genetically distinct groups of males and females. Specifically, two bands were amplified in males, while only one band was observed in females.

Some figure (Figure 7), have unnecessary portion with alignment browser screen, it should be cropped or manually edited for better visualization.

Reply:

Thanks to your detailed advice, we cropped the resultant display image (Figure 7) after it appeared to be unnecessary when aligning the browser screen. At the same time, we processed all the other results that had similar problems.

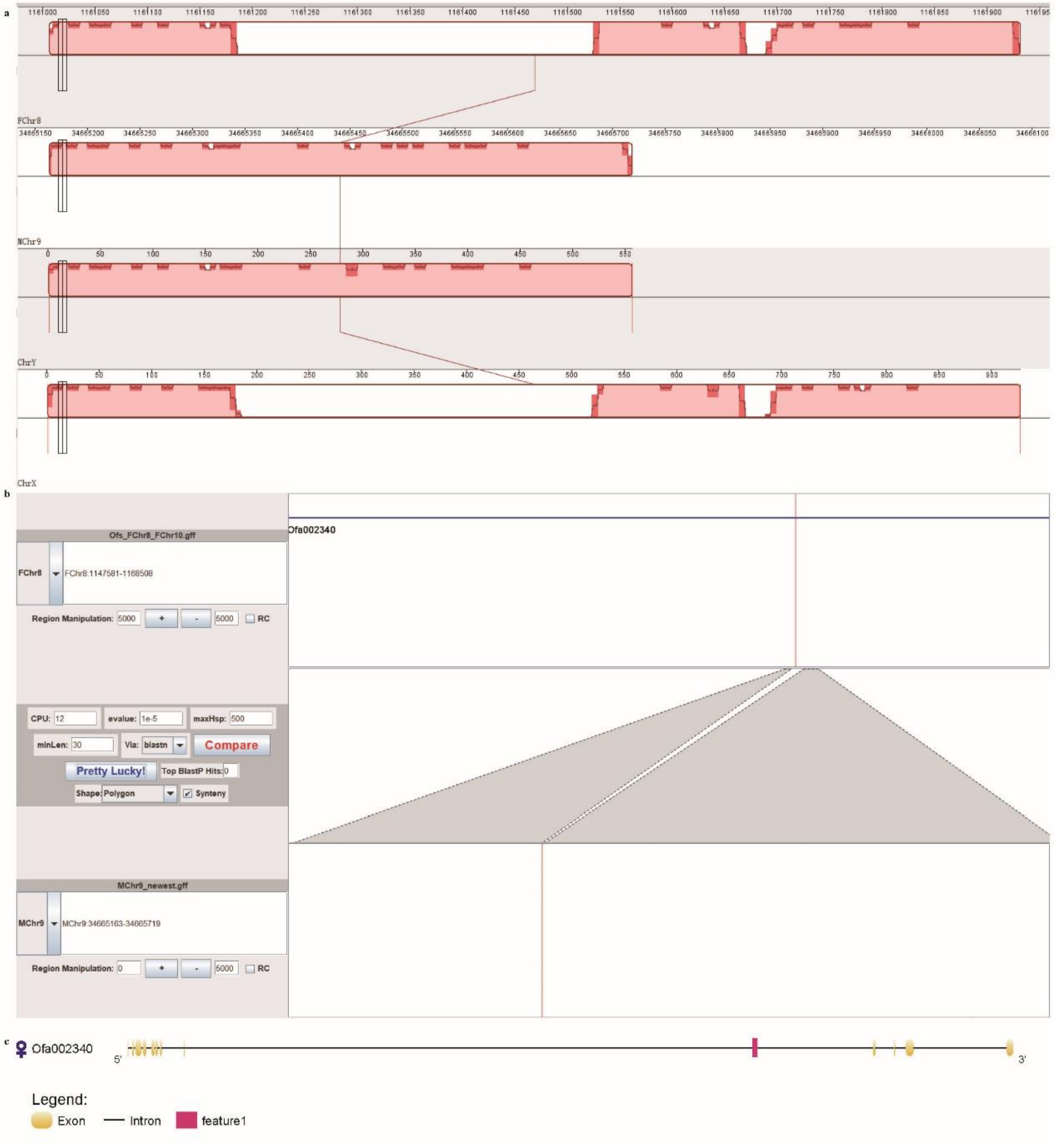
a
FChr8
MChr9
ChrY
ChrX
b
Ofs_FChr8_FChr10.gff
FChr8
FChr8:1147581-1168508
Region Manipulation:
RC
CPU:
evalue:
maxHsp:
minLen:
Via:
Compare
Pretty Lucky!
Top BlastP Hits:
Shape:
Synteny
MChr9_newest.gff
MChr9
MChr9:34665163-34665719
Ofa002340
c
Ofa002340
Legend:
Exon
Intron
feature1

Figure 7

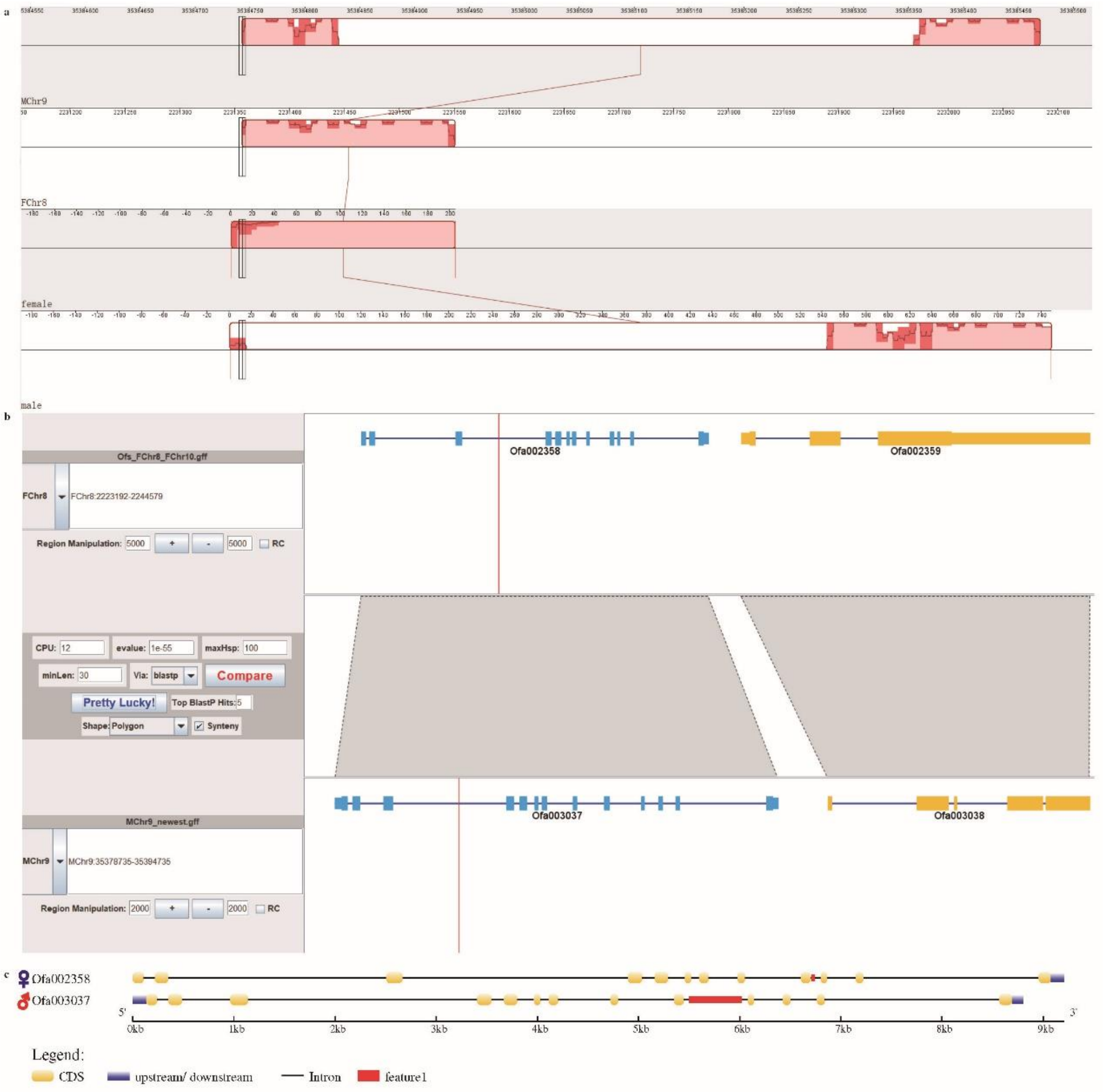

Figure 5

Minor comments:

The use of term "linear genomic information" is not very clear. Authors should clearly explain what they mean by linear genome? Whether this term mean genome assembly or comparative genomic analysis?

Reply:

Thanks to your professional advice, as you said the linear genome information was unclear and what they actually meant was the assembled genome, therefore, we have changed all the linear genome to assembled genome information in the manuscript according to your suggestion as follows

1) Line 197-198, we revised “sequencing technologies has made it possible to exploit high-throughput sex-specific markers in high-precision linear genomic data.” as “sequencing technologies has made it possible to exploit high-throughput sex-specific

markers in high-precision assembled genome data.”.

2) Line 219-220, we revised “constructed based on the linear genomic information of *O. fasciatus* by using bidirectional whole-genome sequence alignment..” as “constructed based on the assembled genome information of *O. fasciatus* by using bidirectional whole-genome sequence alignment”.

3) Line 231-232, we revised “First, a fast scanning method for female and male linear genomic variant loci was introduced…..” as “First, a fast scanning method for female and male assembled genome variant loci was introduced……”.

4) Line 598-599, we revised “By utilizing the linear genomic information of male and female individuals acquired through PacBio sequencing,” as “By utilizing the assembled genome information of male and female individuals acquired through PacBio sequencing,”.

On Line 147, "Using the difference database" should be corrected "Using different databases"

Reply:

Thanks to your detailed advice, we revised “Using the difference database,” as “Using the difference catalogs,”

Reviewer #2: Dear colleagues,

Than you very much for giving me the opportunity to read and comment the article of Xiao et al., entitled "A novel and scalable method for high-throughput and accurate exploitation of sex-specific markers: a case study on Oplegnathus fasciatus".

Most Eukaryotes do sex, and pluricellular organisms have highly differentiated organs, using sex determination systems and sex differentiation genes. In mammals, sex chromosomes are highly dimorphic, while most fish genomes show no distinction of their sexual chromosomes, and the genes responsible for the sex determination have been hard to characterize, sometimes being located in regions full of repeated sequences. Also, if sexual organs are easy to visualize and are used for chick sexing at just one day old in the egg industry (male chicks are culled as they are an unwanted by-product of egg production), such a selection can be harder to do in fish. Sexing the striped beakfish (Oplegnathus fascinatus), a major in Asian fish farm industry, is less easy, and the sexual differentiation can only be observed once the animals have passed the 500 g weight size, when males start to grow faster that females, bringing better yields. Among the different methods and technics used to

sex the individuals in the past, none were really conclusive. The authors propose to take advantage of the long reads provided with the third generation sequencing to access a class of mutations that are hundred-bp long insertions and deletions, not taken much into consideration before then. They were able to find occurrences of long indels that could be used as proxies of genetic sex markers.

In this work, the authors do present convincing technics and results. However, in that framework, it can be expected that the authors will also provide the weaknesses of this technic. Here, I see at least two points that should be considered. The first one is related to population genetics and the second should deal with the origin, if any, of these deletions and insertions, and their stability.

Starting with the second, what is the origin of such insertions and deletions? Could it be due to the mobilisation of a transposable element? Or any hot spot of deletion due for example to the formation of a fragile non-conventional B-DNA? Is there any repetitive sequence? Could these deletions or insertions occur frequently?

Reply:

We are very grateful to you for your constructive comments, which led us to think deeply and explore the origin of insertion deletion sites in large segments as well as the drivers, which would facilitate the extension and progression of *O. punctatus* from structural genomics to functional genomics. Therefore, we carefully examined the your suggestions and added variant site transposons and tandem repeat systematic analyses as follows:

"**To identify the origin of large segment insertion deletion sites, we evaluated whether the variant sites in the dataset were transposons by integrating RepeatModeler (v 2.0.1) and RepeatMasker (v 4.1.2) softwares. The results of**

transposon determination obtained by the two softwares were combined and the duplicates were removed for all transposon identification results. At the same time, we utilized TRF (v 4.09.1) software to evaluate whether the variant sites in the dataset were tandem repeats." Since deletion variants in large segments can more clearly show the differences between males and females, especially the read-return effect of the long read-length information of females with the male genome as the reference was more effective, we counted the sequence tagging sites (STS) attributed to transposons or tandem repeats of large segment deletions of males and females with the male and male genomes as the references, respectively.

Table 4 Statistics of transposons and tandem repeat sequences attributed to large deletion sequence tag sites obtained with male and female reference genomes

Reference genome	Mutation type	Transposon	Tandem Repeat	Total
Female vs. Male	Deletion	3,667 (78.44%)	1,574 (33.67%)	4,675
Male vs. Female	Deletion	3,380 (77.08%)	1,399 (31.90%)	4,385

The results showed that 78.44% (3667) of the deletion sequence tag sites with female as reference belonged to transposons and 33.67% (1574) of the STSs belonged to tandem repeat sequences. Meanwhile, the statistical results showed that 3380 (77.08%) of the missing sequence tag sites with male fish as reference belonged to transposons and 1399 (31.90%) of the STSs belonged to tandem repeats. As you predicted, at present, more than 77% of the large-band deletion sequence tag sites obtained from both female and male fish genomes as references are from transposons. The widespread presence of transposons in animal genomes and their important roles in the evolution of animal genomes, transposons can cause genome and chromosome evolution in a variety of ways, including insertion-induced gene mutations, gene transposons can cause genome and chromosome evolution in many ways, including insertion-induced gene mutation, gene backward movement, chromosome recombination, gene duplication and providing pivot points for ectopic recombination, etc., which is of great significance to adaptive gene evolution.

"The findings further indicated that a substantial proportion of deletion sequence tag sites referencing the female genome were situated in transposon regions (78.44%, n=3,667) and tandem repeat sequences regions (33.67%, n=1,574). Similarly, statistical analysis revealed that a notable percentage of deletion sequence tag sites referencing the male genome were linked to transposon regions (77.08%, n=3,380) and tandem repeat regions (31.90%, n=1,399) (Table 4)."

The RepeatModeler (v 2.0.1), RepeatMasker (v 4.1.2) and TRF (v 4.09.1) softwares run the following scripts:

RepeatModeler (v 2.0.1)

1) RepeatModeler-2.0.1/BuildDatabase -name mydb genome.fa > assembly_final.fasta.repeatmodeler.log

2) RepeatModeler-2.0.1/RepeatModeler -database mydb -pa 6 > run.out

3) mv ./RM_* ./result

4) cat result/consensi.fa.classified > final.library

RepeatMasker (v 4.1.2)

1) RepeatMasker-4.1.2/RepeatMasker/RepeatMasker -nolow -no_is -norna -parallel 2 -lib RepeatMasker-4.1.2/RepeatMasker/Libraries/RepeatMasker.lib genome.fa

2) RepeatMasker-4.1.2/RepeatMasker/RepeatMasker -nolow -no_is -norna -parallel 2 -lib final.library genome.fa

TRF (v 4.09.1)

1) trf-v4.09.1/trf genome.fa 2 7 7 80 10 50 2000 -d -h

The first question deals with the level of polymorphism of these indels. **How frequent are these insertions/deletions comparing different geographic populations**?

I understand that these two points are not the main interest of the present article. However, these evolutionary considerations would deserve to be taken into account, at least correctly addressed in the discussion.

Reply:

Thanks to your professional advice, this issue made us think seriously and deeply, on the basis of which we extended the results of the seven markers (four stable) that

had been validated in the original manuscript to validate the new nine genetic sex markers (six stable) in different geographic populations, and the population samples used were from the Zhoushan population and the Jiaonan population, and the results indicated that although the samples were from different geographic populations, large segments of the deletion sequence tag loci were stable across geographic populations with male and female differences as follows.

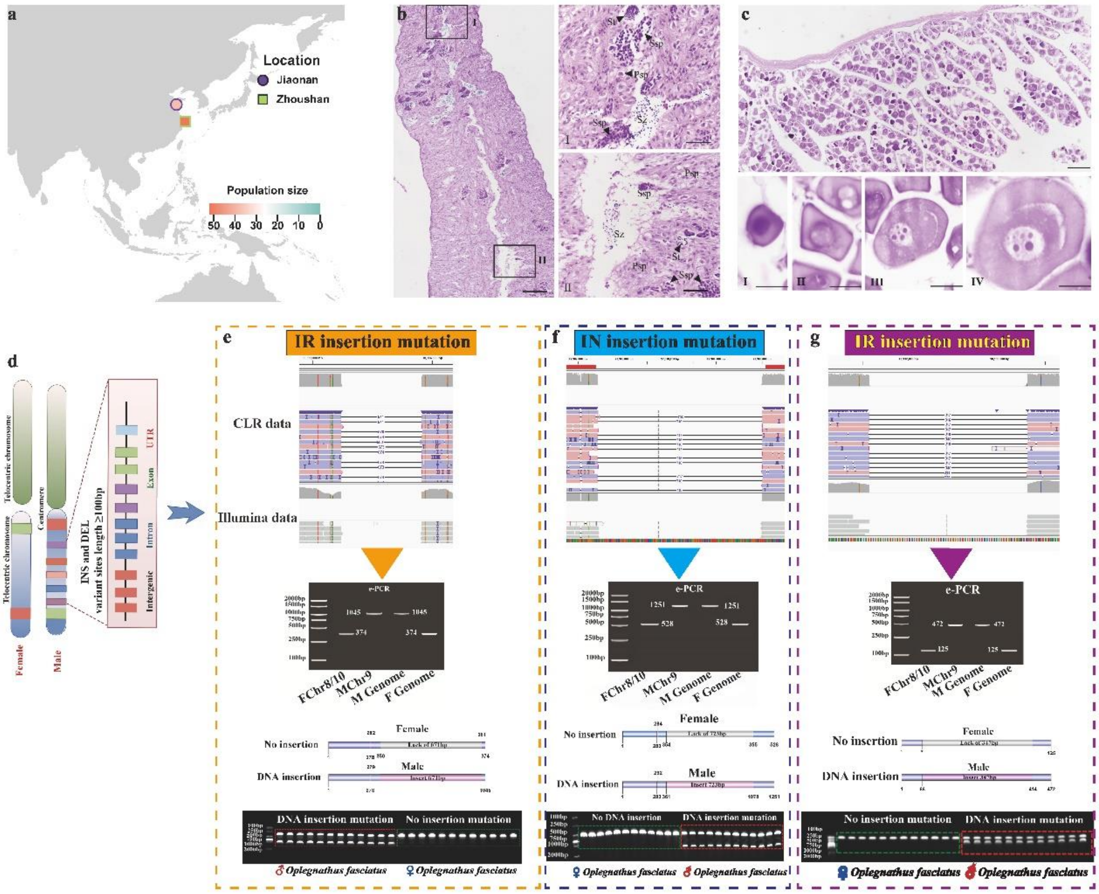

Validation of long read deletion sites for geographic populations using the male genome as a reference

As you mentioned large segments of insertion deletions are worth considering in terms of evolutionary considerations, especially since we found that most of the deletion sites belonged to transposons, which was the source of genetic diversity of the species and the origin of adaptive variation, so we reviewed the insertion deletion cases and discussed them in the Discussion section as follows:

"Variation in genome structure was intricately linked to human diseases, evolution, gene regulation, and species phenotypes in the biological realm, making it a prominent area of study in genomics research [79]. Previous studies have shown that DNA sequences with insertions and deletions undergo increased selective pressure [80-84].

The presence of deletions and insertions in DNA sequences, regardless of their location in noncoding or coding regions, inevitably influences the functionality of the original sequence to varying extents [79]. Furthermore, evidence suggested that the length and placement of insertions were not random but rather heavily influenced by the adjacent DNA sequence [80-82, 85, 86]. Prior research has indicated that insertion deletion variants accounted for 16%, 24%, 13%, 25%, and 15% of genomic genetic polymorphisms in various organisms, including *Drosophila melanogaster*, *Caenorhabditis elegans*, mouse, human, and *Arabidopsis thaliana* [34-36, 87-94]. Recent advancements in long length sequencing technology have allowed for a more precise examination of human genetic variation, revealing that insertions and deletions collectively contribute to a high portion (13.8%, 11.1%) of genetic polymorphisms in the human genome [90-94]. The focus of genomic structural variation studies has predominantly been on single nucleotide polymorphisms (SNPs) and insertions/deletions (InDels) of less than 50 base pairs due to the limitations of high-throughput sequencing technologies for short read lengths. However, larger genomic structural variants, which encompassed genetic variations not fully captured by SNPs and InDels, were closely linked to biological phenotypes and might significantly influence phenotypic traits, disease susceptibility, and adaptive capacity [79]. The utilization of long-read length sequencing technologies such as PacBio and ONT has enabled the generation of extended DNA sequences, thereby enhancing the assembly of diploid genomes of superior quality and the identification of structural variants (SVs) across substantial genomic regions [95]. Prvious research has documented seven deletions (>100bp) within approximately 700bp large segments of the *ZmBAM1d* gene, demonstrating a positive association with maize kernel weight. These deletions were consistently inherited in high-yielding maize lines [96]. In mammalian studies, a deletion of approximately 40Mb on chimpanzee chromosome 2, as compared to humans and gorillas within the great ape family, has been identified, leading to stable genetic distinctions among species [97]. Similar results have been documented in *Astyanax mexicanus*, *Gasterosteus aculeatus*, racehorses, and dogs [98-101]. The emergence of long read-length sequencing technology has propelled large fragment genomic structural variation to the forefront of genetic variation research, particularly in investigations of species' adaptive evolution [95, 96, 102, 103]. The findings of this study indicated that insertions and deletions identified using the female and male *O. fasciatus* genomes as references constituted 26.96% and 26.97% of the genetic

polymorphisms, respectively. Our research has contributed new insights into the prevalence of large-segment insertion-deletion events for *O. fasciatus*, indicating that these events predominantly occurred in the intergenic region in 58.09%~66.33% of instances, with the intronic region following closely at 31.65%~40.35% of cases (Figure 2, Figure S1 Table 2). We also observed a higher proportion of deletion sites compared to insertion sites. A similar study conducted on the β-fibrinogen intron of birds reported similar findings [104]. Furthermore, this study presented novel findings indicating that over 77% of the identified large deletion sequence tag sites in the genomes of both female and male *O. fasciatus* were attributed to transposons (Table 4). It was evident that transposons were prevalent in the animal genome and significantly contribute to its evolutionary processes. Previous research has demonstrated that a significant portion (69.5%) of genomic structural variation in large segments of tomato was situated within the transposon region, which was closely linked to desirable traits in tomato [105]. Comparable studies have also been conducted on gene regulation in human brain diseases and genomic structural variation in evolutionary distinct phenotypes of great apes [103, 106]. Transposons have the potential to drive genome and chromosome evolution through various mechanisms such as insertion-induced gene variation, gene retrotransposition, chromosome recombination, gene duplication, and serving as sites for ectopic recombination. These findings underscored the importance of transposons in facilitating adaptive evolution for *O. fasciatus*. The presence of large fragments of insertion deletion sequence marker sites was frequently influenced by natural selection pressures, with their formation being intricately linked to adjacent DNA sequences and commonly correlated with characteristic phenotypic traits and aberrant gene regulation [83, 84, 102, 103, 105, 106]. Specifically, the manifestation of albino symptoms in dogs resulting from a 965-kb deletion on chromosome 18 exhibited stable inheritance patterns, while a 4.8 Mb deletion variant on the chromosome of male domestic chickens similarly displayed heritability across subsequent generations [100, 107]. Consistent inheritance of variants within populations has also been documented in academic research on maize (*ZmBAM1d*), *Astyanax mexicanus*, *Ailuropoda melanoleuca* (*Bace2*), great apes (*acan*), and human diseases such as hypertension (*ace*) [96, 98, 103, 108]. The findings from the population genetic sex marker amplification analysis conducted in this study indicated that the insertion-deletion markers exhibited stability in both male and female populations of *O. fasciatus*. Additionally, analysis of gonadal histology and sex-validated marker data

from various geographic populations demonstrated consistent inheritance of large segments of the insertion deletion markers in individuals of both sexes across different geographic regions. This observation provided additional evidence to support the theory that large-segment insertions and deletions are not a random occurrence but rather influenced by natural selection pressures [80, 82-84]. Based on the present findings, the stability of large segment insertion and deletion in sequence tag sites was evident. However, this should not be taken to imply that all sequence tag sites exhibit this characteristic. A comprehensive evaluation of the evolutionary rate of sequence tags and the expansion of variant marker detection are necessary for ongoing research in this area."

A (non exhaustive) list of suggestions:

Title. I'd suggest to shorten it and use the vernacular name, at least, or explicitly say that this species is a fish.

Reply:

Thanks to your professional advice, we have modified the title of our paper as follows：

"**Innovative approach for high-throughput exploiting sex-specific markers in japanese parrotfish *Oplegnathus fasciatus***".

Abstract, it is not mentioned on what taxonomic species this article is dealing with.

Reply:

Thanks for your detailed advice, we added the taxonomic information for *Oplegnathus fasciatus* in front of "The criteria for rapid identification….." as follows:

"**The Japanese parrotfish (*Oplegnathus fasciatus*), belonging to the Oplegnathidae family within the Centrarchiformes order, holds significant economic value as a rocky reef fish indigenous to East Asia.** The criteria for rapid identification of male and female differences in *O. fasciatus* were established through agarose gel electrophoresis, which revealed two amplified bands for males and one amplified band for females."

Figures: font size is too small.

Reply:

Thanks for your detailed advice, we have re-enlarged the font in the image as follows:

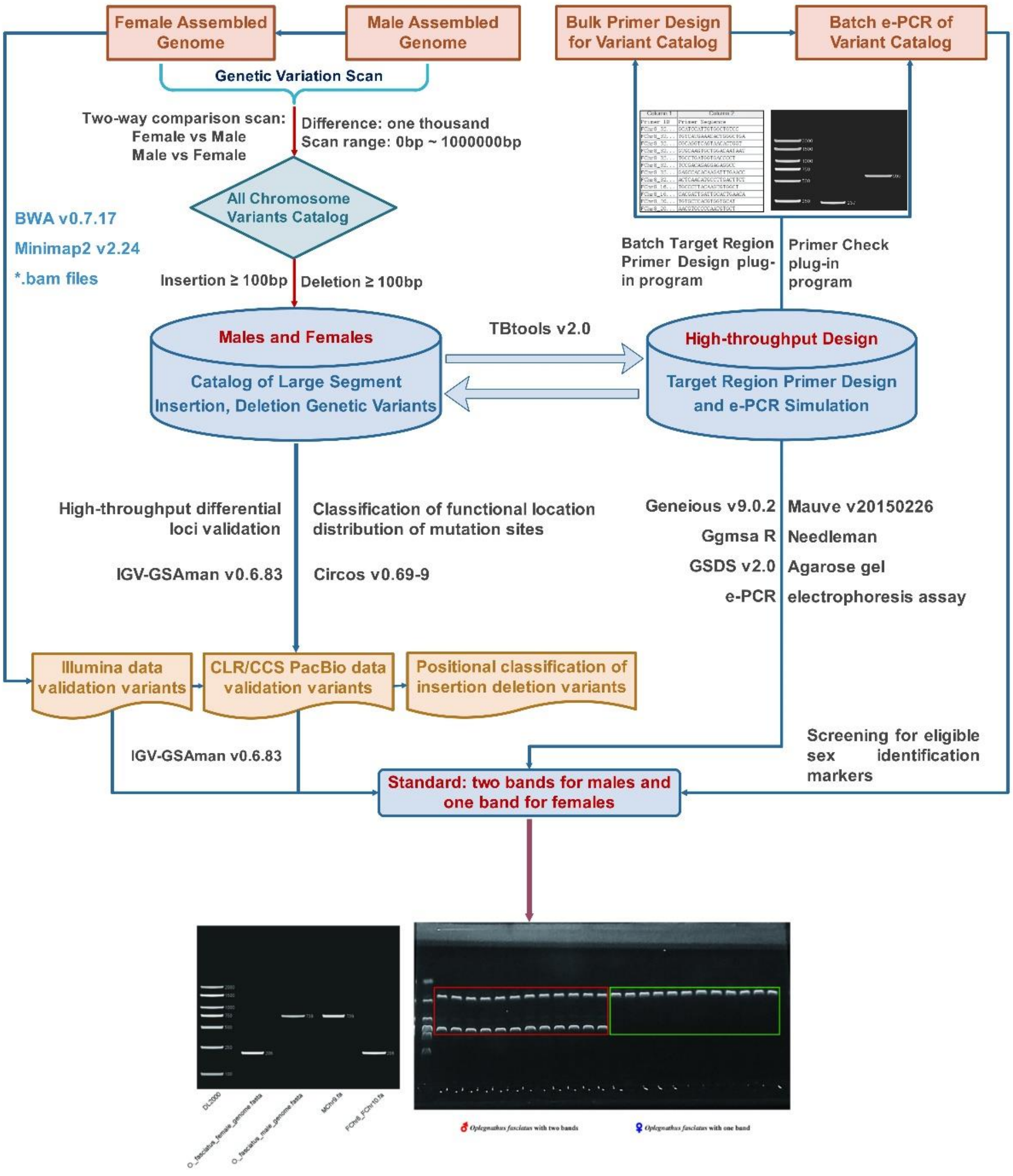

Female Assembled Genome
Male Assembled Genome
Bulk Primer Design for Variant Catalog
Batch e-PCR of Variant Catalog
Genetic Variation Scan
Two-way comparison scan: Female vs Male Male vs Female
Difference: one thousand
Scan range: 0bp ~ 1000000bp
All Chromosome Variants Catalog
BWA v0.7.17
Minimap2 v2.24
*.bam files
Insertion ≥ 100bp
Deletion ≥ 100bp
Batch Target Region Primer Design plug-in program
Primer Check plug-in program
Males and Females
Catalog of Large Segment Insertion, Deletion Genetic Variants
TBtools v2.0
High-throughput Design
Target Region Primer Design and e-PCR Simulation
High-throughput differential loci validation
Classification of functional location distribution of mutation sites
IGV-GSAman v0.6.83
Circos v0.69-9
Geneious v9.0.2
Mauve v20150226
Ggmsa R
Needleman
GSDS v2.0
Agarose gel
e-PCR
electrophoresis assay
Illumina data validation variants
CLR/CCS PacBio data validation variants
Positional classification of insertion deletion variants
IGV-GSAman v0.6.83
Screening for eligible sex identification markers
Standard: two bands for males and one band for females

Figure 1

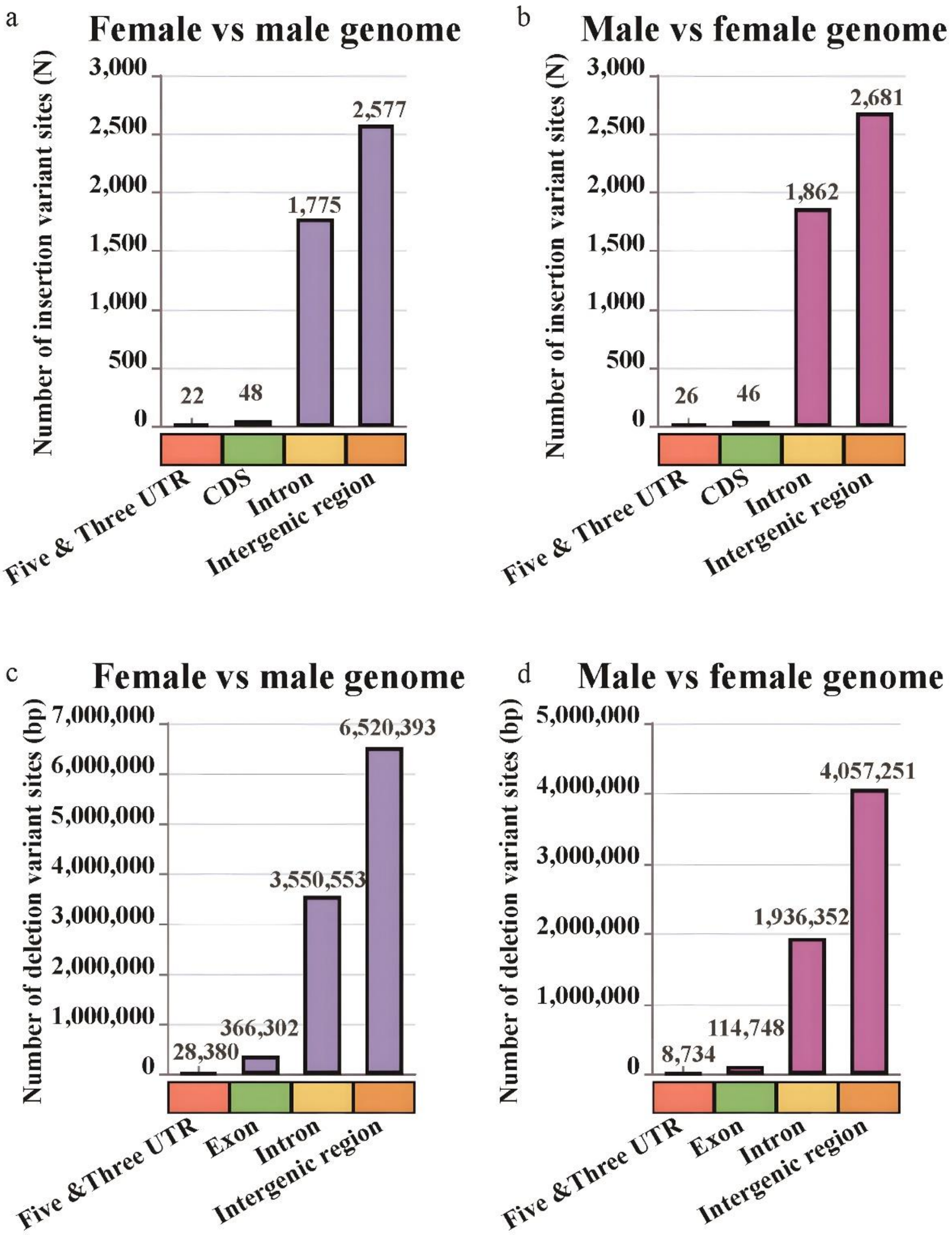
a
Female vs male genome
Number of insertion variant sites (N)
3,000
2,500
2,000
1,500
1,000
500
0
22
48
1,775
2,577
Five & Three UTR
CDS
Intron
Intergenic region
b
Male vs female genome
Number of insertion variant sites (N)
3,000
2,500
2,000
1,500
1,000
500
0
26
46
1,862
2,681
Five & Three UTR
CDS
Intron
Intergenic region
c
Female vs male genome
Number of deletion variant sites (bp)
7,000,000
6,000,000
5,000,000
4,000,000
3,000,000
2,000,000
1,000,000
0
28,380
366,302
3,550,553
6,520,393
Five &Three UTR
Exon
Intron
Intergenic region
d
Male vs female genome
Number of deletion variant sites (bp)
5,000,000
4,000,000
3,000,000
2,000,000
1,000,000
0
8,734
114,748
1,936,352
4,057,251
Five &Three UTR
Exon
Intron
Intergenic region

Figure 2

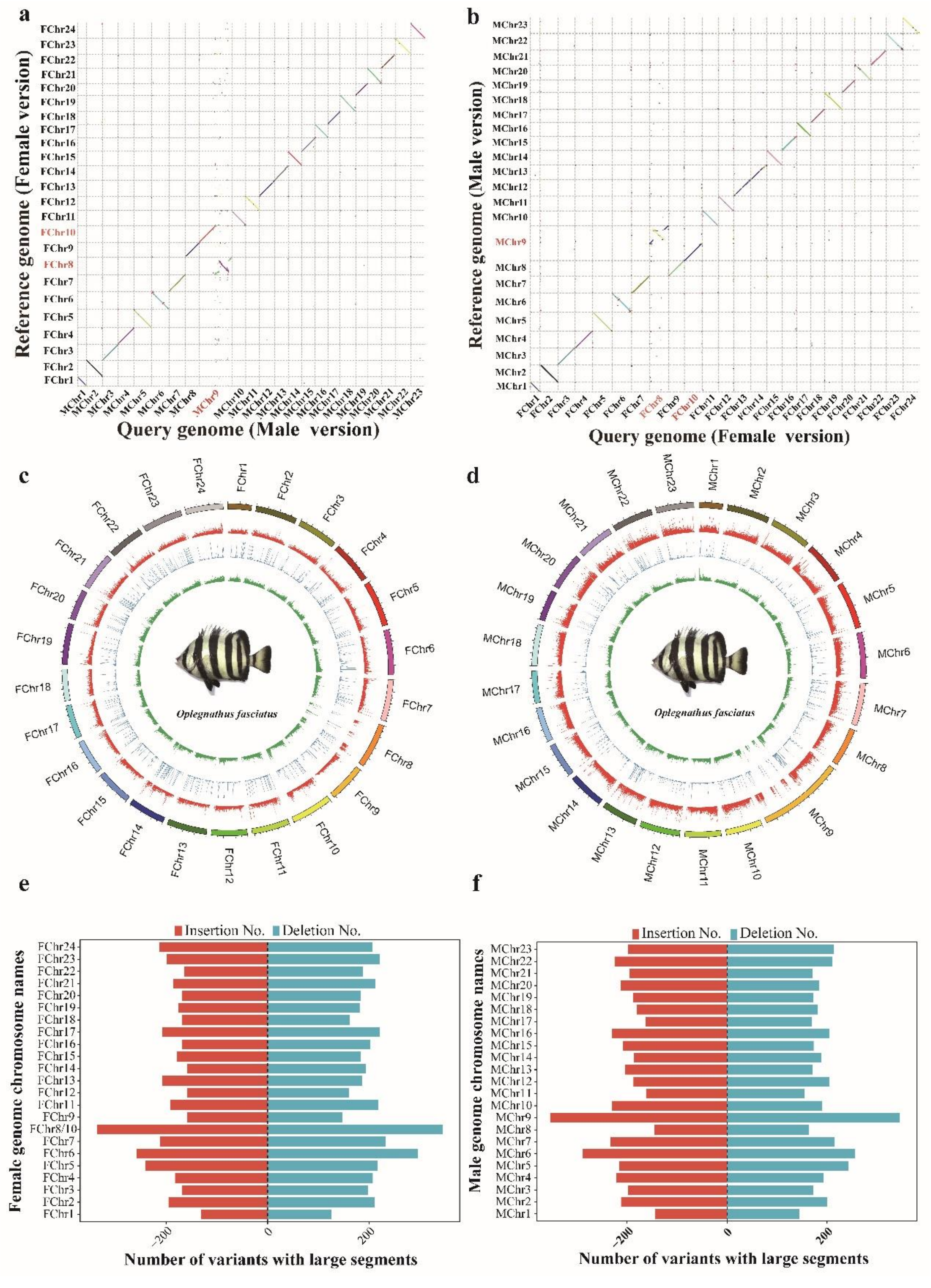
a
b
c
d
e
f
Reference genome (Female version)
Query genome (Male version)
Reference genome (Male version)
Query genome (Female version)
Oplegnathus fasciatus
Oplegnathus fasciatus
Insertion No.
Deletion No.
Female genome chromosome names
Male genome chromosome names
Number of variants with large segments
Number of variants with large segments

Figure 3

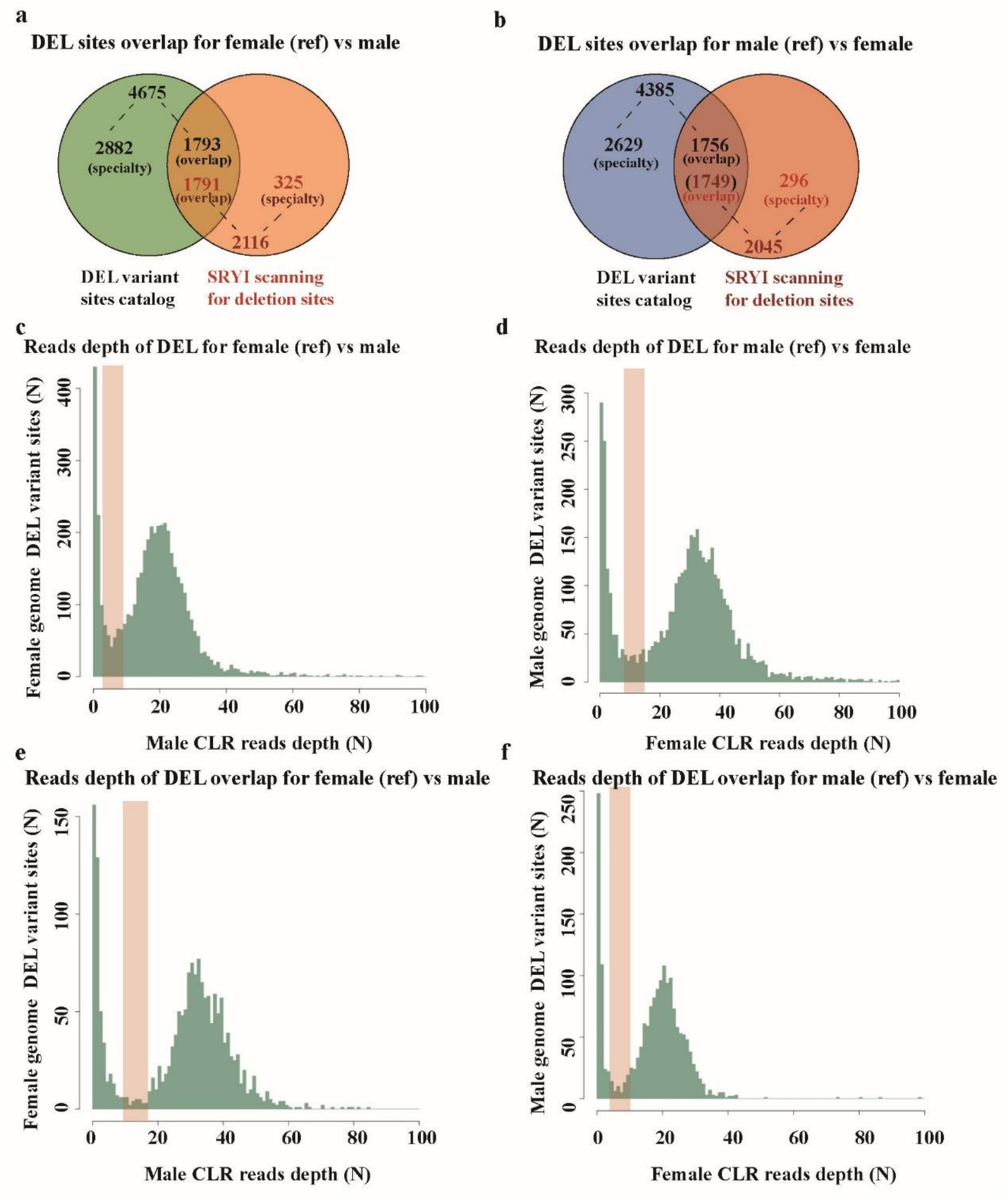

Figure 4

Fig. 4, 5 and others would gain in being more didactical and summarized in one single image. These detailed information would be more valorized as

Reply:

Thanks to your constructive advice, we strongly agree with your comment that this will make the whole study significantly synthesized and more readable. In addition, we have placed images of the results before the merger into the Supplementary Material file, which allows the reader to read through the results at a glance, but also to look at

the detailed results through the Supplementary Material.

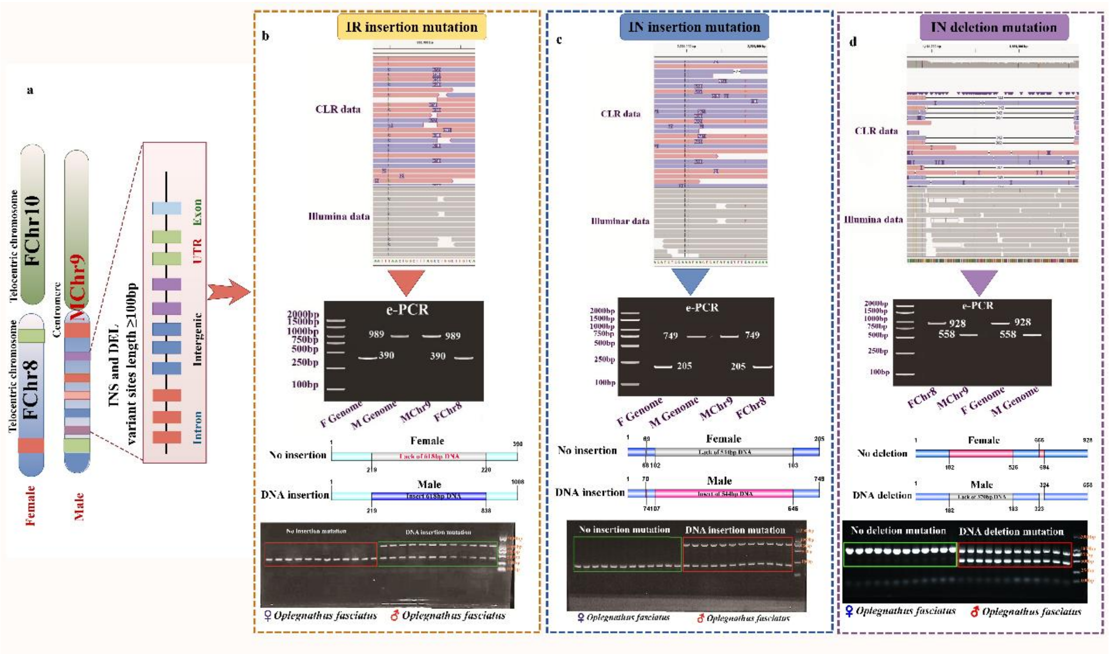

Figure 5

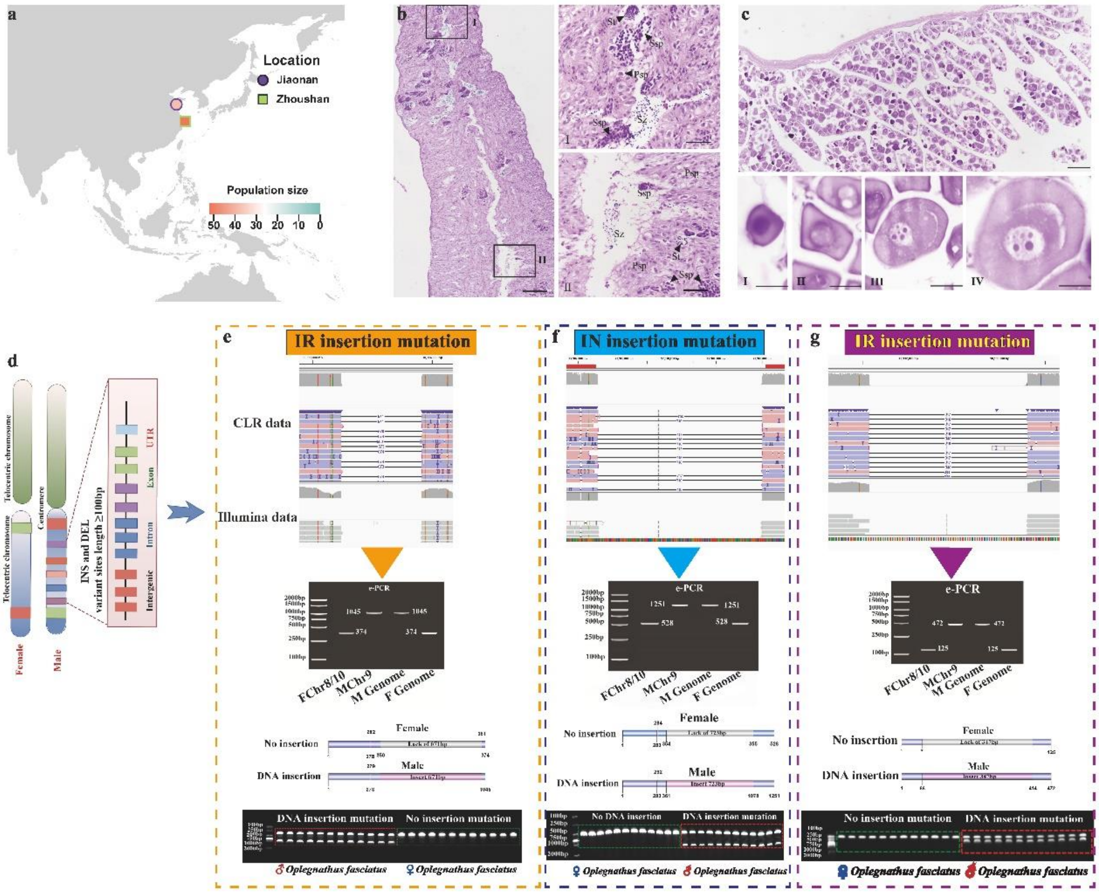

Figure S9

Tables: position numbers using decimal tabulations and homogenize filled cells, text shouldn't be in the "justify" format
L 316 "bp base" is confusing.

Reply:

Thanks for your detailed advice, we revised the "a 518 bp base sequence was……" as "a 518 bp DNA fragment was……". We have read through the entire text and have made the following changes where there are similar errors:

1) Line 507, we revised "……a 599 bp base sequence" as "……a 599 bp DNA fragment".

2) Line 547, we revised "……males were missing a 343 bp base sequence" as "……males were missing a 343 bp DNA fragment".

3) Line 556, we revised "with deletion of a 299 bp base sequence" as "with deletion of a 299 bp DNA fragment".

4) Line 557, we revised "multifragmented base sequence in males" as "DNA multi-fragments in males"

L 486-488: This claim cites no reference. Why large sequences shouldn't evolve at random, unless of course, if they are directly linked to functional genes involved in various, but essential processes?

Reply:

Thanks for your detailed advice, previous research has indicated that insertion deletions are not randomly distributed, but rather are linked to specific sequence characteristics at their boundaries. Deletion events occur when the replicon skips certain nucleotides during replication, with sequences possessing high GC content showing a higher incidence of deletions. This may be attributed to the formation of unique structures in high GC regions, increasing the likelihood of sliding strand mismatch events. Similar patterns were observed in deletion sequences within human genes. Insertion variants arise from errors in DNA replication, where a segment of DNA is mistakenly duplicated and inserted into the genome. This process necessitates the ability to integrate a previously copied DNA fragment. We have added the references and make discussion as follows: "This observation provided additional evidence to support the theory that large-segment insertions and deletions are not a random occurrence but rather influenced by natural selection pressures [80, 82-84]. Based on the present findings, the stability of large segment insertion and deletion in sequence

tag sites was evident. However, this should not be taken to imply that all sequence tag sites exhibit this characteristic. A comprehensive evaluation of the evolutionary rate of sequence tags and the expansion of variant marker detection are necessary for ongoing research in this area."

Fig 5d check spelling of nucleotide (here spelled "nucelotide"). Same for Fig. S2e.

Reply:

Thanks for your detailed advice, We did misspell the word in the image result by looking at it, we co-ordinated all the results and made corrections for all the similar errors as follows：

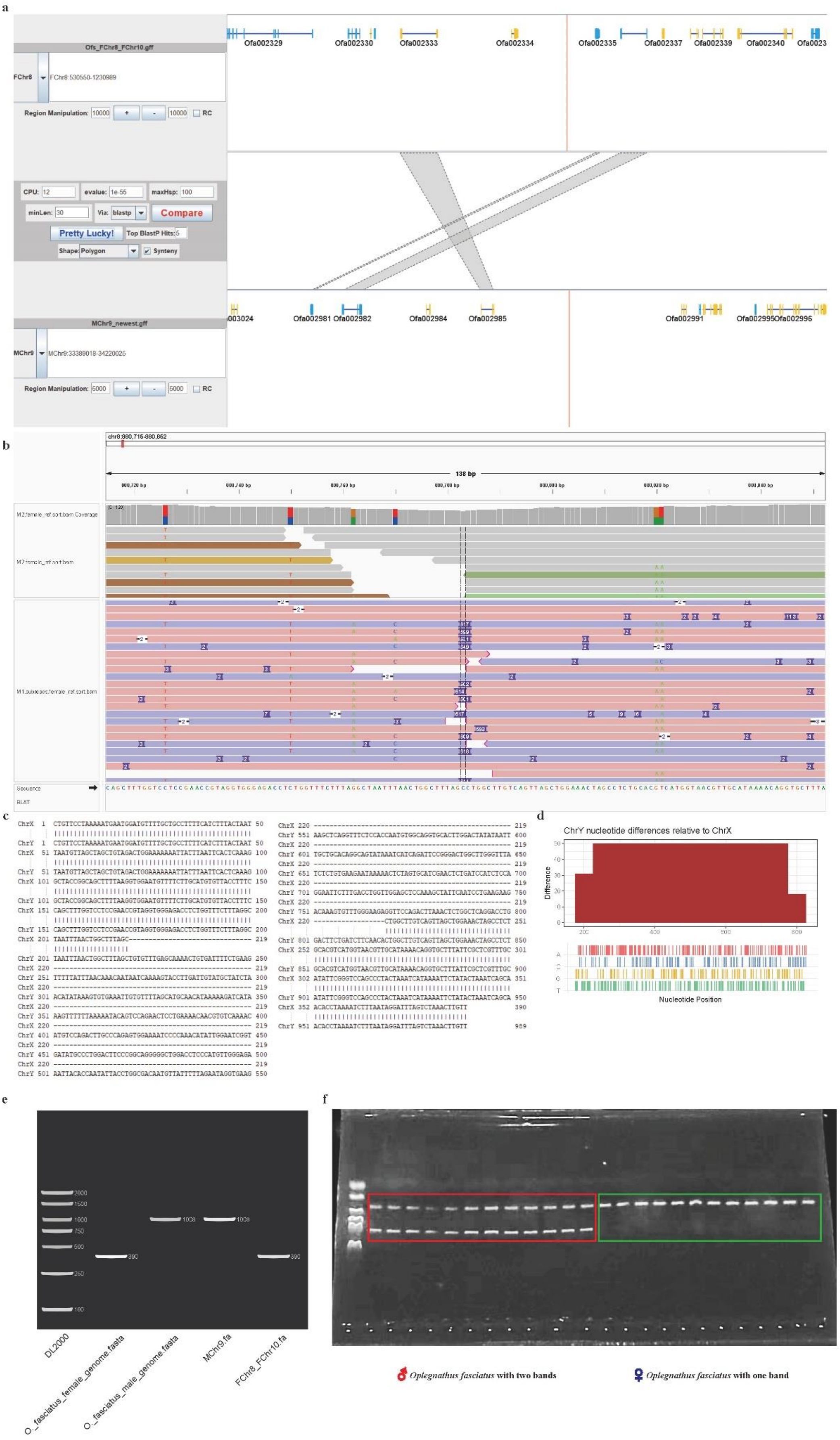
a
Ofs_FChr8_FChr10.gff
FChr8
FChr8:530550-1230989
Region Manipulation:
RC
CPU:
evalue:
maxHsp:
minLen:
Via:
Compare
Pretty Lucky!
Top BlastP Hits:
Shape:
Synteny
MChr9_newest.gff
MChr9
MChr9:33389018-34220025
Ofa002329
Ofa002330
Ofa002333
Ofa002334
Ofa002335
Ofa002337
Ofa002339
Ofa002340
Ofa002981
Ofa002982
Ofa002984
Ofa002985
Ofa002991
Ofa002995
Ofa002996
b
138 bp
M2.female_ref.sort.bam Coverage
M2.female_ref.sort.bam
M1.subreads.female_ref.sort.bam
Sequence
BLAT
c
d
ChrY nucleotide differences relative to ChrX
Difference
Nucleotide Position
e
DL2000
O._fasciatus_female_genome.fasta
O._fasciatus_male_genome.fasta
MChr9.fa
FChr8_FChr10.fa
f
Oplegnathus fasciatus with two bands
Oplegnathus fasciatus with one band

Figure 4

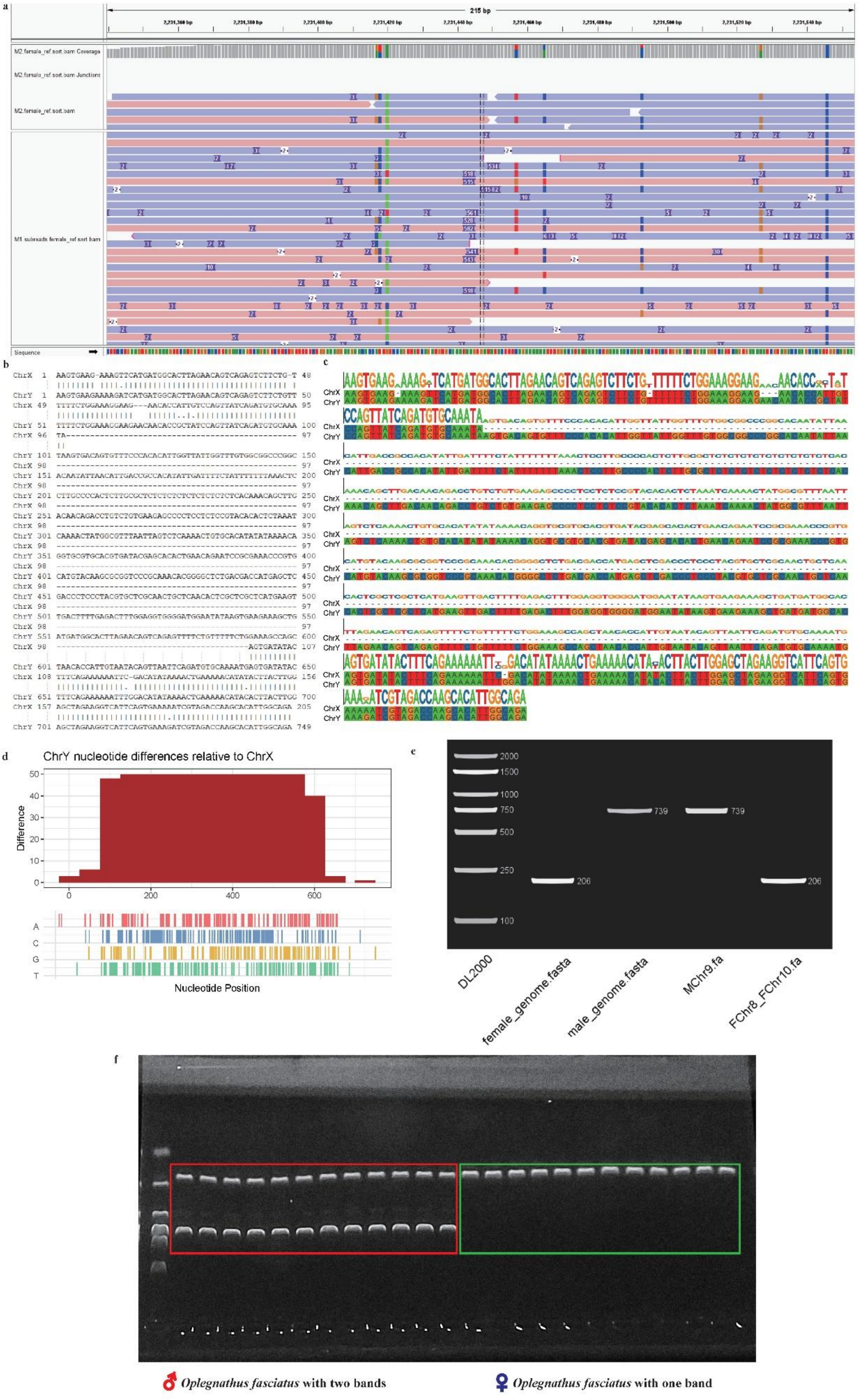
a
215 bp
M2.female_ref.sort.bam Coverage
M2.female_ref.sort.bam Junctions
M2.female_ref.sort.bam
M1.subreads.female_ref.sort.bam
Sequence
b
c
ChrX
ChrY
d
ChrY nucleotide differences relative to ChrX
Difference
Nucleotide Position
e
2000
1500
1000
750
500
250
100
739
206
DL2000
female_genome.fasta
male_genome.fasta
MChr9.fa
FChr8_FChr10.fa
f
♂ Oplegnathus fasciatus with two bands
♀ Oplegnathus fasciatus with one band

Figure 6

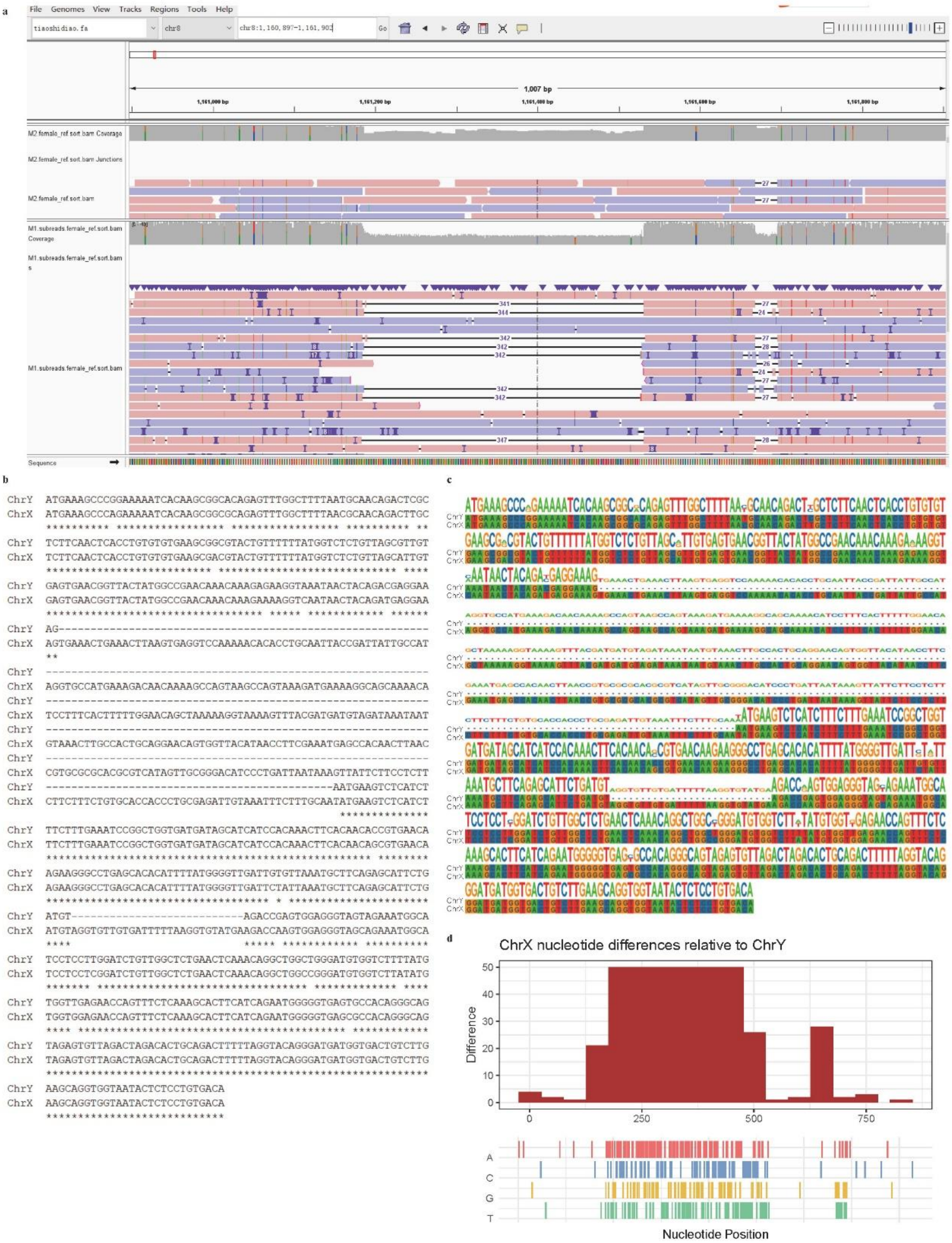

Figure 8

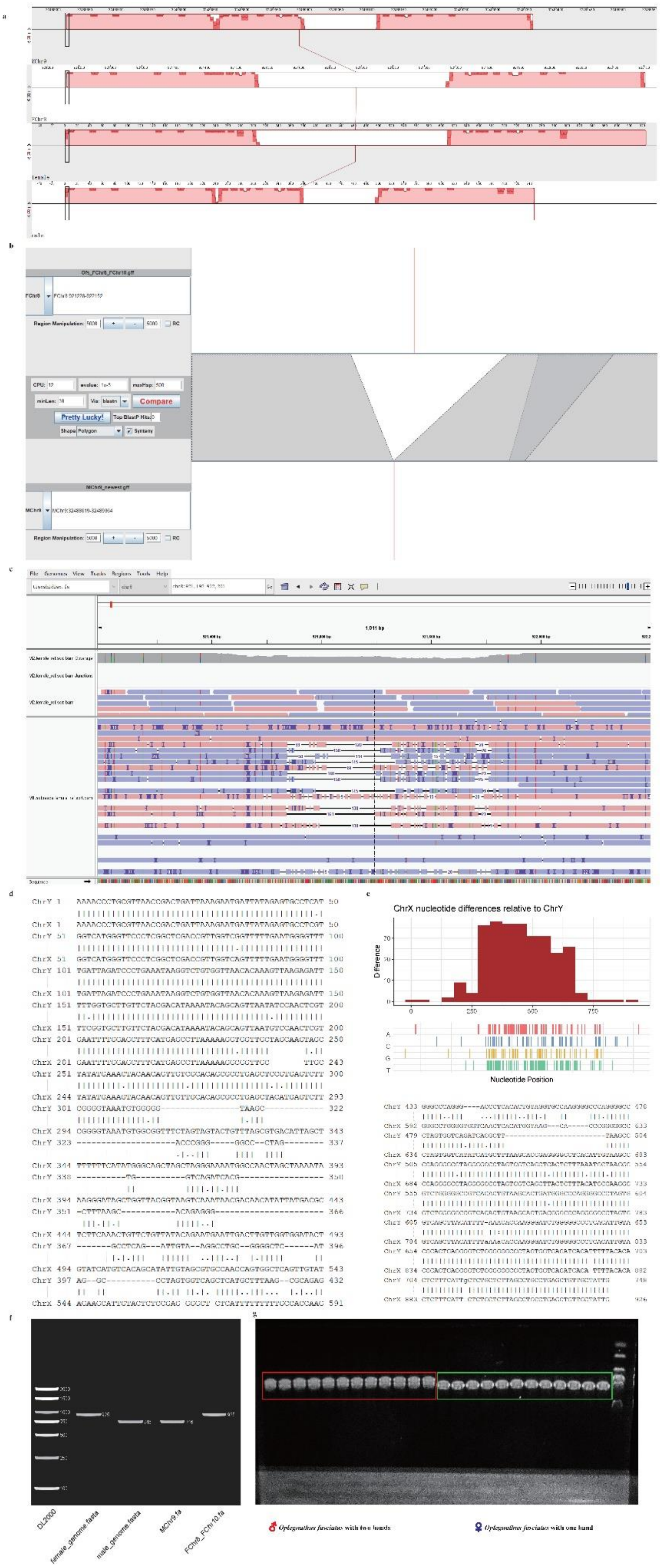
a
b
Compare
Pretty Lucky!
c
d
e
ChrX nucleotide differences relative to ChrY
Difference
Nucleotide Position
f
g
DL2000
female_genome.fasta
male_genome.fasta
MChr9.fa
FChr8_FChr10.fa
Oplegnathus fasciatus with two bands
Oplegnathus fasciatus with one band

Figure S1

Fig8 b, identity is not correctly positioned below line "chrY" 380

Reply:

Thanks for your detailed advice, we re-did the sequence comparison and obtained the following results.

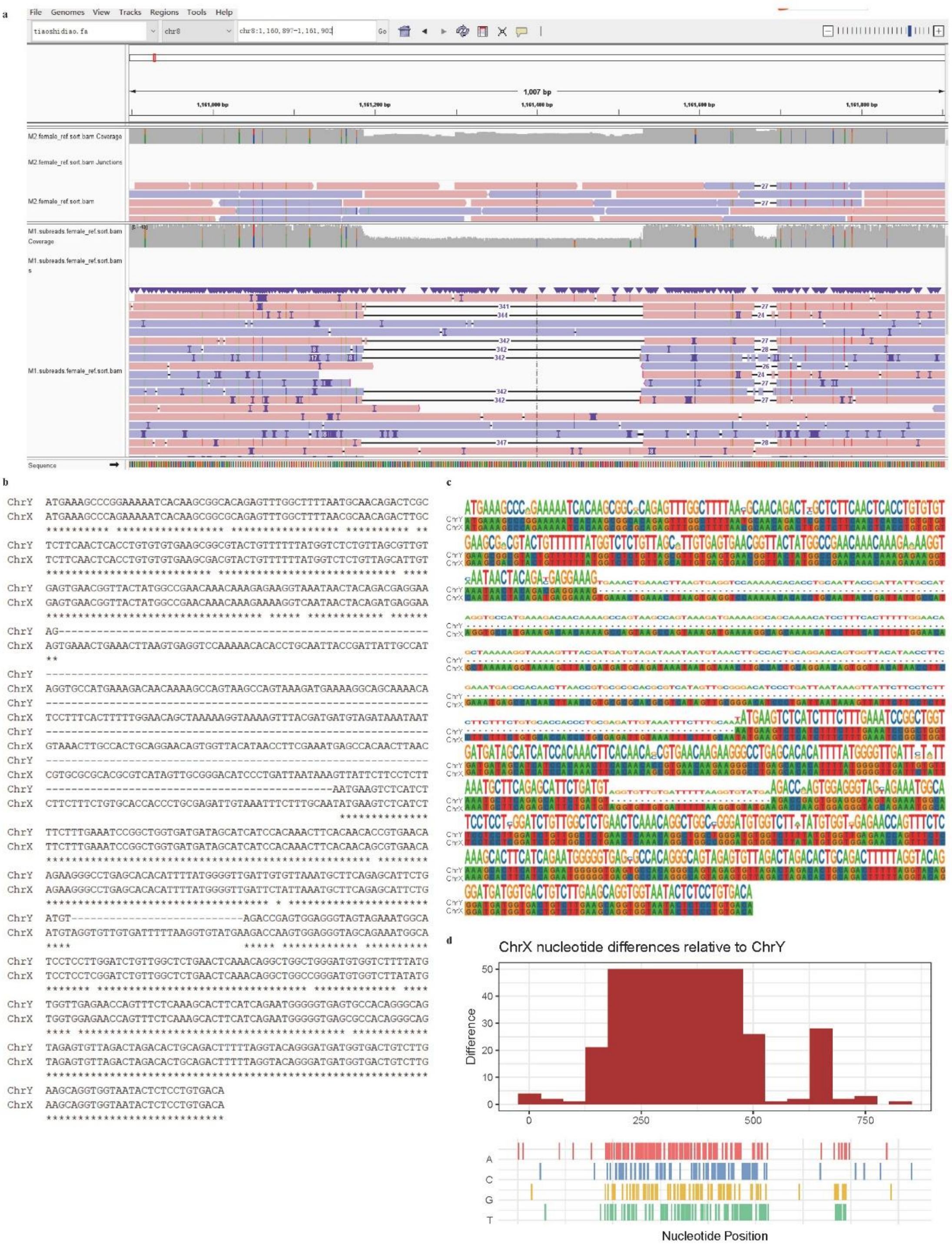

Figure 8

Mat&Meth

Maybe worth to subdivide into paragraphs (and titles).

Reply:

Thanks for your constructive suggestion, we the authors of the paper have agreed that this was very necessary work, so we have added subheadings to the Materials and Methods section as follows:

Sample collection, sequencing and genome quality

Large fragment variant loci scanning and identification

Write large number with separation, being coma (as in Result section) or small space

Reply:

Thanks for your detailed suggestion, we have systematically revised the paper where relevant by comma separating all references to large numbers as follows:

1) Line 172-173, we revised "(diff: oneInThousand, VarRange: 0~1000000 bp, BatchSize: 500 bp, Min Align Length for Cov Calc 10000 bp, Min Align Length for Var Calling: 50000 bp)" as "(diff: oneInThousand, VarRange: 0~1,000,000 bp, BatchSize: 500 bp, Min Align Length for Cov Calc 10,000 bp, Min Align Length for Var Calling: 50,000 bp)".
2) Line 301, we revised "amplicons of 390 bp and 1008 bp, respectively" as "amplicons of 390 bp and 1,008 bp, respectively".
3) Line 308, we revised "with the results of e-PCR corresponding to two bands (390 bp and 1008 bp)" as "with the results of e-PCR corresponding to two bands (390 bp and 1,008 bp)".

L411: "REREgulation" is that correct?

Reply:

Thanks for your detailed suggestion, we revised "reregulation of structural morphogenesis" as "regulation of structural morphogenesis".

References output has not been scrutinized. For example, almost no Journals are cited!!!

Species names should also be italicized in the list of references!

Reply:

Thanks for your detailed suggestion, We re-imported the reference format as requested and italicized the Latin of the species.

Reviewer #3: This manuscript describes a computational approach to develop PCR markers for sex chromosomes in a fish with a complex genetic sex determination system. A few of these markers have been validated using PCR on a panel of 24 fish for which the sex was determined by dissection, showing an excellent correspondence.

I have found some issues that I would ask the authors to address:

1. It is not entirely clear to me what data the study is based on. **The two genome assemblies are not referenced by accession number, and the validation data (PacBio) are not described. Are these the data from the original genome assemblies?**

Reply:

Thanks for your constructive suggestion, we found that the changes you suggested were necessary additions to our paper, which would have resulted in a fuller and richer manuscript, so we have added information about the genome based on your suggestions as follows:

Table 1 Parameters for genome sequencing and assembly quality assessment of chromosome-level genomes of male and female *O. fasciatus*

Illumina platform (Short-read sequencing data survey)		
	Male *O. fasciatus*	Female *O. fasciatus*
Reads number	432,091,798	613,163,122
Raw data (bp)	63,910,932,339	90,702,298,813
Clean data (bp)	56,617,669,600	80,887,039,002
Read length (bp)	148, 147	148, 147
Q20 (%)	97.9, 97.3	98.0, 97.1
Q30 (%)	93.8, 92.0	94.1, 91.4
PacBio Sequel platform (Long-read PacBio sequencing data)		
	Male *O. fasciatus*	Female *O. fasciatus*
Polymerase read number	3,674,737	4,760,021
Polymerase read bases (bp)	39,844,228,109	62,877,786,278
Average polymerase read	10,847	13,219

length (bp)		
Polymerase read N50 (bp)	18,850	22,964
Subreads number	4,711,720	6,689,662
Total bases of subreads (bp)	39,793,172,442	62,783,888,050
Average subread length (bp)	8,448	9,397
Length of genome (bp)	762,267,613	768,808,243
Number of contigs	1,355	1,372
Contigs N50 (bp)	2,183,645	2,130,780
Number of Scaffolds	23	24
Scaffold N50 (bp)	32,431,321	33,548,962
Genome coverage (X)	251.1	314.6
Complete BUSCOs (%)	98.2	98.0

Materials and Methods

Sample collection, sequencing and genome quality

Male and female *O. fasciatus* captured from Qingdao, Shandong Province (Yellow Sea) were utilized for genome sequencing and assembly. High-quality genomic DNA was extracted from fresh muscle tissue and blood samples from pairs of male and female *O. fasciatus* using the Illumina platform (Illumina Inc., San Diego, CA, USA) and PacBio Sequel (Pacific Biosciences of California, Menlo Park, CA, USA). Genomic DNA from fresh muscle tissue and blood samples of male and female *O. fasciatus* pairs was extracted to construct short insert libraries (300~350 bp) for males and females, respectively, using standard protocols provided by the Illumina HiSeq X Ten platform (Illumina Inc., San Diego, CA, USA). Raw reads totaling approximately 90.7 Gb and 63.9 Gb were generated. After removing splice-containing sequences, low-quality, and redundant reads, approximately 80.8 Gb and 56.6 Gb of pure data were obtained from the genomes of males and females, respectively, corresponding to Q20 averages of 97.55% and 97.60%, respectively. Genomic DNA libraries (20 kb) for male and female fish were then constructed using the PacBio Sequel platform (Pacific Biosciences of

California, Menlo Park, CA, USA). Sequencing was performed on the PacBio Sequel platform using the Sequel Binding Kit 2.0, Sequel Sequencing Kit 2.1, and Sequel SMRT Cell 1M v2. Seven and five SMRT Cells were used for sequencing the male and female genomes, respectively, with SMRT LINK 5.0 used to filter the raw data from the zero-mode waveguide. We obtained 62.8Gb and 39.8Gb of long read length PacBio sequencing data for female (accession SRP160016) and male (accession SRP220007) genome assembly, respectively. The female and male *O. fasciatus* genome were de novo assembled using Canu v1.4 with Corrected-Error-Rate set to 0.040, followed by redundancy removal using Redundans v0.13c (minCoverage = 15), genome polishing with Arrow tool in SMRT Link 5.0, and final assembly refinement with Pilon using Illumina NGS short reads from the genome surveys. Based on the male and female draft genomes and Hi-C technology, we obtained high-quality genomes at the chromosome level of female (768.8Mb, accession SUB14064808) and male (762.2Mb, accession SUB14065793) *O. fasciatus*, and the contig N50 and scaffold N50 of the male and female fish genomes reached 2.13 Mb/2.18 Mb and 33.5 Mb/32.4 Mb, respectively. To assess the genomic completeness of assembled male and female *O. fasciatus*, we subjected the assembled sequences to BUSCO version 5 evaluation (BUSCO, actinopterygii_odb10), with Complete BUSCOs assessed values up to 98.2% and 98.0% for male and female genomes, respectively.

2. The Introduction is outdated. The state of the art in long-read sequencing as described in the Introduction is at least 5 years old; the mention of the human genome with a contig N50 of 8.3 Mb (line 118) should rather be replaced by the more recent telomere to telomere efforts; the single reference (26) to a random fish genome is similarly out of place. There are far more than 40 (line 101) fish species that have had their genome sequenced. And finally, the inclusion of Helicos (111) as a mature long-read platform is strange - the company disappeared over a decade ago, and although their platform was single-molecule, it certainly didn't produce long reads.

Reply:

Thanks for your professional suggestions, I apologized for the confusion due to the lack of clarity. Here, we would like to highlight that the methods used for sequencing

fish genomes before 2018 were mainly short-read-long sequencing methods represented by Illumina, making it difficult to obtain high-quality genome maps and precise high-throughput information on chromosomal structural variants. To avoid misunderstanding, we have modified the content here as follows. We revised "In 2002, the genome sequencing of *Takifugu rubripes* was completed, making it the first model fish to have its entire genome sequenced[18]. Since then, whole-genome sequencing of more than 40 fish species, such as *Oryzias latipes*, *Gasterosteus aculeatus* and *Salmo salar*, has been completed both nationally and internationally" as "In 2002, the genome sequencing of *Takifugu rubripes* was successfully accomplished, marking it as the pioneering model fish species to undergo complete genome sequencing[18]. Subsequently, the whole-genome sequencing of more than 200 fish species, including *Oryzias latipes*, *Gasterosteus aculeatus*, *Pelteobagrus fulvidraco*, *Mola mola*, *Nothobranchius furzeri*, and *Salmo salar*, has been carried out on a national and international scale. Over the span of approximately 16 years until 2018, the majority of fish genomes were sequenced utilizing second-generation sequencing technology, specifically Illumina[4, 18-21]. Despite the high throughput capabilities of second-generation sequencing, the relatively short sequencing read lengths posed challenges in obtaining precise genome maps and accurate information on chromosome structural variation. This limitation was especially pronounced in genomes with high complexity and repetitive regions.".

We also greatly appreciated your professional reminder that we have removed Helicos from the long-read length high-throughput sequencing content as follows:

We revised "At present, third-generation sequencing technology with high precision of long fragments represented by single-molecule real-time sequencing has matured, which includes Helicos' single-molecule sequencing technology (SMSTM), Oxford Nanopore's single-molecule nanopore sequencing technology, Pacific Biosciences' (PacBio) single-molecule real-time sequencing technology (SMRT), and so on." as "At present, third-generation sequencing technology with high precision of long fragments represented by single-molecule real-time sequencing has matured, which included Pacific Biosciences' (PacBio) single-molecule real-time sequencing technology (SMRT), Oxford Nanopore's single-molecule nanopore sequencing technology , and so on.".

Thanks to your professional advice, we have introduced the latest T2T assembly results of the human genome into our paper, and as you said, the quality of the assembly of the human genome has reached an almost perfect level with the enhancement of long read long sequencing technology. We have made the following changes to the text about the assembly of the human genome. We revised "For the Chinese human genome sequenced by PacBio technology, the assembly level reached a contig N50 of 8.3 Mb, which met the standard of the completed map" as "The initial assembly of the human telomere-to-telomere (T2T) CHM13 genome, utilizing PacBio HiFi and Oxford Nanopore ultralong-read sequencing, achieved a contig N50 of 154.26 Mb, resulting in a seamless assembly that fills a crucial gap in the human DNA genetic code map".

"In recent years, with the rapid development of genome sequencing technology and the significant decrease in the cost of sequencing, it has become possible to perform whole-genome sequencing of important cultured species. Rapid developments in next-generation sequencing (NGS) technologies have created significant opportunities for the discovery and use of species germplasm resources[4]. In 2002, the genome sequencing of *Takifugu rubripes* was successfully accomplished, marking it as the pioneering model fish species to undergo complete genome sequencing [18]. Subsequently, over the course of nearly 16 years, the whole-genome sequencing of more than 40 fish species, including *Oryzias latipes*, *Gasterosteus aculeatus*, *Pelteobagrus fulvidraco*, *Mola mola*, *Nothobranchius furzeri*, and *Salmo salar*, has been carried out on a national and international scale [19-30]. Over the span of approximately 16 years until 2018, the majority of fish genomes were sequenced utilizing second-generation sequencing technology, specifically Illumina [4, 18-21]. Despite the high throughput capabilities of second-generation sequencing, the relatively short sequencing read lengths posed challenges in obtaining precise genome maps and accurate information on chromosome structural variation. This limitation was especially pronounced in genomes with high complexity and repetitive regions.

At present, third-generation sequencing technology with high precision of long fragments represented by single-molecule real-time sequencing has matured, which included Pacific Biosciences' (PacBio) single-molecule real-time sequencing technology (SMRT), Oxford Nanopore's single-molecule nanopore sequencing technology, and so on. Among them, PacBio RS sequencing technology was the most

widely used and commercialized core third-generation sequencing technology and has been widely used in the genome assembly of several species, such as an Asian man, *Oropetium thomaeum* and *Lates calcarifer* [31-33]. The initial assembly of the human telomere-to-telomere (T2T) CHM13 genome, utilizing PacBio HiFi and Oxford Nanopore ultralong-read sequencing, achieved a contig N50 of 154.26 Mb, resulting in a seamless assembly that fills a crucial gap in the human DNA genetic code map [31, 34-36]. In addition, Hi-C technology based on next-generation sequencing technology has gradually become a favourable tool for chromosome assembly [37]. With the rapid development of sequencing technology and the improvement of assembly technology, the genome integrity and accuracy have been greatly improved, especially in marine fishes, and the assembly now obtained Contig N50 up to the level of 28 Mb for *Sciaenops ocellatus* [38]. Thus, the development of next-generation sequencing technologies has made it possible to exploit high-throughput sex-specific markers in high-precision assembled genome~~linear genomic~~ data."

3. The Discussion is severely outdated. All references to structural variation are from the dawn of the genomics era, and even older: ref 68, 69: 1991, ref 70-72: 2001, ref 73: 2006, ref 74: 2003. Structural variation in genomes is a major research theme these days, and the Discussion should acknowledge that by placing the results in the context of recent knowledge.

Reply:

We are very grateful for your expert comments and perceptive insights, which are extremely rewarding to learn from. Based on your comments, we have reviewed and supplemented the proportions of insertion deletion variants in the genomes of the latest human, mouse, and Arabidopsis species. Of course, we have retained the previous data for the classical species Drosophila and nematode in consideration of the continuity of the data. At the same time, based on the latest results of long sequencing technology, we further discuss the relationship between genomic structural variation in large segments and phenotypic traits, species differentiation and adaptive capacity of organisms as follows.

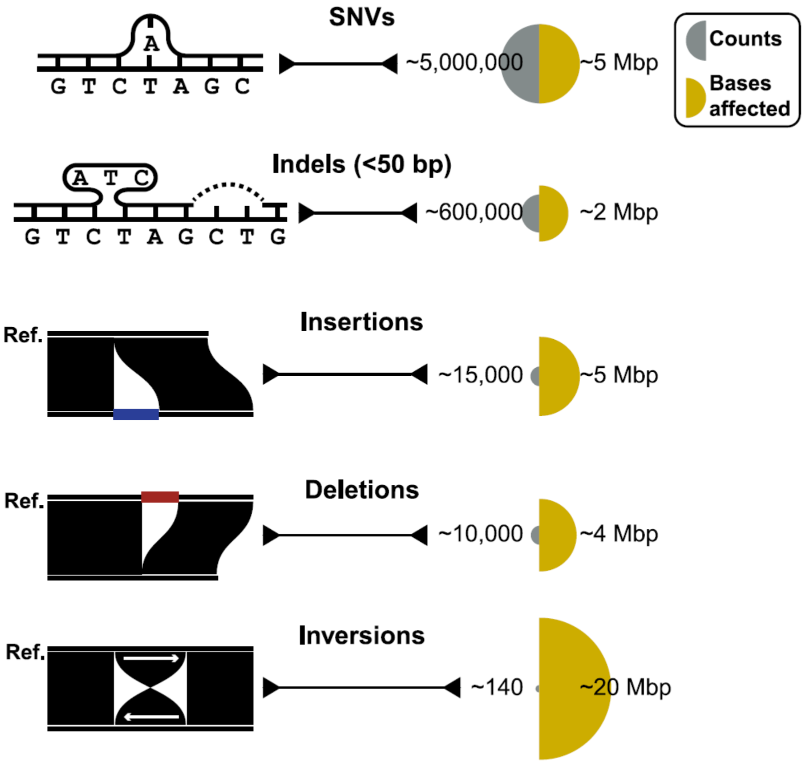

Figure 4. Types of human genetic variants by abundance

"Variation in genome structure was intricately linked to human diseases, evolution, gene regulation, and species phenotypes in the biological realm, making it a prominent area of study in genomics research [79]. Previous studies have shown that DNA sequences with insertions and deletions undergo increased selective pressure [80-84]. The presence of deletions and insertions in DNA sequences, regardless of their location in noncoding or coding regions, inevitably influences the functionality of the original sequence to varying extents [79]. Furthermore, evidence suggested that the length and placement of insertions were not random but rather heavily influenced by the adjacent DNA sequence [80-82, 85, 86]. Prior research has indicated that insertion deletion variants accounted for 16%, 24%, 13%, 25%, and 15% of genomic genetic polymorphisms in various organisms, including *Drosophila melanogaster*, *Caenorhabditis elegans*, mouse, human, and *Arabidopsis thaliana* [34-36, 87-94]. Recent advancements in long length sequencing technology have allowed for a more precise examination of human genetic variation, revealing that insertions and deletions collectively contribute to a high portion (13.8%, 11.1%) of genetic polymorphisms in the human genome [90-94]. The focus of genomic structural variation studies has predominantly been on single nucleotide polymorphisms (SNPs) and

insertions/deletions (InDels) of less than 50 base pairs due to the limitations of high-throughput sequencing technologies for short read lengths. However, larger genomic structural variants, which encompassed genetic variations not fully captured by SNPs and InDels, were closely linked to biological phenotypes and might significantly influence phenotypic traits, disease susceptibility, and adaptive capacity [79]. The utilization of long-read length sequencing technologies such as PacBio and ONT has enabled the generation of extended DNA sequences, thereby enhancing the assembly of diploid genomes of superior quality and the identification of structural variants (SVs) across substantial genomic regions [95]. Prvious research has documented seven deletions (>100bp) within approximately 700bp large segments of the *ZmBAM1d* gene, demonstrating a positive association with maize kernel weight. These deletions were consistently inherited in high-yielding maize lines [96]. In mammalian studies, a deletion of approximately 40Mb on chimpanzee chromosome 2, as compared to humans and gorillas within the great ape family, has been identified, leading to stable genetic distinctions among species [97]. Similar results have been documented in *Astyanax mexicanus*, *Gasterosteus aculeatus*, racehorses, and dogs [98-101]. The emergence of long read-length sequencing technology has propelled large fragment genomic structural variation to the forefront of genetic variation research, particularly in investigations of species' adaptive evolution [95, 96, 102, 103]. The findings of this study indicated that insertions and deletions identified using the female and male *O. fasciatus* genomes as references constituted 26.96% and 26.97% of the genetic polymorphisms, respectively. Our research has contributed new insights into the prevalence of large-segment insertion-deletion events for *O. fasciatus*, indicating that these events predominantly occurred in the intergenic region in 58.09%~66.33% of instances, with the intronic region following closely at 31.65%~40.35% of cases (Figure 2, Figure S1 Table 2). We also observed a higher proportion of deletion sites compared to insertion sites. A similar study conducted on the β-fibrinogen intron of birds reported similar findings [104]. Furthermore, this study presented novel findings indicating that over 77% of the identified large deletion sequence tag sites in the genomes of both female and male *O. fasciatus* were attributed to transposons (Table 4). It was evident that transposons were prevalent in the animal genome and significantly contribute to its evolutionary processes. Previous research has demonstrated that a significant portion (69.5%) of genomic structural variation in large segments of tomato was situated within the transposon region, which was closely linked to desirable traits

in tomato [105]. Comparable studies have also been conducted on gene regulation in human brain diseases and genomic structural variation in evolutionary distinct phenotypes of great apes [103, 106]. Transposons have the potential to drive genome and chromosome evolution through various mechanisms such as insertion-induced gene variation, gene retrotransposition, chromosome recombination, gene duplication, and serving as sites for ectopic recombination. These findings underscored the importance of transposons in facilitating adaptive evolution for *O. fasciatus*. The presence of large fragments of insertion deletion sequence marker sites was frequently influenced by natural selection pressures, with their formation being intricately linked to adjacent DNA sequences and commonly correlated with characteristic phenotypic traits and aberrant gene regulation [83, 84, 102, 103, 105, 106]. Specifically, the manifestation of albino symptoms in dogs resulting from a 965-kb deletion on chromosome 18 exhibited stable inheritance patterns, while a 4.8 Mb deletion variant on the chromosome of male domestic chickens similarly displayed heritability across subsequent generations [100, 107]. Consistent inheritance of variants within populations has also been documented in academic research on maize (*ZmBAM1d*), *Astyanax mexicanus*, *Ailuropoda melanoleuca* (*Bace2*), great apes (*acan*), and human diseases such as hypertension (*ace*) [96, 98, 103, 108]. The findings from the population genetic sex marker amplification analysis conducted in this study indicated that the insertion-deletion markers exhibited stability in both male and female populations of *O. fasciatus*. Additionally, analysis of gonadal histology and sex-validated marker data from various geographic populations demonstrated consistent inheritance of large segments of the insertion deletion markers in individuals of both sexes across different geographic regions. This observation provided additional evidence to support the theory that large-segment insertions and deletions are not a random occurrence but rather influenced by natural selection pressures [80, 82-84]. Based on the present findings, the stability of large segment insertion and deletion in sequence tag sites was evident. However, this should not be taken to imply that all sequence tag sites exhibit this characteristic. A comprehensive evaluation of the evolutionary rate of sequence tags and the expansion of variant marker detection are necessary for ongoing research in this area.”

4. Results and Figure 2/Table 1: needs to be presented more clearly. The first reference to fig 2 (line 218) does instead refer to the unfiltered data. Insertions (panels a, c) are summarized by number of

events, deletions (b, d) by length in nucleotides. The data in fig 2d is not described in the text.

Reply:

We thanks for your detailed and professional comments. Incorporating the revisions you made, we find that it will make our results clearer and enable readers to understand us more clearly without misunderstanding, and therefore, based on your comments, we have made the following changes.

1) Firstly, we obtained raw variation data based on the male and female genomes as a reference using bar charts for presentation, and placed the results of insertion and deletion type data in a row for easy comparison. At the same time, we made an explanation in the figure notes to clarify that the insertion variant types were summarised according to the number of events, and the deletion variant types were summarised according to the nucleotide length. At the same time, we used dynamic pie charts to show the number and proportion of insertions and deletions in intergenic regions, intronic regions, cds regions, and so on, and put them in the Supplementary file to assist the explanation.

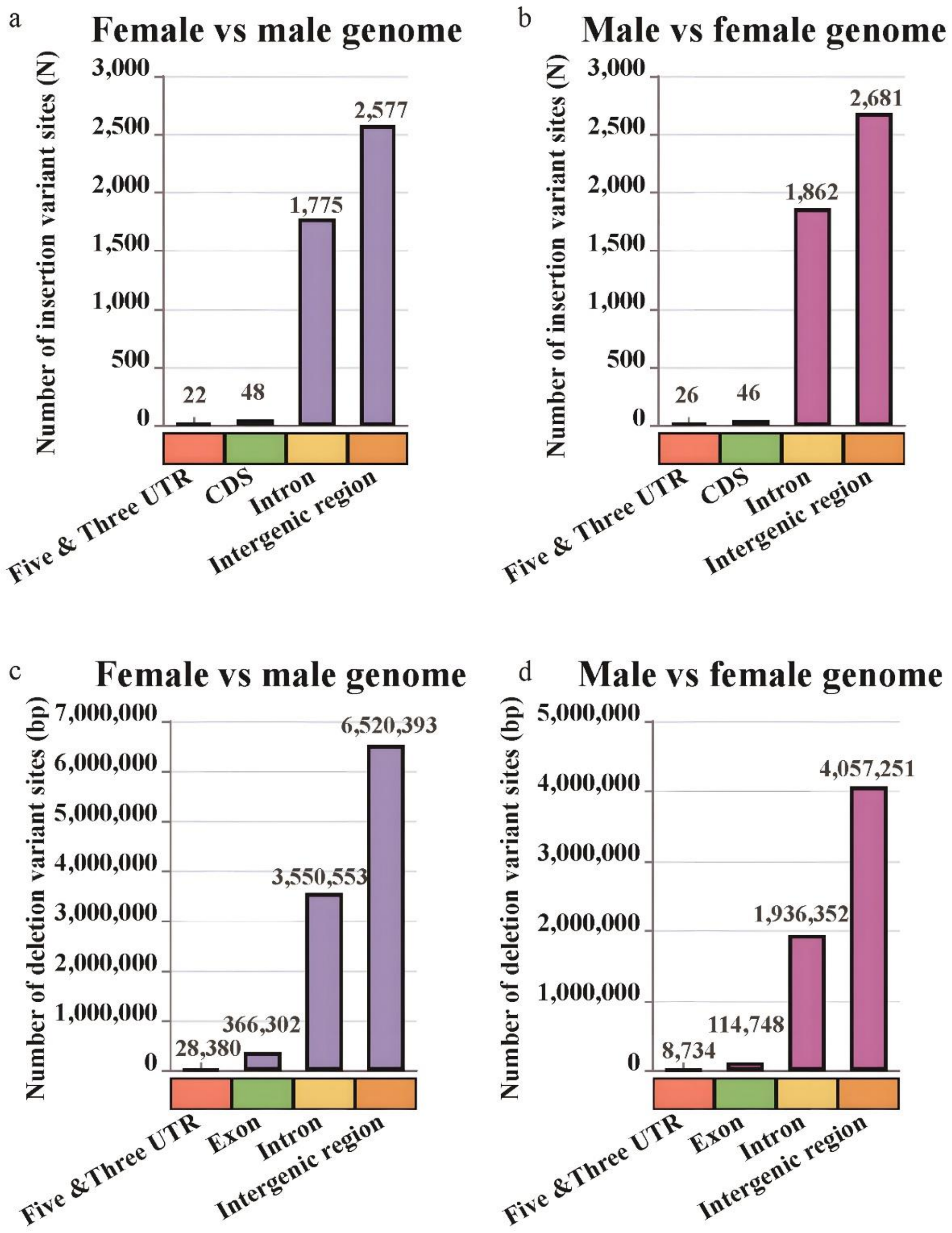

Figure 2: Distribution statistics of insertion and deletion fragment (＞100bp) positions in genomic functional regions of *O. fasciatus*.

(a) Distribution statistics of insertion variant loci using the female fish genome as a reference. (b) Distribution statistics of insertion variant loci utilizing the male fish genome as a point of reference. (c) Distribution statistics of deletion variant length using the female fish genome as a reference. (d) Distribution statistics of deletion variant loci using the male fish genome as a reference.

Note: The colors red, green, yellow, and orange represented the UTR, CDS, intron, and intergenic regions. The frequency of insertions (a, b) was quantified based on the number of events, while deletions (c, d) were characterized by their length in nucleotides.

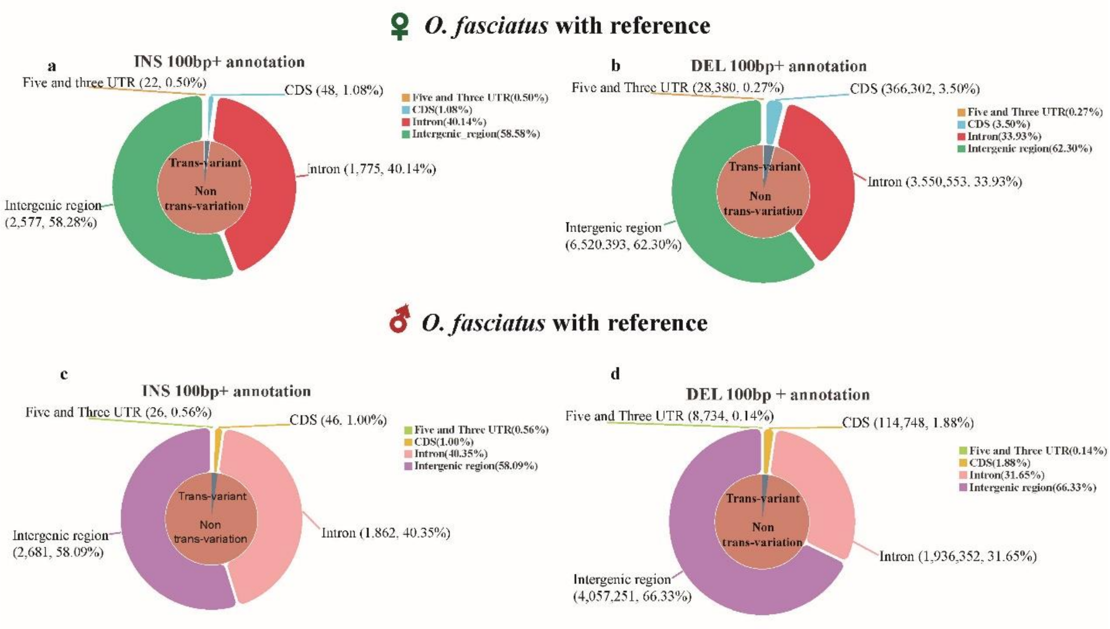

Figure S1: Frequency of insertion and deletions (＞100bp) in functional regions of the *O. fasciatus* genome

(a) Number and frequency of insertion events using the female *O. fasciatus* genome as reference. (b) Distribution statistics of deletion variant length using the female *O. fasciatus* genome as a reference. (c) Number and frequency of insertion variant loci utilizing the male *O. fasciatus* genome as a point of reference. (d) Distribution statistics of deletion variant loci using the male *O. fasciatus* genome as a reference.

Note: Non trans-variation represented the intron, and intergenic regions. Trans-variation represented the UTR, CDS regions. The frequency of insertions (a, c) was quantified based on the number of events, while deletions (b, d) were characterized by their length in nucleotides.

2) Secondly, we have added labelling to the insertion deletion variant locus statistics table to make the distribution of the data clearer to the reader.

Table 2. Statistics of the positional distribution of insertion/deletion variants in the male and female genomes of *O. fasciatus*.

Type of gene function region	Female genome as a reference		Male genome as a reference	
	Insertion variant loci no. (>100 bp)	Deletion variant length (>100 bp)	Insertion variant loci no. (>100 bp)	Deletion variant length (>100 bp)
5′ UTR	5	7,859	6	2,482
3′ UTR	17	20,521	20	6,252
CDS	48	366,302	46	114,748
Intron	1,775	3,550,553	1,862	1,936,352
Intergenic region	2,577	6,520,393	2,681	4,057,251
Total	4,422	10,465,628	4,615	6,117,085

Note: Insertions (>100 bp) were summarized by number of events. Deletions (>100 bp) were summarized by length in nucleotides.

3) Thirdly, we explicitly indicated unfiltered and filtered data and supplemented the unfiltered male-female difference dataset as a supplementary file as follows.

① We revised “Using the female genome as a reference, we obtained 1,919,620 male and female differential variant sites, including 191,915 insertion sites, 325,620 deletion sites and 1,402,085 nucleotide substitutions (Figure 2).” as “**Using the female genome as a reference, we obtained 1,919,620 male and female differential variant sites with unfiltered data, including 191,915 insertion sites, 325,620 deletion sites and 1,402,085 nucleotide substitutions (Supplementary Female_reference_all_variation_Set.xls).**”

② We revised “Using the male *O. fasciatus* genome as a reference, 1,926,295 female–male differential variant sites were obtained, including 326,823 insertion sites, 192,672 deletion sites and 1,406,800 nucleotide substitution sites (Figure 2).” as “**Using the male *O. fasciatus* genome as a reference, 1,926,295 female–male differential variant sites with unfiltered data were obtained, including 326,823 insertion sites, 192,672 deletion sites and 1,406,800 nucleotide substitution sites (Supplementary Female_reference_all_variation_Set.xls)**”.

5. Figure 3a/b: There is a huge inversion between female chr 8 and male chr 9. Did you consider using this type of structural variation to design PCR tests?

Reply:

We are very grateful for your expert comments and careful advice. As in our previous study, we found that the male and female chromosomes of *O. fasciatus* differed and that the males underwent a chromosome fusion event. Moreover, the male fusion chromosome (MChr9) showed significant structural variation compared to its homologous chromosomes (FChr8 and FChr10 in females). Therefore, we focused on the differences between male and female homologous chromosomes that underwent chromosomal structural variation, especially since there was a large inversion zone. This inversion region allowed for the presence of rich information on male and female genetic variation in this region. As you know, the inversion region is rotated by 180 compared to the original sequence and reverse complementarity occurs at the same time, so when we set up the primers, the upstream and downstream primers are normal for normal sequences, while for inversion region sequences, the upstream and downstream primers are interchanged, but the target region of amplification is unchanged. We have

also listed a case. From the results, the inversion does not affect the amplification of the target region, and we also put this marker with male and female differences into the male and female genomes for e-PCR amplification, and the results show that the identification of male and female genetic sexes can be realized in the male and female genomes.

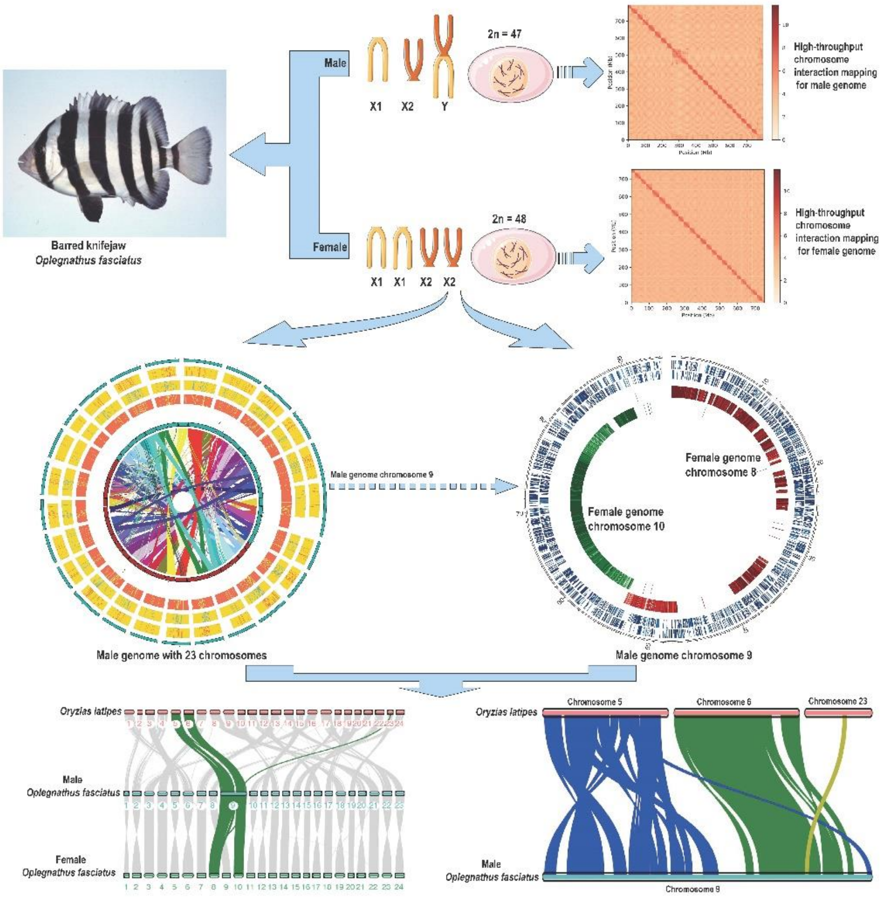

Male and female chromosomal differences in *O. fasciatus* and a chromosomal fusion event in males

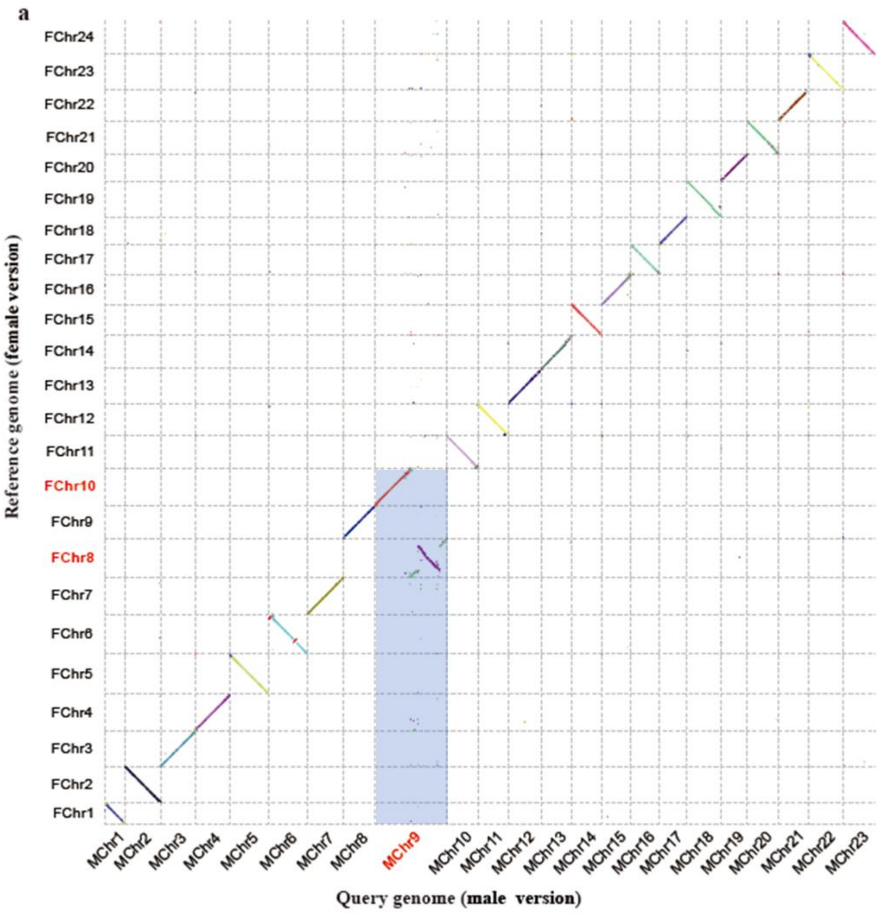

Male fish fusion chromosome (MChr9) undergoes significant structural variation compared to its homologous chromosomes (female FChr8 and FChr10)

Primer:

F: 5~ AAGTGAAGAAAGTTCATGATGGCACTT~3

R: 5~ TCTGCCGATGTGCTTGGTCTACGATCTTTCA~3

>Eaxmple1**- normal sequences (739bp)**

F: 5~ AAGTGAAGAAAGTTCATGATGGCACTT~3

AAGTGAAGAAAGTTCATGATGGCACTTAGAACAGTCAGAGTCTTCTGTTTTTCTGGA

AAGGAAGAACAACACCGCTATCCAGTTATCAGATGTGCAAATAAGTGACAGTGTTTCC

CACACATTGGTTATTGGTTTGTGGCGGCCCGGCACAATATTAACATTGACCGCCACATAT

TGATTTTCTATTTTTTTAAACTCCTTGCCCCACTCTTGCGCTCTCTCTCTCTCTCTCTC

ACAAACAGCTTGACAACAGACCTGTCTGTGAAGAGCCCCTCCTCTCCGTACACACTCT

AAATCAAAACTATGGCGTTTAATTAGTCTCAAAACTGTGCACATATATAAAACAGGTGC

GTGCACGTGATACGAGCACACTGAACAGAATCCGCGAAACCCGTGCATGTACAAGCG

CGGTCCCGCAAACACGGGGCTCTGACGACCATGAGCTCGACCCTCCCTACGTGCTCGC

AACTGCTCAACACTCGCTCGCTCATGAAGTTGACTTTTGAGACTTTGGAGGTGGGGAT

GGAATAGGCATTGGCTGATGTGGCCCCTCCTGTGATTCCTTTTATTGGTCACTTTCAAGC

TCAGCTACCACCACAAATAGAATCTAATTCTGGGGGAAACACTGAGTGATATACTTTTA

GAAAAACTTTGGACATATAAAACTGAAAAACATACACTTACTTGGAGCTAGAAGGTCA

TTCAG**TGAAAGATCGTAGACCAAGCACATCGGCAGA**

3~**ACTTTCTAGCATCTGGTTCGTGTAGCCGTCT~5** **R**

>Eaxmple2- **inversion sequence (739bp)**

R: 5~ TCTGCCGATGTGCTTGGTCTACGATCTTTCA~3

TCTGCCGATGTGCTTGGTCTACGATCTTTCACTGAATGACCTTCTAGCTCCAAGTAA

GTGTATGTTTTTCAGTTTTATATGTCCAAAGTTTTTCTAAAAGTATATCACTCAGTGTTTC

CCCCAGAATTAGATTCTATTTGTGGTGGTAGCTGAGCTTGAAAGTGACCAATAAAAGG

AATCACAGGAGGGGCCACATCAGCCAATGCCTATTCCATCCCCACCTCCAAAGTCTCA

AAAGTCAACTTCATGAGCGAGCGAGTGTTGAGCAGTTGCGAGCACGTAGGGAGGGTC

GAGCTCATGGTCGTCAGAGCCCCGTGTTTGCGGGACCGCGCTTGTACATGCACGGGTT

TCGCGGATTCTGTTCAGTGTGCTCGTATCACGTGCACGCACCTGTTTTATATATGTGCAC

AGTTTTGAGACTAATTAAACGCCATAGTTTTGATTTAGAGTGTGTACGGAGAGGAGGG

GCTCTTCACAGACAGGTCTGTTGTCAAGCTGTTTGTGAGAGAGAGAGAGAGAGAGAG

CGCAAGAGTGGGGCAAGGAGTTTAAAAAAATAGAAAATCAATATGTGGCGGTCAATGT

TAATATTGTGCCGGGCCGCCACAAACCAATAACCAATGTGTGGGAAACACTGTCACTT

ATTTGCACATCTGATAACTGGATAGCGGTGTTGTTCTTCCTTTCCAGAAAAACAGAAGA

CTCTGACTGTTCT**AAGTGCCATCATGAACTTTCTTCACTT**

3~**TTCACGGTAGTACTTGAAAGAAGTGAA**~5 **F**

From the above results, the inversion does not affect the amplification of the target region, and we put this marker with male and female differences into the male and female genomes for e-PCR amplification, and the results show that the identification of male and female genetic sexes can be realized in the male and female genomes.

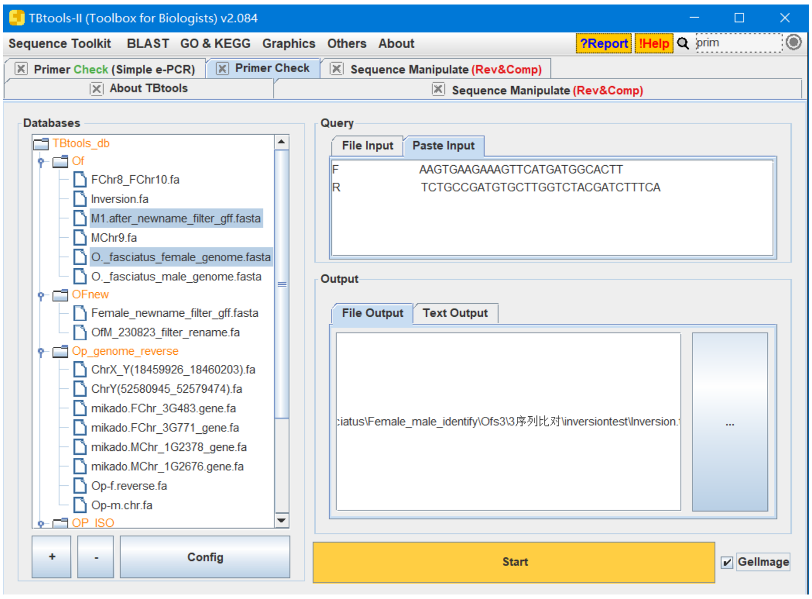

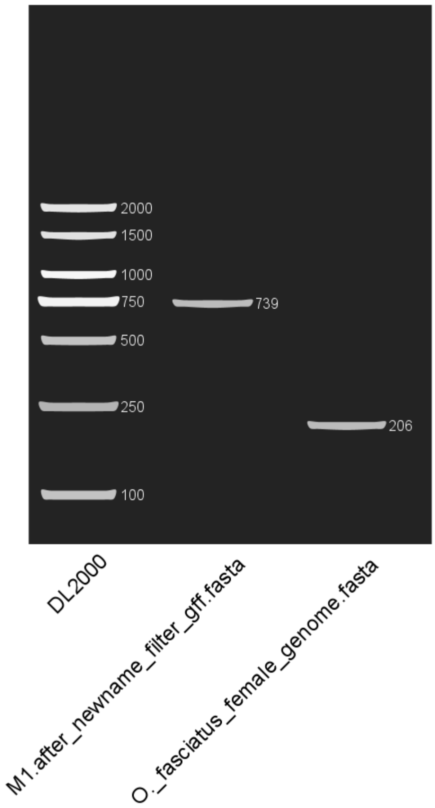

6. Figure 3c/d: According to the legend, ring 2 (from outside) are insertions, and ring 3 deletions. There should be some balance between these two between the two contrasts. Now is seems as in both contrasts there are far fewer deletions than insertions, which is implausible. Also, the corners of the white background of the fish image block out some data.

Reply:

We thanks for your detailed and professional comments. As you pointed out to us earlier in the proposal, insertions are summarized by number of events and deletions are summarized by nucleotide length, as we have already done with the figure and table notes in the previous representations. Here we explain it in more depth, in the circle diagram we use is with a 10k sliding window, our insertions represent the number of insertion positions, the more insertions the higher the point position, the fewer insertions the lower the point position. Whereas deletions are counted by number of bases in a 10k sliding window and represent a length value. Inspired by your professional opinion, we have counted and added the number of insertion deletion sites on each chromosome to the table in order to prevent similar misinterpretations, and at the same time we have updated the double-valued bar charts. From our results, there is some convergence in the number of male and female insertion deletions. Also, we have taken care of the problem of the edges of the white background of the fish image in figure3 obscuring the data.

FChr1	260000	270000	2
FChr1	270000	280000	1
FChr1	280000	290000	4
FChr1	290000	300000	6
FChr1	300000	310000	2
FChr1	310000	320000	4
FChr1	320000	330000	5
FChr1	330000	340000	1
FChr1	340000	350000	1
FChr1	350000	360000	3

Positional statistics of 10k sliding window insertion counts with female genome as reference

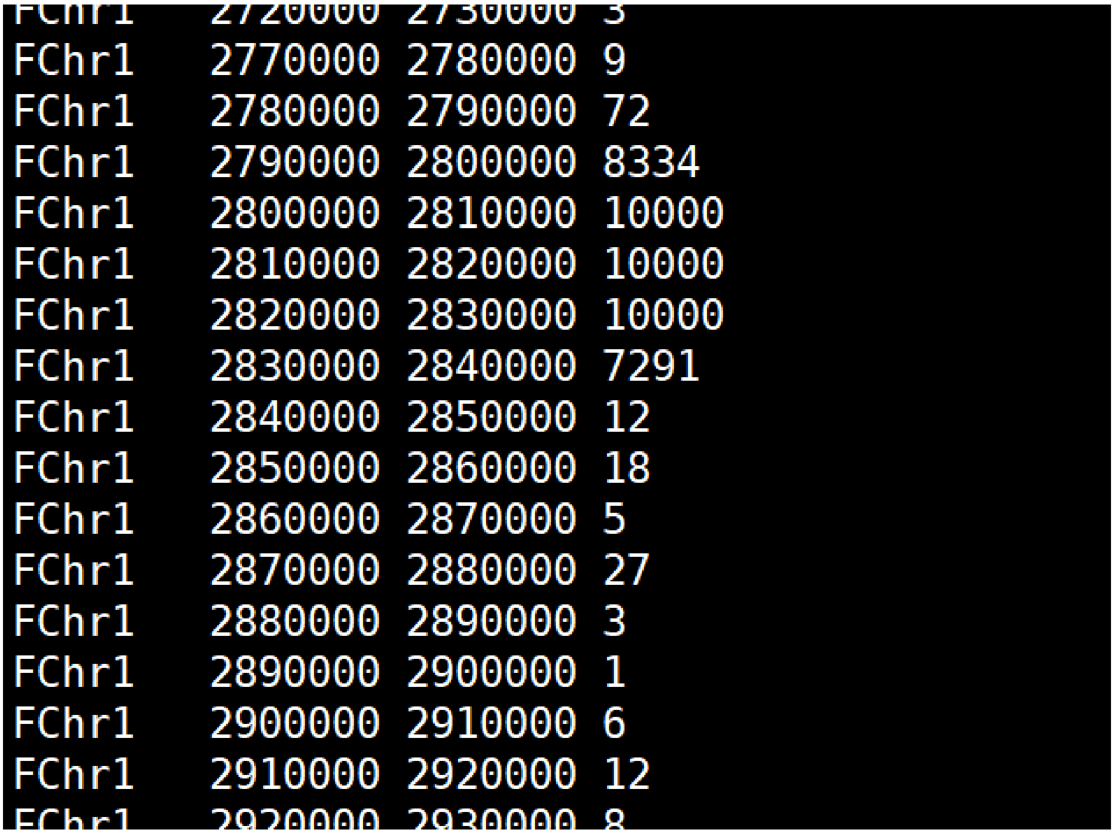

```
[illegible]
FChr1   2770000 2780000 9
FChr1   2780000 2790000 72
FChr1   2790000 2800000 8334
FChr1   2800000 2810000 10000
FChr1   2810000 2820000 10000
FChr1   2820000 2830000 10000
FChr1   2830000 2840000 7291
FChr1   2840000 2850000 12
FChr1   2850000 2860000 18
FChr1   2860000 2870000 5
FChr1   2870000 2880000 27
FChr1   2880000 2890000 3
FChr1   2890000 2900000 1
FChr1   2900000 2910000 6
FChr1   2910000 2920000 12
FChr1   2920000 2930000 8
```

Statistics on the length of deletion bases in the 10k sliding window using female genome as a reference

Table 3. Statistical cross-validation of multiple scanning of genome-wide large insertion deletion sequence marker sites.

Reference genome	Female vs. Male			Male vs. Female		
Mutation type	Insertion		Deletion	Insertion		Deletion
Variant length (>100 bp)	4,422		4,675	4,615		4,385
Chromosomes	FChr1	131	123	MChr1	140	143
	FChr2	194	211	MChr2	208	194
	FChr3	173	199	MChr3	179	161
	FChr4	181	207	MChr4	214	186
	FChr5	241	220	MChr5	212	237
	FChr6	254	292	MChr6	277	246
	FChr7	212	234	MChr7	231	211
	FChr8/10	333	341	MChr8	143	165
	FChr9	165	146	MChr9	345	333
	FChr11	191	217	MChr10	217	179
	FChr12	157	160	MChr11	159	153
	FChr13	205	187	MChr12	185	203
	FChr14	158	193	MChr13	196	162
	FChr15	182	185	MChr14	186	188
	FChr16	169	202	MChr15	190	160

	FChr17	204	220	MChr16	228	196
	FChr18	168	159	MChr17	159	167
	FChr19	175	183	MChr18	168	171
	FChr20	168	184	MChr19	174	166
	FChr21	185	203	MChr20	205	183
	FChr22	161	183	MChr21	184	167
	FChr23	200	219	MChr22	225	211
	FChr24	215	207	MChr23	190	203
SyRI-based scanning of male and female mutant sites (≥100bp)			2,116			2,045
Intersection of the two methods			1,791 (84.64%)			1,749 (85.53%)

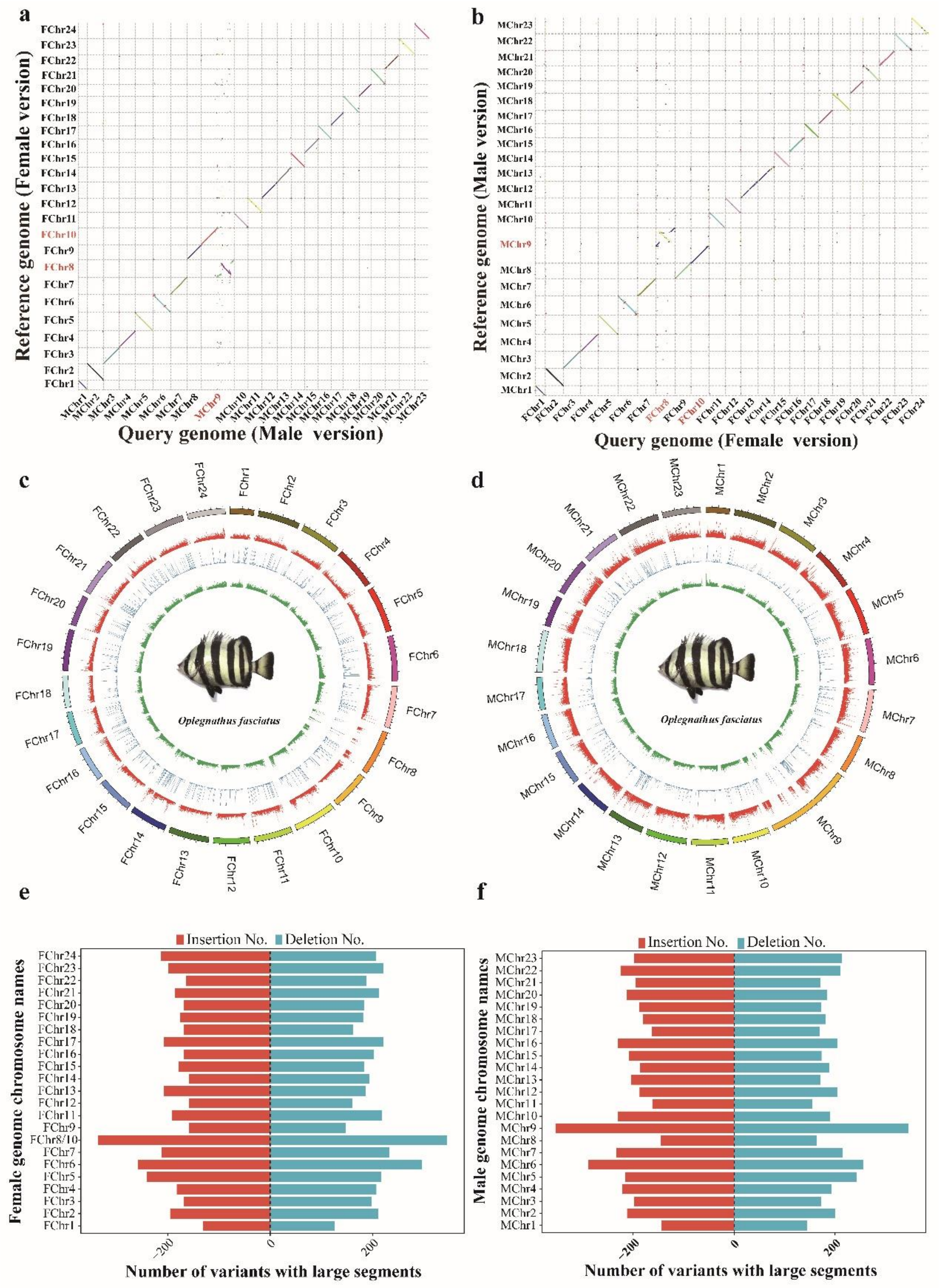

Figure 3: The covariance of genomic sequence differences between the male and female genomes of *O. fasciatus* and distribution statistics of variant sites.

7. Similar issue: In table 2, insertions and deletions are balanced. However, after primer design there are far fewer deletions. How can this be explained?

Reply:

We thanks for your detailed and professional comments. High-throughput primer

design and high-throughput amplification of e-PCR for insertion deletion sites were designed to screen for suitable male and female genetic sex loci, and to assist primer design and amplification detection by agarose gel electrophoresis. In this study, we prioritized primer pairs and loci that could amplify a single target band in both male and female genomes based on e-PCR. Of course, the simulation principle of e-PCR is firstly to design primers at 200bp upstream and downstream of the mutation site, and at the same time to ensure that the primers in the selected region should have similar Tm values, as well as limiting factors such as GC content. We supplemented the process of large-segment differential loci after primer design, e-PCR amplification, and simulated amplification products and size screening.

Previous research has indicated that insertion deletions are not randomly distributed, but rather are linked to specific sequence characteristics at their boundaries. Deletion events occur when the replicon skips certain nucleotides during replication, with sequences possessing high GC content showing a higher incidence of deletions. This may be attributed to the formation of unique structures in high GC regions, increasing the likelihood of sliding strand mismatch events. Similar patterns were observed in deletion sequences within human genes. Insertion variants arise from errors in DNA replication, where a segment of DNA is mistakenly duplicated and inserted into the genome. This process necessitates the ability to integrate a previously copied DNA fragment. The findings of this study indicated that the quantity of primer pairs generated through deletion e-PCR amplification, as well as the number of single amplification products, were lower in comparison to insertion events, irrespective of whether the female or male genome served as a reference. This discrepancy could be attributed to the constraints imposed by e-PCR on the GC content of flanking primers, whereas deletion variants frequently exhibited high GC content at both ends of the locus. Consequently, it was posited that the unique structural characteristics of deletion loci impeded the development of appropriate primers, leading to a reduced number of amplification products. The inquiry into the mechanisms driving the formation of insertion and deletion variants is of particular interest in the scientific community, particularly in light of recent findings linking deletions to the GC base content of

sequences flanking the deletion event. These associations will be further explored in subsequent studies.

Table 5. Statistics of large-segment differential loci after primer design, e-PCR amplification, and simulated amplification products and size screening.

Reference genome	Mutation type	Variant length (>100 bp)	High-throughput design of primers for variant sites	e-PCR of primers with single band	Single band and suitable amplification size (100~2000 bp)
Female vs. Male	Insertion	4,422	4,016	5,735	2,786
	Deletion	4,675	1,314	957	853
Male vs. Female	Insertion	4,615	4,131	3,236	2,876
	Deletion	4,385	1,199	882	796

8. Figure 3e/f: The asymmetry between the chromosomes is quite interesting, i.e. that some are prone to deletions and others to insertion at a whole-chromosome scale. However, please check these data; the figure as it is cannot be an accurate representation, as it should be symmetrical between the two panels. Meaning that an insertion in the male genome should be a deletion in the female, vv. This is not the case for chr 15F/14M and 19F/18M.

Reply:

We thanks for your detailed and professional comments. I am very sorry for the misunderstanding because of our visualization, in fact, as you mentioned earlier, the insertion and deletion sites are basically balanced, and when we made the Fig3e and f presentations, we took into account the number of insertion and deletion sites as positive values, which resulted in the bi-valued histograms being lopsided and obscuring the data. To solve this problem, we record the number of insertions as negative values and the number of deletion sites as positive values, so that when we perform the display, it will be a two-way display. To allow the reader to quantify insertion and deletion variation more clearly, we also added the number of insertions and deletions on each chromosome for illustrative purposes.

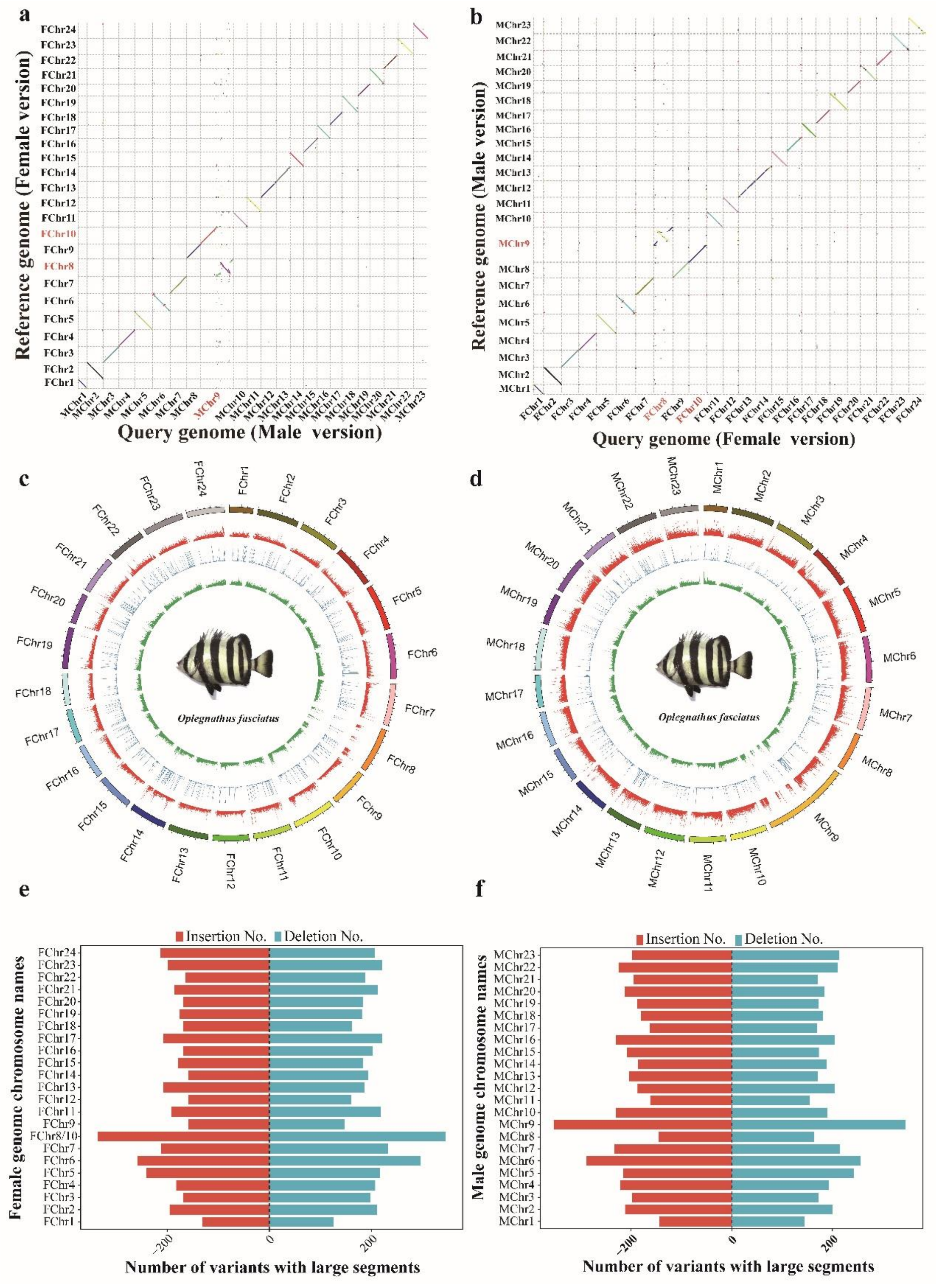

Figure 3: The covariance of genomic sequence differences between the male and female genomes of *O. fasciatus* and distribution statistics of variant sites.

Table 3. Statistical cross-validation of multiple scanning of genome-wide large insertion deletion sequence marker sites.

Reference genome	Female vs. Male		Male vs. Female	
Mutation type	Insertion	Deletion	Insertion	Deletion

Variant length (>100 bp)	4,422		4,675	4,615		4,385
Chromosomes	FChr1	131	123	MChr1	140	143
	FChr2	194	211	MChr2	208	194
	FChr3	173	199	MChr3	179	161
	FChr4	181	207	MChr4	214	186
	FChr5	241	220	MChr5	212	237
	FChr6	254	292	MChr6	277	246
	FChr7	212	234	MChr7	231	211
	FChr8/10	333	341	MChr8	143	165
	FChr9	165	146	MChr9	345	333
	FChr11	191	217	MChr10	217	179
	FChr12	157	160	MChr11	159	153
	FChr13	205	187	MChr12	185	203
	FChr14	158	193	MChr13	196	162
	FChr15	182	185	MChr14	186	188
	FChr16	169	202	MChr15	190	160
	FChr17	204	220	MChr16	228	196
	FChr18	168	159	MChr17	159	167
	FChr19	175	183	MChr18	168	171
	FChr20	168	184	MChr19	174	166
	FChr21	185	203	MChr20	205	183
	FChr22	161	183	MChr21	184	167
	FChr23	200	219	MChr22	225	211
	FChr24	215	207	MChr23	190	203
SyRI-based scanning of male and female mutant sites (≥100bp)			2,116			2,045
Intersection of the two methods			1,791 (84.64%)			1,749 (85.53%)

9. Primer design, line 262 and further: what were the criteria? What were the PCR settings? Also, 264: 'designed high-throughput primers to construct high-throughput primer datasets', please rephrase.

Reply:

Table 5. Statistics of large-segment differential loci after primer design, e-PCR amplification, and simulated amplification products and size screening.

Reference genome	Mutation type	Variant length (>100 bp)	High-throughput design of primers for variant sites	e-PCR of primers with single band	Single band and suitable amplification size (100~2000 bp)

Female vs. Male	Insertion	4,422	4,016	5,735	2,786
	Deletion	4,675	1,314	957	853
Male vs. Female	Insertion	4,615	4,131	3,236	2,876
	Deletion	4,385	1,199	882	796

We thanks for your detailed and professional comments. As you have seen above the table inside the high-throughput primer acquisition process, first we used the insertion and deletion site as the target regions for high-throughput primer design. The principle of primer design was to extend 200bp at each end of the target region to find a suitable primer design region, and at the same time to meet the conditions that the difference in Tm value should not be more than 15 degrees, the upstream and downstream primers should not be complementary, and so on. As you have seen from the table, not all target sequences can be designed, especially the two ends of the missing target region, the number of high throughput primers was much smaller than the number of primers inserted into the target region, which may be due to the fact that the two ends of the missing target region are often accompanied by the presence of high levels of GC content. Secondly, we amplified the whole genome of male and female based on the primer pairs of the target region obtained above, and this process will generate lots of non-specific amplifications, we selected only the primer pairs whose amplification product was a single amplification band through the synergistic screening of the upstream and downstream primers, and then formed the data catalog of the single-primer amplification products.

We also revised "We used the identified large insertion/deletion variants as target regions for male and female sex identification and designed high-throughput primers to construct high-throughput primer datasets for the target markers." as "The identified large insertion/deletion variants were utilized as target regions for sex identification in both male and female genomes. High-throughput primers were designed to generate primer catalogs for the specified target markers.".

10. Figure 4: is different in the inline version (p14) from the attached (p36). Also identical to figure S1.

Reply:

We thanks for your detailed comments. As you say the inline version of Fig. 4 (p. 14) is different from the accompanying drawing (p. 36) but the same as Fig. S1. Because the results in Figure 4 are from Figure S1, there is limited space in the text to exhibit all of what is in Figure S1. Based on your comments and those of the reviewers above, we have integrated the results of the validation images, and integrated the results of Fig. 4 with Fig. S1 as supplementary Figure S2.

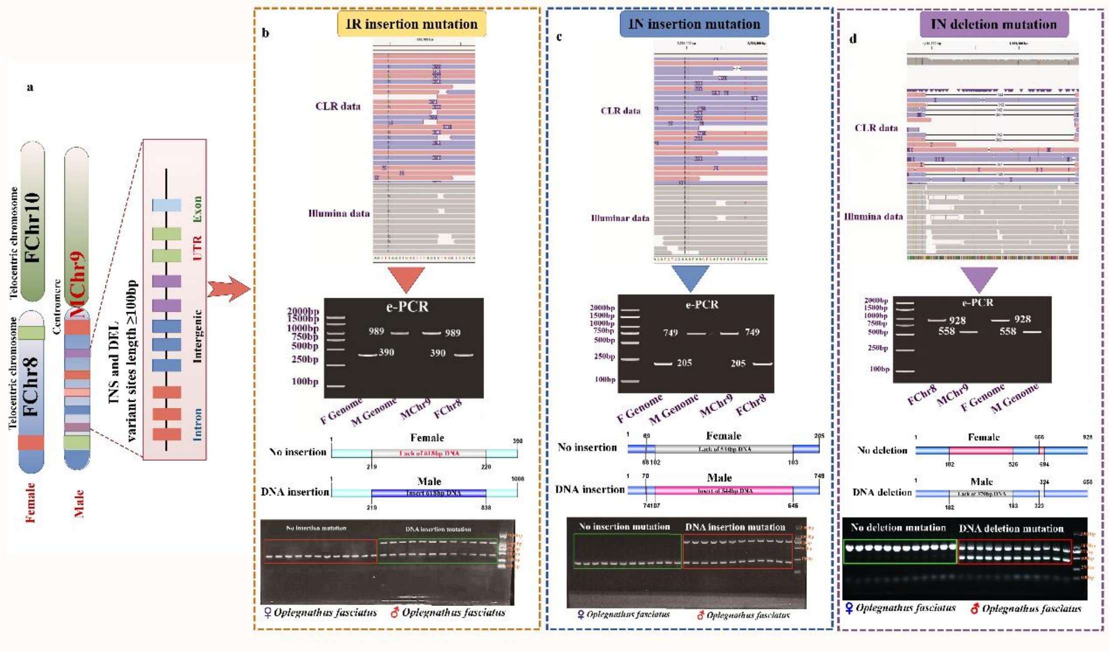

Validation of long read variant loci in males using the female genome as a reference

11. Figures 4-8 consist mostly of screenshots that are not clearly explained, and not intuitively very informative. What am I looking at?

Reply:

Thanks to your professional advice, We have organized all the images as requested the results are explained below

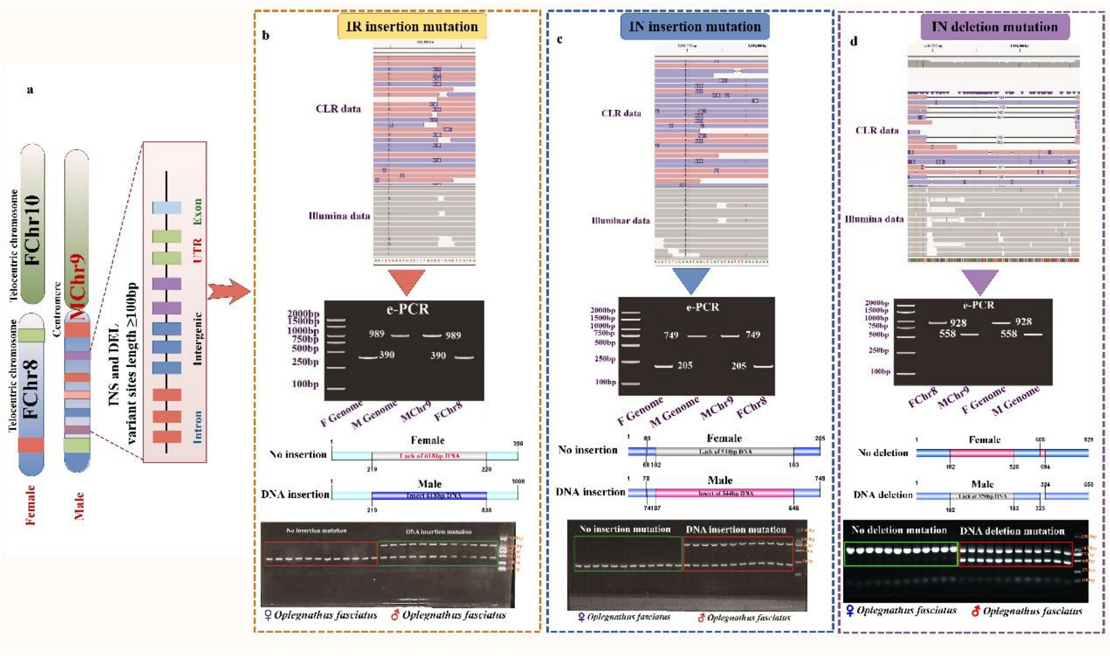

Figure 5: Representative genetic sex marker (insertion deletion variant sites) located in the intergenic region (IR), Intronic region (IN) of the *O. fasciatus* genome (STOSOd393e).

(a) Location of male and female sex markers in the male (MChr9) and female (FChr8) genomes. (b) Male and female sex genetic markers were located at loci in the intergenic region, using the female fish genome as a reference (Figure S2). Illumina and CLR clean data of bam comparison to the genome for insertion/deletion site validity detection. Red represented length long data from PacBio and gray represented was Illumina clean data. The e-PCR amplified bands were 390bp and 989bp in size, corresponding to DNA insertion and deletion patterns and agarose gel electrophoresis for identification of male and female genetically different populations. (c) By utilizing the female genome as a point of comparison, the genetic markers for male and female sexes were identified at specific insertion sites within *nuf2* gene introns (Figure S3, Figure S4). The resulting e-PCR amplified bands, measuring 205bp and 749bp in size, were indicative of distinct DNA insertion patterns and were utilized in agarose gel electrophoresis for the purpose of distinguishing between male and female genetically differentiated groups. Males exhibited two amplified bands, while females exhibited only one. (d) By utilizing the female fish genome as a point of comparison, the genetic markers for male and female sexes were situated within the deletion sites located in the introns of genes (Figure S5, Figure S6). The amplified bands produced through e-PCR were observed to be 558bp and 928bp in length, aligning with the DNA deletion patterns and agarose gel electrophoresis maps utilized for distinguishing genetically distinct groups of males and females. Specifically, two bands were amplified in males, while only one band was observed in females.

We apologize for the confusion caused by the lack of clarity of the results in Figures 4-8. In order to make the results clearer and more understandable, we have integrated the results with your comments, as shown in the figure, we have indicated the genomic origin of male and female sex markers, the types of variants and the results of their three-generation and two-generation data back-comparisons in the IGV in the integrated integration, and we have added the structural diagrams of the differences between male and female sequences and the structural results of e-PCR and PCR

agarose gel electrophoresis tests. We also added the structure map of male and female sequence differences and the structure map of e-PCR and PCR agarose gel electrophoresis test results. At the same time, we put Figure 4-8 as a supplementary file in the Supplementary Materials to assist in the understanding of the integration of the results of the graphs, of course, we also modified Figure 4-8, such as the differences in the information presented more clearly.

12. Enrichment analysis (l 365-426): This text consist almost entirely of a listing of categories that are also printed in the supplementary figures. As there is no clear conclusion to, or discussion of these results, I would suggest leaving the text out entirely.

Reply:

We thanks for your professional comments. All of we authors think your comments are very professional through discussion, the purpose of our paper is to build a high-throughput development technology process for male and female sex markers, and to obtain a catalog of male and female sex markers, and we have removed the gene enrichment content by combining your comments and the comments of the reviewers above. Thank you again for your professional opinion.

Some minor/textual remarks:

Lines 63-67 in the Abstract: repeats the same information twice.

Reply:

We thanks for your detailed comments. We revised "Our research extensively employed whole-genome variant site scanning and identification, high-throughput primer design for the target regions, and e-PCR batch amplification. By employing this comprehensive approach, which encompassed genome-wide variant site scanning, high-throughput primer design within the desired region, and e-PCR batch amplification and validation methodologies,.." as "**Our study utilized a thorough methodology involving whole-genome variant site scanning, high-throughput primer design for specific regions, and e-PCR batch amplification. Through this comprehensive approach, we effectively constructed a catalog containing insertion/deletion loci in large segments (>100 bp) for both male and female *O.***

fasciatus.".

Line 151-152: same statement twice.

Reply:

We thanks for your detailed comments. We revised "On the basis of the *O. fasciatus* large-banded variant loci, we used PCR e-simulation amplification technology to perform a genome-wide PCR e-simulation high-throughput amplification assay on the high-confidence large-banded variant loci dataset and to screen for single amplified-banded variant loci in both the female and male genomes." as "Utilizing the *O. fasciatus* sequence tagged sites as a foundation, we employed e-simulation amplification technology to conduct a genome-wide PCR assay aimed at identifying single amplified-banded variant loci between female and male genomes".

Line 156: PCR electron simulation -> simulated PCR.

Reply:

We thanks for your detailed comments. We revised "PCR electron simulation" as "simulated PCR".

Line 157: primer -> primer pair.

Reply:

We thanks for your detailed comments. We revised "primer" as "primer pair".

Figure 1: Typos Illuminar, onele thousand (?)

Reply:

We thanks for your detailed comments.

Line 179: Was BWA used for Illumina and Minimap2 for PacBio? Minimap2 needs a version number.

Reply:

We thanks for your detailed comments. As you said BWA software is used for Illumina data back comparison and Minimap2 is used for PacBio data back comparison. We have added a version of Minimap2 as Minimap2 (v2.24).

--

Please also take a moment to check our website at https://www.editorialmanager.com/giga/l.asp?i=154262&l=GJGQJFHZ for any additional comments that were saved as attachments. Please note that as GigaScience has a policy of open peer

review, you will be able to see the names of the reviewers.

In compliance with data protection regulations, you may request that we remove your personal registration details at any time. (Use the following URL: https://www.editorialmanager.com/giga/login.asp?a=r). Please contact the publication office if you have any questions.

Manuscript with markup version

Click here to access/download;Manuscript;MS with markup version.docx

Formatted: Left: 1.25", Right: 1.25"

Innovative approach for high-throughput exploiting sex-specific markers in japanese parrotfish *Oplegnathus fasciatus*~~A novel and scalable method for high-throughput and precise exploitation of sex-specific markers: a case study on *Oplegnathus fasciatus*~~

Yongshuang Xiao[1,2,3,4†], Zhizhong Xiao[1,2,3,4†], Yuting Ma[1,2,3,4†], Haixia Zhao[1,2,3,4†], Yanduo Wu[1,2,3,4†], Jinwei Huang[1,2,3,4], Pingrui Xu[1,2,3,4], Lin Liu[5*], Jing Liu[1*], Jun Li[1,2,3,4*]

[1]Center for Ocean Mega-Science, Institute of Oceanology, Chinese Academy of Sciences, Qingdao, China， [2]CAS and Shandong Province Key Laboratory of Experimental Marine Biology, Institute of Oceanology, Chinese Academy of Sciences, Qingdao, China， [3]Key Laboratory of Breeding Biotechnology and Sustainable Aquaculture, Chinese Academy of Sciences, Qingdao, China， [4]Laboratory for Marine Biology and Biotechnology, Qingdao National Laboratory for Marine Science and Technology, Qingdao, China, [5]Wuhan Frasergen Bioinformatics Co., Ltd. East Lake High-Tech Zone, Wuhan, China.

† Co-first authors

∗Correspondence address: Lin Liu, Wuhan Frasergen Bioinformatics Co., Ltd. East Lake High-Tech Zone, Wuhan, 430075, China; Tel: +86-053282896729; E-mail: liulin199306@163.com; Jing Liu, Institute of Oceanology, Chinese Academy of Sciences, 7 Nanhai Road, Qingdao, 266071, China; Tel: +86-053282898790; E-mail: jliu@qdio.ac.cn; Jun Li, Institute of Oceanology, Chinese Academy of Sciences, 7 Nanhai Road, Qingdao, 266071, China; Tel: +86-053282898718; E-mail: junli@qdio.ac.cn.

†Contributed equally to this work.

Yongshuang Xiao, ORCID: 0000-0002-1979-4555

Zhizhong Xiao, ORCID: 0000-0003-2403-1381

Yuting Ma, ORCID: 0000-0002-7158-9364
Haixia Zhao, ORCID: 0009-0003-8305-6009
Yanduo Wu, ORCID: 0000-0003-0060-1685
Jinwei Huang, ORCID: 0009-0003-5238-8318
Lin Liu, ORCID: 0009-0001-7315-2529
Jing Liu, ORCID: 0000-0001-8868-0386
Jun Li, ORCID: 0009-0005-2415-1895

Abstract

Background

The use of sex-specific molecular markers has become a prominent method in enhancing fish production and economic value, as well as providing a foundation for understanding the complex molecular mechanisms involved in fish sex determination. Over the past decades, research on male and female sex identification has predominantly employed molecular biology methodologies such as RFLP (Restriction Fragment Length Polymorphism), RAPD (Random Amplification of Polymorphic DNA), SSR (Simple Sequence Repeat), and AFLP (Amplified Fragment Length Polymorphism). The emergence of high-throughput sequencing technologies, particularly Illumina, has led to the utilization of SNP and InDel variants as significant molecular markers for investigating sex identification in fish. The advancement of sex-controlled breeding encounters numerous challenges, including the inefficiency of current methods, intricate experimental protocols, high costs of development, elevated rates of false positives, marker instability, and cumbersome field testing procedures. Nevertheless, the emergence and swift progress of PacBio high-throughput sequencing technology, characterized by its long read output capabilities, offers novel opportunities to overcome these obstacles. The utilization of sex-specific molecular markers has emerged as a significant technical approach to augment fish production and enhance economic value, while also establishing a fundamental basis for unraveling the intricate molecular mechanisms governing fish sex determination. In the past ten years, the field of genetic sex marker mining has primarily relied on first-generation development methods such as RFLP, RAPD, SSR, and AFLP, as well as second-generation techniques utilizing Illumina's SNP/InDel markers. However, the progress of sex-controlled breeding has been

Formatted: Font: 12 pt, Not Bold, English (United States)
Formatted: Line spacing: 1.5 lines
Formatted: Font: 12 pt, Not Bold, English (United States)
Formatted: Line spacing: 1.5 lines
Formatted: Font: 12 pt, Not Bold, English (United States)

hindered by several factors, including the limited efficiency of the aforementioned methods, complex experimental procedures, high development costs, a high incidence of false positives, unstable markers, and inconvenient on-site testing. Nevertheless, the emergence and rapid advancement of third-generation sequencing technology offers new opportunities for overcoming these limitations.

Findings

Utilizing male-female assembled genome information in conjunction with short-read sequencing data survey and long-read PacBio sequencing data, a catalog of large-segment (>100 bp) insertion-deletion genetic variants was generated through a genome-wide variant site scanning approach with bidirectional comparisons. The sequence tagging sites (STS) were ranked based on the long-read depth of the insertion deletion site, with markers exhibiting lower long read depth being considered more effective for large segment deletion variants. Subsequently, a catalog of bulk primers and simulated PCR for the male-female variant loci was developed, incorporating primer design for the target region and e-PCR technology. The Japanese parrotfish (*Oplegnathus fasciatus*), belonging to the Oplegnathidae family within the Centrarchiformes order, holds significant economic value as a rocky reef fish indigenous to East Asia. The criteria for rapid identification of male and female differences in *O. fasciatus* were established through agarose gel electrophoresis, which revealed two amplified bands for males and one amplified band for females. A high-throughput identification catalog of sex-specific markers was then constructed using this method, resulting in the identification of 3,639 (2,786 INS/853 DEL, ♀ as reference) and 3,672 (2,876 INS/833 DEL, ♂ as reference) markers in conjunction with 1,021 and 894 high-quality genetic sex identification markers, respectively. Sixteen differential loci were randomly chosen from the catalog for validation, with 11 of them meeting the criteria for male-female distinctions. The implementation of cost-effective and efficient technological processes would facilitate the rapid advancement of genetic breeding through expediting the high-throughput development of sex genetic markers for various species.

Using male-female linear genomic information combined with Illumina survey and PacBio CLR / CCS data, a database of large-segment (>100 bp) insertion-deletion genetic variants was constructed using a genome-wide variant site scanning method with bidirectional comparisons. A database of bulk primers and simulated PCR for the

male-female variant loci were then constructed, employing primer design for the target region and e-PCR technology. Finally, the criteria for rapid identification of male and female differences were established based on agarose gel electrophoresis with two amplified bands for males and one amplified band for females. A high-throughput identification database of sex-specific markers for *Oplegnathus fasciatus* was constructed utilizing this method, yielding 3,645 (2,791 INS/854 DEL, ♀ as reference) and 3,872 (3,039 INS/833 DEL, ♂ as reference) genetic sex identification markers. Four differential loci were randomly selected from the database for validation, and the results met the criteria for male-female differences. Utilization of this technology will accelerate the identification of genetic sex markers for species, contributing to the rapid development of genetic breeding.

Conclusions

Our study utilized assembled genome information from male and female individuals obtained from PacBio, in addition to data from short-read sequencing data survey and long-read PacBio sequencing data. We extensively employed genome-wide variant site scanning and identification, high-throughput primer design of target regions, and e-PCR batch amplification, along with statistical analysis and ranking of the long reads depth of the variant sites. Through this integrated approach, we successfully compiled a catalog of large insertion/deletion sites (>100 bp) in both male and female *O. fasciatus*.By utilizing the linear genomic information of male and female individuals acquired through PacBio sequencing, along with data from Illumina surveys and PacBio CLR/CCS. Our research extensively employed whole-genome variant site scanning and identification, high-throughput primer design for the target regions, and e-PCR batch amplification. By employing this comprehensive approach, which encompassed genome-wide variant site scanning, high-throughput primer design within the desired region, and e-PCR batch amplification and validation methodologies, we successfully developed a database encompassing insertion/deletion loci in large segments (>100 bp) for both male and female *O. fasciatus*.

***Keywords*:** large-segment insertion/deletion; bulk primers design; e-PCR technology; *Oplegnathus fasciatus*; high-throughput identification

Data description

Introduction

Sexual dimorphism ~~is~~ was common in the animal kingdom, but the mechanisms of sex determination ~~are~~ were diverse [1, 2]. Thus, sex research has always been a topic of interest in biology. Fish ~~occupy~~ occupied a key position in the vertebrate evolutionary system, and because of their wide distribution, large number of species and diverse sex determination mechanisms, they ~~are~~ were an important target for the study of sex determination mechanisms [3-9]. However, most fish have a low degree of differentiation between the sex chromosomes, which ~~is~~ was difficult to detect from the morphology of the chromosomes [10]. The development of sex-specific molecular markers and sex-control biotechnology has provided an important technical way to increase fish production and economic value and has also laid the foundation for deciphering the molecular mechanism of fish sex determination [11]. Researchers have succeeded in developing sex-specific or correlated molecular markers in many fish species using a variety of techniques, including SSR, RAPD, AFLP, SNP and In Del [5, 6, 12-17]. However, these methods for obtaining sex-specific markers have many problems, such as low throughput, single markers, cumbersome development and experimental procedures, high development costs, low amplification efficiency and inconvenient on-site detection. The first issue to be addressed was to establish a high-throughput and easy-to-detect marker procedure for sex-specific identification in the investigation of sex dimorphism, mechanisms of sex differentiation, and development and use of sex-producing traits.

In recent years, with the rapid development of genome sequencing technology and the significant decrease in the cost of sequencing, it has become possible to perform whole-genome sequencing of important cultured species. Rapid developments in next-generation sequencing (NGS) technologies have created significant opportunities for the discovery and use of species germplasm resources [4]. In 2002, the genome sequencing of *Takifugu rubripes* was successfully accomplished, marking it as the pioneering model fish species to undergo complete genome sequencing ~~In 2002, the genome sequencing of *Takifugu rubripes* was completed, making it the first model fish to have its entire genome sequenced~~ [18]. Subsequently, over the course of nearly 16 years, the whole-genome sequencing of more than 40 fish species, including *Oryzias latipes*, *Gasterosteus aculeatus*, *Pelteobagrus fulvidraco*, *Mola mola*, *Nothobranchius*

furzeri, and *Salmo salar*, has been carried out on a national and international scale ~~Since then, whole genome sequencing of more than 40 fish species, such as *Oryzias latipes*, *Gasterosteus aculeatus* and *Salmo salar*, has been completed both nationally and internationally~~ [19-30]. Over the span of approximately 16 years until 2018, the majority of fish genomes were sequenced utilizing second-generation sequencing technology, specifically Illumina ~~Most of these fish genomes have been sequenced using second-generation sequencing (Illumina), and although second-generation sequencing technology has high throughput, the sequencing read lengths are relatively short~~ [4, 18-21]. Despite the high throughput capabilities of second-generation sequencing, the relatively short sequencing read lengths posed challenges in obtaining precise genome maps and accurate information on chromosome structural variation. This limitation was especially pronounced in genomes with high complexity and repetitive regions.~~Therefore, it is difficult to obtain high-quality genome maps and accurate high-throughput information on chromosome structural variation by relying on second-generation sequencing technology alone. This is particularly true for genomes that are highly complex and have highly repetitive regions.~~

At present, third-generation sequencing technology with high precision of long fragments represented by single-molecule real-time sequencing has matured, which included Pacific Biosciences' (PacBio) single-molecule real-time sequencing technology (SMRT)~~includes Helicos' single-molecule sequencing technology (SMSTM)~~, Oxford Nanopore's single-molecule nanopore sequencing technology, ~~Pacific Biosciences' (PacBio) single-molecule real-time sequencing technology (SMRT),~~ and so on. Among them, PacBio RS sequencing technology ~~is~~ was the most widely used and commercialized core third-generation sequencing technology and has been widely used in the genome assembly of several species, such as an Asian man, *Oropetium thomaeum* and *Lates calcarifer* [31-33]. The initial assembly of the human telomere-to-telomere (T2T) CHM13 genome, utilizing PacBio HiFi and Oxford Nanopore ultralong-read sequencing, achieved a contig N50 of 154.26 Mb, resulting in a seamless assembly that fills a crucial gap in the human DNA genetic code map ~~For the Chinese human genome sequenced by PacBio technology, the assembly level reached a contig N50 of 8.3 Mb, which met the standard of the completed map~~ [31, 34-36]. In addition, Hi-C technology based on next-generation sequencing technology has gradually become a favourable tool for chromosome assembly [37]. With the rapid development of sequencing technology and the improvement of assembly technology,

the genome integrity and accuracy have been greatly improved, especially in marine fishes, and the assembly now obtained Contig N50 up to the level of 28 Mb for *Sciaenops ocellatus* [38]. Thus, the development of next-generation sequencing technologies has made it possible to exploit high-throughput sex-specific markers in high-precision assembled genome~~linear genomic~~ data.

Oplegnathus fasciatus (Temminck & Schlegel, 1844, Fishbase ID: 1709) ~~is~~ was a rocky reef fish distributed in a wide range of shallow waters around Korea, Japan, China and Hawaii [39-42]. *O. fasciatus* has become an important fishery resource for offshore cage culture and fish stocking in marine ranches in China, Japan and Korea [41, 42]. *O. fasciatus* has been a commercially important species for sashimi production and recreational angling, with engineered aquaculture in China yielding ex-factory prices of up to US$30/kg [42]. Meanwhile, *O. fasciatus* ~~is~~ was characterized by a multiple $X_1X_1X_2X_2/X_1X_2Y$ sex chromosome system [43-46]. Sexual dimorphism has been observed in *O. fasciatus*, with males growing faster than females, especially at weights greater than 500 grams [7, 8, 45, 46]. Therefore, the breeding of new strains with a male predominance has become one of the goals for future breeding of *O. fasciatus*. The development of appropriate genetic sex markers ~~is~~ was a prerequisite for achieving a new variety of *O. fasciatus* with male predominance. Currently, available markers for the genetic characterization of *O. fasciatus* ~~are~~ were extremely limited and have mainly been identified by AFLP, SSR and Illumina technologies, which constrained the process of exploiting new varieties of *O. fasciatus* [47-49]. The completion of the female and male genomes of *O. fasciatus* has enabled high-throughput development of large segments to visualize genetic sex markers from a genome-wide perspective.

In this work, a catalog~~database~~ of genomic differences between females and males was constructed based on the assembled genome~~linear genomic~~ information of *O. fasciatus* by using bidirectional whole-genome sequence alignment [50, 51]. Using the difference catalogs~~database~~, we obtained datasets of insertion and deletion variant sites larger than 100 bp by screening and used short-read sequencing data survey~~Illumina data~~ from genome sequencing and long-read PacBio sequencing~~CLR/CCS~~ data to test the confidence of large segmental variant sites [52]. Utilizing the *O. fasciatus* sequence tagged sites as a foundation, we employed e-simulation amplification technology to conduct a genome-wide PCR assay aimed at identifying single amplified-banded variant loci between female and male genomes~~On the basis of the *O. fasciatus* large-banded variant loci, we used PCR e-simulation amplification technology to perform a~~

~~genome-wide PCR e-simulation high-throughput amplification assay on the high-confidence large banded variant loci dataset and to screen for single amplified banded variant loci in both the female and male genomes~~ [52]. Then, we performed PCR amplification and agarose gel electrophoresis for the mutant loci detected by simulated PCR~~PCR electron simulation~~. Since *O. fasciatus* exhibited~~exhibits~~ an $X_1X_1X_2X_2/X_1X_2Y$ sex chromosome system, a single primer pair~~primer~~ amplifies two bands in males and one band~~primer~~ in females.

The paper was organized as follows: First, a fast scanning method for female and male assembled genome~~linear genomic~~ variant loci was introduced, and an insertion/deletion dataset of large segments was constructed. Using the constructed insertion/deletion dataset of large fragments, high-throughput candidate molecular marker loci for female and male genetic sex identification of *O. fasciatus* were identified. Then, the new technique of PCR electronic simulation with bulk amplification was used to perform simulated amplification of high-throughput candidate molecular marker loci to screen primers for the single amplification of the female and male genomes with amplification differences. Finally, a high-throughput precision marker dataset for male and female genetic sex identification was obtained and tested by agarose gel electrophoresis (Figure 1).

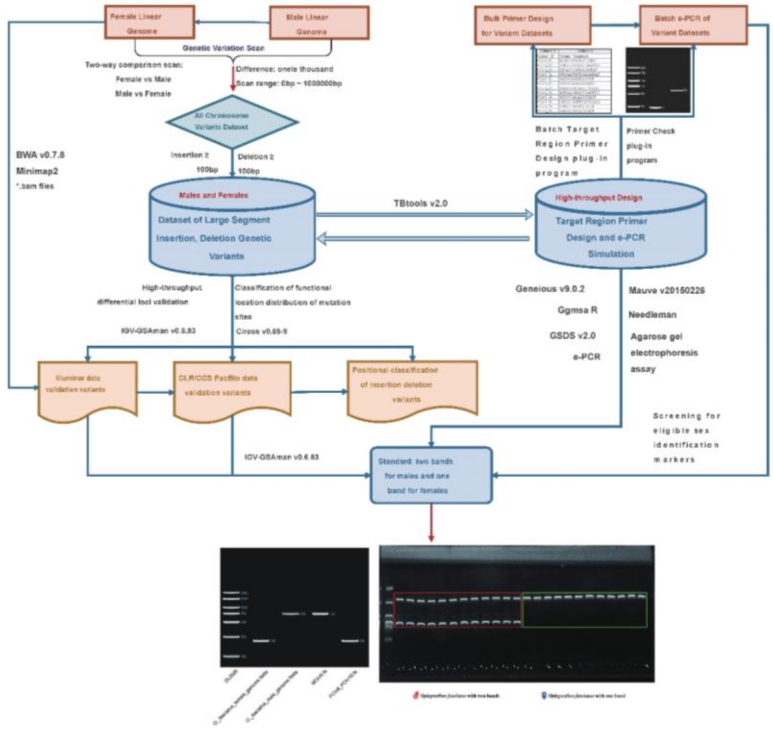

Female Linear Genome
Male Linear Genome
Genetic Variation Scan
Two-way comparison scan:
Female vs Male
Male vs Female
Difference: onele thousand
Scan range: 0bp ~ 1000000bp
All Chromosome Variants Dataset
BWA v0.7.8
Minimap2
*.bam files
Insertion ≥ 100bp
Deletion ≥ 100bp
Males and Females
Dataset of Large Segment Insertion, Deletion Genetic Variants
TBtools v2.0
High-throughput Design
Target Region Primer Design and e-PCR Simulation
Bulk Primer Design for Variant Datasets
Batch e-PCR of Variant Datasets
Batch Target Region Primer Design plug-in program
Primer Check plug-in program
High-throughput differential loci validation
Classification of functional location distribution of mutation sites
IGV-GSAman v0.6.83
Circos v0.69-9
Geneious v9.0.2
Ggmsa R
GSDS v2.0
e-PCR
Mauve v20150226
Needleman
Agarose gel electrophoresis assay
Illumina data validation variants
CLR/CCS PacBio data validation variants
Positional classification of insertion deletion variants
Screening for eligible sex identification markers
IGV-GSAman v0.6.83
Standard: two bands for males and one band for females

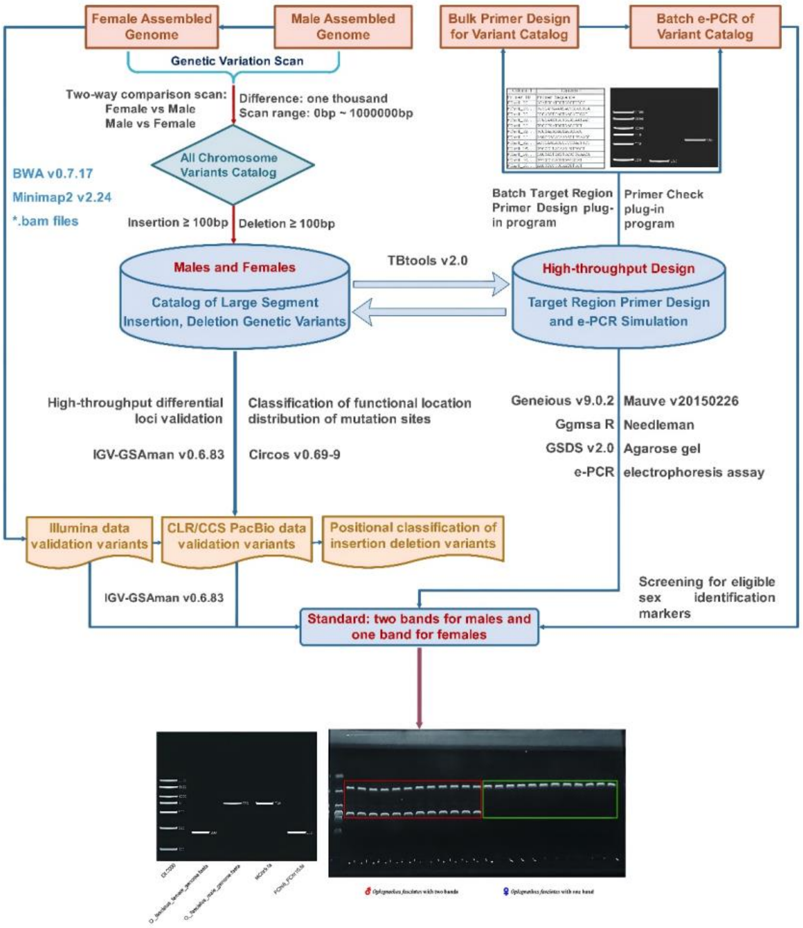

Figure 1: High-throughput exploitation process of genetic sex markers for *O. fasciatus*

Table 1 Parameters for genome sequencing and assembly quality assessment of chromosome-level genomes of male and female *O. fasciatus*

Illumina platform (Short-read sequencing data survey)		
	Male *O. fasciatus*	Female *O. fasciatus*
Reads number	432,091,798	613,163,122
Raw data (bp)	63,910,932,339	90,702,298,813
Clean data (bp)	56,617,669,600	80,887,039,002
Read length (bp)	148, 147	148, 147

Q20 (%)	97.9, 97.3	98.0, 97.1
Q30 (%)	93.8, 92.0	94.1, 91.4
PacBio Sequel platform (Long-read PacBio sequencing data)		
	Male *O. fasciatus*	Female *O. fasciatus*
Polymerase read number	3,674,737	4,760,021
Polymerase read bases (bp)	39,844,228,109	62,877,786,278
Average polymerase read length (bp)	10,847	13,219
Polymerase read N50 (bp)	18,850	22,964
Subreads number	4,711,720	6,689,662
Total bases of subreads (bp)	39,793,172,442	62,783,888,050
Average subread length (bp)	8,448	9,397
Length of genome (bp)	762,267,613	768,808,243
Number of contigs	1,355	1,372
Contigs N50 (bp)	2,183,645	2,130,780
Number of Scaffolds	23	24
Scaffold N50 (bp)	32,431,321	33,548,962
Genome coverage (X)	251.1	314.6
Complete BUSCOs (%)	98.2	98.0

Note： male and female genomic quality parameters of *O. fasciatus* [45, 46].

Formatted: Font: Not Bold

Formatted: Font: Italic

Formatted: Font: Not Bold

Materials and Methods

Sample collection, sequencing and genome quality

Male and female *O. fasciatus* captured from Qingdao, Shandong Province (Yellow Sea) were utilized for genome sequencing and assembly. High-quality genomic DNA was extracted from fresh muscle tissue and blood samples from pairs of male and female *O. fasciatus* using the Illumina platform (Illumina Inc., San Diego, CA, USA) and PacBio Sequel (Pacific Biosciences of California, Menlo Park, CA, USA) [45, 46]. Genomic DNA from fresh muscle tissue and blood samples of male and female *O. fasciatus* pairs was extracted to construct short insert libraries (300~350 bp) for males and females,

respectively, using standard protocols provided by the Illumina HiSeq X Ten platform (Illumina Inc., San Diego, CA, USA). Raw reads totaling approximately 90.7 Gb and 63.9 Gb were generated. After removing splice-containing sequences, low-quality, and redundant reads, approximately 80.8 Gb and 56.6 Gb of pure data were obtained from the genomes of males and females, respectively, corresponding to Q20 averages of 97.55% and 97.60%, respectively (Table 1). Genomic DNA libraries (20 kb) for male and female fish were then constructed using the PacBio Sequel platform (Pacific Biosciences of California, Menlo Park, CA, USA) (Table 1). Sequencing was performed on the PacBio Sequel platform using the Sequel Binding Kit 2.0, Sequel Sequencing Kit 2.1, and Sequel SMRT Cell 1M v2. Seven and five SMRT Cells were used for sequencing the male and female genomes, respectively, with SMRT LINK 5.0 used to filter the raw data from the zero-mode waveguide. We obtained 62.8Gb and 39.8Gb of long read length PacBio sequencing data for female (accession SRP160016) and male (accession SRP220007) genome assembly, respectively [45, 46]. The female and male *O. fasciatus* genome were de novo assembled using Canu v1.4 with Corrected-Error-Rate set to 0.040, followed by redundancy removal using Redundans v0.13c (minCoverage = 15), genome polishing with Arrow tool in SMRT Link 5.0, and final assembly refinement with Pilon using Illumina NGS short reads from the genome surveys. Based on the male and female draft genomes and Hi-C technology, we obtained high-quality genomes at the chromosome level of female (768.8Mb, accession SUB14064808) and male (762.2Mb, accession SUB14065793) *O. fasciatus*, and the contig N50 and scaffold N50 of the male and female fish genomes reached 2.13 Mb/2.18 Mb and 33.5 Mb/32.4 Mb, respectively [45, 46]. To assess the genomic completeness of assembled male and female *O. fasciatus*, we subjected the assembled sequences to BUSCO version 5 evaluation (BUSCO, actinopterygii_odb10), with Complete BUSCOs assessed values up to 98.2% and 98.0% for male and female genomes, respectively (Table 1).

Large fragment variant loci scanning and identification

We employed the genome variant site scanning plugin with accompanying parameters (diff: oneInThousand, VarRange: 0~1,000,000 bp, BatchSize: 500 bp, Min Align

Length for Cov Calc 10,000 bp, Min Align Length for Var Calling: 50000 bp) in TBtools (v 2.0) software to conduct bidirectional comparisons of the complete genomic sequences of male and female organisms [52, 53]. Additionally, we established catalogs~~databases~~ containing genomic disparities between males and females. To classify base substitution, insertion, and deletion sites, as well as to screen large insertion-deletion sites (>100 bp) against a differential catalogs~~database~~, we utilized the Table Row Extract program. In order to further evaluate the stability and accuracy of the dataset containing large segment insertion deletion sites, we employed SyRI software (python3 syri_env/bin/syri -c out.filtered.coords -d out.filtered.delta -r FM.fasta -q genome.format.fa --nosnp) to autonomously detect variant sites (≥100bp) in the assembled genomes of both male and female individuals, and subsequently compared these sites with our existing dataset of variant sites. Additionally, we utilized BWA (v 0.7.8) (bwa-0.7.17/bwa mem -t 6 -SP genome.fa M2_1P.fq.gz M2_2P.fq.gz | samtools view -bS > alignment.bam) and Minimap2 (v2.24) (minimap2 –ax map-pb –t 8 genome.fasta M1.subreads.fasta > M1.subreads.sam) softwares to align the short-read sequencing data generated from Illumina platform and the long-read sequencing data obtained from PacBio platform to the male and female genomes, respectively, in order to validate the large segment variant sites ~~. Furthermore, we utilized BWA (v 0.7.8) and Minimap2 software to align the survey data obtained through Illumina sequencing and the CLR/CCS data reads obtained through PacBio sequencing to the male and female genomes using default parameters to conduct a large-scale comparison as a means of validation~~ [54, 55]. To further improve the exploitation efficiency of sex-specific markers based on long reads, we calculated the read depth of long reads of insertion deletion sites, and then ranked the insertion deletion site markers based on the depth of long reads, for large segment deletion variants, the lower the depth of long reads, the more effective the markers were, so we constructed a frequency map of the distribution of the depth of long reads of the large segment deletion sites of the male and female genomes, and then classified the markers. Subsequently, employing the Batch Target Region Primer Design plug-in and e-PCR plug-in in TBtools (v 2.0) software, we designed primers for high-throughput amplification and screened them for

single amplification bands at the insertion and deletion sites specific to the male and female genomes [52]. To identify the origin of large segment insertion deletion sites, we evaluated whether the variant sites in the dataset were transposons by integrating RepeatModeler (v 2.0.1) and RepeatMasker (v 4.1.2) softwares. The results of transposon determination obtained by the two softwares were combined and the duplicates were removed for all transposon identification results. At the same time, we utilized TRF (v 4.09.1) software to evaluate whether the variant sites in the dataset were tandem repeats.

The positional distribution of female and male genetic variant loci on the genome was depicted using Circos (v 0.69-9) software [56]. Homology comparison and visualization of insertion-deletion sites with male and female linear genomes were conducted using a combination of Geneious (v 9.0.2) software and Mauve program (v 20150226) [57, 58]. The comparison of genetic sex marker sequences with the Global Alignment standard was performed using the Needleman Wunsch plug-in in TBtools (v 2.0) [52]. Additionally, the visualization of genetic sex marker variant site regions was accomplished using Ggmsa program [59]. GSDS (v 2.0) software was utilized to illustrate the location of the variant site within the functional region of the gene [60]. We performed functional enrichment analysis using Metascape (https://metascape.org/) to identify enriched Gene Ontology (GO) and Kyoto Encyclopedia of Genes and Genomes (KEGG) pathways associated with genes harbouring significant insertion/deletion variants within extensive segments of the exon region(Zhou et al., 2019). The analysis was conducted with specific parameters, including a minimum overlap of 1, a *p value* cut-off of 0.05, and a minimum enrichment threshold of 1.5. A total of 24 tails from both male and female *O. fasciatus* specimens were collected and distinguished through gonadal histology. Simultaneously, we gathered 93 tails from wild population samples of *O. fasciatus* along the coasts of Zhejiang and Shandong Provinces, China, to verify the efficacy of sex markers across various geographic regions. The reliability of the genetic sex markers was assessed by randomly identifying male and female populations using e-PCR in conjunction with agarose gel

Formatted: Font: Italic

electrophoresis.

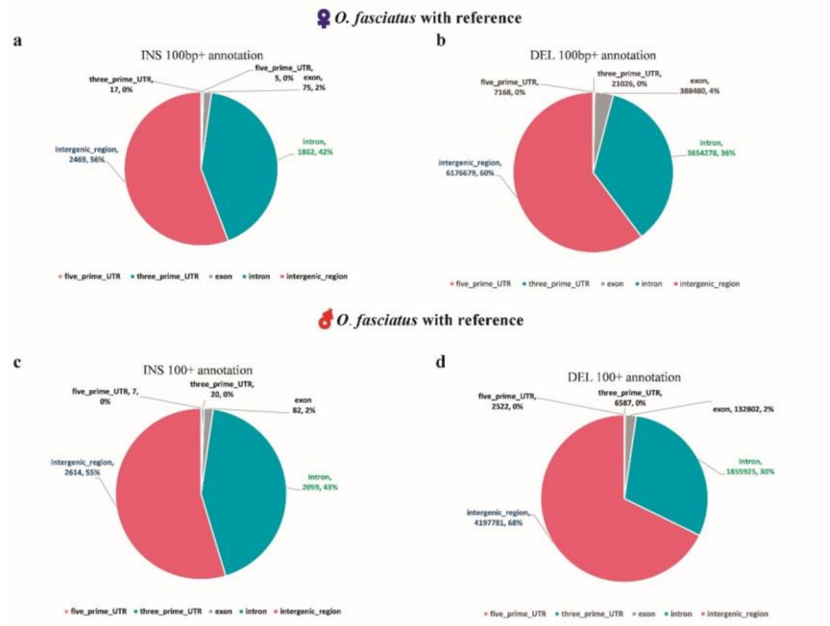

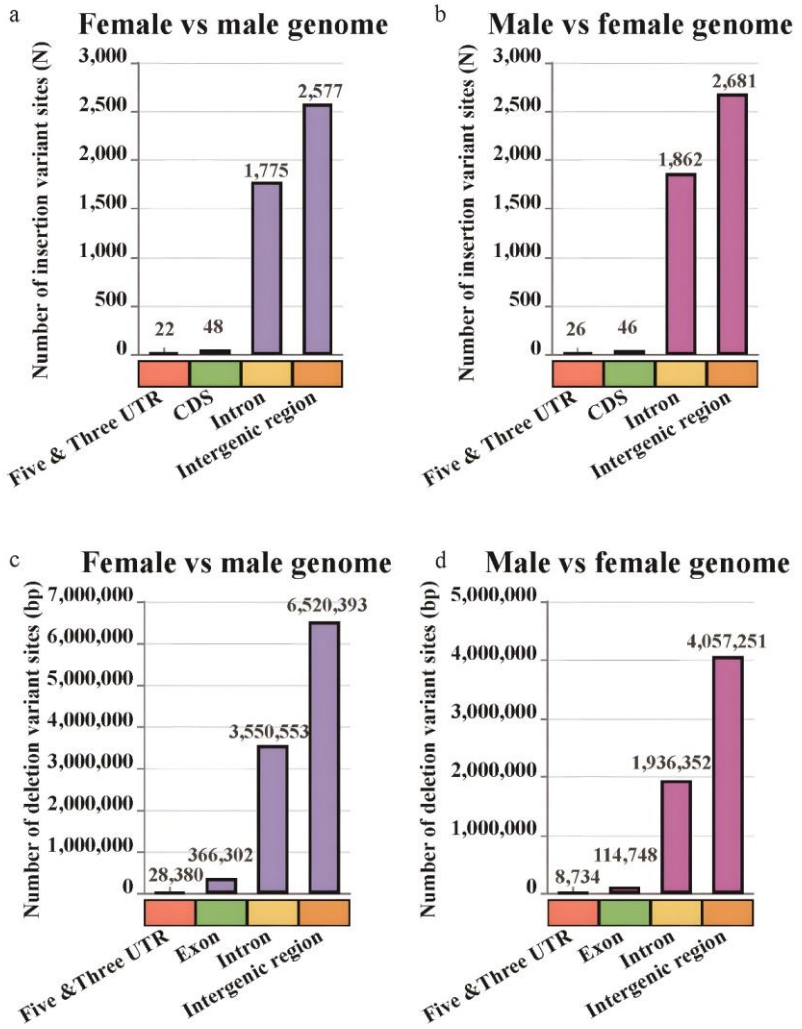

Figure 2: Distribution statistics of insertion and deletion fragment (＞100bp) positions in genomic functional regions of *O. fasciatus*.

(a) Distribution statistics of insertion variant loci using the female fish genome as a reference. (b) Distribution statistics of insertion variant loci utilizing the male fish genome as a point of reference. (c) Distribution statistics of deletion variant length using the female fish genome as a reference. (d) Distribution statistics of deletion variant loci using the male fish genome as a reference.

Note: The colors red, green, yellow, and orange represented the UTR, CDS, intron, and intergenic regions. The frequency of insertions (a, b) was quantified based on the number of events, while deletions (c, d) were characterized by their length in nucleotides.

~~**Figure 2:** Distribution statistics of insertion and deletion fragment positions in genomic functional regions of *O. fasciatus*.~~

Formatted: Font: (Default) Times New Roman

~~(a) Distribution statistics of insertion variant loci using the female fish genome as a reference. (b) Distribution statistics of deletion variant length using the female fish genome as a reference. (c) Distribution statistics of insertion variant loci using the male fish genome as a reference. (d) Distribution statistics of deletion variant loci using the male fish genome as a reference.~~

Results

Construction of insertion/deletion variant loci datasets for large segments

Using the female genome as a reference, we obtained 1,919,620 male and female differential variant sites with unfiltered data, including 191,915 insertion sites, 325,620 deletion sites and 1,402,085 nucleotide substitutions (Supplementary Female_reference_all_variation_Set.xls)~~(Figure 2)~~. To screen suitable field-actionable markers for large-band genetic sex identification, we screened this insertion/deletion variant site catalog~~database~~ for variants larger than 100 bp and obtained 4,422~~4,428~~ insertion sites with a differential sequence size of 5,908,147~~6,003,658~~ bp, of which 0.11% were located in 5′ UTRs, 0.38% in 3′ UTRs, 40.14~~42.05~~% in intronic regions, 1.08~~1.69~~% in CDS~~exonic~~ regions, and 58.28~~55.76~~% in intergenic regions (Figure 2, Figure S1， Supplementary Female_reference_INS/DEL100_Set.xlsx). In addition, we obtained 4,675~~4,691~~ deletion sites with a differential sequence size of 10,465,628~~10,247,631~~ bp (Supplementary Female_reference_INS/DEL100_Set.xlsx). As the deletion variant sites spanned multiple genomic functional regions, we performed statistical analysis of the actual locations where the deletion bases were located, with 0.08~~0.07~~% located in 5′ UTRs, 0.19~~0.21~~% in 3′ UTRs, 33.93~~35.66~~% in intronic regions, 3.50~~3.79~~% in CDS~~exonic~~ regions, and 62.30~~60.27~~% in intergenic regions (Figure 2, Table 2~~1~~). The highest number of chromosomal mutations occurred in female fish chromosomes 8 and 10, with 333~~335~~ and 341~~344~~ insertion and deletion sites, respectively (Figure 3).

Using the male *O. fasciatus* genome as a reference, 1,926,295 female–male differential variant sites were obtained with unfiltered data, including 326,823 insertion sites, 192,672 deletion sites and 1,406,800 nucleotide substitution sites (Supplementary Female_reference_all_variation_Set.xls~~Figure 2~~). Through large fragment screening (>100 bp), we obtained 4,615~~4,782~~ insertion sites with a differential sequence size of 10,100,983~~10,447,125~~ bp, of which 0.13~~0.15~~% were located in 5′ UTRs, 0.43~~0.42~~% in 3′ UTRs, 40.35~~43.06~~% in intronic regions, 1.00~~1.72~~% in CDS~~exonic~~ regions, and 58.09~~54.66~~% in intergenic regions (Figure 2, Figure S1, Table ~~1~~2, Supplementary Male_reference_INS100_Set.xlsx). The chromosome with the highest number of mutations was chromosome 9 in male *O. fasciatus*, in which the number of insertion

and deletion sites were 345~~354~~ and 333~~345~~, respectively (Figure 3); male chromosome 9 ~~is~~was homologous to chromosomes 8 and 10 of female *O. fasciatus*. Male chromosome 6 had the second highest number of mutations, with 289 insertion sites and 256 deletion sites (Figure 3).

~~**Table 1.** Statistics of the positional distribution of insertion/deletion variants in the male and female genomes of *O. fasciatus*.~~

~~Type of gene function region~~	~~Female genome as a reference~~		~~Male genome as a reference~~	
	~~Insertion variant loci no. (>100 bp)~~	~~Deletion variant length (>100 bp)~~	~~Insertion variant loci no. (>100 bp)~~	~~Deletion variant length (>100 bp)~~
~~5′ UTR~~	~~5~~	~~7,168~~	~~7~~	~~2,522~~
~~3′ UTR~~	~~17~~	~~21,026~~	~~20~~	~~6,587~~
~~Exon~~	~~75~~	~~388,480~~	~~82~~	~~132,802~~
~~Intron~~	~~1,862~~	~~3,654,278~~	~~2,059~~	~~1,855,925~~
~~Intergenic region~~	~~2,469~~	~~6,176,679~~	~~2,614~~	~~4,197,781~~
~~Total~~	~~4,428~~	~~10,247,631~~	~~4,782~~	~~6,195,617~~

Table 2. Statistics of the positional distribution of insertion/deletion variants in the male and female genomes of *O. fasciatus*.

Type of gene function region	Female genome as a reference		Male genome as a reference	
	Insertion variant loci no. (>100 bp)	Deletion variant length (>100 bp)	Insertion variant loci no. (>100 bp)	Deletion variant length (>100 bp)
5′ UTR	5	7,859	6	2,482
3′ UTR	17	20,521	20	6,252
CDS	48	366,302	46	114,748
Intron	1,775	3,550,553	1,862	1,936,352
Intergenic region	2,577	6,520,393	2,681	4,057,251
Total	4,422	10,465,628	4,615	6,117,085

Note: Insertions (>100 bp) were summarized by number of events. Deletions (>100 bp) were summarized by length in nucleotides.

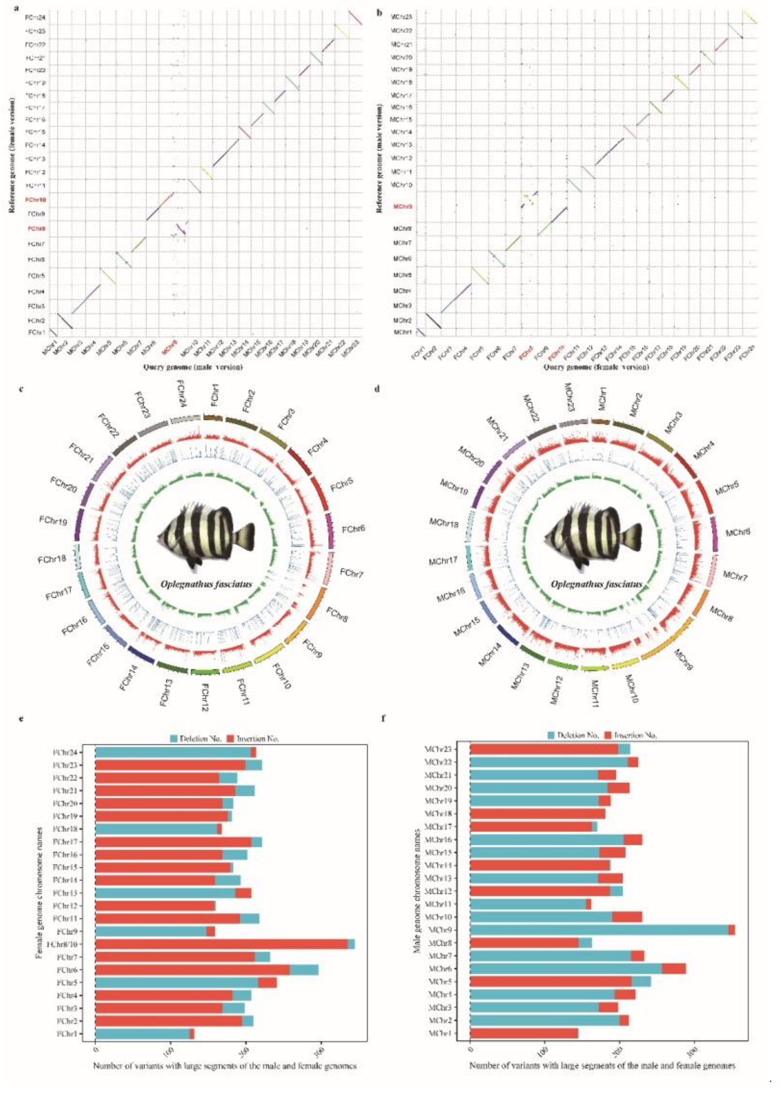
a
Reference genome (female version)
Query genome (male version)
b
Reference genome (male version)
Query genome (female version)
c
Oplegnathus fasciatus
d
Oplegnathus fasciatus
e
Deletion No.
Insertion No.
Female genome chromosome names
Number of variants with large segments of the male and female genomes
f
Deletion No.
Insertion No.
Male genome chromosome names
Number of variants with large segments of the male and female genomes

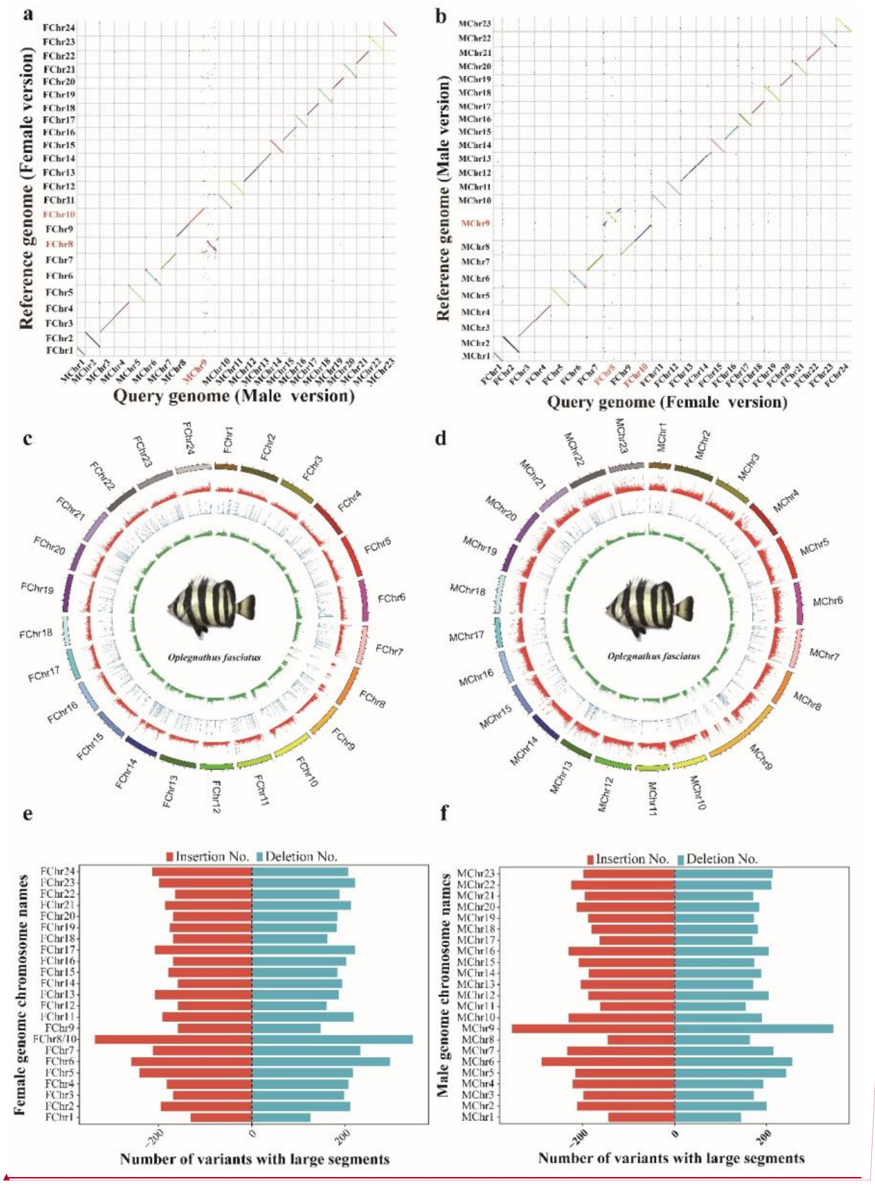

Figure 3: The covariance of genomic sequence differences between the male and female genomes of *O. fasciatus* and distribution statistics of variant sites.

(a) The female genome (reference) of *O. fasciatus* vs. the male genome for homology comparison using the minimap2 software with a 5000 bp sliding window. The female genome was the vertical axis, and female chromosomes 8 and 10 were homologous to male chromosome 9. (b) The comparison of homology between the male genome of *O. fasciatus* and the female genome was conducted using the minimap2 software with a 5000 bp sliding window. The male genome was represented on the vertical axis, revealing homology between male chromosome 9 and female chromosomes 8 and 10. (c) Distribution statistics of variant loci using the female fish

Formatted: Font: (Default) Times New Roman

genome as a reference. Insertions (>100 bp) were summarized by number of events. Deletions (>100 bp) were summarized by length in nucleotides. From outer to inner circles were the chromosome name, positional distribution of insertion sites, base length distribution of deletion sites, and base substitution distribution, respectively. (d) Distribution statistics of variant loci using the male fish genome as a reference. (e) - (f) Dual-valued histograms corresponding to the Table 3 showed the number of insertion/deletion sites per chromosome with reference to the female (4,422 inertion sites, 4,675 deletion sites) and male (4,615 inertion sites, 4,385 deletion sites) genomes, respectively, where chromosome 9 in males was homologous to chromosomes 8 and 10 in females. Insertion sites were represented by negative values, and deletion sites were represented by positive values.

The SyRI software was further utilized to assess variant sites (＞100bp) between the male and female reference genomes of *O. fasciatus*. By utilizing the male genome as a reference for comparison with the female genome, a total of 2,045 deletion sites (＞100bp) and 2,110 insertion sites (＞100bp) were identified. Additionally, comparison of the female genome with the male genome as a reference revealed 2,116 large segment deletion sites (＞100bp) and 2,055 large segment insertion sites (＞100bp). Considering the long-read length depth calculations and ranking large segments of deletion sites were beneficial when utilizing short-read sequencing data and long-read PacBio sequencing data to analyze visual differences between male and female genomes. Our study revealed that 1,791 deletion sequence tag sites identified through SyRI scanning overlapped with deletion sites identified in the female fish reference genome, accounting for 84.64% of the total 2,116 deletion sites identified (Fig.4a, Table 3). Meanwhile, the SyRI scanning method identified 1749 deletion STS that intersected with deletion sites in the reference genome of male fish, representing 85.53% of the total 2,045 deletion sites (Fig.4b, Table 3). In the context of large deletion variants, a correlation was observed between the long-read depth and the effectiveness of markers. A frequency distribution map was created for the long-read depths of large deletion loci within male and female genomes, followed by marker categorization. The results indicated that markers with long-read depths below 5.48 (895, ♀ as reference) and 12.50 (1,021, ♂ as reference) demonstrated high recognition rates as sex markers, based on the male and female genomes as reference (Fig.4c-f). The findings further indicated that a substantial proportion of deletion sequence tag sites referencing the female genome were situated in transposon regions (78.44%, n=3,667) and tandem repeat sequences regions (33.67%, n=1,574). Similarly, statistical analysis revealed that a notable percentage of deletion sequence tag sites referencing the male genome were linked to transposon regions (77.08%, n=3,380) and tandem repeat regions (31.90%, n=1,399) (Table 4).

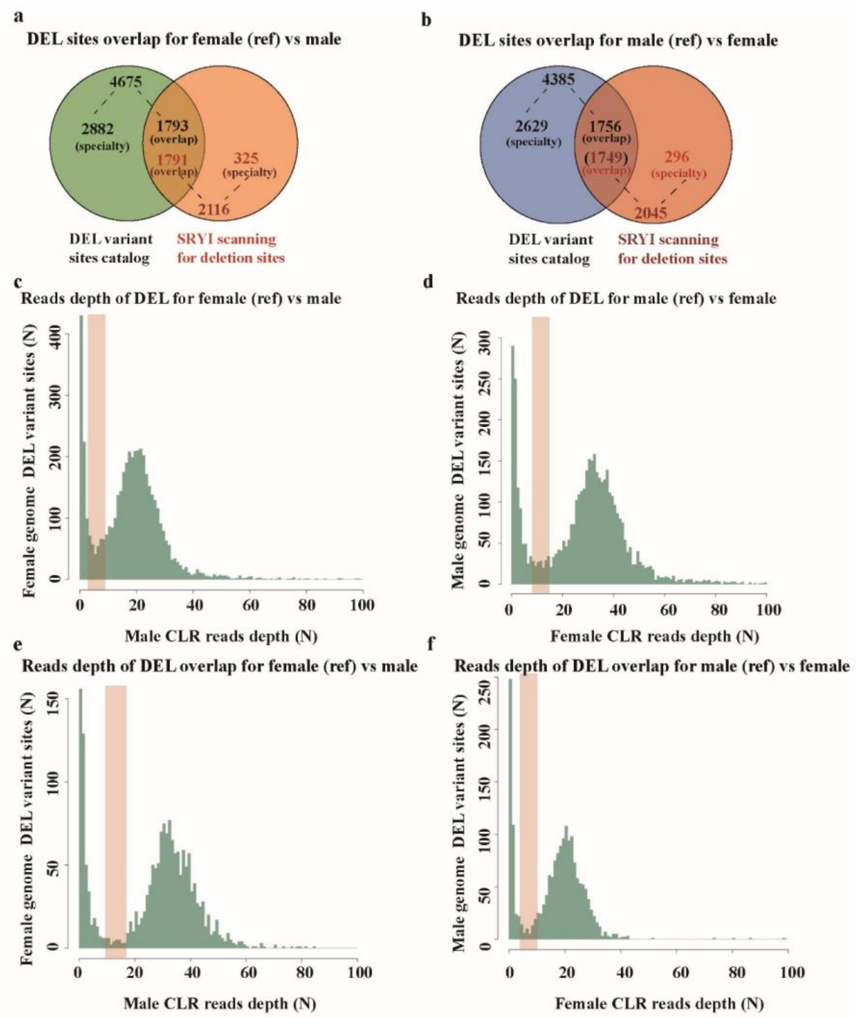

Figure 4: Intersection results of two variant site scanning methods and ranking of variant sites with long read depth frequency distributions

Table 2. Statistics of large-segment differential loci after primer design, e-PCR amplification, and simulated amplification products and size screening.

Reference genome	Mutation type	Variant length (>100 bp)	High-throughput design of primers and e-PCR	Single band and suitable amplification size (100~2000 bp)
Female vs. Male	Insertion	4,428	5,044	2,791
	Deletion	4,691	1,628	854
Male vs. Female	Insertion	4,782	5,505	3,039
	Deletion	4,520	1,545	833

(a) Venn results of deletion locus data were obtained based on TBtools and SyRI software using the female fish genome as a reference, and 1,791 out of 2,116 deletion loci obtained based on SyRI software were shared deletion variant loci. (b) Utilizing the male fish genome as a point of reference for acquiring deletion site data in the context of Venn analysis, it was found that 1,749 out of the 2,045 deletion sites identified through the use of the SyRI software were common deletion variant sites. (c) Depth distribution of CLR reads in male fish using all female genomic deletion locus (>100bp) information as a reference, with the area where high-quality male and female genetic marker information was located before the orange labeled trough position (~5.4). (d) The depth distribution of CLR reads in females was analyzed by utilizing male genomic deletion sites as a reference (>100bp), focusing on the region preceding the orange labeled trough position (~12.5) where high-quality male and female genetic marker information was present. (e) - (f) Depth distribution of CLR reads in males and females based on intersecting male and female genomic deletion site information (>100bp), respectively.

Table 3. Statistical cross-validation of multiple scanning of genome-wide large insertion deletion sequence marker sites.

Reference genome	Female vs. Male			Male vs. Female		
Mutation type	Insertion		Deletion	Insertion		Deletion
Variant length (>100 bp)	4,422		4,675	4,615		4,385
Chromosomes	FChr1	131	123	MChr1	140	143
	FChr2	194	211	MChr2	208	194
	FChr3	173	199	MChr3	179	161
	FChr4	181	207	MChr4	214	186
	FChr5	241	220	MChr5	212	237
	FChr6	254	292	MChr6	277	246
	FChr7	212	234	MChr7	231	211
	FChr8/10	333	341	MChr8	143	165
	FChr9	165	146	MChr9	345	333
	FChr11	191	217	MChr10	217	179
	FChr12	157	160	MChr11	159	153
	FChr13	205	187	MChr12	185	203
	FChr14	158	193	MChr13	196	162
	FChr15	182	185	MChr14	186	188
	FChr16	169	202	MChr15	190	160
	FChr17	204	220	MChr16	228	196
	FChr18	168	159	MChr17	159	167
	FChr19	175	183	MChr18	168	171
	FChr20	168	184	MChr19	174	166
	FChr21	185	203	MChr20	205	183
	FChr22	161	183	MChr21	184	167
	FChr23	200	219	MChr22	225	211
	FChr24	215	207	MChr23	190	203
SyRI-based scanning of male and			2,116			2,045

female mutant sites (≥100bp)				
Intersection of the two methods		1,791 (84.64%)		1,749 (85.53%)

Table 4. Statistics of transposons and tandem repeat sequences attributed to large deletion sequence tag sites obtained with male and female reference genomes

Reference genome	Mutation type	Transposon	Tandem Repeat	Total
Female vs. Male	Deletion	3,667 (78.44%)	1,574 (33.67%)	4,675
Male vs. Female	Deletion	3,380 (77.08%)	1,399 (31.90%)	4,385

Table 5. Statistics of large-segment differential loci after primer design, e-PCR amplification, and simulated amplification products and size screening.

Reference genome	Mutation type	Variant length (>100 bp)	High-throughput design of primers for variant sites	e-PCR of primers with single band	Single band and suitable amplification size (100~2000 bp)
Female vs. Male	Insertion	4,422	4,016	5,735	2,786
	Deletion	4,675	1,314	957	853
Male vs. Female	Insertion	4,615	4,131	3,236	2,876
	Deletion	4,385	1,199	882	796

High-throughput primer design and e-PCR bulk simulation for target regions

The identified large insertion/deletion variants were utilized as target regions for sex identification in both male and female genomes. High-throughput primers were designed to generate primer catalogs for the specified target markers. ~~We used the identified large insertion/deletion variants as target regions for male and female sex identification and designed high-throughput primers to construct high-throughput primer datasets for the target markers.~~ A total of 4,016 ~~5,044~~ and 1,314~~1,628~~ primer pairs were obtained by high-throughput primer design for the insertion/deletion target regions, respectively, using females genome as the reference (Table ~~2~~5). To further test the accuracy and uniqueness of the high-throughput primers across the genome, we performed genome-wide electronic simulated amplification of the obtained primer pairs

Formatted: Font: Not Bold

by using e-PCR. A total of 2,786 and 853 genome-wide single amplification band primer pairs were identified through screening of 5,735 primer pairs and 957 primer pairs with single bands, respectively, resulting in amplification products ranging from 100 to 2,000 base pairs~~A total of 2,791 and 854 genome-wide single amplification band primer pairs were obtained by screening from 5,044 primer pairs and 1,628 primer pairs, respectively, which yielded amplification products of 100 to 2,000 bp~~ (Table ~~2~~5, Supplementary Female_Male_INS/DEL100_PrimerSet.xlsx). Utilizing the *O. fasciatus* male genome as a point of reference, 4,131 and 1,199 primer pairs were obtained to target insertions/deletions of specific variant sites~~Using the *O. fasciatus* male genome as a reference, we obtained 5,505 and 1,545 primer pairs corresponding to insertions/deletions of the target variant sites~~, respectively (Table 2, Supplementary Female_Male_INS/DEL100_PrimerSet.xlsx). Notably, 2,876 and 796 primer pairs for whole-genome single amplification bands (100~2,000bp) were identified through screening of 3,236 primer pairs and 882 primer pairs with single band~~Among them, 3,039 and 833 primer pairs for whole-genome single amplification bands were obtained~~ (Table ~~2~~5, Supplementary Female_Male_INS/DEL100_PrimerSet.xlsx).

Formatted: Font: Italic

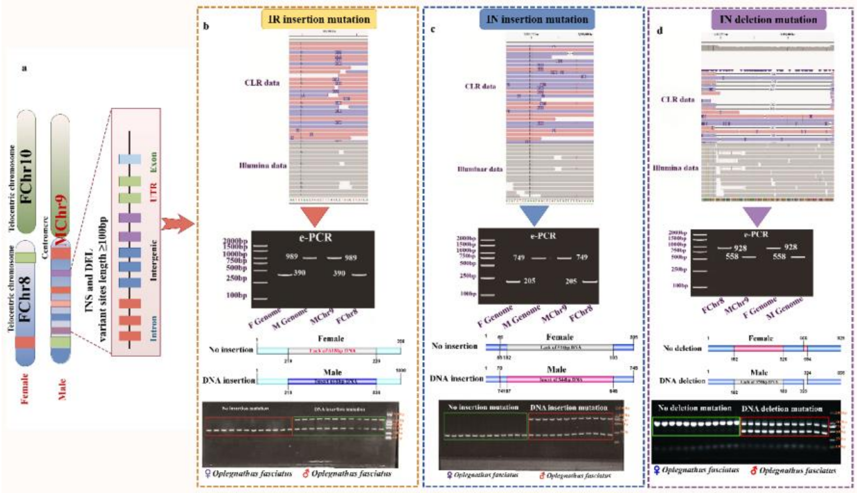

Figure 5: Representative genetic sex marker (insertion deletion variant sites) located in the intergenic region (IR), Intronic region (IN) of the *O. fasciatus* genome (STOSOd393e).

(a) Location of male and female sex markers in the male (MChr9) and female (FChr8) genomes. (b) Male and female sex genetic markers were located at loci in the intergenic region, using the female fish genome as a reference (Figure S2). Illumina and CLR clean data of bam comparison to the genome for insertion/deletion site validity detection. Red represented length long data from PacBio and gray represented was Illumina clean data. The e-PCR amplified bands were 390bp and 989bp in size, corresponding to DNA insertion and deletion patterns and agarose gel

electrophoresis for identification of male and female genetically different populations. (c) By utilizing the female genome as a point of comparison, the genetic markers for male and female sexes were identified at specific insertion sites within *nuf2* gene introns (Figure S3, Figure S4). The resulting e-PCR amplified bands, measuring 205bp and 749bp in size, were indicative of distinct DNA insertion patterns and were utilized in agarose gel electrophoresis for the purpose of distinguishing between male and female genetically differentiated groups. Males exhibited two amplified bands, while females exhibited only one. (d) By utilizing the female fish genome as a point of comparison, the genetic markers for male and female sexes were situated within the deletion sites located in the introns of genes (Figure S5, Figure S6). The amplified bands produced through e-PCR were observed to be 558bp and 928bp in length, aligning with the DNA deletion patterns and agarose gel electrophoresis maps utilized for distinguishing genetically distinct groups of males and females. Specifically, two bands were amplified in males, while only one band was observed in females.

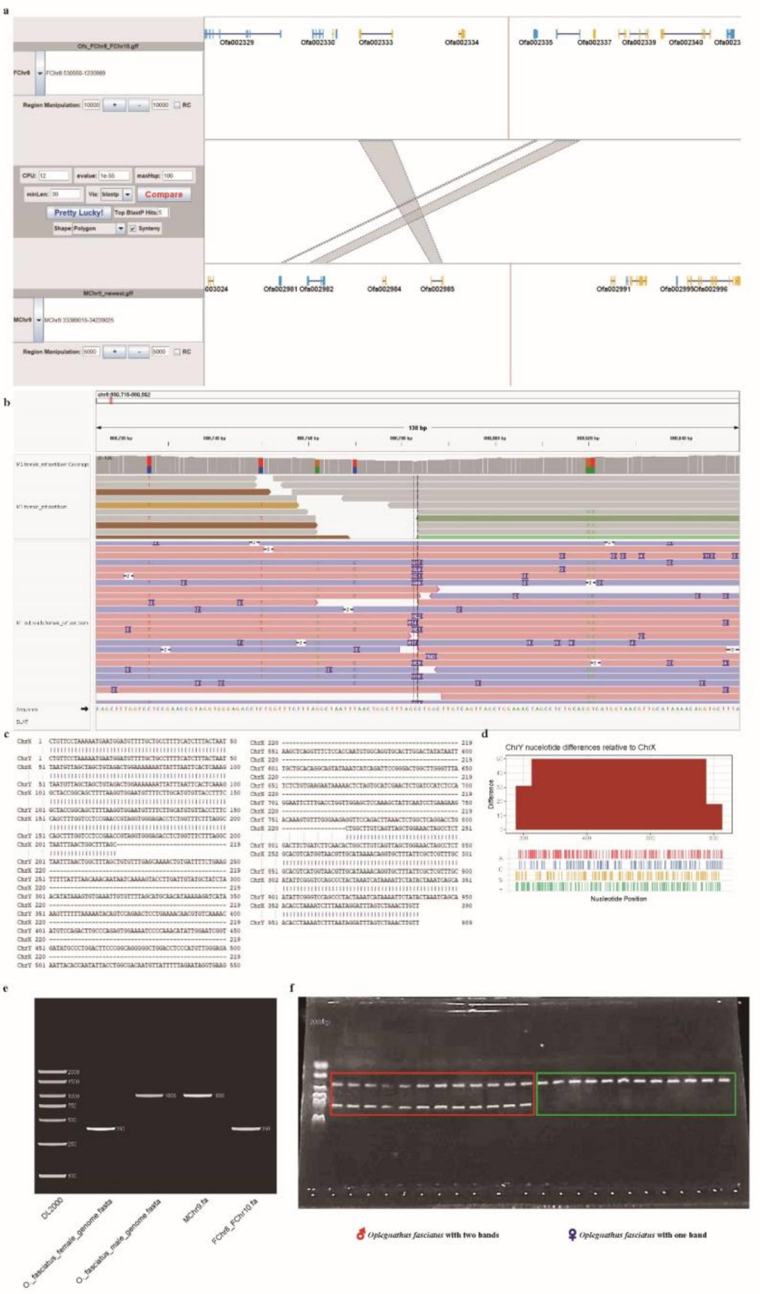

Figure 4: Representative genetic sex marker (insertion deletion variant sites) located in the intergenic region of the *O. fasciatus* genome.

(a) Location of male and female sex markers in the male and female genomes. (b) Illumina and CLR clean data of

~~Bam comparison to the genome for insertion/deletion site validity detection. (c) Nucleotide sequence comparison of markers for female and male genetic sex identification. (d) Visualization of regions of nucleotide sequence heterogeneity in markers for female and male genetic sex identification. (e) Electronically simulated amplification results of the target marker primer (e-PCR). (f) Results of PCR amplification and agarose gel electrophoresis detection of target markers (two bands for males and one band for females).~~

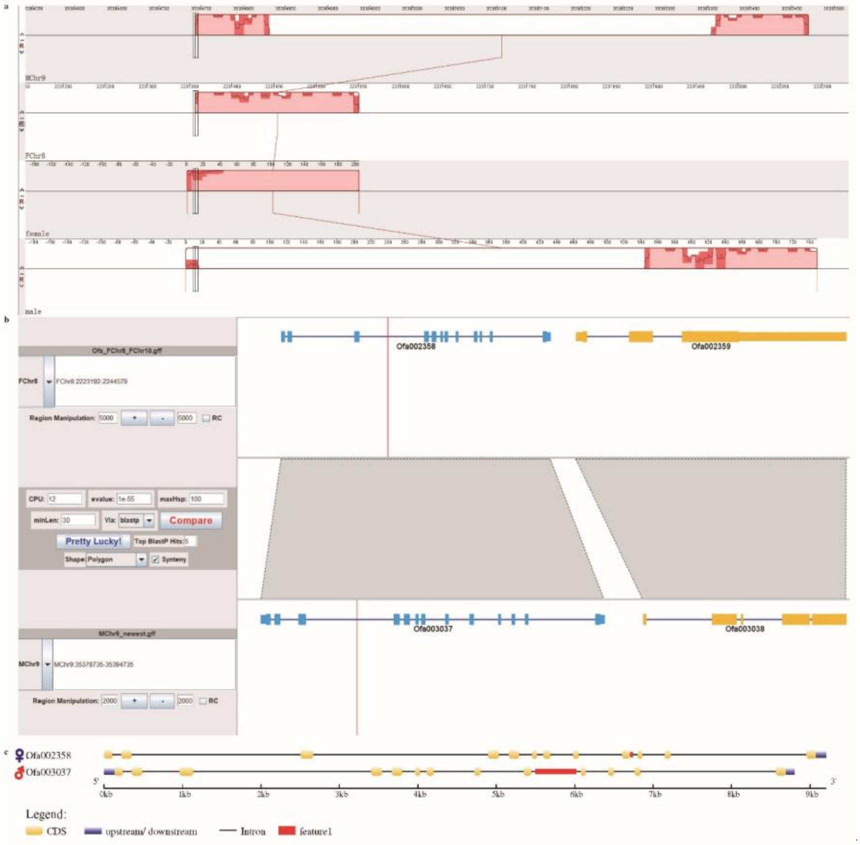

~~**Figure 5:** Representative genetic sex marker (insertion and deletion variant sites) located in the intron region of the *nuf2* gene in *O. fasciatus*.~~

~~(a) Collinearity relationships between male and female genetic sex markers and their association with the male and female genomes. (b) Location of male and female sex markers in the male and female genomes. (c) Unit composition of gene functional regions, occurrence of insertions and deletions of genetic sex markers, and sequence length patterns.~~

Validation of molecular markers for genetic sex identification of females and males

We selected markers located in the intergenic and intragenic regions for female and male genetic sex identification for validation testing. Using the female genome as a reference, a 599 bp DNA fragment~~base sequence~~ was inserted into the male genome at

position 880,783 bp on female chromosome 8 (after TTTAGC), corresponding to e-PCR amplicons of 390 bp and 1008 bp, respectively (Figure ~~4~~5, Figure ~~S1~~S2). Sequence comparisons of the genetic sex markers showed that the genetic differences between male and female markers were mainly concentrated in the region of 200~800 bp. This variant locus was further confirmed as a valid insertion site using female fish as a reference genome, synthesized using genomic survey data from short-read sequencing data survey~~Illumina sequencing~~ of male fish and long-read sequencing~~CLR~~ data from PacBio platform~~sequencing~~ for read assignment (Figure 4, Figure S1). The results of PCR amplification and agarose gel electrophoresis were consistent with the results of e-PCR corresponding to two bands (390 bp and 1,008 bp) for males and one band (390 bp) for females. According to the genetic sex identification marker localization, the marker lies between the *samd3* gene (sterile alpha motif.) (Ofa002334) and *elf3* gene (E74-like factor 3) (Ofa002335) in the female genome (Figure ~~4~~5, Figure S2).

The intragenic markers obtained from screening were also used to test the effectiveness of female and male genetic sex identification in *O. fasciatus*. In the region corresponding to the intron of the female *nuf2* gene (component of the NDC80 kinetochore complex) (Ofa002358) on chromosome 8 (2,231,443 bp, after GCAAATA), a 518 bp DNA fragment~~base sequence~~ was inserted into the intron of the male *nuf2* gene, corresponding to e-PCR amplified fragment sizes of 206 bp and 739 bp, respectively (Figure ~~5~~S3). With the female genome as a reference, we utilized the male short-read sequencing data and long-read PacBio sequencing~~Illumina survey data and PacBio CLR~~ data for read assignment and further confirmed that this candidate variant site was a valid insertion site (Figure ~~6~~S4). Comparison of the sequences for the genetic sex marker showed that the genetic differences between the male and female markers were mainly in the region of 90~650 bp. The results of PCR amplification and agarose gel electrophoresis further showed that the males exhibited two bands (206 bp and 739 bp), and the females had one band (206 bp), in agreement with the e-PCR results (Figure ~~6~~S4).

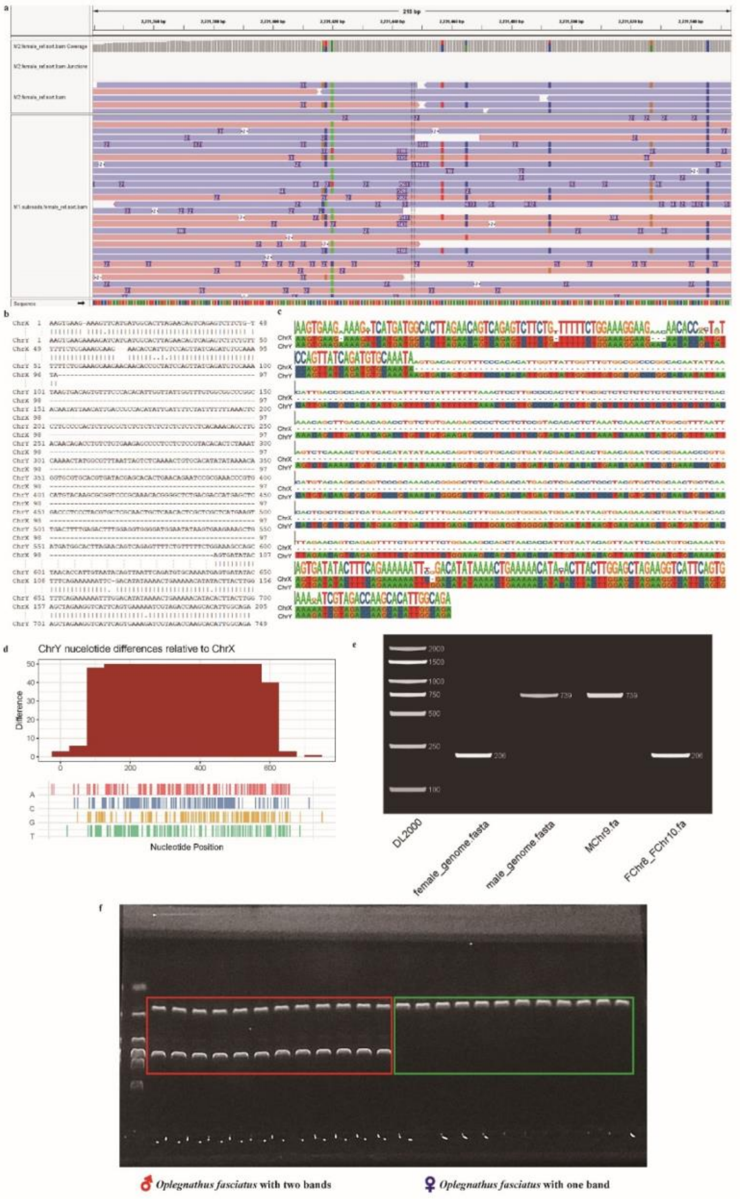

Figure 6: Validity testing of genetic markers located in the intronic region of the *nuf2* gene.

(a) Illumina and CLR clean data of Bam comparison to the genome for insertion/deletion site validity detection. (b) Nucleotide sequence comparison of markers for female and male genetic sex identification. (c) Comparison of genetic sex marker sequences based on the global alignment standard. (d) Visualization regions of nucleotide sequence heterogeneity in markers for female and male genetic sex identification. (e) Electronically simulated amplification results of the target marker primer (e-PCR). (f) Results of PCR amplification and agarose gel electrophoresis detection of target markers (two bands for males and one band for females).

In addition to the male insertion genetic sex markers, a male deletion genetic sex marker was also identified. In the region corresponding to female chromosome 8 from 1,161,186 bp (after GATGAGGAAAG) to 1,161,530 bp (before TATGAAGTCT), the males were missing a 343 bp DNA fragment~~base sequence~~, corresponding to e-PCR amplicon sizes of ~~557~~ 558 bp and 928 bp, respectively (Figure ~~7~~S5). A comparison of sequence differences, short-read sequencing data and long-read PacBio sequencing~~Illumina survey data and PacBio CLR~~ data read assignments all confirmed that the genetic differences between male and female markers were mainly concentrated in the ~~181~~182~~~550~~ 551 bp and ~~664~~665~~~692~~ 693 bp regions (Figure ~~8~~S6). PCR amplification and agarose gel electrophoresis assays showed two bands in males (~~557~~ 558 bp and 928 bp) and one band in females (928 bp), which was consistent with the results of e-PCR (Figure ~~8~~S6). A similar genetic sex marker for the deletion locus in males was also identified in the region corresponding to chromosome 8 of females at 921,537 bp (after AAATGTGGCGG), with deletion of a 299 bp DNA fragment~~base sequence~~ and insertion of a 119 bp DNA multi-fragments~~multifragmented base sequence~~ in males. PCR amplification and agarose gel electrophoresis tests showed two bands in males (748 bp and 926 bp) and one band in females (926 bp), consistent with the e-PCR results (Figure ~~S2~~S7, Figure ~~S3~~S8).

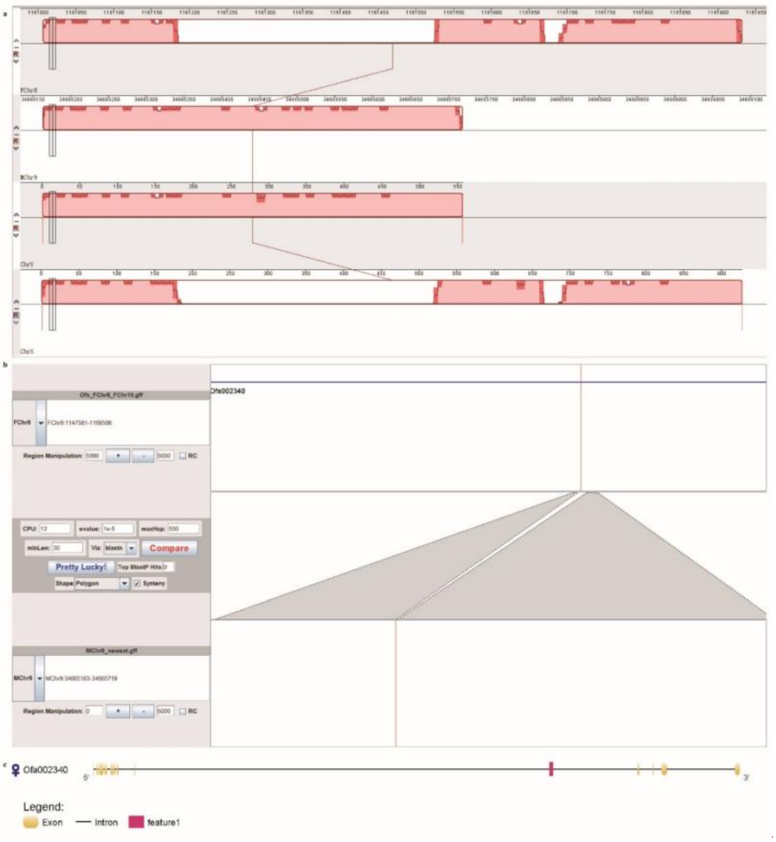

Figure 7: Representative male deletion genetic sex marker (insertion deletion variant sites).

(a) Collinearity relationships between male and female genetic sex markers and their association with the male and female genomes. (b) Location of male and female sex markers in the male and female genomes. (c) Unit composition of gene functional regions and occurrence of male deletion genetic sex markers.

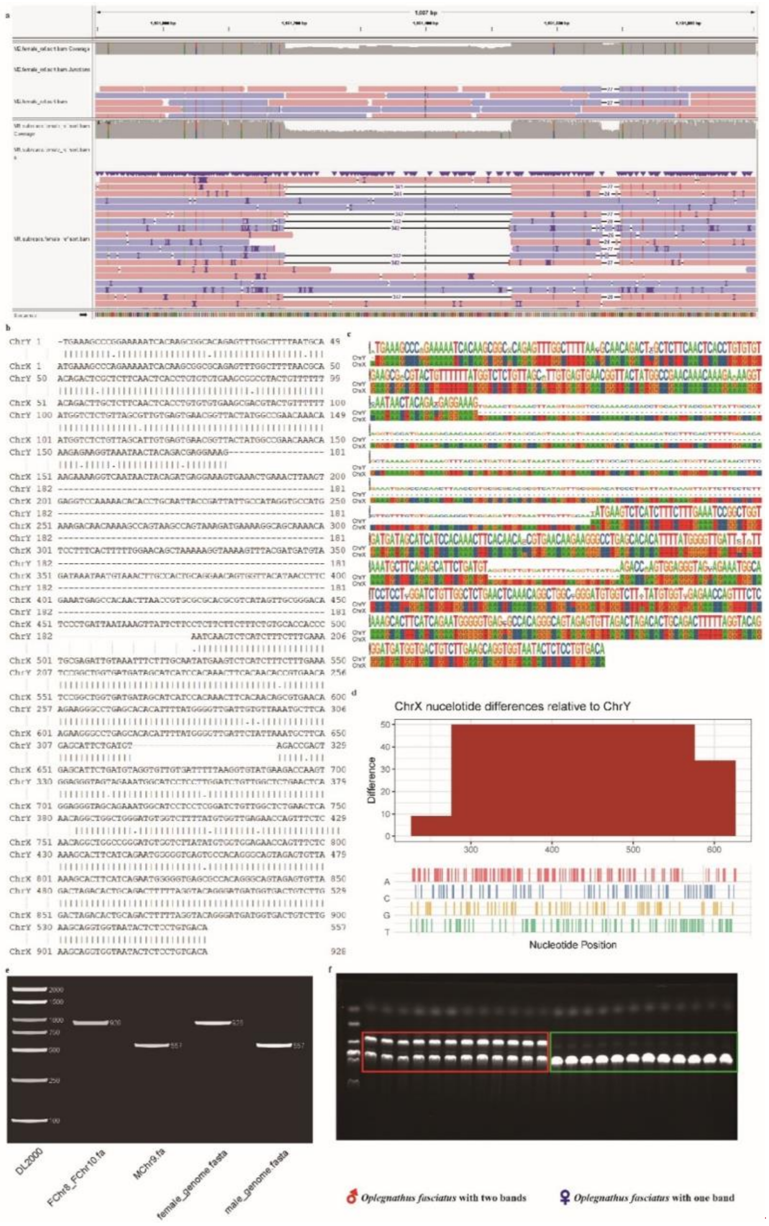

~~**Figure 8:** Validity testing of the male deletion genetic markers.~~

~~(a) Illumina and CLR clean data of Bam comparison to the genome for insertion/deletion site validity detection. (b) Nucleotide sequence comparison of markers for female and male genetic sex identification. (c) Comparison of genetic sex marker sequences based on the global alignment standard. (d) Visualization regions of nucleotide sequence heterogeneity in markers for female and male genetic sex identification. (e) Electronically simulated amplification results of the target marker primer (e-PCR). (f) Results of PCR amplification and agarose gel~~

electrophoresis detection of target markers (two bands for males and one band for females).

Enrichment analysis of functional genes for genetic sex markers

To further investigate the effect of large-segment insertion/deletion loci on gene function in female and male *O. fasciatus*, we initiated an enrichment analysis of functional genes for genetic sex markers. Using the female genome as a reference, 73 and 435 genes were affected by large-segment insertion/deletion sites located in the exonic region (Supplementary Female_reference_INS_DEL100_ExonSet_annotation.xlsx). The genes involved in insertion site markers were enriched in glycerophospholipid metabolism, blood circulation and other biological processes and involved in the molecular regulation of phosphatidylinositol deacylase activity, histone H3R2 demethylase activity, choline kinase activity, thioredoxin-disulfide reductase (NADP) activity, arylamine N-acetyltransferase activity, and calcium-dependent phospholipase A2 activity (Figure S4). Genes involved in the deletion site markers were enriched in biological processes such as regulation of morphogenesis of the epithelium, protein maturation, cell-matrix adhesion, cranial nerve formation, regulation of lysosome size, dendritic cell chemotaxis, and regulation of systemic arterial blood pressure by circulatory renin-angiotensin and participated in molecular regulation of glial cell-derived neurotrophic factor receptor binding, carbohydrate proton symporter activity, D-amino-acid oxidase activity, ATP-activated inwards rectifier potassium channel activity, ATP-dependent protein folding chaperone, sterol binding, and other molecular regulatory processes (Figure S4). In addition, KEGG results showed that the genes involved in the deletion site markers were enriched in regulatory pathways such as cholesterol metabolism and sphingolipid metabolism (Figure S4).

Using the male fish genome as a point of reference, it was observed that the exon region contained significant loci for large-segment insertions and deletions, encompassing 79 and 233 genes, respectively (Supplementary Male_reference_INS_DEL100_ExonSet_annotation.xlsx). Furthermore, in conjunction with the findings pertaining to the deletion sites in female fish, gene enrichment analysis revealed a notable association with the regulation of vascular development and the promotion of myelin formation **(Figure S5)**. Additionally, the insertion site marker genes exhibited enrichment in functions such as stem cell population maintenance, response to sucrose, UDP-N-acetylgalactosamine metabolic process, digestive system

~~development, negative regulation of the execution phase of apoptosis, positive regulation of actin nucleation, negative regulation of cyclin-dependent protein serine/threonine kinase activity, intermediate filament bundle assembly, resolution of meiotic recombination intermediates, and biological regulation of certain processes (Figure S5). In addition, the related genes were involved in the molecular regulation of hepatocyte growth factor receptor activity, N6-isopentenyladenosine methyltransferase activity, ketohexokinase activity, keratin filament binding, sterol 12-alpha-hydroxylase activity, carbohydrate proton symporter activity, ATP-activated inwards rectifier potassium channel activity, alpha-N-acetylneuraminate alpha-2,8-sialyltransferase activity, superoxide-generating NADPH oxidase activator activity, and laminin binding (Figure S5). KEGG results showed that the genes involved in the insertion site markers were enriched in regulatory pathways such as caffeine metabolism, glycerophospholipid metabolism, selenium compound metabolism, and glycosylphosphatidylinositol (GPI) anchor biosynthesis (Figure S5). Deletion site marker genes were enriched in the reregulation of structural morphogenesis, negative regulation of the GIS phase transition of the cell cycle, pancreatic regeneration, regulation of deoxyribonuclease activity, activation of basophils, localization of proteins in the T-tubules, germline stem cell division, mitotic/amnestic phase transition of the mitotic cell cycle, and other biological processes (Figure S6). In addition, these genes participate in molecular regulatory processes such as phosphatidylinositol deacylase activity, 5-tyrosyl DNA phosphodiesterase activity, fatty acid α-hydroxylase activity, ATP adenylyltransferase activity, mRNA N6-methyladenosine dioxygenase activity, N-acylneuraminic acid cytidylyltransferase activity, vascular endothelial growth factor receptor 3 binding, phosphatidylethanolamine transfer activity, neurotransmitter receptor activity involved in the regulation of postsynaptic cytosolic calcium concentration, cosynaptic receptor binding, and structural formation of chromatin. KEGG results showed that genes involved in the insertion-deletion site markers were enriched in the regulatory pathways of tight junctions, caffeine metabolism, ubiquitin-mediated protein hydrolysis, cell adhesion molecules, and the apelin signalling pathway (Figure S6).~~

Discussion

The majority of fish exhibited~~exhibit~~ limited sex chromosome differentiation, which posed~~poses~~ challenges in identifying them based on chromosome morphology [10].

Additionally, fish sex was~~is~~ influenced by environmental factors, particularly temperature, resulting in discrepancies between the physiological phenotype and genotype of sex [11]. Consequently, it was~~is~~ crucial within the field of aquaculture to discover a straightforward approach capable of rapidly determining the genetic composition of fish sex [61]. DNA molecular markers ~~are~~ were created by exploiting the prevalence of polymorphisms in the genomic DNA of eukaryotic organisms, which serve as indicators of individual variations at the DNA level [7]. A review of the literature revealed that over the past decade, approximately 48% of recent studies on male and female sex determination had predominantly employed molecular biology techniques such as RFLP, RAPD, SSR, and AFLP ~~Analysis of relevant literature reveals that over the past decade, approximately 48% of genetic sex markers were derived from first-generation techniques such as RFLP, RAPD, SSR, and AFLP~~ [61-72]. Meanwhile, with the advancement of second-generation high-throughput sequencing technologies, particularly Illumina, the utilization of sex markers based on SNP/InDel variants has increased to approximately 51% ~~Meanwhile, second-generation techniques such as Illumina's SNP/InDel identified approximately 51% of sex markers~~ [16, 73-78]. However, the advancement of sex-controlled breeding has faced limitations due to various factors, such as low throughput of the above methods, cumbersome experimental procedures, expensive development cost, high rate of false positives, unstable markers, and inconvenient on-site testing. With the emergence and rapid advancement of third-generation sequencing technology, exemplified by long-fragment read-length PacBio sequencing, the development of third-generation high-throughput sex markers based on insertions or deletions of large fragments (>100 bp) has become feasible [37, 45, 46]. In this study, *O. fasciatus* with the $X_1X_1X_2X_2/X_1X_2Y$ system served as the focal point [44-46, 48]. By utilizing the assembled genome~~linear genomic~~ information of male and female individuals acquired through PacBio sequencing, along with data from the short-read sequencing data and long-read PacBio sequencing data~~Illumina surveys and PacBio CLR/CCS~~, we extensively employed genome-wide variant sites multiple scanning and identification, STS long reads depth evaluation and ranking~~whole genome variant site scanning and identification~~, high-throughput design of primers for the target regions, and e-PCR batch amplification (Figure 1). By employing a comprehensive approach involving genome-wide variant site scanning, high-throughput primer design within the desired region, and e-PCR batch amplification and validation methodologies, we successfully exploited a

catalog~~developed a database~~ encompassing insertion/deletion loci in large segments (>100 bp) for both male and female *O. fasciatus* (Figure 2, Figure 3, Table ~~1~~2, Table ~~2~~3). Subsequently, a high-throughput identification catalog of sex-specific markers was established through this methodology, leading to the discovery of 3,639 (2,786 INS/853 DEL, ♀ as reference) and 3,672 (2,876 INS/833 DEL, ♂ as reference) markers, along with 1,021 and 894 high-quality genetic sex identification markers, respectively~~This effort yielded a total of 3,645 (2,791 insertions and 854 deletions) and 3,872 (3,039 insertions and 833 deletions) genetic markers in females and males, respectively~~, serving as a reliable reference for sex identification (Table 2, Table 3). Following agarose gel electrophoresis, two amplification bands for males and one amplification band for females were successfully identified, enabling rapid differentiation between the sexes in *O. fasciatus*. This advancement in sex marker development, utilizing third-generation high-throughput and precise techniques, will expedite the progress of genetic breeding and facilitate the establishment of genetic sex markers.

Variation in genome structure was intricately linked to human diseases, evolution, gene regulation, and species phenotypes in the biological realm, making it a prominent area of study in genomics research [79]. Previous studies have shown that DNA sequences with insertions and deletions undergo increased selective pressure ~~Prior research has demonstrated that DNA sequences containing insertions and deletions experience heightened selective pressure~~ [80-84]. The presence of deletions and insertions in DNA sequences, regardless of their location in noncoding or coding regions, inevitably influences the functionality of the original sequence to varying extents [79]~~Both deletions and insertions in DNA sequences, whether in noncoding or coding regions, inevitably impact the functionality of the original sequence to varying degrees~~. Furthermore, evidence suggested~~suggests~~ that the length and placement of insertions ~~are~~were not random but rather heavily influenced by the adjacent DNA sequence [80-82, 85, 86]. Prior research has indicated that insertion deletion variants accounted for 16%, 24%, 13%, 25%, and 15% of genomic genetic polymorphisms in various organisms, including *Drosophila melanogaster*, *Caenorhabditis elegans*, mouse, human, and *Arabidopsis thaliana* ~~In recent years, research has revealed that insertions and deletions contribute to 16.2% and 24.9% of the genetic polymorphisms in *Drosophila melanogaster* and *Caenorhabditis elegans*, respectively~~ [34-36, 87-94]~~(Dawson et al., 2001; Devine et al., 2006)~~. Recent advancements in long length sequencing technology have allowed for a more precise examination of human genetic

variation, revealing that insertions and deletions collectively contribute to a high portion (13.8%, 11.1%) of genetic polymorphisms in the human genome [90-94]. The focus of genomic structural variation studies has predominantly been on single nucleotide polymorphisms (SNPs) and insertions/deletions (InDels) of less than 50 base pairs due to the limitations of high-throughput sequencing technologies for short read lengths. However, larger genomic structural variants, which encompassed genetic variations not fully captured by SNPs and InDels, were closely linked to biological phenotypes and might significantly influence phenotypic traits, disease susceptibility, and adaptive capacity [79]. The utilization of long-read length sequencing technologies such as PacBio and ONT has enabled the generation of extended DNA sequences, thereby enhancing the assembly of diploid genomes of superior quality and the identification of structural variants (SVs) across substantial genomic regions [95]. Prvious research has documented seven deletions (>100bp) within approximately 700bp large segments of the *ZmBAM1d* gene, demonstrating a positive association with maize kernel weight. These deletions were consistently inherited in high-yielding maize lines [96]. In mammalian studies, a deletion of approximately 40Mb on chimpanzee chromosome 2, as compared to humans and gorillas within the great ape family, has been identified, leading to stable genetic distinctions among species [97]. Similar results have been documented in *Astyanax mexicanus*, *Gasterosteus aculeatus*, racehorses, and dogs [98-101]. The emergence of long read-length sequencing technology has propelled large fragment genomic structural variation to the forefront of genetic variation research, particularly in investigations of species' adaptive evolution [95, 96, 102, 103]. The findings of this study indicated~~indicate~~ that insertions and deletions identified using the female and male *O. fasciatus* genomes as references constituted 26.96% and 26.97%~~25.26% and 26.99%~~ of the genetic polymorphisms, respectively. Our research has contributed new insights into the prevalence of large-segment insertion-deletion events for *O. fasciatus*, indicating that these events predominantly occurred in the intergenic region in 58.09%~66.33% of instances, with the intronic region following closely at 31.65%~40.35% of cases~~Our study has provided additional insights into the occurrence of large-segment insertion-deletion events, revealing that these events occur in the intergenic region in 68% of cases, followed by the intronic region in 43% of cases~~ (Figure 2, Figure S1 Table 2~~Figure 3, Table 1~~). ~~Furthermore, w~~We also observed a higher proportion of deletion sites compared to insertion sites. A similar study conducted on the β-fibrinogen intron of

birds reported similar findings [104]. Furthermore, this study presented novel findings indicating that over 77% of the identified large deletion sequence tag sites in the genomes of both female and male *O. fasciatus* were attributed to transposons (Table 4). It was evident that transposons were prevalent in the animal genome and significantly contribute to its evolutionary processes. Previous research has demonstrated that a significant portion (69.5%) of genomic structural variation in large segments of tomato was situated within the transposon region, which was closely linked to desirable traits in tomato [105]. Comparable studies have also been conducted on gene regulation in human brain diseases and genomic structural variation in evolutionary distinct phenotypes of great apes [103, 106]. Transposons have the potential to drive genome and chromosome evolution through various mechanisms such as insertion-induced gene variation, gene retrotransposition, chromosome recombination, gene duplication, and serving as sites for ectopic recombination. These findings underscored the importance of transposons in facilitating adaptive evolution for *O. fasciatus*. The presence of large fragments of insertion deletion sequence marker sites was frequently influenced by natural selection pressures, with their formation being intricately linked to adjacent DNA sequences and commonly correlated with characteristic phenotypic traits and aberrant gene regulation [83, 84, 102, 103, 105, 106]. Specifically, the manifestation of albino symptoms in dogs resulting from a 965-kb deletion on chromosome 18 exhibited stable inheritance patterns, while a 4.8 Mb deletion variant on the chromosome of male domestic chickens similarly displayed heritability across subsequent generations [100, 107]. Consistent inheritance of variants within populations has also been documented in academic research on maize (*ZmBAM1d*), *Astyanax mexicanus*, *Ailuropoda melanoleuca* (*Bace2*), great apes (*acan*), and human diseases such as hypertension (*ace*) [96, 98, 103, 108]. The findings from the population genetic sex marker amplification analysis conducted in this study indicated that the insertion-deletion markers exhibited stability in both male and female populations of *O. fasciatus*. Additionally, analysis of gonadal histology and sex-validated marker data from various geographic populations demonstrated consistent inheritance of large segments of the insertion deletion markers in individuals of both sexes across different geographic regions (Figure S9). This observation provided additional evidence to support the theory that large-segment insertions and deletions are not a random occurrence but rather influenced by natural selection pressures [80, 82-84]. Based on the present findings, the stability of large segment insertion and deletion in sequence tag sites was evident. However, this should

not be taken to imply that all sequence tag sites exhibit this characteristic. A comprehensive evaluation of the evolutionary rate of sequence tags and the expansion of variant marker detection are necessary for ongoing research in this area.~~Notably, the genes located at these large insertion-deletion sites were predominantly associated with cholesterol metabolism, sphingolipid metabolism, and other glycerophospholipid metabolism processes. This observation indicates a potential direction for future investigations into the divergent functional traits between males and females.~~

Conclusions

These results highlighted the substantial prevalence of insertion and deletion events in the genetic makeup derived from the $X_1X_1X_2X_2/X_1X_2Y$ sex determination system of *O. fasciatus*. In our study, we successfully introduced a novel approach for the development of genetic sex markers in *O. fasciatus*. This approach involved the utilization of a primer batch electronic design technique, which utilized the insertion and deletion loci as the target regions. Additionally, we implemented an electronic batch amplification technique, allowing for the efficient and rapid high-throughput identification and screening of a substantial number of variant sites. This methodology represents a significant advancement in the field of *O. fasciatus* genetic sex marker development. The study yielded a greater number of genetic sex markers with higher amplification efficiency compared to those generated by previous technologies spanning the first or second generation. This methodology was applicable not only to the efficient generation of genetic sex markers for the $X_1X_1X_2X_2/X_1X_2Y$ sex-determination system but also to the determination of genetic sex in various sex-determination types (including autosomal and sex chromosome differences). It ~~is~~ was particularly well suited for sex-determination systems featuring heteromorphic chromosomes, such as the XY-type, ZW-type, XX/XY_1Y_2-type, ZZ/ZW_1W_2-type, and other species with different sex-determination mechanisms.

Ethics Statement

This research was approved by the Animal Care and Use committee of the Chinese Academy of Science.

Competing Interests

The authors declare no competing interests.

Data Availability

Formatted: Font: 10.5 pt

All additional supporting data are available in the GigaScience repository, GigaDB [109].

Availability of Supporting Source Code and Requirements

The code used for statistics on insertions and deletions as well as annotation scripts and base variant region visualization scripts are stored in jihuGitLab (https://jihulab.com/gigascience/sex-markers) with Username: xiaoyongshuang19@163.com and Login Password: Wwyxys1982The code used for statistics on insertions and deletions as well as annotation scripts and base variant region visualization scripts are publicly available in an online repository [109].

Abbreviations

CLR: Continuous Long Reads; CCS: circular consensus sequence; GO: Gene ontology; KEGG: Kyoto Encyclopaedia of Genes and Genomes.

References

1. Hughes JF, Skaletsky H, Pyntikova T et al. Chimpanzee and human Y chromosomes are remarkably divergent in structure and gene content, Nature 2010;463:536-539.
2. Bitencourt JA, Sampaio I, Ramos RT et al. First Report of Sex Chromosomes in Achiridae (Teleostei: Pleuronectiformes) with Inferences About the Origin of the Multiple X(1)X(1)X(2)X(2)/X(1)X(2)Y System and Dispersal of Ribosomal Genes in *Achirus achirus*, Zebrafish 2017;14:90-95.
3. Chen S, Zhang G, Shao C et al. Whole-genome sequence of a flatfish provides insights into ZW sex chromosome evolution and adaptation to a benthic lifestyle, Nat Genet 2014;46:253-260.
4. Lu G, Luo M. Genomes of major fishes in world fisheries and aquaculture: Status, application and perspective, Aquaculture and Fisheries 2020;5:163-173.
5. Chen SL, Ji XS, Shao CW et al. Induction of mitogynogenetic diploids and identification of WW super-female using sex-specific SSR markers in half-smooth tongue sole (*Cynoglossus semilaevis*), Mar Biotechnol (NY) 2012;14:120-128.
6. Pan ZJ, Li XY, Zhou FJ et al. Identification of Sex-Specific Markers Reveals Male Heterogametic Sex Determination in Pseudobagrus ussuriensis, Mar Biotechnol (NY) 2015;17:441-451.
7. Ma Y, Xiao Y, Xiao Z et al. Identification of Male-Specific Molecular Marker and Development of PCR-Based Genetic Sex Identification Technique in Spotted Knifejaw (*Oplegnathus punctatus*), Mar Biotechnol (NY) 2022;24:969-978.
8. Wu Y, Xiao Y, Xiao Z et al. Identification of Male-Specific Molecular Markers by Recombination of RhoGEF10 Gene in Spotted Knifejaw (*Oplegnathus punctatus*), Genes (Basel) 2022;13.
9. Li XY, Gui JF. Diverse and variable sex determination mechanisms in vertebrates, Sci China Life Sci 2018;61:1503-1514.
10. Chen J, Zhu Z, Hu W. Progress in research on fish sex determining genes, Water Biology and Security 2022;1.
11. Shen Z-G, Wang H-P. Molecular players involved in temperature-dependent sex determination and sex differentiation in Teleost fish, Genetics Selection Evolution 2014;46:26.
12. Dan C, Mei J, Wang D et al. Genetic differentiation and efficient sex-specific marker development of a pair of Y- and X-linked markers in yellow catfish, Int J Biol Sci 2013;9:1043-1049.

Formatted: Font: 10.5 pt

Formatted: Font: 10.5 pt

Field Code Changed

Field Code Changed

Formatted: Font: 10.5 pt

Formatted: Font: Italic

Formatted: Font: Italic

Formatted: Font: Italic

Formatted: Font: Italic

13. Liao X, Xu G, Chen SL. Molecular method for sex identification of half-smooth *tongue sole* (Cynoglossus semilaevis) using a novel sex-linked microsatellite marker, Int J Mol Sci 2014;15:12952-12958.

14. Ning Y, Liu X, Wang ZY et al. A genetic map of large yellow croaker *Pseudosciaena crocea*, Aquaculture 2007;264:16-26.

15. Xiao S, Wang P, Zhang Y et al. Gene map of large yellow croaker (*Larimichthys crocea*) provides insights into teleost genome evolution and conserved regions associated with growth, Sci Rep 2015;5:18661.

16. Lin A, Xiao S, Xu S et al. Identification of a male-specific DNA marker in the large yellow croaker (*Larimichthys crocea*), Aquaculture 2017;480:116-122.

17. Tao W, Zhu X, Cao J et al. Screening and characterization of sex-linked DNA markers in Mozambique tilapia (*Oreochromis mossambicus*), Aquaculture 2022;557.

18. Aparicio S, Chapman J, Stupka E et al. Whole-genome shotgun assembly and analysis of the genome of *Fugu rubripes*, Science 2002;297:1301-1310.

19. Mitani H, Kamei Y, Fukamachi S et al. The medaka genome: why we need multiple fish models in vertebrate functional genomics, Genome Dyn 2006;2:165-182.

20. Jones FC, Grabherr MG, Chan YF et al. The genomic basis of adaptive evolution in threespine sticklebacks, Nature 2012;484:55-61.

21. Davidson WS, Koop BF, Jones SJ et al. Sequencing the genome of the Atlantic salmon (*Salmo salar*), Genome Biol 2010;11:403.

22. Gong G, Dan C, Xiao S et al. Chromosomal-level assembly of yellow catfish genome using third-generation DNA sequencing and Hi-C analysis, GigaScience 2018;7:giy120.

23. Luo H, Zhang Y, Liu F et al. The male and female genomes of golden pompano (*Trachinotus ovatus*) provide insights into the sex chromosome evolution and rapid growth, Journal of Advanced Research 2023.

24. McGaugh SE, Gross JB, Aken B et al. The cavefish genome reveals candidate genes for eye loss, Nature Communications 2014;5:5307.

25. Pan H, Yu H, Ravi V et al. The genome of the largest bony fish, ocean sunfish (*Mola mola*), provides insights into its fast growth rate, GigaScience 2016;5:s13742-13016-10144-13743.

26. Schartl M, Walter RB, Shen Y et al. The genome of the platyfish, *Xiphophorus maculatus*, provides insights into evolutionary adaptation and several complex traits, Nature genetics 2013;45:567-572.

27. Valenzano DR, Benayoun BA, Singh PP et al. The African turquoise killifish genome provides insights into evolution and genetic architecture of lifespan, Cell 2015;163:1539-1554.

28. Xiao Y, Liu J, Wei J et al. Improved high-quality reference genome of red drum facilitates the processes of resistance-related gene exploration, Scientific Data 2023;10:774.

29. Xu M-R-X, Liao Z-Y, Brock JR et al. Maternal dominance contributes to subgenome differentiation in allopolyploid fishes, Nature Communications 2023;14:8357.

30. Zhang W, Yang Y, Hua S et al. Chromosome-level genome assembly and annotation of the yellow grouper, *Epinephelus awoara*, Scientific Data 2024;11:151.

31. Shi L, Guo Y, Dong C et al. Long-read sequencing and de novo assembly of a Chinese genome, Nature Communications 2016;7:12065.

32. VanBuren R, Bryant D, Edger PP et al. Single-molecule sequencing of the desiccation-tolerant grass *Oropetium thomaeum,* Nature 2015;527:508-511.

33. Vij S, Kuhl H, Kuznetsova IS et al. Chromosomal-Level Assembly of the Asian Seabass Genome

Using Long Sequence Reads and Multi-layered Scaffolding, PLoS Genet 2016;12:e1005954.

34. Aganezov S, Yan SM, Soto DC et al. A complete reference genome improves analysis of human genetic variation, Science 2022;376:eabl3533.

35. Nurk S, Koren S, Rhie A et al. The complete sequence of a human genome, Science 2022;376:44-53.

36. Schneider VA, Graves-Lindsay T, Howe K et al. Evaluation of GRCh38 and de novo haploid genome assemblies demonstrates the enduring quality of the reference assembly, Genome research 2017;27:849-864.

37. Burton JN, Adey A, Patwardhan RP et al. Chromosome-scale scaffolding of de novo genome assemblies based on chromatin interactions, Nat Biotechnol 2013;31:1119-1125.

38. Xiao Y, Liu J, Wei J et al. Improved high-quality reference genome of red drum facilitates the processes of resistance-related gene exploration, Sci Data 2023;10:774.

39. Schembri PJ, Bodilis P, Evans J et al. Occurrence of barred knifejaw, *Oplegnathus fasciatus* (Actinopterygii: Perciformes: Oplegnathidae), in Malta (Central Mediterranean) with a discussion on possible modes of entry 2010;40:101-104.

40. Mundy BCJBMBiZ. Checklist of the fishes of the Hawaiian Archipelago 2005;6:1-704.

41. An HS, Kim MJ, Hong SWJG et al. Genetic diversity of rock bream *Oplegnathus fasciatus* in Southern Korea, Genes and genomics 2008;30:451-459.

42. Xiao Y, Li J, Ren G et al. Pronounced population genetic differentiation in the rock bream *Oplegnathus fasciatus* inferred from mitochondrial DNA sequences 2016;27:2045-2052.

43. Xu D, Lou B, Bertollo LAC et al. Chromosomal mapping of microsatellite repeats in the rock bream fish *Oplegnathus fasciatus*, with emphasis of their distribution in the neo-Y chromosome 2013;6:1-6.

44. Xue R, An H, Liu Q et al. Karyotype and Ag-NORs in male and female of *Oplegnathus punctatus* 2016;47:626-632.

45. Xiao Y, Xiao Z, Ma D et al. Genome sequence of the barred knifejaw *Oplegnathus fasciatus* (Temminck & Schlegel, 1844): The first chromosome-level draft genome in the family Oplegnathidae 2019;8:giz013.

46. Xiao Y, Xiao Z, Ma D et al. Chromosome-Level Genome Reveals the Origin of Neo-Y Chromosome in the Male Barred Knifejaw *Oplegnathus fasciatus*, iScience 2020;23:101039.

47. Xu T, Shao C, Liao X et al. Isolation and characterization of polymorphic microsatellite DNA markers in the rock bream (*Oplegnathus fasciatus*) 2009;10:527-529.

48. Xu D, Lou B, Xu H et al. Isolation and characterization of male-specific DNA markers in the rock bream *Oplegnathus fasciatus* 2013;15:221-229.

49. Gong J, Zhao J, Ke Q et al. First genomic prediction and genome-wide association for complex growth-related traits in Rock Bream (*Oplegnathus fasciatus*) 2022;15:523-536.

50. Yong SX. NCBI Sequence Read Archive https://identifiers.org/ncbi/insdc.sra:SRP220007. 2020.

51. Xiao Y, Xiao Z, Ma D et al. Supporting data for "Genome sequence of the barred knifejaw *Oplegnathus fasciatus* (Temminck & Schlegel, 1844): the first chromosome-level draft genome in the family Oplegnathidae", GigaScience Database 2019:http://dx.doi.org/10.5524/100556.

52. Chen C, Wu Y, Xia R. A painless way to customize Circos plot: From data preparation to visualization using TBtools, iMeta 2022;1.

53. Chen C, Chen H, Zhang Y et al. TBtools: An Integrative Toolkit Developed for Interactive Analyses of Big Biological Data, Mol Plant 2020;13:1194-1202.

54. Li H. Minimap2: pairwise alignment for nucleotide sequences, J Bioinformatics 2018;34:3094-

3100.

55. Li H, Durbin RJb. Fast and accurate short read alignment with Burrows–Wheeler transform 2009;25:1754-1760.

56. Krzywinski M, Schein J, Birol I et al. Circos: an information aesthetic for comparative genomics 2009;19:1639-1645.

57. Kearse M, Moir R, Wilson A et al. Geneious Basic: an integrated and extendable desktop software platform for the organization and analysis of sequence data 2012;28:1647-1649.

58. Darling AE, Mau B, Perna NTJPo. progressiveMauve: multiple genome alignment with gene gain, loss and rearrangement 2010;5:e11147.

59. Zhou L, Feng T, Xu S et al. ggmsa: A visual exploration tool for multiple sequence alignment and associated data 2022;23:bbac222.

60. Hu B, Jin J, Guo A-Y et al. GSDS 2.0: an upgraded gene feature visualization server 2015;31:1296-1297.

61. Mei J, Gui JF. Genetic basis and biotechnological manipulation of sexual dimorphism and sex determination in fish, Sci. China Life Sci. 2014;44:5.

62. Agawa Y, Saiki T, Miyamoto T et al. Identification of the male sex-linked DNA sequence of cultured white trevally *Pseudocaranx dentex* 2022;88:319-327.

63. Goyat S, Grewal A, Singh D et al. Sex-linked AFLP marker identification in *dioecious Betelvine* (Piper betle L.) 2019;94:422-427.

64. Hashem MHJDJoVS. Early sexing of Tilapia nilotica (*Oreochromis niloticus*) by using short sequence repeats (SSRs) molecular markers 2022;8:22-25.

65. Hua J, Qiang J, Tao Y et al. Development and validation of a PCR-RFLP/TaqMan MGB probe method for rapid sex identification of largemouth bass (*Micropterus salmoides*) 2023;30:101593.

66. Jia X, Lu J, Tang X et al. A new method for molecular sex identification in the emu (Dromaius novaehollandiae) 2023:1-6.

67. Lajan S, Al-Barzinji YJIJoAS. Detection of quantitative loci correlation with growth traites in local quail using PCR-RFLP technique 2022;53:16-26.

68. Law D, Ping VC, Yee TC et al. Use of amplified fragment length polymorphism and sequence characterized amplified region marker for identifying the sex of the *Oxyeleotris marmorata* 2021.

69. Mohamed G, Youssef M, Mohamed E et al. Identification of Sex-specific Molecular Markers in Barbel and Nile Carp using SCoT and ISSR Markers 2021;52:60-73.

70. Panagiotopoulou H, Marzecki K, Gawor J et al. Extensive search of genetic sex markers in Siberian (*Acipenser baerii*) and Atlantic (*A. oxyrinchus*) sturgeons 2023;573:739517.

71. Vaux F, Aycock HM, Bohn S et al. Sex identification PCR–RFLP assay tested in eight species of Sebastes rockfish, Conservation Genetics Resources 2020;12:541-544.

72. Wen M, Zhang Y, Wang S et al. Characterization of sex locus and sex-specific sequences in the mandarin fishes 2022;561:738650.

73. Zheng S, Wang X, Zhang S et al. Screening and characterization of sex-linked DNA markers and marker-assisted selection in the Southern catfish (*Silurus meridionalis*), Aquaculture 2020;517:734783.

74. Liu H, Pang M, Yu X et al. Sex-specific markers developed by next-generation sequencing confirmed an XX/XY sex determination system in bighead carp (*Hypophthalmichehys nobilis*) and silver carp (Hypophthalmichthys molitrix), DNA Res 2018;25:257-264.

75. Han C, Zhu Q, Lu H et al. Screening and characterization of sex-specific markers developed by a simple NGS method in mandarin fish (*Siniperca chuatsi*), Aquaculture 2020;527:735495.

76. Palaiokostas C, Bekaert M, Khan MG et al. Mapping and validation of the major sex-determining region in Nile tilapia (*Oreochromis niloticus* L.) using RAD sequencing 2013;8:e68389.

77. Wang L, Xie N, Shen Y et al. Constructing high-density genetic maps and developing sexing markers in northern snakehead (*Channa argus*) 2019;21:348-358.

78. Wu X, Zhao L, Fan Z et al. Screening and characterization of sex-linked DNA markers and marker-assisted selection in blue tilapia (*Oreochromis aureus*), Aquaculture 2021;530:735934.

79. Sedlazeck FJ, Rescheneder P, Smolka M et al. Accurate detection of complex structural variations using single molecule sequencing 2017.

80. Chen Y, Wang AY, Barkley CA et al. Deciphering the exact breakpoints of structural variations using long sequencing reads with DeBreak, Nature Communications 2023;14:283.

81. Maki H. Origins of spontaneous mutations: specificity and directionality of base-substitution, frameshift, and sequence-substitution mutageneses, Annual review of genetics 2002;36:279-303.

82. Tian D, Wang Q, Zhang P et al. Single-nucleotide mutation rate increases close to insertions/deletions in eukaryotes, Nature 2008;455:105-108.

83. Cooper DN, Krawczak M. Mechanisms of insertional mutagenesis in human genes causing genetic disease, Human genetics 1991;87:409-415.

84. Krawczak M, Cooper DN. Gene deletions causing human genetic disease: mechanisms of mutagenesis and the role of the local DNA sequence environment, Human genetics 1991;86:425-441.

85. DN CJHG. Mechanisms of insertional mutagenesis in human genes causing genetic disease 1991;87:409-415.

86. Krawczak M, Cooper DNJHg. Gene deletions causing human genetic disease: mechanisms of mutagenesis and the role of the local DNA sequence environment 1991;86:425-441.

87. Berger J, Suzuki T, Senti K-A et al. Genetic mapping with SNP markers in Drosophila 2001;29:475-481.

88. Wicks SR, Yeh RT, Gish WR et al. Rapid gene mapping in *Caenorhabditis elegans* using a high density polymorphism map 2001;28:160-164.

89. Cao J, Schneeberger K, Ossowski S et al. Whole-genome sequencing of multiple *Arabidopsis thaliana* populations, Nature genetics 2011;43:956-963.

90. Porubsky D, Eichler EE. A 25-year odyssey of genomic technology advances and structural variant discovery, Cell 2024.

91. Ebert P, Audano PA, Zhu Q et al. Haplotype-resolved diverse human genomes and integrated analysis of structural variation, Science 2021;372:eabf7117.

92. Liao W-W, Asri M, Ebler J et al. A draft human pangenome reference, Nature 2023;617:312-324.

93. Porubsky D, Harvey WT, Rozanski AN et al. Inversion polymorphism in a complete human genome assembly, Genome Biol 2023;24:100.

94. Porubsky D, Höps W, Ashraf H et al. Recurrent inversion polymorphisms in humans associate with genetic instability and genomic disorders, Cell 2022;185:1986-2005. e1926.

95. Liu YH, Luo C, Golding SG et al. Tradeoffs in alignment and assembly-based methods for structural variant detection with long-read sequencing data, Nature Communications 2024;15:2447.

96. Yang N, Liu J, Gao Q et al. Genome assembly of a tropical maize inbred line provides insights into structural variation and crop improvement, Nature genetics 2019;51:1052-1059.

97. Goto H, Peng L, Makova KD. Evolution of X-degenerate Y chromosome genes in greater apes: conservation of gene content in human and gorilla, but not chimpanzee, Journal of molecular evolution 2009;68:134-144.

Formatted: Font: Italic

Formatted: Font: Italic

Formatted: Font: Italic

Formatted: Font: Italic

Formatted: Font: Italic

98. Gross JB, Wilkens H. Albinism in phylogenetically and geographically distinct populations of *Astyanax cavefish* arises through the same loss-of-function Oca2 allele, Heredity 2013;111:122-130.
99. Jones FC, Grabherr MG, Chan YF et al. The genomic basis of adaptive evolution in threespine sticklebacks, Nature 2012;484:55-61.
100. Schmutz S, Berryere T. Genes affecting coat colour and pattern in domestic dogs: a review, Animal genetics 2007;38:539-549.
101. Tozaki T, Ohnuma A, Kikuchi M et al. Short insertion and deletion discoveries via whole-genome sequencing of 101 Thoroughbred racehorses, Genes 2023;14:638.
102. Sudmant PH, Rausch T, Gardner EJ et al. An integrated map of structural variation in 2,504 human genomes, Nature 2015;526:75-81.
103. Zhou B, He Y, Chen Y et al. Comparative Genomic Analysis Identifies Great–Ape–Specific Structural Variants and Their Evolutionary Relevance, Molecular Biology and Evolution 2023;40:msad184.
104. Prychitko TM, Moore WSJMB, Evolution. Alignment and phylogenetic analysis of β-fibrinogen intron 7 sequences among avian orders reveal conserved regions within the intron 2003;20:762-771.
105. Alonge M, Wang X, Benoit M et al. Major impacts of widespread structural variation on gene expression and crop improvement in tomato, Cell 2020;182:145-161. e123.
106. Vialle RA, de Paiva Lopes K, Bennett DA et al. Integrating whole-genome sequencing with multi-omic data reveals the impact of structural variants on gene regulation in the human brain, Nature neuroscience 2022;25:504-514.
107. Fulton Lucinda A. MER, Wilson Richard K. Sequence and comparative analysis of the chicken genome provide unique perspectives on vertebrate evolution, Nature 2004;432:695-716.
108. Razaq A, Khan A, Shah ST et al. Association of insertion/deletion polymorphism of ace gene with essential hypertension in patients of Khyber Pakhtunkhwa, Pakistan Journal of Medical Sciences 2024;40:461.
109. Xiao Y, Xiao Z, Ma Y et al. Datasets and markers development workflow run scripts for high-throughput exploitation of genetic sex markers, figshare 2023.

Formatted: Font: Italic

Manuscript with clean version
Click here to access/download;Manuscript;MS clean version.docx

Innovative approach for high-throughput exploiting sex-specific markers in japanese parrotfish *Oplegnathus fasciatus*

Yongshuang Xiao[1,2,3,4†], Zhizhong Xiao[1,2,3,4†], Yuting Ma[1,2,3,4†], Haixia Zhao[1,2,3,4†], Yanduo Wu[1,2,3,4†], Jinwei Huang[1,2,3,4], Pingrui Xu[1,2,3,4], Lin Liu[5*], Jing Liu[1*], Jun Li[1,2,3,4*]

[1]Center for Ocean Mega-Science, Institute of Oceanology, Chinese Academy of Sciences, Qingdao, China， [2]CAS and Shandong Province Key Laboratory of Experimental Marine Biology, Institute of Oceanology, Chinese Academy of Sciences, Qingdao, China， [3]Key Laboratory of Breeding Biotechnology and Sustainable Aquaculture, Chinese Academy of Sciences, Qingdao, China， [4]Laboratory for Marine Biology and Biotechnology, Qingdao National Laboratory for Marine Science and Technology, Qingdao, China, [5]Wuhan Frasergen Bioinformatics Co., Ltd. East Lake High-Tech Zone, Wuhan, China.

† Co-first authors

*Correspondence address: Lin Liu, Wuhan Frasergen Bioinformatics Co., Ltd. East Lake High-Tech Zone, Wuhan, 430075, China; Tel: +86-053282896729; E-mail: liulin199306@163.com; Jing Liu, Institute of Oceanology, Chinese Academy of Sciences, 7 Nanhai Road, Qingdao, 266071, China; Tel: +86-053282898790; E-mail: jliu@qdio.ac.cn; Jun Li, Institute of Oceanology, Chinese Academy of Sciences, 7 Nanhai Road, Qingdao, 266071, China; Tel: +86-053282898718; E-mail: junli@qdio.ac.cn.

†Contributed equally to this work.

Yongshuang Xiao, ORCID: 0000-0002-1979-4555

Zhizhong Xiao, ORCID: 0000-0003-2403-1381

Yuting Ma, ORCID: 0000-0002-7158-9364

Haixia Zhao, ORCID: 0009-0003-8305-6009

Yanduo Wu, ORCID: 0000-0003-0060-1685

Jinwei Huang, ORCID: 0009-0003-5238-8318

Lin Liu, ORCID: 0009-0001-7315-2529

Jing Liu, ORCID: 0000-0001-8868-0386

Jun Li, ORCID: 0009-0005-2415-1895

Abstract

Background

The use of sex-specific molecular markers has become a prominent method in enhancing fish production and economic value, as well as providing a foundation for understanding the complex molecular mechanisms involved in fish sex determination. Over the past decades, research on male and female sex identification has predominantly employed molecular biology methodologies such as RFLP (Restriction Fragment Length Polymorphism), RAPD (Random Amplification of Polymorphic DNA), SSR (Simple Sequence Repeat), and AFLP (Amplified Fragment Length Polymorphism). The emergence of high-throughput sequencing technologies, particularly Illumina, has led to the utilization of SNP and InDel variants as significant molecular markers for investigating sex identification in fish. The advancement of sex-controlled breeding encounters numerous challenges, including the inefficiency of current methods, intricate experimental protocols, high costs of development, elevated rates of false positives, marker instability, and cumbersome field testing procedures. Nevertheless, the emergence and swift progress of PacBio high-throughput sequencing technology, characterized by its long read output capabilities, offers novel opportunities to overcome these obstacles.

Findings

Utilizing male-female assembled genome information in conjunction with short-read sequencing data survey and long-read PacBio sequencing data, a catalog of large-segment (>100 bp) insertion-deletion genetic variants was generated through a genome-wide variant site scanning approach with bidirectional comparisons. The sequence tagging sites (STS) were ranked based on the long-read depth of the insertion deletion site, with markers exhibiting lower long read depth being considered more effective for large segment deletion variants. Subsequently, a catalog of bulk primers and simulated PCR for the male-female variant loci was developed, incorporating primer design for the target region and e-PCR technology. The Japanese parrotfish (*Oplegnathus fasciatus*), belonging to the Oplegnathidae family within the Centrarchiformes order, holds significant economic value as a rocky reef fish indigenous to East Asia. The criteria for rapid identification of male and female differences in *O. fasciatus* were established through agarose gel electrophoresis, which revealed two amplified bands

for males and one amplified band for females. A high-throughput identification catalog of sex-specific markers was then constructed using this method, resulting in the identification of 3,639 (2,786 INS/853 DEL, ♀ as reference) and 3,672 (2,876 INS/833 DEL, ♂ as reference) markers in conjunction with 1,021 and 894 high-quality genetic sex identification markers, respectively. Sixteen differential loci were randomly chosen from the catalog for validation, with 11 of them meeting the criteria for male-female distinctions. The implementation of cost-effective and efficient technological processes would facilitate the rapid advancement of genetic breeding through expediting the high-throughput development of sex genetic markers for various species.

Conclusions

Our study utilized assembled genome information from male and female individuals obtained from PacBio, in addition to data from short-read sequencing data survey and long-read PacBio sequencing data. We extensively employed genome-wide variant site scanning and identification, high-throughput primer design of target regions, and e-PCR batch amplification, along with statistical analysis and ranking of the long reads depth of the variant sites. Through this integrated approach, we successfully compiled a catalog of large insertion/deletion sites (>100 bp) in both male and female *O. fasciatus*.

Keywords**:** large-segment insertion/deletion; bulk primers design; e-PCR technology; *Oplegnathus fasciatus*; high-throughput identification

Data description

Introduction

Sexual dimorphism was common in the animal kingdom, but the mechanisms of sex determination were diverse [1, 2]. Thus, sex research has always been a topic of interest in biology. Fish occupied a key position in the vertebrate evolutionary system, and because of their wide distribution, large number of species and diverse sex determination mechanisms, they were an important target for the study of sex determination mechanisms [3-9]. However, most fish have a low degree of differentiation between the sex chromosomes, which was difficult to detect from the morphology of the chromosomes [10]. The development of sex-specific molecular markers and sex-control biotechnology has provided an important technical way to increase fish production and economic value and has also laid the foundation for deciphering the molecular mechanism of fish sex determination [11]. Researchers have succeeded in developing sex-specific or correlated molecular markers in many fish

species using a variety of techniques, including SSR, RAPD, AFLP, SNP and In Del [5, 6, 12-17]. However, these methods for obtaining sex-specific markers have many problems, such as low throughput, single markers, cumbersome development and experimental procedures, high development costs, low amplification efficiency and inconvenient on-site detection. The first issue to be addressed was to establish a high-throughput and easy-to-detect marker procedure for sex-specific identification in the investigation of sex dimorphism, mechanisms of sex differentiation, and development and use of sex-producing traits.

In recent years, with the rapid development of genome sequencing technology and the significant decrease in the cost of sequencing, it has become possible to perform whole-genome sequencing of important cultured species. Rapid developments in next-generation sequencing (NGS) technologies have created significant opportunities for the discovery and use of species germplasm resources [4]. In 2002, the genome sequencing of *Takifugu rubripes* was successfully accomplished, marking it as the pioneering model fish species to undergo complete genome sequencing [18]. Subsequently, over the course of nearly 16 years, the whole-genome sequencing of more than 40 fish species, including *Oryzias latipes*, *Gasterosteus aculeatus*, *Pelteobagrus fulvidraco*, *Mola mola*, *Nothobranchius furzeri*, and *Salmo salar*, has been carried out on a national and international scale [19-30]. Over the span of approximately 16 years until 2018, the majority of fish genomes were sequenced utilizing second-generation sequencing technology, specifically Illumina [4, 18-21]. Despite the high throughput capabilities of second-generation sequencing, the relatively short sequencing read lengths posed challenges in obtaining precise genome maps and accurate information on chromosome structural variation. This limitation was especially pronounced in genomes with high complexity and repetitive regions.

At present, third-generation sequencing technology with high precision of long fragments represented by single-molecule real-time sequencing has matured, which included Pacific Biosciences' (PacBio) single-molecule real-time sequencing technology (SMRT), Oxford Nanopore's single-molecule nanopore sequencing technology, and so on. Among them, PacBio RS sequencing technology was the most widely used and commercialized core third-generation sequencing technology and has been widely used in the genome assembly of several species, such as an Asian man, *Oropetium thomaeum* and *Lates calcarifer* [31-33]. The initial assembly of the human telomere-to-telomere (T2T) CHM13 genome, utilizing PacBio HiFi and Oxford

Nanopore ultralong-read sequencing, achieved a contig N50 of 154.26 Mb, resulting in a seamless assembly that fills a crucial gap in the human DNA genetic code map [31, 34-36]. In addition, Hi-C technology based on next-generation sequencing technology has gradually become a favourable tool for chromosome assembly [37]. With the rapid development of sequencing technology and the improvement of assembly technology, the genome integrity and accuracy have been greatly improved, especially in marine fishes, and the assembly now obtained Contig N50 up to the level of 28 Mb for *Sciaenops ocellatus* [38]. Thus, the development of next-generation sequencing technologies has made it possible to exploit high-throughput sex-specific markers in high-precision assembled genome data.

Oplegnathus fasciatus (Temminck & Schlegel, 1844, Fishbase ID: 1709) was a rocky reef fish distributed in a wide range of shallow waters around Korea, Japan, China and Hawaii [39-42]. *O. fasciatus* has become an important fishery resource for offshore cage culture and fish stocking in marine ranches in China, Japan and Korea [41, 42]. *O. fasciatus* has been a commercially important species for sashimi production and recreational angling, with engineered aquaculture in China yielding ex-factory prices of up to US$30/kg [42]. Meanwhile, *O. fasciatus* was characterized by a multiple $X_1X_1X_2X_2/X_1X_2Y$ sex chromosome system [43-46]. Sexual dimorphism has been observed in *O. fasciatus*, with males growing faster than females, especially at weights greater than 500 grams [7, 8, 45, 46]. Therefore, the breeding of new strains with a male predominance has become one of the goals for future breeding of *O. fasciatus*. The development of appropriate genetic sex markers was a prerequisite for achieving a new variety of *O. fasciatus* with male predominance. Currently, available markers for the genetic characterization of *O. fasciatus* were extremely limited and have mainly been identified by AFLP, SSR and Illumina technologies, which constrained the process of exploiting new varieties of *O. fasciatus* [47-49]. The completion of the female and male genomes of *O. fasciatus* has enabled high-throughput development of large segments to visualize genetic sex markers from a genome-wide perspective.

In this work, a catalog of genomic differences between females and males was constructed based on the assembled genome information of *O. fasciatus* by using bidirectional whole-genome sequence alignment [50, 51]. Using the difference catalogs, we obtained datasets of insertion and deletion variant sites larger than 100 bp by screening and used short-read sequencing data survey from genome sequencing and long-read PacBio sequencing data to test the confidence of large segmental variant sites

[52]. Utilizing the *O. fasciatus* sequence tagged sites as a foundation, we employed e-simulation amplification technology to conduct a genome-wide PCR assay aimed at identifying single amplified-banded variant loci between female and male genomes [52]. Then, we performed PCR amplification and agarose gel electrophoresis for the mutant loci detected by simulated PCR. Since *O. fasciatus* exhibited an $X_1X_1X_2X_2/X_1X_2Y$ sex chromosome system, a single primer pair amplifies two bands in males and one band in females.

The paper was organized as follows: First, a fast scanning method for female and male assembled genome variant loci was introduced, and an insertion/deletion dataset of large segments was constructed. Using the constructed insertion/deletion dataset of large fragments, high-throughput candidate molecular marker loci for female and male genetic sex identification of *O. fasciatus* were identified. Then, the new technique of PCR electronic simulation with bulk amplification was used to perform simulated amplification of high-throughput candidate molecular marker loci to screen primers for the single amplification of the female and male genomes with amplification differences. Finally, a high-throughput precision marker dataset for male and female genetic sex identification was obtained and tested by agarose gel electrophoresis (Figure 1).

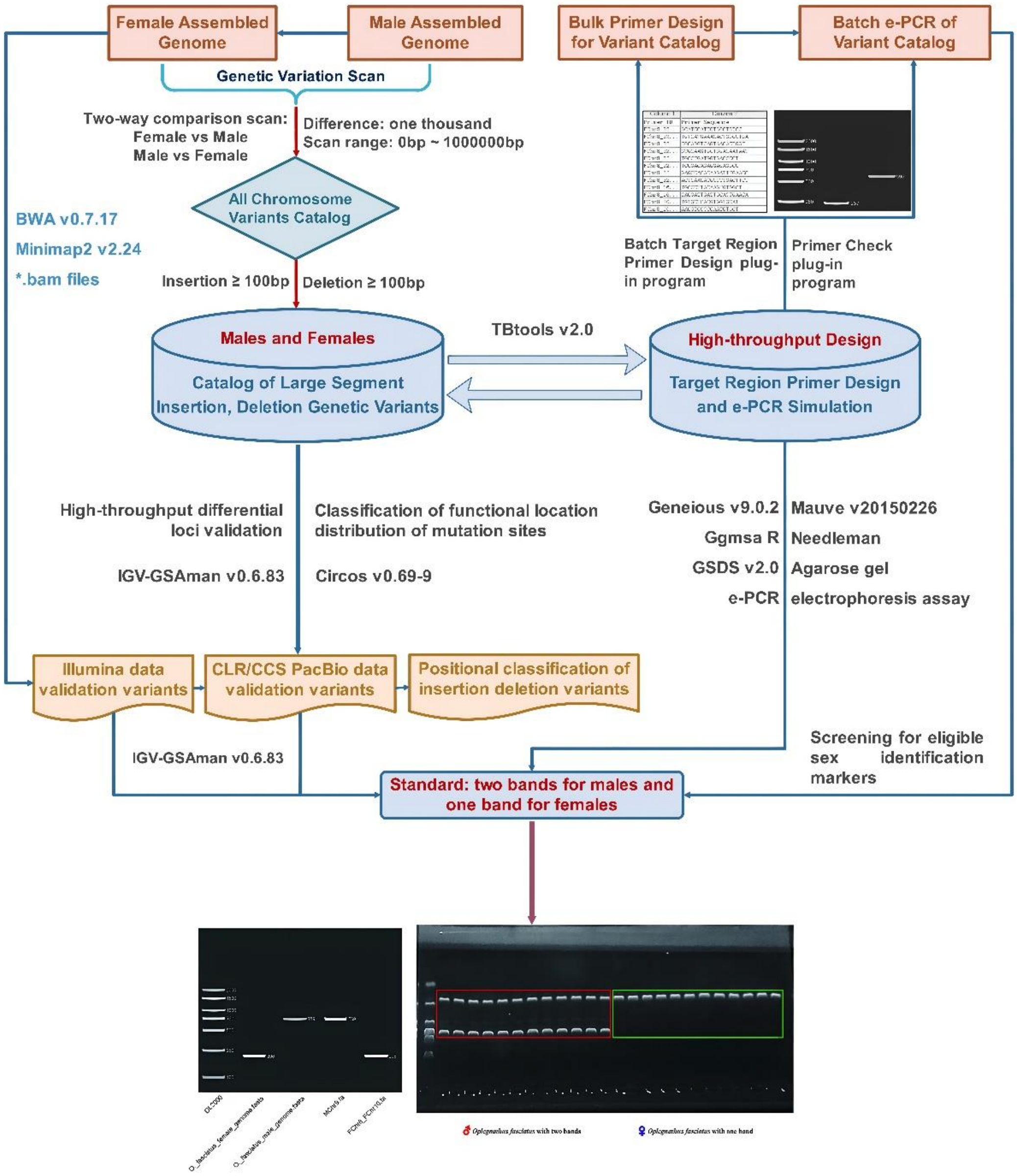

Figure 1: High-throughput exploitation process of genetic sex markers for *O. fasciatus*.

Table 1 Parameters for genome sequencing and assembly quality assessment of chromosome-level genomes of male and female *O. fasciatus*

Illumina platform (Short-read sequencing data survey)		
	Male *O. fasciatus*	Female *O. fasciatus*
Reads number	432,091,798	613,163,122
Raw data (bp)	63,910,932,339	90,702,298,813
Clean data (bp)	56,617,669,600	80,887,039,002
Read length (bp)	148, 147	148, 147

Q20 (%)	97.9, 97.3	98.0, 97.1
Q30 (%)	93.8, 92.0	94.1, 91.4
PacBio Sequel platform (Long-read PacBio sequencing data)		
	Male *O. fasciatus*	Female *O. fasciatus*
Polymerase read number	3,674,737	4,760,021
Polymerase read bases (bp)	39,844,228,109	62,877,786,278
Average polymerase read length (bp)	10,847	13,219
Polymerase read N50 (bp)	18,850	22,964
Subreads number	4,711,720	6,689,662
Total bases of subreads (bp)	39,793,172,442	62,783,888,050
Average subread length (bp)	8,448	9,397
Length of genome (bp)	762,267,613	768,808,243
Number of contigs	1,355	1,372
Contigs N50 (bp)	2,183,645	2,130,780
Number of Scaffolds	23	24
Scaffold N50 (bp)	32,431,321	33,548,962
Genome coverage (X)	251.1	314.6
Complete BUSCOs (%)	98.2	98.0

Note：male and female genomic quality parameters of *O. fasciatus* [45, 46].

Materials and Methods

Sample collection, sequencing and genome quality

Male and female *O. fasciatus* captured from Qingdao, Shandong Province (Yellow Sea) were utilized for genome sequencing and assembly. High-quality genomic DNA was extracted from fresh muscle tissue and blood samples from pairs of male and female *O. fasciatus* using the Illumina platform (Illumina Inc., San Diego, CA, USA) and PacBio Sequel (Pacific Biosciences of California, Menlo Park, CA, USA) [45, 46]. Genomic DNA from fresh muscle tissue and blood samples of male and female *O. fasciatus* pairs was extracted to construct short insert libraries (300~350 bp) for males and females,

respectively, using standard protocols provided by the Illumina HiSeq X Ten platform (Illumina Inc., San Diego, CA, USA). Raw reads totaling approximately 90.7 Gb and 63.9 Gb were generated. After removing splice-containing sequences, low-quality, and redundant reads, approximately 80.8 Gb and 56.6 Gb of pure data were obtained from the genomes of males and females, respectively, corresponding to Q20 averages of 97.55% and 97.60%, respectively (Table 1). Genomic DNA libraries (20 kb) for male and female fish were then constructed using the PacBio Sequel platform (Pacific Biosciences of California, Menlo Park, CA, USA) (Table 1). Sequencing was performed on the PacBio Sequel platform using the Sequel Binding Kit 2.0, Sequel Sequencing Kit 2.1, and Sequel SMRT Cell 1M v2. Seven and five SMRT Cells were used for sequencing the male and female genomes, respectively, with SMRT LINK 5.0 used to filter the raw data from the zero-mode waveguide. We obtained 62.8Gb and 39.8Gb of long read length PacBio sequencing data for female (accession SRP160016) and male (accession SRP220007) genome assembly, respectively [45, 46]. The female and male *O. fasciatus* genome were de novo assembled using Canu v1.4 with Corrected-Error-Rate set to 0.040, followed by redundancy removal using Redundans v0.13c (minCoverage = 15), genome polishing with Arrow tool in SMRT Link 5.0, and final assembly refinement with Pilon using Illumina NGS short reads from the genome surveys. Based on the male and female draft genomes and Hi-C technology, we obtained high-quality genomes at the chromosome level of female (768.8Mb, accession SUB14064808) and male (762.2Mb, accession SUB14065793) *O. fasciatus*, and the contig N50 and scaffold N50 of the male and female fish genomes reached 2.13 Mb/2.18 Mb and 33.5 Mb/32.4 Mb, respectively [45, 46]. To assess the genomic completeness of assembled male and female *O. fasciatus*, we subjected the assembled sequences to BUSCO version 5 evaluation (BUSCO, actinopterygii_odb10), with Complete BUSCOs assessed values up to 98.2% and 98.0% for male and female genomes, respectively (Table 1).

Large fragment variant loci scanning and identification

We employed the genome variant site scanning plugin with accompanying parameters (diff: oneInThousand, VarRange: 0~1,000,000 bp, BatchSize: 500 bp, Min Align

Length for Cov Calc 10,000 bp, Min Align Length for Var Calling: 50000 bp) in TBtools (v 2.0) software to conduct bidirectional comparisons of the complete genomic sequences of male and female organisms [52, 53]. Additionally, we established catalogs containing genomic disparities between males and females. To classify base substitution, insertion, and deletion sites, as well as to screen large insertion-deletion sites (>100 bp) against a differential catalogs, we utilized the Table Row Extract program. In order to further evaluate the stability and accuracy of the dataset containing large segment insertion deletion sites, we employed SyRI software (python3 syri_env/bin/syri -c out.filtered.coords -d out.filtered.delta -r FM.fasta -q genome.format.fa --nosnp) to autonomously detect variant sites (≥100bp) in the assembled genomes of both male and female individuals, and subsequently compared these sites with our existing dataset of variant sites. Additionally, we utilized BWA (v 0.7.8) (bwa-0.7.17/bwa mem -t 6 -SP genome.fa M2_1P.fq.gz M2_2P.fq.gz | samtools view -bS > alignment.bam) and Minimap2 (v2.24) (minimap2 –ax map-pb –t 8 genome.fasta M1.subreads.fasta > M1.subreads.sam) softwares to align the short-read sequencing data generated from Illumina platform and the long-read sequencing data obtained from PacBio platform to the male and female genomes, respectively, in order to validate the large segment variant sites [54, 55]. To further improve the exploitation efficiency of sex-specific markers based on long reads, we calculated the read depth of long reads of insertion deletion sites, and then ranked the insertion deletion site markers based on the depth of long reads, for large segment deletion variants, the lower the depth of long reads, the more effective the markers were, so we constructed a frequency map of the distribution of the depth of long reads of the large segment deletion sites of the male and female genomes, and then classified the markers. Subsequently, employing the Batch Target Region Primer Design plug-in and e-PCR plug-in in TBtools (v 2.0) software, we designed primers for high-throughput amplification and screened them for single amplification bands at the insertion and deletion sites specific to the male and female genomes [52]. To identify the origin of large segment insertion deletion sites, we evaluated whether the variant sites in the dataset were transposons by integrating RepeatModeler (v 2.0.1) and RepeatMasker (v 4.1.2) softwares. The results

of transposon determination obtained by the two softwares were combined and the duplicates were removed for all transposon identification results. At the same time, we utilized TRF (v 4.09.1) software to evaluate whether the variant sites in the dataset were tandem repeats.

The positional distribution of female and male genetic variant loci on the genome was depicted using Circos (v 0.69-9) software [56]. Homology comparison and visualization of insertion-deletion sites with male and female linear genomes were conducted using a combination of Geneious (v 9.0.2) software and Mauve program (v 20150226) [57, 58]. The comparison of genetic sex marker sequences with the Global Alignment standard was performed using the Needleman Wunsch plug-in in TBtools (v 2.0) [52]. Additionally, the visualization of genetic sex marker variant site regions was accomplished using Ggmsa program [59]. GSDS (v 2.0) software was utilized to illustrate the location of the variant site within the functional region of the gene [60]. A total of 24 tails from both male and female *O. fasciatus* specimens were collected and distinguished through gonadal histology. Simultaneously, we gathered 93 tails from wild population samples of *O. fasciatus* along the coasts of Zhejiang and Shandong Provinces, China, to verify the efficacy of sex markers across various geographic regions. The reliability of the genetic sex markers was assessed by randomly identifying male and female populations using e-PCR in conjunction with agarose gel electrophoresis.

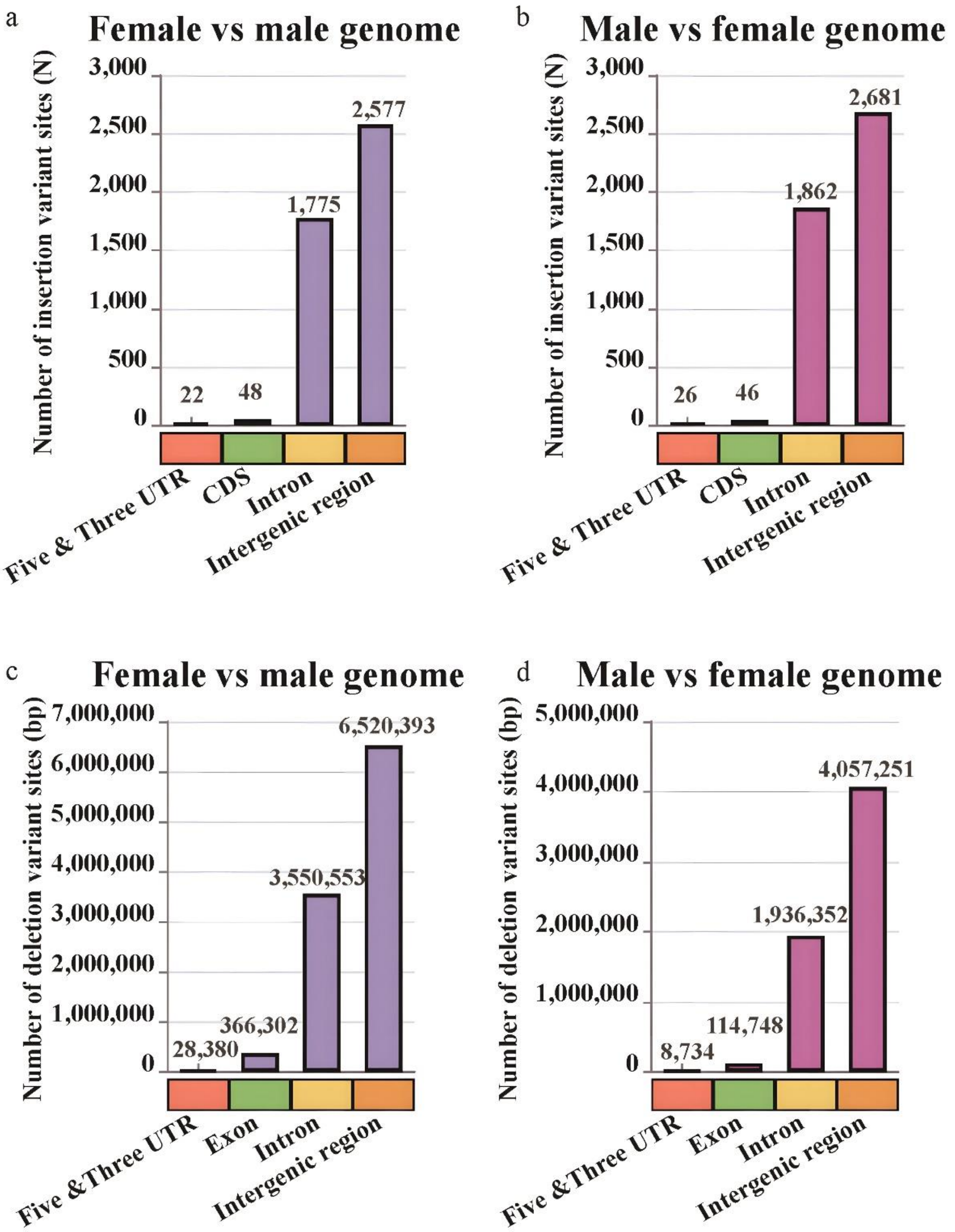

Figure 2: Distribution statistics of insertion and deletion fragment (＞100bp) positions in genomic functional regions of *O. fasciatus*.

(a) Distribution statistics of insertion variant loci using the female fish genome as a reference. (b) Distribution statistics of insertion variant loci utilizing the male fish genome as a point of reference. (c) Distribution statistics of deletion variant length using the female fish genome as a reference. (d) Distribution statistics of deletion variant loci using the male fish genome as a reference.

Note: The colors red, green, yellow, and orange represented the UTR, CDS, intron, and intergenic regions. The frequency of insertions (a, b) was quantified based on the number of events, while deletions (c, d) were characterized by their length in nucleotides.

Results

Construction of insertion/deletion variant loci datasets for large segments

Using the female genome as a reference, we obtained 1,919,620 male and female differential variant sites with unfiltered data, including 191,915 insertion sites, 325,620 deletion sites and 1,402,085 nucleotide substitutions (Supplementary Female_reference_all_variation_Set.xls). To screen suitable field-actionable markers for large-band genetic sex identification, we screened this insertion/deletion variant site catalog for variants larger than 100 bp and obtained 4,422 insertion sites with a differential sequence size of 5,908,147 bp, of which 0.11% were located in 5′ UTRs, 0.38% in 3′ UTRs, 40.14% in intronic regions, 1.08% in CDS regions, and 58.28% in intergenic regions (Figure 2, Figure S1, Supplementary Female_reference_INS/DEL100_Set.xlsx). In addition, we obtained 4,675 deletion sites with a differential sequence size of 10,465,628 bp (Supplementary Female_reference_INS/DEL100_Set.xlsx). As the deletion variant sites spanned multiple genomic functional regions, we performed statistical analysis of the actual locations where the deletion bases were located, with 0.08% located in 5′ UTRs, 0.19% in 3′ UTRs, 33.93% in intronic regions, 3.50% in CDS regions, and 62.30% in intergenic regions (Figure 2, Table 2). The highest number of chromosomal mutations occurred in female fish chromosomes 8 and 10, with 333 and 341 insertion and deletion sites, respectively (Figure 3).

Using the male *O. fasciatus* genome as a reference, 1,926,295 female–male differential variant sites were obtained with unfiltered data, including 326,823 insertion sites, 192,672 deletion sites and 1,406,800 nucleotide substitution sites (Supplementary Female_reference_all_variation_Set.xls). Through large fragment screening (>100 bp), we obtained 4,615 insertion sites with a differential sequence size of 10,100,983 bp, of which 0.13% were located in 5′ UTRs, 0.43% in 3′ UTRs, 40.35% in intronic regions, 1.00% in CDS regions, and 58.09% in intergenic regions (Figure 2, Figure S1, Table 2, Supplementary Male_reference_INS100_Set.xlsx). The chromosome with the highest number of mutations was chromosome 9 in male *O. fasciatus*, in which the number of insertion and deletion sites were 345 and 333, respectively (Figure 3); male chromosome 9 was homologous to chromosomes 8 and 10 of female *O. fasciatus*. Male chromosome 6 had the second highest number of mutations, with 289 insertion sites and 256 deletion sites (Figure 3).

Table 2. Statistics of the positional distribution of insertion/deletion variants in the male and female genomes of *O. fasciatus*.

Type of gene function region	Female genome as a reference		Male genome as a reference	
	Insertion variant loci no. (>100 bp)	Deletion variant length (>100 bp)	Insertion variant loci no. (>100 bp)	Deletion variant length (>100 bp)
5′ UTR	5	7,859	6	2,482
3′ UTR	17	20,521	20	6,252
CDS	48	366,302	46	114,748
Intron	1,775	3,550,553	1,862	1,936,352
Intergenic region	2,577	6,520,393	2,681	4,057,251
Total	4,422	10,465,628	4,615	6,117,085

Note: Insertions (>100 bp) were summarized by number of events. Deletions (>100 bp) were summarized by length in nucleotides.

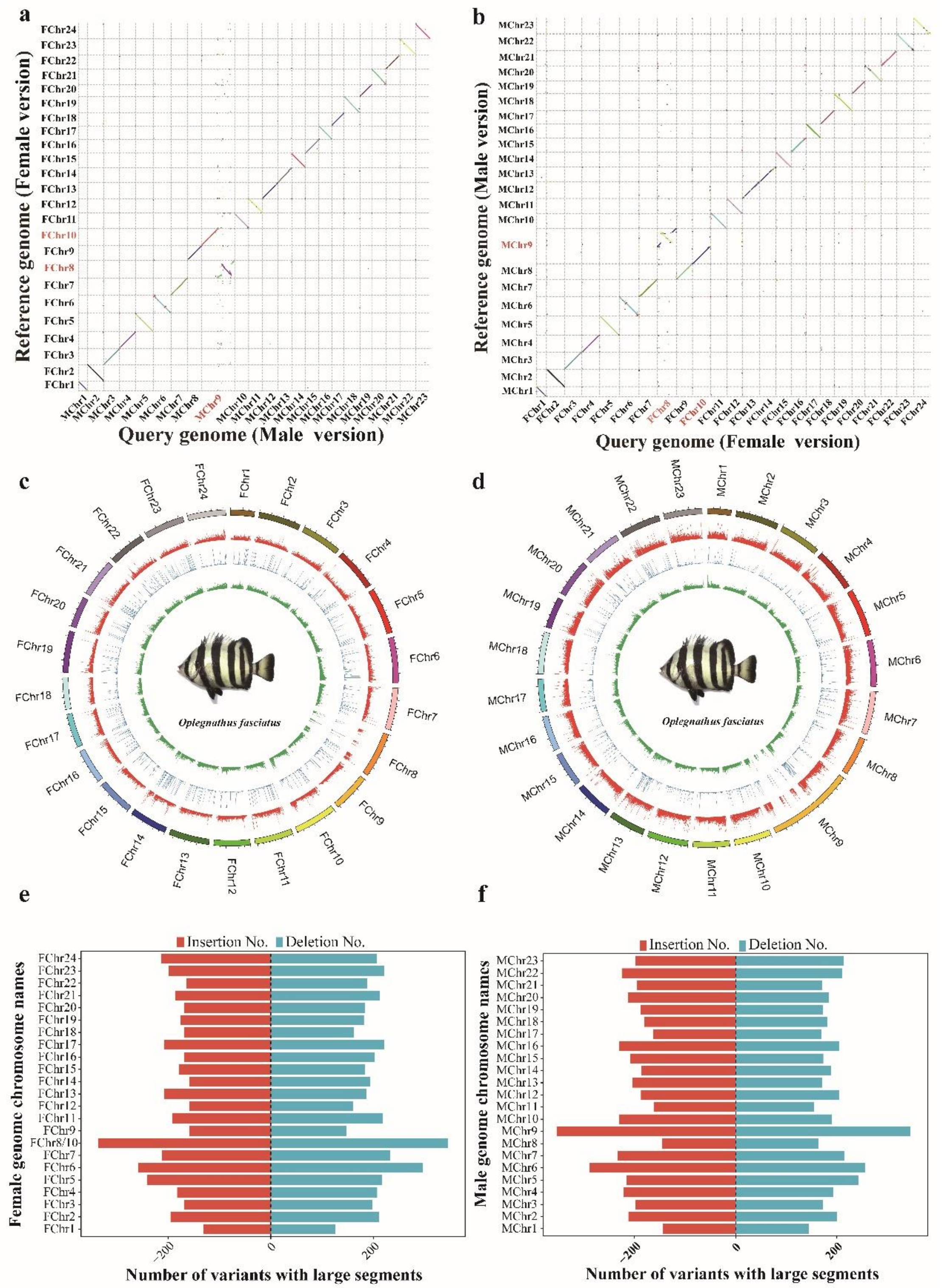

Figure 3: The covariance of genomic sequence differences between the male and female genomes of *O. fasciatus* and distribution statistics of variant sites.

(a) The female genome (reference) of *O. fasciatus* vs. the male genome for homology comparison using the minimap2 software with a 5000 bp sliding window. The female genome was the vertical axis, and female chromosomes 8 and 10 were homologous to male chromosome 9. (b) The comparison of homology between the male genome of *O. fasciatus* and the female genome was conducted using the minimap2 software with a 5000 bp sliding window. The male genome was represented on the vertical axis, revealing homology between male chromosome 9 and female chromosomes 8 and 10. (c) Distribution statistics of variant loci using the female fish

genome as a reference. Insertions (>100 bp) were summarized by number of events. Deletions (>100 bp) were summarized by length in nucleotides. From outer to inner circles were the chromosome name, positional distribution of insertion sites, base length distribution of deletion sites, and base substitution distribution, respectively. (d) Distribution statistics of variant loci using the male fish genome as a reference. (e) - (f) Dual-valued histograms corresponding to the Table 3 showed the number of insertion/deletion sites per chromosome with reference to the female (4,422 inertion sites, 4,675 deletion sites) and male (4,615 inertion sites, 4,385 deletion sites) genomes, respectively, where chromosome 9 in males was homologous to chromosomes 8 and 10 in females. Insertion sites were represented by negative values, and deletion sites were represented by positive values.

The SyRI software was further utilized to assess variant sites (＞100bp) between the male and female reference genomes of *O. fasciatus*. By utilizing the male genome as a reference for comparison with the female genome, a total of 2,045 deletion sites (＞100bp) and 2,110 insertion sites (＞100bp) were identified. Additionally, comparison of the female genome with the male genome as a reference revealed 2,116 large segment deletion sites (＞100bp) and 2,055 large segment insertion sites (＞100bp). Considering the long-read length depth calculations and ranking large segments of deletion sites were beneficial when utilizing short-read sequencing data and long-read PacBio sequencing data to analyze visual differences between male and female genomes. Our study revealed that 1,791 deletion sequence tag sites identified through SyRI scanning overlapped with deletion sites identified in the female fish reference genome, accounting for 84.64% of the total 2,116 deletion sites identified (Fig.4a, Table 3). Meanwhile, the SyRI scanning method identified 1749 deletion STS that intersected with deletion sites in the reference genome of male fish, representing 85.53% of the total 2,045 deletion sites (Fig.4b, Table 3). In the context of large deletion variants, a correlation was observed between the long-read depth and the effectiveness of markers. A frequency distribution map was created for the long-read depths of large deletion loci within male and female genomes, followed by marker categorization. The results indicated that markers with long-read depths below 5.48 (895, ♀ as reference) and 12.50 (1,021, ♂ as reference) demonstrated high recognition rates as sex markers, based on the male and female genomes as reference (Fig.4c-f). The findings further indicated that a substantial proportion of deletion sequence tag sites referencing the female genome were situated in transposon regions (78.44%, n=3,667) and tandem repeat sequences regions (33.67%, n=1,574). Similarly, statistical analysis revealed that a notable percentage of deletion sequence tag sites referencing the male genome were linked to transposon regions (77.08%, n=3,380) and tandem repeat regions (31.90%, n=1,399) (Table 4).

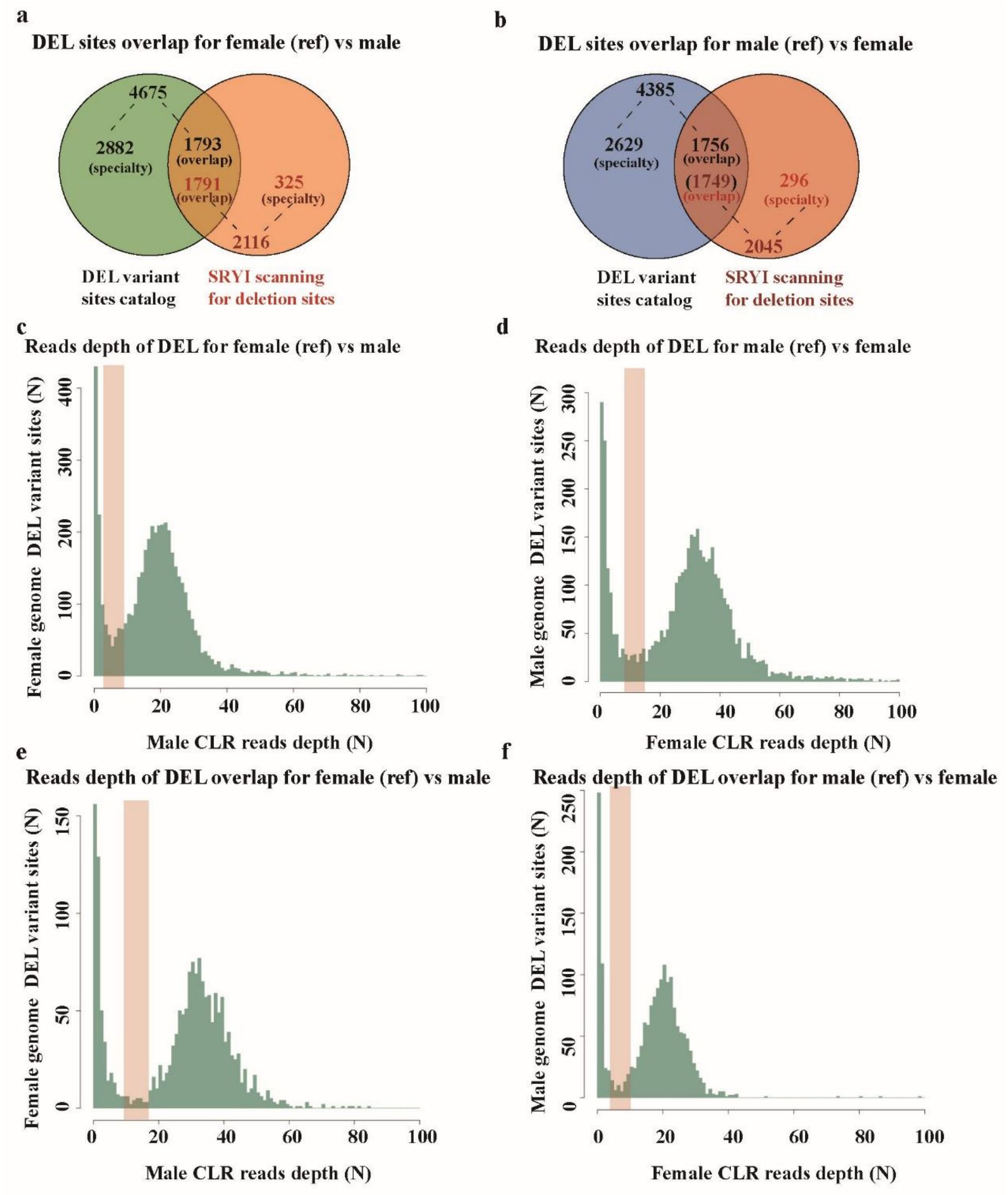

Figure 4: Intersection results of two variant site scanning methods and ranking of variant sites with long read depth frequency distributions

(a) Venn results of deletion locus data were obtained based on TBtools and SyRI software using the female fish genome as a reference, and 1,791 out of 2,116 deletion loci obtained based on SyRI software were shared deletion variant loci. (b) Utilizing the male fish genome as a point of reference for acquiring deletion site data in the context of Venn analysis, it was found that 1,749 out of the 2,045 deletion sites identified through the use of the SyRI software were common deletion variant sites. (c) Depth distribution of CLR reads in male fish using all female genomic deletion locus (＞100bp) information as a reference, with the area where high-quality male and female genetic marker information was located before the orange labeled trough position (~5.4). (d) The depth distribution of CLR reads in females was analyzed by utilizing male genomic deletion sites as a reference (>100bp), focusing on the region preceding the orange labeled trough position (~12.5) where high-quality male and female genetic marker information was present. (e) - (f) Depth distribution of CLR reads in males and females based on intersecting male

and female genomic deletion site information (>100bp), respectively.

Table 3. Statistical cross-validation of multiple scanning of genome-wide large insertion deletion sequence marker sites.

Reference genome	Female vs. Male			Male vs. Female		
Mutation type	Insertion		Deletion	Insertion		Deletion
Variant length (>100 bp)	4,422		4,675	4,615		4,385
Chromosomes	FChr1	131	123	MChr1	140	143
	FChr2	194	211	MChr2	208	194
	FChr3	173	199	MChr3	179	161
	FChr4	181	207	MChr4	214	186
	FChr5	241	220	MChr5	212	237
	FChr6	254	292	MChr6	277	246
	FChr7	212	234	MChr7	231	211
	FChr8/10	333	341	MChr8	143	165
	FChr9	165	146	MChr9	345	333
	FChr11	191	217	MChr10	217	179
	FChr12	157	160	MChr11	159	153
	FChr13	205	187	MChr12	185	203
	FChr14	158	193	MChr13	196	162
	FChr15	182	185	MChr14	186	188
	FChr16	169	202	MChr15	190	160
	FChr17	204	220	MChr16	228	196
	FChr18	168	159	MChr17	159	167
	FChr19	175	183	MChr18	168	171
	FChr20	168	184	MChr19	174	166
	FChr21	185	203	MChr20	205	183
	FChr22	161	183	MChr21	184	167
	FChr23	200	219	MChr22	225	211
	FChr24	215	207	MChr23	190	203
SyRI-based scanning of male and female mutant sites (≥100bp)			2,116			2,045
Intersection of the two methods			1,791 (84.64%)			1,749 (85.53%)

Table 4. Statistics of transposons and tandem repeat sequences attributed to large deletion sequence tag sites obtained with male and female reference genomes

Reference	Mutation type	Transposon	Tandem Repeat	Total

genome				
Female vs. Male	Deletion	3,667 (78.44%)	1,574 (33.67%)	4,675
Male vs. Female	Deletion	3,380 (77.08%)	1,399 (31.90%)	4,385

Table 5. Statistics of large-segment differential loci after primer design, e-PCR amplification, and simulated amplification products and size screening.

Reference genome	Mutation type	Variant length (>100 bp)	High-throughput design of primers for variant sites	e-PCR of primers with single band	Single band and suitable amplification size (100~2000 bp)
Female vs. Male	Insertion	4,422	4,016	5,735	2,786
	Deletion	4,675	1,314	957	853
Male vs. Female	Insertion	4,615	4,131	3,236	2,876
	Deletion	4,385	1,199	882	796

High-throughput primer design and e-PCR bulk simulation for target regions

The identified large insertion/deletion variants were utilized as target regions for sex identification in both male and female genomes. High-throughput primers were designed to generate primer catalogs for the specified target markers. A total of 4,016 and 1,314 primer pairs were obtained by high-throughput primer design for the insertion/deletion target regions, respectively, using females genome as the reference (Table 5). To further test the accuracy and uniqueness of the high-throughput primers across the genome, we performed genome-wide electronic simulated amplification of the obtained primer pairs by using e-PCR. A total of 2,786 and 853 genome-wide single amplification band primer pairs were identified through screening of 5,735 primer pairs and 957 primer pairs with single bands, respectively, resulting in amplification products ranging from 100 to 2,000 base pairs (Table 5, Supplementary Female_Male_INS/DEL100_PrimerSet.xlsx). Utilizing the *O. fasciatus* male genome as a point of reference, 4,131 and 1,199 primer pairs were obtained to target insertions/deletions of specific variant sites, respectively (Table 2, Supplementary Female_Male_INS/DEL100_PrimerSet.xlsx). Notably, 2,876 and 796 primer pairs for whole-genome single amplification bands (100~2,000bp) were identified through screening of 3,236 primer pairs and 882 primer pairs with single band (Table 5,

Supplementary Female_Male_INS/DEL100_PrimerSet.xlsx).

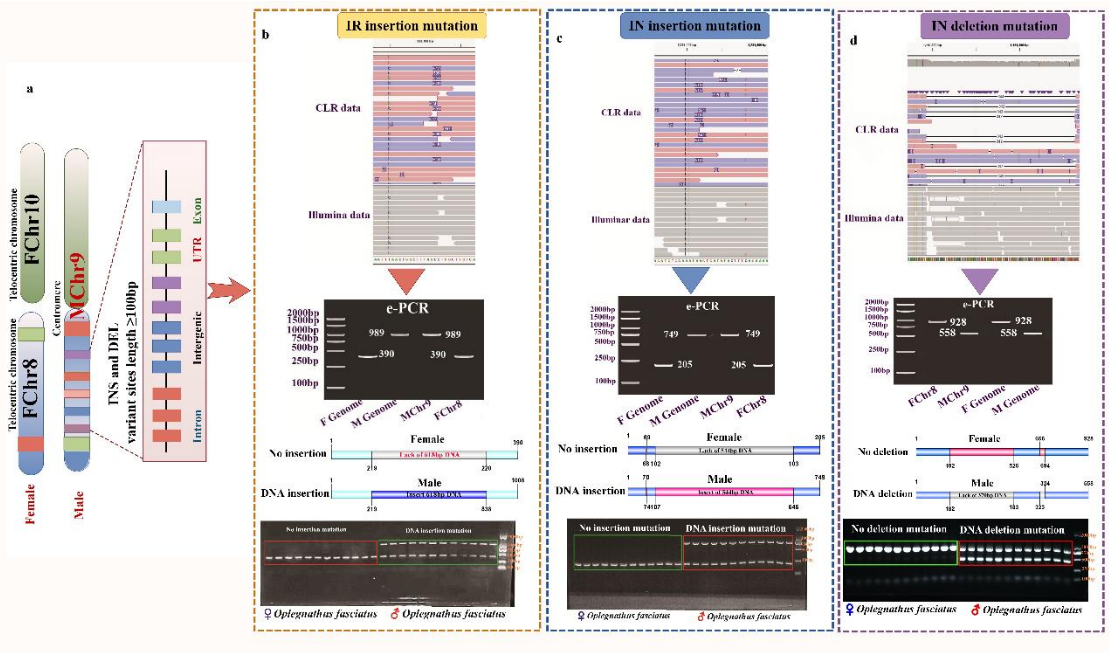

Figure 5: Representative genetic sex marker (insertion deletion variant sites) located in the intergenic region (IR), Intronic region (IN) of the *O. fasciatus* genome (STOSOd393e).

(a) Location of male and female sex markers in the male (MChr9) and female (FChr8) genomes. (b) Male and female sex genetic markers were located at loci in the intergenic region, using the female fish genome as a reference (Figure S2). Illumina and CLR clean data of bam comparison to the genome for insertion/deletion site validity detection. Red represented length long data from PacBio and gray represented was Illumina clean data. The e-PCR amplified bands were 390bp and 989bp in size, corresponding to DNA insertion and deletion patterns and agarose gel electrophoresis for identification of male and female genetically different populations. (c) By utilizing the female genome as a point of comparison, the genetic markers for male and female sexes were identified at specific insertion sites within *nuf2* gene introns (Figure S3, Figure S4). The resulting e-PCR amplified bands, measuring 205bp and 749bp in size, were indicative of distinct DNA insertion patterns and were utilized in agarose gel electrophoresis for the purpose of distinguishing between male and female genetically differentiated groups. Males exhibited two amplified bands, while females exhibited only one. (d) By utilizing the female fish genome as a point of comparison, the genetic markers for male and female sexes were situated within the deletion sites located in the introns of genes (Figure S5, Figure S6). The amplified bands produced through e-PCR were observed to be 558bp and 928bp in length, aligning with the DNA deletion patterns and agarose gel electrophoresis maps utilized for distinguishing genetically distinct groups of males and females. Specifically, two bands were amplified in males, while only one band was observed in females.

Validation of molecular markers for genetic sex identification of females and males

We selected markers located in the intergenic and intragenic regions for female and male genetic sex identification for validation testing. Using the female genome as a reference, a 599 bp DNA fragment was inserted into the male genome at position 880,783 bp on female chromosome 8 (after TTTAGC), corresponding to e-PCR amplicons of 390 bp and 1008 bp, respectively (Figure 5, Figure S2). Sequence comparisons of the genetic sex markers showed that the genetic differences between male and female markers were mainly concentrated in the region of 200~800 bp. This variant locus was further confirmed as a valid insertion site using female fish as a reference genome, synthesized using genomic survey data from short-read sequencing data survey of male fish and long-read sequencing data from PacBio platform for read assignment (Figure 4, Figure S1). The results of PCR amplification and agarose gel electrophoresis were consistent with the results of e-PCR corresponding to two bands (390 bp and 1,008 bp) for males and one band (390 bp) for females. According to the genetic sex identification marker localization, the marker lies between the *samd3* gene (sterile alpha motif.) (Ofa002334) and *elf3* gene (E74-like factor 3) (Ofa002335) in the female genome (Figure 5, Figure S2).

The intragenic markers obtained from screening were also used to test the effectiveness of female and male genetic sex identification in *O. fasciatus*. In the region corresponding to the intron of the female *nuf2* gene (component of the NDC80 kinetochore complex) (Ofa002358) on chromosome 8 (2,231,443 bp, after GCAAATA), a 518 bp DNA fragment was inserted into the intron of the male *nuf2* gene, corresponding to e-PCR amplified fragment sizes of 206 bp and 739 bp, respectively (Figure S3). With the female genome as a reference, we utilized the male short-read sequencing data and long-read PacBio sequencing data for read assignment and further confirmed that this candidate variant site was a valid insertion site (Figure S4). Comparison of the sequences for the genetic sex marker showed that the genetic differences between the male and female markers were mainly in the region of 90~650 bp. The results of PCR amplification and agarose gel electrophoresis further showed that the males exhibited two bands (206 bp and 739 bp), and the females had one band (206 bp), in agreement with the e-PCR results (Figure S4).

In addition to the male insertion genetic sex markers, a male deletion genetic sex marker was also identified. In the region corresponding to female chromosome 8 from

1,161,186 bp (after GATGAGGAAAG) to 1,161,530 bp (before TATGAAGTCT), the males were missing a 343 bp DNA fragment, corresponding to e-PCR amplicon sizes of 558 bp and 928 bp, respectively (Figure S5). A comparison of sequence differences, short-read sequencing data and long-read PacBio sequencing data read assignments all confirmed that the genetic differences between male and female markers were mainly concentrated in the 182~551 bp and 665~693 bp regions (Figure S6). PCR amplification and agarose gel electrophoresis assays showed two bands in males (558 bp and 928 bp) and one band in females (928 bp), which was consistent with the results of e-PCR (Figure S6). A similar genetic sex marker for the deletion locus in males was also identified in the region corresponding to chromosome 8 of females at 921,537 bp (after AAATGTGGCGG), with deletion of a 299 bp DNA fragment and insertion of a 119 bp DNA multi-fragments in males. PCR amplification and agarose gel electrophoresis tests showed two bands in males (748 bp and 926 bp) and one band in females (926 bp), consistent with the e-PCR results (Figure S7, Figure S8).

Discussion

The majority of fish exhibited limited sex chromosome differentiation, which posed challenges in identifying them based on chromosome morphology [10]. Additionally, fish sex was influenced by environmental factors, particularly temperature, resulting in discrepancies between the physiological phenotype and genotype of sex [11]. Consequently, it was crucial within the field of aquaculture to discover a straightforward approach capable of rapidly determining the genetic composition of fish sex [61]. DNA molecular markers were created by exploiting the prevalence of polymorphisms in the genomic DNA of eukaryotic organisms, which serve as indicators of individual variations at the DNA level [7]. A review of the literature revealed that over the past decade, approximately 48% of recent studies on male and female sex determination had predominantly employed molecular biology techniques such as RFLP, RAPD, SSR, and AFLP [61-72]. Meanwhile, with the advancement of second-generation high-throughput sequencing technologies, particularly Illumina, the utilization of sex markers based on SNP/InDel variants has increased to approximately 51% [16, 73-78]. However, the advancement of sex-controlled breeding has faced limitations due to various factors, such as low throughput of the above methods, cumbersome experimental procedures, expensive development cost, high rate of false positives, unstable markers, and inconvenient on-site testing. With the emergence and

rapid advancement of third-generation sequencing technology, exemplified by long-fragment read-length PacBio sequencing, the development of third-generation high-throughput sex markers based on insertions or deletions of large fragments (>100 bp) has become feasible [37, 45, 46]. In this study, *O. fasciatus* with the $X_1X_1X_2X_2/X_1X_2Y$ system served as the focal point [44-46, 48]. By utilizing the assembled genome information of male and female individuals acquired through PacBio sequencing, along with data from the short-read sequencing data and long-read PacBio sequencing data, we extensively employed genome-wide variant sites multiple scanning and identification, STS long reads depth evaluation and ranking, high-throughput design of primers for the target regions, and e-PCR batch amplification (Figure 1). By employing a comprehensive approach involving genome-wide variant site scanning, high-throughput primer design within the desired region, and e-PCR batch amplification and validation methodologies, we successfully exploited a catalog encompassing insertion/deletion loci in large segments (>100 bp) for both male and female *O. fasciatus* (Figure 2, Figure 3, Table 2, Table 3). Subsequently, a high-throughput identification catalog of sex-specific markers was established through this methodology, leading to the discovery of 3,639 (2,786 INS/853 DEL, ♀ as reference) and 3,672 (2,876 INS/833 DEL, ♂ as reference) markers, along with 1,021 and 894 high-quality genetic sex identification markers, respectively, serving as a reliable reference for sex identification (Table 2, Table 3). Following agarose gel electrophoresis, two amplification bands for males and one amplification band for females were successfully identified, enabling rapid differentiation between the sexes in *O. fasciatus*. This advancement in sex marker development, utilizing third-generation high-throughput and precise techniques, will expedite the progress of genetic breeding and facilitate the establishment of genetic sex markers.

Variation in genome structure was intricately linked to human diseases, evolution, gene regulation, and species phenotypes in the biological realm, making it a prominent area of study in genomics research [79]. Previous studies have shown that DNA sequences with insertions and deletions undergo increased selective pressure [80-84]. The presence of deletions and insertions in DNA sequences, regardless of their location in noncoding or coding regions, inevitably influences the functionality of the original sequence to varying extents [79]. Furthermore, evidence suggested that the length and placement of insertions were not random but rather heavily influenced by the adjacent DNA sequence [80-82, 85, 86]. Prior research has indicated that insertion deletion

variants accounted for 16%, 24%, 13%, 25%, and 15% of genomic genetic polymorphisms in various organisms, including *Drosophila melanogaster*, *Caenorhabditis elegans*, mouse, human, and *Arabidopsis thaliana* [34-36, 87-94]. Recent advancements in long length sequencing technology have allowed for a more precise examination of human genetic variation, revealing that insertions and deletions collectively contribute to a high portion (13.8%, 11.1%) of genetic polymorphisms in the human genome [90-94]. The focus of genomic structural variation studies has predominantly been on single nucleotide polymorphisms (SNPs) and insertions/deletions (InDels) of less than 50 base pairs due to the limitations of high-throughput sequencing technologies for short read lengths. However, larger genomic structural variants, which encompassed genetic variations not fully captured by SNPs and InDels, were closely linked to biological phenotypes and might significantly influence phenotypic traits, disease susceptibility, and adaptive capacity [79]. The utilization of long-read length sequencing technologies such as PacBio and ONT has enabled the generation of extended DNA sequences, thereby enhancing the assembly of diploid genomes of superior quality and the identification of structural variants (SVs) across substantial genomic regions [95]. Prvious research has documented seven deletions (>100bp) within approximately 700bp large segments of the *ZmBAM1d* gene, demonstrating a positive association with maize kernel weight. These deletions were consistently inherited in high-yielding maize lines [96]. In mammalian studies, a deletion of approximately 40Mb on chimpanzee chromosome 2, as compared to humans and gorillas within the great ape family, has been identified, leading to stable genetic distinctions among species [97]. Similar results have been documented in *Astyanax mexicanus*, *Gasterosteus aculeatus*, racehorses, and dogs [98-101]. The emergence of long read-length sequencing technology has propelled large fragment genomic structural variation to the forefront of genetic variation research, particularly in investigations of species' adaptive evolution [95, 96, 102, 103]. The findings of this study indicated that insertions and deletions identified using the female and male *O. fasciatus* genomes as references constituted 26.96% and 26.97% of the genetic polymorphisms, respectively. Our research has contributed new insights into the prevalence of large-segment insertion-deletion events for *O. fasciatus*, indicating that these events predominantly occurred in the intergenic region in 58.09%~66.33% of instances, with the intronic region following closely at 31.65%~40.35% of cases (Figure 2, Figure S1 Table 2). We also observed a higher proportion of deletion sites

compared to insertion sites. A similar study conducted on the β-fibrinogen intron of birds reported similar findings [104]. Furthermore, this study presented novel findings indicating that over 77% of the identified large deletion sequence tag sites in the genomes of both female and male *O. fasciatus* were attributed to transposons (Table 4). It was evident that transposons were prevalent in the animal genome and significantly contribute to its evolutionary processes. Previous research has demonstrated that a significant portion (69.5%) of genomic structural variation in large segments of tomato was situated within the transposon region, which was closely linked to desirable traits in tomato [105]. Comparable studies have also been conducted on gene regulation in human brain diseases and genomic structural variation in evolutionary distinct phenotypes of great apes [103, 106]. Transposons have the potential to drive genome and chromosome evolution through various mechanisms such as insertion-induced gene variation, gene retrotransposition, chromosome recombination, gene duplication, and serving as sites for ectopic recombination. These findings underscored the importance of transposons in facilitating adaptive evolution for *O. fasciatus*. The presence of large fragments of insertion deletion sequence marker sites was frequently influenced by natural selection pressures, with their formation being intricately linked to adjacent DNA sequences and commonly correlated with characteristic phenotypic traits and aberrant gene regulation [83, 84, 102, 103, 105, 106]. Specifically, the manifestation of albino symptoms in dogs resulting from a 965-kb deletion on chromosome 18 exhibited stable inheritance patterns, while a 4.8 Mb deletion variant on the chromosome of male domestic chickens similarly displayed heritability across subsequent generations [100, 107]. Consistent inheritance of variants within populations has also been documented in academic research on maize (*ZmBAM1d*), *Astyanax mexicanus*, *Ailuropoda melanoleuca* (*Bace2*), great apes (*acan*), and human diseases such as hypertension (*ace*) [96, 98, 103, 108]. The findings from the population genetic sex marker amplification analysis conducted in this study indicated that the insertion-deletion markers exhibited stability in both male and female populations of *O. fasciatus*. Additionally, analysis of gonadal histology and sex-validated marker data from various geographic populations demonstrated consistent inheritance of large segments of the insertion deletion markers in individuals of both sexes across different geographic regions (Figure S9). This observation provided additional evidence to support the theory that large-segment insertions and deletions are not a random occurrence but rather influenced by natural selection pressures [80, 82-84]. Based on the present findings, the stability of large

segment insertion and deletion in sequence tag sites was evident. However, this should not be taken to imply that all sequence tag sites exhibit this characteristic. A comprehensive evaluation of the evolutionary rate of sequence tags and the expansion of variant marker detection are necessary for ongoing research in this area.

Conclusions

These results highlighted the substantial prevalence of insertion and deletion events in the genetic makeup derived from the $X_1X_1X_2X_2/X_1X_2Y$ sex determination system of *O. fasciatus*. In our study, we successfully introduced a novel approach for the development of genetic sex markers in *O. fasciatus*. This approach involved the utilization of a primer batch electronic design technique, which utilized the insertion and deletion loci as the target regions. Additionally, we implemented an electronic batch amplification technique, allowing for the efficient and rapid high-throughput identification and screening of a substantial number of variant sites. This methodology represents a significant advancement in the field of *O. fasciatus* genetic sex marker development. The study yielded a greater number of genetic sex markers with higher amplification efficiency compared to those generated by previous technologies spanning the first or second generation. This methodology was applicable not only to the efficient generation of genetic sex markers for the $X_1X_1X_2X_2/X_1X_2Y$ sex-determination system but also to the determination of genetic sex in various sex-determination types (including autosomal and sex chromosome differences). It was particularly well suited for sex-determination systems featuring heteromorphic chromosomes, such as the XY-type, ZW-type, XX/XY_1Y_2-type, ZZ/ZW_1W_2-type, and other species with different sex-determination mechanisms.

Ethics Statement

This research was approved by the Animal Care and Use committee of the Chinese Academy of Science.

Competing Interests

The authors declare no competing interests.

Data Availability

All additional supporting data are available in the GigaScience repository, GigaDB [109].

Availability of Supporting Source Code and Requirements

The code used for statistics on insertions and deletions as well as annotation scripts and base variant

region visualization scripts are stored in jihuGitLab (https://jihulab.com/gigascience/sex-markers) with Username: xiaoyongshuang19@163.com and Login Password: Wwyxys1982 [109].

Abbreviations

CLR: Continuous Long Reads; CCS: circular consensus sequence.

References

1. Hughes JF, Skaletsky H, Pyntikova T et al. Chimpanzee and human Y chromosomes are remarkably divergent in structure and gene content, Nature 2010;463:536-539.
2. Bitencourt JA, Sampaio I, Ramos RT et al. First Report of Sex Chromosomes in Achiridae (Teleostei: Pleuronectiformes) with Inferences About the Origin of the Multiple X(1)X(1)X(2)X(2)/X(1)X(2)Y System and Dispersal of Ribosomal Genes in *Achirus achirus*, Zebrafish 2017;14:90-95.
3. Chen S, Zhang G, Shao C et al. Whole-genome sequence of a flatfish provides insights into ZW sex chromosome evolution and adaptation to a benthic lifestyle, Nat Genet 2014;46:253-260.
4. Lu G, Luo M. Genomes of major fishes in world fisheries and aquaculture: Status, application and perspective, Aquaculture and Fisheries 2020;5:163-173.
5. Chen SL, Ji XS, Shao CW et al. Induction of mitogynogenetic diploids and identification of WW super-female using sex-specific SSR markers in half-smooth tongue sole (*Cynoglossus semilaevis*), Mar Biotechnol (NY) 2012;14:120-128.
6. Pan ZJ, Li XY, Zhou FJ et al. Identification of Sex-Specific Markers Reveals Male Heterogametic Sex Determination in Pseudobagrus ussuriensis, Mar Biotechnol (NY) 2015;17:441-451.
7. Ma Y, Xiao Y, Xiao Z et al. Identification of Male-Specific Molecular Marker and Development of PCR-Based Genetic Sex Identification Technique in Spotted Knifejaw (*Oplegnathus punctatus*), Mar Biotechnol (NY) 2022;24:969-978.
8. Wu Y, Xiao Y, Xiao Z et al. Identification of Male-Specific Molecular Markers by Recombination of RhoGEF10 Gene in Spotted Knifejaw (*Oplegnathus punctatus*), Genes (Basel) 2022;13.
9. Li XY, Gui JF. Diverse and variable sex determination mechanisms in vertebrates, Sci China Life Sci 2018;61:1503-1514.
10. Chen J, Zhu Z, Hu W. Progress in research on fish sex determining genes, Water Biology and Security 2022;1.
11. Shen Z-G, Wang H-P. Molecular players involved in temperature-dependent sex determination and sex differentiation in Teleost fish, Genetics Selection Evolution 2014;46:26.
12. Dan C, Mei J, Wang D et al. Genetic differentiation and efficient sex-specific marker development of a pair of Y- and X-linked markers in yellow catfish, Int J Biol Sci 2013;9:1043-1049.
13. Liao X, Xu G, Chen SL. Molecular method for sex identification of half-smooth *tongue sole* (Cynoglossus semilaevis) using a novel sex-linked microsatellite marker, Int J Mol Sci 2014;15:12952-12958.
14. Ning Y, Liu X, Wang ZY et al. A genetic map of large yellow croaker *Pseudosciaena crocea*, Aquaculture 2007;264:16-26.
15. Xiao S, Wang P, Zhang Y et al. Gene map of large yellow croaker (*Larimichthys crocea*) provides insights into teleost genome evolution and conserved regions associated with growth, Sci Rep 2015;5:18661.
16. Lin A, Xiao S, Xu S et al. Identification of a male-specific DNA marker in the large yellow croaker

(*Larimichthys crocea*), Aquaculture 2017;480:116-122.
17. Tao W, Zhu X, Cao J et al. Screening and characterization of sex-linked DNA markers in Mozambique tilapia (*Oreochromis mossambicus*), Aquaculture 2022;557.
18. Aparicio S, Chapman J, Stupka E et al. Whole-genome shotgun assembly and analysis of the genome of *Fugu rubripes*, Science 2002;297:1301-1310.
19. Mitani H, Kamei Y, Fukamachi S et al. The medaka genome: why we need multiple fish models in vertebrate functional genomics, Genome Dyn 2006;2:165-182.
20. Jones FC, Grabherr MG, Chan YF et al. The genomic basis of adaptive evolution in threespine sticklebacks, Nature 2012;484:55-61.
21. Davidson WS, Koop BF, Jones SJ et al. Sequencing the genome of the Atlantic salmon (*Salmo salar*), Genome Biol 2010;11:403.
22. Gong G, Dan C, Xiao S et al. Chromosomal-level assembly of yellow catfish genome using third-generation DNA sequencing and Hi-C analysis, GigaScience 2018;7:giy120.
23. Luo H, Zhang Y, Liu F et al. The male and female genomes of golden pompano (*Trachinotus ovatus*) provide insights into the sex chromosome evolution and rapid growth, Journal of Advanced Research 2023.
24. McGaugh SE, Gross JB, Aken B et al. The cavefish genome reveals candidate genes for eye loss, Nature Communications 2014;5:5307.
25. Pan H, Yu H, Ravi V et al. The genome of the largest bony fish, ocean sunfish (*Mola mola*), provides insights into its fast growth rate, GigaScience 2016;5:s13742-13016-10144-13743.
26. Schartl M, Walter RB, Shen Y et al. The genome of the platyfish, *Xiphophorus maculatus*, provides insights into evolutionary adaptation and several complex traits, Nature genetics 2013;45:567-572.
27. Valenzano DR, Benayoun BA, Singh PP et al. The African turquoise killifish genome provides insights into evolution and genetic architecture of lifespan, Cell 2015;163:1539-1554.
28. Xiao Y, Liu J, Wei J et al. Improved high-quality reference genome of red drum facilitates the processes of resistance-related gene exploration, Scientific Data 2023;10:774.
29. Xu M-R-X, Liao Z-Y, Brock JR et al. Maternal dominance contributes to subgenome differentiation in allopolyploid fishes, Nature Communications 2023;14:8357.
30. Zhang W, Yang Y, Hua S et al. Chromosome-level genome assembly and annotation of the yellow grouper, *Epinephelus awoara*, Scientific Data 2024;11:151.
31. Shi L, Guo Y, Dong C et al. Long-read sequencing and de novo assembly of a Chinese genome, Nature Communications 2016;7:12065.
32. VanBuren R, Bryant D, Edger PP et al. Single-molecule sequencing of the desiccation-tolerant grass *Oropetium thomaeum,* Nature 2015;527:508-511.
33. Vij S, Kuhl H, Kuznetsova IS et al. Chromosomal-Level Assembly of the Asian Seabass Genome Using Long Sequence Reads and Multi-layered Scaffolding, PLoS Genet 2016;12:e1005954.
34. Aganezov S, Yan SM, Soto DC et al. A complete reference genome improves analysis of human genetic variation, Science 2022;376:eabl3533.
35. Nurk S, Koren S, Rhie A et al. The complete sequence of a human genome, Science 2022;376:44-53.
36. Schneider VA, Graves-Lindsay T, Howe K et al. Evaluation of GRCh38 and de novo haploid genome assemblies demonstrates the enduring quality of the reference assembly, Genome research 2017;27:849-864.
37. Burton JN, Adey A, Patwardhan RP et al. Chromosome-scale scaffolding of de novo genome

assemblies based on chromatin interactions, Nat Biotechnol 2013;31:1119-1125.
38. Xiao Y, Liu J, Wei J et al. Improved high-quality reference genome of red drum facilitates the processes of resistance-related gene exploration, Sci Data 2023;10:774.
39. Schembri PJ, Bodilis P, Evans J et al. Occurrence of barred knifejaw, *Oplegnathus fasciatus* (Actinopterygii: Perciformes: Oplegnathidae), in Malta (Central Mediterranean) with a discussion on possible modes of entry 2010;40:101-104.
40. Mundy BCJBMBiZ. Checklist of the fishes of the Hawaiian Archipelago 2005;6:1-704.
41. An HS, Kim MJ, Hong SWJG et al. Genetic diversity of rock bream *Oplegnathus fasciatus* in Southern Korea, Genes and genomics 2008;30:451-459.
42. Xiao Y, Li J, Ren G et al. Pronounced population genetic differentiation in the rock bream *Oplegnathus fasciatus* inferred from mitochondrial DNA sequences 2016;27:2045-2052.
43. Xu D, Lou B, Bertollo LAC et al. Chromosomal mapping of microsatellite repeats in the rock bream fish *Oplegnathus fasciatus*, with emphasis of their distribution in the neo-Y chromosome 2013;6:1-6.
44. Xue R, An H, Liu Q et al. Karyotype and Ag-NORs in male and female of *Oplegnathus punctatus* 2016;47:626-632.
45. Xiao Y, Xiao Z, Ma D et al. Genome sequence of the barred knifejaw *Oplegnathus fasciatus* (Temminck & Schlegel, 1844): The first chromosome-level draft genome in the family Oplegnathidae 2019;8:giz013.
46. Xiao Y, Xiao Z, Ma D et al. Chromosome-Level Genome Reveals the Origin of Neo-Y Chromosome in the Male Barred Knifejaw *Oplegnathus fasciatus*, iScience 2020;23:101039.
47. Xu T, Shao C, Liao X et al. Isolation and characterization of polymorphic microsatellite DNA markers in the rock bream (*Oplegnathus fasciatus*) 2009;10:527-529.
48. Xu D, Lou B, Xu H et al. Isolation and characterization of male-specific DNA markers in the rock bream *Oplegnathus fasciatus* 2013;15:221-229.
49. Gong J, Zhao J, Ke Q et al. First genomic prediction and genome-wide association for complex growth-related traits in Rock Bream (*Oplegnathus fasciatus*) 2022;15:523-536.
50. Yong SX. NCBI Sequence Read Archive https://identifiers.org/ncbi/insdc.sra:SRP220007. 2020.
51. Xiao Y, Xiao Z, Ma D et al. Supporting data for "Genome sequence of the barred knifejaw *Oplegnathus fasciatus* (Temminck & Schlegel, 1844): the first chromosome-level draft genome in the family Oplegnathidae", GigaScience Database 2019:http://dx.doi.org/10.5524/100556.
52. Chen C, Wu Y, Xia R. A painless way to customize Circos plot: From data preparation to visualization using TBtools, iMeta 2022;1.
53. Chen C, Chen H, Zhang Y et al. TBtools: An Integrative Toolkit Developed for Interactive Analyses of Big Biological Data, Mol Plant 2020;13:1194-1202.
54. Li H. Minimap2: pairwise alignment for nucleotide sequences, J Bioinformatics 2018;34:3094-3100.
55. Li H, Durbin RJb. Fast and accurate short read alignment with Burrows–Wheeler transform 2009;25:1754-1760.
56. Krzywinski M, Schein J, Birol I et al. Circos: an information aesthetic for comparative genomics 2009;19:1639-1645.
57. Kearse M, Moir R, Wilson A et al. Geneious Basic: an integrated and extendable desktop software platform for the organization and analysis of sequence data 2012;28:1647-1649.
58. Darling AE, Mau B, Perna NTJPo. progressiveMauve: multiple genome alignment with gene gain, loss and rearrangement 2010;5:e11147.

59. Zhou L, Feng T, Xu S et al. ggmsa: A visual exploration tool for multiple sequence alignment and associated data 2022;23:bbac222.
60. Hu B, Jin J, Guo A-Y et al. GSDS 2.0: an upgraded gene feature visualization server 2015;31:1296-1297.
61. Mei J, Gui JF. Genetic basis and biotechnological manipulation of sexual dimorphism and sex determination in fish, Sci. China Life Sci. 2014;44:5.
62. Agawa Y, Saiki T, Miyamoto T et al. Identification of the male sex-linked DNA sequence of cultured white trevally *Pseudocaranx dentex* 2022;88:319-327.
63. Goyat S, Grewal A, Singh D et al. Sex-linked AFLP marker identification in *dioecious Betelvine* (Piper betle L.) 2019;94:422-427.
64. Hashem MHJDJoVS. Early sexing of Tilapia nilotica (*Oreochromis niloticus*) by using short sequence repeats (SSRs) molecular markers 2022;8:22-25.
65. Hua J, Qiang J, Tao Y et al. Development and validation of a PCR-RFLP/TaqMan MGB probe method for rapid sex identification of largemouth bass (*Micropterus salmoides*) 2023;30:101593.
66. Jia X, Lu J, Tang X et al. A new method for molecular sex identification in the emu (Dromaius novaehollandiae) 2023:1-6.
67. Lajan S, Al-Barzinji YJIJoAS. Detection of quantitative loci correlation with growth traites in local quail using PCR-RFLP technique 2022;53:16-26.
68. Law D, Ping VC, Yee TC et al. Use of amplified fragment length polymorphism and sequence characterized amplified region marker for identifying the sex of the *Oxyeleotris marmorata* 2021.
69. Mohamed G, Youssef M, Mohamed E et al. Identification of Sex-specific Molecular Markers in Barbel and Nile Carp using SCoT and ISSR Markers 2021;52:60-73.
70. Panagiotopoulou H, Marzecki K, Gawor J et al. Extensive search of genetic sex markers in Siberian (*Acipenser baerii*) and Atlantic (*A. oxyrinchus*) sturgeons 2023;573:739517.
71. Vaux F, Aycock HM, Bohn S et al. Sex identification PCR–RFLP assay tested in eight species of Sebastes rockfish, Conservation Genetics Resources 2020;12:541-544.
72. Wen M, Zhang Y, Wang S et al. Characterization of sex locus and sex-specific sequences in the mandarin fishes 2022;561:738650.
73. Zheng S, Wang X, Zhang S et al. Screening and characterization of sex-linked DNA markers and marker-assisted selection in the Southern catfish (*Silurus meridionalis*), Aquaculture 2020;517:734783.
74. Liu H, Pang M, Yu X et al. Sex-specific markers developed by next-generation sequencing confirmed an XX/XY sex determination system in bighead carp (*Hypophthalmichehys nobilis*) and silver carp (Hypophthalmichthys molitrix), DNA Res 2018;25:257-264.
75. Han C, Zhu Q, Lu H et al. Screening and characterization of sex-specific markers developed by a simple NGS method in mandarin fish (*Siniperca chuatsi*), Aquaculture 2020;527:735495.
76. Palaiokostas C, Bekaert M, Khan MG et al. Mapping and validation of the major sex-determining region in Nile tilapia (*Oreochromis niloticus* L.) using RAD sequencing 2013;8:e68389.
77. Wang L, Xie N, Shen Y et al. Constructing high-density genetic maps and developing sexing markers in northern snakehead (*Channa argus*) 2019;21:348-358.
78. Wu X, Zhao L, Fan Z et al. Screening and characterization of sex-linked DNA markers and marker-assisted selection in blue tilapia (*Oreochromis aureus*), Aquaculture 2021;530:735934.
79. Sedlazeck FJ, Rescheneder P, Smolka M et al. Accurate detection of complex structural variations using single molecule sequencing 2017.
80. Chen Y, Wang AY, Barkley CA et al. Deciphering the exact breakpoints of structural variations using

long sequencing reads with DeBreak, Nature Communications 2023;14:283.

81. Maki H. Origins of spontaneous mutations: specificity and directionality of base-substitution, frameshift, and sequence-substitution mutageneses, Annual review of genetics 2002;36:279-303.

82. Tian D, Wang Q, Zhang P et al. Single-nucleotide mutation rate increases close to insertions/deletions in eukaryotes, Nature 2008;455:105-108.

83. Cooper DN, Krawczak M. Mechanisms of insertional mutagenesis in human genes causing genetic disease, Human genetics 1991;87:409-415.

84. Krawczak M, Cooper DN. Gene deletions causing human genetic disease: mechanisms of mutagenesis and the role of the local DNA sequence environment, Human genetics 1991;86:425-441.

85. DN CJHG. Mechanisms of insertional mutagenesis in human genes causing genetic disease 1991;87:409-415.

86. Krawczak M, Cooper DNJHg. Gene deletions causing human genetic disease: mechanisms of mutagenesis and the role of the local DNA sequence environment 1991;86:425-441.

87. Berger J, Suzuki T, Senti K-A et al. Genetic mapping with SNP markers in Drosophila 2001;29:475-481.

88. Wicks SR, Yeh RT, Gish WR et al. Rapid gene mapping in *Caenorhabditis elegans* using a high density polymorphism map 2001;28:160-164.

89. Cao J, Schneeberger K, Ossowski S et al. Whole-genome sequencing of multiple *Arabidopsis thaliana* populations, Nature genetics 2011;43:956-963.

90. Porubsky D, Eichler EE. A 25-year odyssey of genomic technology advances and structural variant discovery, Cell 2024.

91. Ebert P, Audano PA, Zhu Q et al. Haplotype-resolved diverse human genomes and integrated analysis of structural variation, Science 2021;372:eabf7117.

92. Liao W-W, Asri M, Ebler J et al. A draft human pangenome reference, Nature 2023;617:312-324.

93. Porubsky D, Harvey WT, Rozanski AN et al. Inversion polymorphism in a complete human genome assembly, Genome Biol 2023;24:100.

94. Porubsky D, Höps W, Ashraf H et al. Recurrent inversion polymorphisms in humans associate with genetic instability and genomic disorders, Cell 2022;185:1986-2005. e1926.

95. Liu YH, Luo C, Golding SG et al. Tradeoffs in alignment and assembly-based methods for structural variant detection with long-read sequencing data, Nature Communications 2024;15:2447.

96. Yang N, Liu J, Gao Q et al. Genome assembly of a tropical maize inbred line provides insights into structural variation and crop improvement, Nature genetics 2019;51:1052-1059.

97. Goto H, Peng L, Makova KD. Evolution of X-degenerate Y chromosome genes in greater apes: conservation of gene content in human and gorilla, but not chimpanzee, Journal of molecular evolution 2009;68:134-144.

98. Gross JB, Wilkens H. Albinism in phylogenetically and geographically distinct populations of *Astyanax cavefish* arises through the same loss-of-function Oca2 allele, Heredity 2013;111:122-130.

99. Jones FC, Grabherr MG, Chan YF et al. The genomic basis of adaptive evolution in threespine sticklebacks, Nature 2012;484:55-61.

100. Schmutz S, Berryere T. Genes affecting coat colour and pattern in domestic dogs: a review, Animal genetics 2007;38:539-549.

101. Tozaki T, Ohnuma A, Kikuchi M et al. Short insertion and deletion discoveries via whole-genome sequencing of 101 Thoroughbred racehorses, Genes 2023;14:638.

102. Sudmant PH, Rausch T, Gardner EJ et al. An integrated map of structural variation in 2,504 human

genomes, Nature 2015;526:75-81.

103. Zhou B, He Y, Chen Y et al. Comparative Genomic Analysis Identifies Great–Ape–Specific Structural Variants and Their Evolutionary Relevance, Molecular Biology and Evolution 2023;40:msad184.

104. Prychitko TM, Moore WSJMB, Evolution. Alignment and phylogenetic analysis of β-fibrinogen intron 7 sequences among avian orders reveal conserved regions within the intron 2003;20:762-771.

105. Alonge M, Wang X, Benoit M et al. Major impacts of widespread structural variation on gene expression and crop improvement in tomato, Cell 2020;182:145-161. e123.

106. Vialle RA, de Paiva Lopes K, Bennett DA et al. Integrating whole-genome sequencing with multi-omic data reveals the impact of structural variants on gene regulation in the human brain, Nature neuroscience 2022;25:504-514.

107. Fulton Lucinda A. MER, Wilson Richard K. Sequence and comparative analysis of the chicken genome provide unique perspectives on vertebrate evolution, Nature 2004;432:695-716.

108. Razaq A, Khan A, Shah ST et al. Association of insertion/deletion polymorphism of ace gene with essential hypertension in patients of Khyber Pakhtunkhwa, Pakistan Journal of Medical Sciences 2024;40:461.

109. Xiao Y, Xiao Z, Ma Y et al. Datasets and markers development workflow run scripts for high-throughput exploitation of genetic sex markers, figshare 2023.

 Click here to access/download;Figure;Figure Legends.docx

Figure 1: High-throughput exploitation process of genetic sex markers for O. fasciatus.

Figure 2: Distribution statistics of insertion and deletion fragment (＞100bp) positions in genomic functional regions of *O. fasciatus*.

(a) Distribution statistics of insertion variant loci using the female fish genome as a reference. (b) Distribution statistics of insertion variant loci utilizing the male fish genome as a point of reference. (c) Distribution statistics of deletion variant length using the female fish genome as a reference. (d) Distribution statistics of deletion variant loci using the male fish genome as a reference.

Note: The colors red, green, yellow, and orange represented the UTR, CDS, intron, and intergenic regions. The frequency of insertions (a, b) was quantified based on the number of events, while deletions (c, d) were characterized by their length in nucleotides.

Figure 3: The covariance of genomic sequence differences between the male and female genomes of *O. fasciatus* and distribution statistics of variant sites.

(a) The female genome (reference) of *O. fasciatus* vs. the male genome for homology comparison using the minimap2 software with a 5000 bp sliding window. The female genome was the vertical axis, and female chromosomes 8 and 10 were homologous to male chromosome 9. (b) The comparison of homology between the male genome of *O. fasciatus* and the female genome was conducted using the minimap2 software with a 5000 bp sliding window. The male genome was represented on the vertical axis, revealing homology between male chromosome 9 and female chromosomes 8 and 10. (c) Distribution statistics of variant loci using the female fish genome as a reference. Insertions (>100 bp) were summarized by number of events. Deletions (>100 bp) were summarized by length in nucleotides. From outer to inner circles were the chromosome name, positional distribution of insertion sites, base length distribution of deletion sites, and base substitution distribution, respectively. (d) Distribution statistics of variant loci using the male fish genome as a reference. (e) - (f) Dual-valued histograms corresponding to the Table 3 showed the number of insertion/deletion sites per chromosome with reference to the female (4,422 inertion sites, 4,675 deletion sites) and male (4,615 inertion sites, 4,385 deletion sites) genomes, respectively, where chromosome 9 in males was homologous to chromosomes 8 and 10 in females. Insertion sites were represented by negative values, and deletion sites were represented by positive values.

Figure 4: Intersection results of two variant site scanning methods and ranking of variant sites with long read depth frequency distributions

(a) Venn results of deletion locus data were obtained based on TBtools and SyRI software using the female fish genome as a reference, and 1,791 out of 2,116 deletion loci obtained based on SyRI software were shared deletion variant loci. (b) Utilizing the male fish genome as a point of reference for acquiring deletion site data in the context of Venn analysis, it was found that 1,749 out of the 2,045 deletion sites identified through the use of the SyRI software were common deletion variant sites. (c) Depth distribution of CLR reads in male fish using all female genomic deletion locus (＞100bp) information as a reference, with the area where high-quality male and female genetic marker information was located before the orange labeled trough position (~5.4). (d) The depth distribution of CLR reads in females was analyzed by utilizing male genomic deletion sites as a reference (>100bp), focusing on the region preceding the orange labeled trough position (~12.5) where high-quality male and female genetic marker information was present. (e) - (f) Depth distribution of CLR reads in males and females based on intersecting male and female genomic deletion site information (>100bp), respectively.

Figure 5: Representative genetic sex marker (insertion deletion variant sites) located in the intergenic region (IR), Intronic region (IN) of the *O. fasciatus* genome (STOSOd393e).

(a) Location of male and female sex markers in the male (MChr9) and female (FChr8) genomes. (b) Male and female sex genetic markers were located at loci in the intergenic region, using the female fish genome as a reference (Figure S2). Illumina and CLR clean data of bam comparison to the genome for insertion/deletion site validity detection. Red represented length long data from PacBio and gray represented was Illumina clean data. The e-PCR amplified bands were 390bp and 989bp in size, corresponding to DNA insertion and deletion patterns and agarose gel electrophoresis for identification of male and female genetically different populations. (c) By utilizing the female genome as a point of comparison, the genetic markers for male and female sexes were identified at specific insertion sites within *nuf2* gene introns (Figure S3, Figure S4). The resulting e-PCR amplified bands, measuring 205bp and 749bp in size, were indicative of distinct DNA insertion patterns and were utilized in agarose gel electrophoresis for the purpose of distinguishing between male and female genetically

differentiated groups. Males exhibited two amplified bands, while females exhibited only one. (d) By utilizing the female fish genome as a point of comparison, the genetic markers for male and female sexes were situated within the deletion sites located in the introns of genes (Figure S5, Figure S6). The amplified bands produced through e-PCR were observed to be 558bp and 928bp in length, aligning with the DNA deletion patterns and agarose gel electrophoresis maps utilized for distinguishing genetically distinct groups of males and females. Specifically, two bands were amplified in males, while only one band was observed in females.

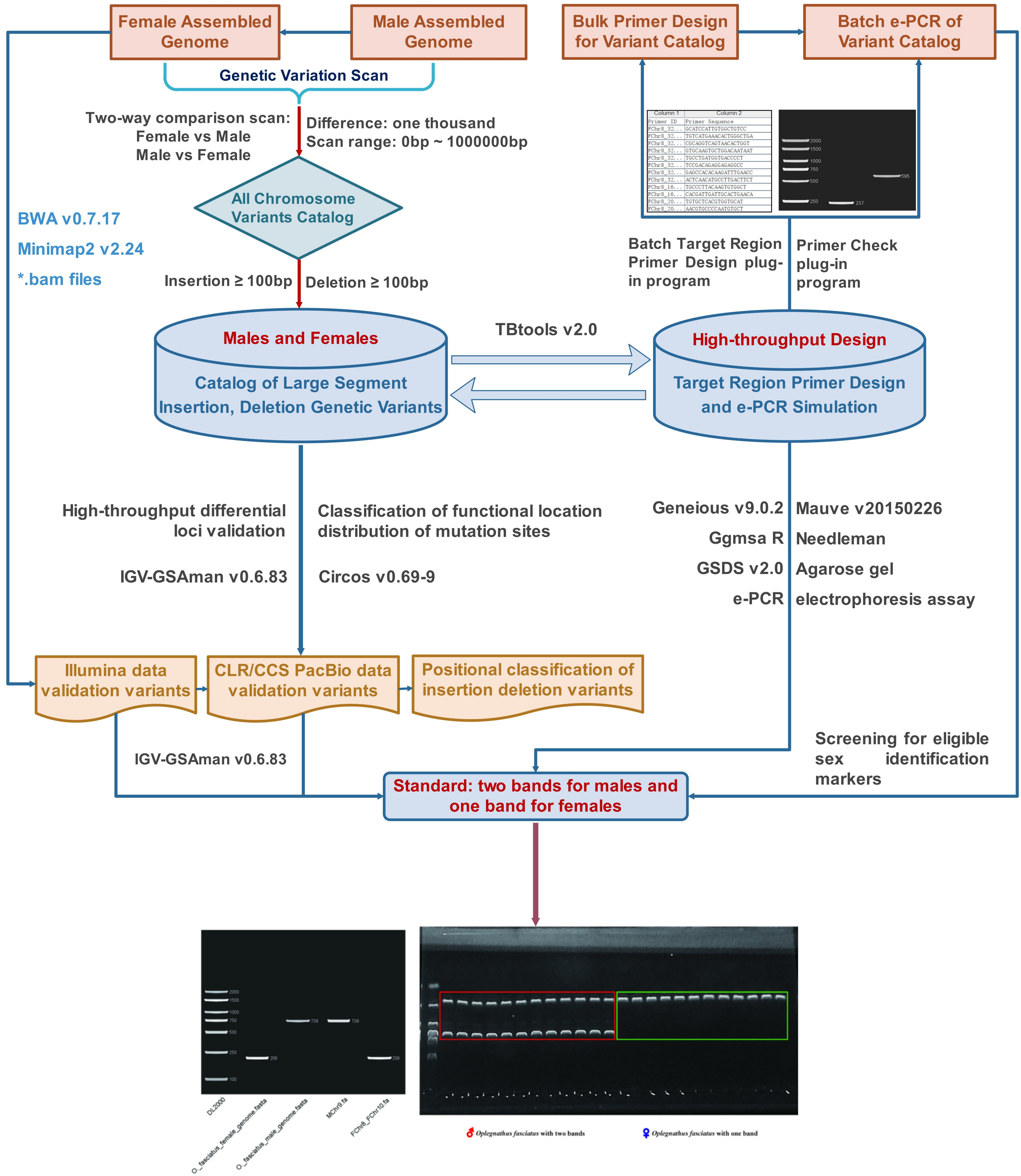

Female Assembled Genome
Male Assembled Genome
Bulk Primer Design for Variant Catalog
Batch e-PCR of Variant Catalog
Genetic Variation Scan
Two-way comparison scan:
Female vs Male
Male vs Female
Difference: one thousand
Scan range: 0bp ~ 1000000bp
All Chromosome Variants Catalog
BWA v0.7.17
Minimap2 v2.24
*.bam files
Insertion ≥ 100bp
Deletion ≥ 100bp
Batch Target Region Primer Design plug-in program
Primer Check plug-in program
Males and Females
Catalog of Large Segment Insertion, Deletion Genetic Variants
TBtools v2.0
High-throughput Design
Target Region Primer Design and e-PCR Simulation
High-throughput differential loci validation
Classification of functional location distribution of mutation sites
IGV-GSAman v0.6.83
Circos v0.69-9
Geneious v9.0.2
Mauve v20150226
Ggmsa R
Needleman
GSDS v2.0
Agarose gel
e-PCR
electrophoresis assay
Illumina data validation variants
CLR/CCS PacBio data validation variants
Positional classification of insertion deletion variants
IGV-GSAman v0.6.83
Screening for eligible sex identification markers
Standard: two bands for males and one band for females
DL2000
O._fasciatus_female_genome.fasta
O._fasciatus_male_genome.fasta
MChr9.fa
FChr8_FChr10.fa
♂ Oplegnathus fasciatus with two bands
♀ Oplegnathus fasciatus with one band

Click here to access/download;Figure;Figure 2.pdf

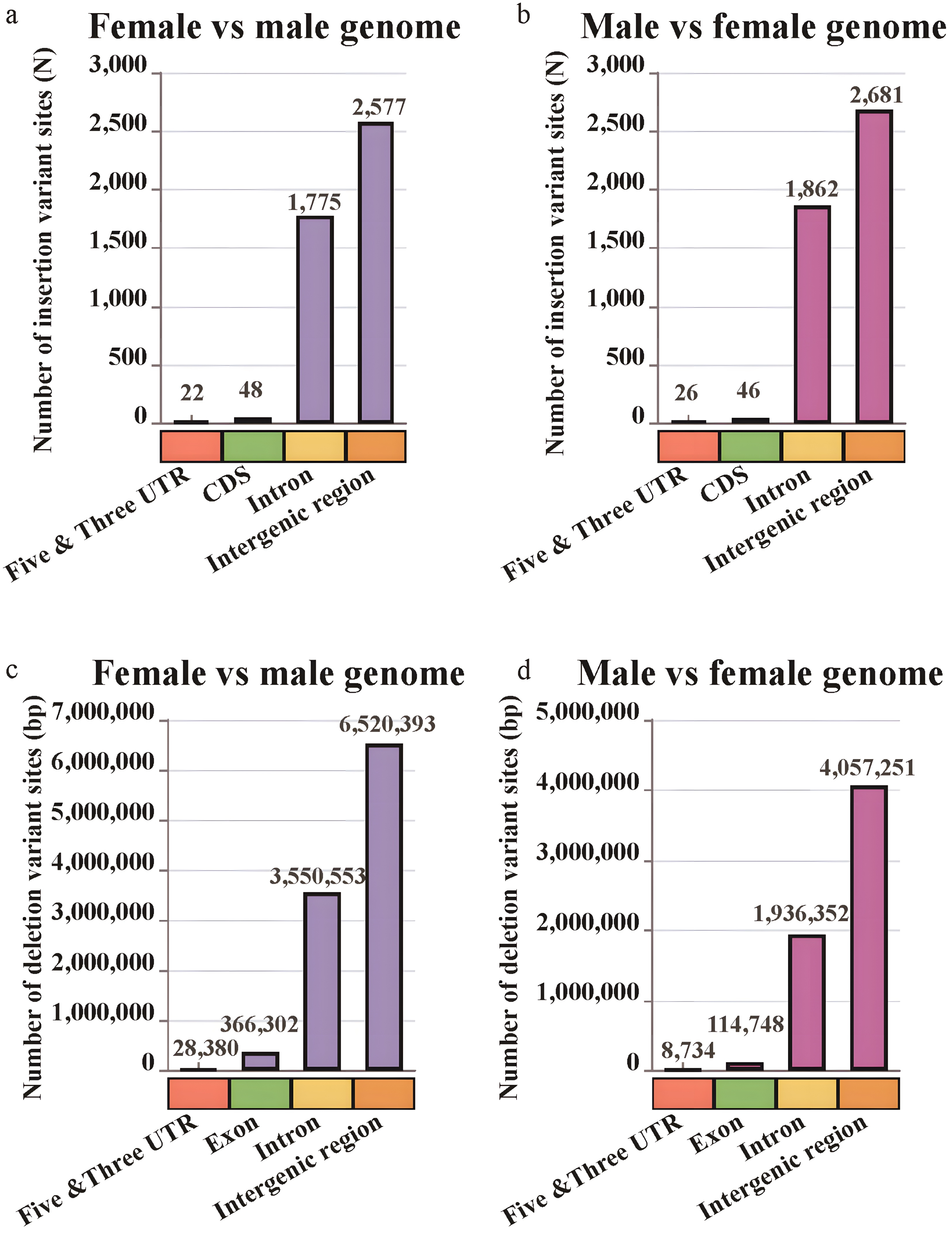

Figure3

Click here to access/download;Figure;Figure 3.pdf

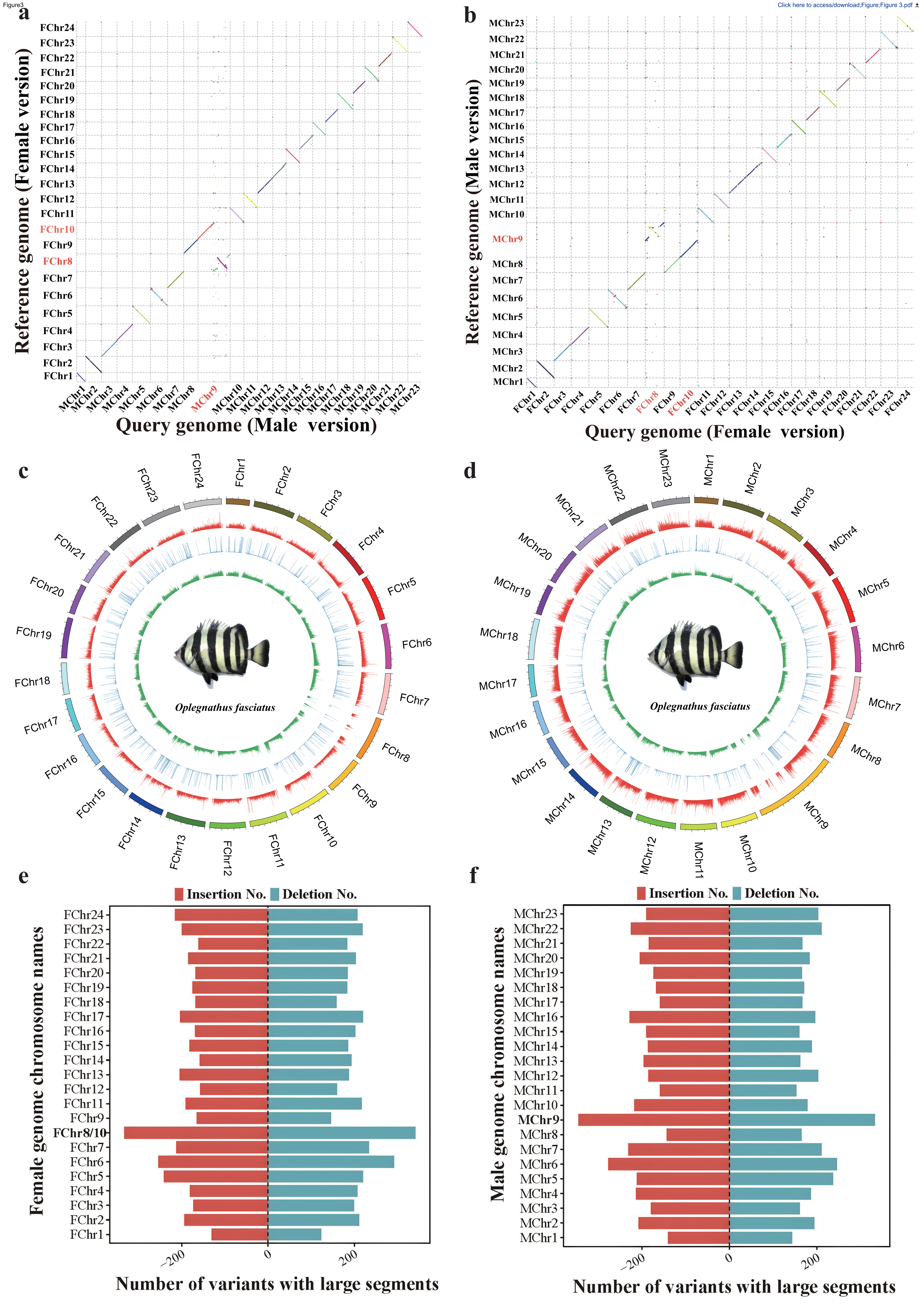

Figure4

Click here to access/download;Figure;Figure 4.pdf

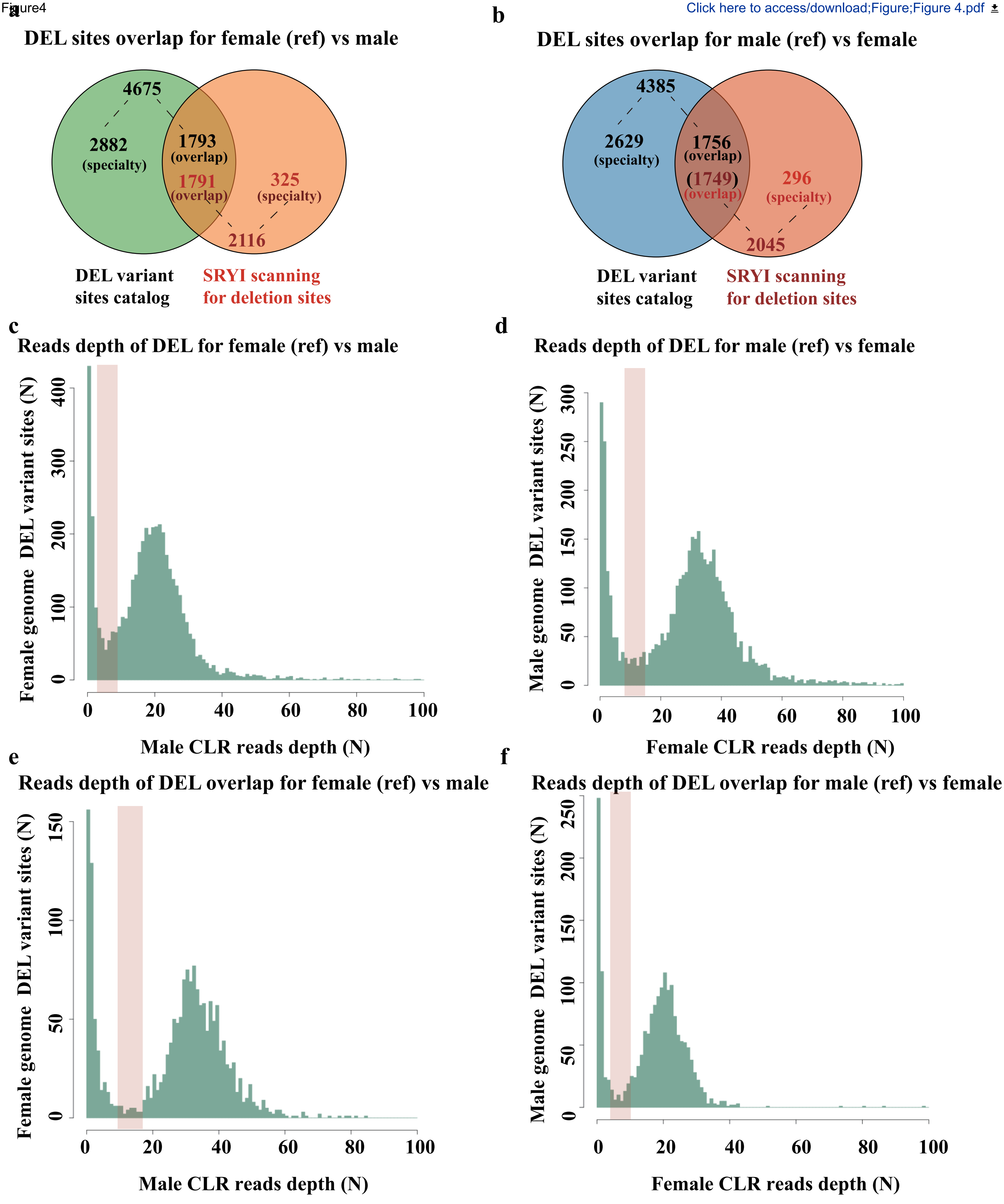

Figure5

Click here to access/download;Figure;Figure 5.pdf

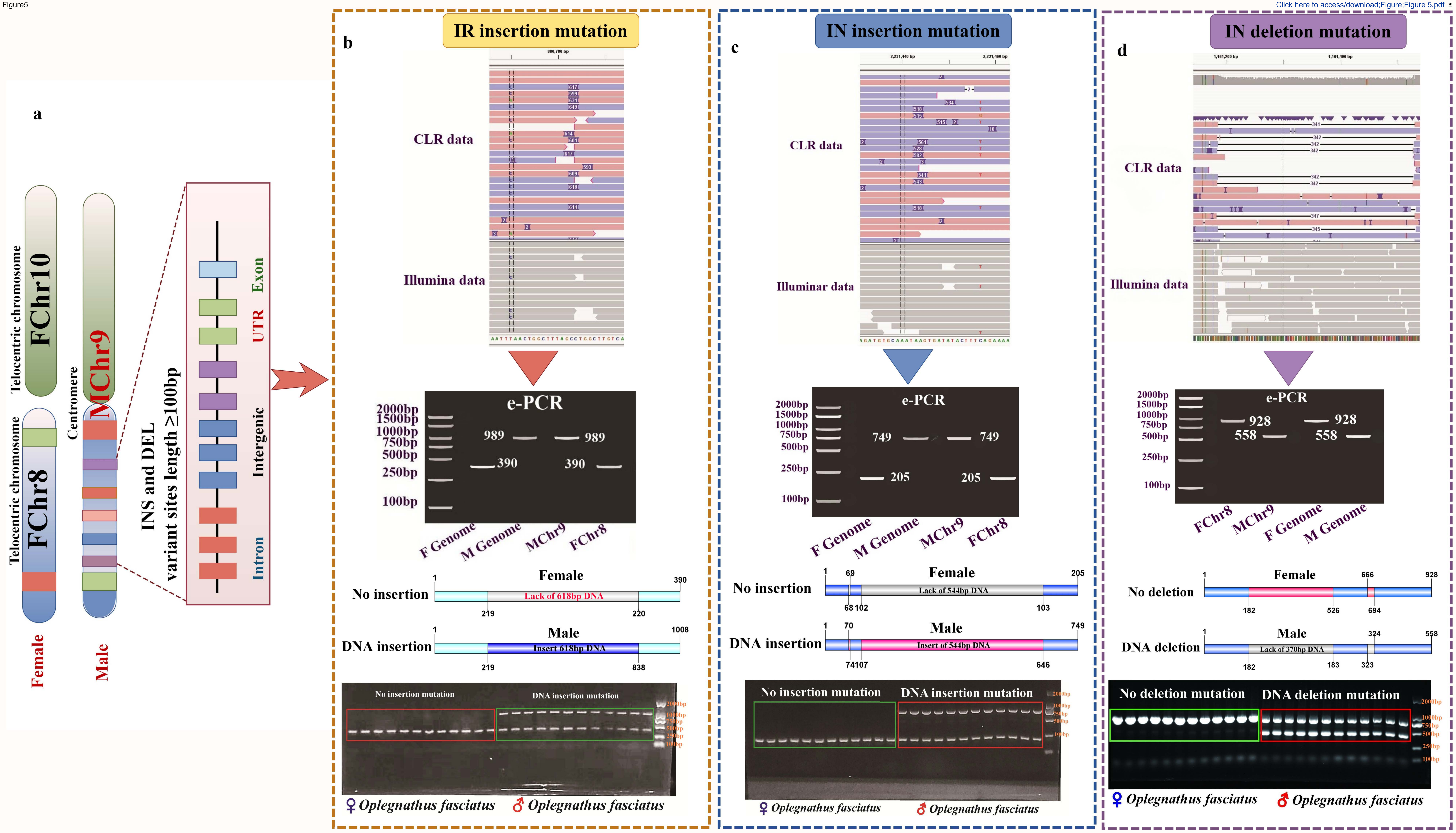

FigureS1
Click here to access/download;Figure;Figure S1.pdf

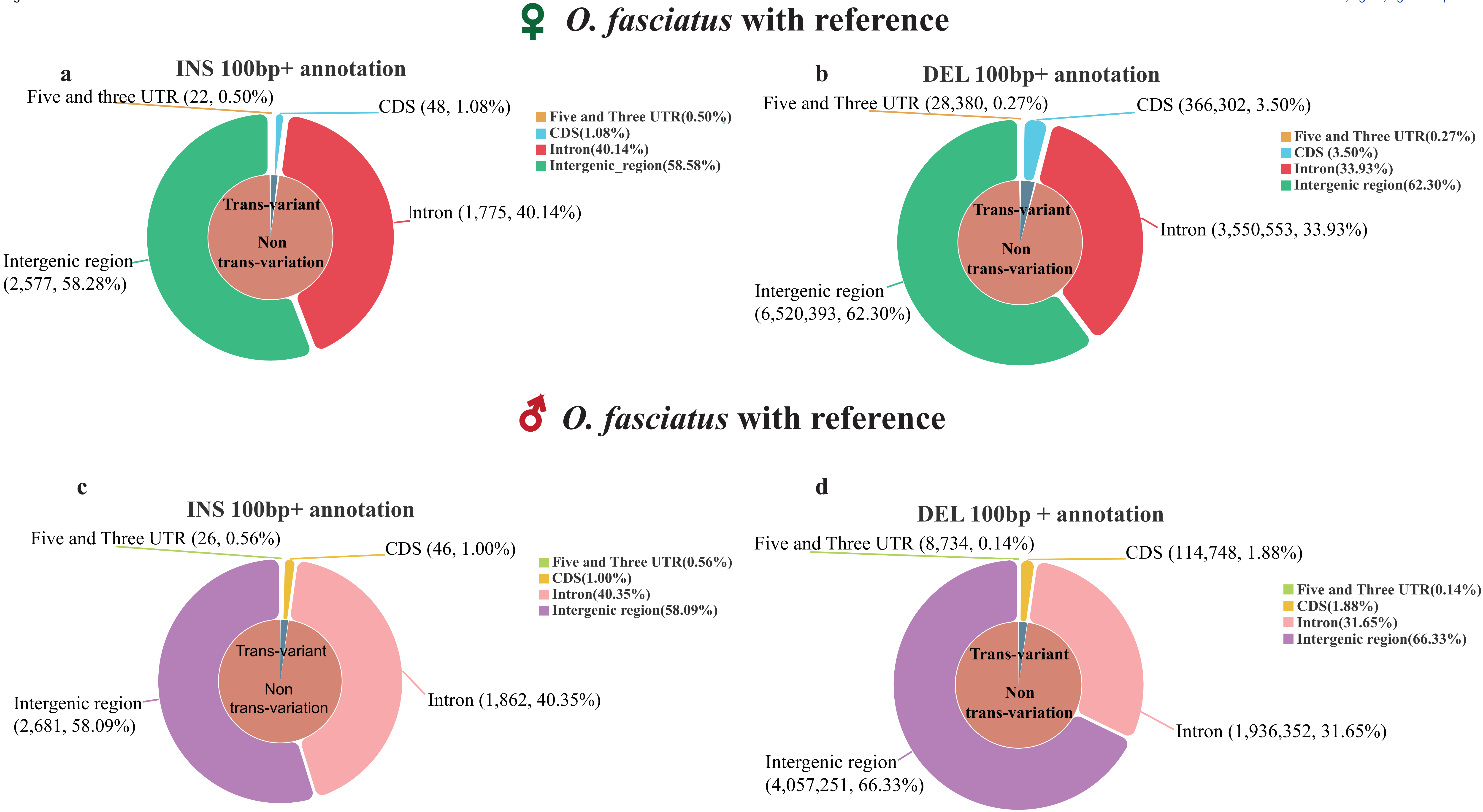

FigureS2

Click here to access/download;Figure;Figure S2.pdf

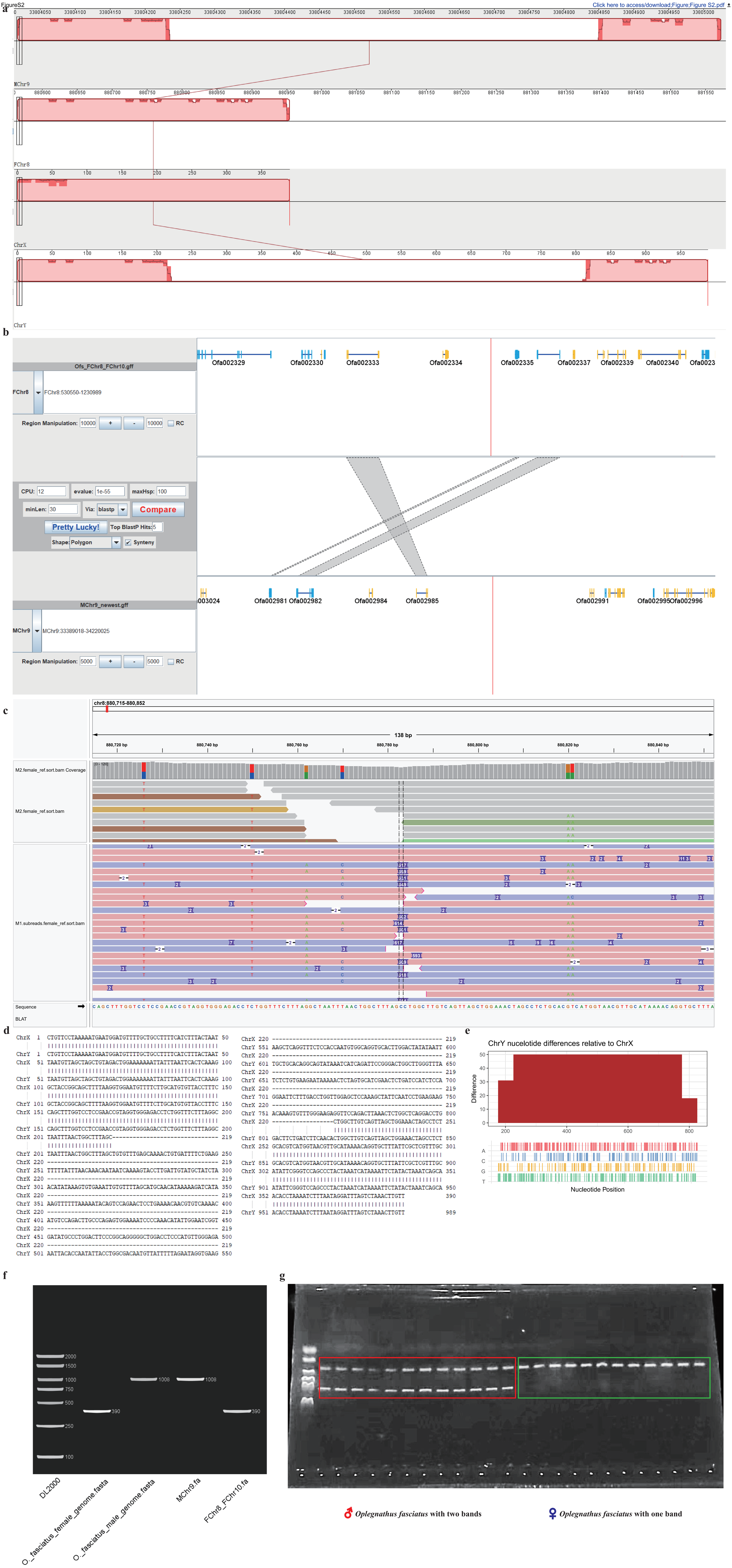

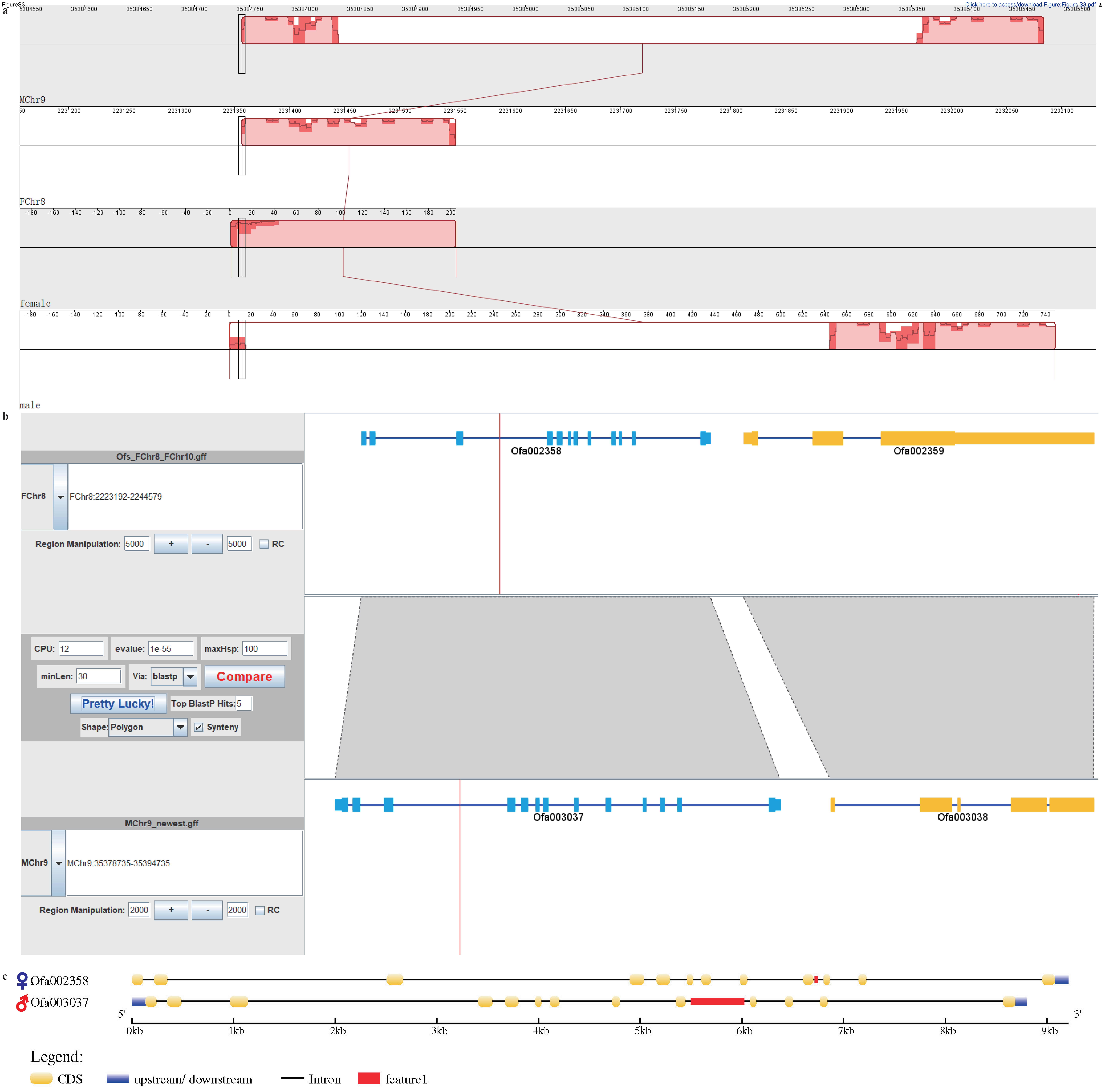
a
MChr9
FChr8
female
male
b
Ofs_FChr8_FChr10.gff
FChr8
FChr8:2223192-2244579
Region Manipulation: 5000 + - 5000 RC
CPU: 12
evalue: 1e-55
maxHsp: 100
minLen: 30
Via: blastp
Compare
Pretty Lucky!
Top BlastP Hits: 5
Shape: Polygon
Synteny
MChr9_newest.gff
MChr9
MChr9:35378735-35394735
Region Manipulation: 2000 + - 2000 RC
Ofa002358
Ofa002359
Ofa003037
Ofa003038
c
Ofa002358
Ofa003037
5'
3'
0kb
1kb
2kb
3kb
4kb
5kb
6kb
7kb
8kb
9kb
Legend:
CDS
upstream/ downstream
Intron
feature1

FigureS4

Click here to access/download;Figure;Figure S4.pdf

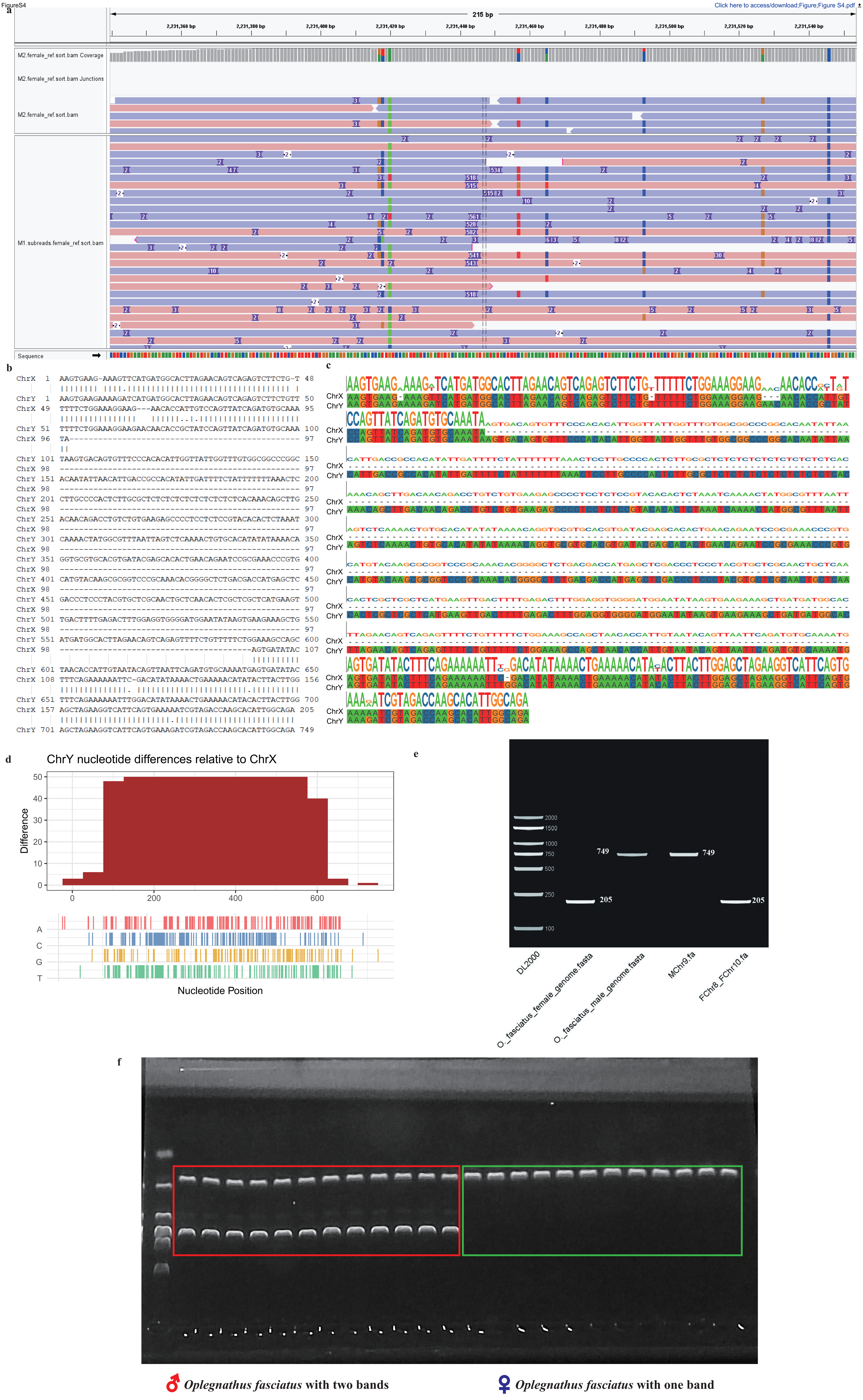

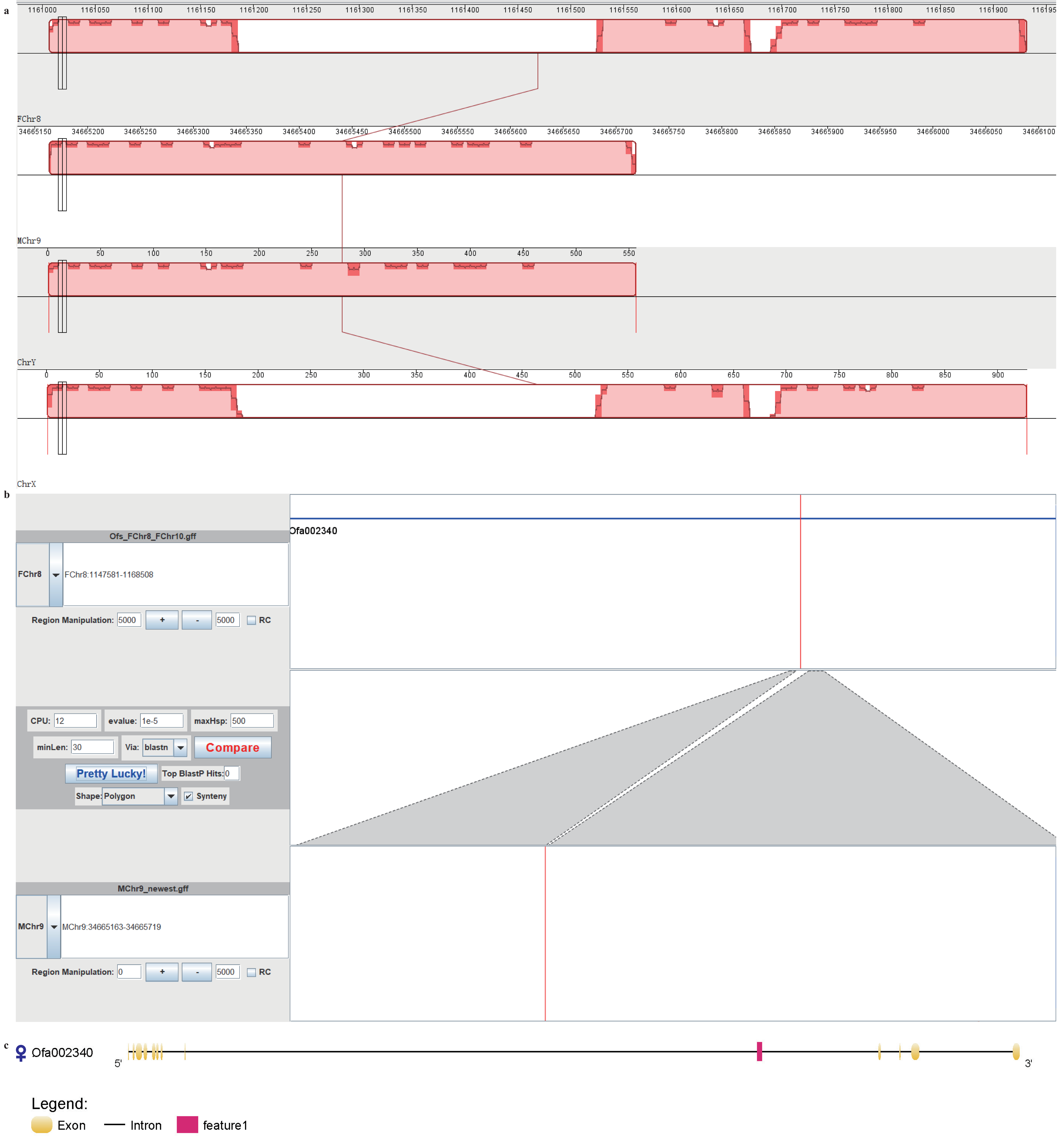
a
FChr8
MChr9
ChrY
ChrX
b
Ofs_FChr8_FChr10.gff
FChr8
FChr8:1147581-1168508
Region Manipulation: 5000 + - 5000 RC
CPU: 12
evalue: 1e-5
maxHsp: 500
minLen: 30
Via: blastn
Compare
Pretty Lucky!
Top BlastP Hits: 0
Shape: Polygon
Synteny
MChr9_newest.gff
MChr9
MChr9:34665163-34665719
Region Manipulation: 0 + - 5000 RC
Ofa002340
c
Ofa002340
5'
3'
Legend:
Exon
Intron
feature1

FigureS6
Click here to access/download;Figure;Figure S6.pdf

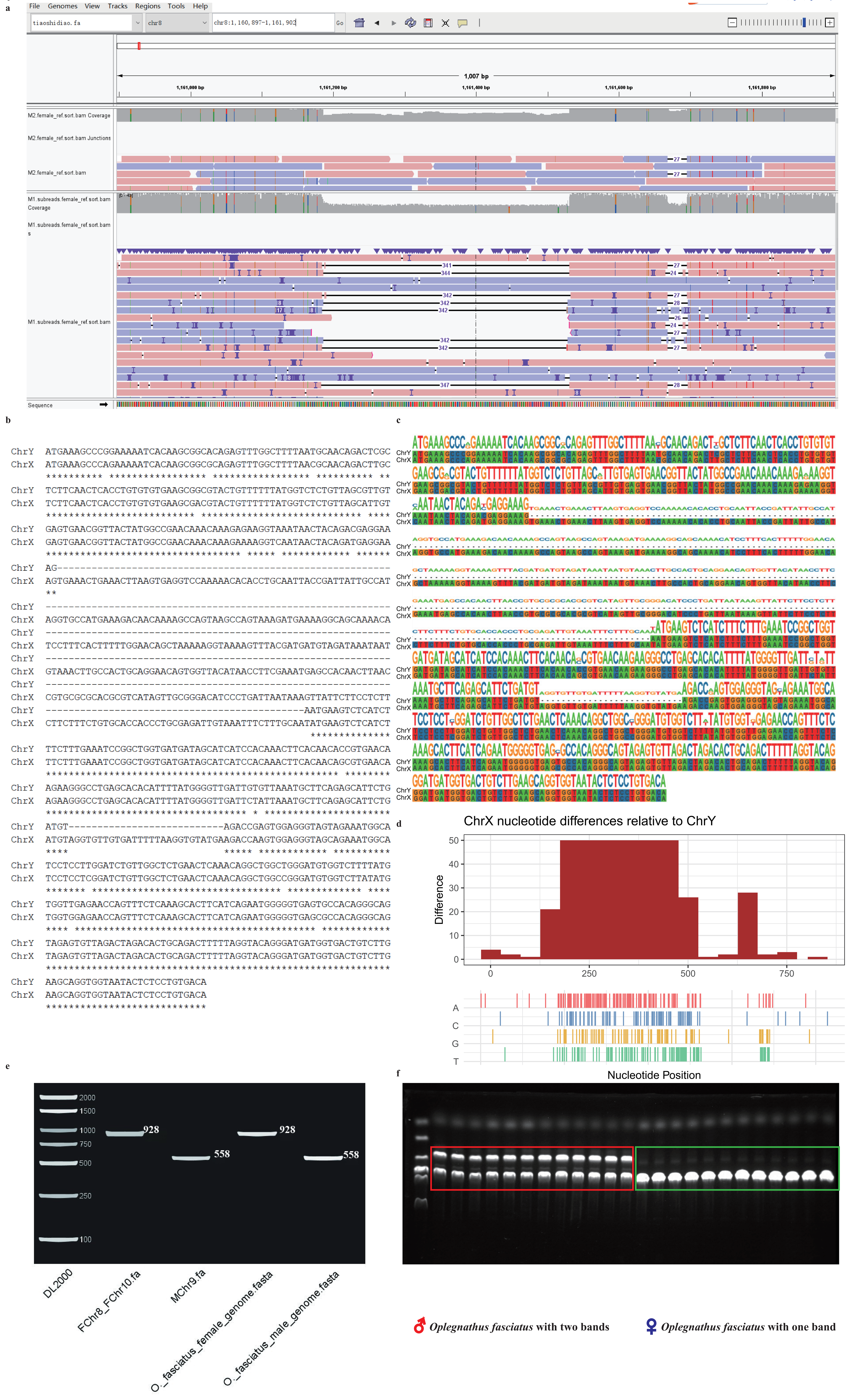

FigureS7

Click here to access/download;Figure;Figure S7.pdf

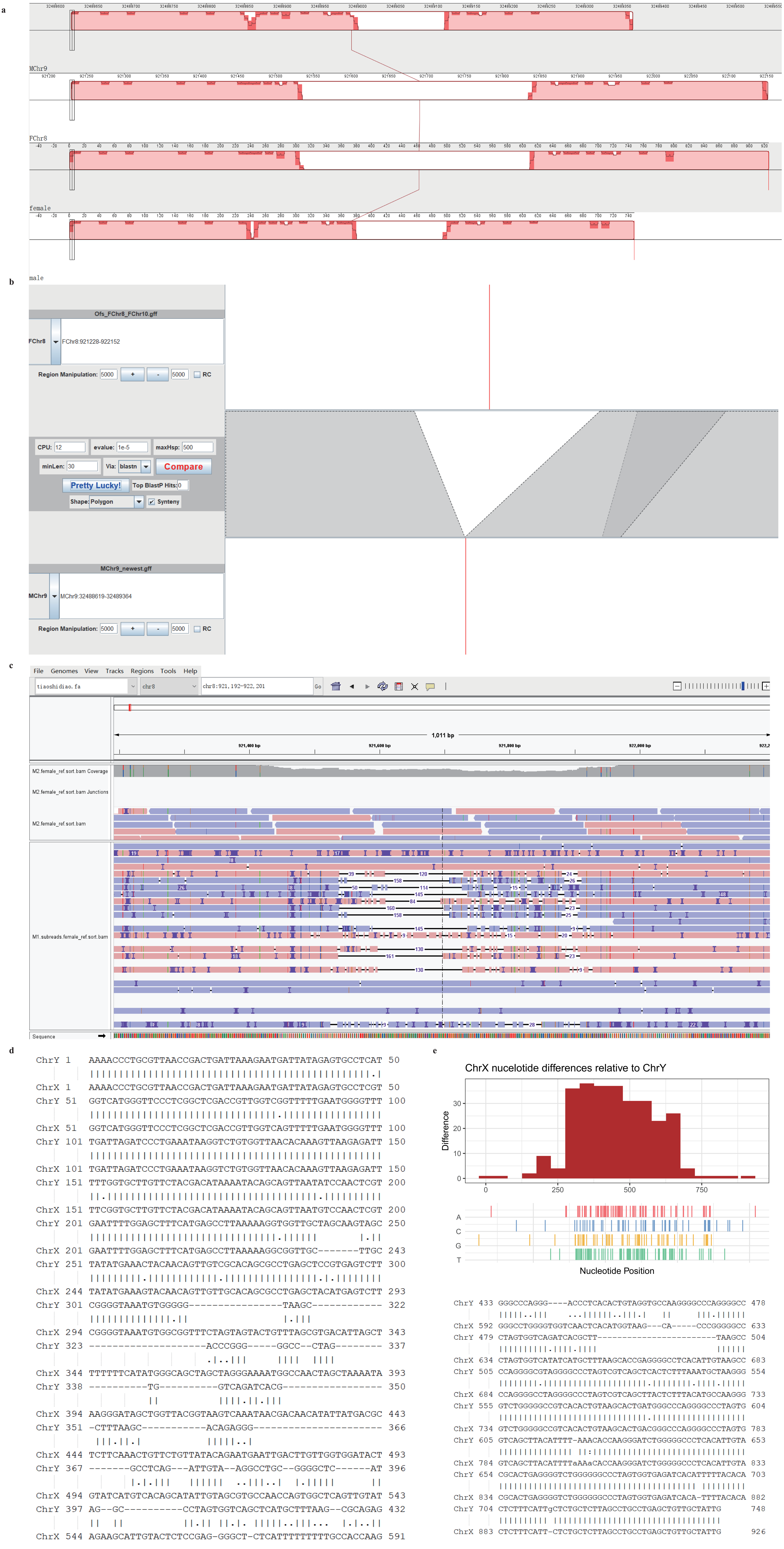

FigureS8

Click here to access/download;Figure;Figure S8.pdf

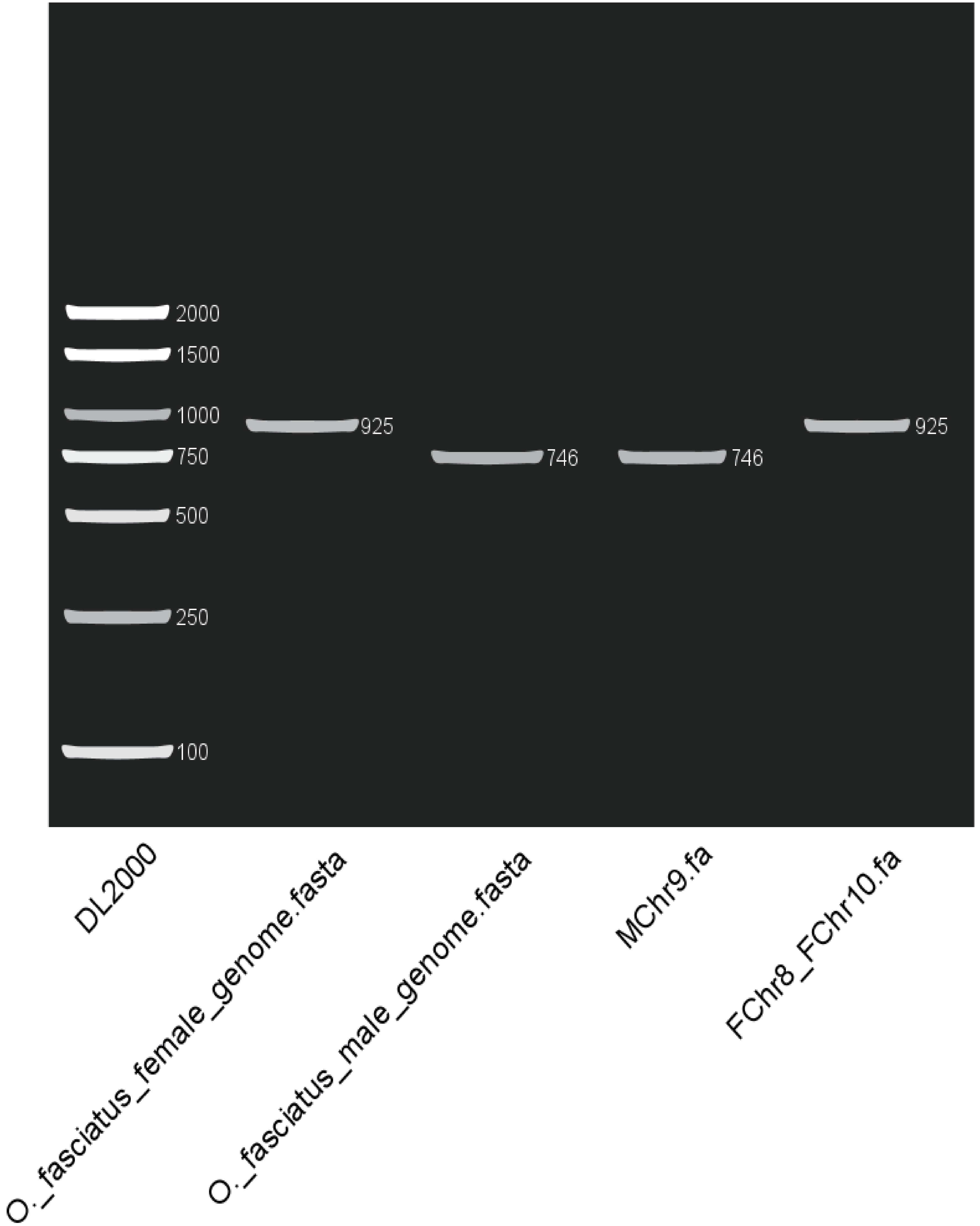

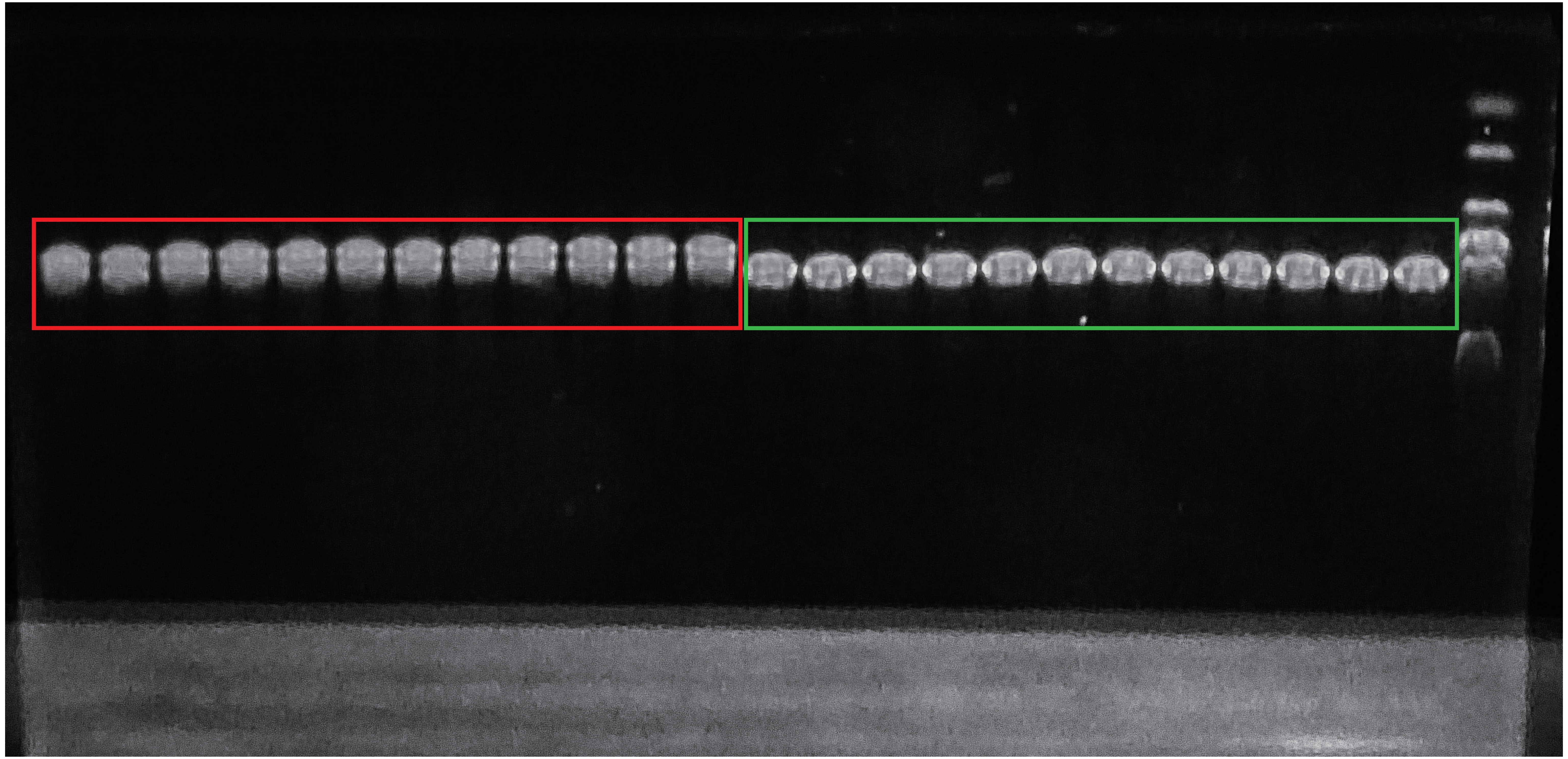

♂ *Oplegnathus fasciatus* with two bands

♀ *Oplegnathus fasciatus* with one band

FigureS9

Click here to access/download;Figure;Figure S9.pdf

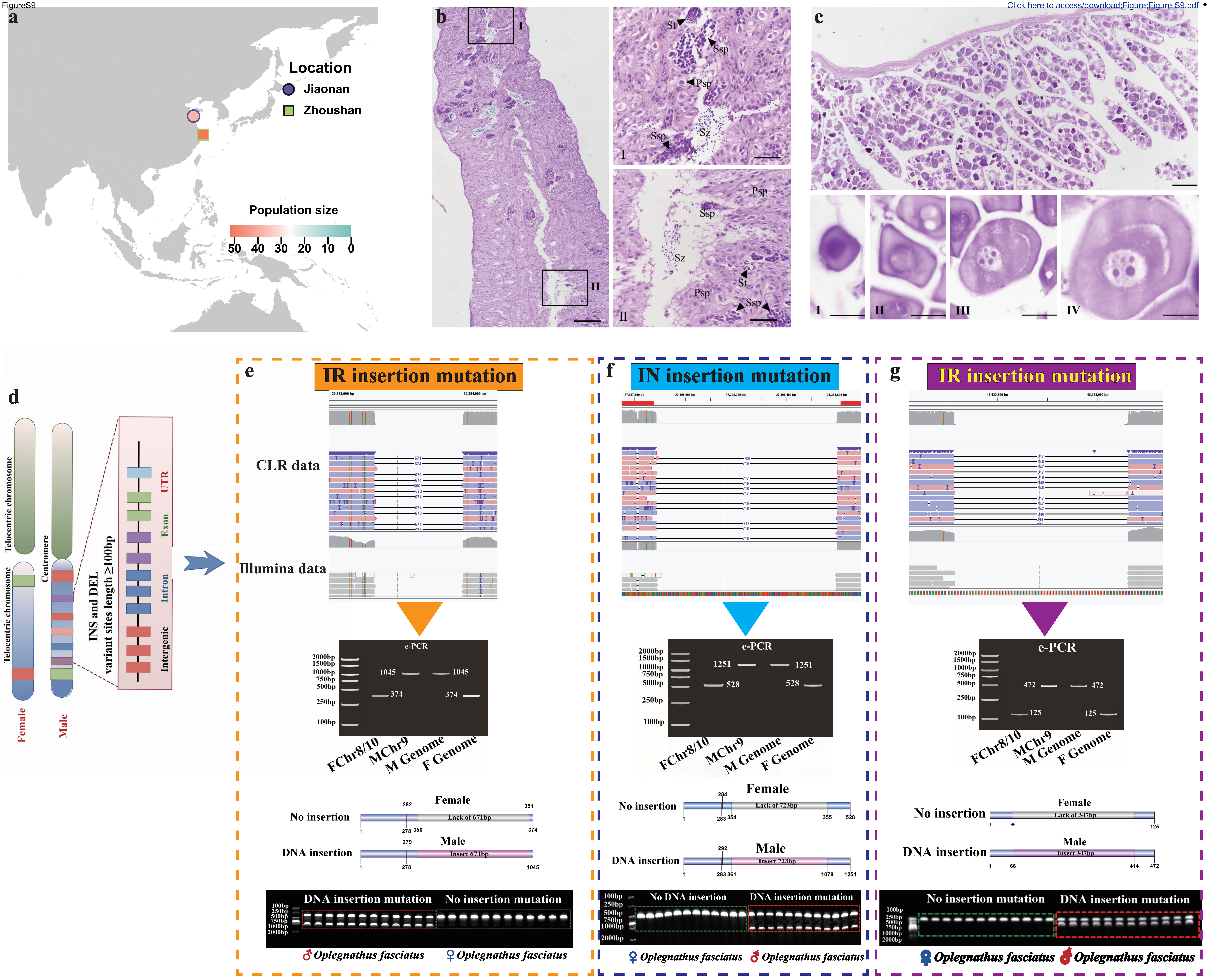

Click here to access/download
Supplementary Material
Supplementary information.docx

Click here to access/download
Supplementary Material
new-Female_reference_all_variation_Set.xls

Click here to access/download
Supplementary Material
new-Male_reference_all_variation_Set.xls

Click here to access/download
Supplementary Material
new-Female_reference_ INS_DEL 100_Set.xlsx

Click here to access/download
Supplementary Material
new-Male_reference_ INS_DEL 100_Set.xlsx

Click here to access/download
Supplementary Material
new-Female_reference_ INS_DEL 100_ExonSet_annotation.xlsx

Click here to access/download
Supplementary Material
new-Male_reference_ INS_DEL 100_ExonSet_annotation.xlsx

Click here to access/download
Supplementary Material
SyRI_Female_Reference_male_Get_all_DEL_100bp.xlsx

Click here to access/download
Supplementary Material
SyRI_Male_Reference_female_Get_all_DEL_100bp.xlsx

Female_reference_Male_DEL_100p.ann.with_CLR_reads_depth

Click here to access/download
Supplementary Material
Female_reference_Male_DEL_100p.ann.with_CLR_reads_depth.xls

Male_reference_Female_DEL_100p.ann.depthwith_CLR_reads_de
pth

Click here to access/download

Supplementary Material

Male_reference_Female_DEL_100p.ann.depthwith_CLR
_reads_depth.xls

Click here to access/download
Supplementary Material
Female_reference_Male_DEL_repeat.txt

Click here to access/download
Supplementary Material
Female_reference_Male_DEL_repeattype.txt

Male_reference_Female_DEL_repeat

Click here to access/download
Supplementary Material
Male_reference_Female_DEL_repeat.txt

Click here to access/download
Supplementary Material
Male_reference_Female_DEL_repeattype.txt

Click here to access/download
Supplementary Material
new-Female_Male_INSD_EL100_PrimerSet.xlsx
