# Supplementary material for: Innovative approach for high-throughput exploiting sex-specific markers in Japanese parrotfish Oplegnathus fasciatus
Source: Gigascience. 2024 Jul 19;13:giae045. doi: 10.1093/gigascience/giae045 (PMC11258905; doi:10.1093/gigascience/giae045)
Supplement: giae045_GIGA-D-23-00373_Revision_2 [file giae045_giga-d-23-00373_revision_2.pdf]

GigaScience

Innovative approach for high-throughput exploiting sex-specific markers in Japanese parrotfish Oplegnathus fasciatus

--Manuscript Draft--

**Manuscript Number:**	GIGA-D-23-00373R2
**Full Title:**	Innovative approach for high-throughput exploiting sex-specific markers in Japanese parrotfish Oplegnathus fasciatus
**Article Type:**	Research
**Funding Information:**	National Outstanding Youth Science Fund Project of National Natural Science Foundation of China (No. 42276107; No. 32270472) — Prof Yongshuang Xiao
**Abstract:**	Background<br>The use of sex-specific molecular markers has become a prominent method in enhancing fish production and economic value, as well as providing a foundation for understanding the complex molecular mechanisms involved in fish sex determination. Over the past decades, research on male and female sex identification has predominantly employed molecular biology methodologies such as RFLP (Restriction Fragment Length Polymorphism), RAPD (Random Amplification of Polymorphic DNA), SSR (Simple Sequence Repeat), and AFLP (Amplified Fragment Length Polymorphism). The emergence of high-throughput sequencing technologies, particularly Illumina, has led to the utilization of SNP and InDel variants as significant molecular markers for investigating sex identification in fish. The advancement of sex-controlled breeding encounters numerous challenges, including the inefficiency of current methods, intricate experimental protocols, high costs of development, elevated rates of false positives, marker instability, and cumbersome field testing procedures. Nevertheless, the emergence and swift progress of PacBio high-throughput sequencing technology, characterized by its long read output capabilities, offers novel opportunities to overcome these obstacles.<br>Findings<br>Utilizing male-female assembled genome information in conjunction with short-read sequencing data survey and long-read PacBio sequencing data, a catalog of large-segment (>100 bp) insertion-deletion genetic variants was generated through a genome-wide variant site scanning approach with bidirectional comparisons. The sequence tagging sites (STS) were ranked based on the long-read depth of the insertion deletion site, with markers exhibiting lower long read depth being considered more effective for large segment deletion variants. Subsequently, a catalog of bulk primers and simulated PCR for the male-female variant loci was developed, incorporating primer design for the target region and e-PCR technology. The Japanese parrotfish (Oplegnathus fasciatus), belonging to the Oplegnathidae family within the Centrarchiformes order, holds significant economic value as a rocky reef fish indigenous to East Asia. The criteria for rapid identification of male and female differences in O. fasciatus were established through agarose gel electrophoresis, which revealed two amplified bands for males and one amplified band for females. A high-throughput identification catalog of sex-specific markers was then constructed using this method, resulting in the identification of 3,639 (2,786 INS/853 DEL, ♀ as reference) and 3,672 (2,876 INS/833 DEL, ♂ as reference) markers in conjunction with 1,021 and 894 high-quality genetic sex identification markers, respectively. Sixteen differential loci were randomly chosen from the catalog for validation, with 11 of them meeting the criteria for male-female distinctions. The implementation of cost-effective and efficient technological processes would facilitate the rapid advancement of genetic breeding through expediting the high-throughput development of sex genetic markers for various species.<br>Conclusions<br>Our study utilized assembled genome information from male and female individuals obtained from PacBio, in addition to data from short-read sequencing data survey and long-read PacBio sequencing data. We extensively employed genome-wide variant site scanning and identification, high-throughput primer design of target regions, and e-PCR batch amplification, along with statistical analysis and ranking of the long reads depth of the variant sites. Through this integrated approach, we successfully compiled

Powered by Editorial Manager® and ProduXion Manager® from Aries Systems Corporation

	a catalog of large insertion/deletion sites (>100 bp) in both male and female O. fasciatus.
**Corresponding Author:**	Yongshuang Xiao, phD<br>Institute of Oceanology Chinese Academy of Sciences<br>Qingdao, CHINA
**Corresponding Author Secondary Information:**	
**Corresponding Author's Institution:**	Institute of Oceanology Chinese Academy of Sciences
**Corresponding Author's Secondary Institution:**	
**First Author:**	shuang Yong Xiao
**First Author Secondary Information:**	
**Order of Authors:**	shuang Yong Xiao
	Yongshuang Xiao, phD
	Zhizhong Xiao
	Yuting Ma
	Haixia Zhao
	Yanduo Wu
	Jinwei Huang
	Pingrui Xu
	Lin Liu
	Jing Liu
	Jun Li
**Order of Authors Secondary Information:**	
**Response to Reviewers:**	Reviewer reports:<br>Reviewer #1: The revised manuscript is significantly improved and the authors have done a great job addressing all my previous comments. I appreciate authors responses and revisions to all points.<br>Reply :<br>Thanks for your previous comments on our manuscript, which have been very helpful in improving the quality of our manuscript.<br><br>Reviewer #2: Dear colleagues,<br><br>Thank you very much for the thorough, extensive, cautious and precise replies to the previous round of comments that I provide, as well as that of the two other reviewers in my opinion.<br>I just have a list of minor comments to provide.<br><br>Best regards,<br><br>Being a non-native English speaker, please double-check my comments below.<br><br>Minor comments:<br>In the first paragraph of the new version (starting line 125), the use of the present tense was better than that of the actual paste tense (also line 195, the use of "was" would mean that the species no longer exists in this geographical area). L228, it is still the same sex chromosome system. Line 683, etc. Same for the Conclusion final paragraph, the present tense should be used, or the present perfect, but not the preterit.<br>Reply :<br>Thank you for your detailed and professional comments. We have put the manuscript

in the present tense where needed. Please see the labelled version.
Watch out the terminology used: "sex research (line 126)" deal with other fields of research (see for example the content of the "journal of sex research") and you might want to use a more specific wording, example given:
sexual reproduction, sex chromosomes, sex determinism and differentiation, sexual dimorphism, genetics/genomics of sex...
Reply :
Thanks for your professional advice, what we want to convey is that sex determination research, and we have made this place clear and descriptive. At the same time, we have made similar changes throughout the text. We revised “sex research” as “sex determination research”.
L246: Table 1":" (is missing) and put the number in an easy format (e.g. align right, number display)
Reply :
Thanks for your detailed comments. We have added the notation at the right of the Table 1.
L288: number display homogeneity. (an line 228 page 157
Reply :
We have taken these numbers to show homogeneity as follows: “(diff: oneInThousand, VarRange: 0 ~ 1,000,000 bp, BatchSize: 500 bp, Min Align Length for Cov Calc 10,000 bp, Min Align Length for Var Calling: 50,000 bp)”.
Why not using parrotfish instead of O. fasciatus along the text, tables and figures?
Reply :
Thanks to your professional comments, we have changed the species names to English throughout the text, except for the first occurrence where we have used Latin names. Please check the labelled version.
L765: PrEvious
Reply :
Thanks for your detailed comments. We have revised the “PrEvious” as “Previous”.
Line 244 page 157: Two consecutive blank spaces
Reply :
Thanks for your detailed comments. We replaced two consecutive spaces with a single space, and at the same time we searched the whole text and modified the double-space phenomenon in a similar way. Please check the labelled version.
Try not to use scientific names and vernacular ones in the same sentence (e.g. Drosophila melanogaster, Caenorhabditis elegans, mouse, human, and Arabidopsis thaliana)
Reply :
Thanks to your professional comments, we have standardised the names of species with scientific names in a single sentence as follows: “genomic genetic polymorphisms in various organisms, including Drosophila melanogaster, Caenorhabditis elegans, Mus musculus castaneus, Homo sapiens, and Arabidopsis thaliana”.
again, check homogeneity of occurrence of empty character between a value and its unit (eg line 768: 40Mb with no space, as other values has one.
Also line 590 and 591: a 965-kb deletion and a 4.8 Mb deletion, please homogenize.
Reply :
Thanks for your detailed comments. We have standardised the criteria, with uniform spaces after numbers and units, and have made changes throughout the text. Please see the labelled version.
Watch out that transposons are only class II (DNA) transposable elements. I guess that the authors were talking about Transposable Elements (TEs)
Reply :
Thanks to your professional comments. It is indeed transposon elements that are mentioned in our MS. And we changed transposons to "transposable elements". Please see the labelled version.

Reviewer #3: I thank the authors for their revision. Especially the inclusion of the actual genome assemblies this work is based on is important.

Below I return to my comments on the previous version:
1. Thank you for the addition. One comment: in the Sample collection, please rephrase line 191: HQ DNA was not extracted by Illumina/PacBio, but analyzed?
Reply :
Thanks for your detailed comments. Sorry for the confusion due to the mis-expression.

Powered by Editorial Manager® and ProduXion Manager® from Aries Systems Corporation

We meant to extract high-quality genomic DNA for high-throughput sequencing on the PacBio and Illumina platforms. We revised “High-quality genomic DNA was extracted from fresh muscle tissue and blood samples from pairs of male and female Japanese parrotfish using the Illumina platform (Illumina Inc., San Diego, CA, USA) and PacBio Sequel (Pacific Biosciences of California, Menlo Park, CA, USA) [45, 46]” as “High-quality genomic DNA was extracted from fresh muscle tissue and blood samples from pairs of male and female Japanese parrotfish using for high-throughput sequencing on the Illumina platform (Illumina Inc., San Diego, CA, USA) and PacBio Sequel (Pacific Biosciences of California, Menlo Park, CA, USA) [45, 46]”

2-3. Thank you for updating. I would suggest summarizing a bit more in the discussion, as it is very lengthy and some information is presented twice. Line 543: I would replace 'genomic structural variation' with just 'genomic variation', as you include SNPs under this definition.

Reply:

Thanks to your professional comments. We have deleted and modified redundancies and repetitions in the discussion by careful contextual comparison as follows:

1)Line 485 -486: We deleted “The majority of fish exhibited limited sex chromosome differentiation, which posed challenges in identifying them based on chromosome morphology [10]”.

2)Line 499-502: We deleted “However, the advancement of sex-controlled breeding has faced limitations due to various factors, such as low throughput of the above methods, cumbersome experimental procedures, expensive development cost, high rate of false positives, unstable markers, and inconvenient on-site testing.”.

3)Line 506-512: We revised the “In this study, O. fasciatus with the X1X1X2X2/X1X2Y system served as the focal point [44-46, 48]. By utilizing the assembled genome information of male and female individuals acquired through PacBio sequencing, along with data from the short-read sequencing data and long-read PacBio sequencing data, we extensively employed genome-wide variant sites multiple scanning and identification, STS long reads depth evaluation and ranking, high-throughput design of primers for the target regions, and e-PCR batch amplification.” as “Our study focused on O. fasciatus with the X1X1X2X2/X1X2Y system [44-46, 48]. We used the assembled genome information of male and female individuals, along with short-read and long-read data, to identify variant sites and evaluate read depth. We also designed primers for target regions and performed batch amplification using e-PCR (Figure 1, Figure 2, Figure 3, Table 2, Table 3).”

4)Line 512-517: We deleted “By employing a comprehensive approach involving genome-wide variant site scanning, high-throughput primer design within the desired region, and e-PCR batch amplification and validation methodologies, we successfully exploited a catalog encompassing insertion/deletion loci in large segments (>100 bp) for both male and female O. fasciatus”.

5)Line 546-549: We deleted “However, larger genomic structural variants, which encompassed genetic variations not fully captured by SNPs and InDels, are closely linked to biological phenotypes and might significantly influence phenotypic traits, disease susceptibility, and adaptive capacity [79].”.

4/6. I still think it is an odd choice to present deletions and insertions in different units.

Reply：

Thank you for your professional opinion. As you pointed out in your previous comments, insertions are summarised by the number of events, while deletions are summarised by nucleotide length, as we have explained in our previous presentation in the figure and table notes. We know that an insertion event is as a locus, which may exist in an intronic, exonic or intergenic region, and presents the character of a single functional region. However, deletions are different, as the size of deletion sequences varies, there is uncertainty in the functional regions of the genome, and a very large deletion site may span both intronic and exonic regions or even intergenic regions. In order to avoid the confusion of the statistics, we have determined through consultation that we use site counts for insertion events and sliding window counts for deletion sequences to count the number of deletions. Of course, we also carried out subsequent studies and validation of functional genomics of insertion deletion variants, and we will consider the statistics separately in the subsequent studies. Thank you again for your professional opinion.

5. What I meant is that the joint between chr F8 and F10 can be used to generate a PCR fragment in males, but not in females. This is not a major issue, it was just a suggestion.

Reply：

Powered by Editorial Manager® and ProduXion Manager® from Aries Systems Corporation

	Thank you for your professional opinion. As you mentioned, we also focused on the fusion region of the two chromosomes and performed a variant site scan, and we found that the fusion region had significant structural variation, but it was difficult for us to use it as a region for genetic sex marker development due to the inefficiency of amplification caused by the drastic variation. We found through variant site scanning that abundant variant sites also exist in the inverted region, which can be used as a stable region for sex marker exploitation.<br>Figure (Please check the Reply point-to-point)<br>7. Thank you for the explanation, PCR constraints are plausible. But that would be good to highlight in the text. And it does not explain the imbalance in Table 5? Any deletion in females should be an insertion in males, and vice versa? If it is detectable from one perspective, then why not from the other?<br>Reply：<br>Thanks for your professional comments. As you said any deletion in females should be an insertion in males. In conjunction with your concerns we have found through technical discussion that the number of loci for insertions is 4422 using the female as the reference male comparison, so in turn the number of deletions occurring when using the male as the reference female comparison should be close to 4422, which we have detected as 4385. The number of deletion sites is 4675 when using female as the reference male comparison, and conversely, the number of insertion sites is 4615 when using male as the reference female comparison, so that the number of insertion and deletion sites in our results are almost balanced under different reference groups. Meanwhile, we also explained the differences in the efficiency of PCR high-throughput amplification primer design for insertion and deletion sites in the paper as follows: "Variations in the efficacy of PCR high-pass primer design at insertion and deletion sites may be attributed to disparities in structural characteristics and GC content within the regions flanking the insertion/deletion variants.".<br>Table 5. Statistics of large-segment differential loci after primer design, e-PCR amplification, and simulated amplification products and size screening.<br>8. Fig 3e/f is clearer now.<br>Reply：<br>Thanks for your previous comments on our manuscript, which have been very helpful in improving the quality of our manuscript.<br><br>9. OK<br>Reply：<br>Thanks for your previous comments on our manuscript, which have been very helpful in improving the quality of our manuscript.<br><br>10/11. I think this figure still needs some work. The legend says that PacBio reads are in red, but I see both pink and blue (I think fwd/reverse? Also sort of suggests male/female). These IGV(ish) screenshots are not very clear, please explain a bit more. Why just the ~25 nt stretches? Are the dashed lines the deletions (I guess not)? The PacBio reads suggest a deletion in half the reads, which fits the story, but those dark blue scribbles are hard to see. Do we see entire reads, or just aligned portions?<br>Reply：<br>Thanks for your professional comments. We are very sorry for the confusion caused by our description. Our comparison strategy here is to use the female genome as a reference male for comparison. As we mentioned before the sex chromosome system of female fish is X1X1X2X2 and the sex chromosome system of male fish is X1X2Y, so there will be dimorphism in the high throughput sequenced reads of male fish, especially in the inverted region, and this phenomenon will be more obvious. Because PacBio's CLR sequencing method is used, there is no problem with bidirectional sequencing. However, because we used male and female fish with inverted structural variants, the phenomenon of matching direction was generated. We are sure that the pink and blue reads you see should be the reflection of the inversion of the male reads with the female fish as the reference. We have changed the content of CLR reads described in the figure to pink and blue to represent the same and opposite orientation of the female genome.<br>As you can see and as we provide the IGV insertion results plot below, since we have consolidated representative results in the manuscript, we have intercepted a portion of the sequence that contains the insertion deletion site and both ends in order to reflect the presence of the insertion and deletion events clearly. The dotted line you see is the base pair baseline, which appears as a single dotted line from a distance, and when

	zoomed in is two dotted lines corresponding to a single base. The blue scribble you see is actually the result of the size of the insertion fragment as shown below. In order to fully demonstrate this result of ours, we have changed the vertical orientation to horizontal.<br>Figure (Please check the Reply point-to-point)<br><br>Line 553: Prvious -> Previous<br>Reply :<br>Thanks for your detailed comments. We have revised the “PrEvious” as “Previous”.
**Additional Information:**	
**Question**	**Response**
Are you submitting this manuscript to a special series or article collection?	No
**Experimental design and statistics**<br><br>Full details of the experimental design and statistical methods used should be given in the Methods section, as detailed in our Minimum Standards Reporting Checklist. Information essential to interpreting the data presented should be made available in the figure legends.<br><br>Have you included all the information requested in your manuscript?	Yes
**Resources**<br><br>A description of all resources used, including antibodies, cell lines, animals and software tools, with enough information to allow them to be uniquely identified, should be included in the Methods section. Authors are strongly encouraged to cite Research Resource Identifiers (RRIDs) for antibodies, model organisms and tools, where possible.<br><br>Have you included the information requested as detailed in our Minimum Standards Reporting Checklist?	Yes
**Availability of data and materials**<br><br>All datasets and code on which the conclusions of the paper rely must be	Yes

either included in your submission or deposited in publicly available repositories (where available and ethically appropriate), referencing such data using a unique identifier in the references and in the "Availability of Data and Materials" section of your manuscript.<br><br>Have you have met the above requirement as detailed in our Minimum Standards Reporting Checklist?	

Powered by Editorial Manager® and ProduXion Manager® from Aries Systems Corporation

Manuscript

Click here to access/download;Manuscript;renamed_eaac9.docx

Innovative approach for high-throughput exploiting sex-specific markers in Japanese parrotfish *Oplegnathus fasciatus*

Yongshuang Xiao[1,2,3,4†], Zhizhong Xiao[1,2,3,4,6†], Lin Liu[5†], Yuting Ma[1,2,3,4], Haixia Zhao[1,2,3,4], Yanduo Wu[1,2,3,4], Jinwei Huang[1,2,3,4], Pingrui Xu[1,2,3,4], Jing Liu[1*], Jun Li[1,2,3,4*]

[1]Center for Ocean Mega-Science, Institute of Oceanology, Chinese Academy of Sciences, Qingdao, China， [2]CAS and Shandong Province Key Laboratory of Experimental Marine Biology, Institute of Oceanology, Chinese Academy of Sciences, Qingdao, China， [3]Key Laboratory of Breeding Biotechnology and Sustainable Aquaculture, Chinese Academy of Sciences, Qingdao, China， [4]Laboratory for Marine Biology and Biotechnology, Qingdao National Laboratory for Marine Science and Technology, Qingdao, China, [5]Wuhan Frasergen Bioinformatics Co., Ltd. East Lake High-Tech Zone, Wuhan, China, [6]Weihai Hao Huigan Marine Biotechnology Co, Weihai, China.

[†] Co-first authors

∗Correspondence address: Jing Liu, Institute of Oceanology, Chinese Academy of Sciences, 7 Nanhai Road, Qingdao, 266071, China; Tel: +86-053282898790; E-mail: jliu@qdio.ac.cn; Jun Li, Institute of Oceanology, Chinese Academy of Sciences, 7 Nanhai Road, Qingdao, 266071, China; Tel: +86-053282898718; E-mail: junli@qdio.ac.cn.

†Contributed equally to this work.

Yongshuang Xiao, ORCID: 0000-0002-1979-4555

Zhizhong Xiao, ORCID: 0000-0003-2403-1381

Lin Liu, ORCID: 0009-0001-7315-2529

Yuting Ma, ORCID: 0000-0002-7158-9364

Haixia Zhao, ORCID: 0009-0003-8305-6009

Yanduo Wu, ORCID: 0000-0003-0060-1685

Jinwei Huang, ORCID: 0009-0003-5238-8318

Jing Liu, ORCID: 0000-0001-8868-0386

Jun Li, ORCID: 0009-0005-2415-1895

Abstract

Background

The use of sex-specific molecular markers has become a prominent method in enhancing fish production and economic value, as well as providing a foundation for understanding the complex molecular mechanisms involved in fish sex determination. Over the past decades, research on male and female sex identification has predominantly employed molecular biology methodologies such as RFLP (Restriction Fragment Length Polymorphism), RAPD (Random Amplification of Polymorphic DNA), SSR (Simple Sequence Repeat), and AFLP (Amplified Fragment Length Polymorphism). The emergence of high-throughput sequencing technologies, particularly Illumina, has led to the utilization of SNP and InDel variants as significant molecular markers for investigating sex identification in fish. The advancement of sex-controlled breeding encounters numerous challenges, including the inefficiency of current methods, intricate experimental protocols, high costs of development, elevated rates of false positives, marker instability, and cumbersome field testing procedures. Nevertheless, the emergence and swift progress of PacBio high-throughput sequencing technology, characterized by its long read output capabilities, offers novel opportunities to overcome these obstacles.

Findings

Utilizing male-female assembled genome information in conjunction with short-read sequencing data survey and long-read PacBio sequencing data, a catalog of large-segment (>100 bp) insertion-deletion genetic variants was generated through a genome-wide variant site scanning approach with bidirectional comparisons. The sequence tagging sites (STS) were ranked based on the long-read depth of the insertion deletion site, with markers exhibiting lower long read depth being considered more effective for large segment deletion variants. Subsequently, a catalog of bulk primers and simulated PCR for the male-female variant loci was developed, incorporating primer design for the target region and e-PCR technology. The Japanese parrotfish (*Oplegnathus fasciatus*), belonging to the Oplegnathidae family within the Centrarchiformes order, holds significant economic value as a rocky reef fish indigenous to East Asia. The criteria for rapid identification of male and female differences in Japanese parrotfish were established through agarose gel electrophoresis, which revealed two amplified bands for males and one amplified band for females. A high-throughput identification catalog of sex-specific markers was then constructed using this method, resulting in the identification of 3,639 (2,786 INS/853 DEL, ♀ as reference) and 3,672 (2,876 INS/833

DEL, ♂ as reference) markers in conjunction with 1,021 and 894 high-quality genetic sex identification markers, respectively. Sixteen differential loci were randomly chosen from the catalog for validation, with 11 of them meeting the criteria for male-female distinctions. The implementation of cost-effective and efficient technological processes would facilitate the rapid advancement of genetic breeding through expediting the high-throughput development of sex genetic markers for various species.

Conclusions

Our study utilized assembled genome information from male and female individuals obtained from PacBio, in addition to data from short-read sequencing data survey and long-read PacBio sequencing data. We extensively employed genome-wide variant site scanning and identification, high-throughput primer design of target regions, and e-PCR batch amplification, along with statistical analysis and ranking of the long reads depth of the variant sites. Through this integrated approach, we successfully compiled a catalog of large insertion/deletion sites (>100 bp) in both male and female Japanese parrotfish.

***Keywords*:** large-segment insertion/deletion; bulk primers design; e-PCR technology; *Oplegnathus fasciatus*; high-throughput identification

Data description

Introduction

Sexual dimorphism is common in the animal kingdom, but the mechanisms of sex determination are diverse [1, 2]. Thus, sex determination research has always been a topic of interest in biology. Fish occupy a key position in the vertebrate evolutionary system, and because of their wide distribution, large number of species and diverse sex determination mechanisms, they are an important target for the study of sex determination mechanisms [3-9]. However, most fish have a low degree of differentiation between the sex chromosomes, which is difficult to detect from the morphology of the chromosomes [10]. The development of sex-specific molecular markers and sex-control biotechnology has provided an important technical way to increase fish production and economic value and has also laid the foundation for deciphering the molecular mechanism of fish sex determination [11]. Researchers have succeeded in developing sex-specific or correlated molecular markers in many fish species using a variety of techniques, including SSR, RAPD, AFLP, SNP and In Del [5, 6, 12-17]. However, these methods for obtaining sex-specific markers have many

problems, such as low throughput, single markers, cumbersome development and experimental procedures, high development costs, low amplification efficiency and inconvenient on-site detection. The first issue to be addressed was to establish a high-throughput and easy-to-detect marker procedure for sex-specific identification in the investigation of sex dimorphism, mechanisms of sex differentiation, and development and use of sex-producing traits.

In recent years, with the rapid development of genome sequencing technology and the significant decrease in the cost of sequencing, it has become possible to perform whole-genome sequencing of important cultured species. Rapid developments in next-generation sequencing (NGS) technologies have created significant opportunities for the discovery and use of species germplasm resources [4]. In 2002, the genome sequencing of *Takifugu rubripes* was successfully accomplished, marking it as the pioneering model fish species to undergo complete genome sequencing [18]. Subsequently, over the course of nearly 16 years, the whole-genome sequencing of more than 40 fish species, including *Oryzias latipes*, *Gasterosteus aculeatus*, *Pelteobagrus fulvidraco*, *Mola mola*, *Nothobranchius furzeri*, and *Salmo salar*, has been carried out on a national and international scale [19-30]. Over the span of approximately 16 years until 2018, the majority of fish genomes were sequenced utilizing second-generation sequencing technology, specifically Illumina [4, 18-21]. Despite the high throughput capabilities of second-generation sequencing, the relatively short sequencing read lengths pose challenges in obtaining precise genome maps and accurate information on chromosome structural variation. This limitation is especially pronounced in genomes with high complexity and repetitive regions.

At present, third-generation sequencing technology with high precision of long fragments represented by single-molecule real-time sequencing has matured, which includes Pacific Biosciences' (PacBio) single-molecule real-time sequencing technology (SMRT), Oxford Nanopore's single-molecule nanopore sequencing technology, and so on. Among them, PacBio RS sequencing technology is the most widely used and commercialized core third-generation sequencing technology and has been widely used in the genome assembly of several species, such as *Oropetium thomaeum* and *Lates calcarifer* [31-33]. The initial assembly of the human telomere-to-telomere (T2T) CHM13 genome, utilizing PacBio HiFi and Oxford Nanopore ultralong-read sequencing, achieved a contig N50 of 154.26 Mb, resulting in a seamless assembly that fills a crucial gap in the human DNA genetic code map [31, 34-36]. In

addition, Hi-C technology based on next-generation sequencing technology has gradually become a favourable tool for chromosome assembly [37]. With the rapid development of sequencing technology and the improvement of assembly technology, the genome integrity and accuracy have been greatly improved, especially in marine fishes, and the assembly now obtained Contig N50 up to the level of 28 Mb for *Sciaenops ocellatus* [38]. Thus, the development of next-generation sequencing technologies has made it possible to exploit high-throughput sex-specific markers in high-precision assembled genome data.

Oplegnathus fasciatus (Temminck & Schlegel, 1844, Fishbase ID: 1709; NCBI:txid881956; marinespecies.org:taxname:277892) is a rocky reef fish distributed in a wide range of shallow waters around Korea, Japan, China and Hawaii [39-42]. Japanese parrotfish has become an important fishery resource for offshore cage culture and fish stocking in marine ranches in China, Japan and Korea [41, 42]. Japanese parrotfish has been a commercially important species for sashimi production and recreational angling, with engineered aquaculture in China yielding ex-factory prices of up to USA$30/kg [42]. Meanwhile, Japanese parrotfish is characterized by a multiple $X_1X_1X_2X_2/X_1X_2Y$ sex chromosome system [43-46]. Sexual dimorphism has been observed in Japanese parrotfish, with males growing faster than females, especially at weights greater than 500 grams [7, 8, 45, 46]. Therefore, the breeding of new strains with a male predominance has become one of the goals for the future breeding of Japanese parrotfish. The development of appropriate genetic sex markers was a prerequisite for achieving a new variety of Japanese parrotfish with male predominance. Currently, available markers for the genetic characterization of Japanese parrotfish are extremely limited and have mainly been identified by AFLP, SSR and Illumina technologies, which constrains the process of exploiting new varieties of Japanese parrotfish [47-49]. The completion of the female and male genomes of Japanese parrotfish has enabled high-throughput development of large segments to visualize genetic sex markers from a genome-wide perspective.

In this work, a catalog of genomic differences between females and males has been constructed based on the assembled genome information of Japanese parrotfish by using bidirectional whole-genome sequence alignment [50, 51]. Using the difference catalogs, we obtain datasets of insertion and deletion variant sites larger than 100 bp by screening and use short-read sequencing data surveys from genome sequencing and long-read PacBio sequencing data to test the confidence of large segmental variant sites

[52]. Utilizing the Japanese parrotfish sequence tagged sites as a foundation, we employ e-simulation amplification technology to conduct a genome-wide PCR assay aimed at identifying single amplified-banded variant loci between female and male genomes [52]. Then, we perform PCR amplification and agarose gel electrophoresis for the mutant loci detected by simulated PCR. Since Japanese parrotfish exhibits an $X_1X_1X_2X_2/X_1X_2Y$ sex chromosome system, a single primer pair amplifies two bands in males and one band in females.

The paper has been organized as follows: First, a fast scanning method for female and male assembled genome variant loci is introduced, and an insertion/deletion dataset of large segments has been constructed. Using the constructed insertion/deletion dataset of large fragments, high-throughput candidate molecular marker loci for female and male genetic sex identification of Japanese parrotfish are identified. Then, the new technique of PCR electronic simulation with bulk amplification is used to perform simulated amplification of high-throughput candidate molecular marker loci to screen primers for the single amplification of the female and male genomes with amplification differences. Finally, a high-throughput precision marker dataset for male and female genetic sex identification is obtained and tested by agarose gel electrophoresis (Figure 1).

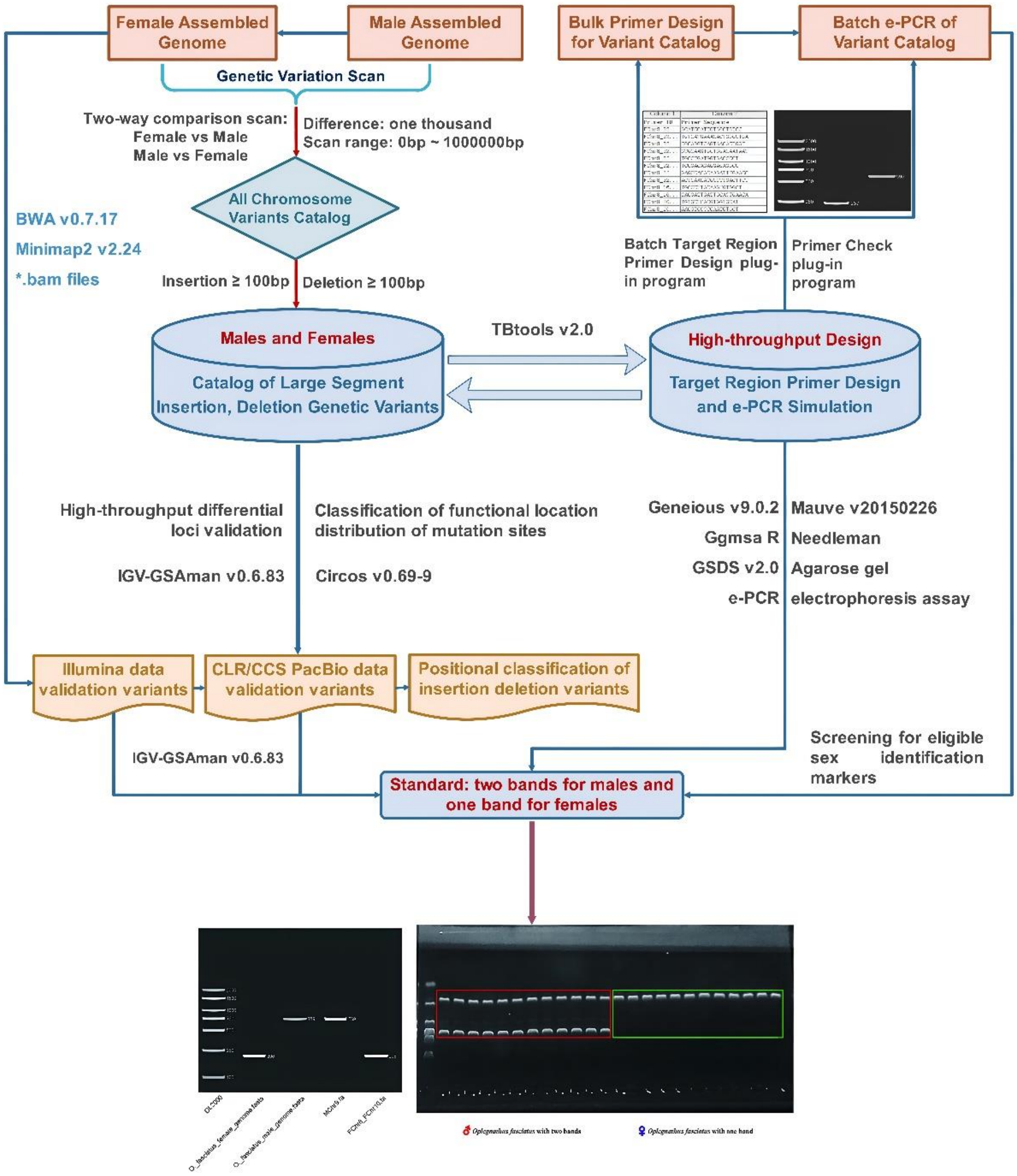

Figure 1: High-throughput exploitation process of genetic sex markers for Japanese parrotfish.

Table 1. Parameters for genome sequencing and assembly quality assessment of chromosome-level genomes of male and female Japanese parrotfish

Illumina platform (Short-read sequencing data survey)		
	Male Japanese parrotfish	Female Japanese parrotfish
Reads number	432,091,798	613,163,122
Raw data (bp)	63,910,932,339	90,702,298,813
Clean data (bp)	56,617,669,600	80,887,039,002
Read length (bp)	148, 147	148, 147

Q20 (%)	97.9, 97.3	98.0, 97.1
Q30 (%)	93.8, 92.0	94.1, 91.4
PacBio Sequel platform (Long-read PacBio sequencing data)		
	Male Japanese parrotfish	Female Japanese parrotfish
Polymerase read number	3,674,737	4,760,021
Polymerase read bases (bp)	39,844,228,109	62,877,786,278
Average polymerase read length (bp)	10,847	13,219
Polymerase read N50 (bp)	18,850	22,964
Subreads number	4,711,720	6,689,662
Total bases of subreads (bp)	39,793,172,442	62,783,888,050
Average subread length (bp)	8,448	9,397
Length of genome (bp)	762,267,613	768,808,243
Number of contigs	1,355	1,372
Contigs N50 (bp)	2,183,645	2,130,780
Number of Scaffolds	23	24
Scaffold N50 (bp)	32,431,321	33,548,962
Genome coverage (X)	251.1	314.6
Complete BUSCOs (%)	98.2	98.0

Note：male and female genomic quality parameters of Japanese parrotfish [45, 46].

Materials and Methods

Sample collection, sequencing and genome quality

Male and female Japanese parrotfish captured from Qingdao, Shandong Province (Yellow Sea) were utilized for genome sequencing and assembly. High-quality genomic DNA was extracted from fresh muscle tissue and blood samples from pairs of male and female Japanese parrotfish using for high-throughput sequencing on the Illumina platform (Illumina Inc., San Diego, CA, USA) and PacBio Sequel (Pacific Biosciences of California, Menlo Park, CA, USA) [45, 46]. Genomic DNA from fresh muscle tissue and blood samples of male and female Japanese parrotfish pairs was extracted to

construct short insert libraries (300 ~ 350 bp) for males and females, respectively, using standard protocols provided by the Illumina HiSeq X Ten platform (Illumina Inc., San Diego, CA, USA. RRID:SCR_016385). Raw reads totaling approximately 90.7 Gb and 63.9 Gb were generated. After removing splice-containing sequences, low-quality, and redundant reads, approximately 80.8 Gb and 56.6 Gb of pure data were obtained from the genomes of males and females, respectively, corresponding to Q20 averages of 97.55% and 97.60%, respectively (Table 1). Genomic DNA libraries (20 kb) for male and female fish were then constructed using the PacBio Sequel platform (Pacific Biosciences of California, Menlo Park, CA, USA) (Table 1). Sequencing was performed on the PacBio Sequel platform using the Sequel Binding Kit 2.0, Sequel Sequencing Kit 2.1, and Sequel SMRT Cell 1M v2. Seven and five SMRT Cells were used for sequencing the male and female genomes, respectively, with SMRT LINK 5.0 used to filter the raw data from the zero-mode waveguide. We obtained 62.8Gb and 39.8Gb of long read length PacBio sequencing data for female (accession SRP160016) and male (accession SRP220007) genome assembly, respectively [45, 46]. The female and male Japanese parrotfish genome were de novo assembled using Canu (RRID:SCR_015880) (v 1.4) with Corrected-Error-Rate set to 0.040, followed by redundancy removal using Redundans (v 0.13c) (minCoverage = 15), genome polishing with Arrow tool in SMRT Link 5.0, and final assembly refinement with Pilon (RRID:SCR_014731) using Illumina NGS short reads from the genome surveys. Based on the male and female draft genomes and Hi-C technology, we obtained high-quality genomes at the chromosome level of female (768.8 Mb, accession SUB14064808) and male (762.2 Mb, accession SUB14065793) Japanese parrotfish, and the contig N50 and scaffold N50 of the male and female fish genomes reached 2.13 Mb/2.18 Mb and 33.5 Mb/32.4 Mb, respectively [45, 46]. To assess the genomic completeness of assembled male and female Japanese parrotfish, we subjected the assembled sequences to BUSCO (RRID:SCR_015008) (v 5) evaluation (BUSCO, actinopterygii_odb10), with Complete BUSCOs assessed values up to 98.2% and 98.0% for male and female genomes, respectively (Table 1).

Large fragment variant loci scanning and identification

We employed the genome variant site scanning plugin with accompanying parameters (diff: oneInThousand, VarRange: 0 ~ 1,000,000 bp, BatchSize: 500 bp, Min Align Length for Cov Calc 10,000 bp, Min Align Length for Var Calling: 50,000 bp) in TBtools (RRID:SCR_023018) (v 2.0) software to conduct bidirectional comparisons of the complete genomic sequences of male and female organisms [52, 53]. Additionally, we established catalogs containing genomic disparities between males and females. To classify base substitution, insertion, and deletion sites, as well as to screen large insertion-deletion sites (>100 bp) against a differential catalogs, we utilized the Table Row Extract program. In order to further evaluate the stability and accuracy of the dataset containing large segment insertion deletion sites, we employed SyRI software (python3 syri_env/bin/syri -c out.filtered.coords -d out.filtered.delta -r FM.fasta -q genome.format.fa --nosnp) to autonomously detect variant sites (≥100 bp) in the assembled genomes of both male and female individuals, and subsequently compared these sites with our existing dataset of variant sites. Additionally, we utilized BWA (v 0.7.8) (bwa-0.7.17/bwa mem -t 6 -SP genome.fa M2_1P.fq.gz M2_2P.fq.gz | samtools (RRID:SCR_005227) view -bS > alignment.bam) and Minimap2 (v 2.24) (minimap2 –ax map-pb –t 8 genome.fasta M1.subreads.fasta > M1.subreads.sam) softwares to align the short-read sequencing data generated from Illumina platform and the long-read sequencing data obtained from PacBio platform to the male and female genomes, respectively, in order to validate the large segment variant sites [54, 55]. To further improve the exploitation efficiency of sex-specific markers based on long reads, we calculated the read depth of long reads of insertion deletion sites, and then ranked the insertion deletion site markers based on the depth of long reads, for large segment deletion variants, the lower the depth of long reads, the more effective the markers were, so we constructed a frequency map of the distribution of the depth of long reads of the large segment deletion sites of the male and female genomes, and then classified the markers. Subsequently, employing the Batch Target Region Primer Design plug-in and e-PCR plug-in in TBtools (v 2.0) software, we designed primers for high-throughput amplification and screened them for single amplification bands at the insertion and deletion sites specific to the male and female genomes [52]. To identify the origin of

large segment insertion deletion sites, we evaluated whether the variant sites in the dataset were transposable elements by integrating RepeatModeler (RRID:SCR_015027) (v 2.0.1) and RepeatMasker (RRID:SCR_012954) (v 4.1.2) softwares. The results of transposable elements determination obtained by the two softwares were combined and the duplicates were removed for all transposable elements identification results. At the same time, we utilized TRF (RRID:SCR_022193) (v 4.09.1) software to evaluate whether the variant sites in the dataset were tandem repeats.

The positional distribution of female and male genetic variant loci on the genome was depicted using Circos (RRID:SCR_011798) (v 0.69-9) software [56]. Homology comparison and visualization of insertion-deletion sites with male and female linear genomes were conducted using a combination of Geneious (RRID:SCR_010519) (v 9.0.2) software and Mauve program (RRID:SCR_012852) (v 20150226) [57, 58]. The comparison of genetic sex marker sequences with the Global Alignment standard was performed using the Needleman Wunsch plug-in in TBtools (v 2.0) [52]. Additionally, the visualization of genetic sex marker variant site regions was accomplished using Ggmsa program [59]. GSDS (v 2.0) software was utilized to illustrate the location of the variant site within the functional region of the gene [60]. A total of 24 tails from both male and female Japanese parrotfish specimens were collected and distinguished through gonadal histology. Simultaneously, we gathered 93 tails from wild population samples of Japanese parrotfish along the coasts of Zhejiang and Shandong Provinces, China, to verify the efficacy of sex markers across various geographic regions. The reliability of the genetic sex markers was assessed by randomly identifying male and female populations using e-PCR in conjunction with agarose gel electrophoresis.

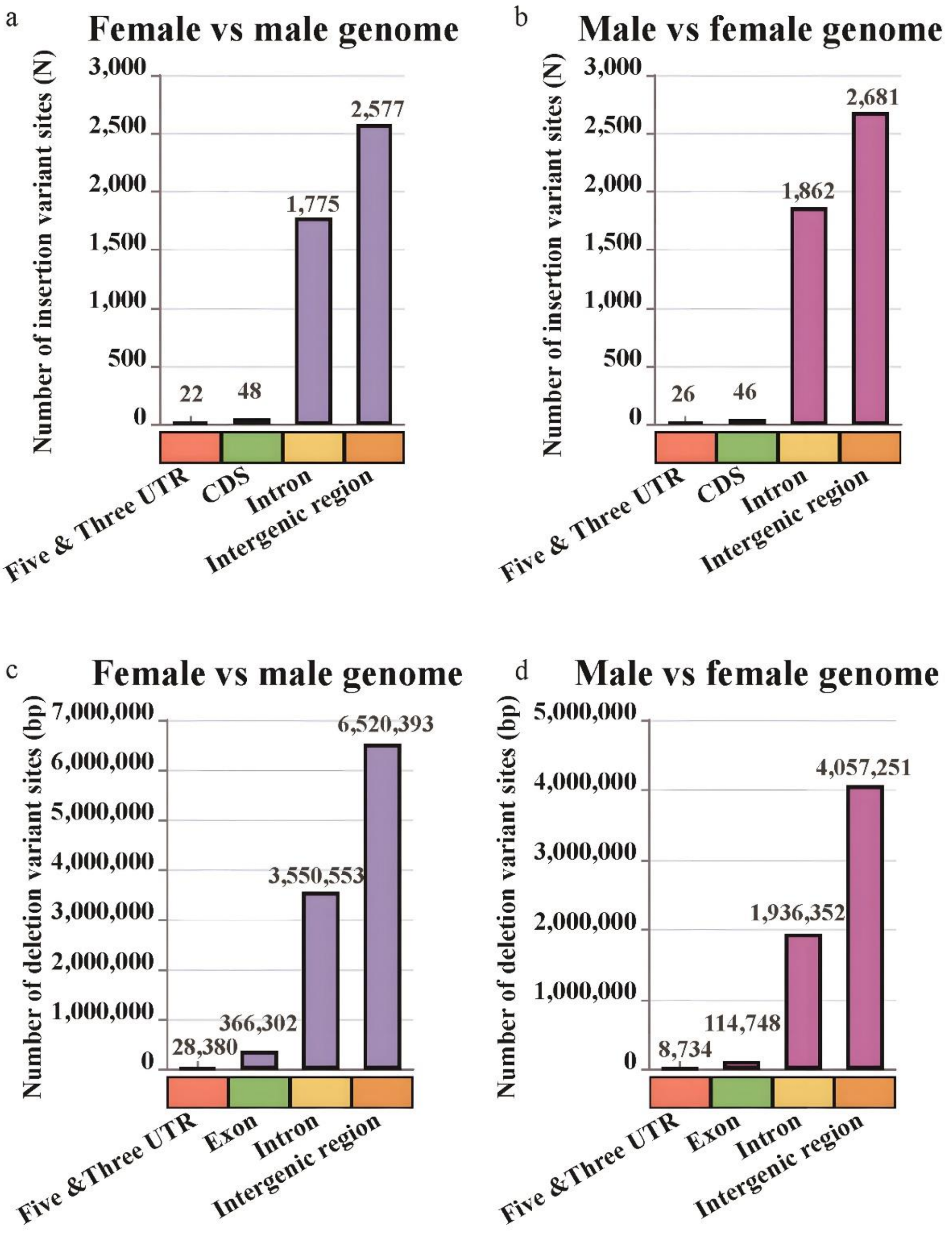

Figure 2: Distribution statistics of insertion and deletion fragment (＞100 bp) positions in genomic functional regions of Japanese parrotfish.

(a) Distribution statistics of insertion variant loci using the female fish genome as a reference. (b) Distribution statistics of insertion variant loci utilizing the male fish genome as a point of reference. (c) Distribution statistics of deletion variant length using the female fish genome as a reference. (d) Distribution statistics of deletion variant loci using the male fish genome as a reference.

Note: The colors red, green, yellow, and orange represented the UTR, CDS, intron, and intergenic regions. The frequency of insertions (a, b) was quantified based on the number of events, while deletions (c, d) were characterized by their length in nucleotides.

Results

Construction of insertion/deletion variant loci datasets for large segments

Using the female genome as a reference, we obtained 1,919,620 male and female differential variant sites with unfiltered data, including 191,915 insertion sites, 325,620 deletion sites and 1,402,085 nucleotide substitutions (Supplementary Female_reference_all_variation_Set.xls). To screen suitable field-actionable markers for large-band genetic sex identification, we screened this insertion/deletion variant site catalog for variants larger than 100 bp and obtained 4,422 insertion sites with a differential sequence size of 5,908,147 bp, of which 0.11% were located in 5′ UTRs, 0.38% in 3′ UTRs, 40.14% in intronic regions, 1.08% in CDS regions, and 58.28% in intergenic regions (Figure 2, Figure S1 , Supplementary Female_reference_INS/DEL100_Set.xlsx). In addition, we obtained 4,675 deletion sites with a differential sequence size of 10,465,628 bp (Supplementary Female_reference_INS/DEL100_Set.xlsx). As the deletion variant sites spanned multiple genomic functional regions, we performed statistical analysis of the actual locations where the deletion bases were located, with 0.08% located in 5′ UTRs, 0.19% in 3′ UTRs, 33.93% in intronic regions, 3.50% in CDS regions, and 62.30% in intergenic regions (Figure 2, Table 2). The highest number of chromosomal mutations occurred in female fish chromosomes 8 and 10, with 333 and 341 insertion and deletion sites, respectively (Figure 3).

Using the male Japanese parrotfish genome as a reference, 1,926,295 female-male differential variant sites were obtained with unfiltered data, including 326,823 insertion sites, 192,672 deletion sites and 1,406,800 nucleotide substitution sites (Supplementary Female_reference_all_variation_Set.xls). Through large fragment screening (>100 bp), we obtained 4,615 insertion sites with a differential sequence size of 10,100,983 bp, of which 0.13% were located in 5′ UTRs, 0.43% in 3′ UTRs, 40.35% in intronic regions, 1.00% in CDS regions, and 58.09% in intergenic regions (Figure 2, Figure S1, Table 2, Supplementary Male_reference_INS100_Set.xlsx). The chromosome with the highest number of mutations was chromosome 9 in male Japanese parrotfish, in which the number of insertion and deletion sites were 345 and 333, respectively (Figure 3); male chromosome 9 was homologous to chromosomes 8 and 10 of female Japanese parrotfish. Male chromosome 6 had the second highest number of mutations, with 289 insertion sites and 256 deletion sites (Figure 3).

Table 2. Statistics of the positional distribution of insertion/deletion variants in the male and female genomes of Japanese parrotfish.

Type of gene function region	Female genome as a reference		Male genome as a reference	
	Insertion variant loci no. (>100 bp)	Deletion variant length (>100 bp)	Insertion variant loci no. (>100 bp)	Deletion variant length (>100 bp)
5′ UTR	5	7,859	6	2,482
3′ UTR	17	20,521	20	6,252
CDS	48	366,302	46	114,748
Intron	1,775	3,550,553	1,862	1,936,352
Intergenic region	2,577	6,520,393	2,681	4,057,251
Total	4,422	10,465,628	4,615	6,117,085

Note: Insertions (>100 bp) were summarized by number of events. Deletions (>100 bp) were summarized by length in nucleotides.

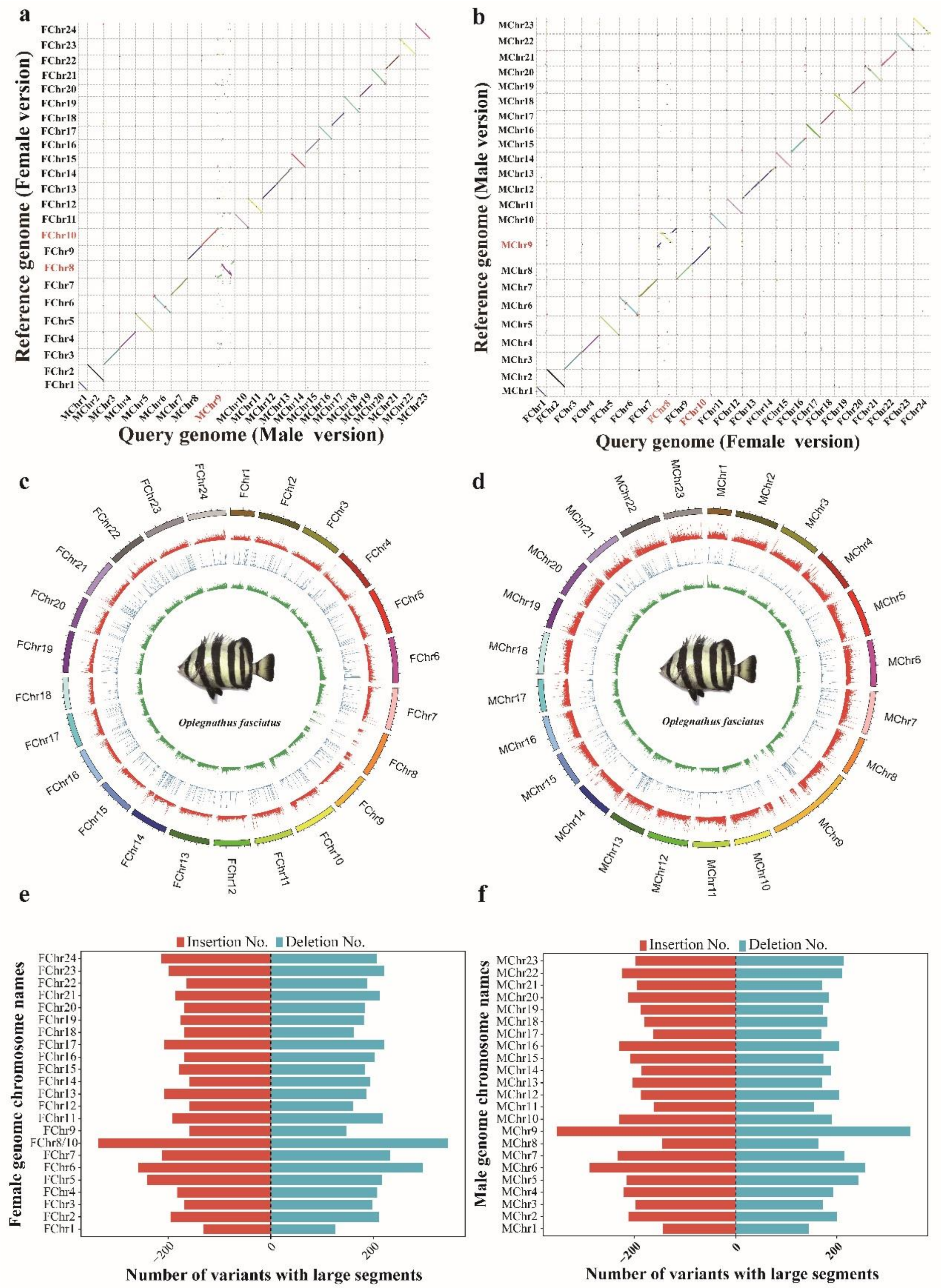

Figure 3: The covariance of genomic sequence differences between the male and female genomes of Japanese parrotfish and distribution statistics of variant sites.

(a) The female genome (reference) of Japanese parrotfish vs. the male genome for homology comparison using the minimap2 software with a 5,000 bp sliding window. The female genome was the vertical axis, and female chromosomes 8 and 10 were homologous to male chromosome 9. (b) The comparison of homology between the male genome of Japanese parrotfish and the female genome was conducted using the minimap2 software with a 5,000 bp sliding window. The male genome was represented on the vertical axis, revealing homology between male chromosome 9 and female chromosomes 8 and 10. (c) Distribution statistics of variant loci using the female fish

genome as a reference. Insertions (>100 bp) were summarized by number of events. Deletions (>100 bp) were summarized by length in nucleotides. From outer to inner circles were the chromosome name, positional distribution of insertion sites, base length distribution of deletion sites, and base substitution distribution, respectively. (d) Distribution statistics of variant loci using the male fish genome as a reference. (e) - (f) Dual-valued histograms corresponding to the Table 3 showed the number of insertion/deletion sites per chromosome with reference to the female (4,422 insertion sites, 4,675 deletion sites) and male (4,615 insertion sites, 4,385 deletion sites) genomes, respectively, where chromosome 9 in males was homologous to chromosomes 8 and 10 in females. Insertion sites were represented by negative values, and deletion sites were represented by positive values.

The SyRI software was further utilized to assess variant sites (＞100 bp) between the male and female reference genomes of Japanese parrotfish. By utilizing the male genome as a reference for comparison with the female genome, a total of 2,045 deletion sites (＞100 bp) and 2,110 insertion sites (＞100 bp) were identified. Additionally, comparison of the female genome with the male genome as a reference revealed 2,116 large segment deletion sites (＞100 bp) and 2,055 large segment insertion sites (＞100 bp). Considering the long-read length depth calculations and ranking large segments of deletion sites were beneficial when utilizing short-read sequencing data and long-read PacBio sequencing data to analyze visual differences between male and female genomes. Our study revealed that 1,791 deletion sequence tag sites identified through SyRI scanning overlapped with deletion sites identified in the female fish reference genome, accounting for 84.64% of the total 2,116 deletion sites identified (Fig.4a, Table 3). Meanwhile, the SyRI scanning method identified 1,749 deletion STS that intersected with deletion sites in the reference genome of male fish, representing 85.53% of the total 2,045 deletion sites (Fig.4b, Table 3). In the context of large deletion variants, a correlation was observed between the long-read depth and the effectiveness of markers. A frequency distribution map was created for the long-read depths of large deletion loci within male and female genomes, followed by marker categorization. The results indicated that markers with long-read depths below 5.48 (895, ♀ as reference) and 12.50 (1,021, ♂ as reference) demonstrated high recognition rates as sex markers, based on the male and female genomes as reference (Fig.4c-f). The findings further indicated that a substantial proportion of deletion sequence tag sites referencing the female genome were situated in transposable element regions (78.44%, n=3,667) and tandem repeat sequences regions (33.67%, n=1,574). Similarly, statistical analysis revealed that a notable percentage of deletion sequence tag sites referencing the male genome were linked to transposable element regions (77.08%, n=3,380) and tandem repeat regions (31.90%, n=1,399) (Table 4).

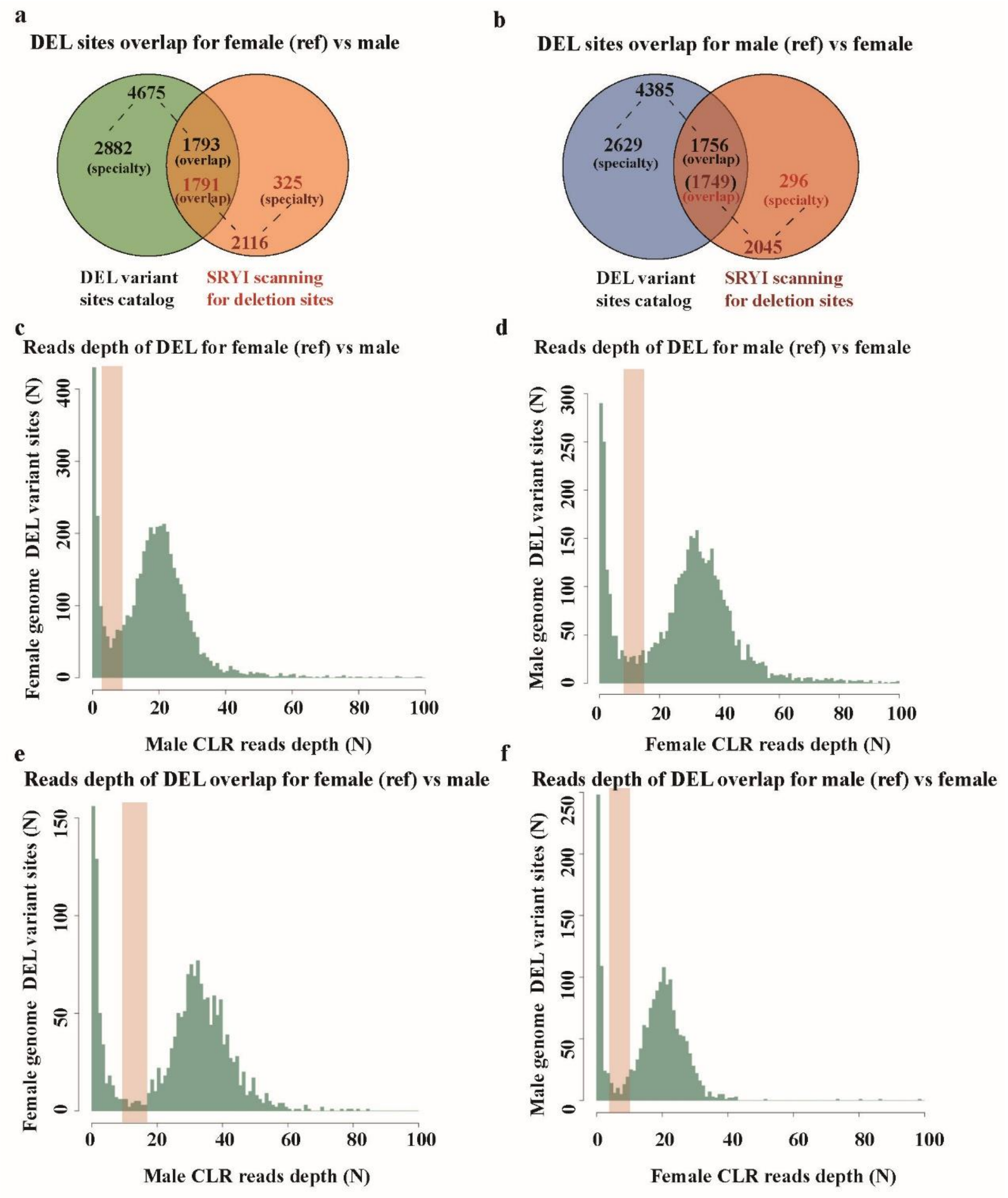

Figure 4: Intersection results of two variant site scanning methods and ranking of variant sites with long read depth frequency distributions

(a) Venn results of deletion locus data were obtained based on TBtools and SyRI software using the female fish genome as a reference, and 1,791 out of 2,116 deletion loci obtained based on SyRI software were shared deletion variant loci. (b) Utilizing the male fish genome as a point of reference for acquiring deletion site data in the context of Venn analysis, it was found that 1,749 out of the 2,045 deletion sites identified through the use of the SyRI software were common deletion variant sites. (c) Depth distribution of CLR reads in male fish using all female genomic deletion locus (＞100 bp) information as a reference, with the area where high-quality male and female genetic marker information was located before the orange labeled trough position (~5.4). (d) The depth distribution of CLR reads in females was analyzed by utilizing male genomic deletion sites as a reference (>100 bp), focusing on the region preceding the orange labeled trough position (~12.5) where high-quality male and female genetic marker information was present. (e) - (f) Depth distribution of CLR reads in males and females based on intersecting

male and female genomic deletion site information (>100 bp), respectively.

Table 3. Statistical cross-validation of multiple scanning of genome-wide large insertion deletion sequence marker sites.

Reference genome	Female vs. Male			Male vs. Female		
Mutation type	Insertion		Deletion	Insertion		Deletion
Variant length (>100 bp)	4,422		4,675	4,615		4,385
Chromosomes	FChr1	131	123	MChr1	140	143
	FChr2	194	211	MChr2	208	194
	FChr3	173	199	MChr3	179	161
	FChr4	181	207	MChr4	214	186
	FChr5	241	220	MChr5	212	237
	FChr6	254	292	MChr6	277	246
	FChr7	212	234	MChr7	231	211
	FChr8/10	333	341	MChr8	143	165
	FChr9	165	146	MChr9	345	333
	FChr11	191	217	MChr10	217	179
	FChr12	157	160	MChr11	159	153
	FChr13	205	187	MChr12	185	203
	FChr14	158	193	MChr13	196	162
	FChr15	182	185	MChr14	186	188
	FChr16	169	202	MChr15	190	160
	FChr17	204	220	MChr16	228	196
	FChr18	168	159	MChr17	159	167
	FChr19	175	183	MChr18	168	171
	FChr20	168	184	MChr19	174	166
	FChr21	185	203	MChr20	205	183
	FChr22	161	183	MChr21	184	167
	FChr23	200	219	MChr22	225	211
	FChr24	215	207	MChr23	190	203
SyRI-based scanning of male and female mutant sites (≥100 bp)			2,116			2,045
Intersection of the two methods			1,791 (84.64%)			1,749 (85.53%)

Table 4. Statistics of transposable elements and tandem repeat sequences attributed to large deletion sequence tag sites obtained with male and female reference genomes

Reference	Mutation type	Transposable	Tandem Repeat	Total

genome		elements		
Female vs. Male	Deletion	3,667 (78.44%)	1,574 (33.67%)	4,675
Male vs. Female	Deletion	3,380 (77.08%)	1,399 (31.90%)	4,385

Table 5. Statistics of large-segment differential loci after primer design, e-PCR amplification, and simulated amplification products and size screening.

Reference genome	Mutation type	Variant length (>100 bp)	High-throughput design of primers for variant sites	e-PCR of primers with single band	Single band and suitable amplification size (100~2000 bp)
Female vs. Male	Insertion	4,422	4,016	5,735	2,786
	Deletion	4,675	1,314	957	853
Male vs. Female	Insertion	4,615	4,131	3,236	2,876
	Deletion	4,385	1,199	882	796

High-throughput primer design and e-PCR bulk simulation for target regions

The identified large insertion/deletion variants were utilized as target regions for sex identification in both male and female genomes. High-throughput primers were designed to generate primer catalogs for the specified target markers. A total of 4,016 and 1,314 primer pairs were obtained by high-throughput primer design for the insertion/deletion target regions, respectively, using female genome as the reference (Table 5). To further test the accuracy and uniqueness of the high-throughput primers across the genome, we performed genome-wide electronic simulated amplification of the obtained primer pairs by using e-PCR. A total of 2,786 and 853 genome-wide single amplification band primer pairs were identified through screening of 5,735 primer pairs and 957 primer pairs with single bands, respectively, resulting in amplification products ranging from 100 to 2,000 bp (Table 5, Supplementary Female_Male_INS/DEL100_PrimerSet.xlsx). Utilizing the Japanese parrotfish male genome as a point of reference, 4,131 and 1,199 primer pairs were obtained to target insertions/deletions of specific variant sites, respectively (Table 2, Supplementary Female_Male_INS/DEL100_PrimerSet.xlsx). Notably, 2,876 and 796 primer pairs for whole-genome single amplification bands (100~2,000 bp) were identified through screening of 3,236 primer pairs and 882 primer pairs with single band (Table 5,

Supplementary Female_Male_INS/DEL100_PrimerSet.xlsx). Variations in the efficacy of PCR high-pass primer design at insertion and deletion sites may be attributed to disparities in structural characteristics and GC content within the regions flanking the insertion/deletion variants.

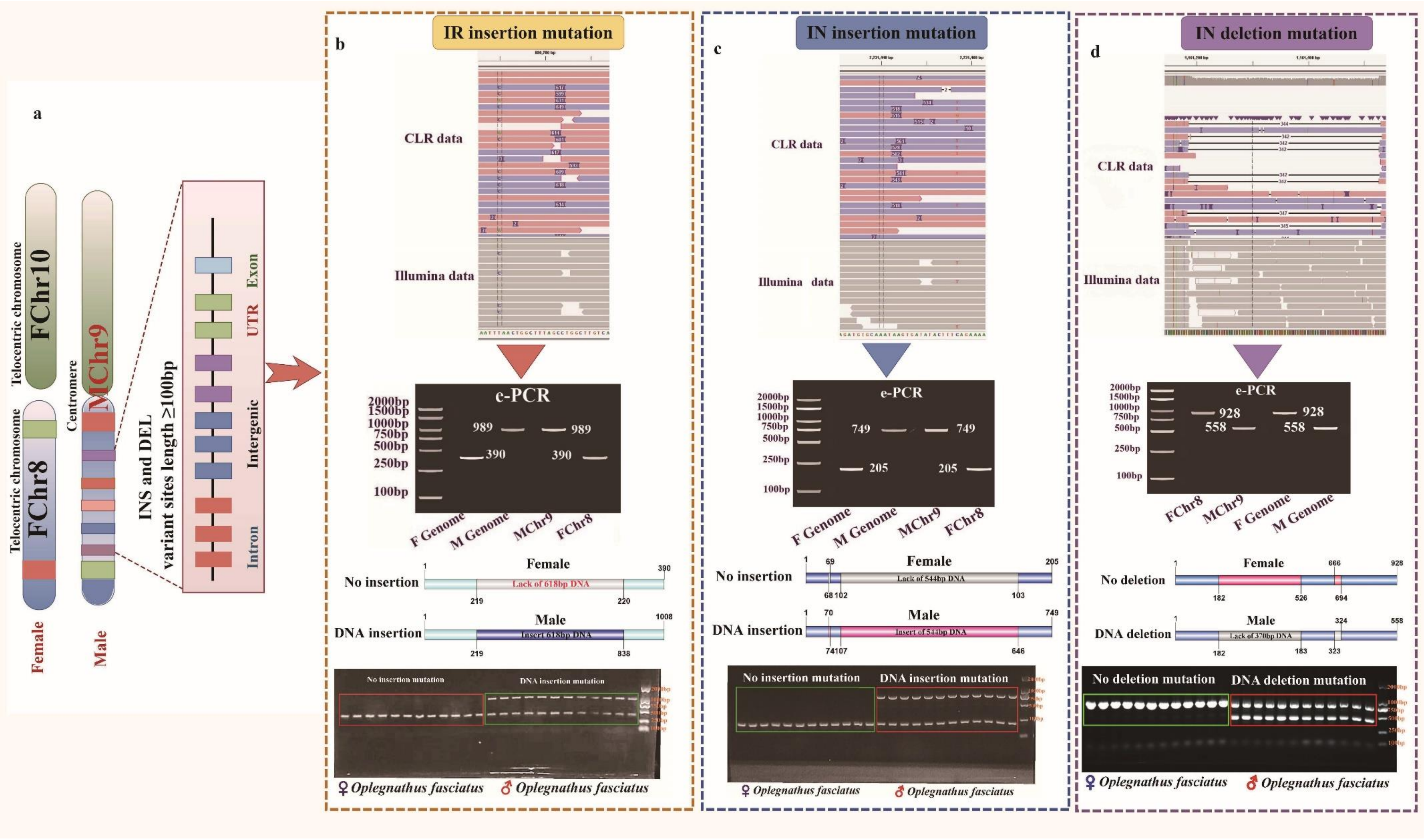

Figure 5: Representative genetic sex marker (insertion deletion variant sites) located in the intergenic region (IR), Intronic region (IN) of the Japanese parrotfish genome (STOSOd393e).

(a) Location of male and female sex markers in the male (MChr9) and female (FChr8) genomes. (b) Male and female sex genetic markers were located at loci in the intergenic region, using the female fish genome as a reference (Figure S2). Illumina and CLR clean data of bam comparison to the genome for insertion/deletion site validity detection. Pink and blue data from PacBio represented the same and opposite orientation of female genome and gray represented Illumina clean data. The e-PCR amplified bands were 390 bp and 989 bp in size, corresponding to DNA insertion and deletion patterns and agarose gel electrophoresis for identification of male and female genetically different populations. (c) By utilizing the female genome as a point of comparison, the genetic markers for male and female sexes were identified at specific insertion sites within *nuf2* gene introns (Figure S3, Figure S4). The resulting e-PCR amplified bands, measuring 205 bp and 749 bp in size, were indicative of distinct DNA insertion patterns and were utilized in agarose gel electrophoresis for the purpose of distinguishing between male and female genetically differentiated groups. Males exhibited two amplified bands, while females exhibited only one. (d) By utilizing the female fish genome as a point of comparison, the genetic markers for male and female sexes were situated within the deletion sites located in the introns of genes (Figure S5, Figure S6). The amplified bands produced through e-PCR were observed to be 558 bp and 928 bp in length, aligning with the DNA deletion patterns and agarose gel electrophoresis maps utilized for distinguishing genetically distinct groups of males and females. Specifically, two bands were amplified in males, while only one band was observed in females.

Validation of molecular markers for genetic sex identification of females and males We selected markers located in the intergenic and intragenic regions for female and male genetic sex identification for validation testing. Using the female genome as a reference, a 599 bp DNA fragment was inserted into the male genome at position 880,783 bp on female chromosome 8 (after TTTAGC), corresponding to e-PCR amplicons of 390 bp and 1008 bp, respectively (Figure 5, Figure S2). Sequence comparisons of the genetic sex markers showed that the genetic differences between male and female markers were mainly concentrated in the region of 200~800 bp. This variant locus was further confirmed as a valid insertion site using female fish as a reference genome, synthesized using genomic survey data from short-read sequencing data survey of male fish and long-read sequencing data from PacBio platform for read assignment (Figure 4, Figure S1). The results of PCR amplification and agarose gel electrophoresis were consistent with the results of e-PCR corresponding to two bands (390 bp and 1,008 bp) for males and one band (390 bp) for females. According to the genetic sex identification marker localization, the marker lies between the *samd3* gene (sterile alpha motif.) (Ofa002334) and *elf3* gene (E74-like factor 3) (Ofa002335) in the female genome (Figure 5, Figure S2).

The intragenic markers obtained from screening were also used to test the effectiveness of female and male genetic sex identification in Japanese parrotfish. In the region corresponding to the intron of the female *nuf2* gene (component of the NDC80 kinetochore complex) (Ofa002358) on chromosome 8 (2,231,443 bp, after GCAAATA), a 518 bp DNA fragment was inserted into the intron of the male *nuf2* gene, corresponding to e-PCR amplified fragment sizes of 206 bp and 739 bp, respectively (Figure S3). With the female genome as a reference, we utilized the male short-read sequencing data and long-read PacBio sequencing data for read assignment and further confirmed that this candidate variant site was a valid insertion site (Figure S4). Comparison of the sequences for the genetic sex marker showed that the genetic differences between the male and female markers were mainly in the region of 90~650 bp. The results of PCR amplification and agarose gel electrophoresis further showed that the males exhibited two bands (206 bp and 739 bp), and the females had one band (206 bp), in agreement with the e-PCR results (Figure S4).

In addition to the male insertion genetic sex markers, a male deletion genetic sex marker was also identified. In the region corresponding to female chromosome 8 from 1,161,186 bp (after GATGAGGAAAG) to 1,161,530 bp (before TATGAAGTCT), the males were missing a 343 bp DNA fragment, corresponding to e-PCR amplicon sizes of 558 bp and 928 bp, respectively (Figure S5). A comparison of sequence differences, short-read sequencing data

and long-read PacBio sequencing data read assignments all confirmed that the genetic differences between male and female markers were mainly concentrated in the 182~551 bp and 665~693 bp regions (Figure S6). PCR amplification and agarose gel electrophoresis assays showed two bands in males (558 bp and 928 bp) and one band in females (928 bp), which was consistent with the results of e-PCR (Figure S6). A similar genetic sex marker for the deletion locus in males was also identified in the region corresponding to chromosome 8 of females at 921,537 bp (after AAATGTGGCGG), with deletion of a 299 bp DNA fragment and insertion of a 119 bp DNA multi-fragments in males. PCR amplification and agarose gel electrophoresis tests showed two bands in males (748 bp and 926 bp) and one band in females (926 bp), consistent with the e-PCR results (Figure S7, Figure S8).

Discussion

Fish physiological sex is influenced by environmental factors, particularly temperature, resulting in discrepancies between the physiological phenotype and genotype of sex [10-11]. Consequently, it is crucial within the field of fish aquaculture to discover a straightforward approach capable of rapidly determining the genetic sex [61]. DNA molecular markers are created by exploiting the prevalence of polymorphisms in the genomic DNA of eukaryotic organisms, which serves as indicators of individual variations at the DNA level [7]. A review of the literature revealed that over the past decade, approximately 48% of recent studies on male and female sex determination have predominantly employed molecular biology techniques such as RFLP, RAPD, SSR, and AFLP [61-72]. Meanwhile, with the advancement of second-generation high-throughput sequencing technologies, particularly Illumina, the utilization of sex markers based on SNP/InDel variants has increased to approximately 51% [16, 73-78]. With the emergence and rapid advancement of third-generation sequencing technology, exemplified by long-fragment read-length PacBio sequencing, the development of third-generation high-throughput sex markers based on insertions or deletions of large fragments (>100 bp) has become feasible [37, 45, 46]. Our study focuses on Japanese parrotfish with the $X_1X_1X_2X_2/X_1X_2Y$ system [44-46, 48]. We use the assembled genome information of male and female individuals, along with short-read and long-read data, to identify variant sites and evaluate read depth. We also design primers for target regions and perform batch amplification using e-PCR (Figure 1, Figure 2, Figure 3, Table 2, Table 3). Subsequently, a high-throughput identification catalog of sex-specific markers has been established through this methodology, leading to the discovery of 3,639 (2,786 INS/853 DEL, ♀ as reference) and 3,672 (2,876 INS/833 DEL, ♂ as reference) markers, along with 1,021 and 894 high-quality

genetic sex identification markers, respectively (Table 2, Table 3). Following agarose gel electrophoresis, two amplification bands for males and one amplification band for females are successfully identified, enabling rapid differentiation between the sexes in Japanese parrotfish. This advancement in sex marker development, utilizing third-generation high-throughput and precise techniques, will expedite the progress of genetic breeding and facilitate the establishment of genetic sex markers.

Variation in genome structure is intricately linked to human diseases, evolution, gene regulation, and species phenotypes in the biological realm, making it a prominent area of study in genomics research [79]. Previous studies have shown that DNA sequences with insertions and deletions undergo increased selective pressure [80-84]. The presence of deletions and insertions in DNA sequences, regardless of their location in noncoding or coding regions, inevitably influences the functionality of the original sequence to varying extents [79]. Furthermore, evidence also suggests that the length and placement of insertions are not random but rather heavily influenced by the adjacent DNA sequence [80-82, 85, 86]. Prior research has indicated that insertion deletion variants accounted for 16%, 24%, 13%, 25%, and 15% of genomic genetic polymorphisms in various organisms, including *Drosophila melanogaster*, *Caenorhabditis elegans*, *Mus musculus castaneus*, *Homo sapiens*, and *Arabidopsis thaliana* [34-36, 87-94]. Recent advancements in long-read length sequencing technology have allowed for a more precise examination of human genetic variation, revealing that insertions and deletions collectively contribute to a high portion (13.8%, 11.1%) of genetic polymorphisms in the human genome [90-94]. The focus of genomic variation studies has predominantly been on single nucleotide polymorphisms (SNPs) and insertions/deletions (InDels) of less than 50 base pairs due to the limitations of high-throughput sequencing technologies for short read lengths. However, larger genomic structural variants, which encompassed genetic variations not fully captured by SNPs and InDels, are closely linked to biological phenotypes and might significantly influence phenotypic traits, disease susceptibility, and adaptive capacity [79]. The utilization of long-read length sequencing technologies such as PacBio and ONT has enabled the generation of extended DNA sequences, thereby enhancing the assembly of diploid genomes of superior quality and the identification of structural variants (SVs) across substantial genomic regions [95]. Previous research has documented seven deletions (>100 bp) within approximately 700bp large segments of the *ZmBAM1d* gene, demonstrating a positive association with maize kernel weight. These deletions are consistently inherited in high-yielding maize lines [96]. In mammalian studies, deletion of approximately 40 Mb on chimpanzee chromosome 2, as compared to humans and gorillas within the great ape family,

has been identified, leading to stable genetic differentiation among species [97]. Similar results have been documented in *Astyanax mexicanus*, *Gasterosteus aculeatus*, *Equus caballus*, and *Canis lupus familiaris* [98-101]. The emergence of long-read length sequencing technology has propelled large fragment genomic structural variation to the forefront of genetic variation research, particularly in investigations of species' adaptive evolution [95, 96, 102, 103]. The findings of this study indicate that insertions and deletions identified using the female and male Japanese parrotfish genomes as references constituted 26.96% and 26.97% of the genetic polymorphisms, respectively. Our research has contributed new insights into the prevalence of large-segment insertion-deletion events for Japanese parrotfish, indicating that these events predominantly occurred in the intergenic region in 58.09%~66.33% of instances, with the intronic region following closely at 31.65%~40.35% of cases (Figure 2, Figure S1 Table 2). We also observe a higher proportion of deletion sites compared to insertion sites. A similar study conducted on the *β-fibrinogen* intron of birds has reported similar findings [104]. Furthermore, this study presents novel findings indicating that over 77% of the identified large deletion sequence tag sites in the genomes of both female and male Japanese parrotfish are attributed to transposable elements (Table 4). It is evident that transposable elements are prevalent in the animal genome and significantly contribute to its evolutionary processes. Previous research has demonstrated that a significant portion (69.5%) of genomic structural variation in large segments of tomato is situated within the transposable element region, which is closely linked to desirable traits in tomato [105]. Comparable studies have also been conducted on gene regulation in human brain diseases and genomic structural variation in evolutionary distinct phenotypes of great apes [103, 106]. Transposable elements have the potential to drive genome and chromosome evolution through various mechanisms such as insertion-induced gene variation, gene retrotransposition, chromosome recombination, gene duplication, and serving as sites for ectopic recombination. These findings underscore the importance of transposable elements in facilitating adaptive evolution for Japanese parrotfish. The presence of large fragments of insertion deletion sequence marker sites is frequently influenced by natural selection pressures, with their formation being intricately linked to adjacent DNA sequences and commonly correlated with characteristic phenotypic traits and aberrant gene regulation [83, 84, 102, 103, 105, 106]. Specifically, the manifestation of albino symptoms in dogs resulting from a 965-kb deletion on chromosome 18 exhibits stable inheritance patterns, while a 4.8 Mb deletion variant on the chromosome of male domestic chickens similarly displays heritability across subsequent generations [100, 107]. Consistent inheritance of variants within populations has also been documented in academic research on

maize (*ZmBAM1d*), *Astyanax mexicanus*, *Ailuropoda melanoleuca* (*Bace2*), great apes (*acan*), and human diseases such as hypertension (*ace*) [96, 98, 103, 108]. The findings from the population genetic sex marker amplification analysis conducted in this study indicated that the insertion-deletion markers exhibited stability in both male and female populations of Japanese parrotfish. Additionally, analysis of gonadal histology and sex-validated marker data from various geographic populations demonstrate the consistent inheritance of large segments of the insertion deletion markers in individuals of both sexes across different geographic regions (Figure S9). This observation provided additional evidence to support the theory that large-segment insertions and deletions are not random occurrences but rather influenced by natural selection pressures [80, 82-84]. Based on the present findings, the stability of large segment insertion and deletion in sequence tag sites is evident. However, this should not be taken to imply that all sequence tag sites exhibit this characteristic. A comprehensive evaluation of the evolutionary rate of sequence tags and the expansion of variant marker detection is necessary for ongoing research in this area.

Conclusions

These results highlight the substantial prevalence of insertion and deletion events in the genetic makeup derived from the $X_1X_1X_2X_2/X_1X_2Y$ sex determination system of Japanese parrotfish. In our study, we have successfully exploited a novel approach for the development of genetic sex markers in Japanese parrotfish. This approach involved the utilization of a primer batch electronic design technique, which utilized the insertion and deletion loci as the target regions. Additionally, we implement an electronic batch amplification technique, allowing for the efficient and rapid high-throughput identification and screening of a substantial number of variant sites. This methodology represents a significant advancement in the field of Japanese parrotfish genetic sex marker development. The study yields a greater number of genetic sex markers with higher amplification efficiency compared to those generated by previous technologies spanning the first or second generation. This methodology is applicable not only to the efficient generation of genetic sex markers for the $X_1X_1X_2X_2/X_1X_2Y$ sex-determination system but also to the determination of genetic sex in various sex-determination types (including autosomal and sex chromosome differences). It was particularly well suited for sex-determination systems featuring heteromorphic chromosomes, such as the XY-type, ZW-type, XX/XY_1Y_2-type, ZZ/ZW_1W_2-type, and other species with different sex-determination mechanisms.

Ethics Statement

This research was approved by the Animal Care and Use Committee of the Chinese Academy of Science.

Competing Interests

The authors declare no competing interests.

Acknowledgement

This work was supported by grants from the National Natural Science Foundation of China (No. 42276107, 32270472), CARs for Marine Fish Culture Industry (CARS-47), the National Key Research and Development Program (2022YFC3103600), Shandong Province Key R&D Program (2023LZGCQY001), Key Deployment Projects of Center for Ocean Mega-Science, Chinese Academy of Sciences (Fron-tier Cross-category, COMS2020Q05).

Data Availability

Sequencing data is available from NCBI BioProjects PRJNA486885 and PRJNA563003. The code used for statistics on insertions and deletions as well as annotation scripts and base variant region visualization scripts can be access from Figshare [109]. All additional supporting data are available in the *GigaScience* repository, GigaDB [110].

Abbreviations

CLR: Continuous Long Reads; CCS: circular consensus sequence.

References

1. Hughes JF, Skaletsky H, Pyntikova T et al. Chimpanzee and human Y chromosomes are remarkably divergent in structure and gene content, Nature. 2010;463:536-539.
2. Bitencourt JA, Sampaio I, Ramos RT et al. First Report of Sex Chromosomes in Achiridae (Teleostei: Pleuronectiformes) with Inferences About the Origin of the Multiple X(1)X(1)X(2)X(2)/X(1)X(2)Y System and Dispersal of Ribosomal Genes in *Achirus achirus*, Zebrafish. 2017;14:90-95.
3. Chen S, Zhang G, Shao C et al. Whole-genome sequence of a flatfish provides insights into ZW sex chromosome evolution and adaptation to a benthic lifestyle, Nat Genet. 2014;46:253-260.
4. Lu G, Luo M. Genomes of major fishes in world fisheries and aquaculture: Status, application and perspective, Aqua Fish. 2020;5:163-173.
5. Chen SL, Ji XS, Shao CW et al. Induction of mitogynogenetic diploids and identification of WW super-female using sex-specific SSR markers in half-smooth tongue sole (*Cynoglossus semilaevis*), Mar Biotechnol (NY). 2012;14:120-128.
6. Pan ZJ, Li XY, Zhou FJ et al. Identification of Sex-Specific Markers Reveals Male Heterogametic Sex Determination in Pseudobagrus ussuriensis, Mar Biotechnol (NY). 2015;17:441-451.
7. Ma Y, Xiao Y, Xiao Z et al. Identification of Male-Specific Molecular Marker and Development of PCR-Based Genetic Sex Identification Technique in Spotted Knifejaw (*Oplegnathus punctatus*), Mar Biotechnol (NY).

2022;24:969-978.

8. Wu Y, Xiao Y, Xiao Z et al. Identification of Male-Specific Molecular Markers by Recombination of RhoGEF10 Gene in Spotted Knifejaw (*Oplegnathus punctatus*), Genes (Basel). 2022;13.

9. Li XY, Gui JF. Diverse and variable sex determination mechanisms in vertebrates, Sci China Life Sci. 2018;61:1503-1514.

10. Chen J, Zhu Z, Hu W. Progress in research on fish sex determining genes, Water Biol Secur. 2022;1.

11. Shen Z-G, Wang H-P. Molecular players involved in temperature-dependent sex determination and sex differentiation in Teleost fish, Genet Sel Evol. 2014;46:26.

12. Dan C, Mei J, Wang D et al. Genetic differentiation and efficient sex-specific marker development of a pair of Y- and X-linked markers in yellow catfish, Int J Biol Sci. 2013;9:1043-1049.

13. Liao X, Xu G, Chen SL. Molecular method for sex identification of half-smooth *tongue sole* (Cynoglossus semilaevis) using a novel sex-linked microsatellite marker, Int J Mol Sci. 2014;15:12952-12958.

14. Ning Y, Liu X, Wang ZY et al. A genetic map of large yellow croaker *Pseudosciaena crocea*, Aquaculture. 2007;264:16-26.

15. Xiao S, Wang P, Zhang Y et al. Gene map of large yellow croaker (*Larimichthys crocea*) provides insights into teleost genome evolution and conserved regions associated with growth, Sci Rep. 2015;5:18661.

16. Lin A, Xiao S, Xu S et al. Identification of a male-specific DNA marker in the large yellow croaker (*Larimichthys crocea*), Aquaculture. 2017;480:116-122.

17. Tao W, Zhu X, Cao J et al. Screening and characterization of sex-linked DNA markers in Mozambique tilapia (*Oreochromis mossambicus*), Aquaculture. 2022;557.

18. Aparicio S, Chapman J, Stupka E et al. Whole-genome shotgun assembly and analysis of the genome of *Fugu rubripes*, Science. 2002;297:1301-1310.

19. Mitani H, Kamei Y, Fukamachi S et al. The medaka genome: why we need multiple fish models in vertebrate functional genomics, Genome Dyn. 2006;2:165-182.

20. Jones FC, Grabherr MG, Chan YF et al. The genomic basis of adaptive evolution in threespine sticklebacks, Nature. 2012;484:55-61.

21. Davidson WS, Koop BF, Jones SJ et al. Sequencing the genome of the Atlantic salmon (*Salmo salar*), Genome Biol. 2010;11:403.

22. Gong G, Dan C, Xiao S et al. Chromosomal-level assembly of yellow catfish genome using third-generation DNA sequencing and Hi-C analysis, GigaScience. 2018;7:giy120.

23. Luo H, Zhang Y, Liu F et al. The male and female genomes of golden pompano (*Trachinotus ovatus*) provide insights into the sex chromosome evolution and rapid growth, J Adv Res. 2023. https://doi.org/10.1016/j.jare.2023.11.030.

24. McGaugh SE, Gross JB, Aken B et al. The cavefish genome reveals candidate genes for eye loss, Nat Commun. 2014;5:5307.

25. Pan H, Yu H, Ravi V et al. The genome of the largest bony fish, ocean sunfish (*Mola mola*), provides insights into its fast growth rate, GigaScience. 2016;5:36.

26. Schartl M, Walter RB, Shen Y et al. The genome of the platyfish, *Xiphophorus maculatus*, provides insights into evolutionary adaptation and several complex traits, Nat Genet. 2013;45:567-572.

27. Valenzano DR, Benayoun BA, Singh PP et al. The African turquoise killifish genome provides insights into evolution and genetic architecture of lifespan, Cell. 2015;163:1539-1554.

28. Xiao Y, Liu J, Wei J et al. Improved high-quality reference genome of red drum facilitates the processes of resistance-related gene exploration, Sci Data. 2023;10:774.

29. Xu M-R-X, Liao Z-Y, Brock JR et al. Maternal dominance contributes to subgenome differentiation in

allopolyploid fishes, Nat Commun. 2023;14:8357.

30. Zhang W, Yang Y, Hua S et al. Chromosome-level genome assembly and annotation of the yellow grouper, *Epinephelus awoara*, Sci Data. 2024;11:151.

31. Shi L, Guo Y, Dong C et al. Long-read sequencing and de novo assembly of a Chinese genome, Nat Commun. 2016;7:12065.

32. VanBuren R, Bryant D, Edger PP et al. Single-molecule sequencing of the desiccation-tolerant grass *Oropetium thomaeum,* Nature. 2015;527:508-511.

33. Vij S, Kuhl H, Kuznetsova IS et al. Chromosomal-Level Assembly of the Asian Seabass Genome Using Long Sequence Reads and Multi-layered Scaffolding, PLoS Genet. 2016;12:e1005954.

34. Aganezov S, Yan SM, Soto DC et al. A complete reference genome improves analysis of human genetic variation, Science. 2022;376:eabl3533.

35. Nurk S, Koren S, Rhie A et al. The complete sequence of a human genome, Science. 2022;376:44-53.

36. Schneider VA, Graves-Lindsay T, Howe K et al. Evaluation of GRCh38 and de novo haploid genome assemblies demonstrates the enduring quality of the reference assembly, Genome Res. 2017;27:849-864.

37. Burton JN, Adey A, Patwardhan RP et al. Chromosome-scale scaffolding of de novo genome assemblies based on chromatin interactions, Nat Biotechnol. 2013;31:1119-1125.

38. Xiao Y, Liu J, Wei J et al. Improved high-quality reference genome of red drum facilitates the processes of resistance-related gene exploration, Sci Data. 2023;10:774.

39. Schembri PJ, Bodilis P, Evans J et al. Occurrence of barred knifejaw, *Oplegnathus fasciatus* (Actinopterygii: Perciformes: Oplegnathidae), in Malta (Central Mediterranean) with a discussion on possible modes of entry, Acta Ichthyol Piscat. 2010;40:101-104.

40. Mundy BC. Checklist of the fishes of the Hawaiian Archipelago. Bishop Museum Press; 2005.

41. An HS, Kim MJ, Hong SWJG et al. Genetic diversity of rock bream *Oplegnathus fasciatus* in Southern Korea, Genes Genom. 2008;30:451-459.

42. Xiao Y, Li J, Ren G et al. Pronounced population genetic differentiation in the rock bream *Oplegnathus fasciatus* inferred from mitochondrial DNA sequences, Mitochondrial DNA A. 2016;27:2045-2052.

43. Xu D, Lou B, Bertollo LAC et al. Chromosomal mapping of microsatellite repeats in the rock bream fish *Oplegnathus fasciatus*, with emphasis of their distribution in the neo-Y chromosome, Mol Cytogenet. 2013;6:1-6.

44. Xue R, An H, Liu Q et al. Karyotype and Ag-NORs in male and female of *Oplegnathus punctatus*, Oceanol Limnol Sin. 2016;47:626-632.

45. Xiao Y, Xiao Z, Ma D et al. Genome sequence of the barred knifejaw *Oplegnathus fasciatus* (Temminck & Schlegel, 1844): The first chromosome-level draft genome in the family Oplegnathidae, GigaScience. 2019;8:giz013.

46. Xiao Y, Xiao Z, Ma D et al. Chromosome-Level Genome Reveals the Origin of Neo-Y Chromosome in the Male Barred Knifejaw *Oplegnathus fasciatus*, iScience. 2020;23:101039.

47. Xu T, Shao C, Liao X et al. Isolation and characterization of polymorphic microsatellite DNA markers in the rock bream (*Oplegnathus fasciatus*), Conserv Genet. 2009;10:527-529.

48. Xu D, Lou B, Xu H et al. Isolation and characterization of male-specific DNA markers in the rock bream *Oplegnathus fasciatus*, Mar Biotechnol (NY). 2013;15:221-229.

49. Gong J, Zhao J, Ke Q et al. First genomic prediction and genome-wide association for complex growth-related traits in Rock Bream (*Oplegnathus fasciatus*), Evol Appl. 2022;15:523-536.

50. Yong SX. NCBI Sequence Read Archive https://identifiers.org/ncbi/insdc.sra:SRP220007. 2020.

51. Xiao Y, Xiao Z, Ma D et al. Supporting data for "Genome sequence of the barred knifejaw *Oplegnathus*

fasciatus (Temminck & Schlegel, 1844): the first chromosome-level draft genome in the family Oplegnathidae", GigaScience Database 2019:http://dx.doi.org/10.5524/100556.

52. Chen C, Wu Y, Xia R. A painless way to customize Circos plot: From data preparation to visualization using TBtools, iMeta. 2022;1.

53. Chen C, Chen H, Zhang Y et al. TBtools: An Integrative Toolkit Developed for Interactive Analyses of Big Biological Data, Mol Plant. 2020;13:1194-1202.

54. Li H. Minimap2: pairwise alignment for nucleotide sequences, J Bioinformatics. 2018;34:3094-3100.

55. Li H, Durbin RJb. Fast and accurate short read alignment with Burrows–Wheeler transform, Bioinformatics. 2009;25:1754-1760.

56. Krzywinski M, Schein J, Birol I et al. Circos: an information aesthetic for comparative genomics, Genome Res. 2009;19:1639-1645.

57. Kearse M, Moir R, Wilson A et al. Geneious Basic: an integrated and extendable desktop software platform for the organization and analysis of sequence data, Bioinformatics. 2012;28:1647-1649.

58. Darling AE, Mau B, Perna NT. ProprogressiveMauve: multiple genome alignment with gene gain, loss and rearrangement, PLoS One. 2010;5:e11147.

59. Zhou L, Feng T, Xu S et al. ggmsa: A visual exploration tool for multiple sequence alignment and associated data, Brief Bioinform. 2022;23:bbac222.

60. Hu B, Jin J, Guo A-Y et al. GSDS 2.0: an upgraded gene feature visualization server, Bioinformatics. 2015;31:1296-1297.

61. Mei J, Gui JF. Genetic basis and biotechnological manipulation of sexual dimorphism and sex determination in fish, Sci China Life Sci. 2014;44:5.

62. Agawa Y, Saiki T, Miyamoto T et al. Identification of the male sex-linked DNA sequence of cultured white trevally *Pseudocaranx dentex*, Fisheries Sci. 2022;88:319-327.

63. Goyat S, Grewal A, Singh D et al. Sex-linked AFLP marker identification in dioecious Betelvine (*Piper betle* L.), J Hortic Sci Biotech. 2019;94:422-427.

64. Hashem MH. Early sexing of Tilapia nilotica (*Oreochromis niloticus*) by using short sequence repeats (SSRs) molecular markers, Damanhour J Vet Sci. 2022;8:22-25.

65. Hua J, Qiang J, Tao Y et al. Development and validation of a PCR-RFLP/TaqMan MGB probe method for rapid sex identification of largemouth bass (*Micropterus salmoides*), Aquacult Rep. 2023;30:101593.

66. Jia X, Lu J, Tang X et al. A new method for molecular sex identification in the emu (*Dromaius novaehollandiae*), Brit Poultry Sci. 2023:1-6.

67. Lajan S, Al-Barzinji YMS. Detection of quantitative loci correlation with growth traites in local quail using PCR-RFLP technique, Iraqi J Agric Sci. 2022;53:16-26.

68. Law D, Ping VC, Yee TC et al. Use of amplified fragment length polymorphism and sequence characterized amplified region marker for identifying the sex of the *Oxyeleotris marmorata*, Pertanika J Trop Agr. 2021;1.

69. Mohamed G, Youssef M, Mohamed E et al. Identification of Sex-specific Molecular Markers in Barbel and Nile Carp using SCoT and ISSR Markers, Assiut J Agr Sci. 2021;52:60-73.

70. Panagiotopoulou H, Marzecki K, Gawor J et al. Extensive search of genetic sex markers in Siberian (*Acipenser baerii*) and Atlantic (*A. oxyrinchus*) sturgeons, Aquaculture. 2023;573:739517.

71. Vaux F, Aycock HM, Bohn S et al. Sex identification PCR–RFLP assay tested in eight species of Sebastes rockfish, Conserv Genet Resour. 2020;12:541-544.

72. Wen M, Zhang Y, Wang S et al. Characterization of sex locus and sex-specific sequences in the mandarin fishes, Aquaculture. 2022;561:738650.

73. Zheng S, Wang X, Zhang S et al. Screening and characterization of sex-linked DNA markers and marker-

assisted selection in the Southern catfish (*Silurus meridionalis*), Aquaculture. 2020;517:734783.

74. Liu H, Pang M, Yu X et al. Sex-specific markers developed by next-generation sequencing confirmed an XX/XY sex determination system in bighead carp (*Hypophthalmichehys nobilis*) and silver carp (*Hypophthalmichthys molitrix*), DNA Res. 2018;25:257-264.

75. Han C, Zhu Q, Lu H et al. Screening and characterization of sex-specific markers developed by a simple NGS method in mandarin fish (*Siniperca chuatsi*), Aquaculture. 2020;527:735495.

76. Palaiokostas C, Bekaert M, Khan MG et al. Mapping and validation of the major sex-determining region in Nile tilapia (*Oreochromis niloticus* L.) using RAD sequencing, PLoS One. 2013;8:e68389.

77. Wang L, Xie N, Shen Y et al. Constructing high-density genetic maps and developing sexing markers in northern snakehead (*Channa argus*) 2019;21:348-358.

78. Wu X, Zhao L, Fan Z et al. Screening and characterization of sex-linked DNA markers and marker-assisted selection in blue tilapia (*Oreochromis aureus*), Aquaculture. 2021;530:735934.

79. Sedlazeck FJ, Rescheneder P, Smolka M et al. Accurate detection of complex structural variations using single molecule sequencing, Nat Methods. 2018;15: 461-468 .

80. Chen Y, Wang AY, Barkley CA et al. Deciphering the exact breakpoints of structural variations using long sequencing reads with DeBreak, Nat Commun. 2023;14:283.

81. Maki H. Origins of spontaneous mutations: specificity and directionality of base-substitution, frameshift, and sequence-substitution mutageneses, Annu Rev Genet. 2002;36:279-303.

82. Tian D, Wang Q, Zhang P et al. Single-nucleotide mutation rate increases close to insertions/deletions in eukaryotes, Nature. 2008;455:105-108.

83. Cooper DN, Krawczak M. Mechanisms of insertional mutagenesis in human genes causing genetic disease, Hum Genet. 1991;87:409-415.

84. Krawczak M, Cooper DN. Gene deletions causing human genetic disease: mechanisms of mutagenesis and the role of the local DNA sequence environment, Hum Genet. 1991;86:425-441.

85. Cooper DN, Krawczak M. Mechanisms of insertional mutagenesis in human genes causing genetic disease Hum Genet. 1991;87:409-415.

86. Krawczak M, Cooper DN. Gene deletions causing human genetic disease: mechanisms of mutagenesis and the role of the local DNA sequence environment, Hum Genet. 1991;86:425-441.

87. Berger J, Suzuki T, Senti K-A et al. Genetic mapping with SNP markers in Drosophila. Nat Genet. 2001;29:475-481.

88. Wicks SR, Yeh RT, Gish WR et al. Rapid gene mapping in *Caenorhabditis elegans* using a high density polymorphism map, Nat Genet. 2001;28:160-164.

89. Cao J, Schneeberger K, Ossowski S et al. Whole-genome sequencing of multiple *Arabidopsis thaliana* populations, Nat Genet. 2011;43:956-963.

90. Porubsky D, Eichler EE. A 25-year odyssey of genomic technology advances and structural variant discovery, Cell 2024;187:1024-1037.

91. Ebert P, Audano PA, Zhu Q et al. Haplotype-resolved diverse human genomes and integrated analysis of structural variation, Science. 2021;372:eabf7117.

92. Liao W-W, Asri M, Ebler J et al. A draft human pangenome reference, Nature. 2023;617:312-324.

93. Porubsky D, Harvey WT, Rozanski AN et al. Inversion polymorphism in a complete human genome assembly, Genome Biol. 2023;24:100.

94. Porubsky D, Höps W, Ashraf H et al. Recurrent inversion polymorphisms in humans associate with genetic instability and genomic disorders, Cell. 2022;185:1986-2005.e26.

95. Liu YH, Luo C, Golding SG et al. Tradeoffs in alignment and assembly-based methods for structural variant

detection with long-read sequencing data, Nat Commun. 2024;15:2447.
96. Yang N, Liu J, Gao Q et al. Genome assembly of a tropical maize inbred line provides insights into structural variation and crop improvement, Nat Genet. 2019;51:1052-1059.
97. Goto H, Peng L, Makova KD. Evolution of X-degenerate Y chromosome genes in greater apes: conservation of gene content in human and gorilla, but not chimpanzee, J Mol Evol. 2009;68:134-144.
98. Gross JB, Wilkens H. Albinism in phylogenetically and geographically distinct populations of *Astyanax cavefish* arises through the same loss-of-function Oca2 allele, Heredity. 2013;111:122-130.
99. Jones FC, Grabherr MG, Chan YF et al. The genomic basis of adaptive evolution in threespine sticklebacks, Nature. 2012;484:55-61.
100. Schmutz S, Berryere T. Genes affecting coat colour and pattern in domestic dogs: a review, Anim Genet. 2007;38:539-549.
101. Tozaki T, Ohnuma A, Kikuchi M et al. Short insertion and deletion discoveries via whole-genome sequencing of 101 Thoroughbred racehorses, Genes. 2023;14:638.
102. Sudmant PH, Rausch T, Gardner EJ et al. An integrated map of structural variation in 2,504 human genomes, Nature. 2015;526:75-81.
103. Zhou B, He Y, Chen Y et al. Comparative Genomic Analysis Identifies Great–Ape–Specific Structural Variants and Their Evolutionary Relevance, Mol Biol Evol. 2023;40:msad184.
104. Prychitko TM, Moore WS, Evolution. Alignment and phylogenetic analysis of β-fibrinogen intron 7 sequences among avian orders reveal conserved regions within the intron, Mol Biol Evol. 2003;20:762-771.
105. Alonge M, Wang X, Benoit M et al. Major impacts of widespread structural variation on gene expression and crop improvement in tomato, Cell. 2020;182:145-161. e123.
106. Vialle RA, de Paiva Lopes K, Bennett DA et al. Integrating whole-genome sequencing with multi-omic data reveals the impact of structural variants on gene regulation in the human brain, Nat Neurosci. 2022;25:504-514.
107. Fulton Lucinda A. MER, Wilson Richard K. Sequence and comparative analysis of the chicken genome provide unique perspectives on vertebrate evolution, Nature. 2004;432:695-716.
108. Razaq A, Khan A, Shah ST et al. Association of insertion/deletion polymorphism of ace gene with essential hypertension in patients of Khyber Pakhtunkhwa, Pak J Med Sci. 2024;40:461.
109. Xiao Y, Xiao Z, Ma Y et al. Datasets and markers development workflow run scripts for high-throughput exploitation of genetic sex markers, figshare 2023. https://doi.org/10.6084/m9.figshare.24647598.v3
110. Xiao YS; Xiao ZZ; Ma YT et al. Supporting data for "Innovative approach for high-throughput exploiting sex-specific markers in Japanese parrotfish *Oplegnathus fasciatus*" GigaScience Database. 2024. https://doi.org/10.5524/102548.

 Click here to access/download;Figure;Figure Legends.docx

Figure 1: High-throughput exploitation process of genetic sex markers for Japanese parrotfish.

Figure 2: Distribution statistics of insertion and deletion fragment (＞100bp) positions in genomic functional regions of Japanese parrotfish.

(a) Distribution statistics of insertion variant loci using the female fish genome as a reference. (b) Distribution statistics of insertion variant loci utilizing the male fish genome as a point of reference. (c) Distribution statistics of deletion variant length using the female fish genome as a reference. (d) Distribution statistics of deletion variant loci using the male fish genome as a reference.

Note: The colors red, green, yellow, and orange represented the UTR, CDS, intron, and intergenic regions. The frequency of insertions (a, b) was quantified based on the number of events, while deletions (c, d) were characterized by their length in nucleotides.

Figure 3: The covariance of genomic sequence differences between the male and female genomes of Japanese parrotfish and distribution statistics of variant sites.

(a) The female genome (reference) of *Japanese parrotfish* vs. the male genome for homology comparison using the minimap2 software with a 5,000 bp sliding window. The female genome was the vertical axis, and female chromosomes 8 and 10 were homologous to male chromosome 9. (b) The comparison of homology between the male genome of *Japanese parrotfish* and the female genome was conducted using the minimap2 software with a 5,000 bp sliding window. The male genome was represented on the vertical axis, revealing homology between male chromosome 9 and female chromosomes 8 and 10. (c) Distribution statistics of variant loci using the female fish genome as a reference. Insertions (>100 bp) were summarized by number of events. Deletions (>100 bp) were summarized by length in nucleotides. From outer to inner circles were the chromosome name, positional distribution of insertion sites, base length distribution of deletion sites, and base substitution distribution, respectively. (d) Distribution statistics of variant loci using the male fish genome as a reference. (e) - (f) Dual-valued histograms corresponding to the Table 3 showed the number of insertion/deletion sites per chromosome with reference to the female (4,422 inertion sites, 4,675 deletion sites) and male (4,615 inertion sites, 4,385 deletion sites) genomes, respectively, where chromosome 9 in males was homologous to chromosomes 8 and 10 in females. Insertion sites were represented by negative values, and deletion sites were

represented by positive values.

Figure 4: Intersection results of two variant site scanning methods and ranking of variant sites with long read depth frequency distributions

(a) Venn results of deletion locus data were obtained based on TBtools and SyRI software using the female fish genome as a reference, and 1,791 out of 2,116 deletion loci obtained based on SyRI software were shared deletion variant loci. (b) Utilizing the male fish genome as a point of reference for acquiring deletion site data in the context of Venn analysis, it was found that 1,749 out of the 2,045 deletion sites identified through the use of the SyRI software were common deletion variant sites. (c) Depth distribution of CLR reads in male fish using all female genomic deletion locus (＞100 bp) information as a reference, with the area where high-quality male and female genetic marker information was located before the orange labeled trough position (~5.4). (d) The depth distribution of CLR reads in females was analyzed by utilizing male genomic deletion sites as a reference (>100 bp), focusing on the region preceding the orange labeled trough position (~12.5) where high-quality male and female genetic marker information was present. (e) - (f) Depth distribution of CLR reads in males and females based on intersecting male and female genomic deletion site information (>100bp), respectively.

Figure 5: Representative genetic sex marker (insertion deletion variant sites) located in the intergenic region (IR), Intronic region (IN) of the Japanese parrotfish genome (STOSOd393e).

(a) Location of male and female sex markers in the male (MChr9) and female (FChr8) genomes. (b) Male and female sex genetic markers were located at loci in the intergenic region, using the female fish genome as a reference (Figure S2). Illumina and CLR clean data of bam comparison to the genome for insertion/deletion site validity detection. Pink and blue data from PacBio represented the same and opposite orientation of female genome and gray represented Illumina clean data. The e-PCR amplified bands were 390 bp and 989 bp in size, corresponding to DNA insertion and deletion patterns and agarose gel electrophoresis for identification of male and female genetically different populations. (c) By utilizing the female genome as a point of comparison, the genetic markers for male and female sexes were identified at specific insertion sites within *nuf2* gene introns (Figure S3, Figure S4). The resulting e-PCR amplified bands, measuring 205 bp and 749 bp in size, were indicative of distinct DNA

insertion patterns and were utilized in agarose gel electrophoresis for the purpose of distinguishing between male and female genetically differentiated groups. Males exhibited two amplified bands, while females exhibited only one. (d) By utilizing the female fish genome as a point of comparison, the genetic markers for male and female sexes were situated within the deletion sites located in the introns of genes (Figure S5, Figure S6). The amplified bands produced through e-PCR were observed to be 558bp and 928 bp in length, aligning with the DNA deletion patterns and agarose gel electrophoresis maps utilized for distinguishing genetically distinct groups of males and females. Specifically, two bands were amplified in males, while only one band was observed in females.

Figure1
Click here to access/download;Figure;Figure 1.tif

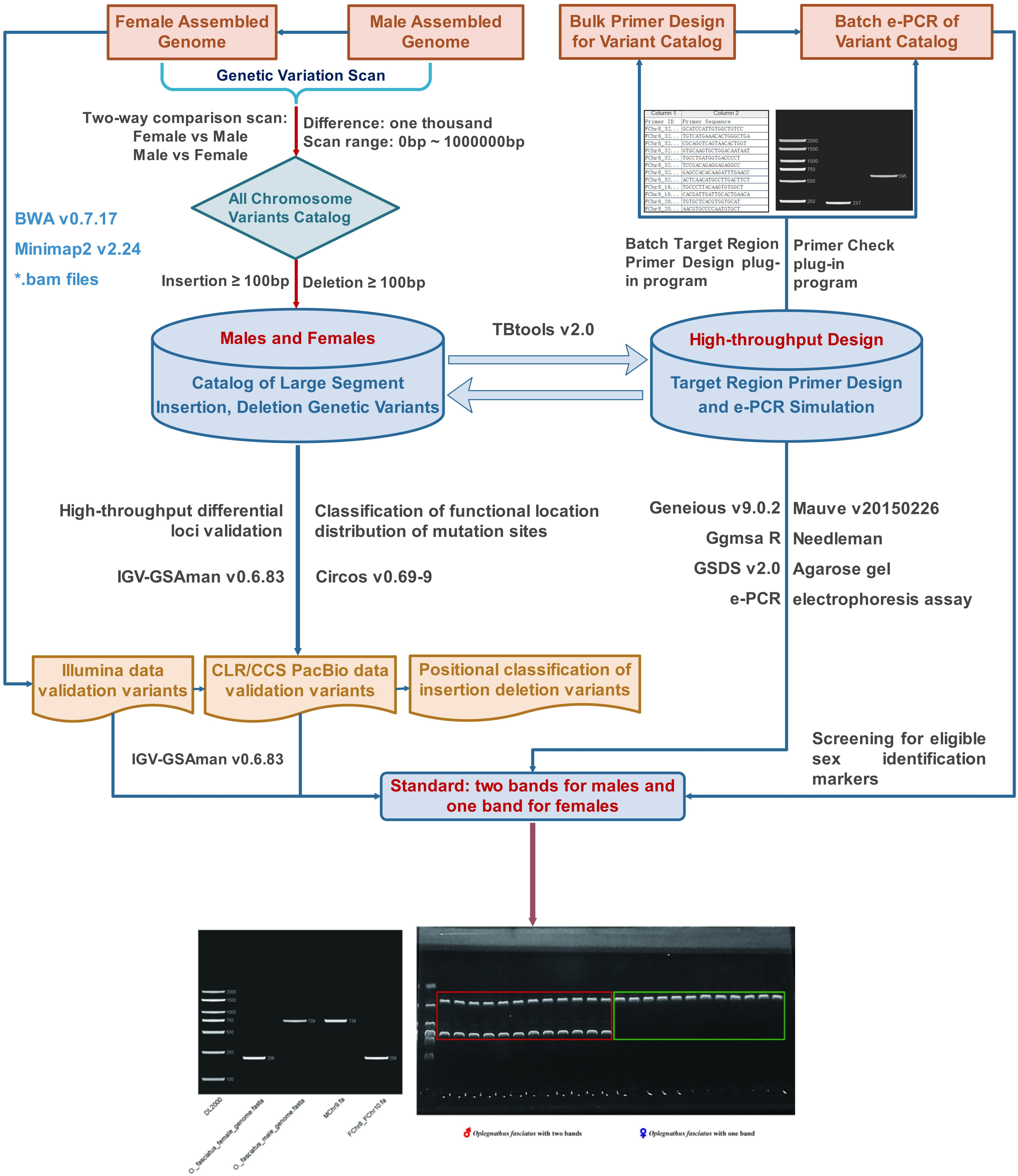

Click here to access/download;Figure;Figure 2.pdf

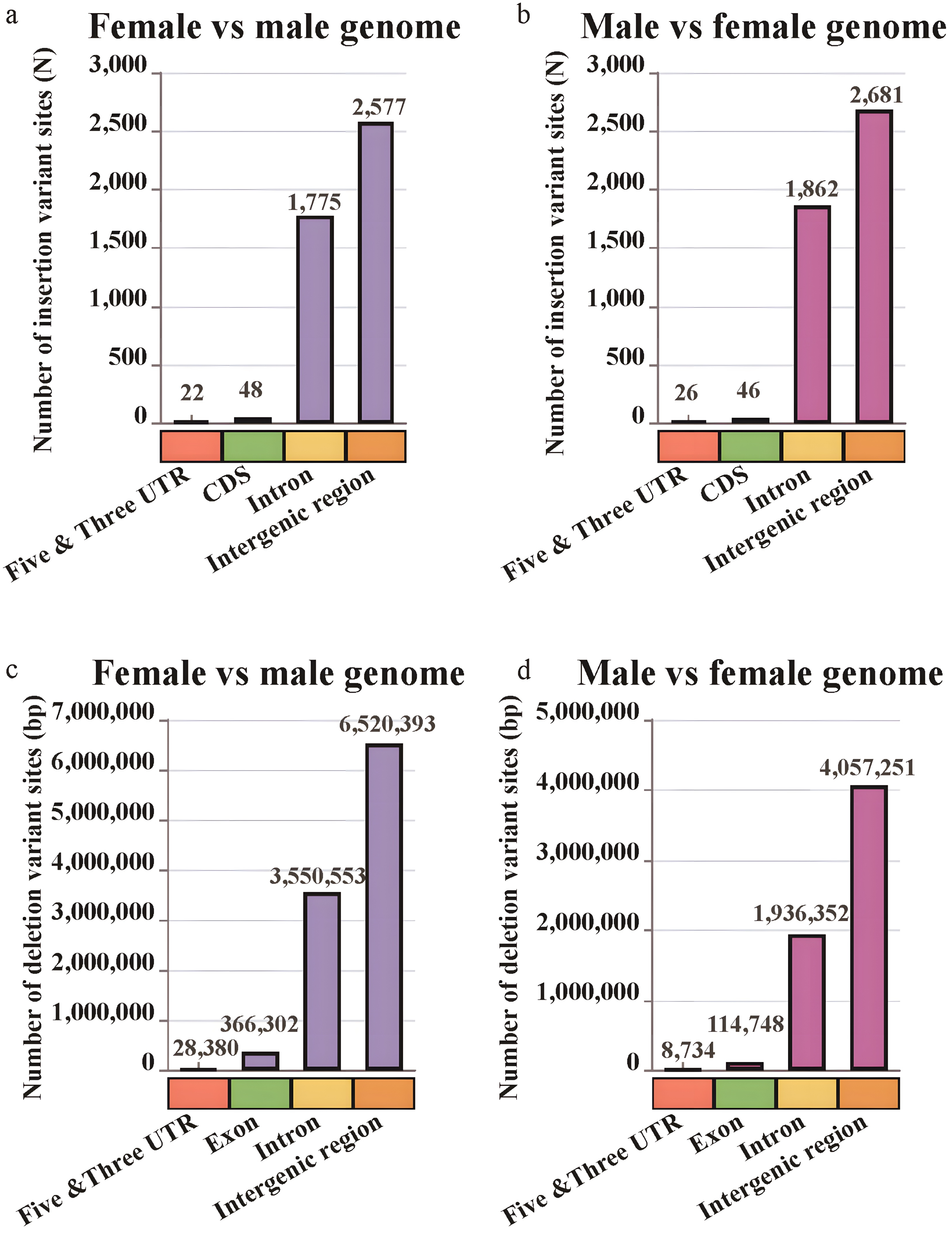

Figure3

Click here to access/download;Figure;Figure 3.pdf

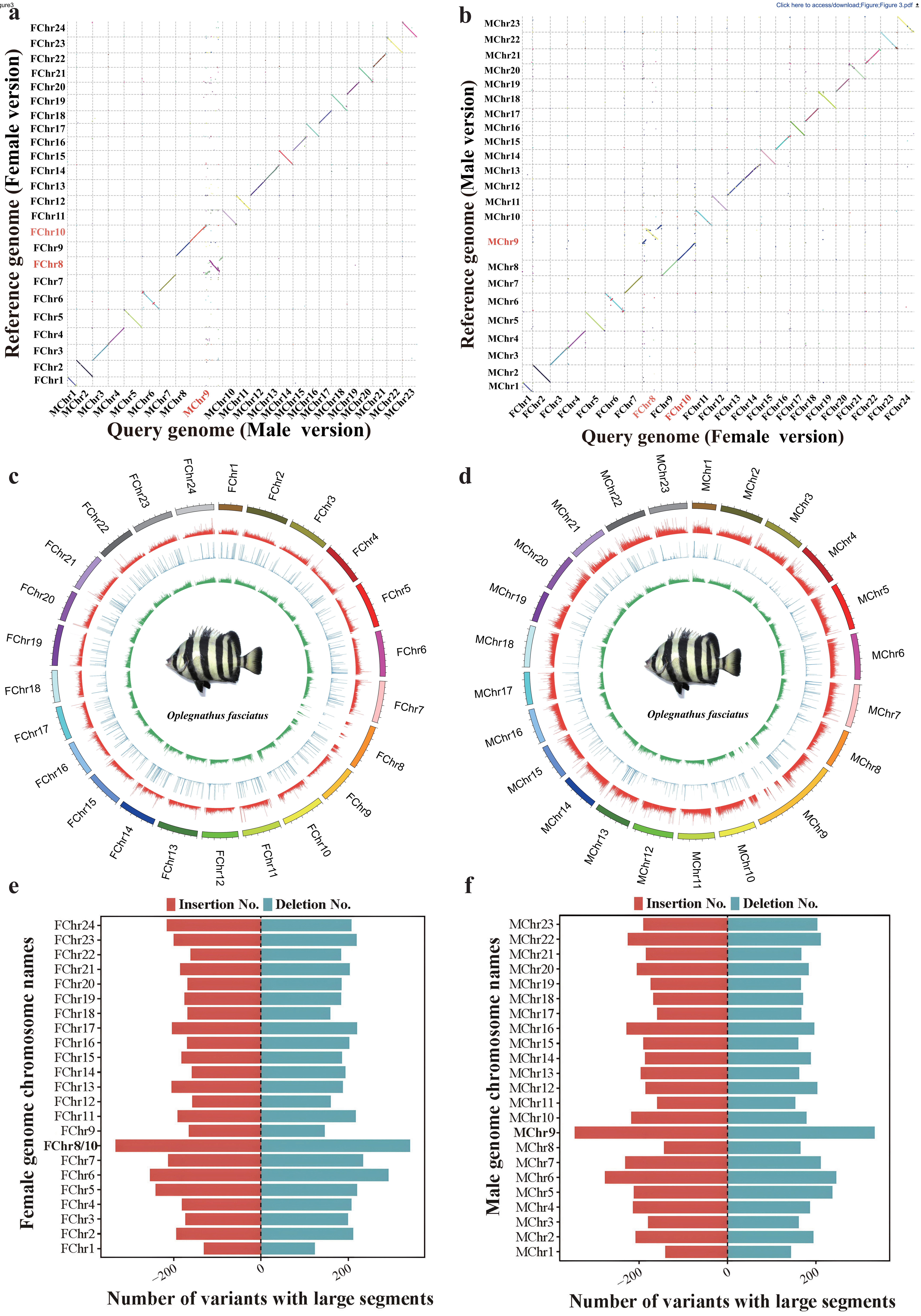

Figure4 Click here to access/download;Figure;Figure 4.pdf

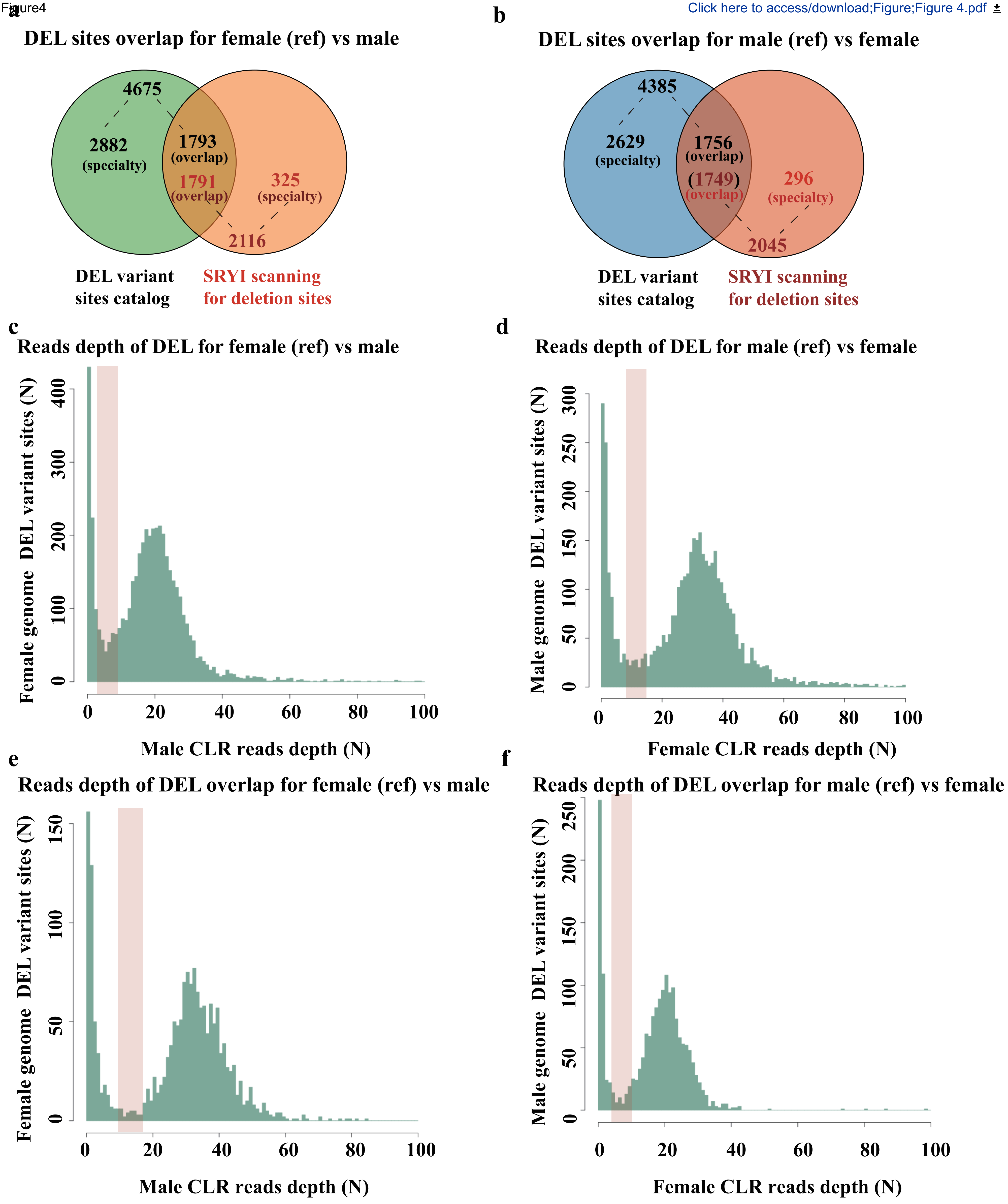

Click here to access/download;Figure;Figure 5.pdf

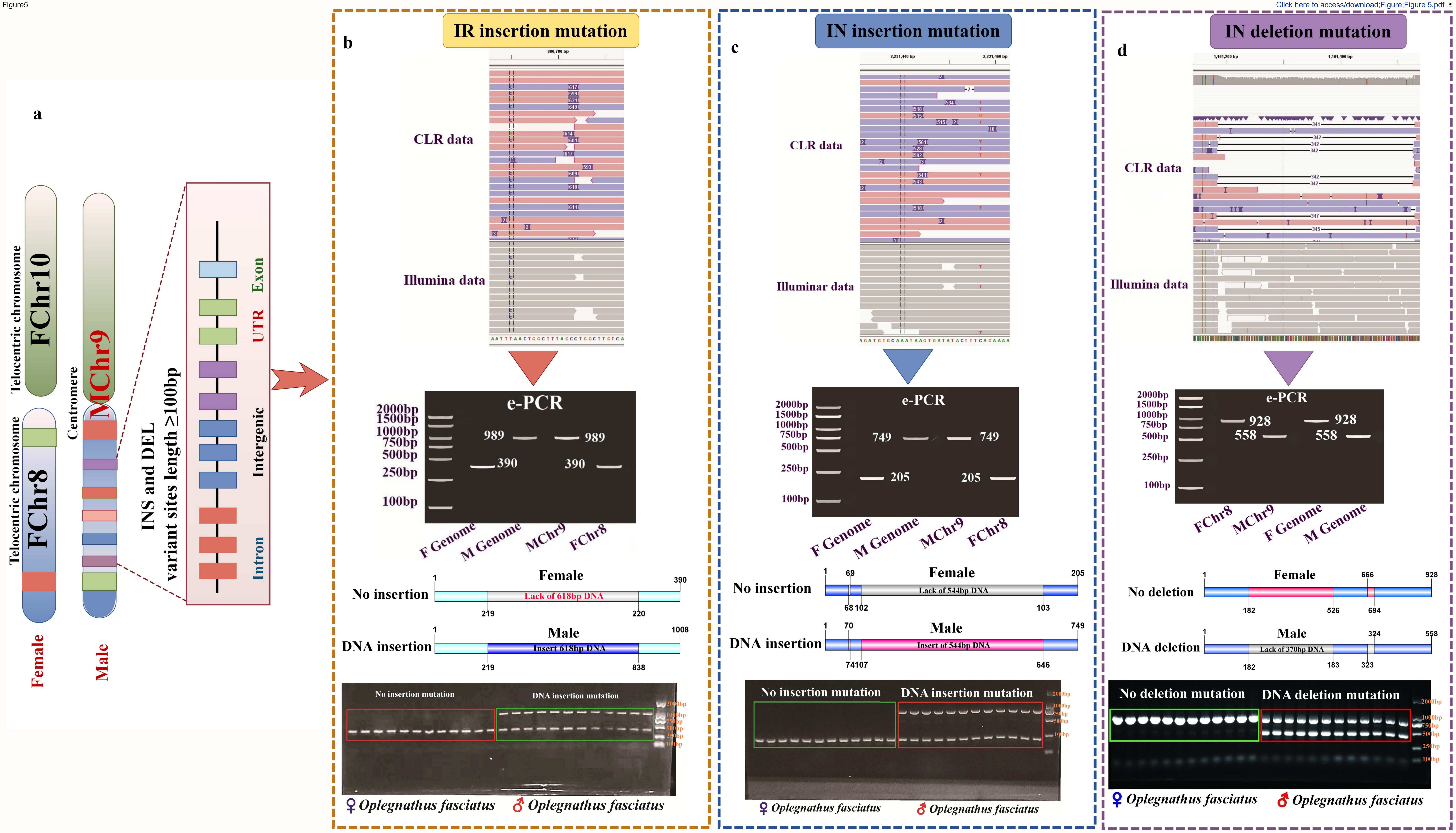

FigureS1

Click here to access/download;Figure;Figure S1.pdf

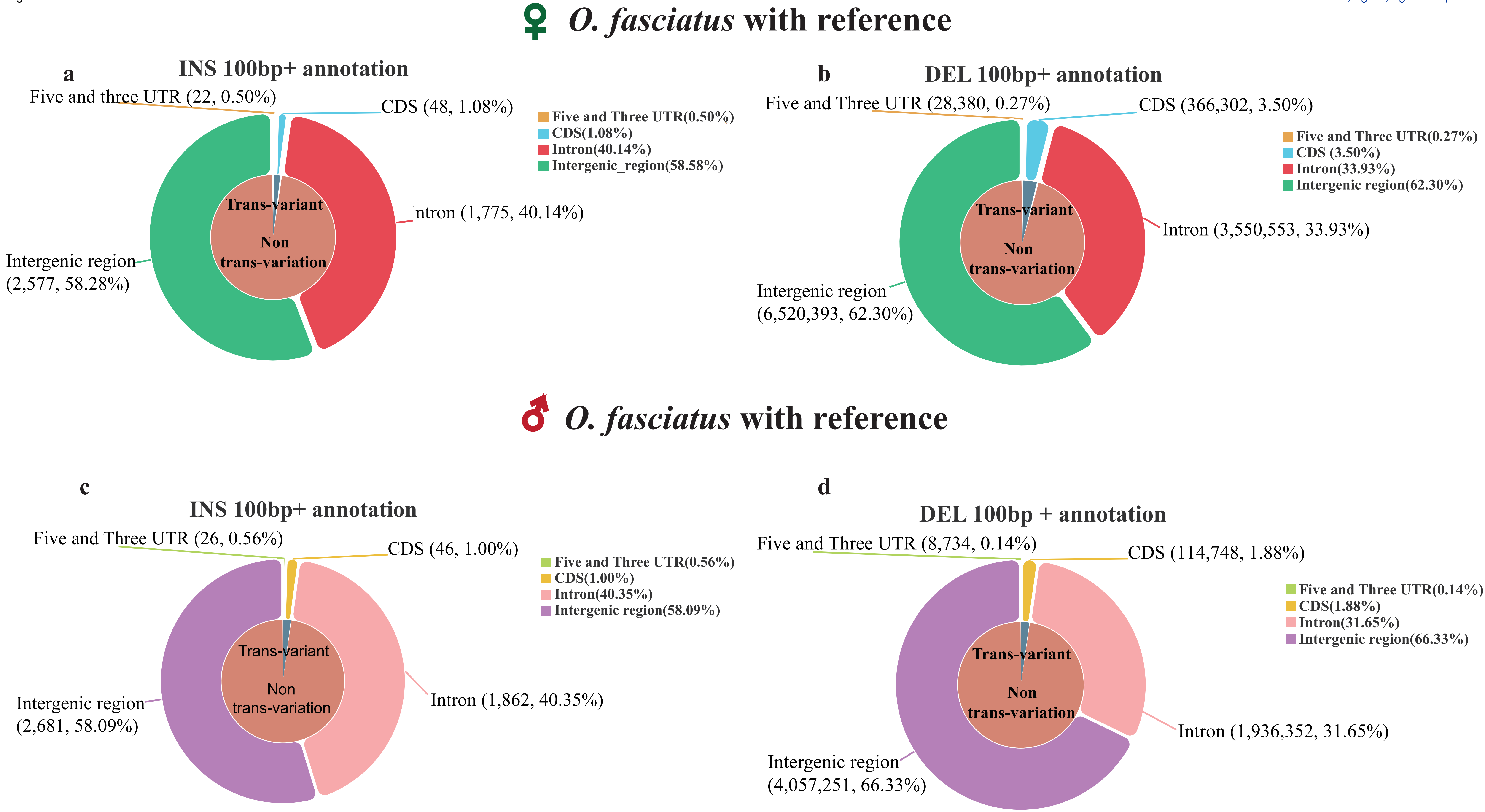

FigureS2

Click here to access/download;Figure;Figure S2.pdf

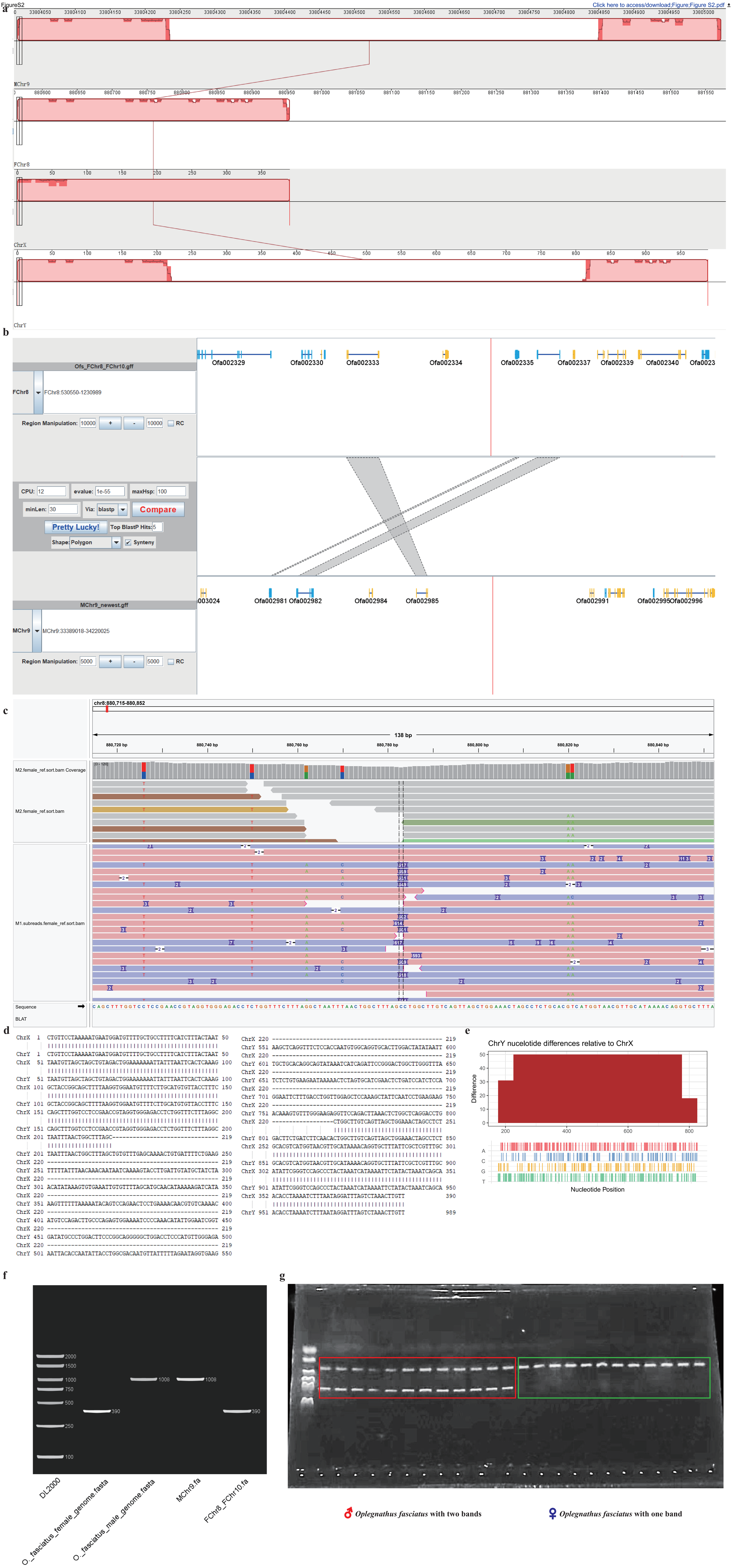

FigureS3

Click here to access/download;Figure;Figure S3.pdf

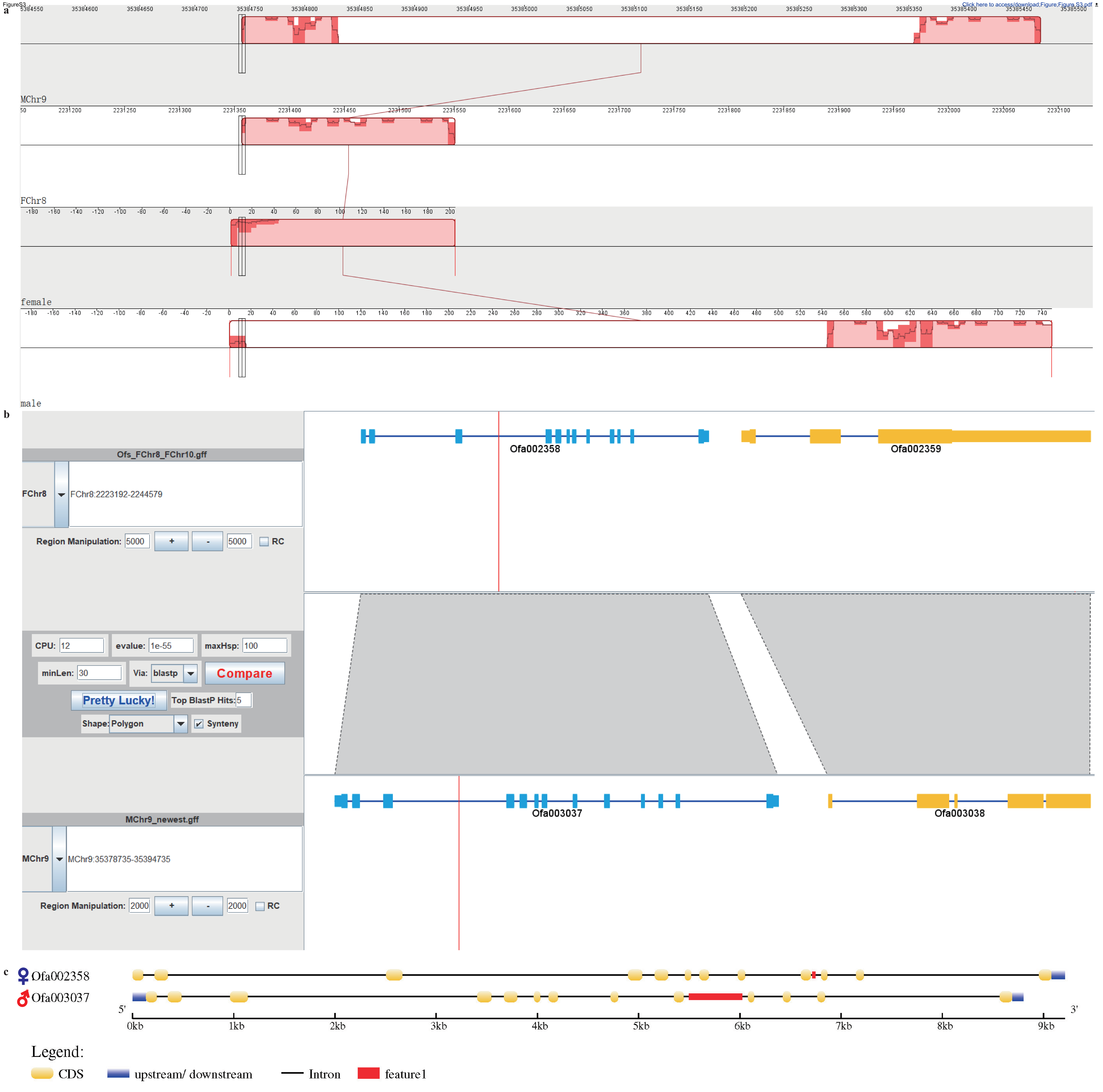

FigureS4

Click here to access/download;Figure;Figure S4.pdf

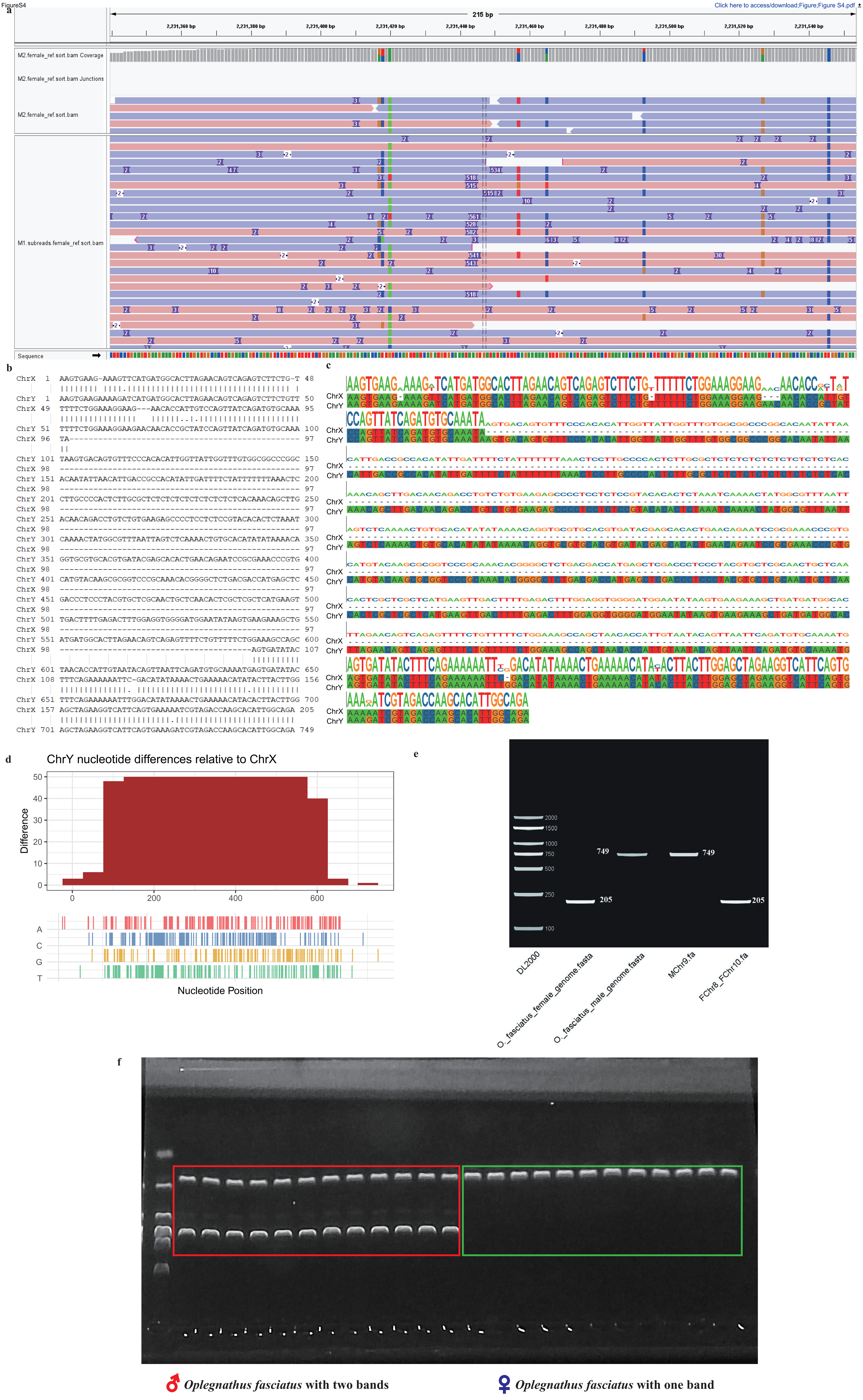

 Click here to access/download;Figure;Figure S5.pdf

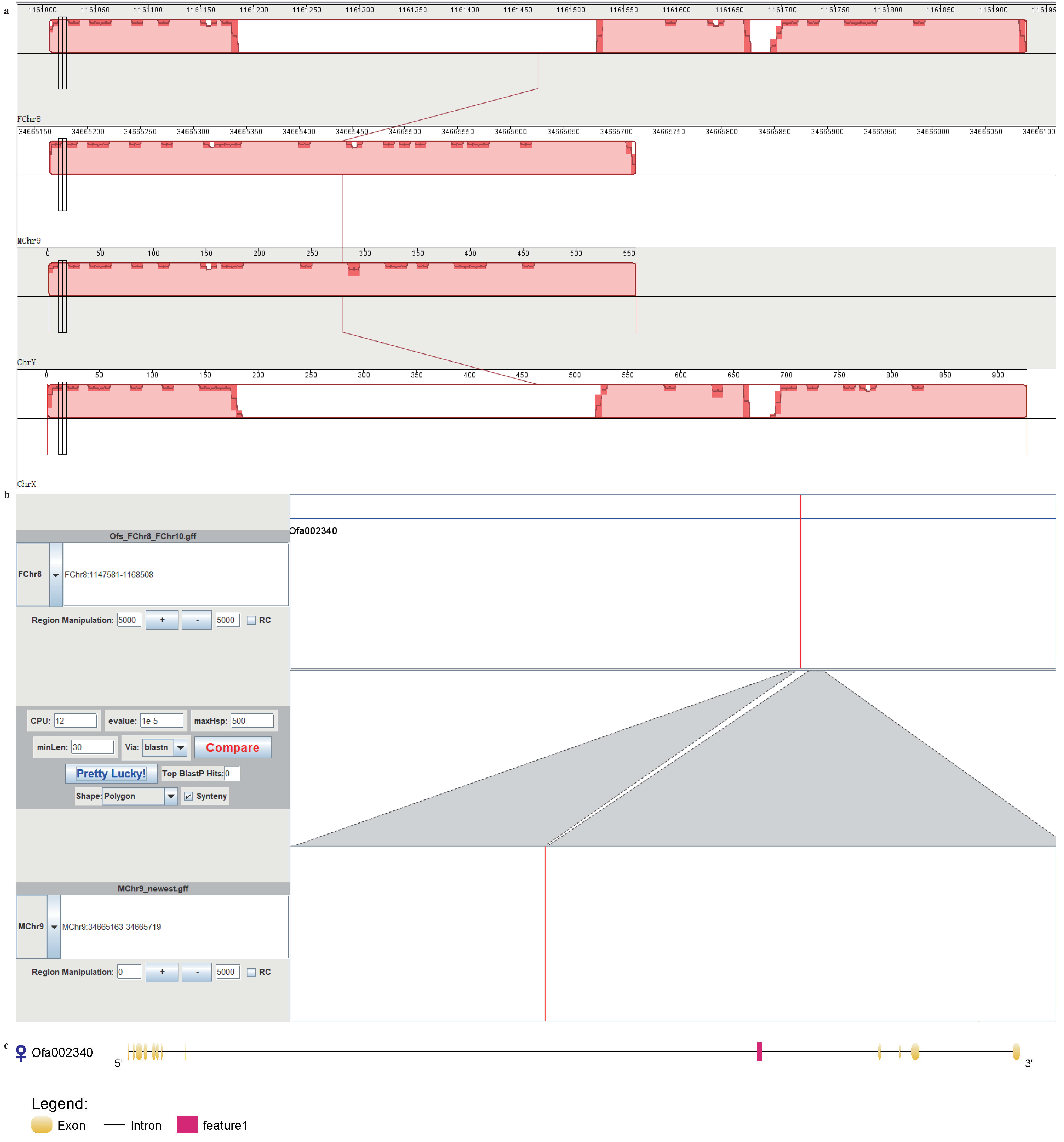

FigureS6
Click here to access/download;Figure;Figure S6.pdf

a

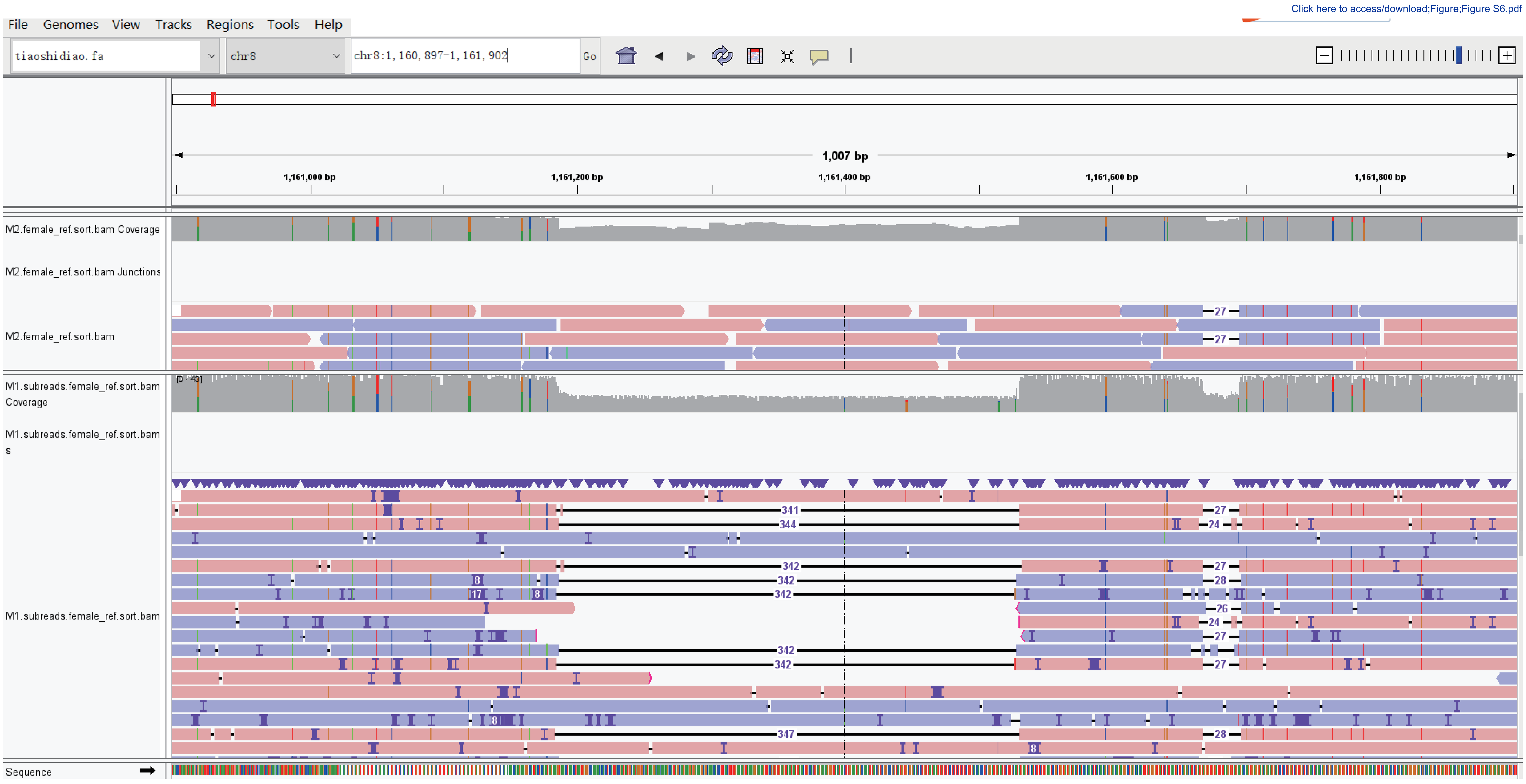

b

```
ChrY   ATGAAAGCCCGGAAAAATCACAAGCGGCACAGAGTTTGGCTTTTAATGCAACAGACTCGC
ChrX   ATGAAAGCCCAGAAAAATCACAAGCGGCGCAGAGTTTGGCTTTTAACGCAACAGACTTGC
       ********** ***************** ***************** ********** **

ChrY   TCTTCAACTCACCTGTGTGTGAAGCGGCGTACTGTTTTTTATGGTCTCTGTTAGCGTTGT
ChrX   TCTTCAACTCACCTGTGTGTGAAGCGACGTACTGTTTTTTATGGTCTCTGTTAGCATTGT
       ************************** **************************** ****

ChrY   GAGTGAACGGTTACTATGGCCGAACAAACAAAGAGAAGGTAAATAACTACAGACGAGGAA
ChrX   GAGTGAACGGTTACTATGGCCGAACAAACAAAGAAAAGGTCAATAACTACAGATGAGGAA
       ********************************** ***** ************ ******

ChrY   AG----------------------------------------------------------
ChrX   AGTGAAACTGAAACTTAAGTGAGGTCCAAAAACACACCTGCAATTACCGATTATTGCCAT
       **

ChrY   ------------------------------------------------------------
ChrX   AGGTGCCATGAAAGACAACAAAAGCCAGTAAGCCAGTAAAGATGAAAAGGCAGCAAAACA
ChrY   ------------------------------------------------------------
ChrX   TCCTTTCACTTTTTGGAACAGCTAAAAAGGTAAAAGTTTACGATGATGTAGATAAATAAT
ChrY   ------------------------------------------------------------
ChrX   GTAAACTTGCCACTGCAGGAACAGTGGTTACATAACCTTCGAAATGAGCCACAACTTAAC
ChrY   ------------------------------------------------------------
ChrX   CGTGCGCGCACGCGTCATAGTTGCGGGACATCCCTGATTAATAAAGTTATTCTTCCTCTT
ChrY   ---------------------------------------------AATGAAGTCTCATCT
ChrX   CTTCTTTCTGTGCACCACCCTGCGAGATTGTAAATTTCTTTGCAATATGAAGTCTCATCT
                                                     **************

ChrY   TTCTTTGAAATCCGGCTGGTGATGATAGCATCATCCACAAACTTCACAACACCGTGAACA
ChrX   TTCTTTGAAATCCGGCTGGTGATGATAGCATCATCCACAAACTTCACAACAGCGTGAACA
       *************************************************** ********

ChrY   AGAAGGGCCTGAGCACACATTTTATGGGGTTGATTGTGTTAAATGCTTCAGAGCATTCTG
ChrX   AGAAGGGCCTGAGCACACATTTTATGGGGTTGATTCTATTAAATGCTTCAGAGCATTCTG
       *********************************** * **********************

ChrY   ATGT---------------------------AGACCGAGTGGAGGGTAGTAGAAATGGCA
ChrX   ATGTAGGTGTTGTGATTTTTAAGGTGTATGAAGACCAAGTGGAGGGTAGCAGAAATGGCA
       ****                           ***** ************ **********

ChrY   TCCTCCTTGGATCTGTTGGCTCTGAACTCAAACAGGCTGGCTGGGATGTGGTCTTTTATG
ChrX   TCCTCCTCGGATCTGTTGGCTCTGAACTCAAACAGGCTGGCCGGGATGTGGTCTTATATG
       ******* ********************************* ************* ****

ChrY   TGGTTGAGAACCAGTTTCTCAAAGCACTTCATCAGAATGGGGGTGAGTGCCACAGGGCAG
ChrX   TGGTGGAGAACCAGTTTCTCAAAGCACTTCATCAGAATGGGGGTGAGCGCCACAGGGCAG
       **** ****************************************** ************

ChrY   TAGAGTGTTAGACTAGACACTGCAGACTTTTTAGGTACAGGATGATGGTGACTGTCTTG
ChrX   TAGAGTGTTAGACTAGACACTGCAGACTTTTTAGGTACAGGATGATGGTGACTGTCTTG
       ***********************************************************

ChrY   AAGCAGGTGGTAATACTCTCCTGTGACA
ChrX   AAGCAGGTGGTAATACTCTCCTGTGACA
       ****************************
```

c

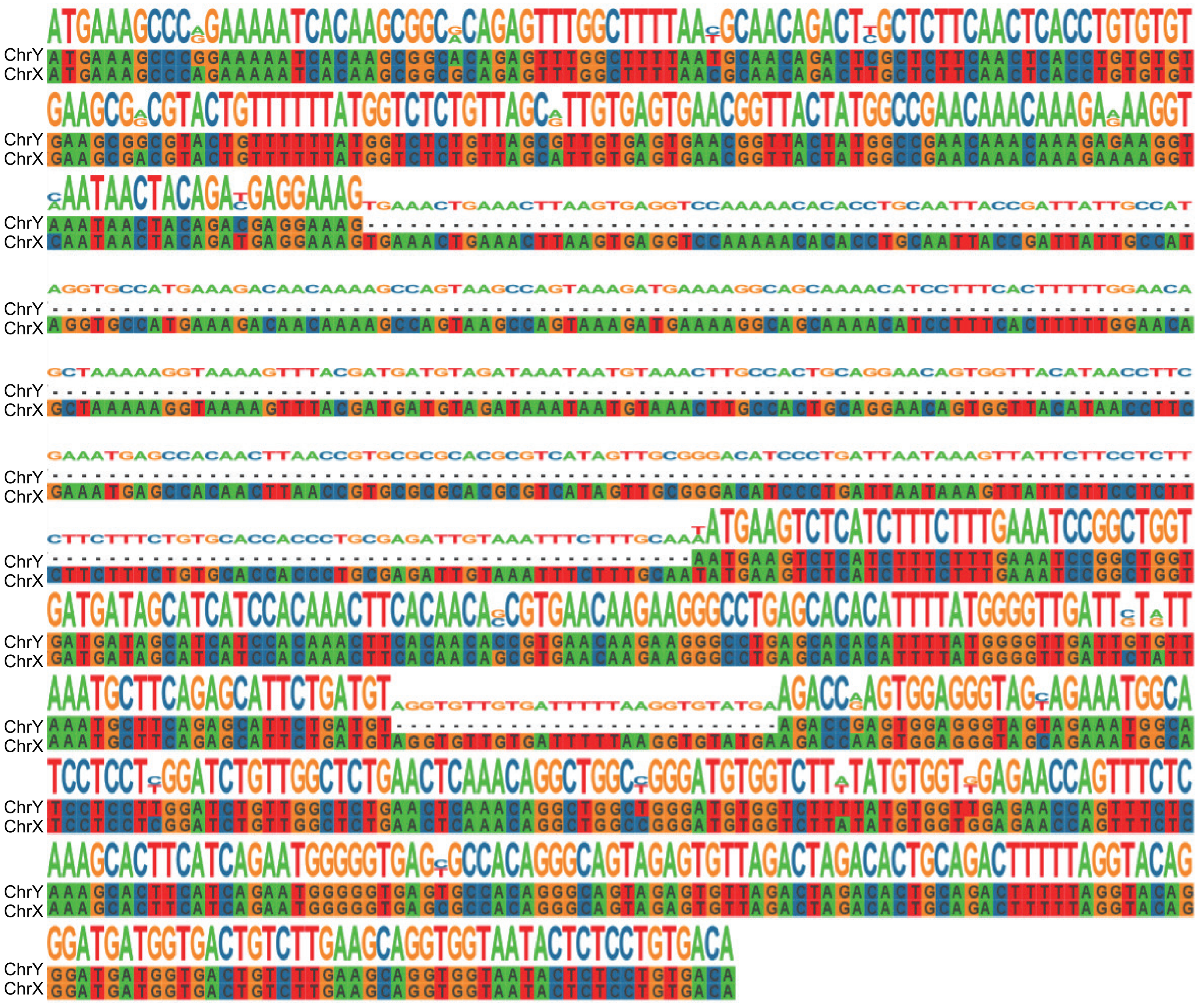

d

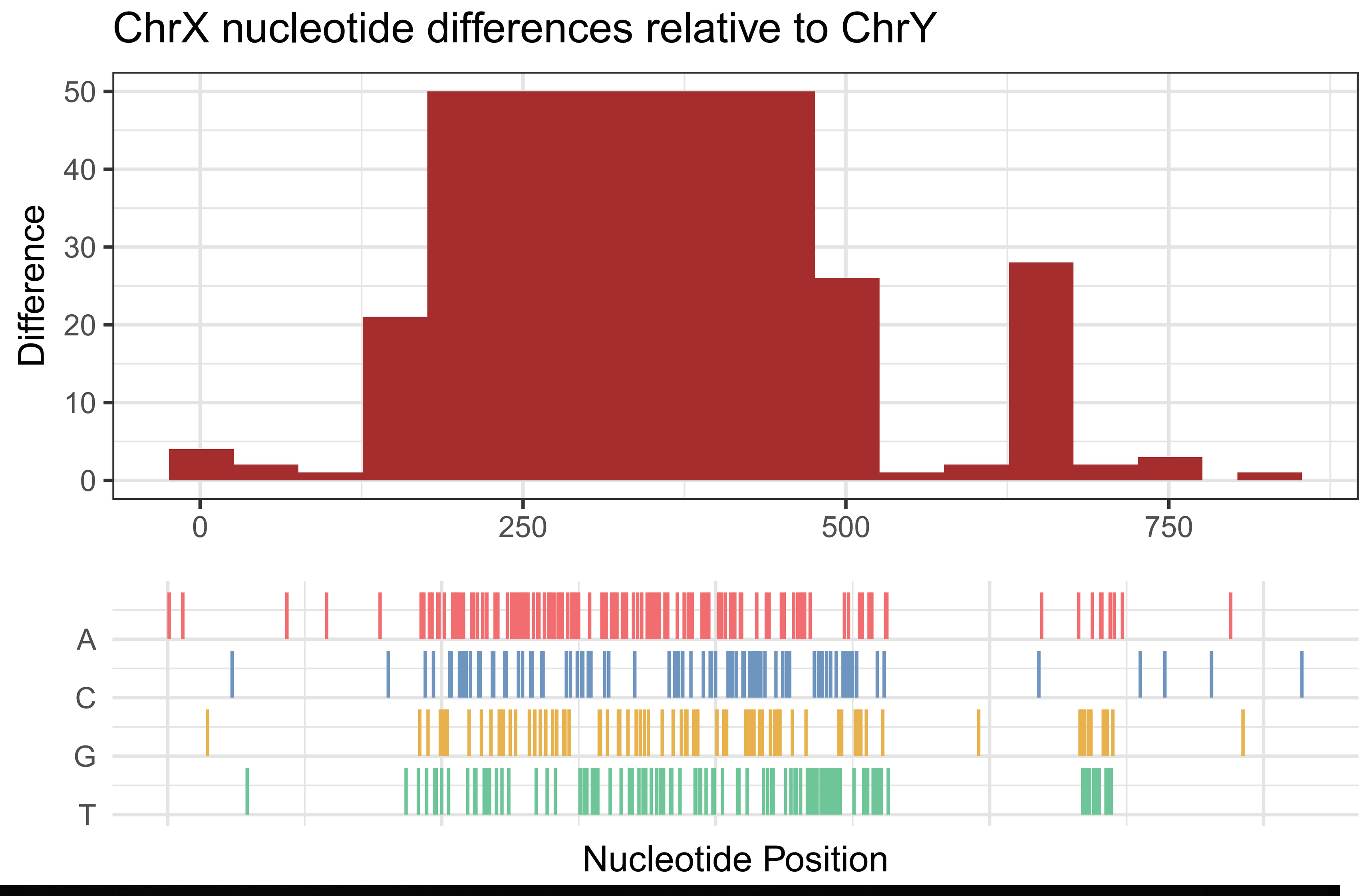

e

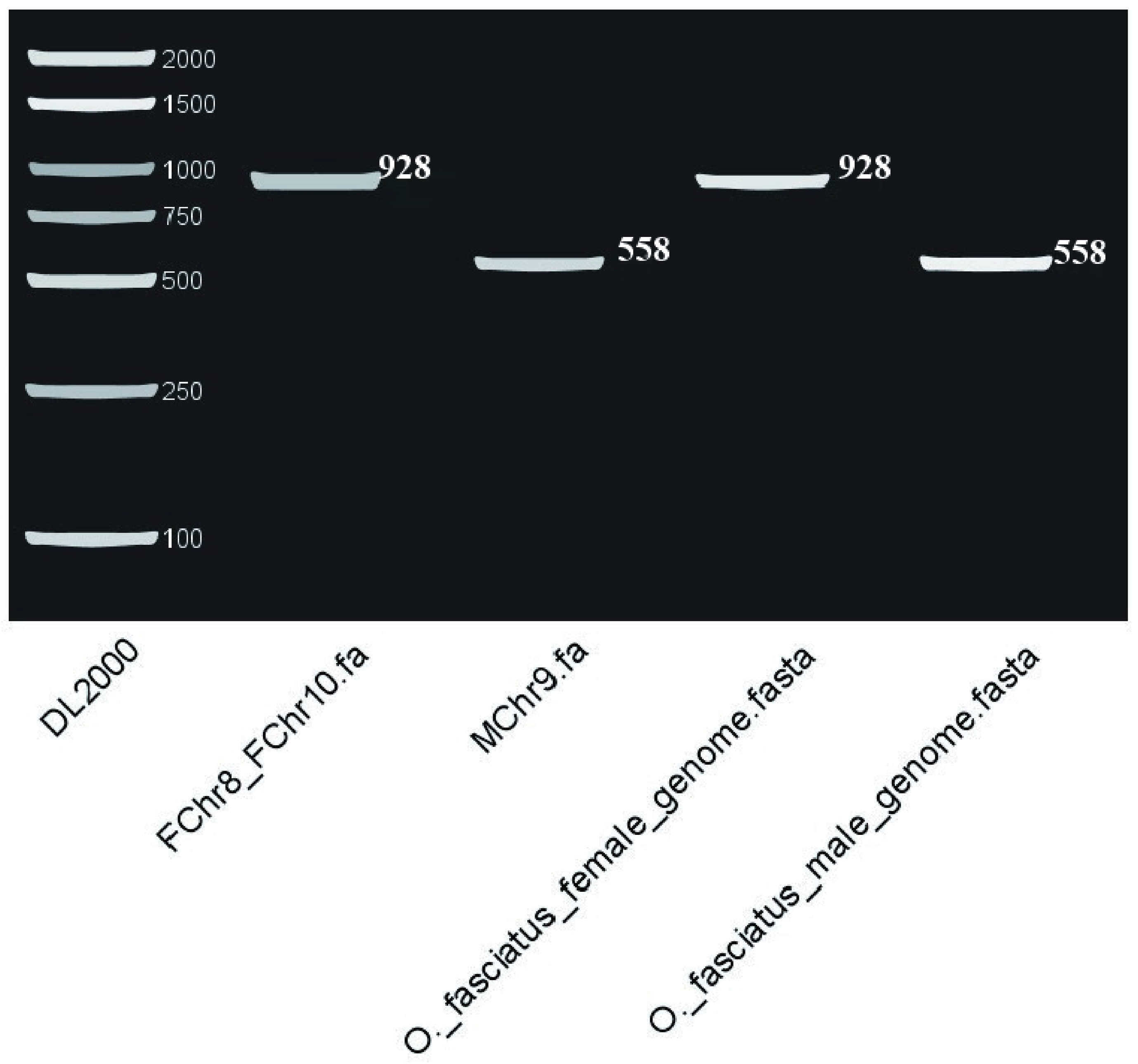

f

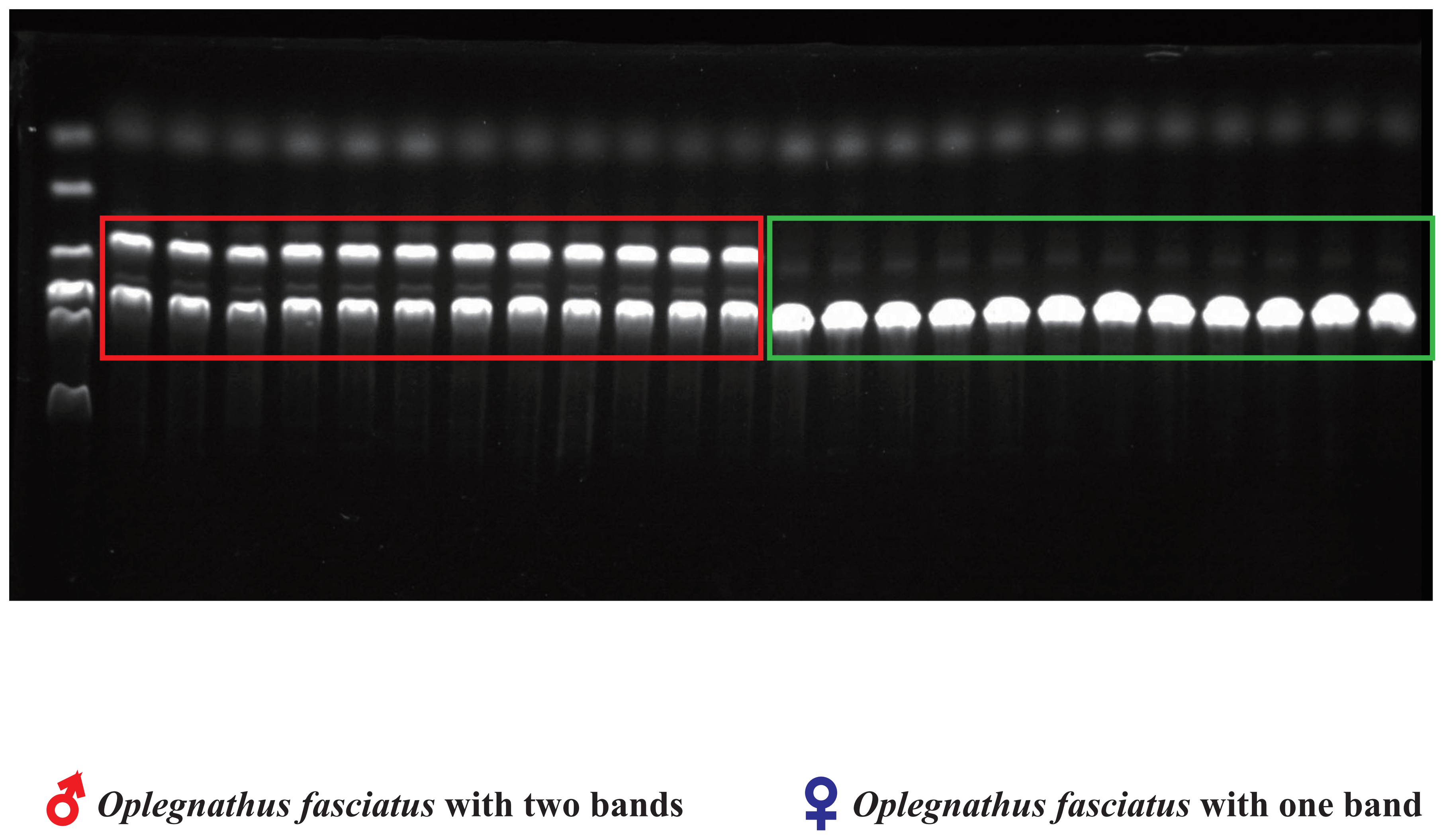

Click here to access/download;Figure;Figure S7.pdf

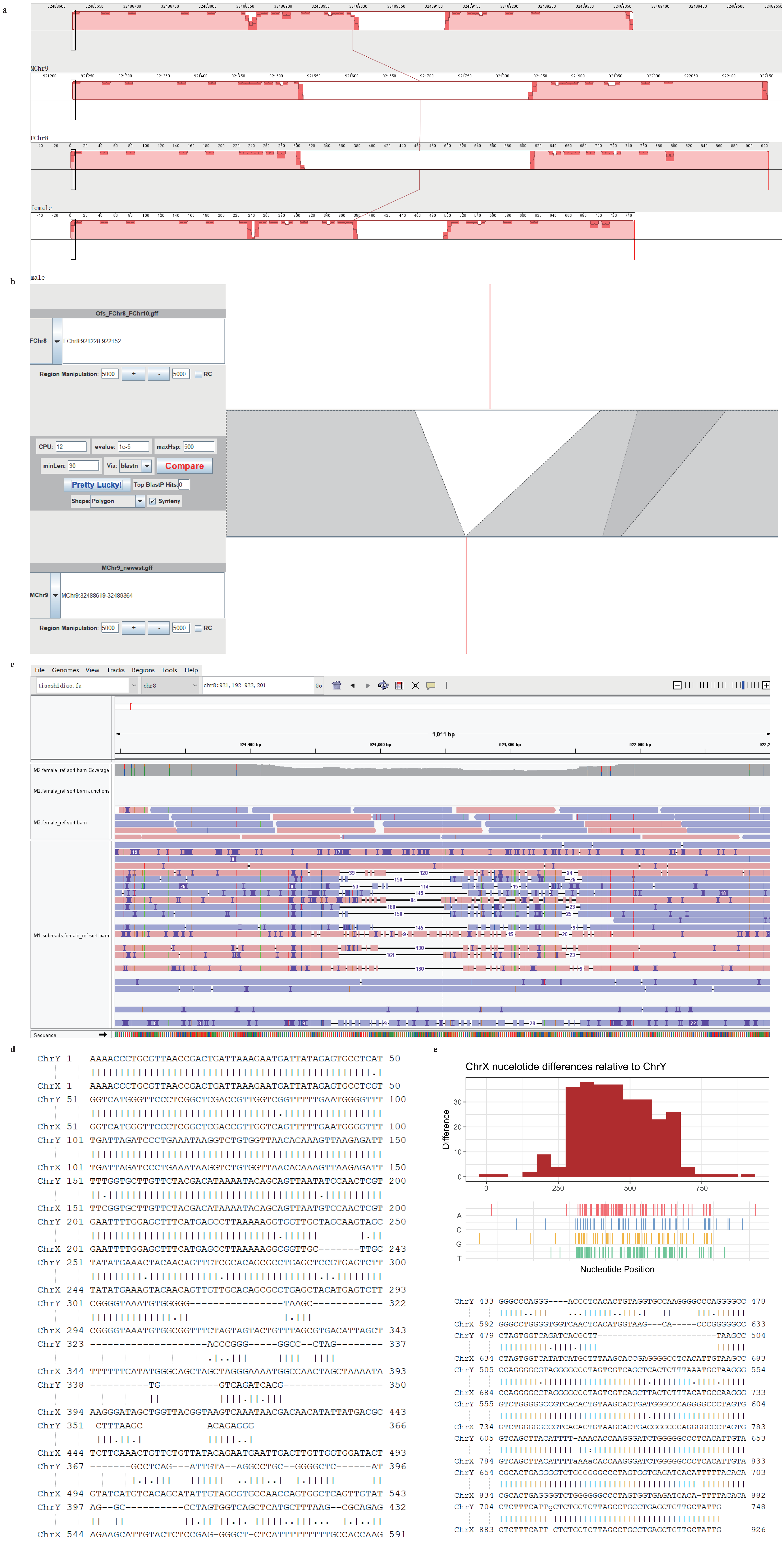

Click here to access/download;Figure;Figure S8.pdf

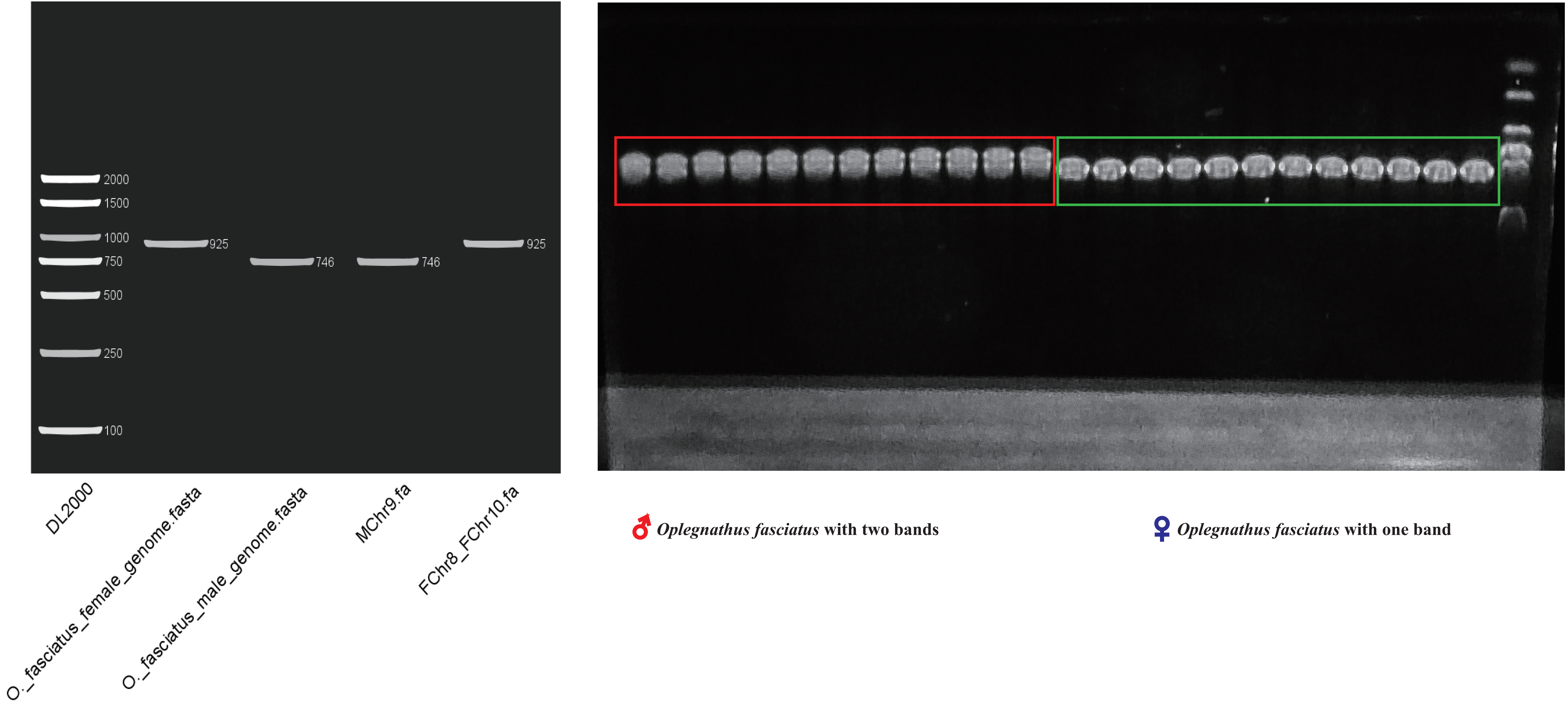

FigureS9

Click here to access/download;Figure;Figure S9.pdf

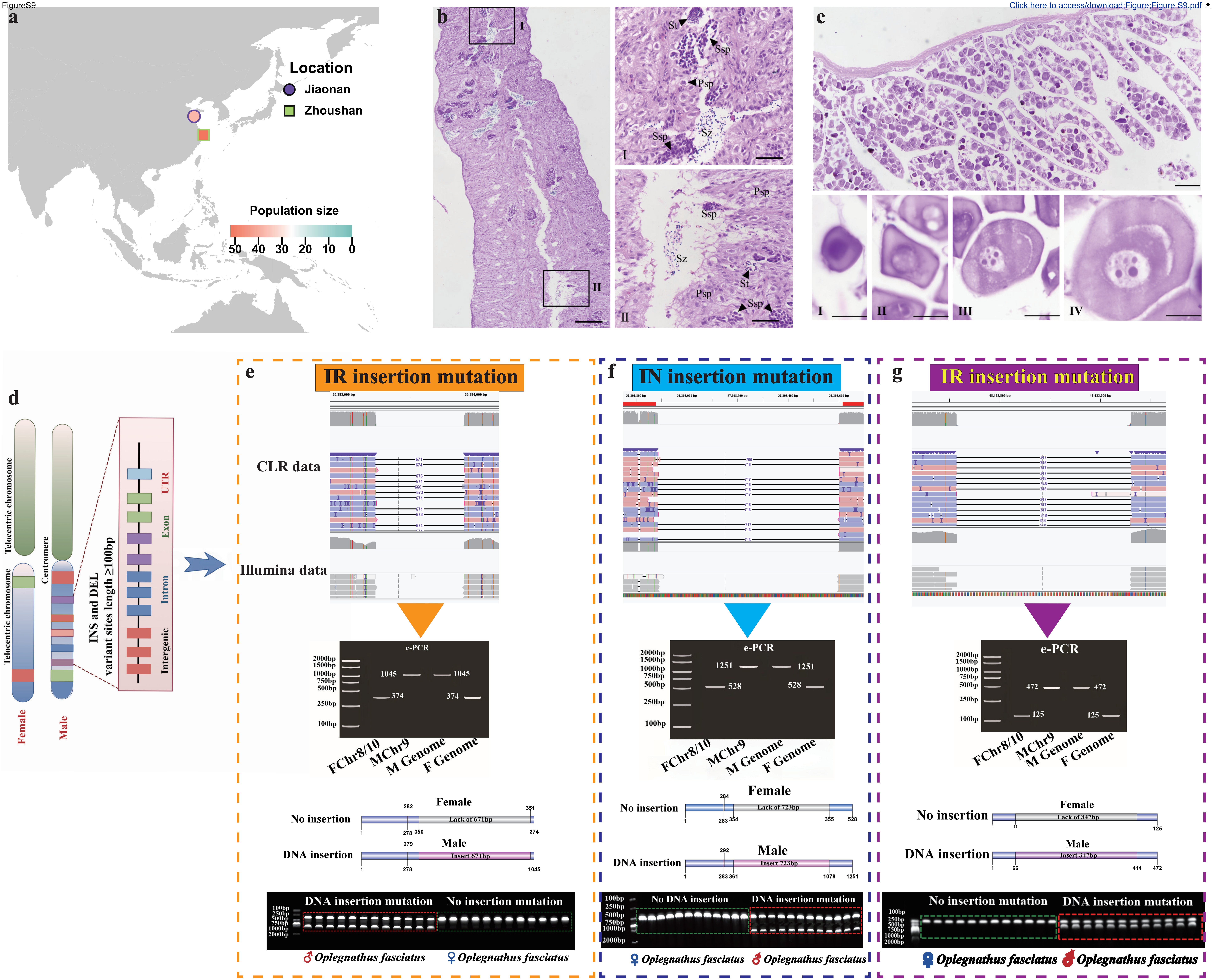

Click here to access/download
Supplementary Material
Supplementary information.docx

Click here to access/download
Supplementary Material
new-Female_reference_all_variation_Set.xls

Click here to access/download
Supplementary Material
new-Male_reference_all_variation_Set.xls

Click here to access/download
Supplementary Material
new-Female_reference_ INS_DEL 100_Set.xlsx

Click here to access/download
Supplementary Material
new-Male_reference_ INS_DEL 100_Set.xlsx

Click here to access/download
Supplementary Material
new-Female_reference_ INS_DEL
100_ExonSet_annotation.xlsx

Click here to access/download
Supplementary Material
new-Male_reference_ INS_DEL 100_ExonSet_annotation.xlsx

Click here to access/download
Supplementary Material
SyRI_Female_Reference_male_Get_all_DEL_100bp.xlsx

Click here to access/download
Supplementary Material
SyRI_Male_Reference_female_Get_all_DEL_100bp.xlsx

Click here to access/download
Supplementary Material
Female_reference_Male_DEL_100p.ann.with_CLR_read
s_depth.xls

Male_reference_Female_DEL_100p.ann.depthwith_CLR_reads_de
pth

Click here to access/download
Supplementary Material
Male_reference_Female_DEL_100p.ann.depthwith_CLR
_reads_depth.xls

Click here to access/download
Supplementary Material
Female_reference_Male_DEL_repeat.txt

Click here to access/download
Supplementary Material
Female_reference_Male_DEL_repeattype.txt

Click here to access/download
Supplementary Material
Male_reference_Female_DEL_repeat.txt

Click here to access/download
Supplementary Material
Male_reference_Female_DEL_repeattype.txt

Click here to access/download
Supplementary Material
new-Female_Male_INSD_EL100_PrimerSet.xlsx

Reviewer reports:

Reviewer #1: The revised manuscript is significantly improved and the authors have done a great job addressing all my previous comments. I appreciate authors responses and revisions to all points.

Reply:

Thanks for your previous comments on our manuscript, which have been very helpful in improving the quality of our manuscript.

Reviewer #2: Dear colleagues,

Thank you very much for the thorough, extensive, cautious and precise replies to the previous round of comments that I provide, as well as that of the two other reviewers in my opinion.
I just have a list of minor comments to provide.

Best regards,

Being a non-native English speaker, please double-check my comments below.

Minor comments:

In the first paragraph of the new version (starting line 125), the use of the present tense was better than that of the actual paste tense (also line 195, the use of "was" would mean that the species no longer exists in this geographical area). L228, it is still the same sex chromosome system. Line 683, etc. Same for the Conclusion final paragraph, the present tense should be used, or the present perfect, but not the preterit.

Reply:

Thank you for your detailed and professional comments. We have put the manuscript in the present tense where needed. Please see the labelled version.

Watch out the terminology used: "sex research (line 126)" deal with other fields of research (see for example the content of the "journal of sex research") and you might want to use a more specific wording, example given:

sexual reproduction, sex chromosomes, sex determinism and differentiation, sexual dimorphism, genetics/genomics of sex...

Reply:

Thanks for your professional advice, what we want to convey is that sex determination research, and we have made this place clear and descriptive. At the same time, we have made similar changes throughout the text. We revised “sex research” as “sex determination research”.

L246: Table 1":" (is missing) and put the number in an easy format (e.g. align right, number display)

Reply:

Thanks for your detailed comments. We have added the notation at the right of the Table 1.

L288: number display homogeneity. (an line 228 page 157

Reply:

We have taken these numbers to show homogeneity as follows: “(diff: oneInThousand, VarRange: 0 ~ 1,000,000 bp, BatchSize: 500 bp, Min Align Length for Cov Calc 10,000 bp, Min Align Length for Var Calling: 50,000 bp)”.

Why not using parrotfish instead of O. fasciatus along the text, tables and figures?

Reply:

Thanks to your professional comments, we have changed the species names to English throughout the text, except for the first occurrence where we have used Latin names. Please check the labelled version.

L765: PrEvious

Reply:

Thanks for your detailed comments. We have revised the “PrEvious” as “Previous”.

Line 244 page 157: Two consecutive blank spaces

Reply:

Thanks for your detailed comments. We replaced two consecutive spaces with a single space, and at the same time we searched the whole text and modified the double-space phenomenon in a similar way. Please check the labelled version.

Try not to use scientific names and vernacular ones in the same sentence (e.g. Drosophila melanogaster, Caenorhabditis elegans, mouse, human, and Arabidopsis thaliana)

Reply:

Thanks to your professional comments, we have standardised the names of species with scientific names in a single sentence as follows: “genomic genetic polymorphisms in various organisms, including *Drosophila melanogaster*, *Caenorhabditis elegans*, *Mus musculus castaneus*, *Homo sapiens*, and *Arabidopsis thaliana*”.

again, check homogeneity of occurrence of empty character between a value and its unit (eg line 768: 40Mb with no space, as other values has one.

Also line 590 and 591: a 965-kb deletion and a 4.8 Mb deletion, please homogenize.

Reply:

Thanks for your detailed comments. We have standardised the criteria, with uniform spaces after numbers and units, and have made changes throughout the text. Please see the labelled version.

Watch out that transposons are only class II (DNA) transposable elements. I guess that the authors were talking about Transposable Elements (TEs)

Reply:

Thanks to your professional comments. It is indeed transposon elements that are mentioned in our MS. And we changed transposons to "transposable elements". Please see the labelled version.

Reviewer #3: I thank the authors for their revision. Especially the inclusion of the actual genome assemblies this work is based on is important.

Below I return to my comments on the previous version:

1. Thank you for the addition. One comment: in the Sample collection, please rephrase line 191: HQ DNA was not extracted by Illumina/PacBio, but analyzed?

Reply:

Thanks for your detailed comments. Sorry for the confusion due to the mis-expression. We meant to extract high-quality genomic DNA for high-throughput sequencing on the PacBio and Illumina

platforms. We revised "High-quality genomic DNA was extracted from fresh muscle tissue and blood samples from pairs of male and female Japanese parrotfish using the Illumina platform (Illumina Inc., San Diego, CA, USA) and PacBio Sequel (Pacific Biosciences of California, Menlo Park, CA, USA) [45, 46]" as "High-quality genomic DNA was extracted from fresh muscle tissue and blood samples from pairs of male and female Japanese parrotfish using for high-throughput sequencing on the Illumina platform (Illumina Inc., San Diego, CA, USA) and PacBio Sequel (Pacific Biosciences of California, Menlo Park, CA, USA) [45, 46]"

2-3. Thank you for updating. I would suggest summarizing a bit more in the discussion, as it is very lengthy and some information is presented twice. Line 543: I would replace 'genomic structural variation' with just 'genomic variation', as you include SNPs under this definition.

Reply:

Thanks to your professional comments. We have deleted and modified redundancies and repetitions in the discussion by careful contextual comparison as follows:

1) Line 485 -486: We deleted "The majority of fish exhibited limited sex chromosome differentiation, which posed challenges in identifying them based on chromosome morphology [10]".
2) Line 499-502: We deleted "However, the advancement of sex-controlled breeding has faced limitations due to various factors, such as low throughput of the above methods, cumbersome experimental procedures, expensive development cost, high rate of false positives, unstable markers, and inconvenient on-site testing.".
3) Line 506-512: We revised the "In this study, O. fasciatus with the X1X1X2X2/X1X2Y system served as the focal point [44-46, 48]. By utilizing the assembled genome information of male and female individuals acquired through PacBio sequencing, along with data from the short-read sequencing data and long-read PacBio sequencing data, we extensively employed genome-wide variant sites multiple scanning and identification, STS long reads depth evaluation and ranking, high-throughput design of primers for the target regions, and e-PCR batch amplification." as "Our study focused on O. fasciatus with the X1X1X2X2/X1X2Y system [44-46, 48]. We used the assembled genome information of male and female individuals, along with short-read and long-read data, to identify variant sites and evaluate read depth. We also designed primers for target regions and performed batch amplification using e-PCR (Figure 1, Figure 2, Figure 3, Table 2, Table 3)."
4) Line 512-517: We deleted "By employing a comprehensive approach involving genome-wide variant site scanning, high-throughput primer design within the desired region, and e-PCR batch amplification and validation methodologies, we successfully exploited a catalog encompassing insertion/deletion loci in large segments (>100 bp) for both male and female O. fasciatus".
5) Line 546-549: We deleted "However, larger genomic structural variants, which encompassed genetic variations not fully captured by SNPs and InDels, are closely linked to biological phenotypes and might significantly influence phenotypic traits, disease susceptibility, and adaptive capacity [79].".

4/6. I still think it is an odd choice to present deletions and insertions in different units.

Reply:

Thank you for your professional opinion. As you pointed out in your previous comments, insertions are summarised by the number of events, while deletions are summarised by nucleotide length, as

we have explained in our previous presentation in the figure and table notes. We know that an insertion event is as a locus, which may exist in an intronic, exonic or intergenic region, and presents the character of a single functional region. However, deletions are different, as the size of deletion sequences varies, there is uncertainty in the functional regions of the genome, and a very large deletion site may span both intronic and exonic regions or even intergenic regions. In order to avoid the confusion of the statistics, we have determined through consultation that we use site counts for insertion events and sliding window counts for deletion sequences to count the number of deletions. Of course, we also carried out subsequent studies and validation of functional genomics of insertion deletion variants, and we will consider the statistics separately in the subsequent studies. Thank you again for your professional opinion.

5. What I meant is that the joint between chr F8 and F10 can be used to generate a PCR fragment in males, but not in females. This is not a major issue, it was just a suggestion.

Reply:

Thank you for your professional opinion. As you mentioned, we also focused on the fusion region of the two chromosomes and performed a variant site scan, and we found that the fusion region had significant structural variation, but it was difficult for us to use it as a region for genetic sex marker development due to the inefficiency of amplification caused by the drastic variation. We found through variant site scanning that abundant variant sites also exist in the inverted region, which can be used as a stable region for sex marker exploitation.

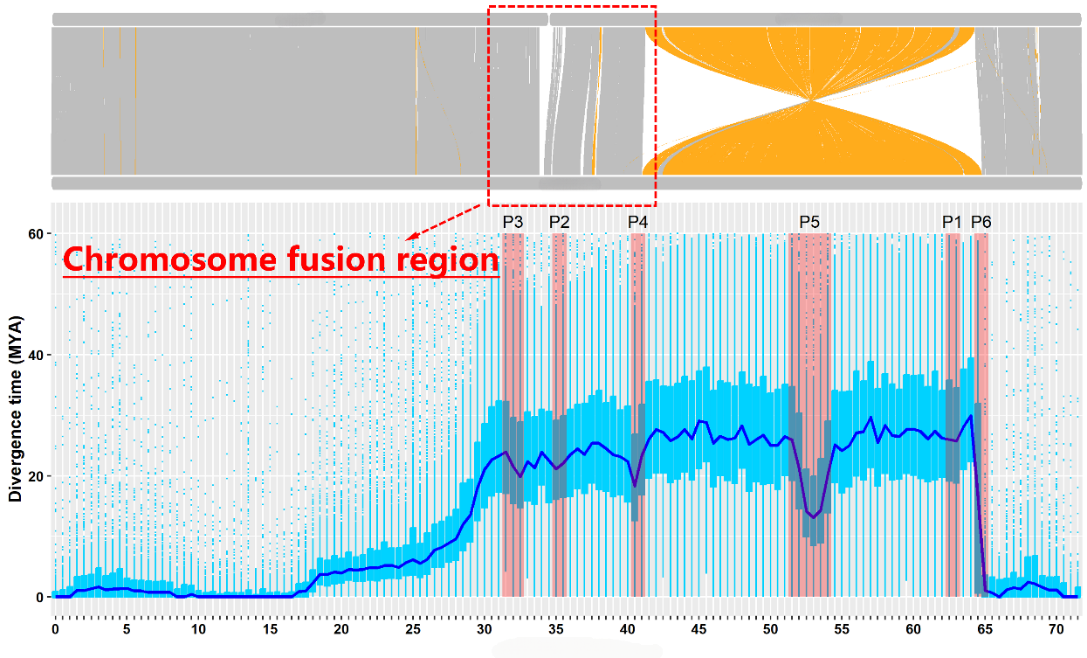

7. Thank you for the explanation, PCR constraints are plausible. But that would be good to highlight in the text. And it does not explain the imbalance in Table 5? Any deletion in females should be an insertion in males, and vice versa? If it is detectable from one perspective, then why not from the other?

Reply:

Thanks for your professional comments. As you said any deletion in females should be an insertion in males. In conjunction with your concerns we have found through technical discussion that the number of loci for insertions is 4422 using the female as the reference male comparison, so in turn

the number of deletions occurring when using the male as the reference female comparison should be close to 4422, which we have detected as 4385. The number of deletion sites is 4675 when using female as the reference male comparison, and conversely, the number of insertion sites is 4615 when using male as the reference female comparison, so that the number of insertion and deletion sites in our results are almost balanced under different reference groups.

Meanwhile, we also explained the differences in the efficiency of PCR high-throughput amplification primer design for insertion and deletion sites in the paper as follows: "Variations in the efficacy of PCR high-pass primer design at insertion and deletion sites may be attributed to disparities in structural characteristics and GC content within the regions flanking the insertion/deletion variants.".

Table 5. Statistics of large-segment differential loci after primer design, e-PCR amplification, and simulated amplification products and size screening.

Reference genome	Mutation type	Variant length (>100 bp)	High-throughput design of primers for variant sites	e-PCR of primers with single band	Single band and suitable amplification size (100~2000 bp)
Female vs. Male	Insertion	4,422	4,016	5,735	2,786
	Deletion	4,675	1,314	957	853
Male vs. Female	Insertion	4,615	4,131	3,236	2,876
	Deletion	4,385	1,199	882	796

8. Fig 3e/f is clearer now.

Reply:

Thanks for your previous comments on our manuscript, which have been very helpful in improving the quality of our manuscript.

9. OK

Reply:

Thanks for your previous comments on our manuscript, which have been very helpful in improving the quality of our manuscript.

10/11. I think this figure still needs some work. The legend says that PacBio reads are in red, but I see both pink and blue (I think fwd/reverse? Also sort of suggests male/female). These IGV(ish) screenshots are not very clear, please explain a bit more. Why just the ~25 nt stretches? Are the dashed lines the deletions (I guess not)? The PacBio reads suggest a deletion in half the reads, which fits the story, but those dark blue scribbles are hard to see. Do we see entire reads, or just aligned portions?

Reply:

Thanks for your professional comments. We are very sorry for the confusion caused by our description. Our comparison strategy here is to use the female genome as a reference male for comparison. As we mentioned before the sex chromosome system of female fish is X1X1X2X2 and the sex chromosome system of male fish is X1X2Y, so there will be dimorphism in the high throughput sequenced reads of male fish, especially in the inverted region, and this phenomenon will be more obvious. Because PacBio's CLR sequencing method is used, there is no problem with bidirectional sequencing. However, because we used male and female fish with inverted structural

variants, the phenomenon of matching direction was generated. We are sure that the pink and blue reads you see should be the reflection of the inversion of the male reads with the female fish as the reference. We have changed the content of CLR reads described in the figure to pink and blue to represent the same and opposite orientation of the female genome.

As you can see and as we provide the IGV insertion results plot below, since we have consolidated representative results in the manuscript, we have intercepted a portion of the sequence that contains the insertion deletion site and both ends in order to reflect the presence of the insertion and deletion events clearly. The dotted line you see is the base pair baseline, which appears as a single dotted line from a distance, and when zoomed in is two dotted lines corresponding to a single base. The blue scribble you see is actually the result of the size of the insertion fragment as shown below. In order to fully demonstrate this result of ours, we have changed the vertical orientation to horizontal.

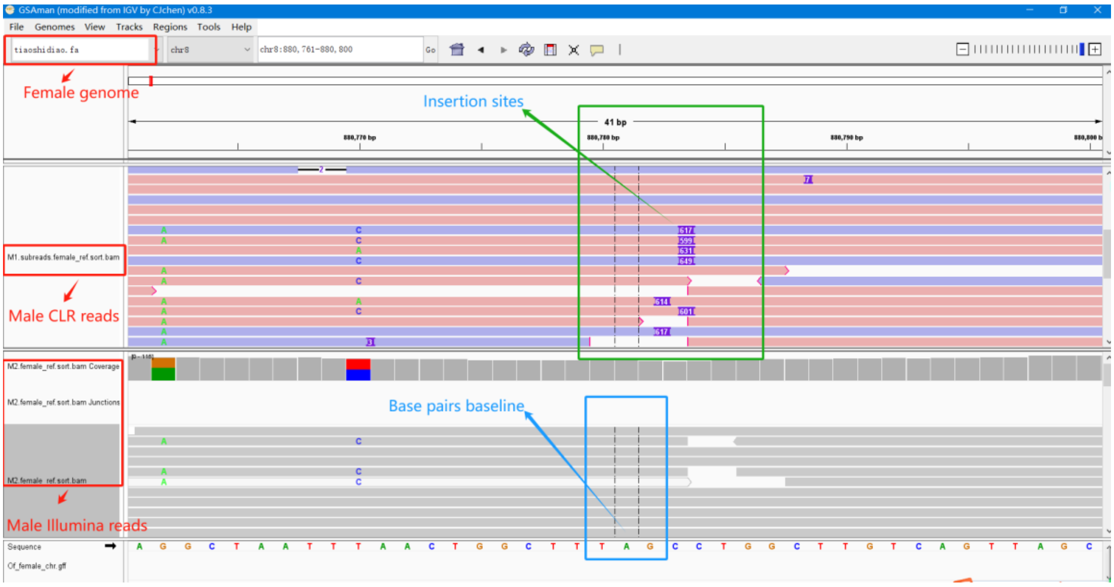

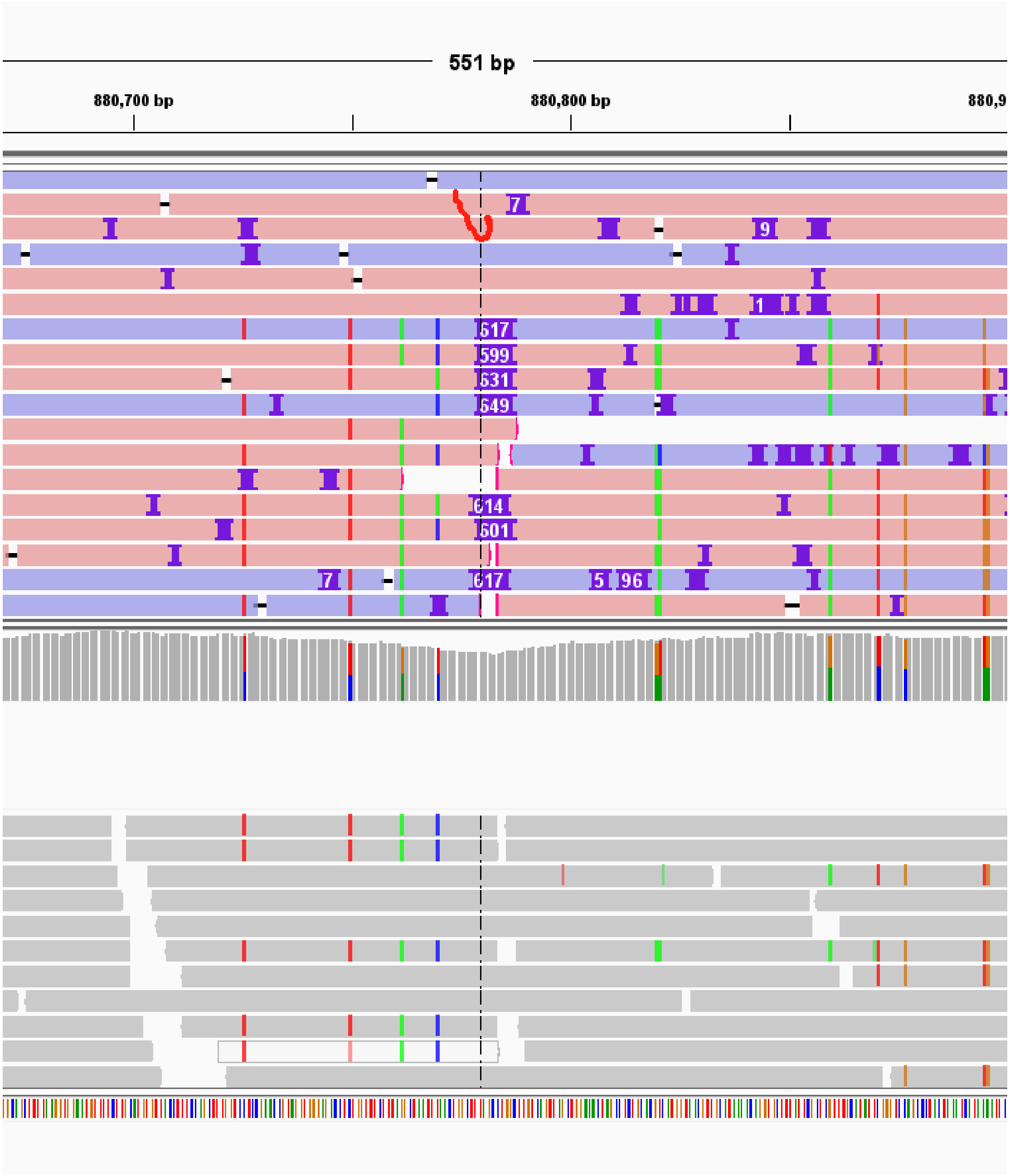

Line 553: Prvious -> Previous

Reply:

Thanks for your detailed comments. We have revised the “PrEvious” as “Previous”.
