# Supplementary material for: Innovative approach for high-throughput exploiting sex-specific markers in Japanese parrotfish Oplegnathus fasciatus
Source: Gigascience. 2024 Jul 19;13:giae045. doi: 10.1093/gigascience/giae045 (PMC11258905; doi:10.1093/gigascience/giae045)
Supplement: giae045_Supplemental_Files [file Figure S9.pdf › Figure S9.pdf]

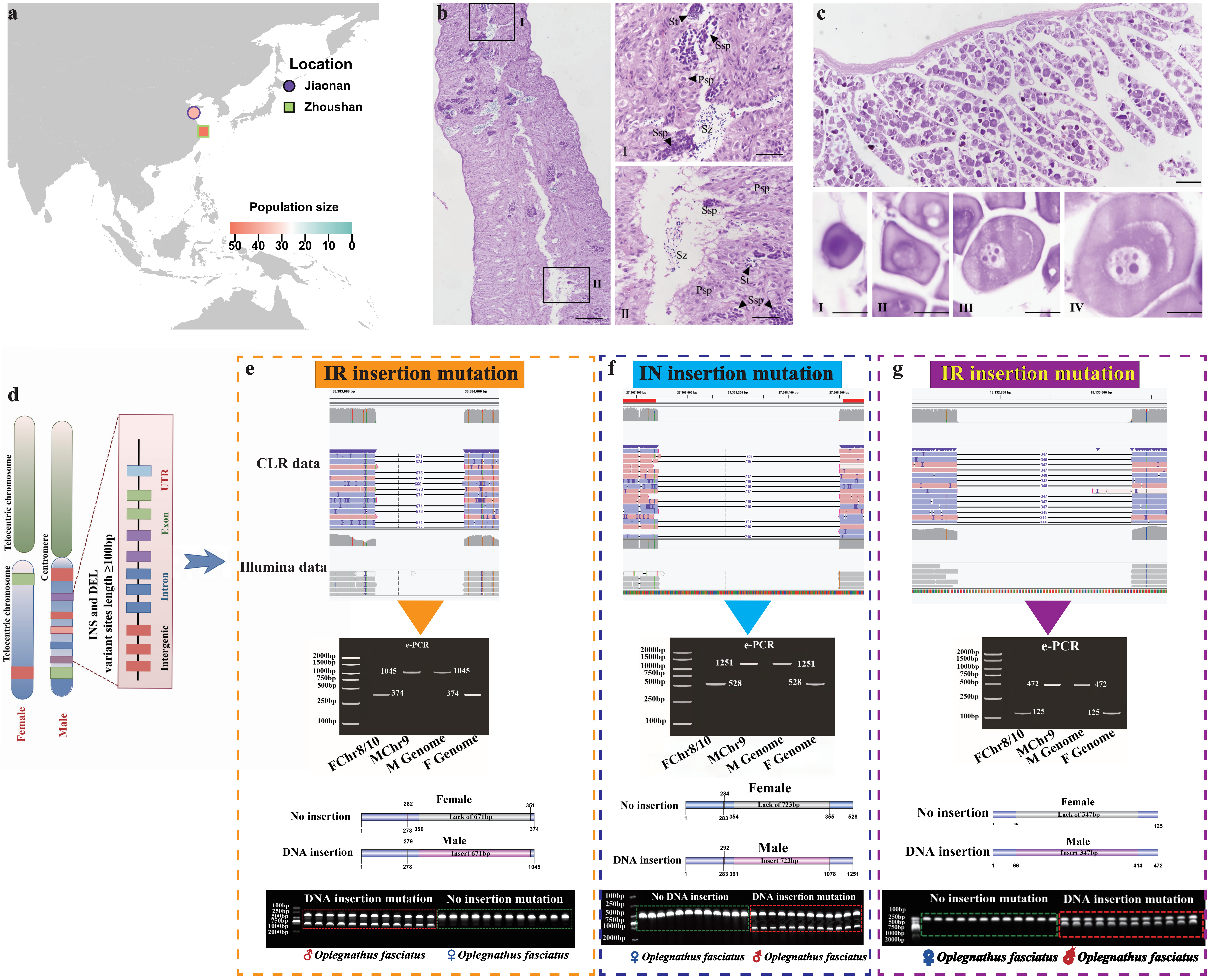

a
Location
Jiaonan
Zhoushan
Population size
50 40 30 20 10 0
b
I
II
St
Ssp
Psp
Sz
c
I
II
III
IV
d
Telocentric chromosome
Centromere
Telocentric chromosome
Female
Male
INS and DEL
variant sites length ≥100bp
UTR
Exon
Intron
Intergenic
e
IR insertion mutation
CLR data
Illumina data
e-PCR
2000bp
1500bp
1000bp
750bp
500bp
250bp
100bp
1045
374
FChr8/10
MChr9
M Genome
F Genome
Female
No insertion
Lack of 671bp
Male
DNA insertion
Insert 671bp
DNA insertion mutation
No insertion mutation
♂ Oplegnathus fasciatus
♀ Oplegnathus fasciatus
f
IN insertion mutation
e-PCR
1251
528
FChr8/10
MChr9
M Genome
F Genome
Female
No insertion
Lack of 723bp
Male
DNA insertion
Insert 723bp
No DNA insertion
DNA insertion mutation
♀ Oplegnathus fasciatus
♂ Oplegnathus fasciatus
g
IR insertion mutation
e-PCR
472
125
FChr8/10
MChr9
M Genome
F Genome
Female
No insertion
Lack of 347bp
Male
DNA insertion
Insert 347bp
No insertion mutation
DNA insertion mutation
♀ Oplegnathus fasciatus
♂ Oplegnathus fasciatus
