# Supplementary material for: Innovative approach for high-throughput exploiting sex-specific markers in Japanese parrotfish Oplegnathus fasciatus
Source: Gigascience. 2024 Jul 19;13:giae045. doi: 10.1093/gigascience/giae045 (PMC11258905; doi:10.1093/gigascience/giae045)
Supplement: giae045_Response_to_Reviewer_Comments_Original_Submission [file giae045_response_to_reviewer_comments_original_submission.pdf]

Author's Response To Reviewer Comments

Reviewer #1:
In this manuscript, Xiao et al performed bioinformatic analysis of Illumina and PacBio sequencing data to detect insertion/dele
clearly supported by PCR results. But there are still a few shortcomings that needs to be addressed. Below I explain my majo

In this manuscript, the authors assert that they have created a database of insertion/deletion-based sex-markers. After revie
"variants catalog" for clarity and accuracy.
Reply:
By carefully reading the reviewer' comments, we realized that we had indeed obtained a catalog rather than a database of ma
database-related contents of the paper into catalogs, and the list of changes is as follows:
1. We have changed "Datasets" in "All Chromosome variants Dataset", "Bulk Primer Design for Variant Datasets" and "Batch

2. Line 46, we revised "a database of large-segment (>100 bp) insertion–deletion" as "a catalog of large-segment (>100 bp)
3. Line 48, we revised "A database of bulk primers and simulated PCR for the…." as "A catalog of bulk primers and simulated
4. Line 53-54, we revised "A high-throughput identification database of sex-specific markers for Oplegnathus fasciatus was co
5. Line 56, we revised "Four differential loci were randomly selected from the database for validation, and the……" as "Four di
6. Line 68, we revised "we successfully developed a database encompassing insertion/deletion loci in large segments (>100 b
7. Line 145, we revised "In this work, a database of genomic differences between females and males was" as "In this work, a
8. Line 147-148, we revised "Using the difference database, we obtained datasets of insertion and deletion variant sites large
9. Line 166-167, we revised "a high-throughput precision marker dataset for male and female genetic sex identification was c
10. Line 176-177, we revised "we established databases containing genomic disparities between males and females." as "we
11. Line 178-179, we revised "as well as to screen large insertion-deletion sites (>100 bp) against a differential database," a
12. Line 221-222, we revised "we screened this insertion/deletion variant site database for variants larger than" as "we scree
13. Line 462-463, we revised "we successfully developed a database encompassing insertion/deletion loci in large segments (

In addition to mapping Illumina and PacBio reads against references, did the author also consider to directly map both male a
male and female fish used for the reads alignments. Authors should clarify whether or not the variants detection was affected
Reply:
We would like to extend our sincere thanks to you for your insights and thoughtful suggestions. And it was your suggestion th
We used SYRI software for the evaluation of variant sites (≥100bp) between the male and female reference genomes of Ople
(≥100bp) and 2055 large segment insertion sites (≥100bp) by comparing the female genome with the male genome as a ref
segments repost visualization presentation. We found that by using the intersection of deletion sites, the results of SYRI scan
reference genome showed that 1791 out of 2116 deletion sites were in our deletion site catalog set, accounting for 84.64% o
fasciatus. We have put the information of large segment insertion deletion sites obtained by SYRI and the information of larg

The SYRI software runs the following script:
1) nucmer --mum -l 100 -c 1000 FM.fasta OfM_230823_filter_rename.format.fa
2) delta-filter -i 90 -l 100 out.delta > out.filtered.delta
3) Show-coords -THrd out.filtered.delta > out.filtered.coords
4) python3 syri_env/bin/syri -c out.filtered.coords -d out.filtered.delta -r FM.fasta -q OfM_230823_filter_rename.format.fa --
As your mentioned, we should have stated in the text the assembly quality evaluation parameters of the reference genome u
"The assembly of the male and female genomes of O. fasciatus was successfully completed utilizing PacBio CLR sequencing te
Mb, while the male genome size was 762.27 Mb, contig N50 at 2.18 Mb, and scaffold N50 at 32.43 Mb. The Benchmarking Un
As you noted, it is important to consider the potential impact of artifacts on the variant sites obtained in the genome assembl
reference genomes, and ranking the reads depth of the large fragments of the variants. By implementing this approach, we a
For the efficiency of long-read based sex-specific markers, authors should clarify how many variants were experimentally vali
Reply:
We express our gratitude to you for your valuable feedback and suggestions. The development of low-cost high-throughput g
this end, we are undertaking a two-pronged approach: first, assessing the stability and reliability of insertion-deletion sites. I
and female chromosomes of O. fasciatus differed and that the males underwent a chromosome fusion event. Moreover, the m

that underwent chromosomal structural variation. In order to avoid the influence of the structural dimorphism of the male chr
females to the reference genome of males.
We obtained deletion sites for 4675 (female genome as reference) and 4385 (male genome as reference) large segments bas
(female genome as reference) and 2045 (male genome as reference) variant sites, respectively, and 84.64% and 85.53% of
deletion sites, and then ranked the insertion deletion site markers based on the depth of long reads, for large segment deleti
then classified the markers. We found that long read depths less than 5.48 (895 markers) and 12.50 (1,021 markers) belong
As we have known, stable variant loci are the basis for the exploitation of genetic sex markers, but effective amplification is f
for high-throughput amplification of variant markers. Based on this method, on the one hand, we can quickly identify the loci
development of markers for both male and female genetic sexes. We targeted the fusion chromosome (MChr9) of male fish a
successfully completed the validation of the male and female of 11 loci. Meanwhile, we found that the insertion deletion sites
of the PCR reaction (using touch down method) are the key to the development of markers after the need.
In the manuscript's abstract, there are numerous undefined abbreviations and technical terms. Authors should revise the abs
Line 36-37: "first-generation development methods such as RFLP, 37 RAPD, SSR, and AFLP as well as second-generation tech
It is confusing molecular biology techniqies with next generation sequencing (NGS) techniques.
Line 45: "Illumina survey" can be revised "short-read sequencing data survey"
Line 46: "PacBio CLR / CCS" can be revised "long-read PacBio sequencing"
Reply:
We are very grateful to you for your professional advice, which made us realize that biotechnology and sequencing technolog
“Background
The use of sex-specific molecular markers has become a prominent method in enhancing fish production and economic value
biology methodologies such as RFLP (Restriction Fragment Length Polymorphism), RAPD (Random Amplification of Polymorph
significant molecular markers for investigating sex identification in fish. The advancement of sex-controlled breeding encount
the emergence and swift progress of PacBio high-throughput sequencing technology, characterized by its long read output ca
We also appreciate your wonderful suggestions. First, we revised “Illumina survey” and “PacBio CLR / CCS” as “short-read se
“Utilizing male-female linear genomic information in conjunction with short-read sequencing data survey and long-read PacBi
based on the long read depth of the insertion deletion site, with markers exhibiting lower long read depth being considered m
for rapid identification of male and female differences in Oplegnathus fasciatus were exploited through agarose gel electropho
3,872 markers in conjunction with 1,021 and 894 high-quality genetic sex identification markers, respectively. Sixteen differe
the rapid advancement of genetic breeding through expediting the high-throughput development of sex genetic markers for v
“Conclusions
Our study utilized linear genomic information from male and female individuals obtained from PacBio, in addition to data from
with statistical analysis and ranking of the long reads depth of the variant sites. Through this integrated approach, we succes
On this basis, we revised the manuscript from "Illumina survey and PacBio CLR/CCS data" to "short-read sequencing data su
1) Line 318-320, we revised “we utilized the male Illumina survey data and PacBio CLR data for read assignment and further
valid insertion site”.
2) Line 338-340, we revised “A comparison of sequence differences, Illumina survey data and PacBio CLR data read assignme
confirmed that the genetic differences between male and female markers were mainly concentrated in the….”.
3) Line 449-452, we revised “along with data from Illumina surveys and PacBio CLR/CCS, we extensively employed whole-ge
extensively employed whole-genome variant site scanning and identification, high-throughput design of primers for the targe
The methods described in this manuscript is not clear and missing details. how many samples were sequenced using Illumina
BWA (v 0.7.8) and Minimap2 were used, but as could not follow which aligner map Illumina or PacBio reads?
Reply:
We are very grateful to your constructive comments, and we have added the sample sources of the genomes and the quality
Table 1. Parameters for Illumina and PacBio high-throughput sequencing and sequencing quality assessment of male and fem

“Sample collection, sequencing and genome quality
Male and female O. fasciatus captured from Qingdao, Shandong Province (Yellow Sea) were utilized for genome sequencing a
Biosciences of California, Menlo Park, CA, USA). Genomic DNA from fresh muscle tissue and blood samples of male and femal
totaling approximately 90.7 Gb and 63.9 Gb were generated. After removing splice-containing sequences, low-quality, and re
male and female fish were then constructed using the PacBio Sequel platform (Pacific Biosciences of California, Menlo Park, C
genomes, respectively, with SMRT LINK 5.0 used to filter the raw data from the zero-mode waveguide. We obtained 62.8Gb a
with Corrected-Error-Rate set to 0.040, followed by redundancy removal using Redundans v0.13c (minCoverage = 15), geno
genomes at the chromosome level of female (768.8Mb, accession SUB14064808) and male (762.2Mb, accession SUB1406579
subjected the assembled sequences to BUSCO version 5 evaluation (BUSCO, actinopterygii_odb10), with Complete BUSCOs a
One of my main concerns is the simulated e-PCR. I am not sure, how much this technique is reliable for validation studies an
yes, it is recommended to cite with brief description of reliability of this e-PCR versus normal PCR in lab.
Reply:

Thanks to your meticulous suggestions, as you mentioned e-PCR is indeed a very interesting tool, we checked the developme
the use of e-PCR with a single primer on the web, specifically for the Human Genome UCSC website database. As envisioned
limited genomic data and the amplification of a single STS have not allowed the proposed rapid visualisation, making the tech
Gregory D. Schuler. (1997) Sequence Mapping by Electronic PCR, Genome Research, 7:541–550.
Kirill Rotmistrovsky, Wonhee Jang and Gregory D. Schuler. (2004) A web server for performing electronic PCR. Nucleic Acids
With the development of high-throughput sequencing technology and the improvement of genome analysis methods, the flux
variant markers and high-throughput development. Therefore, we integrated high-throughput identification of large insertion-
(STS) markers based on male and female genome data, and accelerate the process of fish genetic breeding. Let's take a case

Female genome STS

GGACCGGGGCAAGGGACCCACACAATCGATGATCAAGTGTTCAAAAGGCTGTCCAATTGCAGGGATTGGATGCAAGGGAGCAGGTTTAAC
CAACAACTTTAAAAATTGGCTCTCCTACAAACTGTTCACCATGGGGCATTCACTTTCTAACCAGGTTACTGGTTAGAAAGTGAAAGCCTCCCT

Male genome STS

GGACCGGGGCAAGGGACCCACACAATCGATGATCAAGTGTTCAAAAGGCTGTCCAATTGCAGGGATTGGATGCAAGGGAGCAGGTTTAAC
CAACAACTTTTGTTGGCTCTCCTACAAACTGTTCACCATGGGGCATTCACTTTCTAACCAGGTTACTGGTTAGAAAGTGAATGCCCCATGGT
ACCACATCCTGTGCGGCATGGCTGGCAGCACGGGTGACAGCACAGGCTGGAAAAGTGTCATCCAGTGACCTCTCTGCGGGTGCTACCACA
ATGGAACCAAACCCATTCCTAGAATGATTACACACCCACCAGTATCAGTTTTGGATGAAAATGGCAACATAGACTGAAAGAATCCAACGCAC

On this basis, STS high-throughput e-PCR amplification detection was realized by using high-throughput scanning of sequenc
PCR.

High-throughput e-PCR amplification assay for STS

The gene otology enrichment findings seems to be out of context from the scope of the manuscript. The authors described th
consequences explanation.

Reply:

We appreciate your constructive comments, and based on your and the other reviewers' common suggestions, which we agre

The figures captions are not clearly described to explain the data. For example, in figure 3,
Line 251-252, what is meaning of different alignments patterns in dotplots? All the captions should be explanatory for the dat

Reply:

Thanks to your professional advice, We have organized all the images as requested the results are explained below

Figure 2: Distribution statistics of insertion and deletion fragment (>100bp) positions in genomic functional regions of O. fasc
(a) Distribution statistics of insertion variant loci using the female fish genome as a reference. (b) Distribution statistics of ins
genome as a reference.
Note: The colors red, green, yellow, and orange represented the UTR, CDS, intron, and intergenic regions. The frequency of i

Figure 3: The covariance of genomic sequence differences between the male and female genomes of O. fasciatus and distribu
(a) The female genome (reference) of O. fasciatus vs. the male genome for homology comparison using the minimap2 softwa
female genome was conducted using the minimap2 software with a 5000 bp sliding window. The male genome was represent
number of events. Deletions (>100 bp) were summarized by length in nucleotides. From outer to inner circles were the chror
valued histograms corresponding to the Table 3 showed the number of insertion/deletion sites per chromosome with referenc
represented by negative values, and deletion sites were represented by positive values..

Figure 4: Intersection results of two variant site scanning methods and ranking of variant sites with long read depth frequenc
(a) Venn results of deletion locus data were obtained based on TBtools and SyRI software using the female fish genome as a
found that 1,749 out of the 2,045 deletion sites identified through the use of the SyRI software were common deletion varian
labeled trough position (~5.4). (d) The depth distribution of CLR reads in females was analyzed by utilizing male genomic del
and females based on intersecting male and female genomic deletion site information (>100bp), respectively.

Figure 5: Representative genetic sex marker (insertion deletion variant sites) located in the intergenic region (IR), Intronic re
(a) Location of male and female sex markers in the male (MChr9) and female (FChr8) genomes. (b) Male and female sex gen
length long data from PacBio and gray represented was Illumina clean data. The e-PCR amplified bands were 390bp and 989b
markers for male and female sexes were identified at specific insertion sites within nuf2 gene introns (Figure S3, Figure S4).
differentiated groups. Males exhibited two amplified bands, while females exhibited only one. (d) By utilizing the female fish g
558bp and 928bp in length, aligning with the DNA deletion patterns and agarose gel electrophoresis maps utilized for distingu

Some figure (Figure 7), have unnecessary portion with alignment browser screen, it should be cropped or manually edited fo

Reply:
Thanks to your detailed advice, we cropped the resultant display image (Figure 7) after it appeared to be unnecessary when a

Figure 7

Figure 5

Minor comments:
The use of term "linear genomic information" is not very clear. Authors should clearly explain what they mean by linear geno
Reply:
Thanks to your professional advice, as you said the linear genome information was unclear and what they actually meant was
1) Line 197-198, we revised "sequencing technologies has made it possible to exploit high-throughput sex-specific markers in
2) Line 219-220, we revised "constructed based on the linear genomic information of O. fasciatus by using bidirectional whole
3) Line 231-232, we revised "First, a fast scanning method for female and male linear genomic variant loci was introduced.....
4) Line 598-599, we revised "By utilizing the linear genomic information of male and female individuals acquired through Pac

On Line 147, "Using the difference database" should be corrected "Using different databases"
Reply:
Thanks to your detailed advice, we revised "Using the difference database," as "Using the difference catalogs,"

Reviewer #2: Dear colleagues,

Than you very much for giving me the opportunity to read and comment the article of Xiao et al., entitled "A novel and scalab
Most Eukaryotes do sex, and pluricellular organisms have highly differentiated organs, using sex determination systems and s
sometimes being located in regions full of repeated sequences. Also, if sexual organs are easy to visualize and are used for cl
Asian fish farm industry, is less easy, and the sexual differentiation can only be observed once the animals have passed the 5
sex the individuals in the past, none were really conclusive. The authors propose to take advantage of the long reads provide
proxies of genetic sex markers.
In this work, the authors do present convincing technics and results. However, in that framework, it can be expected that the
insertions, and their stability.
Starting with the second, what is the origin of such insertions and deletions? Could it be due to the mobilisation of a transpos
Reply:
We are very grateful to you for your constructive comments, which led us to think deeply and explore the origin of insertion c
variant site transposons and tandem repeat systematic analyses as follows:
"To identify the origin of large segment insertion deletion sites, we evaluated whether the variant sites in the dataset were tra
results. At the same time, we utilized TRF (v 4.09.1) software to evaluate whether the variant sites in the dataset were tande
was more effective, we counted the sequence tagging sites (STS) attributed to transposons or tandem repeats of large segme
Table 4 Statistics of transposons and tandem repeat sequences attributed to large deletion sequence tag sites obtained with r
The results showed that 78.44% (3667) of the deletion sequence tag sites with female as reference belonged to transposons
of the STSs belonged to tandem repeats. As you predicted, at present, more than 77% of the large-band deletion sequence t
genome and chromosome evolution in a variety of ways, including insertion-induced gene mutations, gene transposons can c
of great significance to adaptive gene evolution.
"The findings further indicated that a substantial proportion of deletion sequence tag sites referencing the female genome we
were linked to transposon regions (77.08%, n=3,380) and tandem repeat regions (31.90%, n=1,399) (Table 4)."

The RepeatModeler (v 2.0.1), RepeatMasker (v 4.1.2) and TRF (v 4.09.1) softwares run the following scripts:
RepeatModeler (v 2.0.1)
1) RepeatModeler-2.0.1/BuildDatabase -name mydb genome.fa > assembly_final.fasta.repeatmodeler.log
2) RepeatModeler-2.0.1/RepeatModeler -database mydb -pa 6 > run.out
3) mv ./RM_* ./result
4) cat result/consensi.fa.classified > final.library
RepeatMasker (v 4.1.2)
1) RepeatMasker-4.1.2/RepeatMasker/RepeatMasker -nolow -no_is -norna -parallel 2 -lib RepeatMasker-4.1.2/RepeatMasker/
2) RepeatMasker-4.1.2/RepeatMasker/RepeatMasker -nolow -no_is -norna -parallel 2 -lib final.library genome.fa

TRF (v 4.09.1)
1) trf-v4.09.1/trf genome.fa 2 7 7 80 10 50 2000 -d -h

The first question deals with the level of polymorphism of these indels. How frequent are these insertions/deletions comparin
I understand that these two points are not the main interest of the present article. However, these evolutionary consideration
Reply:
Thanks to your professional advice, this issue made us think seriously and deeply, on the basis of which we extended the res
population and the Jiaonan population, and the results indicated that although the samples were from different geographic p

Validation of long read deletion sites for geographic populations using the male genome as a reference
As you mentioned large segments of insertion deletions are worth considering in terms of evolutionary considerations, especi
Discussion section as follows:
"Variation in genome structure was intricately linked to human diseases, evolution, gene regulation, and species phenotypes
and insertions in DNA sequences, regardless of their location in noncoding or coding regions, inevitably influences the functio
research has indicated that insertion deletion variants accounted for 16%, 24%, 13%, 25%, and 15% of genomic genetic pol
precise examination of human genetic variation, revealing that insertions and deletions collectively contribute to a high portic
50 base pairs due to the limitations of high-throughput sequencing technologies for short read lengths. However, larger geno
[79]. The utilization of long-read length sequencing technologies such as PacBio and ONT has enabled the generation of exte
(>100bp) within approximately 700bp large segments of the ZmBAM1d gene, demonstrating a positive association with maiz
family, has been identified, leading to stable genetic distinctions among species [97]. Similar results have been documented
research, particularly in investigations of species' adaptive evolution [95, 96, 102, 103]. The findings of this study indicated t
of large-segment insertion-deletion events for O. fasciatus, indicating that these events predominantly occurred in the interge
study conducted on the β-fibrinogen intron of birds reported similar findings [104]. Furthermore, this study presented novel f
genome and significantly contribute to its evolutionary processes. Previous research has demonstrated that a significant porti
in human brain diseases and genomic structural variation in evolutionary distinct phenotypes of great apes [103, 106]. Trans
ectopic recombination. These findings underscored the importance of transposons in facilitating adaptive evolution for O. fasc
with characteristic phenotypic traits and aberrant gene regulation [83, 84, 102, 103, 105, 106]. Specifically, the manifestatio
subsequent generations [100, 107]. Consistent inheritance of variants within populations has also been documented in acade
amplification analysis conducted in this study indicated that the insertion-deletion markers exhibited stability in both male an
individuals of both sexes across different geographic regions. This observation provided additional evidence to support the th
tag sites was evident. However, this should not be taken to imply that all sequence tag sites exhibit this characteristic. A com
A (non exhaustive) list of suggestions:

Title. I'd suggest to shorten it and use the vernacular name, at least, or explicitly say that this species is a fish.
Reply:
Thanks to your professional advice, we have modified the title of our paper as follows : "Innovative approach for high-throug
Abstract, it is not mentioned on what taxonomic species this article is dealing with.
Reply:
Thanks for your detailed advice, we added the taxonomic information for Oplegnathus fasciatus in front of "The criteria for ra
criteria for rapid identification of male and female differences in O. fasciatus were established through agarose gel electropho
Figures: font size is too small.
Reply:
Thanks for your detailed advice, we have re-enlarged the font in the image as follows:

Figure 1

Figure 2

Figure 3

Figure 4
Fig. 4, 5 and others would gain in being more didactical and summarized in one single image. These detailed information wou
Reply:
Thanks to your constructive advice, we strongly agree with your comment that this will make the whole study significantly sy
through the Supplementary Material.

Figure 5

Figure S9

Tables: position numbers using decimal tabulations and homogenize filled cells, text shouldn't be in the "justify" format
L 316 "bp base" is confusing.
Reply:
Thanks for your detailed advice, we revised the "a 518 bp base sequence was……" as "a 518 bp DNA fragment was……". We h
1) Line 507, we revised "……a 599 bp base sequence" as "……a 599 bp DNA fragment".
2) Line 547, we revised "…….males were missing a 343 bp base sequence" as "……males were missing a 343 bp DNA fragmen
3) Line 556, we revised "with deletion of a 299 bp base sequence" as "with deletion of a 299 bp DNA fragment".
4) Line 557, we revised "multifragmented base sequence in males" as "DNA multi-fragments in males"

L 486-488: This claim cites no reference. Why large sequences shouldn't evolve at random, unless of course, if they are direc
Reply:
Thanks for your detailed advice, previous research has indicated that insertion deletions are not randomly distributed, but rat
This may be attributed to the formation of unique structures in high GC regions, increasing the likelihood of sliding strand mis
necessitates the ability to integrate a previously copied DNA fragment. We have added the references and make discussion a
findings, the stability of large segment insertion and deletion in sequence tag sites was evident. However, this should not be
Fig 5d check spelling of nucleotide (here spelled "nucelotide"). Same for Fig. S2e.
Reply:
Thanks for your detailed advice, We did misspell the word in the image result by looking at it, we co-ordinated all the results

Figure 4

Figure 6

Figure 8

Figure S1

Fig8 b, identity is not correctly positioned below line "chrY" 380
Reply:
Thanks for your detailed advice, we re-did the sequence comparison and obtained the following results.

Figure 8

Mat&Meth
Maybe worth to subdivide into paragraphs (and titles).
Reply:
Thanks for your constructive suggestion, we the authors of the paper have agreed that this was very necessary work, so we
Sample collection, sequencing and genome quality
Large fragment variant loci scanning and identification
Write large number with separation, being coma (as in Result section) or small space
Reply:
Thanks for your detailed suggestion, we have systematically revised the paper where relevant by comma separating all refere
1) Line 172-173, we revised "(diff: oneInThousand, VarRange: 0~1000000 bp, BatchSize: 500 bp, Min Align Length for Cov
2) Line 301, we revised "amplicons of 390 bp and 1008 bp, respectively" as "amplicons of 390 bp and 1,008 bp, respectively
3) Line 308, we revised "with the results of e-PCR corresponding to two bands (390 bp and 1008 bp)" as "with the results of
L411: "REREgulation" is that correct?
Reply:
Thanks for your detailed suggestion, we revised "reregulation of structural morphogenesis" as "regulation of structural morph
References output has not been scrutinized. For example, almost no Journals are cited!!!
Species names should also be italicized in the list of references!
Reply:
Thanks for your detailed suggestion, We re-imported the reference format as requested and italicized the Latin of the species

Reviewer #3: This manuscript describes a computational approach to develop PCR markers for sex chromosomes in a fish wit

I have found some issues that I would ask the authors to address:
1. It is not entirely clear to me what data the study is based on. The two genome assemblies are not referenced by accession
Reply:
Thanks for your constructive suggestion, we found that the changes you suggested were necessary additions to our paper, w
Table 1 Parameters for genome sequencing and assembly quality assessment of chromosome-level genomes of male and fem

Materials and Methods
Sample collection, sequencing and genome quality
Male and female O. fasciatus captured from Qingdao, Shandong Province (Yellow Sea) were utilized for genome sequencing a
Biosciences of California, Menlo Park, CA, USA). Genomic DNA from fresh muscle tissue and blood samples of male and femal
totaling approximately 90.7 Gb and 63.9 Gb were generated. After removing splice-containing sequences, low-quality, and re
male and female fish were then constructed using the PacBio Sequel platform (Pacific Biosciences of California, Menlo Park, C
genomes, respectively, with SMRT LINK 5.0 used to filter the raw data from the zero-mode waveguide. We obtained 62.8Gb a
with Corrected-Error-Rate set to 0.040, followed by redundancy removal using Redundans v0.13c (minCoverage = 15), geno
genomes at the chromosome level of female (768.8Mb, accession SUB14064808) and male (762.2Mb, accession SUB1406579
subjected the assembled sequences to BUSCO version 5 evaluation (BUSCO, actinopterygii_odb10), with Complete BUSCOs a

2. The Introduction is outdated. The state of the art in long-read sequencing as described in the Introduction is at least 5 yea
are far more than 40 (line 101) fish species that have had their genome sequenced. And finally, the inclusion of Helicos (111)
Reply:
Thanks for your professional suggestions, I apologized for the confusion due to the lack of clarity. Here, we would like to high
on chromosomal structural variants. To avoid misunderstanding, we have modified the content here as follows. We revised "I
Gasterosteus aculeatus and Salmo salar, has been completed both nationally and internationally" as "In 2002, the genome se
including Oryzias latipes, Gasterosteus aculeatus, Pelteobagrus fulvidraco, Mola mola, Nothobranchius furzeri, and Salmo sala
21]. Despite the high throughput capabilities of second-generation sequencing, the relatively short sequencing read lengths p
We also greatly appreciated your professional reminder that we have removed Helicos from the long-read length high-throug
We revised "At present, third-generation sequencing technology with high precision of long fragments represented by single-r
sequencing technology (SMRT), and so on." as "At present, third-generation sequencing technology with high precision of lon
technology , and so on.".
Thanks to your professional advice, we have introduced the latest T2T assembly results of the human genome into our paper
the human genome. We revised "For the Chinese human genome sequenced by PacBio technology, the assembly level reache
contig N50 of 154.26 Mb, resulting in a seamless assembly that fills a crucial gap in the human DNA genetic code map".
"In recent years, with the rapid development of genome sequencing technology and the significant decrease in the cost of se
species germplasm resources[4]. In 2002, the genome sequencing of Takifugu rubripes was successfully accomplished, mark
aculeatus, Pelteobagrus fulvidraco, Mola mola, Nothobranchius furzeri, and Salmo salar, has been carried out on a national ar
capabilities of second-generation sequencing, the relatively short sequencing read lengths posed challenges in obtaining prec
At present, third-generation sequencing technology with high precision of long fragments represented by single-molecule rea
sequencing technology was the most widely used and commercialized core third-generation sequencing technology and has b
Oxford Nanopore ultralong-read sequencing, achieved a contig N50 of 154.26 Mb, resulting in a seamless assembly that fills a
of sequencing technology and the improvement of assembly technology, the genome integrity and accuracy have been greatl
high-throughput sex-specific markers in high-precision assembled genome data."
3. The Discussion is severely outdated. All references to structural variation are from the dawn of the genomics era, and ever
knowledge.
Reply:
We are very grateful for your expert comments and perceptive insights, which are extremely rewarding to learn from. Based
species Drosophila and nematode in consideration of the continuity of the data. At the same time, based on the latest results

"Variation in genome structure was intricately linked to human diseases, evolution, gene regulation, and species phenotypes
and insertions in DNA sequences, regardless of their location in noncoding or coding regions, inevitably influences the functio
research has indicated that insertion deletion variants accounted for 16%, 24%, 13%, 25%, and 15% of genomic genetic pol
precise examination of human genetic variation, revealing that insertions and deletions collectively contribute to a high portio
50 base pairs due to the limitations of high-throughput sequencing technologies for short read lengths. However, larger geno
[79]. The utilization of long-read length sequencing technologies such as PacBio and ONT has enabled the generation of exter
(>100bp) within approximately 700bp large segments of the ZmBAM1d gene, demonstrating a positive association with maiz

family, has been identified, leading to stable genetic distinctions among species [97]. Similar results have been documented
research, particularly in investigations of species' adaptive evolution [95, 96, 102, 103]. The findings of this study indicated t
of large-segment insertion-deletion events for O. fasciatus, indicating that these events predominantly occurred in the interge
study conducted on the β-fibrinogen intron of birds reported similar findings [104]. Furthermore, this study presented novel f
genome and significantly contribute to its evolutionary processes. Previous research has demonstrated that a significant porti
in human brain diseases and genomic structural variation in evolutionary distinct phenotypes of great apes [103, 106]. Trans
ectopic recombination. These findings underscored the importance of transposons in facilitating adaptive evolution for O. fasc
with characteristic phenotypic traits and aberrant gene regulation [83, 84, 102, 103, 105, 106]. Specifically, the manifestatio
subsequent generations [100, 107]. Consistent inheritance of variants within populations has also been documented in acade
amplification analysis conducted in this study indicated that the insertion-deletion markers exhibited stability in both male an
individuals of both sexes across different geographic regions. This observation provided additional evidence to support the th
tag sites was evident. However, this should not be taken to imply that all sequence tag sites exhibit this characteristic. A com

4. Results and Figure 2/Table 1: needs to be presented more clearly. The first reference to fig 2 (line 218) does instead refer
Reply:
We thanks for your detailed and professional comments. Incorporating the revisions you made, we find that it will make our r
1) Firstly, we obtained raw variation data based on the male and female genomes as a reference using bar charts for present
of events, and the deletion variant types were summarised according to the nucleotide length. At the same time, we used dy

Figure 2: Distribution statistics of insertion and deletion fragment (>100bp) positions in genomic functional regions of O. fasc
(a) Distribution statistics of insertion variant loci using the female fish genome as a reference. (b) Distribution statistics of ins
genome as a reference.
Note: The colors red, green, yellow, and orange represented the UTR, CDS, intron, and intergenic regions. The frequency of i

Figure S1: Frequency of insertion and deletions (>100bp) in functional regions of the O. fasciatus genome
(a) Number and frequency of insertion events using the female O. fasciatus genome as reference. (b) Distribution statistics o
using the male O. fasciatus genome as a reference.
Note: Non trans-variation represented the intron, and intergenic regions. Trans-variation represented the UTR, CDS regions.
2) Secondly, we have added labelling to the insertion deletion variant locus statistics table to make the distribution of the dat
Table 2. Statistics of the positional distribution of insertion/deletion variants in the male and female genomes of O. fasciatus.
Note: Insertions (>100 bp) were summarized by number of events. Deletions (>100 bp) were summarized by length in nucle
3) Thirdly, we explicitly indicated unfiltered and filtered data and supplemented the unfiltered male-female difference dataset
φ We revised "Using the female genome as a reference, we obtained 1,919,620 male and female differential variant sites, inc
including 191,915 insertion sites, 325,620 deletion sites and 1,402,085 nucleotide substitutions (Supplementary Female_refe
κ We revised "Using the male O. fasciatus genome as a reference, 1,926,295 female–male differential variant sites were obta
data were obtained, including 326,823 insertion sites, 192,672 deletion sites and 1,406,800 nucleotide substitution sites (Sup

5. Figure 3a/b: There is a huge inversion between female chr 8 and male chr 9. Did you consider using this type of structural
Reply:
We are very grateful for your expert comments and careful advice. As in our previous study, we found that the male and fem
and FChr10 in females). Therefore, we focused on the differences between male and female homologous chromosomes that u
region is rotated by 180 compared to the original sequence and reverse complementarity occurs at the same time, so when w
unchanged. We have also listed a case. From the results, the inversion does not affect the amplification of the target region,
genomes.

Male and female chromosomal differences in O. fasciatus and a chromosomal fusion event in males

Male fish fusion chromosome (MChr9) undergoes significant structural variation compared to its homologous chromosomes (f
Primer:
F: 5~ AAGTGAAGAAAGTTCATGATGGCACTT~3
R: 5~ TCTGCCGATGTGCTTGGTCTACGATCTTTCA~3
>Eaxmple1- normal sequences (739bp)
F: 5~ AAGTGAAGAAAGTTCATGATGGCACTT~3
AAGTGAAGAAAGTTCATGATGGCACTTAGAACAGTCAGAGTCTTCTGTTTTTCTGGAAAGGAAGAACAACACCGCTATCCAGTTATCAGATG
CCCCTCCTCTCCGTACACACTCTAAATCAAAACTATGGCGTTTAATTAGTCTCAAAACTGTGCACATATATAAAACAGGTGCGTGCACGTGAT

GGCTGATGTGGCCCCTCCTGTGATTCCTTTTATTGGTCACTTTCAAGCTCAGCTACCACCACAAATAGAATCTAATTCTGGGGGAAACACTG
3~ACTTTCTAGCATCTGGTTCGTGTAGCCGTCT~5 R
>Eaxmple2- inversion sequence (739bp)
R: 5~ TCTGCCGATGTGCTTGGTCTACGATCTTTCA~3
TCTGCCGATGTGCTTGGTCTACGATCTTTCACTGAATGACCTTCTAGCTCCAAGTAAGTGTATGTTTTTCAGTTTTATATGTCCAAAGTTTTTC
GCGAGCACGTAGGGAGGGTCGAGCTCATGGTCGTCAGAGCCCCGTGTTTGCGGGACCGCGCTTGTACATGCACGGGTTTCGCGGATTCTC
GCAAGGAGTTTAAAAAAATAGAAAATCAATATGTGGCGGTCAATGTTAATATTGTGCCGGGCCGCCACAAACCAATAACCAATGTGTGGGA
3~TTCACGGTAGTACTTGAAAGAAGTGAA~5 F
From the above results, the inversion does not affect the amplification of the target region, and we put this marker with male

6. Figure 3c/d: According to the legend, ring 2 (from outside) are insertions, and ring 3 deletions. There should be some bala
Reply:
We thanks for your detailed and professional comments. As you pointed out to us earlier in the proposal, insertions are summ
10k sliding window, our insertions represent the number of insertion positions, the more insertions the higher the point positi
insertion deletion sites on each chromosome to the table in order to prevent similar misinterpretations, and at the same time
image in figure3 obscuring the data.

Positional statistics of 10k sliding window insertion counts with female genome as reference

Statistics on the length of deletion bases in the 10k sliding window using female genome as a reference
Table 3. Statistical cross-validation of multiple scanning of genome-wide large insertion deletion sequence marker sites.

Figure 3: The covariance of genomic sequence differences between the male and female genomes of O. fasciatus and distribu

7. Similar issue: In table 2, insertions and deletions are balanced. However, after primer design there are far fewer deletions
Reply:
We thanks for your detailed and professional comments. High-throughput primer design and high-throughput amplification of
that could amplify a single target band in both male and female genomes based on e-PCR. Of course, the simulation principle
content. We supplemented the process of large-segment differential loci after primer design, e-PCR amplification, and simula
Previous research has indicated that insertion deletions are not randomly distributed, but rather are linked to specific sequen
formation of unique structures in high GC regions, increasing the likelihood of sliding strand mismatch events. Similar pattern
integrate a previously copied DNA fragment. The findings of this study indicated that the quantity of primer pairs generated t
attributed to the constraints imposed by e-PCR on the GC content of flanking primers, whereas deletion variants frequently e
The inquiry into the mechanisms driving the formation of insertion and deletion variants is of particular interest in the scientif
Table 5. Statistics of large-segment differential loci after primer design, e-PCR amplification, and simulated amplification proc

8. Figure 3e/f: The asymmetry between the chromosomes is quite interesting, i.e. that some are prone to deletions and othe
should be a deletion in the female, vv. This is not the case for chr 15F/14M and 19F/18M.

Reply:
We thanks for your detailed and professional comments. I am very sorry for the misunderstanding because of our visualizatio
the bi-valued histograms being lopsided and obscuring the data. To solve this problem, we record the number of insertions as
insertions and deletions on each chromosome for illustrative purposes.

Figure 3: The covariance of genomic sequence differences between the male and female genomes of O. fasciatus and distribu
Table 3. Statistical cross-validation of multiple scanning of genome-wide large insertion deletion sequence marker sites.

9. Primer design, line 262 and further: what were the criteria? What were the PCR settings? Also, 264: 'designed high-throug
Reply:
Table 5. Statistics of large-segment differential loci after primer design, e-PCR amplification, and simulated amplification proc
We thanks for your detailed and professional comments. As you have seen above the table inside the high-throughput primer
region, and at the same time to meet the conditions that the difference in Tm value should not be more than 15 degrees, the
primers was much smaller than the number of primers inserted into the target region, which may be due to the fact that the
process will generate lots of non-specific amplifications, we selected only the primer pairs whose amplification product was a
We also revised "We used the identified large insertion/deletion variants as target regions for male and female sex identificat

genomes. High-throughput primers were designed to generate primer catalogs for the specified target markers.".
10. Figure 4: is different in the inline version (p14) from the attached (p36). Also identical to figure S1.
Reply:
We thanks for your detailed comments. As you say the inline version of Fig. 4 (p. 14) is different from the accompanying draw
the results of the validation images, and integrated the results of Fig. 4 with Fig. S1 as supplementary Figure S2.

Validation of long read variant loci in males using the female genome as a reference

11. Figures 4-8 consist mostly of screenshots that are not clearly explained, and not intuitively very informative. What am I l
Reply:
Thanks to your professional advice, We have organized all the images as requested the results are explained below

Figure 5: Representative genetic sex marker (insertion deletion variant sites) located in the intergenic region (IR), Intronic re
(a) Location of male and female sex markers in the male (MChr9) and female (FChr8) genomes. (b) Male and female sex gen
length long data from PacBio and gray represented was Illumina clean data. The e-PCR amplified bands were 390bp and 989
markers for male and female sexes were identified at specific insertion sites within nuf2 gene introns (Figure S3, Figure S4).
differentiated groups. Males exhibited two amplified bands, while females exhibited only one. (d) By utilizing the female fish g
558bp and 928bp in length, aligning with the DNA deletion patterns and agarose gel electrophoresis maps utilized for distingu
We apologize for the confusion caused by the lack of clarity of the results in Figures 4-8. In order to make the results clearer
two-generation data back-comparisons in the IGV in the integrated integration, and we have added the structural diagrams o
and PCR agarose gel electrophoresis test results. At the same time, we put Figure 4-8 as a supplementary file in the Supplem

12. Enrichment analysis (l 365-426): This text consist almost entirely of a listing of categories that are also printed in the sup
Reply:
We thanks for your professional comments. All of we authors think your comments are very professional through discussion,
combining your comments and the comments of the reviewers above. Thank you again for your professional opinion.

Some minor/textual remarks:
Lines 63-67 in the Abstract: repeats the same information twice.
Reply:
We thanks for your detailed comments. We revised "Our research extensively employed whole-genome variant site scanning
within the desired region, and e-PCR batch amplification and validation methodologies,.." as "Our study utilized a thorough m
insertion/deletion loci in large segments (>100 bp) for both male and female O. fasciatus.".
Line 151-152: same statement twice.
Reply:
We thanks for your detailed comments. We revised "On the basis of the O. fasciatus large-banded variant loci, we used PCR e
female and male genomes." as "Utilizing the O. fasciatus sequence tagged sites as a foundation, we employed e-simulation a
Line 156: PCR electron simulation -> simulated PCR.
Reply:
We thanks for your detailed comments. We revised "PCR electron simulation" as "simulated PCR".
Line 157: primer -> primer pair.
Reply:
We thanks for your detailed comments. We revised "primer" as "primer pair".
Figure 1: Typos Illuminar, onele thousand (?)
Reply:
We thanks for your detailed comments.
Line 179: Was BWA used for Illumina and Minimap2 for PacBio? Minimap2 needs a version number.
Reply:
We thanks for your detailed comments. As you said BWA software is used for Illumina data back comparison and Minimap2 is
