# Supplementary material for: Innovative approach for high-throughput exploiting sex-specific markers in Japanese parrotfish Oplegnathus fasciatus
Source: Gigascience. 2024 Jul 19;13:giae045. doi: 10.1093/gigascience/giae045 (PMC11258905; doi:10.1093/gigascience/giae045)
Supplement: giae045_Response_to_Reviewer_Comments_Revision_1 [file giae045_response_to_reviewer_comments_revision_1.pdf]

Author's Response To Reviewer Comments

Reviewer reports:
Reviewer #1: The revised manuscript is significantly improved and the authors have done a great job addressing all my previous comments. I appreciate authors responses and revisions to all points.
Reply :
Thanks for your previous comments on our manuscript, which have been very helpful in improving the quality of our manuscript.

Reviewer #2: Dear colleagues,

Thank you very much for the thorough, extensive, cautious and precise replies to the previous round of comments that I provide, as well as that of the two other reviewers in my opinion.
I just have a list of minor comments to provide.

Best regards,

Being a non-native English speaker, please double-check my comments below.

Minor comments:
In the first paragraph of the new version (starting line 125), the use of the present tense was better than that of the actual paste tense (also line 195, the use of "was" would mean that the species no longer exists in this geographical area). L228, it is still the same sex chromosome system. Line 683, etc. Same for the Conclusion final paragraph, the present tense should be used, or the present perfect, but not the preterit.
Reply :
Thank you for your detailed and professional comments. We have put the manuscript in the present tense where needed. Please see the labelled version.
Watch out the terminology used: "sex research (line 126)" deal with other fields of research (see for example the content of the "journal of sex research") and you might want to use a more specific wording, example given:
sexual reproduction, sex chromosomes, sex determinism and differentiation, sexual dimorphism, genetics/genomics of sex...
Reply :
Thanks for your professional advice, what we want to convey is that sex determination research, and we have made this place clear and descriptive. At the same time, we have made similar changes throughout the text. We revised "sex research" as "sex determination research".
L246: Table 1":" (is missing) and put the number in an easy format (e.g. align right, number display)
Reply :
Thanks for your detailed comments. We have added the notation at the right of the Table 1.
L288: number display homogeneity. (an line 228 page 157
Reply :
We have taken these numbers to show homogeneity as follows: "(diff: oneInThousand, VarRange: 0 ~ 1,000,000 bp, BatchSize: 500 bp, Min Align Length for Cov Calc 10,000 bp, Min Align Length for Var Calling: 50,000 bp)".
Why not using parrotfish instead of O. fasciatus along the text, tables and figures?
Reply :
Thanks to your professional comments, we have changed the species names to English throughout the text, except for the first occurrence where we have used Latin names. Please check the labelled version.
L765: PrEvious
Reply :
Thanks for your detailed comments. We have revised the "PrEvious" as "Previous".
Line 244 page 157: Two consecutive blank spaces
Reply :
Thanks for your detailed comments. We replaced two consecutive spaces with a single space, and at the same time we searched the whole text and modified the double-space phenomenon in a similar way. Please

check the labelled version.
Try not to use scientific names and vernacular ones in the same sentence (e.g. Drosophila melanogaster, Caenorhabditis elegans, mouse, human, and Arabidopsis thaliana)
Reply：
Thanks to your professional comments, we have standardised the names of species with scientific names in a single sentence as follows: “genomic genetic polymorphisms in various organisms, including Drosophila melanogaster, Caenorhabditis elegans, Mus musculus castaneus, Homo sapiens, and Arabidopsis thaliana”.
again, check homogeneity of occurrence of empty character between a value and its unit (eg line 768: 40Mb with no space, as other values has one.
Also line 590 and 591: a 965-kb deletion and a 4.8 Mb deletion, please homogenize.
Reply：
Thanks for your detailed comments. We have standardised the criteria, with uniform spaces after numbers and units, and have made changes throughout the text. Please see the labelled version.
Watch out that transposons are only class II (DNA) transposable elements. I guess that the authors were talking about Transposable Elements (TEs)
Reply：
Thanks to your professional comments. It is indeed transposon elements that are mentioned in our MS. And we changed transposons to "transposable elements". Please see the labelled version.

Reviewer #3: I thank the authors for their revision. Especially the inclusion of the actual genome assemblies this work is based on is important.

Below I return to my comments on the previous version:
1. Thank you for the addition. One comment: in the Sample collection, please rephrase line 191: HQ DNA was not extracted by Illumina/PacBio, but analyzed?
Reply：
Thanks for your detailed comments. Sorry for the confusion due to the mis-expression. We meant to extract high-quality genomic DNA for high-throughput sequencing on the PacBio and Illumina platforms. We revised “High-quality genomic DNA was extracted from fresh muscle tissue and blood samples from pairs of male and female Japanese parrotfish using the Illumina platform (Illumina Inc., San Diego, CA, USA) and PacBio Sequel (Pacific Biosciences of California, Menlo Park, CA, USA) [45, 46]” as “High-quality genomic DNA was extracted from fresh muscle tissue and blood samples from pairs of male and female Japanese parrotfish using for high-throughput sequencing on the Illumina platform (Illumina Inc., San Diego, CA, USA) and PacBio Sequel (Pacific Biosciences of California, Menlo Park, CA, USA) [45, 46]”
2-3. Thank you for updating. I would suggest summarizing a bit more in the discussion, as it is very lengthy and some information is presented twice. Line 543: I would replace 'genomic structural variation' with just 'genomic variation', as you include SNPs under this definition.
Reply:
Thanks to your professional comments. We have deleted and modified redundancies and repetitions in the discussion by careful contextual comparison as follows:
1) Line 485 -486: We deleted “The majority of fish exhibited limited sex chromosome differentiation, which posed challenges in identifying them based on chromosome morphology [10]”.
2) Line 499-502: We deleted “However, the advancement of sex-controlled breeding has faced limitations due to various factors, such as low throughput of the above methods, cumbersome experimental procedures, expensive development cost, high rate of false positives, unstable markers, and inconvenient on-site testing.”.
3) Line 506-512: We revised the “In this study, O. fasciatus with the X1X1X2X2/X1X2Y system served as the focal point [44-46, 48]. By utilizing the assembled genome information of male and female individuals acquired through PacBio sequencing, along with data from the short-read sequencing data and long-read PacBio sequencing data, we extensively employed genome-wide variant sites multiple scanning and identification, STS long reads depth evaluation and ranking, high-throughput design of primers for the target regions, and e-PCR batch amplification.” as “Our study focused on O. fasciatus with the X1X1X2X2/X1X2Y system [44-46, 48]. We used the assembled genome information of male and female individuals, along with short-read and long-read data, to identify variant sites and evaluate read depth. We also designed primers for target regions and performed batch amplification using e-PCR (Figure 1, Figure 2, Figure 3, Table 2, Table 3).”
4) Line 512-517: We deleted “By employing a comprehensive approach involving genome-wide variant site

scanning, high-throughput primer design within the desired region, and e-PCR batch amplification and validation methodologies, we successfully exploited a catalog encompassing insertion/deletion loci in large segments (>100 bp) for both male and female O. fasciatus".
5) Line 546-549: We deleted "However, larger genomic structural variants, which encompassed genetic variations not fully captured by SNPs and InDels, are closely linked to biological phenotypes and might significantly influence phenotypic traits, disease susceptibility, and adaptive capacity [79].".
4/6. I still think it is an odd choice to present deletions and insertions in different units.
Reply :
Thank you for your professional opinion. As you pointed out in your previous comments, insertions are summarised by the number of events, while deletions are summarised by nucleotide length, as we have explained in our previous presentation in the figure and table notes. We know that an insertion event is as a locus, which may exist in an intronic, exonic or intergenic region, and presents the character of a single functional region. However, deletions are different, as the size of deletion sequences varies, there is uncertainty in the functional regions of the genome, and a very large deletion site may span both intronic and exonic regions or even intergenic regions. In order to avoid the confusion of the statistics, we have determined through consultation that we use site counts for insertion events and sliding window counts for deletion sequences to count the number of deletions. Of course, we also carried out subsequent studies and validation of functional genomics of insertion deletion variants, and we will consider the statistics separately in the subsequent studies. Thank you again for your professional opinion.
5. What I meant is that the joint between chr F8 and F10 can be used to generate a PCR fragment in males, but not in females. This is not a major issue, it was just a suggestion.
Reply :
Thank you for your professional opinion. As you mentioned, we also focused on the fusion region of the two chromosomes and performed a variant site scan, and we found that the fusion region had significant structural variation, but it was difficult for us to use it as a region for genetic sex marker development due to the inefficiency of amplification caused by the drastic variation. We found through variant site scanning that abundant variant sites also exist in the inverted region, which can be used as a stable region for sex marker exploitation.
Figure (Please check the Reply point-to-point)
7. Thank you for the explanation, PCR constraints are plausible. But that would be good to highlight in the text. And it does not explain the imbalance in Table 5? Any deletion in females should be an insertion in males, and vice versa? If it is detectable from one perspective, then why not from the other?
Reply :
Thanks for your professional comments. As you said any deletion in females should be an insertion in males. In conjunction with your concerns we have found through technical discussion that the number of loci for insertions is 4422 using the female as the reference male comparison, so in turn the number of deletions occurring when using the male as the reference female comparison should be close to 4422, which we have detected as 4385. The number of deletion sites is 4675 when using female as the reference male comparison, and conversely, the number of insertion sites is 4615 when using male as the reference female comparison, so that the number of insertion and deletion sites in our results are almost balanced under different reference groups.
Meanwhile, we also explained the differences in the efficiency of PCR high-throughput amplification primer design for insertion and deletion sites in the paper as follows: "Variations in the efficacy of PCR high-pass primer design at insertion and deletion sites may be attributed to disparities in structural characteristics and GC content within the regions flanking the insertion/deletion variants.".
Table 5. Statistics of large-segment differential loci after primer design, e-PCR amplification, and simulated amplification products and size screening.
8. Fig 3e/f is clearer now.
Reply :
Thanks for your previous comments on our manuscript, which have been very helpful in improving the quality of our manuscript.

9. OK
Reply :
Thanks for your previous comments on our manuscript, which have been very helpful in improving the quality of our manuscript.

10/11. I think this figure still needs some work. The legend says that PacBio reads are in red, but I see both pink and blue (I think fwd/reverse? Also sort of suggests male/female). These IGV(ish) screenshots are not very clear, please explain a bit more. Why just the ~25 nt stretches? Are the dashed lines the deletions (I guess not)? The PacBio reads suggest a deletion in half the reads, which fits the story, but those dark blue scribbles are hard to see. Do we see entire reads, or just aligned portions?

Reply：

Thanks for your professional comments. We are very sorry for the confusion caused by our description. Our comparison strategy here is to use the female genome as a reference male for comparison. As we mentioned before the sex chromosome system of female fish is X1X1X2X2 and the sex chromosome system of male fish is X1X2Y, so there will be dimorphism in the high throughput sequenced reads of male fish, especially in the inverted region, and this phenomenon will be more obvious. Because PacBio's CLR sequencing method is used, there is no problem with bidirectional sequencing. However, because we used male and female fish with inverted structural variants, the phenomenon of matching direction was generated. We are sure that the pink and blue reads you see should be the reflection of the inversion of the male reads with the female fish as the reference. We have changed the content of CLR reads described in the figure to pink and blue to represent the same and opposite orientation of the female genome.

As you can see and as we provide the IGV insertion results plot below, since we have consolidated representative results in the manuscript, we have intercepted a portion of the sequence that contains the insertion deletion site and both ends in order to reflect the presence of the insertion and deletion events clearly. The dotted line you see is the base pair baseline, which appears as a single dotted line from a distance, and when zoomed in is two dotted lines corresponding to a single base. The blue scribble you see is actually the result of the size of the insertion fragment as shown below. In order to fully demonstrate this result of ours, we have changed the vertical orientation to horizontal.

Figure (Please check the Reply point-to-point)

Line 553: Prvious -> Previous

Reply：

Thanks for your detailed comments. We have revised the “PrEvious” as “Previous”.
