# Supplementary material for: Innovative approach for high-throughput exploiting sex-specific markers in Japanese parrotfish Oplegnathus fasciatus
Source: Gigascience. 2024 Jul 19;13:giae045. doi: 10.1093/gigascience/giae045 (PMC11258905; doi:10.1093/gigascience/giae045)
Supplement: giae045_Reviewer_1_Report_Original_Submission — Syed Farhan Ahmad -- 1/23/2024 Reviewed [file giae045_reviewer_1_report_original_submission.pdf]

Reviewer Report

Title: Innovative approach for high-throughput exploiting sex-specific markers in Japanese parrotfish *Oplegnathus fasciatus*

Version: Original Submission **Date:** 1/23/2024

Reviewer name: Syed Farhan Ahmad

Reviewer Comments to Author:

In this manuscript, Xiao et al performed bioinformatic analysis of Illumina and PacBio sequencing data to detect insertion/deletion variants, coupled with PCR based experimental validation to enable identification of genetic sex markers in a fish species. Overall, the manuscript main strength is the findings of sex specific markers in both male and female genomes as clearly supported by PCR results. But there are still a few shortcomings that needs to be addressed. Below I explain my major points:

In this manuscript, the authors assert that they have created a database of insertion/deletion-based sex-markers. After reviewing, it appears that this claim may be overselling their findings. In bioinformatics, the term "database" typically refers to an online portal for users to explore data. Therefore, it is suggested to consider revising the term "database" to "variants catalog" for clarity and accuracy.

In addition to mapping Illumina and PacBio reads against references, did the author also consider to directly map both male and female genomes using pairwise whole-genome aligner (for example LASTZ) to see the differences in both genomes? Additionally, I did not find any relevant information in the manuscript on the quality of reference genomes assemblies for male and female fish used for the reads alignments. Authors should clarify whether or not the variants detection was affected by any possible artifacts in the genome assemblies?

For the efficiency of long-read based sex-specific markers, authors should clarify how many variants were experimentally validated ? Were there any cases of false-positive variants?

In the manuscript's abstract, there are numerous undefined abbreviations and technical terms. Authors should revise the abstract to minimize the use of jargon. For example:

Line 36-37: "first-generation development methods such as RFLP, 37 RAPD, SSR, and AFLP as well as second-generation techniques utilizing Illumina's 38 SNP/InDel markers."

It is confusing molecular biology techniqies with next generation sequencing (NGS) techniques.

Line 45: "Illumina survey" can be revised "short-read sequencing data survey"

Line 46: "PacBio CLR / CCS" can be revised "long-read PacBio sequencing"

The methods described in this manuscript is not clear and missing details. how many samples were sequenced using Illumina and PacBio? How was the sequencing data preprocessed for QC, poor reads filtering and adapter trimming? What source, database and accession ID was the reference genomes of male and female downloaded? It is mentioned that aligner BWA (v 0.7.8) and Minimap2 were used, but as could not follow which aligner map Illumina or PacBio reads?

One of my main concerns is the simulated e-PCR. I am not sure, how much this technique is reliable for validation studies and what is the rate of false positive/negative relative to the actual experimental PCR

technique. My question to authors, Are, there any previous studied that have utilized the e-PCR amplified fragment sizes for genome variants validation? If yes, it is recommended to cite with brief description of reliability of this e-PCR versus normal PCR in lab.
The gene otology enrichment findings seems to be out of context from the scope of the manuscript. The authors described the enriched GO terms for the exonic variants detected in their analysis, but there is no discussions for the meaningful biology of these functions and most of these results are just descriptive reports without further mechanisms and consequences explanation.
The figures captions are not clearly described to explain the data. For example, in figure 3,
Line 251-252, what is meaning of different alignments patterns in dotplots? All the captions should be explanatory for the data plotted in figures.
Some figure (Figure 7), have unnecessary portion with alignment browser screen, it should be cropped or manually edited for better visualization.
Minor comments:
The use of term "linear genomic information" is not very clear. Authors should clearly explain what they mean by linear genome? Whether this term mean genome assembly or comparative genomic analysis?
On Line 147, "Using the difference database" should be corrected "Using different databases"

Methods

Are the methods appropriate to the aims of the study, are they well described, and are necessary controls included? Choose an item.

Conclusions

Are the conclusions adequately supported by the data shown? Choose an item.

Reporting Standards

Does the manuscript adhere to the journal's guidelines on minimum standards of reporting? Choose an item.

Choose an item.

Statistics

Are you able to assess all statistics in the manuscript, including the appropriateness of statistical tests used? Choose an item.

Quality of Written English

Please indicate the quality of language in the manuscript: Choose an item.

Declaration of Competing Interests

Please complete a declaration of competing interests, considering the following questions:

- Have you in the past five years received reimbursements, fees, funding, or salary from an organisation that may in any way gain or lose financially from the publication of this manuscript, either now or in the future?
- Do you hold any stocks or shares in an organisation that may in any way gain or lose financially from the publication of this manuscript, either now or in the future?
- Do you hold or are you currently applying for any patents relating to the content of the manuscript?
- Have you received reimbursements, fees, funding, or salary from an organization that holds or has applied for patents relating to the content of the manuscript?
- Do you have any other financial competing interests?
- Do you have any non-financial competing interests in relation to this paper?

If you can answer no to all of the above, write 'I declare that I have no competing interests' below. If your reply is yes to any, please give details below.

I declare that I have no competing interests

I agree to the open peer review policy of the journal. I understand that my name will be included on my report to the authors and, if the manuscript is accepted for publication, my named report including any attachments I upload will be posted on the website along with the authors' responses. I agree for my report to be made available under an Open Access Creative Commons CC-BY license (http://creativecommons.org/licenses/by/4.0/). I understand that any comments which I do not wish to be included in my named report can be included as confidential comments to the editors, which will not be published.

Choose an item.

To further support our reviewers, we have joined with Publons, where you can gain additional credit to further highlight your hard work (see: https://publons.com/journal/530/gigascience). On publication of this paper, your review will be automatically added to Publons, you can then choose whether or not to claim your Publons credit. I understand this statement.

Yes Choose an item.
