# Supplementary material for: Innovative approach for high-throughput exploiting sex-specific markers in Japanese parrotfish Oplegnathus fasciatus
Source: Gigascience. 2024 Jul 19;13:giae045. doi: 10.1093/gigascience/giae045 (PMC11258905; doi:10.1093/gigascience/giae045)
Supplement: giae045_Reviewer_2_Report_Original_Submission — Frederic Brunet -- 1/29/2024 Reviewed [file giae045_reviewer_2_report_original_submission.pdf]

Reviewer Report

Title: Innovative approach for high-throughput exploiting sex-specific markers in Japanese parrotfish Oplegnathus fasciatus

Version: Original Submission **Date:** 1/29/2024

Reviewer name: Frederic Brunet

Reviewer Comments to Author:

Dear colleagues,
Than you very much for giving me the opportunity to read and comment the article of Xiao et al., entitled "A novel and scalable method for high-throughput and accurate exploitation of sex-specific markers: a case study on Oplegnathus fasciatus".
Most Eukaryotes do sex, and pluricellular organisms have highly differentiated organs, using sex determination systems and sex differentiation genes. In mammals, sex chromosomes are highly dimorphic, while most fish genomes show no distinction of their sexual chromosomes, and the genes responsible for the sex determination have been hard to characterize, sometimes being located in regions full of repeated sequences. Also, if sexual organs are easy to visualize and are used for chick sexing at just one day old in the egg industry (male chicks are culled as they are an unwanted by-product of egg production), such a selection can be harder to do in fish. Sexing the striped beakfish (Oplegnathus fascinatus), a major in Asian fish farm industry, is less easy, and the sexual differentiation can only be observed once the animals have passed the 500 g weight size, when males start to grow faster that females, bringing better yields. Among the different methods and technics used to sex the individuals in the past, none were really conclusive. The authors propose to take advantage of the long reads provided with the third generation sequencing to access a class of mutations that are hundred-bp long insertions and deletions, not taken much into consideration before then. They were able to find occurrences of long indels that could be used as proxies of genetic sex markers.
In this work, the authors do present convincing technics and results. However, in that framework, it can be expected that the authors will also provide the weaknesses of this technic. Here, I see at least two points that should be considered. The first one is related to population genetics and the second should deal with the origin, if any, of these deletions and insertions, and their stability.
Starting with the second, what is the origin of such insertions and deletions? Could it be due to the mobilisation of a transposable element? Or any hot spot of deletion due for example to the formation of a fragile non-conventional B-DNA? Is there any repetitive sequence? Could these deletions or insertions occur frequently?
The first question deals with the level of polymorphism of these indels. How frequent are these insertions/deletions comparing different geographic populations?
I understand that these two points are not the main interest of the present article. However, these evolutionary considerations would deserve to be taken into account, at least correctly addressed in the discussion.
Best regards,

A (non exhaustive) list of suggestions:
Title. I'd suggest to shorten it and use the vernacular name, at least, or explicitly say that this species is a fish.
Abstract, it is not mentioned on what taxonomic species this article is dealing with.
Figures: font size is too small.
Fig. 4, 5 and others would gain in being more didactical and summarized in one single image. These detailed information would be more valorized as
Tables: position numbers using decimal tabulations and homogenize filled cells, text shouldn't be in the "justify" format
L 316 "bp base" is confusing.
L 486-488: This claim cites no reference. Why large sequences shouldn't evolve at random, unless of course, if they are directly linked to functional genes involved in various, but essential processes?
Fig 5d check spelling of nucleotide (here spelled "nucelotide"). Same for Fig. S2e.
Fig8 b, identity is not correctly positioned below line "chrY" 380
Mat&Meth
Maybe worth to subdivide into paragraphs (and titles).
Write large number with separation, being coma (as in Result section) or small space
L411: "REREgulation" is that correct?
References output has not been scrutinized. For example, almost no Journals are cited!!!
Species names should also be italicized in the list of references!

Methods

Are the methods appropriate to the aims of the study, are they well described, and are necessary controls included? Choose an item.

Conclusions

Are the conclusions adequately supported by the data shown? Choose an item.

Reporting Standards

Does the manuscript adhere to the journal's guidelines on minimum standards of reporting? Choose an item.

Choose an item.

Statistics

Are you able to assess all statistics in the manuscript, including the appropriateness of statistical tests used? Choose an item.

Quality of Written English

Please indicate the quality of language in the manuscript: Choose an item.

Declaration of Competing Interests

Please complete a declaration of competing interests, considering the following questions:

- Have you in the past five years received reimbursements, fees, funding, or salary from an organisation that may in any way gain or lose financially from the publication of this manuscript, either now or in the future?
- Do you hold any stocks or shares in an organisation that may in any way gain or lose financially from the publication of this manuscript, either now or in the future?
- Do you hold or are you currently applying for any patents relating to the content of the manuscript?
- Have you received reimbursements, fees, funding, or salary from an organization that holds or has applied for patents relating to the content of the manuscript?
- Do you have any other financial competing interests?
- Do you have any non-financial competing interests in relation to this paper?

If you can answer no to all of the above, write 'I declare that I have no competing interests' below. If your reply is yes to any, please give details below.

I declare that I have no competing interests.

I agree to the open peer review policy of the journal. I understand that my name will be included on my report to the authors and, if the manuscript is accepted for publication, my named report including any attachments I upload will be posted on the website along with the authors' responses. I agree for my report to be made available under an Open Access Creative Commons CC-BY license (http://creativecommons.org/licenses/by/4.0/). I understand that any comments which I do not wish to be included in my named report can be included as confidential comments to the editors, which will not be published.

Choose an item.

To further support our reviewers, we have joined with Publons, where you can gain additional credit to further highlight your hard work (see: https://publons.com/journal/530/gigascience). On publication of this paper, your review will be automatically added to Publons, you can then choose whether or not to claim your Publons credit. I understand this statement.

Yes Choose an item.
