# Supplementary material for: Innovative approach for high-throughput exploiting sex-specific markers in Japanese parrotfish Oplegnathus fasciatus
Source: Gigascience. 2024 Jul 19;13:giae045. doi: 10.1093/gigascience/giae045 (PMC11258905; doi:10.1093/gigascience/giae045)
Supplement: giae045_Reviewer_2_Report_Revision_1 — Frederic Brunet -- 4/23/2024 Reviewed [file giae045_reviewer_2_report_revision_1.pdf]

Reviewer Report

Title: Innovative approach for high-throughput exploiting sex-specific markers in Japanese parrotfish Oplegnathus fasciatus

Version: Revision 1 **Date:** 4/23/2024

Reviewer name: Frederic Brunet

Reviewer Comments to Author:

Dear colleagues,
Thank you very much for the thorough, extensive, cautious and precise replies to the previous round of comments that I provide, as well as that of the two other reviewers in my opinion.
I just have a list of minor comments to provide.
Best regards,
Being a non-native English speaker, please double-check my comments below.
Minor comments:
In the first paragraph of the new version (starting line 125), the use of the present tense was better than that of the actual paste tense (also line 195, the use of "was" would mean that the species no longer exists in this geographical area). L228, it is still the same sex chromosome system. Line 683, etc. Same for the Conclusion final paragraph, the present tense should be used, or the present perfect, but not the preterit.
Watch out the terminology used: "sex research (line 126)" deal with other fields of research (see for example the content of the "journal of sex research") and you might want to use a more specific wording, example given:
sexual reproduction, sex chromosomes, sex determinism and differentiation, sexual dimorphism, genetics/genomics of sex...
L246: Table 1":" (is missing) and put the number in an easy format (e.g. align right, number display)
L288: number display homogeneity. (an line 228 page 157
Why not using parrotfish instead of O. fasciatus along the text, tables and figures?
L765: PrEvious
Line 244 page 157: Two consecutive blank spaces
Try not to use scientific names and vernacular ones in the same sentence (e.g. Drosophila melanogaster, Caenorhabditis elegans, mouse, human, and Arabidopsis thaliana)
again, check homogeneity of occurrence of empty character between a value and its unit (eg line 768: 40Mb with no space, as other values has one.
Also line 590 and 591: a 965-kb deletion and a 4.8 Mb deletion, please homogenize.
Watch out that transposons are only class II (DNA) transposable elements. I guess that the authors were talking about Transposable Elements (TEs)

Methods

Are the methods appropriate to the aims of the study, are they well described, and are necessary controls included? Choose an item.

Conclusions

Are the conclusions adequately supported by the data shown? Choose an item.

Reporting Standards

Does the manuscript adhere to the journal's guidelines on [minimum standards of reporting?]() Choose an item.

Choose an item.

Statistics

Are you able to assess all statistics in the manuscript, including the appropriateness of statistical tests used? Choose an item.

Quality of Written English

Please indicate the quality of language in the manuscript: Choose an item.

Declaration of Competing Interests

Please complete a declaration of competing interests, considering the following questions:

- Have you in the past five years received reimbursements, fees, funding, or salary from an organisation that may in any way gain or lose financially from the publication of this manuscript, either now or in the future?
- Do you hold any stocks or shares in an organisation that may in any way gain or lose financially from the publication of this manuscript, either now or in the future?
- Do you hold or are you currently applying for any patents relating to the content of the manuscript?
- Have you received reimbursements, fees, funding, or salary from an organization that holds or has applied for patents relating to the content of the manuscript?
- Do you have any other financial competing interests?
- Do you have any non-financial competing interests in relation to this paper?

If you can answer no to all of the above, write 'I declare that I have no competing interests' below. If your reply is yes to any, please give details below.

I declare that I have no competing interests.

I agree to the open peer review policy of the journal. I understand that my name will be included on my report to the authors and, if the manuscript is accepted for publication, my named report including any attachments I upload will be posted on the website along with the authors' responses. I agree for my

report to be made available under an Open Access Creative Commons CC-BY license (http://creativecommons.org/licenses/by/4.0/). I understand that any comments which I do not wish to be included in my named report can be included as confidential comments to the editors, which will not be published.

Choose an item.

To further support our reviewers, we have joined with Publons, where you can gain additional credit to further highlight your hard work (see: https://publons.com/journal/530/gigascience). On publication of this paper, your review will be automatically added to Publons, you can then choose whether or not to claim your Publons credit. I understand this statement.

Yes Choose an item.
