# Supplementary material for: Innovative approach for high-throughput exploiting sex-specific markers in Japanese parrotfish Oplegnathus fasciatus
Source: Gigascience. 2024 Jul 19;13:giae045. doi: 10.1093/gigascience/giae045 (PMC11258905; doi:10.1093/gigascience/giae045)
Supplement: giae045_Reviewer_3_Report_Original_Submission — Christiaan Henkel -- 1/30/2024 Reviewed [file giae045_reviewer_3_report_original_submission.pdf]

Reviewer Report

Title: Innovative approach for high-throughput exploiting sex-specific markers in Japanese parrotfish Oplegnathus fasciatus

Version: Original Submission **Date:** 1/30/2024

Reviewer name: Christiaan Henkel

Reviewer Comments to Author:

This manuscript describes a computational approach to develop PCR markers for sex chromosomes in a fish with a complex genetic sex determination system. A few of these markers have been validated using PCR on a panel of 24 fish for which the sex was determined by dissection, showing an excellent correspondence.

I have found some issues that I would ask the authors to address:

1. It is not entirely clear to me what data the study is based on. The two genome assemblies are not referenced by accession number, and the validation data (PacBio) are not described. Are these the data from the original genome assemblies?
2. The Introduction is outdated. The state of the art in long-read sequencing as described in the Introduction is at least 5 years old; the mention of the human genome with a contig N50 of 8.3 Mb (line 118) should rather be replaced by the more recent telomere to telomere efforts; the single reference (26) to a random fish genome is similarly out of place. There are far more than 40 (line 101) fish species that have had their genome sequenced. And finally, the inclusion of Helicos (111) as a mature long-read platform is strange - the company disappeared over a decade ago, and although their platform was single-molecule, it certainly didn't produce long reads.
3. The Discussion is severely outdated. All references to structural variation are from the dawn of the genomics era, and even older: ref 68, 69: 1991, ref 70-72: 2001, ref 73: 2006, ref 74: 2003. Structural variation in genomes is a major research theme these days, and the Discussion should acknowledge that by placing the results in the context of recent knowledge.
4. Results and Figure 2/Table 1: needs to be presented more clearly. The first reference to fig 2 (line 218) does instead refer to the unfiltered data. Insertions (panels a, c) are summarized by number of events, deletions (b, d) by length in nucleotides. The data in fig 2d is not described in the text.
5. Figure 3a/b: There is a huge inversion between female chr 8 and male chr 9. Did you consider using this type of structural variation to design PCR tests?
6. Figure 3c/d: According to the legend, ring 2 (from outside) are insertions, and ring 3 deletions. There should be some balance between these two between the two contrasts. Now is seems as in both contrasts there are far fewer deletions than insertions, which is implausible. Also, the corners of the white background of the fish image block out some data.
7. Similar issue: In table 2, insertions and deletions are balanced. However, after primer design there are far fewer deletions. How can this be explained?
8. Figure 3e/f: The asymmetry between the chromosomes is quite interesting, i.e. that some are prone to deletions and others to insertion at a whole-chromosome scale. However, please check these data;

the figure as it is cannot be an accurate representation, as it should be symmetrical between the two panels. Meaning that an insertion in the male genome should be a deletion in the female, vv. This is not the case for chr 15F/14M and 19F/18M.
9. Primer design, line 262 and further: what were the criteria? What were the PCR settings? Also, 264: 'designed high-throughput primers to construct high-throughput primer datasets', please rephrase.
10. Figure 4: is different in the inline version (p14) from the attached (p36). Also identical to figure S1.
11. Figures 4-8 consist mostly of screenshots that are not clearly explained, and not intuitively very informative. What am I looking at?
12. Enrichment analysis (l 365-426): This text consist almost entirely of a listing of categories that are also printed in the supplementary figures. As there is no clear conclusion to, or discussion of these results, I would suggest leaving the text out entirely.
Some minor/textual remarks:
Lines 63-67 in the Abstract: repeats the same information twice.
Line 151-152: same statement twice.
Line 156: PCR electron simulation -> simulated PCR.
Line 157: primer -> primer pair.
Figure 1: Typos Illuminar, onele thousand (?)
Line 179: Was BWA used for Illumina and Minimap2 for PacBio? Minimap2 needs a version number.

Methods

Are the methods appropriate to the aims of the study, are they well described, and are necessary controls included? Choose an item.

Conclusions

Are the conclusions adequately supported by the data shown? Choose an item.

Reporting Standards

Does the manuscript adhere to the journal's guidelines on [minimum standards of reporting?]() Choose an item.

Choose an item.

Statistics

Are you able to assess all statistics in the manuscript, including the appropriateness of statistical tests used? Choose an item.

Quality of Written English

Please indicate the quality of language in the manuscript: Choose an item.

Declaration of Competing Interests

Please complete a declaration of competing interests, considering the following questions:

- Have you in the past five years received reimbursements, fees, funding, or salary from an organisation that may in any way gain or lose financially from the publication of this manuscript, either now or in the future?
- Do you hold any stocks or shares in an organisation that may in any way gain or lose financially from the publication of this manuscript, either now or in the future?
- Do you hold or are you currently applying for any patents relating to the content of the manuscript?
- Have you received reimbursements, fees, funding, or salary from an organization that holds or has applied for patents relating to the content of the manuscript?
- Do you have any other financial competing interests?
- Do you have any non-financial competing interests in relation to this paper?

If you can answer no to all of the above, write 'I declare that I have no competing interests' below. If your reply is yes to any, please give details below.

I declare that I have no competing interests

I agree to the open peer review policy of the journal. I understand that my name will be included on my report to the authors and, if the manuscript is accepted for publication, my named report including any attachments I upload will be posted on the website along with the authors' responses. I agree for my report to be made available under an Open Access Creative Commons CC-BY license (http://creativecommons.org/licenses/by/4.0/). I understand that any comments which I do not wish to be included in my named report can be included as confidential comments to the editors, which will not be published.

Choose an item.

To further support our reviewers, we have joined with Publons, where you can gain additional credit to further highlight your hard work (see: https://publons.com/journal/530/gigascience). On publication of this paper, your review will be automatically added to Publons, you can then choose whether or not to claim your Publons credit. I understand this statement.

Yes Choose an item.
