# Supplementary material for: Innovative approach for high-throughput exploiting sex-specific markers in Japanese parrotfish Oplegnathus fasciatus
Source: Gigascience. 2024 Jul 19;13:giae045. doi: 10.1093/gigascience/giae045 (PMC11258905; doi:10.1093/gigascience/giae045)
Supplement: giae045_Reviewer_3_Report_Revision_1 — Christiaan Henkel -- 5/14/2024 Reviewed [file giae045_reviewer_3_report_revision_1.pdf]

Reviewer Report

Title: Innovative approach for high-throughput exploiting sex-specific markers in Japanese parrotfish Oplegnathus fasciatus

Version: Revision 1 **Date:** 5/14/2024

Reviewer name: Christiaan Henkel

Reviewer Comments to Author:

I thank the authors for their revision. Especially the inclusion of the actual genome assemblies this work is based on is important.
Below I return to my comments on the previous version:
1. Thank you for the addition. One comment: in the Sample collection, please rephrase line 191: HQ DNA was not extracted by Illumina/PacBio, but analyzed?
2-3. Thank you for updating. I would suggest summarizing a bit more in the discussion, as it is very lengthy and some information is presented twice. Line 543: I would replace 'genomic structural variation' with just 'genomic variation', as you include SNPs under this definition.
4/6. I still think it is an odd choice to present deletions and insertions in different units.
5. What I meant is that the joint between chr F8 and F10 can be used to generate a PCR fragment in males, but not in females. This is not a major issue, it was just a suggestion.
7. Thank you for the explanation, PCR constraints are plausible. But that would be good to highlight in the text. And it does not explain the imbalance in Table 5? Any deletion in females should be an insertion in males, and vice versa? If it is detectable from one perspective, then why not from the other?
8. Fig 3e/f is clearer now.
9. OK
10/11. I think this figure still needs some work. The legend says that PacBio reads are in red, but I see both pink and blue (I think fwd/reverse? Also sort of suggests male/female). These IGV(ish) screenshots are not very clear, please explain a bit more. Why just the ~25 nt stretches? Are the dashed lines the deletions (I guess not)? The PacBio reads suggest a deletion in half the reads, which fits the story, but those dark blue scribbles are hard to see. Do we see entire reads, or just aligned portions?
Line 553: Prvious -> Previous

Methods

Are the methods appropriate to the aims of the study, are they well described, and are necessary controls included? Choose an item.

Conclusions

Are the conclusions adequately supported by the data shown? Choose an item.

Reporting Standards

Does the manuscript adhere to the journal's guidelines on [minimum standards of reporting?]() Choose an item.

Choose an item.

Statistics

Are you able to assess all statistics in the manuscript, including the appropriateness of statistical tests used? Choose an item.

Quality of Written English

Please indicate the quality of language in the manuscript: Choose an item.

Declaration of Competing Interests

Please complete a declaration of competing interests, considering the following questions:

- Have you in the past five years received reimbursements, fees, funding, or salary from an organisation that may in any way gain or lose financially from the publication of this manuscript, either now or in the future?
- Do you hold any stocks or shares in an organisation that may in any way gain or lose financially from the publication of this manuscript, either now or in the future?
- Do you hold or are you currently applying for any patents relating to the content of the manuscript?
- Have you received reimbursements, fees, funding, or salary from an organization that holds or has applied for patents relating to the content of the manuscript?
- Do you have any other financial competing interests?
- Do you have any non-financial competing interests in relation to this paper?

If you can answer no to all of the above, write 'I declare that I have no competing interests' below. If your reply is yes to any, please give details below.

I declare that I have no competing interests

I agree to the open peer review policy of the journal. I understand that my name will be included on my report to the authors and, if the manuscript is accepted for publication, my named report including any attachments I upload will be posted on the website along with the authors' responses. I agree for my report to be made available under an Open Access Creative Commons CC-BY license (http://creativecommons.org/licenses/by/4.0/). I understand that any comments which I do not wish to be included in my named report can be included as confidential comments to the editors, which will not be published.

Choose an item.

To further support our reviewers, we have joined with Publons, where you can gain additional credit to further highlight your hard work (see: https://publons.com/journal/530/gigascience). On publication of this paper, your review will be automatically added to Publons, you can then choose whether or not to claim your Publons credit. I understand this statement.

Yes Choose an item.
